# Hydrogen sulfide treatment at the late growth stage of *Saccharomyces cerevisiae* extends chronological lifespan

**DOI:** 10.18632/aging.202738

**Published:** 2021-03-19

**Authors:** Arman Ali Shah, Binghua Liu, Zhihuai Tang, Wang Wang, Wenjie Yang, Quanjun Hu, Yan Liu, Nianhui Zhang, Ke Liu

**Affiliations:** 1 Key Laboratory of Bio-Resource and Eco-Environment of Ministry of Education, College of Life Sciences, Sichuan University, Chengdu 610065, Sichuan, China

**Keywords:** *Saccharomyces cerevisiae*, hydrogen sulfide, NaHS, aging, chronological lifespan

## Abstract

Previous studies demonstrated that lifelong treatment with a slow H_2_S releasing donor extends yeast chronological lifespan (CLS), but it is not clear when the action of H_2_S benefits to CLS during yeast growth. Here, we show that short H_2_S treatments by using NaHS as a fast H_2_S releasing donor at 96 hours after inoculation extended yeast CLS while NaHS treatments earlier than 72 hours after inoculation failed to do so. To reveal the mechanism, we analyzed the transcriptome of yeast cells with or without the early and late NaHS treatments. We found that both treatments had similar effects on pathways related to CLS regulation. Follow-up qPCR and ROS analyses suggest that altered expression of some antioxidant genes by the early NaHS treatments were not stable enough to benefit CLS. Moreover, transcriptome data also indicated that some genes were regulated differently by the early and late H_2_S treatment. Specifically, we found that the expression of *YPK2*, a human *SGK2* homolog and also a key regulator of the yeast cell wall synthesis, was significantly altered by the late NaHS treatment but not altered by the early NaHS treatment. Finally, the key role of *YPK2* in CLS regulation by H_2_S is revealed by CLS data showing that the late NaHS treatment did not enhance the CLS of a *ypk2* knockout mutant. This study sheds light on the molecular mechanism of CLS extension induced by H_2_S, and for the first time addresses the importance of H_2_S treatment timing for lifespan extension.

## INTRODUCTION

Aging is a progressive structural and functional decline in cellular components and metabolic activities, resulting in a number of chronic diseases and the death of an organism [1]. Although aging is generally considered inevitable, recent studies have revealed that aging can be slowed by many interventions [2]. A growing number of substances have been identified as potential pharmaceutical interventions to enhance longevity in a variety of organisms from yeasts to humans [3]. Mechanistic studies, which demonstrate these substances including metformin, rapamycin, NAD^+^ boosters and hydrogen sulfide target intracellular signaling pathways that modulate aging, are paving the path toward determining whether they effectively affect aging in human [4–6].

Hydrogen sulfide (H_2_S), the third gasotransmitter after nitric oxide and carbon monoxide, has gained noteworthy scientific consideration in the present era [7–9]. Traditionally, it has been known as an extremely toxic gas having a characteristic smell of rotten eggs. Its cytotoxicity relies on the interruption of intracellular metabolic activities through the down-regulation of the cytochrome c oxidase thereby inhibiting mitochondrial adenosine triphosphate (ATP) synthesis [10]. Also, physiological concentrations of H_2_S regulate vaso-relaxation by opening K_ATP_ channel [11], and by promoting angiogenesis through the activation of Akt and K_ATP_ channel/MAPK pathway [12, 13]. The H_2_S interacts with glucose and the K_ATP_ channel to control insulin secretion [14]. It act as an O_2_ sensor/transducer in vascular response to hypoxia [15]. It augments NMDA receptor-mediated responses to assist the induction of hippocampal long-term potentiation [16]. Moreover, H_2_S increases thermotolerance and lifespan in nematodes through SIR-2.1 activity [17]. In addition, H_2_S protects against neurodegeneration [18, 19], myocardial ischemia-reperfusion injury [20–22], acute inflammation [23] and hypoxia [24].

Recently, it has been demonstrated that ROS modulates lifespan at specific developmental stages [25], suggesting the importance of optimal timing for aging interventions. Considering the co-existence of cyto-protective and cytotoxic effects of H_2_S, it is especially important to understand the optimal timing of exogenous H_2_S administration to mostly slow aging. However, this information is lacking due to the use of slow H_2_S releasing donors which need to be used continuously during lifespan studies [26]. In this study, we treated *S. cerevisiae* with NaHS, a fast H_2_S releasing donor with a short half-life, at the early and late phases of growth. We found that a low level of NaHS at the late phase of growth substantially extended yeast CLS. In contrast, the NaHS treatment at the early phase was surprisingly ineffective. Moreover, the similarities and differences in the gene expression profile of both treatment cases provide new insights into the role of H_2_S in aging.

## RESULTS

### Lifespan extension by H_2_S depends on the timing of NaHS treatment

**Figure 1:**
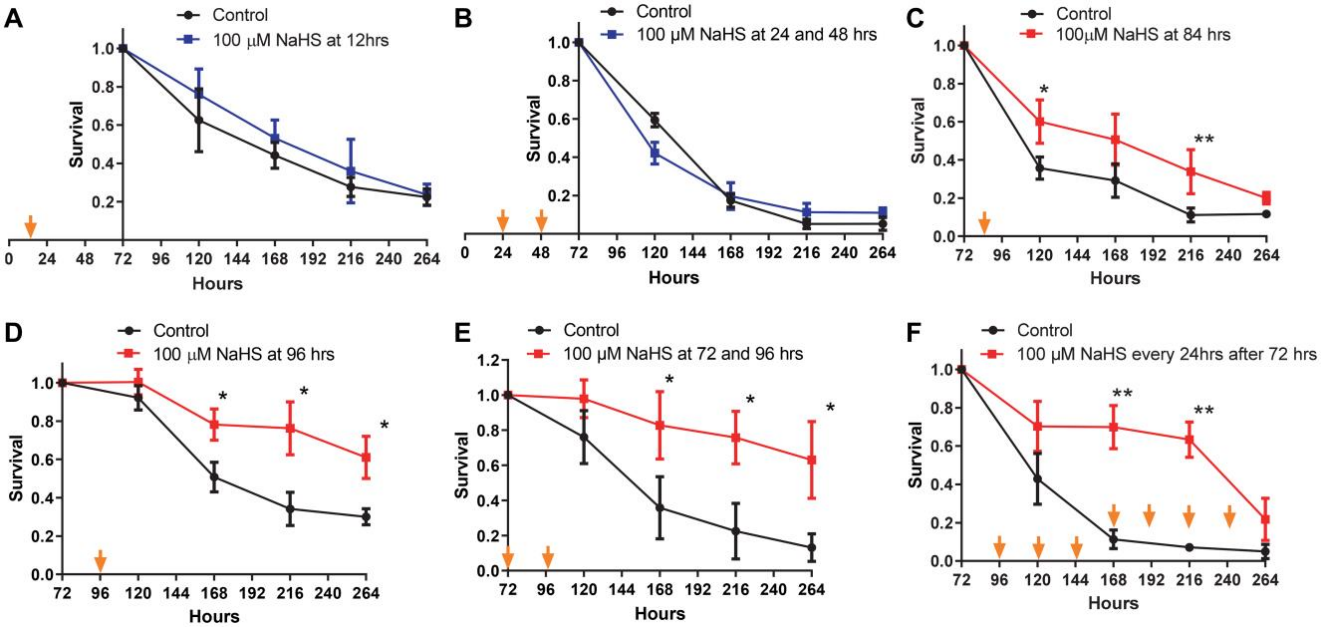
**CLS extension is dependent upon the timing of NaHS treatment.** Viabilities of cells treated without (black plots) or with 100 μM NaHS at the earlier (blue plots) or the later (red plots) phases of growth were plotted. Specifically, NaHS was added once or twice into cell cultures at the 12 hours (**A**), 24 and 48 hours (**B**), 84 hours (**C**), 96 hours (**D**), or 72 and 96 hours (**E**) after inoculation. (**F**) NaHS was added into cell cultures every 24 hours after 72 hours of inoculation. Arrows indicate the time of NaHS treatment. Triplicate cultures were used to achieve mean ± SD of viabilities. ^*^p < 0.05, ^**^p < 0.01, ^***^p < 0.001.

Previous studies have demonstrated that long term H_2_S treatment by using a combination of a slow H_2_S-releasing donor, GYY4137, and fast H_2_S releasing donor, NaHS, extends the CLS of yeast [26]. Considering the dramatic changes in the metabolic pathways of yeast cells during the growth from inoculation up to senescence, the effects of H_2_S treatment at different stages of growth are unclear. To investigate the stage of growth at which H_2_S regulates metabolic pathways to increase lifespan, the *Saccharomyces cerevisiae* strain BY4742 was treated with NaHS at different time periods during growth. While treating yeast cells with two doses of 100 μM NaHS at 24 and 48 hours after inoculation has no effect on cell growth (Supplementary Figure 1), there was also no effect in CLS with one 100 μM dose of NaHS at 12 hours or twice at 24 and 48 hours after inoculation (Figure 1A and 1B). Instead, one NaHS treatment at 84 hours after inoculation resulted in a slight but statically significant increase in CLS (Figure 1C). The extension of CLS was more robust if NaHS was added once at 96 hours or twice at 72 and 96 hours after inoculation (Figure 1D and 1E). Moreover, the day to day treatments starting from 72 hours after inoculation with 100 μM of NaHS also extended the CLS, but there was a sudden decline observed at the later stage of the lifespan (Figure 1F), probably due to the cytotoxic effect of longterm treatments. These results suggest that one or two NaHS treatments later than 72 hours after inoculation are required for CLS extension.

**Figure 2:**
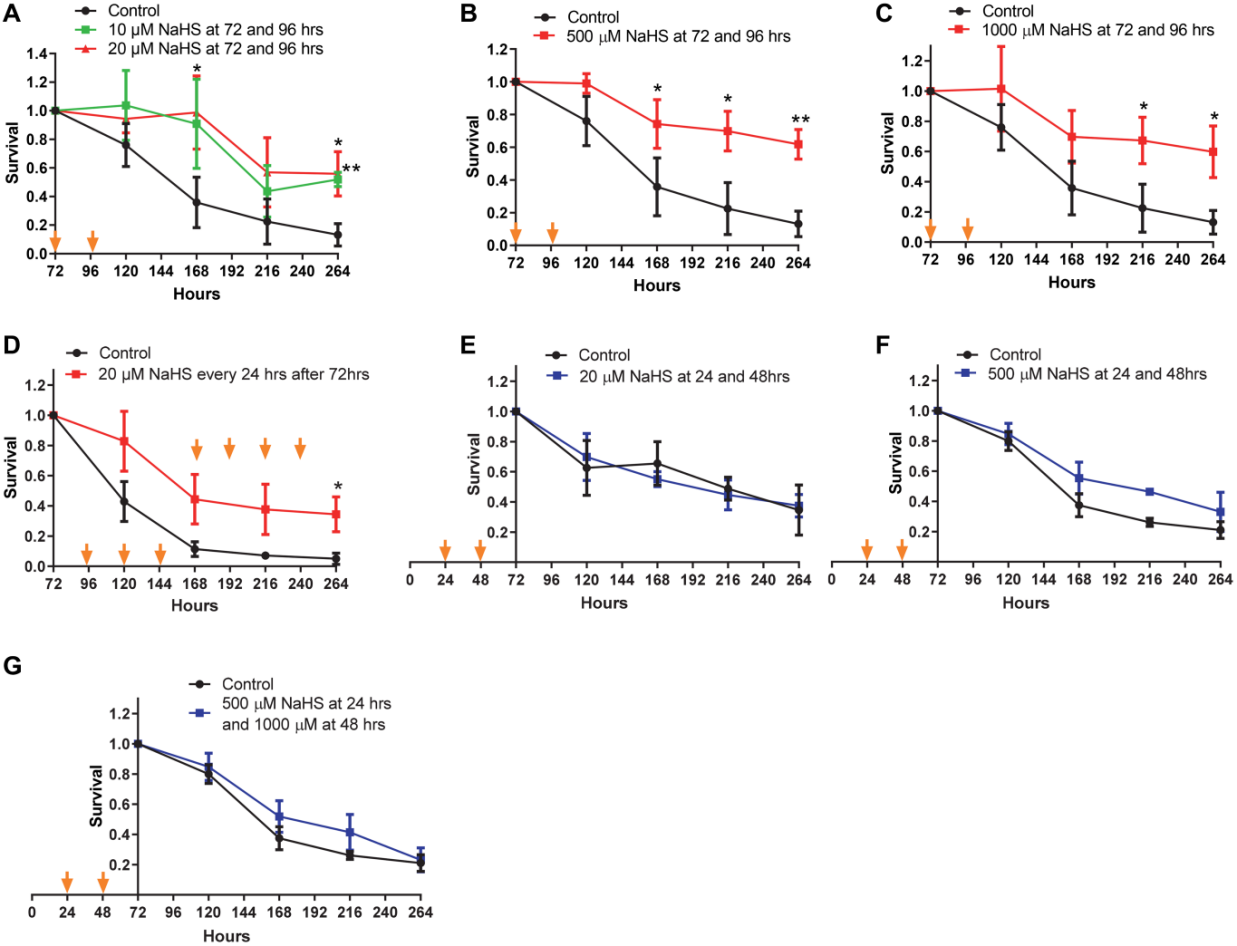
**CLS in response to various concentrations of NaHS.** Cells were treated with different concentration of NaHS at the early and the late phases of growth. (**A–G**) Viabilities of Cells treated with or without NaHS at the indicated time were plotted. Arrows indicate the time of NaHS treatment. Triplicate cultures were used to achieve mean ± SD of viabilities. ^*^p < 0.05, ^**^p < 0.01, ^***^p < 0.001.

Next, we examined if the extension of CLS was dependent on the concentration of NaHS. Two treatments with 10 or 20 μM of NaHS at 72 and 96 hours after inoculation extended lifespan (Figure 2A), although less significantly than treatments at 100 μM (comparing to Figure 1E). And treatments with 500 μM or 1000 μM of NaHS were not more beneficial for lifespan extension than treatment with 100 μM of NaHS (comparing Figure 2B and 2C to Figure 1E). The day to day treatments with 20 μM of NaHS extended the CLS without an accelerated decline in the later stage of lifespan as observed in day to day treatments with 100 μM of NaHS (comparing Figure 2D to Figure 1F), suggesting that the longterm H_2_S treatment at lower level was less toxic, while still cytoprotective. However, treatments with different concentrations of NaHS at 24 and 48 hours after inoculation did not extend CLS (Figure 2E and 2F). These data suggest that exogenous H_2_S does not extend yeast CLS if the H_2_S treatment before 72 hours of growth, which we refer to as the early H_2_S treatment. Instead, the extension of yeast CLS requires H_2_S treatment after 72 hours of growth, which we refer to as late H_2_S treatment.

### Both early and late H_2_S treatments alter the expression of a wide range of yeast genes

**Figure 3:**
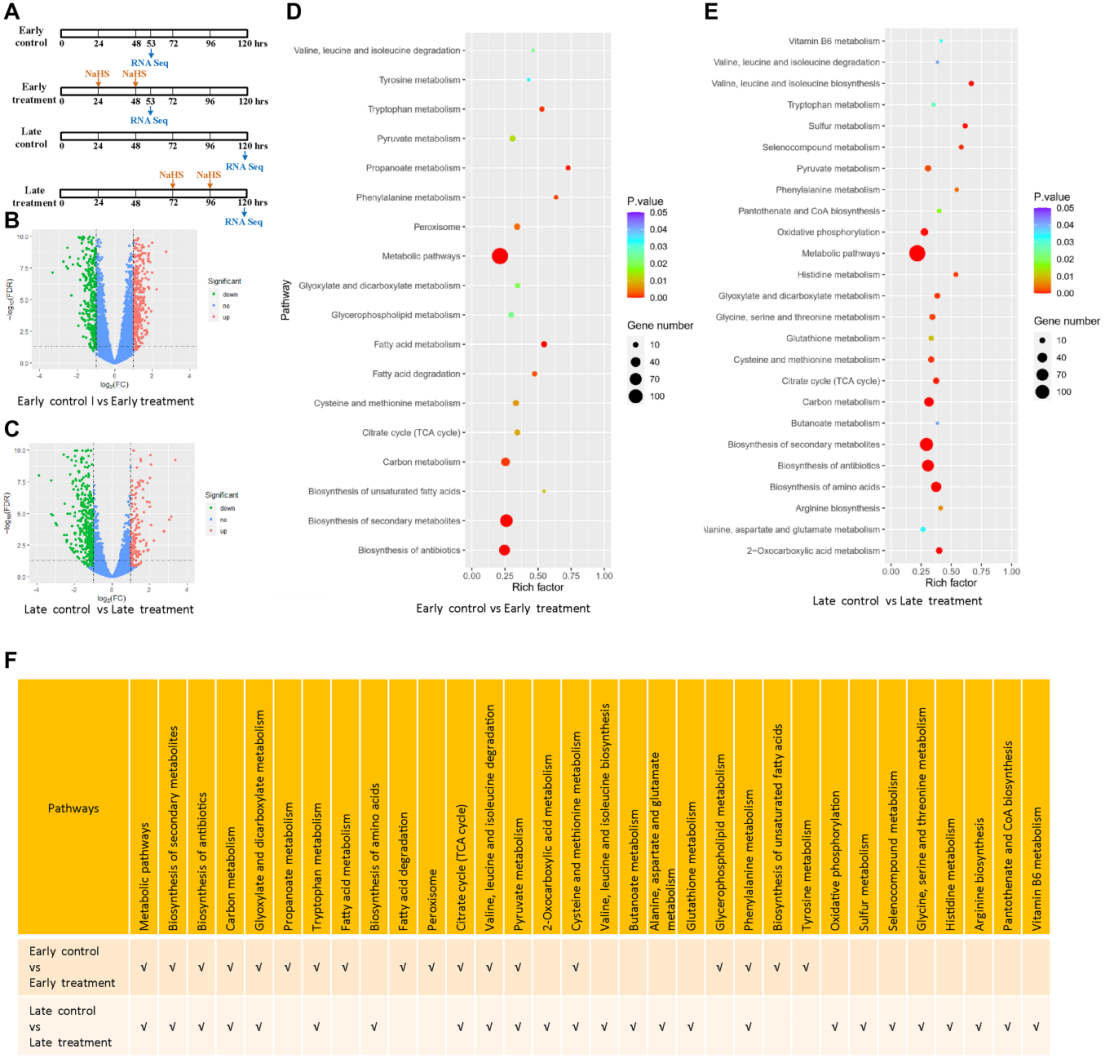
**Transcriptomic alterations by the early and late NaHS treatments.** (**A**) Schematic of the early and late NaHS treatments in relation to their untreated controls. 100 μM of NaHS were used for each dosing. (**B**, **C**) The volcano plots represent DEGs of indicated controls and treatments. (**D, E**) The bubble charts representing KEGG enrichment analysis of indicated DEGs. The rich factor indicates the degree of enrichment represented by the ratio of DEGs in a pathway to the number of total genes annotated to that pathway. (**F**) Comparison of enriched pathways in (D and E).

To gain detailed insights into the molecular mechanisms of H_2_S induced longevity in yeast; we performed RNA-seq analysis of cells with early and late NaHS treatment and their respective untreated controls (Figure 3A). Using (|log2 FC| ≥1, *p* ≤ 0.05) as the threshold, we identified the differentially expressed genes (DEGs) by comparing the gene expression profiles of the untreated controls to NaHS treated samples at two treatment time points. We identified 928 and 723 DEGs in cells treated with NaHS at the early and late stages, respectively. Among them, 408 genes were upregulated and 520 genes were downregulated in the early NaHS treatment (Figure 3B, Supplementary Table 1), whereas 202 genes were upregulated and 521 genes were downregulated in the late NaHS treatment (Figure 3C, Supplementary Table 2).

The KEGG pathway enrichment analysis reveals that both the early and late NaHS treatments have similar impacts on several pathways including biosynthesis of secondary metabolites, carbon metabolism, TCA cycle and metabolism of several amino acids (Figure 3D, 3E and 3F). These results show that early and late NaHS treatments share some gene expression changes. Late NaHS treatment does change a few pathways including oxidative phosphorylation, sulfur metabolism and metabolism of some amino acids and metabolites (Figure 3E and 3F), which may contribute to CLS extension effect of the late NaHS treatment.

**Figure 4:**
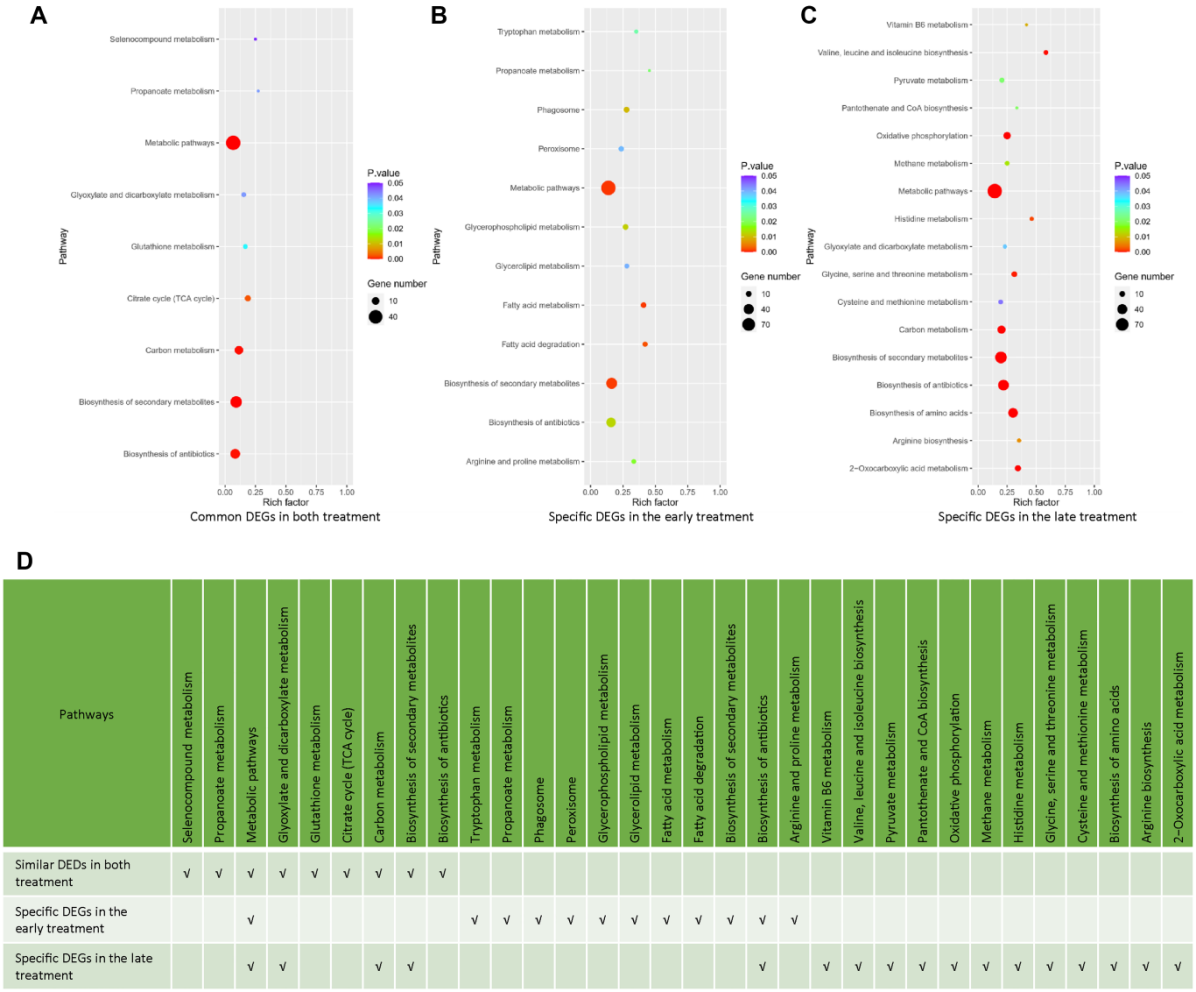
**Analysis of common and specific DEGs in the early and late NaHS treatments.** (**A–C**) The bubble charts representing KEGG enrichment analysis of common or specific DEGs in the early and late NaHS treatments. (**D**) Comparison of enriched pathways in (A–C).

To have a deeper insight to the transcriptomic effects of the early and late NaHS treatments, we identified and analyzed genes, which were altered similarly and differently by these two treatments. There are 213 DEGs common in both treatments, including 177 DEGs regulated in same direction (Supplementary Figure 2A, Supplementary Table 3) and 36 DEGs regulated oppositely (Supplementary Figure 2B, Supplementary Table 3). The remaining 715 DEGs in the early treatment (Supplementary Table 4) and 510 DEGs in the late treatments (Supplementary Table 5) are specific for those treatments, respectively. The KEGG analysis of these DEGs indicates that many metabolic pathways were similarly or specifically regulated by the early and late NaHS treatments (Figure 4A–4D). Therefore, similar to the initial analysis (Figure 3F), the KEGG analysis of DEGs expressed in same direction and DEGs specific to each treatment case also revealed the similarities and differences in the transcriptomic effects of the early and late NaHS treatments. Taken together, these data indicate that both the early and late NaHS treatments had similar and profound influences on some metabolic pathways, which may relate to CLS regulation.

### The late NaHS treatment provided more cytoprotection for life span extension

**Figure 5:**
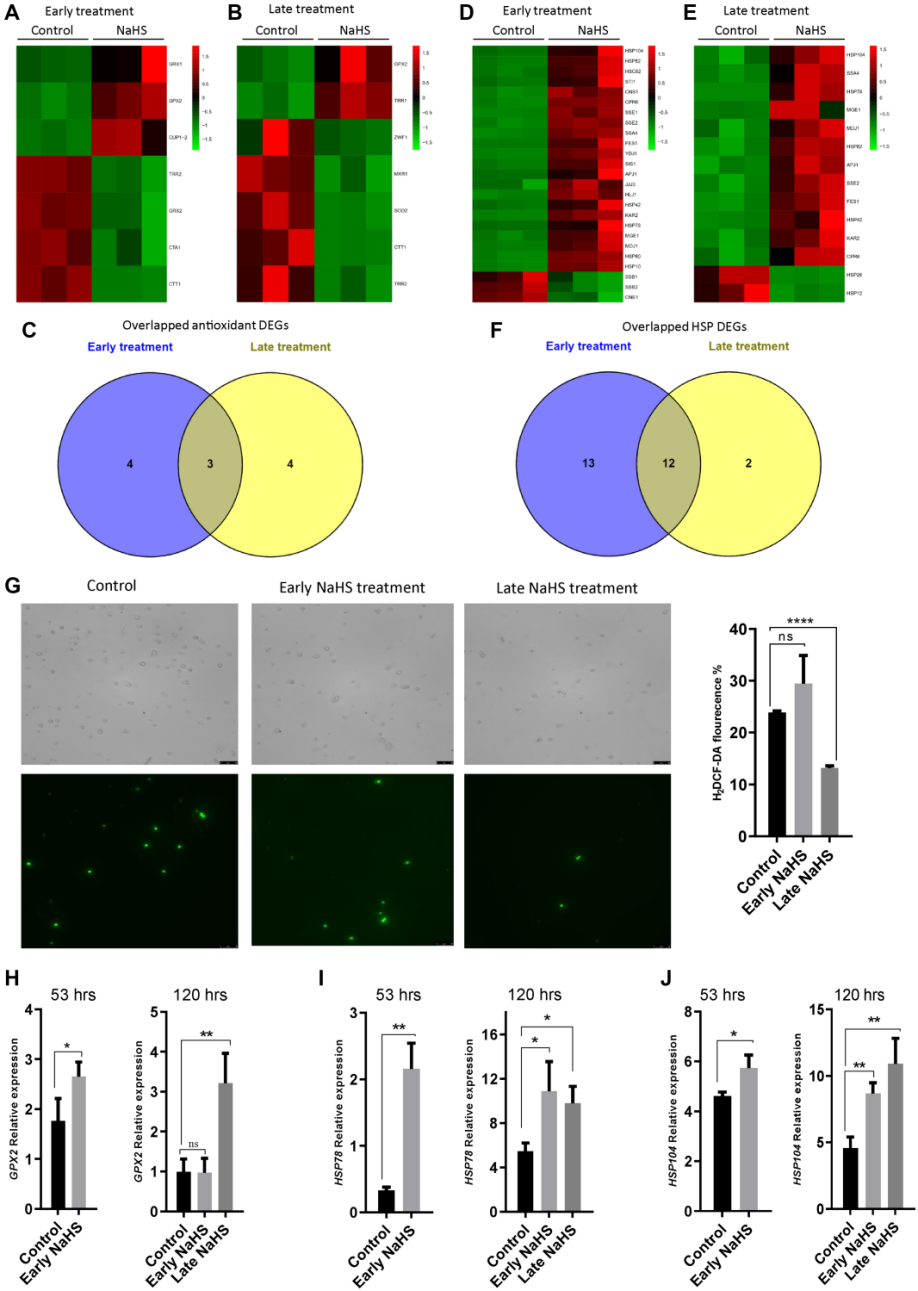
**The late NaHS treatment is more cyto-protective.** (**A** and **B**) Heat maps of antioxidant DEGs in the early and late NaHS treatments. (**C**) Venn diagram representing overlapped antioxidant DEGs. (**D** and **E**) Heat maps of HSP DEGs in the early and late NaHS treatments. (**F**) Venn diagram representing overlapped HSP DEGs. (**G**) The generation of Reactive oxygen species (ROS) in the early and late NaHS treatments at day 5 (120 hours after inoculation) was imaged by fluorescence microscope (left) and quantified by calculating the ratio of positively stained cells (right). (**H**–**J**) qPCR analysis of GPX2 (H) HSP78 (I) and HSP104 (J) at 53 hours or 120 hours after inoculation with or without the indicated NaHS treatment. The expression of these genes were normalized with the expression of actin (ACT).^*^p < 0.05, ^**^p < 0.01, ^***^p < 0.001, ^****^p < 0.0001

Genes involved in oxidative stress response and heat shock response play essential roles in the regulation of life span [27–31]. To explore the effects of H_2_S in these stress responses, we identified and analyzed antioxidant genes and heat shock protein (HSP) genes from the DEGs of early and late NaHS treatments. There are 7 antioxidant genes altered by the early and late NaHS treatments (Figure 5A and 5B, Supplementary Table 6). Among them, expression of 3 genes was similarly altered in both treatments including *GPX2*, *TRR2* and *CTT1* (Figure 5A–5C). As to HSP genes, 25 and 14 DEGs were found in the early and late NaHS treatments respectively with 12 genes were similarly altered in both treatments (Figure 5D–5F, Supplementary Table 7). The similar alteration in the expression of antioxidant genes and HSP genes under NaHS treatments at two different time points is consistent to the observation that both treatments have some common effects on transcriptome (Figure 4). However, when intracellular ROS was monitored at the 5^th^ day (120 hours after inoculation), the late NaHS treatment decreased ROS production but the early NaHS treatment did not (Figure 5G). When the expression of some of similarly altered antioxidant or HSP genes were examined by qPCR, we found that the up-regulation of *HSP78* and *HSP104* by the early NaHS treatment persisted to the 5^th^ day of the growth but the up-regulation of *GPX2* could not (Figure 5H–5J). Instead, the increased GPX2 level induced by the late NaHS treatment stayed up at the 5^th^ day (Figure 5H, right panel). These data suggest that although the early NaHS treatment provided cyto-protective effects, some of them did not last to the later growth stage when the fitness of the cell is essential for increased lifespan.

### The regulation of cell wall integrity contributed to the life span extension by the late NaHS treatment

**Figure 6:**
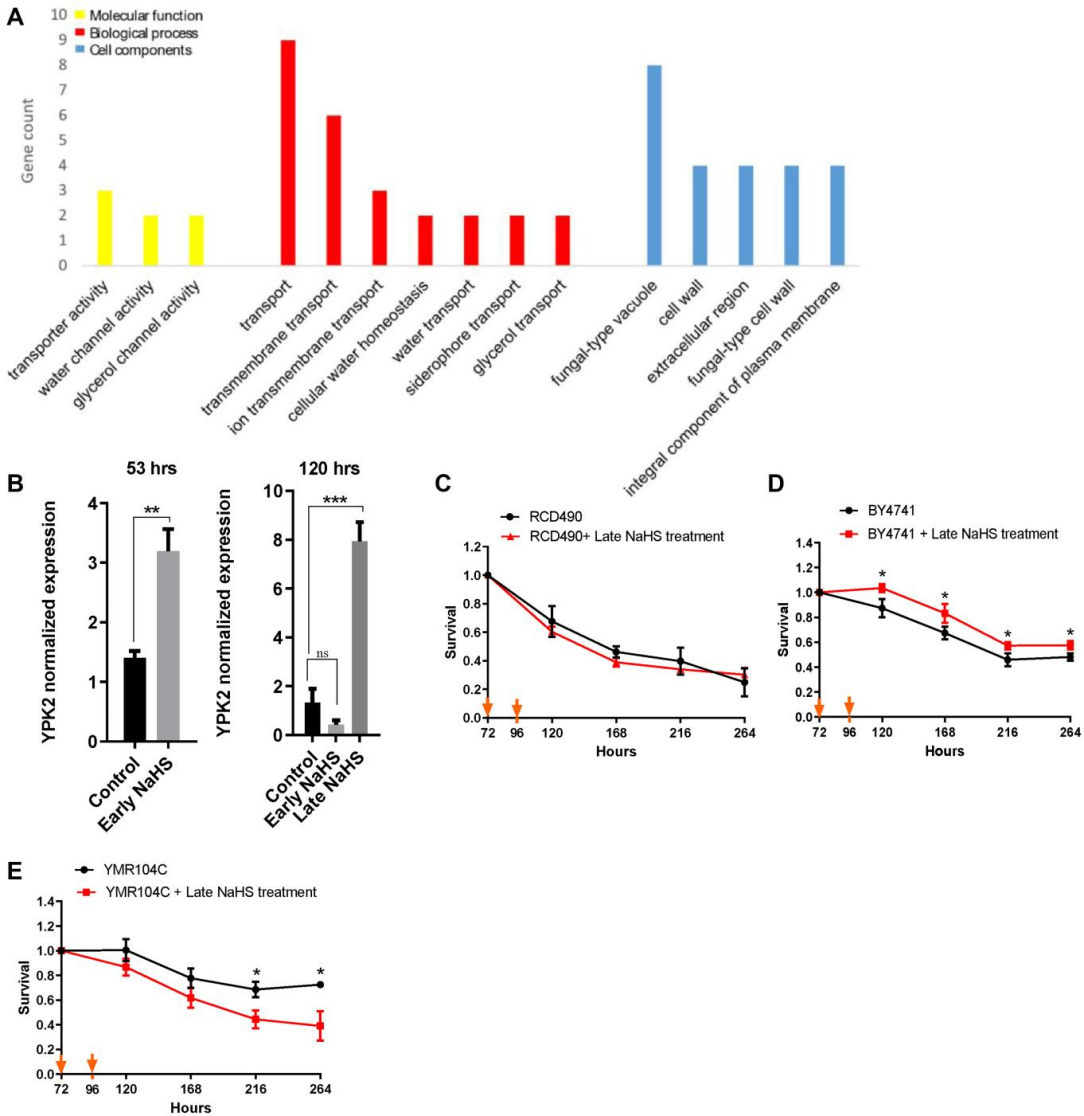
**The late NaHS treatment regulates the cell wall integrity for the extension of CLS.** (**A**) Gene ontology analysis of genes expressed oppositely in response to the early and late NaHS treatments. (**B**) The qPCR analysis of YPK2, an AGC-type protein kinase regulating cell wall integrity. (**C**) CLS of the late NaHS treated RCD490, a ypk2 mutant in BY4742 background. (**D**) CLS of the late NaHS treated BY4741. (**E**) CLS of the late NaHS treated YMR104C, a *ypk2* mutant in BY4741 background. Triplicate cultures were used to achieve mean ± SD of viabilities. ^*^p < 0.05, ^**^p < 0.01, ^***^p < 0.001, ns nonsignificant.

Further analysis of the early and late NaHS treatments identified 36 DEGs that are regulated oppositely (Supplementary Figure 2B, Supplementary Table 4). Interestingly, GO analysis revealed that most of these DEGs regulate cell wall components and transport functions which are also influenced by cell wall [32] (Figure 6A), suggesting there may be a significant difference in the regulation of cell wall integrity between the early and late NaHS treatments. More importantly, these DEGs include *YPK2*, an AGC-type protein kinase and a key regulator of cell wall integrity [33, 34]. The YPK2 is one of the most significant DEGs by the late NaHS treatment while its expression was not altered by the early NaHS treatment (Supplementary Table 6). The qPCR analysis also verified that the expression of *YPK2* was up-regulated about 8-fold by the late NaHS treatment, but not the early NaHS treatment (Figure 6B). Therefore, protein kinase Ypk2 is a potential target of the late NaHS treatment. To test if the increased expression of *YPK2* induced by the late NaHS treatment contributes to the life span extension, the life span of RCD490, a *YPK2* deletion mutant in the BY4742 background, was monitored with or without the late NaHS treatment. We found that the late NaHS treatment did not increase the life span of RCD490 cells (Figure 6C), indicating *YPK2* is required for the life span extension induced by the late NaHS treatment. In addition, the effect of the late NaHS treatment on the CLS of wild type and the *ypk2* mutant from BY4741 background was examined. The late NaHS treatment also increased the life span of wild-type BY4741 cells, although to a less extend than it did to BY4742 (Figure 6D).YMR104C, a *ypk2* mutant at BY4741 background, lived longer than wild-type cells (compare black lines in Figure 6D and 6E). However, the late NaHS treatment did not increase the life span of YMR104C and even decreased the life span (Figure 6E). These data indicate that, although *YPK2* is not a longevity gene by itself, it is required for the life span extension of yeast cells induced by the late NaHS treatment. Therefore, we conclude that in addition to the other pathways altered by the late NaHS treatment, the regulation of cell wall integrity is important for the life span extension.

## DISCUSSION

The beneficial effects of aging interventions are likely to only be achieved with a correct dosage and timing. The relationship between the treatment timing and lifespan extension is studied less due to the complexity of the roles of these interventions during the growth of different organisms. Yeast *Saccharomyces cerevisiae* is a well-established model system for studying aging [35–38]. The growth of yeast cells consists of lag, exponential, stationary and death phases, which are more straightforward for investigating the timing of aging interventions. In this study, we found that one or two NaHS treatments at later time (>96 hrs) are required for CLS extension whereas NaHS treatments earlier than 72 hours of inoculation did not bring any considerable change in CLS (Figure 1). We also found that there is a similar effect on CLS by NaHS treatments at concentrations from 10 μM to 1mM (Figure 2), suggesting that the H_2_S signaling on CLS regulation is not concentration dependent if a critical threshold is crossed. These results indicate that the most crucial factor for the promotion of CLS extension is the timing of NaHS treatment.

In order to achieve greater insight into the underlying mechanism of the differential outcomes from different timing of NaHS treatments, we compared the gene expression profile of the early and late NaHS treated cells to their respective untreated control. We found that both treatments shared some common effects on many metabolic and stress response pathways including biosynthesis of secondary metabolites, carbon metabolism, TCA cycle and metabolism of several amino acids which are related to aging process (Figures 3–5) [39–43].

Despite the obvious similarity of gene expression regulated by the early and late NaHS treatments, there are some DEGs specific to late NaHS treatment were shown by the comparison of the transcriptomes (Figures 4 and 6). Among them, genes related to cell wall integrity seem contribute to CLS extension by the late NaHS treatment because of the importance of cell wall in maintaining yeast lifespan [44]. Indeed, we found that *YPK2*, a key regulator of cell wall integrity, is up-regulated at stationary stage only by the late NaHS treatment and plays essential roles in NaHS induced CLS extension.

ROS, especially mitochondrial ROS are the key regulators of yeast life span [45, 46]. Increased mitochondrial membrane potential and superoxide production are suggested as an adaptive signal during growth that promotes CLS extension [47]. We show that the late NaHS treatment decreased intracellular ROS significantly but the early NaHS treatment had no effect. In addition, apart from some antioxidant genes specifically regulated in each case, both treatments shared common effects on many antioxidant genes. However, we found that some of those effects caused by the early treatments, such as the altered expression of *GPX2*, did not last longer and reverted back after sometime (Figure 5), which may contribute to their incompetence to reduce ROS and promote the CLS extension. Furthermore, the observation of changed expression of antioxidant and HSP genes suggested that there are some intracellular alterations in redox status. It also provided potential candidates which may be responsible for some of the effects induced by NaHS treatment.

Together, our data demonstrate that the timing of H_2_S treatment is vital for promoting CLS extension in yeast. The systematic comparison of the gene expression dynamics of the early and late NaHS treatments indicates that the persistence and specificity of H_2_S induced changes in gene expression are crucial for the longevity benefits. Indeed, these data provided new insights in to the aging intervention by using H_2_S and suggesting that the timing of H_2_S-type of interventions in multicellular eukaryotes is likely to be critical for maximizing health benefits and will require further research efforts.

## MATERIALS AND METHODS

The yeast strains and their genetic backgrounds are enlisted in the Supplementary Table 8. Yeast cells were grown in synthetic dextrose complete (SDC) having composition as shown in Supplementary Table 9. The initial pH of SDC was adjusted to 6.0 [48]. For NaHS treatments, the desired amount of freshly prepared aqueous solution of NaHS (50 mM, Sigma-Aldrich) was added to the cell cultures at the indicated times.

For CLS analysis, yeast cells were grown overnight at 30ºC in SDC medium and then inoculated into 10 ml medium in 50 ml capacity flasks to achieve an initial A_600nm_ of 0.005. These cultures were further kept in a shaking incubator (200 rpm for proper aeration) at 30ºC for indicated time and cell viability was measured by spreading the diluted cultures on YPD agar plates (1% yeast extract, 2% peptone, 2% glucose). The CLS was estimated by counting the number of colonies obtained from the incubation at 30ºC and expressed as fraction of day 3 (72 hours) value.

The 2′, 7′-dichlorodihydrofluorescein diacetate (H_2_DCF-DA) staining was used for the analysis of ROS [49]. For the H_2_DCF-DA staining, cells (OD600nm of 1.0) were collected and incubated with 10 μM H_2_DCF-DA at 30 °C for 60–90 minutes. After the staining, cells were thoroughly washed twice with PBS buffer and then re-suspended in 1 ml PBS. The H_2_DCF-DA-stained cells were observed by fluorescence microscopy (excitation/emission: 488 nm/530 nm).

For transcriptome and real-time quantitative PCR (RT-qPCR) analysis, the early treatment comprised 2 doses of 100 μM NaHS at the time as indicated in the figures. The total RNA was extracted using TRIzol Reagent according the manufacturer’s instructions (Invitrogen) and genomic DNA was removed using DNase I (TaKara, China). RNA-seq transcriptome library was prepared using TruSeq RNA sample preparation Kit from Illumina (San Diego, CA, USA). Libraries were size selected for cDNA target fragments of 200–300 bp on 2% Low Range Ultra Agarose followed by PCR amplified using Phusion DNA polymerase (NEB) for 15 PCR cycles. After quantified by TBS380, paired-end RNA-seq sequencing library was sequenced with the Illumina HiSeq xten/NovaSeq 6000 sequencer. The raw paired end reads were trimmed and quality controlled by SeqPrep and Sickle software with default parameters. Then clean reads were separately aligned to reference genome with orientation mode using TopHat software. R statistical package software EdgeR was used for differential expression analysis, heat maps, volcano graphs and bubble charts. For gene ontology term and KEGG (Kyoto encyclopedia of genes and genomes) pathways enrichment analysis, David bioinformatics database (version 6.8) was used.

For RT-qPCR analysis, reverse transcription reactions were performed by using a Prime Script RT reagent kit (Takara, China). The primers are listed in Supplementary Table 10. Quantitative PCR was performed by using SYBR Premix Ex Taq II (TaKaRa Bio, China) and Bio-Rad CFX manager RT-qPCR system. Data were collected and analyzed by Bio-Rad CFX manager software. All RT-qPCR data from at least three independent experiments are presented as averages ± SD. Statistical analysis and comparisons were performed by using two-tailed, unpaired Student *t*-tests.

### Data availability statement

The data that support the findings of this study are available from the corresponding author upon reasonable request.

## Supplementary Material

Supplementary Figures

Supplementary Table 1

Supplementary Table 2

Supplementary Table 3

Supplementary Table 4

Supplementary Table 5

Supplementary Table 6

Supplementary Table 7

Supplementary Tables 8-10
