# Supplementary material for: Hydrogen sulfide treatment at the late growth stage of Saccharomyces cerevisiae extends chronological lifespan
Source: Aging (Albany NY). 2021 Mar 19;13(7):9859–73. doi: 10.18632/aging.202738 (PMC8064171; doi:10.18632/aging.202738)
Supplement: Supplementary Figures [file aging-13-202738-s001.pdf]

SUPPLEMENTARY FIGURES

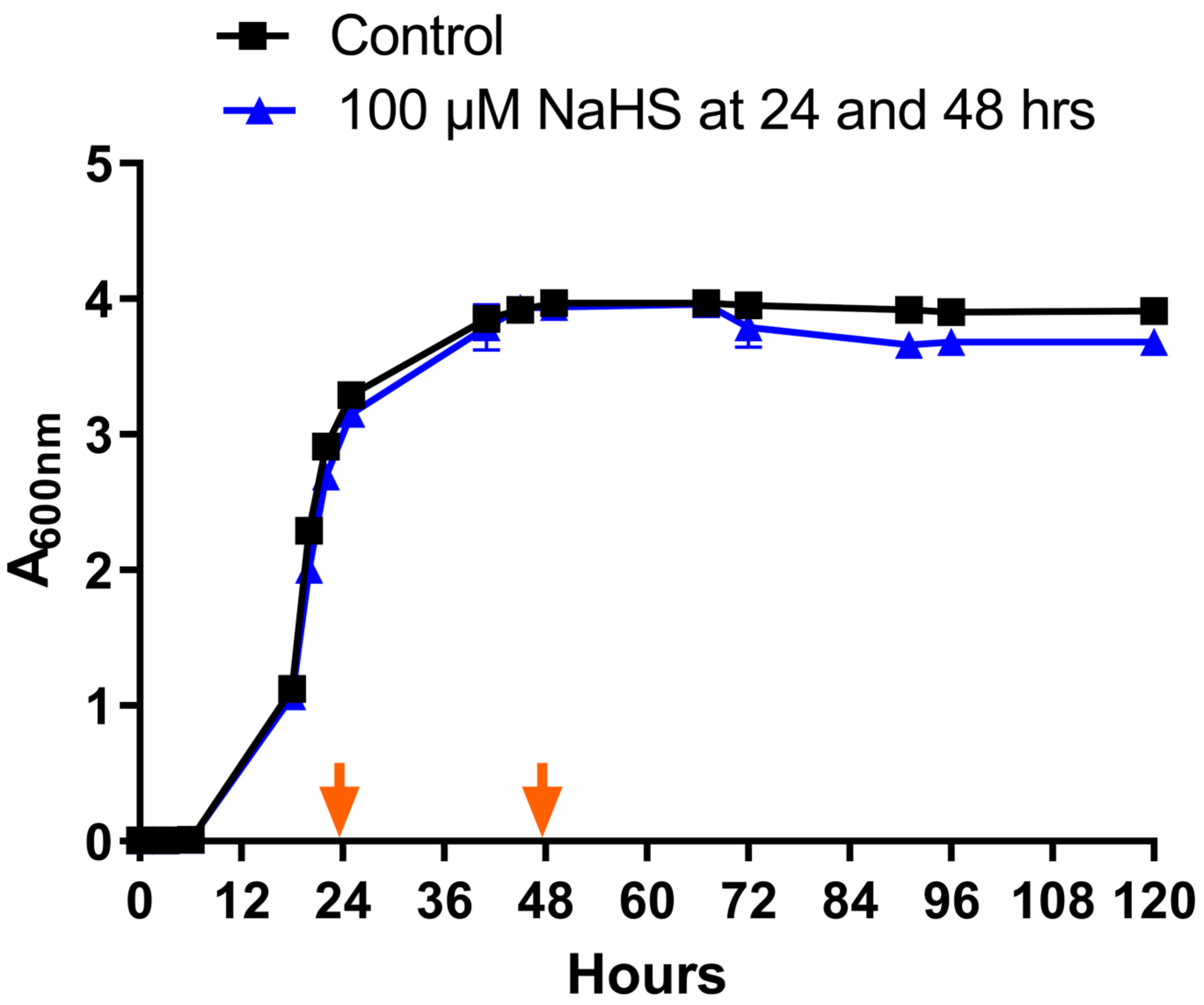

Supplementary Figure 1. The effect of NaHS treatment on yeast cell growth. Yeast cells (BY4742 background) were treated with or without 100 µM of NaHS at 24 and 48 hours after inoculation as indicated as arrows. Biomass growth was measured at OD600.

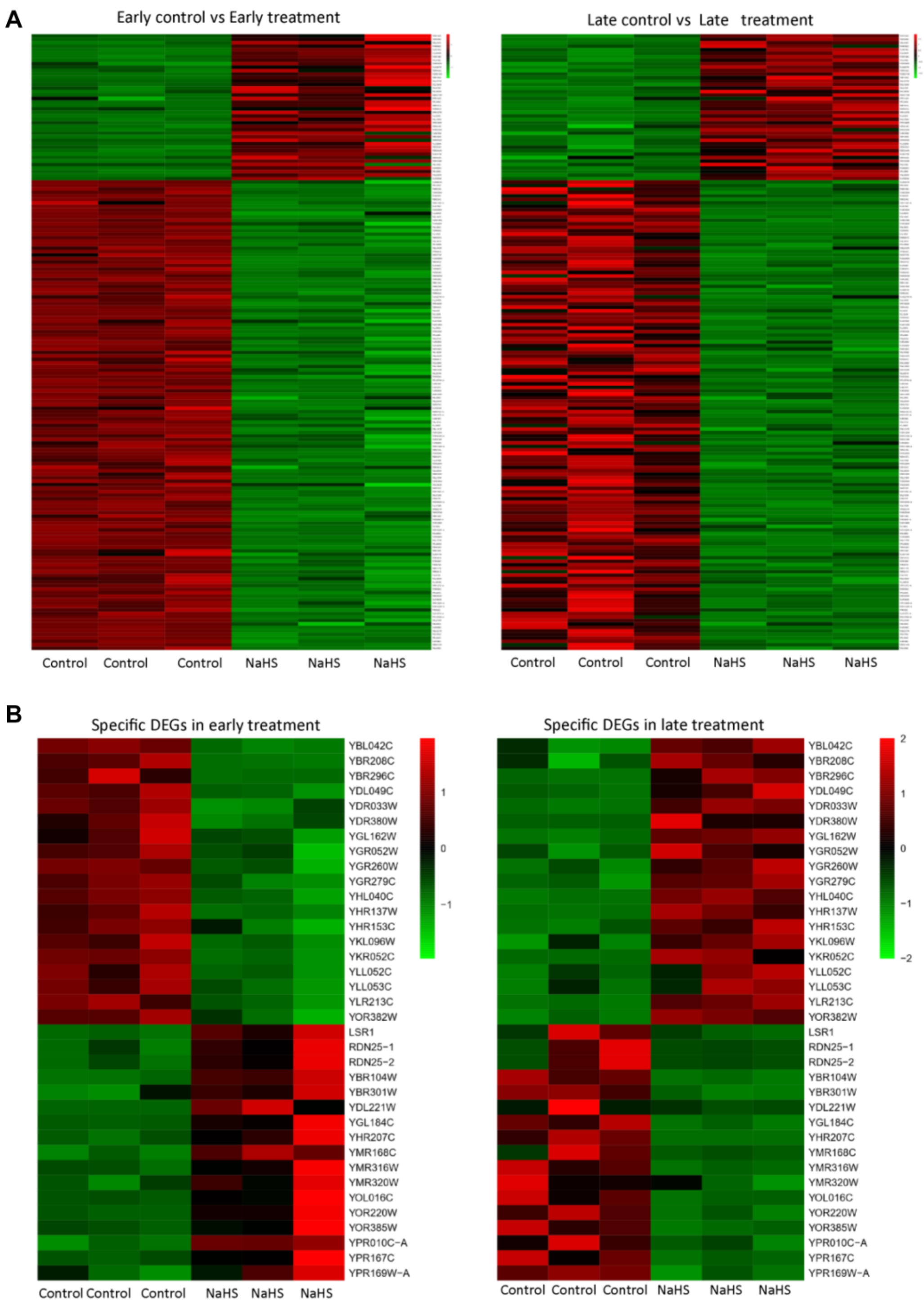

Supplementary Figure 2. DEGs common in both the early and late NaHS treatments. (**A**) Heat maps of similar behaving DEGs. (**B**) Heat maps of opposite behaving DEGs.
