# Supplementary material for: Hydrogen sulfide treatment at the late growth stage of Saccharomyces cerevisiae extends chronological lifespan
Source: Aging (Albany NY). 2021 Mar 19;13(7):9859–73. doi: 10.18632/aging.202738 (PMC8064171; doi:10.18632/aging.202738)
Supplement: Supplementary Table 1 [file aging-13-202738-s002.doc]

**Supplementary Table 1. DEGs induced by the early NaHS treatment.**

| Gene_id | FC(NaHS/Control) | Log2FC(NaHS/Control) | Pvalue | Padjust | Significant | Regulate | Control1_Fpkm | Control2_Fpkm | Control3_Fpkm | NaHS1_Fpkm | NaHS2_Fpkm | NaHS3_Fpkm |
| --- | --- | --- | --- | --- | --- | --- | --- | --- | --- | --- | --- | --- |
| YLR217W | 7.421 | 2.891707 | 2.53E-76 | 3.22E-73 | yes | up | 93.26 | 92.02 | 93.97 | 693.07 | 704.91 | 617.64 |
| YLR216C | 6.262 | 2.646596 | 2.06E-59 | 1.86E-56 | yes | up | 122.42 | 92.72 | 98.45 | 633.59 | 649.3 | 696.89 |
| YKR071C | 4.631 | 2.211279 | 6.09E-57 | 4.83E-54 | yes | up | 309.86 | 245.65 | 319.35 | 1127.03 | 1001.56 | 1273.05 |
| YPL106C | 4.579 | 2.195139 | 1.51E-51 | 7.97E-49 | yes | up | 204.93 | 205.68 | 221.51 | 975.64 | 866.8 | 1047.23 |
| YJL034W | 5.289 | 2.402959 | 1.84E-49 | 8.99E-47 | yes | up | 139.87 | 122.93 | 127.69 | 605.9 | 696.74 | 777.67 |
| YOR204W | 4.147 | 2.052141 | 6.29E-46 | 2E-43 | yes | up | 248.37 | 234.26 | 245.53 | 962.53 | 933.26 | 1110.67 |
| RUF5-2 | 15.023 | 3.909083 | 6.29E-45 | 1.9E-42 | yes | up | 1463.24 | 1289.42 | 1307.1 | 26947.99 | 30955.38 | 13111.4 |
| YPR154W | 3.14 | 1.650921 | 2.94E-43 | 7.78E-41 | yes | up | 887.71 | 877.86 | 873.53 | 2646.71 | 2629.39 | 2825.22 |
| YBR169C | 3.067 | 1.616731 | 1.62E-42 | 3.95E-40 | yes | up | 108.2 | 94.73 | 101.18 | 301.11 | 317.09 | 302.35 |
| YHR054C | 11.256 | 3.49261 | 4.29E-39 | 9.08E-37 | yes | up | 773.43 | 743.31 | 723.93 | 11458.8 | 12778.67 | 5764.22 |
| YHR052W-A | 13.981 | 3.805353 | 6.08E-39 | 1.25E-36 | yes | up | 12.33 | 5.52 | 10.51 | 177.38 | 178.3 | 112.38 |
| YPL239W | 4.587 | 2.197683 | 1.38E-36 | 2.36E-34 | yes | up | 23.51 | 20.43 | 24.81 | 97.11 | 113.55 | 104.36 |
| YNL064C | 4.111 | 2.039646 | 9.28E-36 | 1.51E-33 | yes | up | 78.21 | 71.39 | 72.15 | 272.84 | 293.07 | 345.68 |
| YPL026C | 4.556 | 2.187816 | 1.35E-34 | 2.09E-32 | yes | up | 12.55 | 12.66 | 13 | 69.22 | 50.8 | 56.2 |
| YPL151C | 3.101 | 1.63289 | 6.88E-34 | 9.92E-32 | yes | up | 44.79 | 46.03 | 49.73 | 138.68 | 158.2 | 134.68 |
| RUF5-1 | 12.664 | 3.662623 | 1.1E-32 | 1.52E-30 | yes | up | 1439.91 | 1673.22 | 1697.05 | 29106.23 | 32335.88 | 12053.25 |
| YHR055C | 15.183 | 3.924412 | 7.93E-31 | 9.86E-29 | yes | up | 2546.33 | 4771.31 | 2030.68 | 62502.73 | 64744.93 | 34272.64 |
| YGR211W | 6.118 | 2.613172 | 2.82E-29 | 3.03E-27 | yes | up | 130.84 | 112.49 | 111.03 | 577.48 | 714.57 | 984.99 |
| YBR101C | 5.765 | 2.527205 | 6.09E-28 | 5.77E-26 | yes | up | 336.11 | 313.9 | 313.61 | 1619.2 | 1638.04 | 2603.94 |
| YOR011W | 2.507 | 1.326094 | 1.86E-26 | 1.59E-24 | yes | up | 43.64 | 45.11 | 44.26 | 117.68 | 123.94 | 136.3 |
| YMR161W | 2.776 | 1.472954 | 7.26E-26 | 5.98E-24 | yes | up | 79.59 | 87.13 | 86.02 | 224.31 | 252.14 | 213.67 |
| YGL058W | 3.773 | 1.915681 | 1.94E-25 | 1.48E-23 | yes | up | 64.97 | 64.76 | 69.49 | 221.94 | 235.9 | 288 |
| YML081W | 2.707 | 1.436888 | 3.56E-25 | 2.6E-23 | yes | up | 20.74 | 19.21 | 21.57 | 51.24 | 55.11 | 58.48 |
| YGR142W | 10.406 | 3.379363 | 4.64E-25 | 3.31E-23 | yes | up | 38.79 | 35.08 | 38.48 | 303.14 | 347.23 | 761.46 |
| YMR295C | 2.737 | 1.452566 | 5.99E-25 | 4.22E-23 | yes | up | 964.81 | 991.85 | 992.55 | 2581.33 | 2445.78 | 2866.48 |
| YGR210C | 2.512 | 1.329019 | 1.83E-24 | 1.26E-22 | yes | up | 128.49 | 123.48 | 142.8 | 308.69 | 345.14 | 322.73 |
| YLR259C | 2.428 | 1.279906 | 4.66E-24 | 3.08E-22 | yes | up | 1077.37 | 987.27 | 990.76 | 2345.13 | 2389.45 | 2569.09 |
| YKL156W | 3.04 | 1.60402 | 1.38E-23 | 8.86E-22 | yes | up | 855.15 | 782.58 | 807.11 | 2227.15 | 2125.94 | 2477 |
| YHR053C | 11.301 | 3.498423 | 4.17E-23 | 2.59E-21 | yes | up | 5911.88 | 1996.99 | 4102.87 | 52452.45 | 68558.7 | 26181.01 |
| YLL024C | 3.2 | 1.678053 | 9.09E-23 | 5.55E-21 | yes | up | 175.85 | 174.17 | 172.89 | 470.74 | 561.09 | 640.19 |
| YPL240C | 4.756 | 2.249824 | 1.13E-22 | 6.7E-21 | yes | up | 261.45 | 232.97 | 235.54 | 1000.03 | 1013.29 | 1589.12 |
| YNL074C | 2.565 | 1.358989 | 2.8E-22 | 1.6E-20 | yes | up | 395.45 | 337.86 | 354.09 | 884.45 | 885.35 | 980.79 |
| YOR020C | 2.701 | 1.433577 | 4.91E-22 | 2.74E-20 | yes | up | 1826.47 | 1726.75 | 1665.06 | 4235.47 | 4202.26 | 4887.03 |
| YNL006W | 3.233 | 1.692703 | 5.05E-22 | 2.77E-20 | yes | up | 190.96 | 176.82 | 167.57 | 489.56 | 576.06 | 658.69 |
| YOR208W | 2.643 | 1.402337 | 8.07E-22 | 4.38E-20 | yes | up | 17.61 | 14.79 | 17.78 | 46.89 | 42.39 | 42.17 |
| YER150W | 3.055 | 1.611323 | 1.75E-21 | 9.11E-20 | yes | up | 10664.53 | 9832.02 | 9352.38 | 26959.86 | 27627.15 | 34343.7 |
| YOR338W | 5.112 | 2.353985 | 2.25E-21 | 1.15E-19 | yes | up | 100.49 | 86.94 | 93.57 | 433.5 | 388.71 | 685.82 |
| YKL009W | 4.388 | 2.133566 | 4E-21 | 2.01E-19 | yes | up | 15.88 | 13.47 | 15.15 | 62.42 | 56.05 | 81.53 |
| YNL007C | 4.313 | 2.108784 | 4.03E-21 | 2.02E-19 | yes | up | 494.47 | 415.43 | 417.35 | 1638.47 | 1644.69 | 2531.36 |
| YJR046W | 2.956 | 1.563633 | 4.52E-21 | 2.21E-19 | yes | up | 111.47 | 104.85 | 91.59 | 311.99 | 344.32 | 254.53 |
| YFL016C | 3.779 | 1.917906 | 7.25E-21 | 3.44E-19 | yes | up | 162.28 | 153.03 | 158.14 | 510.59 | 542.95 | 761.31 |
| YPL169C | 2.258 | 1.174916 | 5.38E-20 | 2.35E-18 | yes | up | 40.34 | 37.75 | 42.53 | 84.95 | 93.89 | 89.3 |
| YPR145W | 2.454 | 1.294974 | 6.74E-20 | 2.91E-18 | yes | up | 167.17 | 173.58 | 198.3 | 448.22 | 406.2 | 451.67 |
| YNL124W | 3.123 | 1.64276 | 1.32E-19 | 5.52E-18 | yes | up | 52.02 | 48.23 | 50.52 | 145.9 | 138.5 | 186.43 |
| YMR256C | 3.148 | 1.654255 | 2.74E-19 | 1.1E-17 | yes | up | 4864.24 | 4850.82 | 4930.05 | 12521.33 | 11981.73 | 14539.13 |
| YPL213W | 2.651 | 1.406802 | 2.82E-19 | 1.13E-17 | yes | up | 27.67 | 27.27 | 25.99 | 66.47 | 72.9 | 72.16 |
| YBR118W | 2.027 | 1.019126 | 1.87E-18 | 7.14E-17 | yes | up | 12870.19 | 12896.18 | 14188.46 | 26935.24 | 25777.18 | 26692.12 |
| YIL130W | 2.844 | 1.508013 | 3.2E-18 | 1.2E-16 | yes | up | 5.97 | 5.15 | 6.87 | 17.38 | 16.05 | 17.69 |
| YBL033C | 2.183 | 1.126634 | 9.27E-18 | 3.38E-16 | yes | up | 1158.78 | 1111.35 | 1097.53 | 2458.65 | 2215.46 | 2539.66 |
| YML130C | 4.531 | 2.17973 | 9.4E-18 | 3.41E-16 | yes | up | 12.97 | 11.35 | 14.35 | 48.73 | 52.25 | 83.05 |
| YOL067C | 2.336 | 1.224034 | 2.06E-17 | 7.29E-16 | yes | up | 79.08 | 67.91 | 79.44 | 169.34 | 181.45 | 164.43 |
| YMR294W-A | 2.883 | 1.527623 | 2.43E-17 | 8.5E-16 | yes | up | 532.79 | 540.83 | 492.88 | 1366.81 | 1296.99 | 1690.73 |
| YCR008W | 2.575 | 1.364605 | 1.06E-16 | 3.52E-15 | yes | up | 68.22 | 55.83 | 60.23 | 145.02 | 146.5 | 175.17 |
| YER103W | 2.282 | 1.190199 | 1.09E-16 | 3.6E-15 | yes | up | 108.92 | 109.97 | 120 | 385.23 | 411.79 | 443.8 |
| YOR007C | 2.625 | 1.392113 | 1.58E-16 | 5.11E-15 | yes | up | 279.19 | 270.14 | 270.86 | 619.49 | 689.5 | 823.43 |
| YKR062W | 2.163 | 1.113255 | 1.75E-16 | 5.63E-15 | yes | up | 72.76 | 83.52 | 73.86 | 164.02 | 165.54 | 159.96 |
| YDL221W | 20.763 | 4.375947 | 2.12E-16 | 6.79E-15 | yes | up | 0 | 0 | 0 | 13.16 | 19.67 | 6.23 |
| YNL157W | 2.99 | 1.580236 | 4.31E-16 | 1.34E-14 | yes | up | 1035.74 | 1087.53 | 1014.39 | 2840.22 | 2664.12 | 3768.5 |
| YKL031W | 2.113 | 1.079524 | 5.24E-16 | 1.62E-14 | yes | up | 50.75 | 46.53 | 49.92 | 98.51 | 99.06 | 108.46 |
| YBR150C | 3.733 | 1.900358 | 5.45E-16 | 1.67E-14 | yes | up | 9.64 | 8.72 | 8.51 | 29.23 | 28.76 | 45.4 |
| YBR155W | 2.962 | 1.566774 | 9.16E-16 | 2.72E-14 | yes | up | 7.04 | 6.08 | 6.56 | 21.16 | 17.55 | 19.82 |
| YER147C-A | 2.703 | 1.434826 | 2.39E-15 | 6.9E-14 | yes | up | 96.93 | 85.05 | 83.64 | 246.12 | 200.44 | 251.23 |
| YFR040W | 2.39 | 1.257294 | 3.19E-15 | 9.08E-14 | yes | up | 16.38 | 14.42 | 14.84 | 34.09 | 33.94 | 40.12 |
| YDR186C | 2.075 | 1.052937 | 3.77E-15 | 1.06E-13 | yes | up | 44.48 | 36.71 | 41.56 | 83.66 | 83.09 | 84.14 |
| YGR250C | 2.756 | 1.462475 | 4.77E-15 | 1.34E-13 | yes | up | 57.8 | 57.6 | 62.66 | 146.32 | 146.83 | 197.51 |
| YJL217W | 2.061 | 1.043014 | 6.97E-15 | 1.92E-13 | yes | up | 713.24 | 680.7 | 724.14 | 1574.33 | 1448.08 | 1236.47 |
| YPL089C | 2.252 | 1.171281 | 9.92E-15 | 2.71E-13 | yes | up | 31.69 | 27.91 | 27.79 | 70.69 | 68.46 | 55.96 |
| YER148W | 2.615 | 1.387016 | 1.25E-14 | 3.37E-13 | yes | up | 100.86 | 85.28 | 92.14 | 217.21 | 222.73 | 279.53 |
| YPR190C | 2.917 | 1.544343 | 1.68E-14 | 4.44E-13 | yes | up | 17.4 | 13.48 | 14.9 | 54.81 | 44.05 | 36.24 |
| YKL109W | 3.009 | 1.589093 | 2.79E-14 | 7.24E-13 | yes | up | 114.85 | 90.95 | 117.1 | 382.1 | 238.22 | 390.66 |
| YBR065C | 3.889 | 1.959513 | 3.56E-14 | 9.07E-13 | yes | up | 27.81 | 21.44 | 23.39 | 79.2 | 81.38 | 133.82 |
| YBR104W | 3.267 | 1.707773 | 3.88E-14 | 9.81E-13 | yes | up | 44.27 | 44.19 | 49.77 | 132.55 | 128.21 | 197.68 |
| YDR217C | 4.063 | 2.022684 | 4.56E-14 | 1.14E-12 | yes | up | 0.67 | 0.59 | 1.01 | 2.8 | 3.39 | 3.56 |
| YMR272C | 2.21 | 1.143767 | 9.55E-14 | 2.28E-12 | yes | up | 846.85 | 767.25 | 872.42 | 1772.55 | 1631.45 | 2005.83 |
| YDR151C | 2.281 | 1.189802 | 1.54E-13 | 3.54E-12 | yes | up | 50.14 | 50.37 | 50.22 | 101.55 | 110.95 | 126.54 |
| YER104W | 2.108 | 1.075644 | 2.14E-13 | 4.89E-12 | yes | up | 90 | 89.36 | 81.91 | 168.36 | 180.08 | 189.42 |
| YNR075W | 2.785 | 1.477442 | 3.12E-13 | 7.05E-12 | yes | up | 16.18 | 13.26 | 16.4 | 39.85 | 37.9 | 50.11 |
| YMR043W | 2.798 | 1.484529 | 4.79E-13 | 1.07E-11 | yes | up | 181.08 | 139.49 | 144.46 | 387.2 | 388.83 | 525.35 |
| YPL055C | 2.342 | 1.227663 | 5.9E-13 | 1.3E-11 | yes | up | 619.62 | 560.02 | 570.63 | 1253.52 | 1220.56 | 1574.33 |
| YOR344C | 3.591 | 1.844321 | 7.66E-13 | 1.66E-11 | yes | up | 3.77 | 4.36 | 4.87 | 14.59 | 14.84 | 19.05 |
| YBR105C | 2.101 | 1.070762 | 1.02E-12 | 2.19E-11 | yes | up | 844.39 | 869.48 | 760.98 | 1917.56 | 1744.36 | 1471.45 |
| YDR219C | 2.182 | 1.125887 | 1.12E-12 | 2.4E-11 | yes | up | 15.87 | 15.77 | 14.08 | 30.61 | 34.03 | 34.04 |
| YKR086W | 2.151 | 1.104951 | 1.13E-12 | 2.4E-11 | yes | up | 7.18 | 6.25 | 7.38 | 14.06 | 14.7 | 15.49 |
| YOR213C | 2.163 | 1.112827 | 1.23E-12 | 2.61E-11 | yes | up | 81.2 | 92.72 | 72.83 | 175.33 | 182.86 | 167.32 |
| YML076C | 2.644 | 1.402909 | 1.23E-12 | 2.61E-11 | yes | up | 25.5 | 22.82 | 27.66 | 57.56 | 62.49 | 81.31 |
| YDR171W | 3.503 | 1.808683 | 1.26E-12 | 2.67E-11 | yes | up | 1121.64 | 1020.93 | 951.02 | 2925 | 3091.28 | 5192.61 |
| YNL281W | 3.091 | 1.6283 | 1.4E-12 | 2.92E-11 | yes | up | 166.17 | 151.93 | 155.48 | 416.13 | 407.61 | 636.35 |
| YPR153W | 2.412 | 1.269999 | 1.55E-12 | 3.2E-11 | yes | up | 60.74 | 65.9 | 61.48 | 162.23 | 156.48 | 124.64 |
| YDL048C | 2.866 | 1.518988 | 1.72E-12 | 3.52E-11 | yes | up | 74.45 | 69.71 | 95.34 | 274.88 | 249.53 | 173.03 |
| YOL047C | 2.024 | 1.017275 | 2.07E-12 | 4.22E-11 | yes | up | 16.41 | 21.35 | 17.44 | 52.21 | 47.49 | 47.89 |
| YMR186W | 2.751 | 1.460195 | 2.56E-12 | 5.1E-11 | yes | up | 179.78 | 162.02 | 178.82 | 405.01 | 427.96 | 608.07 |
| YML118W | 2.905 | 1.538668 | 2.6E-12 | 5.16E-11 | yes | up | 50.87 | 43.67 | 40.64 | 113.59 | 116.17 | 167.46 |
| YGR138C | 2.147 | 1.102007 | 2.73E-12 | 5.4E-11 | yes | up | 53.93 | 48.84 | 58.45 | 105.11 | 110.74 | 125.84 |
| YOR027W | 3.471 | 1.795216 | 3.33E-12 | 6.48E-11 | yes | up | 309.94 | 266.61 | 265.61 | 799.5 | 819.32 | 1414.61 |
| YMR115W | 2.191 | 1.131313 | 3.53E-12 | 6.85E-11 | yes | up | 32.36 | 35.94 | 34.52 | 69 | 70.41 | 83.23 |
| YBR025C | 2.285 | 1.192459 | 3.56E-12 | 6.89E-11 | yes | up | 71.2 | 66.34 | 83.01 | 155.76 | 155.94 | 186.77 |
| YBR247C | 2.283 | 1.190904 | 4.26E-12 | 8.21E-11 | yes | up | 15.37 | 11.56 | 14.8 | 30.94 | 31.48 | 31.98 |
| YDL070W | 2.822 | 1.496793 | 4.31E-12 | 8.3E-11 | yes | up | 142.41 | 124.31 | 139.44 | 344.75 | 318.62 | 493.23 |
| YER052C | 2.389 | 1.256459 | 7.49E-12 | 1.39E-10 | yes | up | 68.55 | 66.37 | 81.47 | 147.42 | 169.49 | 197.15 |
| YDR433W | 2.335 | 1.223144 | 7.9E-12 | 1.46E-10 | yes | up | 466 | 377.26 | 413.86 | 971.06 | 805.21 | 1070.18 |
| YKL032C | 2.313 | 1.209609 | 1.19E-11 | 2.16E-10 | yes | up | 184.72 | 160.37 | 165.28 | 360.33 | 351.25 | 459.93 |
| YDR524C-B | 2.091 | 1.064282 | 1.19E-11 | 2.16E-10 | yes | up | 7413.76 | 7436.64 | 8108.47 | 15337.37 | 14003.54 | 12490.59 |
| YDR258C | 4.014 | 2.004966 | 1.88E-11 | 3.34E-10 | yes | up | 89.29 | 80.4 | 79.77 | 273.58 | 255.19 | 550.52 |
| YFR033C | 2.224 | 1.153304 | 2.24E-11 | 3.96E-10 | yes | up | 1640.99 | 1392.44 | 1600.96 | 3100.5 | 3025.59 | 3852.01 |
| YNR010W | 2.472 | 1.305956 | 2.73E-11 | 4.77E-10 | yes | up | 91.93 | 62.14 | 70.98 | 172.03 | 175.09 | 196.19 |
| YOR031W | 2.768 | 1.468646 | 2.86E-11 | 4.98E-10 | yes | up | 3951.4 | 3425.62 | 3377.35 | 8019.28 | 7369.05 | 11160.78 |
| YLL026W | 3.518 | 1.814899 | 3.6E-11 | 6.2E-10 | yes | up | 405.33 | 352.61 | 361.03 | 1080.92 | 1049.53 | 2002.92 |
| YBR114W | 2.117 | 1.081859 | 3.84E-11 | 6.6E-10 | yes | up | 53.79 | 55.73 | 53.49 | 106.77 | 103.85 | 130.65 |
| YEL026W | 2.901 | 1.536499 | 4.2E-11 | 7.19E-10 | yes | up | 85.12 | 86.68 | 107.08 | 265.13 | 212.33 | 321.59 |
| YKR008W | 2.712 | 1.439522 | 4.56E-11 | 7.78E-10 | yes | up | 5.5 | 5.29 | 6.81 | 14.54 | 14.45 | 19.19 |
| YPR045C | 2.264 | 1.178661 | 5.19E-11 | 8.79E-10 | yes | up | 19.41 | 18.39 | 23.32 | 41.6 | 44.9 | 50.73 |
| YIL117C | 2.095 | 1.066854 | 5.69E-11 | 9.58E-10 | yes | up | 75.62 | 71.01 | 73.27 | 162.63 | 169.88 | 123.07 |
| YOR327C | 2.403 | 1.264934 | 8.03E-11 | 1.33E-09 | yes | up | 772.47 | 792.1 | 746.86 | 1581.94 | 1602.64 | 2149.58 |
| YOR298C-A | 2.616 | 1.387188 | 8.52E-11 | 1.4E-09 | yes | up | 2452.86 | 2369.13 | 2376.81 | 5395.25 | 5233.44 | 7910.15 |
| YDR149C | 6.643 | 2.731774 | 1.01E-10 | 1.65E-09 | yes | up | 1.57 | 2.02 | 4.02 | 15.79 | 15.51 | 38.59 |
| YAL005C | 2.766 | 1.467738 | 1.29E-10 | 2.08E-09 | yes | up | 1392.87 | 1385.94 | 1335.99 | 3123.74 | 3315.98 | 5091.48 |
| YLR189C | 2.89 | 1.531188 | 1.3E-10 | 2.1E-09 | yes | up | 9.8 | 7.85 | 9.61 | 21.83 | 23.44 | 35.12 |
| YMR251W-A | 3.081 | 1.623427 | 1.32E-10 | 2.12E-09 | yes | up | 7580.95 | 6328.66 | 6448.26 | 15483.97 | 14081.02 | 23980.83 |
| YNL077W | 3.495 | 1.805163 | 1.41E-10 | 2.26E-09 | yes | up | 344.27 | 320.43 | 323.62 | 881.31 | 961.91 | 1791.87 |
| YPR157W | 2.509 | 1.327112 | 1.72E-10 | 2.73E-09 | yes | up | 20.75 | 19.99 | 18.41 | 62.8 | 48.19 | 38.73 |
| YKR092C | 2.075 | 1.05297 | 1.75E-10 | 2.77E-09 | yes | up | 14.84 | 15.27 | 13.31 | 31.66 | 31.15 | 26.43 |
| YHR179W | 2.625 | 1.392524 | 2.02E-10 | 3.16E-09 | yes | up | 219.59 | 228.77 | 230.9 | 794.33 | 572.07 | 441.92 |
| YML090W | 3.456 | 1.78916 | 2.09E-10 | 3.26E-09 | yes | up | 41.5 | 44.71 | 62.62 | 164.88 | 134.4 | 227.85 |
| YNL164C | 2.3 | 1.201656 | 2.26E-10 | 3.51E-09 | yes | up | 10.94 | 12.08 | 11.76 | 30.66 | 23.73 | 25.33 |
| YGR136W | 2.008 | 1.0059 | 2.3E-10 | 3.57E-09 | yes | up | 451.3 | 423.28 | 431.98 | 817.26 | 777.04 | 973.99 |
| YDL113C | 2.518 | 1.332405 | 2.32E-10 | 3.59E-09 | yes | up | 12.06 | 13.38 | 12.85 | 29.12 | 27.77 | 39.94 |
| YMR135C | 2.128 | 1.089765 | 2.85E-10 | 4.35E-09 | yes | up | 323.7 | 296.3 | 323.22 | 607.3 | 599.18 | 777.02 |
| YER151C | 2.479 | 1.309481 | 2.89E-10 | 4.4E-09 | yes | up | 6.84 | 4.76 | 4.81 | 13.18 | 13 | 14.71 |
| YOR232W | 2.479 | 1.309702 | 3.48E-10 | 5.22E-09 | yes | up | 79.62 | 69.81 | 79.93 | 165.02 | 165.75 | 234.06 |
| YKR075C | 3.445 | 1.784424 | 3.86E-10 | 5.73E-09 | yes | up | 161.18 | 153.68 | 169 | 462.81 | 421.1 | 865.78 |
| YDR054C | 2.562 | 1.357124 | 4.28E-10 | 6.3E-09 | yes | up | 61.84 | 50.78 | 44.38 | 121.27 | 118.95 | 162.83 |
| YJL166W | 2.861 | 1.516631 | 4.77E-10 | 6.96E-09 | yes | up | 3934.75 | 3672.83 | 3989.91 | 9181.48 | 8436.26 | 14265.4 |
| YER050C | 2.828 | 1.499762 | 4.88E-10 | 7.1E-09 | yes | up | 174.61 | 163.8 | 167.78 | 406.33 | 382.95 | 631.39 |
| YBR082C | 2.745 | 1.457045 | 5.8E-10 | 8.31E-09 | yes | up | 885.96 | 736.71 | 791.55 | 1857.36 | 1804.83 | 2909.53 |
| YLR175W | 2.318 | 1.213125 | 6.31E-10 | 8.96E-09 | yes | up | 8.88 | 7.48 | 10.52 | 22.15 | 18.84 | 21.14 |
| YDR029W | 3.44 | 1.782227 | 6.46E-10 | 9.15E-09 | yes | up | 28.13 | 42.82 | 38.81 | 117.38 | 112.09 | 155.65 |
| YPR010C-A | 2.301 | 1.202503 | 6.72E-10 | 9.49E-09 | yes | up | 4214.46 | 5207.52 | 4850.92 | 9599.04 | 9535.07 | 10573.98 |
| YMR193W | 2.018 | 1.013189 | 7.09E-10 | 1E-08 | yes | up | 33.94 | 29.93 | 32.92 | 63.71 | 59.07 | 68.93 |
| YJR014W | 2.077 | 1.054573 | 7.8E-10 | 1.09E-08 | yes | up | 219.18 | 211.61 | 216.4 | 394.97 | 409.27 | 510.25 |
| YOL081W | 3.698 | 1.886651 | 8.02E-10 | 1.12E-08 | yes | up | 7.31 | 6.59 | 7.33 | 20.12 | 20.65 | 43.98 |
| YHR081W | 2.87 | 1.520924 | 8.59E-10 | 1.2E-08 | yes | up | 15.96 | 12.44 | 16.48 | 42.12 | 36.02 | 51.9 |
| YGR274C | 2.901 | 1.536308 | 1.09E-09 | 1.49E-08 | yes | up | 2.74 | 3.23 | 3.07 | 7.97 | 7.19 | 11.8 |
| YOR244W | 2.571 | 1.362567 | 1.61E-09 | 2.14E-08 | yes | up | 22.86 | 15.16 | 19.09 | 47.56 | 41.89 | 58.56 |
| YMR070W | 2.16 | 1.11089 | 1.75E-09 | 2.3E-08 | yes | up | 8.51 | 10.09 | 10.01 | 21.6 | 18.24 | 21.56 |
| YJR052W | 2.308 | 1.206582 | 1.93E-09 | 2.53E-08 | yes | up | 4.71 | 3.68 | 4.76 | 9.43 | 10.82 | 10.1 |
| YMR168C | 2.388 | 1.255743 | 2.28E-09 | 2.97E-08 | yes | up | 3.91 | 4.99 | 4.07 | 9.31 | 11.87 | 10.05 |
| YCL050C | 2.929 | 1.550473 | 2.45E-09 | 3.18E-08 | yes | up | 46.33 | 47.06 | 47.38 | 105.46 | 121.15 | 196.36 |
| YBR290W | 2.407 | 1.267108 | 2.63E-09 | 3.4E-08 | yes | up | 44.68 | 50.44 | 56.36 | 115.53 | 100.78 | 148.07 |
| YML097C | 2.167 | 1.115808 | 3.02E-09 | 3.87E-08 | yes | up | 6.59 | 7.01 | 7.28 | 13.46 | 15.88 | 15.64 |
| YNL075W | 2.322 | 1.215195 | 3.27E-09 | 4.15E-08 | yes | up | 15.26 | 22.12 | 17.94 | 41.32 | 41.69 | 45.34 |
| YGR057C | 2.008 | 1.005584 | 3.34E-09 | 4.22E-08 | yes | up | 23.66 | 20.1 | 22.5 | 45.16 | 41.34 | 44.16 |
| YIL152W | 2.241 | 1.163877 | 3.39E-09 | 4.28E-08 | yes | up | 221.91 | 202.77 | 222.23 | 425.4 | 419.07 | 587.23 |
| YHR092C | 2.789 | 1.479905 | 4.44E-09 | 5.52E-08 | yes | up | 97.22 | 93.77 | 95.06 | 240.69 | 200.88 | 374.52 |
| YOL078W | 2.228 | 1.155671 | 4.64E-09 | 5.76E-08 | yes | up | 2.52 | 2.36 | 2 | 4.79 | 5.06 | 5.49 |
| YDR147W | 2.314 | 1.210199 | 5.1E-09 | 6.26E-08 | yes | up | 6.95 | 6.63 | 5.59 | 15.34 | 17.22 | 12.11 |
| YDR145W | 2.042 | 1.02987 | 5.41E-09 | 6.6E-08 | yes | up | 12.62 | 12.9 | 14.71 | 26.4 | 24.65 | 30.21 |
| YMR044W | 2.267 | 1.180924 | 5.46E-09 | 6.64E-08 | yes | up | 16.13 | 14.79 | 14.41 | 31.34 | 29.88 | 41.25 |
| YDR277C | 3.293 | 1.719222 | 5.69E-09 | 6.9E-08 | yes | up | 140.91 | 130.99 | 147.72 | 359.25 | 360.17 | 739.51 |
| RDN37-2 | 4.024 | 2.008702 | 5.9E-09 | 7.11E-08 | yes | up | 3.81 | 4.99 | 2.26 | 13.07 | 12.33 | 25.07 |
| YDR527W | 2.305 | 1.204569 | 6.18E-09 | 7.41E-08 | yes | up | 31.01 | 30.47 | 35.68 | 65.51 | 66 | 91.98 |
| YPR062W | 2.436 | 1.284704 | 6.61E-09 | 7.92E-08 | yes | up | 1469.42 | 1438.16 | 1410.52 | 3061.65 | 2828.6 | 4487.16 |
| YHR102W | 2.106 | 1.07456 | 7.57E-09 | 8.95E-08 | yes | up | 26.47 | 21.22 | 24.45 | 43.82 | 48.53 | 58.57 |
| YBR022W | 2.183 | 1.126422 | 7.79E-09 | 9.18E-08 | yes | up | 87.08 | 93.76 | 89.97 | 178.78 | 170.47 | 231.09 |
| YOL136C | 3.006 | 1.587701 | 8.31E-09 | 9.71E-08 | yes | up | 44.13 | 43.43 | 46.16 | 111.23 | 104.1 | 201.43 |
| YER049W | 2.312 | 1.209276 | 8.96E-09 | 1.04E-07 | yes | up | 4.81 | 5.63 | 5.5 | 14.98 | 12.03 | 10.12 |
| YDR035W | 2.067 | 1.047654 | 1.26E-08 | 1.41E-07 | yes | up | 1266.08 | 1277.35 | 1231.88 | 2347.5 | 2264.64 | 3101.71 |
| YBL086C | 2.298 | 1.200472 | 1.65E-08 | 1.81E-07 | yes | up | 67.11 | 53.82 | 56.18 | 119.66 | 118.25 | 168.86 |
| YGR137W | 2.264 | 1.17898 | 1.78E-08 | 1.94E-07 | yes | up | 155.94 | 146.51 | 164.57 | 309.38 | 296.67 | 416.49 |
| YBR085C-A | 3.13 | 1.646242 | 1.8E-08 | 1.97E-07 | yes | up | 5058.32 | 5305.79 | 5022.19 | 12343.55 | 12666.17 | 24504.84 |
| YMR255W | 2.161 | 1.111417 | 1.83E-08 | 1.99E-07 | yes | up | 174.18 | 156.56 | 159.85 | 316.65 | 300.36 | 423.84 |
| YMR185W | 2.05 | 1.03555 | 1.93E-08 | 2.09E-07 | yes | up | 5.26 | 4.07 | 5.31 | 9.29 | 10.01 | 10.5 |
| YBL071W-A | 2.758 | 1.463662 | 2.05E-08 | 2.2E-07 | yes | up | 1525.35 | 1516.74 | 1564.22 | 3119.29 | 3406.53 | 5510.26 |
| YML081C-A | 2.141 | 1.098582 | 2.1E-08 | 2.24E-07 | yes | up | 1273.23 | 995.61 | 1048.11 | 2025.98 | 1972.07 | 1900.86 |
| YCR009C | 3.429 | 1.777892 | 2.37E-08 | 2.52E-07 | yes | up | 107.92 | 94.04 | 116.86 | 276.02 | 277.47 | 618.93 |
| YKL157W | 2.001 | 1.000943 | 2.58E-08 | 2.72E-07 | yes | up | 54.65 | 58.84 | 60.65 | 108.36 | 100.6 | 136.16 |
| YPL052W | 2.386 | 1.254306 | 3.06E-08 | 3.15E-07 | yes | up | 31.47 | 33.45 | 32.95 | 67.78 | 65.88 | 100.27 |
| YGR267C | 2.097 | 1.068473 | 3.3E-08 | 3.36E-07 | yes | up | 296.32 | 307.59 | 312.25 | 575.16 | 547.78 | 771.97 |
| YLR333C | 2.629 | 1.394691 | 3.39E-08 | 3.45E-07 | yes | up | 549.02 | 538.02 | 565.28 | 1039.05 | 896.9 | 1293.33 |
| YHR146W | 2.644 | 1.402626 | 3.48E-08 | 3.53E-07 | yes | up | 214.79 | 183.33 | 201.63 | 440.67 | 426.4 | 749.18 |
| YNL139C | 2.019 | 1.01373 | 3.52E-08 | 3.57E-07 | yes | up | 2.04 | 1.81 | 1.86 | 3.97 | 3.48 | 4.01 |
| YPR165W | 2.016 | 1.01154 | 3.56E-08 | 3.6E-07 | yes | up | 378.7 | 362.14 | 362 | 624.37 | 687.86 | 867.49 |
| YBR257W | 2.258 | 1.175242 | 3.72E-08 | 3.75E-07 | yes | up | 13.57 | 16.2 | 14.35 | 31.77 | 28.97 | 38.69 |
| YOR089C | 2.008 | 1.005605 | 4E-08 | 4.01E-07 | yes | up | 154.79 | 160.98 | 157.16 | 320.5 | 256.24 | 355.16 |
| YML125C | 2.07 | 1.049373 | 4.06E-08 | 4.06E-07 | yes | up | 105.46 | 100.34 | 125.02 | 218 | 194.6 | 264.12 |
| YLR327C | 2.185 | 1.127656 | 4.64E-08 | 4.58E-07 | yes | up | 7754.76 | 9002.14 | 7653.83 | 15092.79 | 14904.93 | 19730.75 |
| YAL019W | 2.313 | 1.209897 | 4.64E-08 | 4.58E-07 | yes | up | 2.88 | 1.95 | 2.86 | 6.24 | 6.64 | 5.08 |
| YPR125W | 2.4 | 1.26274 | 4.79E-08 | 4.72E-07 | yes | up | 9.84 | 6.5 | 6.82 | 22.06 | 17.56 | 16.65 |
| YFL001W | 2.356 | 1.236405 | 4.9E-08 | 4.82E-07 | yes | up | 4.41 | 4.43 | 6.02 | 11.38 | 13.58 | 10.35 |
| YLR244C | 2.419 | 1.274582 | 5.13E-08 | 5.04E-07 | yes | up | 296 | 274.24 | 307.93 | 619.34 | 568.88 | 949.62 |
| YDR092W | 2.2 | 1.137486 | 5.25E-08 | 5.14E-07 | yes | up | 719.44 | 682.22 | 674.48 | 1289.16 | 1294.64 | 1881.88 |
| YAR019W-A | 2.002 | 1.001565 | 6.4E-08 | 6.19E-07 | yes | up | 15.51 | 14.99 | 11.71 | 21.65 | 34.46 | 27.19 |
| YOR257W | 2.203 | 1.139671 | 6.78E-08 | 6.52E-07 | yes | up | 235.41 | 226.45 | 234.49 | 412.59 | 462.46 | 629.97 |
| YLR221C | 2.602 | 1.379722 | 6.88E-08 | 6.61E-07 | yes | up | 12.49 | 11.89 | 13.3 | 31.27 | 26.11 | 41.94 |
| YPR143W | 2.4 | 1.262931 | 6.94E-08 | 6.65E-07 | yes | up | 9.2 | 12.54 | 12.36 | 32.73 | 24.48 | 25.51 |
| YOR303W | 2.625 | 1.392503 | 9.92E-08 | 9.21E-07 | yes | up | 970.36 | 955.62 | 898.95 | 2058.11 | 1940.23 | 3579.3 |
| YPR170C | 3.462 | 1.791583 | 1.13E-07 | 1.03E-06 | yes | up | 16.58 | 8.03 | 12.46 | 44.11 | 39.81 | 51.1 |
| YJL152W | 2.24 | 1.16351 | 1.24E-07 | 1.13E-06 | yes | up | 442.24 | 451.59 | 486.6 | 899.12 | 830.89 | 1261.9 |
| YOR036W | 2.028 | 1.020021 | 1.3E-07 | 1.18E-06 | yes | up | 75.99 | 69.52 | 69.28 | 120.62 | 137.73 | 171.76 |
| YMR252C | 2.464 | 1.300833 | 1.43E-07 | 1.28E-06 | yes | up | 984.77 | 1058.32 | 1014.1 | 2064.11 | 2067.83 | 3507.17 |
| RDN37-1 | 2.855 | 1.513665 | 1.5E-07 | 1.34E-06 | yes | up | 9012.26 | 10565.03 | 7619.3 | 19100.34 | 16862.76 | 35336.83 |
| YJL218W | 3.579 | 1.839504 | 1.5E-07 | 1.34E-06 | yes | up | 7.21 | 4.19 | 3.98 | 22.16 | 26.85 | 12.69 |
| YNL244C | 2.124 | 1.086653 | 1.54E-07 | 1.37E-06 | yes | up | 1281.3 | 1160 | 1272.55 | 2248.37 | 2142.87 | 3120.19 |
| YLR336C | 3.051 | 1.609241 | 1.67E-07 | 1.49E-06 | yes | up | 1 | 1.21 | 1.77 | 3.84 | 3.46 | 5.63 |
| YOL080C | 4.727 | 2.240987 | 1.76E-07 | 1.57E-06 | yes | up | 0.55 | 0.66 | 0.6 | 3.35 | 5.07 | 3.02 |
| YDR184C | 2.926 | 1.549053 | 1.78E-07 | 1.58E-06 | yes | up | 3.51 | 4.69 | 5.23 | 12.54 | 11 | 17.68 |
| YDL012C | 2.299 | 1.200757 | 1.86E-07 | 1.64E-06 | yes | up | 581.82 | 611.72 | 486.06 | 1068.05 | 1070.99 | 1574.58 |
| YDR026C | 2.381 | 1.251394 | 1.86E-07 | 1.64E-06 | yes | up | 2.44 | 3.81 | 2.77 | 7.41 | 7.51 | 6.99 |
| YNL002C | 2.618 | 1.388617 | 2.08E-07 | 1.82E-06 | yes | up | 14.29 | 15.28 | 14.5 | 32.65 | 31.03 | 54.39 |
| YDL074C | 2.049 | 1.034963 | 2.24E-07 | 1.94E-06 | yes | up | 3.82 | 4.65 | 4.01 | 7.96 | 8.23 | 9.31 |
| YPR025C | 2.198 | 1.135962 | 2.29E-07 | 1.98E-06 | yes | up | 15.53 | 14.91 | 15.17 | 31.08 | 27.51 | 41.57 |
| YCR035C | 2.044 | 1.031275 | 2.55E-07 | 2.18E-06 | yes | up | 10.25 | 9.02 | 8.32 | 20.19 | 16.25 | 19.66 |
| YNL192W | 2.934 | 1.552678 | 2.95E-07 | 2.51E-06 | yes | up | 19.95 | 20.78 | 21.91 | 47.32 | 46.88 | 99.77 |
| YMR240C | 2.04 | 1.028667 | 3.17E-07 | 2.69E-06 | yes | up | 7.3 | 8.3 | 8.54 | 16.82 | 14.19 | 17.99 |
| YKR014C | 2.462 | 1.299754 | 3.19E-07 | 2.7E-06 | yes | up | 125.69 | 127.52 | 122.66 | 239.99 | 263.09 | 430.92 |
| YOR047C | 2.704 | 1.434872 | 3.29E-07 | 2.77E-06 | yes | up | 4.14 | 3.66 | 2.93 | 10.44 | 7.38 | 12.3 |
| YJL151C | 2.029 | 1.020474 | 3.82E-07 | 3.18E-06 | yes | up | 7098.49 | 6811.6 | 7105.69 | 12255.91 | 11850.19 | 17072.75 |
| YER079W | 2.067 | 1.047579 | 3.83E-07 | 3.19E-06 | yes | up | 165 | 139.4 | 146.53 | 247.32 | 298.21 | 372.94 |
| LSR1 | 2.051 | 1.036039 | 5.65E-07 | 4.6E-06 | yes | up | 490.91 | 509.64 | 463.96 | 938.36 | 806.92 | 1238.37 |
| YKL062W | 2.365 | 1.241845 | 6.73E-07 | 5.4E-06 | yes | up | 14.93 | 13.54 | 20 | 50.36 | 38.84 | 27.99 |
| YJL144W | 2.181 | 1.125284 | 6.89E-07 | 5.52E-06 | yes | up | 1154.31 | 1203.12 | 1094.85 | 1943.16 | 2179.45 | 3078.43 |
| YOR191W | 2.637 | 1.399009 | 7.62E-07 | 6.05E-06 | yes | up | 1.6 | 1.56 | 1.41 | 3.32 | 3.32 | 5.85 |
| YBR142W | 2.998 | 1.583976 | 7.8E-07 | 6.18E-06 | yes | up | 0.88 | 0.72 | 0.95 | 2.88 | 1.93 | 3.36 |
| YGL166W | 2.152 | 1.105682 | 8.07E-07 | 6.34E-06 | yes | up | 576.93 | 566.14 | 552.67 | 1020.99 | 1018.94 | 1581.54 |
| YPL268W | 2.659 | 1.410661 | 8.29E-07 | 6.49E-06 | yes | up | 2.64 | 2.15 | 3.18 | 4.97 | 7.76 | 9.29 |
| YCR021C | 2.929 | 1.550644 | 9.18E-07 | 7.13E-06 | yes | up | 6532.06 | 6071.9 | 6529.72 | 26041.26 | 24525.56 | 9274.27 |
| YKL049C | 2.421 | 1.275734 | 9.57E-07 | 7.41E-06 | yes | up | 21.75 | 17.51 | 19.27 | 41.05 | 38.54 | 63.55 |
| YPL250C | 2.091 | 1.064458 | 9.98E-07 | 7.64E-06 | yes | up | 2157.7 | 2406.46 | 2766.66 | 4325.75 | 4445.45 | 6168.61 |
| YDR464W | 2.131 | 1.091711 | 1.07E-06 | 8.17E-06 | yes | up | 3.19 | 3.06 | 3.16 | 5.61 | 6.05 | 8.44 |
| YBL004W | 2.351 | 1.233037 | 1.1E-06 | 8.36E-06 | yes | up | 0.73 | 0.58 | 0.68 | 1.71 | 1.22 | 1.86 |
| YOR219C | 2.422 | 1.275915 | 1.21E-06 | 9.12E-06 | yes | up | 12.92 | 11.9 | 12.24 | 25.38 | 23.36 | 42.87 |
| YER116C | 2.029 | 1.02065 | 1.29E-06 | 9.72E-06 | yes | up | 56.29 | 50.31 | 55.15 | 99.11 | 90.82 | 134.93 |
| YDR526C | 2.926 | 1.548881 | 1.52E-06 | 1.13E-05 | yes | up | 9.91 | 9.67 | 5.72 | 28.46 | 32.04 | 18.73 |
| YDR343C | 2.747 | 1.457948 | 1.6E-06 | 1.17E-05 | yes | up | 2714.46 | 2408.89 | 3065.43 | 11393.07 | 8367.07 | 4176.88 |
| YER002W | 2.62 | 1.389489 | 1.85E-06 | 1.33E-05 | yes | up | 14.65 | 15.32 | 12.47 | 29.26 | 31.63 | 53.84 |
| YOR123C | 2.087 | 1.061582 | 1.93E-06 | 1.38E-05 | yes | up | 18.24 | 17.55 | 15.77 | 31.52 | 30.68 | 45.34 |
| YBR119W | 2.171 | 1.118478 | 1.94E-06 | 1.38E-05 | yes | up | 40.51 | 40.58 | 38 | 76.71 | 68.97 | 112.96 |
| YPL033C | 2.945 | 1.558342 | 2E-06 | 1.43E-05 | yes | up | 1.87 | 2.29 | 2.27 | 5.18 | 7.97 | 7.27 |
| YOL108C | 2.137 | 1.095677 | 2.24E-06 | 1.58E-05 | yes | up | 201.42 | 196.86 | 186.78 | 361.47 | 324.78 | 532.92 |
| YPR133C | 2.165 | 1.114334 | 2.44E-06 | 1.71E-05 | yes | up | 16.11 | 14.7 | 18.58 | 32.67 | 28.91 | 45.65 |
| YLR067C | 2.057 | 1.040848 | 2.59E-06 | 1.81E-05 | yes | up | 2.86 | 2.34 | 2.12 | 5.54 | 5.48 | 4.15 |
| YPR065W | 2.818 | 1.494559 | 2.73E-06 | 1.89E-05 | yes | up | 125.14 | 112.13 | 113.88 | 499.51 | 397.02 | 168.22 |
| YLR460C | 2.55 | 1.350635 | 2.88E-06 | 1.98E-05 | yes | up | 6.81 | 4.58 | 4.53 | 19.16 | 11.85 | 11.3 |
| YGL184C | 2.633 | 1.396546 | 2.9E-06 | 2E-05 | yes | up | 63.6 | 59.47 | 64.58 | 130.49 | 121.81 | 261.85 |
| YJR025C | 2.539 | 1.344436 | 3.03E-06 | 2.08E-05 | yes | up | 438.47 | 420.15 | 411.81 | 832.68 | 815.58 | 1636.88 |
| YNL271C | 2.069 | 1.049187 | 3.1E-06 | 2.12E-05 | yes | up | 2.37 | 2.05 | 2.2 | 3.97 | 4.02 | 5.75 |
| YPR158W | 2.621 | 1.389876 | 3.13E-06 | 2.14E-05 | yes | up | 27.54 | 19.52 | 18.78 | 40.34 | 55.7 | 82.63 |
| YPR126C | 2.572 | 1.362769 | 3.17E-06 | 2.15E-05 | yes | up | 46.67 | 25.65 | 25.57 | 93.01 | 83.81 | 71.36 |
| 21S_rRNA | 2.298 | 1.200139 | 0.002092 | 0.006974 | yes | up | 0.34 | 0.39 | 0.33 | 1.28 | 0.56 | 0.86 |
| ENSRNA049651861 | 2.542 | 1.34568 | 4.88E-05 | 0.000253 | yes | up | 527.18 | 463.37 | 584.72 | 1015.82 | 864.41 | 1703.71 |
| ENSRNA049651872 | 2.14 | 1.097381 | 0.001877 | 0.006363 | yes | up | 232.6 | 214.54 | 205.16 | 298.61 | 323.38 | 823.76 |
| ENSRNA049652191 | 2.672 | 1.417768 | 0.000109 | 0.000515 | yes | up | 4226.1 | 5914.37 | 3125.26 | 7169.27 | 7388.14 | 15475.39 |
| ENSRNA049652654 | 2.417 | 1.273143 | 0.004982 | 0.014838 | yes | up | 564.84 | 128.94 | 672.98 | 1138.95 | 614.53 | 1522.81 |
| ENSRNA049652728 | 2.107 | 1.075288 | 0.014173 | 0.036391 | yes | up | 140.15 | 49.42 | 104.59 | 190.86 | 136.05 | 270.7 |
| RDN25-1 | 2.318 | 1.212823 | 4.69E-05 | 0.000244 | yes | up | 2789.23 | 3856.38 | 2364.87 | 6219.43 | 5153.67 | 10281.97 |
| RDN25-2 | 2.523 | 1.335199 | 4.55E-06 | 3E-05 | yes | up | 2454.45 | 3309.54 | 2278.22 | 5856.18 | 5085.91 | 10144.52 |
| snR191 | 2.84 | 1.50612 | 0.002531 | 0.008245 | yes | up | 22.45 | 5.49 | 16.56 | 31.44 | 32.41 | 93.61 |
| snR4 | 2.542 | 1.34568 | 4.88E-05 | 0.000253 | yes | up | 527.18 | 463.37 | 584.72 | 1015.82 | 864.41 | 1703.71 |
| snR63 | 2.959 | 1.565069 | 0.000726 | 0.002773 | yes | up | 21.98 | 18.53 | 26.46 | 36.24 | 67.28 | 129.07 |
| snR81 | 2.429 | 1.280543 | 0.019359 | 0.046987 | yes | up | 23.26 | 16.7 | 6.88 | 62.82 | 49.61 | 48.6 |
| YAL018C | 2.58 | 1.367334 | 0.008924 | 0.024424 | yes | up | 0.45 | 0.17 | 0.37 | 1.79 | 1.21 | 0.7 |
| YAR009C | 3.848 | 1.944155 | 1.41E-05 | 8.24E-05 | yes | up | 0.21 | 0.25 | 0.31 | 0.94 | 0.7 | 2.37 |
| YAR029W | 2.731 | 1.449517 | 0.003501 | 0.010973 | yes | up | 16.36 | 10.09 | 25.87 | 54.09 | 42.39 | 77.54 |
| YBL071C | 2.428 | 1.279693 | 3.22E-05 | 0.000175 | yes | up | 355.49 | 264.56 | 327.61 | 536.59 | 587.42 | 1141.24 |
| YBL093C | 2.568 | 1.360757 | 4.66E-06 | 3.06E-05 | yes | up | 417.01 | 448.82 | 393.24 | 831.86 | 812.23 | 1688.38 |
| YBR034C | 2.07 | 1.049311 | 1.92E-05 | 0.000109 | yes | up | 10.16 | 10.2 | 9.34 | 25.49 | 22.46 | 14.33 |
| YBR210W | 2.096 | 1.067894 | 0.005584 | 0.016326 | yes | up | 18.93 | 28.06 | 21.21 | 35.14 | 31.64 | 83.71 |
| YBR244W | 2.143 | 1.099687 | 1.65E-05 | 9.47E-05 | yes | up | 12.38 | 10.93 | 11.95 | 22.41 | 24.95 | 27.86 |
| YBR301W | 2.196 | 1.134837 | 0.028231 | 0.064216 | yes | up | 1.83 | 1.6 | 4.66 | 6.44 | 5.77 | 10.2 |
| YCL035C | 2.274 | 1.185447 | 4.26E-05 | 0.000225 | yes | up | 1225.55 | 1155.68 | 1110.77 | 1956.55 | 1965.13 | 3873.17 |
| YCL058C | 2.256 | 1.17404 | 1.63E-05 | 9.41E-05 | yes | up | 22.68 | 17.26 | 30.03 | 56.54 | 42.38 | 58.7 |
| YCL067C | 2.017 | 1.012333 | 5.41E-05 | 0.000276 | yes | up | 81.77 | 77.33 | 81.51 | 138.91 | 124.83 | 219.29 |
| YCR039C | 2.017 | 1.012333 | 5.41E-05 | 0.000276 | yes | up | 81.77 | 77.33 | 81.51 | 138.91 | 124.83 | 219.29 |
| YDL009C | 2.858 | 1.515197 | 9.89E-06 | 6.07E-05 | yes | up | 36.58 | 36.78 | 38.65 | 83.99 | 79.51 | 167.68 |
| YDL010W | 2.063 | 1.044729 | 6.75E-06 | 4.28E-05 | yes | up | 73.58 | 73.93 | 75.58 | 131.88 | 125.1 | 200.2 |
| YDL011C | 2.018 | 1.013255 | 0.000199 | 0.000884 | yes | up | 106.1 | 109.02 | 76.43 | 143.75 | 184.45 | 242.28 |
| YDL031W | 2.247 | 1.168173 | 1.31E-05 | 7.69E-05 | yes | up | 1.25 | 0.93 | 1.16 | 2.96 | 2.8 | 1.96 |
| YDL065C | 2.036 | 1.025646 | 0.000592 | 0.002326 | yes | up | 16.11 | 12.58 | 14.69 | 25.2 | 21.07 | 43.66 |
| YDL153C | 2.204 | 1.140428 | 0.001037 | 0.003812 | yes | up | 2.46 | 1.29 | 2.26 | 4.07 | 3.34 | 6.58 |
| YDL234C | 2.301 | 1.202082 | 2.19E-05 | 0.000123 | yes | up | 41.45 | 35.36 | 33.58 | 64.2 | 69.28 | 127.01 |
| YDR028C | 2.039 | 1.02805 | 0.000816 | 0.003084 | yes | up | 7.69 | 8.42 | 9.57 | 13.48 | 12.93 | 27.39 |
| YDR042C | 2.103 | 1.072443 | 0.012159 | 0.031902 | yes | up | 1.88 | 2.11 | 1.54 | 5.25 | 2.88 | 4.87 |
| YDR210W | 2.168 | 1.116473 | 0.014611 | 0.037326 | yes | up | 5.38 | 14.28 | 1.86 | 6.8 | 3.49 | 10.84 |
| YDR289C | 2.133 | 1.093194 | 5.66E-05 | 0.000288 | yes | up | 4.66 | 4.75 | 3.73 | 8.75 | 7.68 | 12.05 |
| YDR299W | 2.065 | 1.046166 | 0.000992 | 0.003664 | yes | up | 1.29 | 1.46 | 0.9 | 3.04 | 2.5 | 2.36 |
| YDR345C | 3.092 | 1.628579 | 3.91E-06 | 2.61E-05 | yes | up | 161.21 | 148.15 | 144.17 | 350.29 | 308.48 | 882.37 |
| YDR397C | 2.082 | 1.0581 | 3.54E-05 | 0.00019 | yes | up | 85.61 | 80.99 | 109.46 | 157.07 | 161.72 | 248.62 |
| YDR446W | 2.217 | 1.148369 | 0.034112 | 0.075308 | yes | up | 0.4 | 0.38 | 0.1 | 1.25 | 0.82 | 0.77 |
| YDR457W | 2.506 | 1.325564 | 7.26E-05 | 0.000359 | yes | up | 3.2 | 2.72 | 3.4 | 5.77 | 5.56 | 13.47 |
| YDR485C | 2.103 | 1.072497 | 2.09E-05 | 0.000118 | yes | up | 5.3 | 5.22 | 5.09 | 9.66 | 8.75 | 14.73 |
| YDR532C | 2.373 | 1.246918 | 0.000467 | 0.001878 | yes | up | 1.94 | 3.14 | 3.2 | 4.38 | 6.95 | 9.77 |
| YEL012W | 2.066 | 1.046801 | 1.3E-05 | 7.68E-05 | yes | up | 535.92 | 517.14 | 518.24 | 865.74 | 904.52 | 1456.92 |
| YER044C-A | 2.225 | 1.153804 | 0.036765 | 0.080161 | yes | up | 0.6 | 0.19 | 0.07 | 0.42 | 1.04 | 1.44 |
| YER098W | 2.016 | 1.011368 | 4.27E-06 | 2.83E-05 | yes | up | 12.61 | 8.52 | 8.19 | 20.91 | 17.74 | 20.55 |
| YER130C | 2.802 | 1.486332 | 7.11E-06 | 4.47E-05 | yes | up | 11.49 | 9.23 | 10.07 | 24.29 | 19.43 | 48.38 |
| YER132C | 2.233 | 1.158679 | 1.14E-05 | 6.87E-05 | yes | up | 2.04 | 1.19 | 1.44 | 3.59 | 2.9 | 4.15 |
| YER149C | 2.082 | 1.057683 | 0.000119 | 0.000561 | yes | up | 2.5 | 2.07 | 2.21 | 5.04 | 4.14 | 5.23 |
| YER158W-A | 2.191 | 1.131659 | 0.001811 | 0.006166 | yes | up | 371.25 | 273.18 | 267.04 | 414.02 | 455.8 | 998.96 |
| YER162C | 2.025 | 1.017843 | 0.000405 | 0.001656 | yes | up | 13.29 | 13.6 | 14.39 | 22.71 | 20.5 | 41.89 |
| YER165C-A | 3.456 | 1.789096 | 0.000166 | 0.000754 | yes | up | 1.96 | 3.43 | 12.93 | 29.26 | 29.08 | 25.17 |
| YER167W | 2.056 | 1.039886 | 0.000101 | 0.00048 | yes | up | 7.89 | 5.51 | 7.42 | 17.85 | 16.62 | 9.34 |
| YFL026W | 2.396 | 1.26047 | 5.7E-05 | 0.00029 | yes | up | 7.1 | 7.06 | 10.28 | 15.02 | 16.21 | 29.72 |
| YFL034C-A | 3.218 | 1.686024 | 0.000663 | 0.00256 | yes | up | 2.61 | 4.14 | 11.24 | 25.32 | 12.08 | 61.8 |
| YFL046W | 2.233 | 1.159069 | 0.000115 | 0.000544 | yes | up | 36.33 | 38.56 | 38.47 | 67.04 | 62.38 | 128.82 |
| YFR039C | 2.085 | 1.060192 | 0.000116 | 0.000548 | yes | up | 3.6 | 4.3 | 3.69 | 7.43 | 6.4 | 10.75 |
| YGL005C | 2.106 | 1.074378 | 9.27E-05 | 0.000445 | yes | up | 30.36 | 26.41 | 31.81 | 52.02 | 47.59 | 88.72 |
| YGL013C | 2.308 | 1.206698 | 6.74E-06 | 4.28E-05 | yes | up | 5.87 | 4.51 | 5.96 | 11.21 | 9.77 | 17.49 |
| YGL089C | 2.706 | 1.436039 | 0.001325 | 0.004729 | yes | up | 4.74 | 6.92 | 2.33 | 11.12 | 19.04 | 15.02 |
| YGL094C | 2.413 | 1.270684 | 8.84E-05 | 0.000428 | yes | up | 2.65 | 1.82 | 2.33 | 4.01 | 4.6 | 8.66 |
| YGL165C | 2.086 | 1.061063 | 4.53E-05 | 0.000237 | yes | up | 282.87 | 279.19 | 266.62 | 461.18 | 454.92 | 809.03 |
| YGL183C | 2.322 | 1.215625 | 0.000989 | 0.003655 | yes | up | 6.13 | 6.06 | 6.78 | 14.74 | 8.6 | 23.76 |
| YGR114C | 3.132 | 1.647208 | 0.00172 | 0.005891 | yes | up | 3.29 | 0.03 | 2.31 | 8.78 | 7.8 | 11.01 |
| YGR115C | 3.237 | 1.694809 | 0.001371 | 0.004852 | yes | up | 0 | 1.04 | 0.8 | 1.97 | 3.63 | 4.22 |
| YGR139W | 2.119 | 1.083053 | 0.040725 | 0.08703 | yes | up | 5.86 | 4.56 | 2.14 | 8.58 | 4.93 | 20.27 |
| YGR187C | 2.09 | 1.063388 | 0.000624 | 0.002433 | yes | up | 1.39 | 2.19 | 1.74 | 3.6 | 4.21 | 3.75 |
| YGR264C | 2.142 | 1.098655 | 0.00036 | 0.001494 | yes | up | 3.17 | 3.83 | 3.67 | 5.7 | 6.29 | 11.59 |
| YHR046C | 2.442 | 1.287805 | 2.94E-05 | 0.000161 | yes | up | 2.88 | 4.86 | 5.2 | 14.1 | 9.46 | 9.6 |
| YHR052W | 2.2 | 1.137184 | 0.000289 | 0.001239 | yes | up | 2.52 | 1.76 | 2.46 | 6.7 | 4.62 | 4.13 |
| YHR068W | 2.294 | 1.198045 | 1.41E-05 | 8.23E-05 | yes | up | 7.92 | 7.8 | 6.29 | 12.09 | 17.36 | 22.21 |
| YHR082C | 2.639 | 1.400258 | 9.86E-06 | 6.06E-05 | yes | up | 9.64 | 11.15 | 12.23 | 20.8 | 23.28 | 47.91 |
| YHR088W | 2.384 | 1.253585 | 5.56E-05 | 0.000283 | yes | up | 23.63 | 20.72 | 21.87 | 38.09 | 41.88 | 83.56 |
| YHR099W | 3.023 | 1.59601 | 9.2E-05 | 0.000441 | yes | up | 1.76 | 1.52 | 1.35 | 3.08 | 2.86 | 10.37 |
| YHR162W | 2.103 | 1.072146 | 2.93E-05 | 0.000161 | yes | up | 5232.16 | 5889.94 | 5173.5 | 9229.74 | 8865.52 | 15303.54 |
| YHR207C | 2.655 | 1.408504 | 1.13E-05 | 6.81E-05 | yes | up | 5.47 | 4.15 | 6 | 10 | 12.02 | 21.74 |
| YIL019W | 2.051 | 1.036147 | 0.000313 | 0.001322 | yes | up | 4.54 | 4.39 | 6.85 | 11.45 | 8.4 | 13.07 |
| YIL053W | 3.52 | 1.815671 | 8.02E-06 | 5.01E-05 | yes | up | 88.72 | 104.95 | 112.12 | 659.1 | 523.29 | 124.03 |
| YIL158W | 2.456 | 1.296116 | 0.005277 | 0.015592 | yes | up | 1.27 | 0.85 | 3.35 | 4.71 | 4.46 | 6.97 |
| YJL050W | 2.38 | 1.251168 | 1.26E-05 | 7.5E-05 | yes | up | 1.48 | 1.83 | 2.16 | 4.43 | 3.22 | 5.81 |
| YJL202C | 2.368 | 1.243524 | 0.005372 | 0.015823 | yes | up | 6.88 | 5.83 | 6.59 | 13.96 | 11.35 | 26.36 |
| YJL203W | 2.072 | 1.050794 | 4.8E-05 | 0.00025 | yes | up | 15.69 | 10.19 | 10.05 | 24.76 | 20.88 | 29.18 |
| YJR047C | 2.415 | 1.272083 | 0.000334 | 0.001397 | yes | up | 5.71 | 6.39 | 5.77 | 10.11 | 16.93 | 18.66 |
| YJR078W | 2.393 | 1.258635 | 2.41E-05 | 0.000135 | yes | up | 44.66 | 43.5 | 41.24 | 137.37 | 132.04 | 55.52 |
| YJR097W | 3.238 | 1.695027 | 4.9E-05 | 0.000254 | yes | up | 2.49 | 2.54 | 1.01 | 7.41 | 9.33 | 6.85 |
| YKL061W | 2.043 | 1.030904 | 0.00014 | 0.000647 | yes | up | 99.68 | 62.1 | 104.79 | 169.81 | 141.97 | 216.51 |
| YKL076C | 2.079 | 1.056151 | 0.001685 | 0.005784 | yes | up | 20.66 | 9.2 | 15.51 | 32 | 26.48 | 36.35 |
| YKL082C | 2.36 | 1.238824 | 0.000295 | 0.001262 | yes | up | 1.83 | 2 | 2.44 | 4.46 | 3.72 | 7.53 |
| YKL105C | 2.197 | 1.135557 | 0.008798 | 0.02414 | yes | up | 7.07 | 6.99 | 6.44 | 24.09 | 24.18 | 3.96 |
| YKL106W | 2.017 | 1.011925 | 1.77E-05 | 0.000101 | yes | up | 10.77 | 11.44 | 10.52 | 23.76 | 27.53 | 15.41 |
| YKL152C | 2.713 | 1.439682 | 0.000432 | 0.001752 | yes | up | 759.08 | 790.94 | 880.91 | 3612.58 | 3374.09 | 686.96 |
| YKL153W | 2.649 | 1.405291 | 0.00024 | 0.001044 | yes | up | 373.37 | 375.91 | 383.68 | 1547.86 | 1443.52 | 360.91 |
| YKL172W | 2.334 | 1.222503 | 0.000117 | 0.000554 | yes | up | 4.22 | 3.36 | 5.5 | 7.9 | 8.99 | 14.88 |
| YKL202W | 2.321 | 1.214808 | 0.023682 | 0.055608 | yes | up | 0.45 | 0.75 | 0.82 | 1.21 | 2.04 | 3.18 |
| YKR054C | 2.116 | 1.081574 | 0.005603 | 0.016344 | yes | up | 0.83 | 0.71 | 0.59 | 0.86 | 1.14 | 2.88 |
| YLR106C | 2.208 | 1.142855 | 0.002051 | 0.006864 | yes | up | 0.66 | 0.56 | 0.65 | 0.99 | 0.89 | 2.56 |
| YLR154W-E | 2.545 | 1.347465 | 0.018263 | 0.045067 | yes | up | 1.13 | 0.62 | 0 | 2.08 | 0.73 | 5.64 |
| YLR223C | 2.234 | 1.159357 | 0.001362 | 0.004829 | yes | up | 0.69 | 0.56 | 0.52 | 1.03 | 1.13 | 2.06 |
| YLR225C | 2.214 | 1.14649 | 5.74E-05 | 0.000291 | yes | up | 11.58 | 9.36 | 7.29 | 17.47 | 17.69 | 28.77 |
| YLR297W | 2.05 | 1.035633 | 0.000452 | 0.001824 | yes | up | 207.04 | 186.67 | 158.21 | 276.91 | 291.54 | 550.46 |
| YLR300W | 2.101 | 1.070755 | 0.000613 | 0.002397 | yes | up | 17.45 | 18.26 | 21.46 | 50.05 | 54.65 | 20.89 |
| YLR331C | 2.176 | 1.121869 | 0.039843 | 0.085607 | yes | up | 1.24 | 8.27 | 20.94 | 35.18 | 48.95 | 9.94 |
| YLR345W | 2.134 | 1.09377 | 1.09E-05 | 6.62E-05 | yes | up | 94.63 | 86.71 | 87.13 | 151.18 | 161.33 | 264.37 |
| YLR435W | 2.472 | 1.305846 | 0.002971 | 0.009519 | yes | up | 1.26 | 2.03 | 4.06 | 10.08 | 6.63 | 4.67 |
| YLR447C | 2.338 | 1.225422 | 0.000165 | 0.00075 | yes | up | 54.39 | 61.57 | 61.85 | 102.09 | 99.32 | 232.63 |
| YLR453C | 2.331 | 1.221098 | 0.003066 | 0.009794 | yes | up | 1.88 | 1.39 | 2.89 | 3.51 | 3.76 | 8.71 |
| YML034C-A | 2.463 | 1.300463 | 2.17E-05 | 0.000122 | yes | up | 33.42 | 19.81 | 22.35 | 52.23 | 51.47 | 83.05 |
| YML034W | 2.398 | 1.261587 | 0.000338 | 0.001413 | yes | up | 23.23 | 17.83 | 18.7 | 33.75 | 32.94 | 86.34 |
| YML054C-A | 2.134 | 1.093277 | 0.032162 | 0.071526 | yes | up | 73.05 | 205.24 | 191.25 | 387.88 | 458.61 | 218.74 |
| YML058W-A | 2.29 | 1.195374 | 0.021354 | 0.050915 | yes | up | 26.07 | 37 | 11.61 | 94.76 | 43.57 | 69.56 |
| YMR114C | 2.065 | 1.046204 | 0.000105 | 0.000497 | yes | up | 186.83 | 166.2 | 170.43 | 280.23 | 287.65 | 523.19 |
| YMR122W-A | 2.075 | 1.053015 | 0.003965 | 0.012212 | yes | up | 472.64 | 607.94 | 726.71 | 1804.08 | 1486.17 | 523.56 |
| YMR141W-A | 2.378 | 1.249737 | 0.023496 | 0.055213 | yes | up | 12.27 | 10.09 | 14.78 | 10.82 | 56.52 | 58.16 |
| YMR191W | 2.159 | 1.110144 | 0.00045 | 0.001817 | yes | up | 1136.63 | 1086.23 | 1098.03 | 1700.93 | 1761.48 | 3932.19 |
| YMR193C-A | 3.175 | 1.666929 | 1.55E-05 | 8.98E-05 | yes | up | 5.43 | 12.97 | 18.1 | 38.88 | 37.36 | 54.25 |
| YMR201C | 2.253 | 1.172001 | 4.18E-06 | 2.77E-05 | yes | up | 15.98 | 16.83 | 14.77 | 32.18 | 27.88 | 48.34 |
| YMR239C | 2.218 | 1.14894 | 0.000151 | 0.000689 | yes | up | 1.56 | 2.5 | 2.09 | 5.36 | 3.66 | 5.15 |
| YMR269W | 3.096 | 1.630264 | 1.94E-05 | 0.00011 | yes | up | 2.92 | 2.9 | 4.04 | 9.43 | 7.91 | 16.99 |
| YMR270C | 2.302 | 1.202882 | 2.75E-05 | 0.000152 | yes | up | 4 | 2.56 | 4.23 | 6.96 | 10.4 | 8.2 |
| YMR280C | 2.081 | 1.057248 | 1.17E-05 | 7.04E-05 | yes | up | 14.41 | 13.49 | 13.39 | 23.82 | 23.71 | 38.92 |
| YMR299C | 2.543 | 1.346374 | 3.37E-05 | 0.000182 | yes | up | 14.25 | 13.87 | 16.61 | 31.58 | 26.08 | 61.84 |
| YMR311C | 2.255 | 1.173376 | 3.9E-06 | 2.61E-05 | yes | up | 416.18 | 389.28 | 365.46 | 696.15 | 727.55 | 1227.08 |
| YMR316W | 2.029 | 1.020792 | 0.00036 | 0.001493 | yes | up | 114.96 | 110.02 | 89.46 | 159.78 | 171.89 | 315.34 |
| YMR320W | 2.021 | 1.015214 | 0.000902 | 0.003367 | yes | up | 79.07 | 60 | 90.7 | 136.47 | 109.89 | 205.05 |
| YNL065W | 2.506 | 1.32519 | 3.21E-05 | 0.000174 | yes | up | 8.6 | 9.85 | 9.84 | 34.83 | 28.32 | 12.51 |
| YNL068C | 2.023 | 1.016516 | 3.71E-06 | 2.49E-05 | yes | up | 11.72 | 9.3 | 10.86 | 19.42 | 18.21 | 26.77 |
| YNL093W | 2.024 | 1.01739 | 9.63E-05 | 0.000461 | yes | up | 319.2 | 317.77 | 308.81 | 511.55 | 500.54 | 901.01 |
| YNL113W | 2.043 | 1.030743 | 0.000245 | 0.001065 | yes | up | 29.18 | 19.92 | 19.79 | 38.59 | 42.37 | 58.46 |
| YNL129W | 2.169 | 1.117027 | 0.015954 | 0.040214 | yes | up | 2.93 | 3.55 | 3.58 | 7.84 | 2.72 | 14.82 |
| YNL133C | 2.217 | 1.148761 | 3.68E-05 | 0.000197 | yes | up | 40.85 | 47.58 | 39.23 | 79.56 | 73.03 | 132.39 |
| YNL162W-A | 2.26 | 1.176427 | 0.041905 | 0.088923 | yes | up | 4.74 | 15.5 | 0 | 8.27 | 64.77 | 29.6 |
| YNL171C | 3.347 | 1.74267 | 0.000955 | 0.003537 | yes | up | 2.26 | 1.02 | 1.11 | 4.47 | 9.37 | 9.74 |
| YNL248C | 2.086 | 1.06089 | 0.005428 | 0.015943 | yes | up | 2.55 | 1.49 | 2.92 | 5.43 | 2.92 | 7.16 |
| YNL308C | 2.813 | 1.492058 | 1.01E-05 | 6.18E-05 | yes | up | 5.11 | 5.24 | 6.03 | 12.19 | 10.89 | 26.45 |
| YNR067C | 2.019 | 1.013607 | 0.003914 | 0.012076 | yes | up | 1.52 | 1.86 | 1.59 | 2.81 | 2.16 | 5.56 |
| YOL014W | 2.033 | 1.023922 | 0.001993 | 0.006699 | yes | up | 62.05 | 68.58 | 45.78 | 82.41 | 97.58 | 180.22 |
| YOL016C | 2.295 | 1.198655 | 0.000151 | 0.00069 | yes | up | 137.77 | 143.7 | 130.91 | 237.55 | 224.96 | 520.86 |
| YOL029C | 2.778 | 1.474012 | 6.8E-05 | 0.000339 | yes | up | 2.42 | 3.14 | 3.62 | 6.17 | 11.24 | 10.72 |
| YOL154W | 2.195 | 1.134205 | 9.47E-06 | 5.86E-05 | yes | up | 35.28 | 31.67 | 34.44 | 65.1 | 57.24 | 101.19 |
| YOR051C | 2.091 | 1.064413 | 0.004432 | 0.013422 | yes | up | 4.61 | 5.58 | 3.49 | 7.01 | 6.86 | 16.65 |
| YOR075W | 2.371 | 1.245725 | 0.000257 | 0.001112 | yes | up | 8.09 | 7.06 | 7.02 | 12.24 | 13.98 | 29.28 |
| YOR077W | 2.463 | 1.300338 | 7.81E-06 | 4.89E-05 | yes | up | 6.03 | 6.09 | 8 | 14.84 | 14.2 | 21.85 |
| YOR139C | 2.391 | 1.257497 | 4.73E-05 | 0.000246 | yes | up | 27.88 | 11.66 | 20.38 | 54.11 | 44.96 | 45.68 |
| YOR210W | 2.368 | 1.243792 | 0.000256 | 0.00111 | yes | up | 99.53 | 148.62 | 158.32 | 368.16 | 229.09 | 332.99 |
| YOR220W | 2.135 | 1.094524 | 3.65E-05 | 0.000195 | yes | up | 2908.06 | 2706.46 | 2717.98 | 4679.37 | 4688.54 | 8539.88 |
| YOR242C | 2.783 | 1.47668 | 0.009434 | 0.025633 | yes | up | 0.15 | 0 | 0.39 | 1.03 | 0.39 | 1.69 |
| YOR287C | 2.001 | 1.00103 | 0.034579 | 0.076153 | yes | up | 2.31 | 1.14 | 0.41 | 2.1 | 3.64 | 3.32 |
| YOR304W | 2.042 | 1.029891 | 6.97E-06 | 4.39E-05 | yes | up | 2.08 | 1.55 | 1.78 | 4.01 | 4.16 | 2.98 |
| YOR308C | 2.144 | 1.100568 | 1.19E-05 | 7.15E-05 | yes | up | 2.36 | 1.76 | 1.97 | 4.94 | 3.99 | 4.3 |
| YOR319W | 2.105 | 1.073851 | 6.28E-06 | 4E-05 | yes | up | 30.23 | 29.81 | 21.12 | 56.42 | 47.23 | 66.75 |
| YOR385W | 2.266 | 1.180044 | 0.000584 | 0.002297 | yes | up | 31.05 | 28.4 | 24.57 | 43.75 | 46.59 | 109.42 |
| YPL043W | 2.507 | 1.326126 | 0.000151 | 0.00069 | yes | up | 1.29 | 0.55 | 0.9 | 2.47 | 2.03 | 2.91 |
| YPL081W | 2.19 | 1.130977 | 1.64E-05 | 9.43E-05 | yes | up | 21.63 | 23.86 | 39.47 | 69.3 | 55.99 | 62.32 |
| YPL095C | 2.017 | 1.012523 | 0.003291 | 0.010393 | yes | up | 116.88 | 108.48 | 101.26 | 148.88 | 151.36 | 385.25 |
| YPL232W | 2.273 | 1.184382 | 6.88E-05 | 0.000342 | yes | up | 288.35 | 262.83 | 278.34 | 475.06 | 471.69 | 984.56 |
| YPL257W-B | 2.131 | 1.091746 | 2.74E-05 | 0.000152 | yes | up | 4.53 | 4.31 | 4.35 | 7.6 | 7.67 | 13.23 |
| YPR049C | 2.34 | 1.226679 | 7.38E-05 | 0.000364 | yes | up | 5.83 | 6.56 | 7.13 | 11.96 | 11.24 | 24.33 |
| YPR087W | 2.136 | 1.09478 | 0.00032 | 0.001349 | yes | up | 57.9 | 48.12 | 53.79 | 130.67 | 69.4 | 134.63 |
| YPR142C | 2.171 | 1.118078 | 0.010862 | 0.028978 | yes | up | 4.82 | 1.27 | 2.88 | 9.47 | 6.58 | 5.8 |
| YPR167C | 2.125 | 1.087274 | 0.000514 | 0.00204 | yes | up | 32.5 | 30.47 | 34.39 | 50.6 | 52 | 109.4 |
| YPR169W-A | 2.376 | 1.248719 | 0.005734 | 0.016678 | yes | up | 63.82 | 37.64 | 25.04 | 65.68 | 97.94 | 148.86 |
| ICR1 | 0.4 | -1.32308 | 1.35E-12 | 2.83E-11 | yes | down | 9.75 | 8.09 | 10.98 | 3.5 | 3.87 | 3.58 |
| tS(AGA)H | 0.1 | -3.32306 | 5.78E-09 | 6.99E-08 | yes | down | 0.64 | 8.26 | 3.05 | 0 | 0 | 0 |
| YAL012W | 0.325 | -1.61961 | 7.43E-20 | 3.14E-18 | yes | down | 85.08 | 79.87 | 95.97 | 26.65 | 31.7 | 22.19 |
| YAL021C | 0.449 | -1.15373 | 0.000277 | 0.001191 | yes | down | 111.99 | 109.99 | 124.85 | 60.64 | 59.94 | 22.16 |
| YAL022C | 0.45 | -1.15066 | 4.72E-06 | 3.1E-05 | yes | down | 107.03 | 111.97 | 106.22 | 57.31 | 52.32 | 28.11 |
| YAL040C | 0.435 | -1.20125 | 0.001036 | 0.003809 | yes | down | 6.83 | 8.3 | 7.11 | 4.46 | 2.91 | 1.2 |
| YAL043C | 0.436 | -1.19865 | 0.000366 | 0.001516 | yes | down | 180.95 | 173.44 | 184.08 | 95.72 | 86.31 | 29.89 |
| YAL054C | 0.167 | -2.5826 | 1.98E-09 | 2.59E-08 | yes | down | 4293.89 | 4365.56 | 3623.01 | 393.64 | 888.91 | 129.53 |
| YAL058W | 0.456 | -1.13396 | 1.08E-07 | 9.96E-07 | yes | down | 33.02 | 34.21 | 39.12 | 17.21 | 17.82 | 11.01 |
| YAL061W | 0.367 | -1.44566 | 3.17E-06 | 2.15E-05 | yes | down | 4669.94 | 5106.9 | 4566.36 | 1988.04 | 1999.6 | 760.57 |
| YAL062W | 0.374 | -1.4201 | 4.35E-09 | 5.43E-08 | yes | down | 2714.32 | 2723.23 | 2837.19 | 1161.97 | 1122.42 | 603.72 |
| YAR008W | 0.386 | -1.37361 | 2.5E-05 | 0.000139 | yes | down | 11.47 | 9.77 | 14.65 | 4.16 | 5.75 | 2.43 |
| YAR010C | 0.353 | -1.50279 | 2E-28 | 1.95E-26 | yes | down | 107 | 102.88 | 109.24 | 40.11 | 34.77 | 33.23 |
| YAR035W | 0.428 | -1.22456 | 0.000303 | 0.001288 | yes | down | 6022.68 | 6038.34 | 5474.5 | 2243.05 | 3498.79 | 1014.42 |
| YAR073W | 0.361 | -1.47027 | 1.18E-05 | 7.07E-05 | yes | down | 3.61 | 6.3 | 5.84 | 1.43 | 1.56 | 1.99 |
| YBL024W | 0.464 | -1.10665 | 0.000606 | 0.002372 | yes | down | 8.7 | 11.09 | 13.82 | 6.27 | 5.52 | 2.48 |
| YBL042C | 0.389 | -1.36304 | 1.21E-17 | 4.35E-16 | yes | down | 26.39 | 28.1 | 25.71 | 10.89 | 9.02 | 9.95 |
| YBL057C | 0.458 | -1.1252 | 5.84E-13 | 1.29E-11 | yes | down | 162.95 | 157.95 | 178.22 | 75.43 | 80.77 | 61.96 |
| YBL080C | 0.474 | -1.07725 | 0.000713 | 0.002725 | yes | down | 5.67 | 5.86 | 5.65 | 2.99 | 1.33 | 3.06 |
| YBL082C | 0.385 | -1.37853 | 2.94E-05 | 0.000162 | yes | down | 71.19 | 75.51 | 94.54 | 39.16 | 30.93 | 12.78 |
| YBL083C | 0.441 | -1.17992 | 0.001285 | 0.004599 | yes | down | 50.94 | 55.86 | 46.55 | 33.71 | 11.46 | 13.36 |
| YBL100W-A | 0.459 | -1.12394 | 3.92E-05 | 0.000208 | yes | down | 6.08 | 8.08 | 7.68 | 2.39 | 3.12 | 3.76 |
| YBL111C | 0.476 | -1.07098 | 0.018338 | 0.045154 | yes | down | 2.56 | 8.41 | 3.84 | 0.74 | 2.9 | 2.15 |
| YBR003W | 0.497 | -1.0076 | 6.4E-06 | 4.07E-05 | yes | down | 52.63 | 46.08 | 43.86 | 23.6 | 28.01 | 15.85 |
| YBR008C | 0.38 | -1.39532 | 2.77E-22 | 1.6E-20 | yes | down | 66.42 | 61.02 | 65.44 | 25.25 | 24.95 | 20.3 |
| YBR012W-A | 0.437 | -1.19566 | 7.3E-07 | 5.81E-06 | yes | down | 56.81 | 69.48 | 62.29 | 19.77 | 35.29 | 22.27 |
| YBR026C | 0.261 | -1.93924 | 1.16E-44 | 3.34E-42 | yes | down | 282.51 | 269.52 | 284.04 | 63.79 | 69.11 | 74.69 |
| YBR029C | 0.4 | -1.32098 | 2.29E-08 | 2.44E-07 | yes | down | 34.11 | 31.89 | 32.56 | 9.89 | 10.56 | 16.19 |
| YBR030W | 0.476 | -1.07076 | 0.001573 | 0.005466 | yes | down | 12.96 | 10.91 | 13.97 | 3.85 | 3.56 | 8.66 |
| YBR031W | 0.355 | -1.49569 | 5.21E-11 | 8.8E-10 | yes | down | 2227.8 | 2426.82 | 2636.06 | 1002.44 | 870.85 | 545.06 |
| YBR037C | 0.453 | -1.14227 | 0.00035 | 0.001459 | yes | down | 326.25 | 309.37 | 292.79 | 170.23 | 153.92 | 58.97 |
| YBR038W | 0.277 | -1.85441 | 1.55E-12 | 3.2E-11 | yes | down | 5.88 | 3.99 | 6.5 | 1.4 | 1.57 | 1.14 |
| YBR089W | 0.348 | -1.5231 | 0.003166 | 0.010073 | yes | down | 5.33 | 4.63 | 14.12 | 1.67 | 3.36 | 0.3 |
| YBR110W | 0.413 | -1.27622 | 1.39E-14 | 3.72E-13 | yes | down | 37.46 | 42.34 | 48.15 | 17.28 | 17.14 | 16.08 |
| YBR116C | 0.062 | -4.01843 | 4.8E-84 | 7.61E-81 | yes | down | 267.04 | 240.42 | 262.62 | 14.41 | 10.43 | 16.34 |
| YBR117C | 0.036 | -4.7993 | 0 | 0 | yes | down | 764.61 | 754.78 | 745.69 | 26.39 | 26.82 | 23.57 |
| YBR125C | 0.495 | -1.01395 | 1.03E-10 | 1.68E-09 | yes | down | 95.02 | 100.76 | 96.06 | 46.17 | 53.48 | 39.45 |
| YBR126C | 0.443 | -1.17506 | 3.48E-16 | 1.09E-14 | yes | down | 2167.1 | 2091.55 | 2020.25 | 818.74 | 870.17 | 980.71 |
| YBR138C | 0.438 | -1.19026 | 7.12E-05 | 0.000352 | yes | down | 4.07 | 5.8 | 5.05 | 1.76 | 1.59 | 2.6 |
| YBR157C | 0.325 | -1.62066 | 9.22E-06 | 5.72E-05 | yes | down | 170.17 | 187.14 | 193.65 | 71.85 | 61.8 | 18.45 |
| YBR208C | 0.145 | -2.7822 | 4.71E-38 | 8.79E-36 | yes | down | 8.73 | 9.43 | 12.84 | 1.5 | 1.41 | 1.19 |
| YBR214W | 0.431 | -1.21526 | 8.83E-26 | 7.09E-24 | yes | down | 1169.44 | 1157.93 | 1084.52 | 460.89 | 503.08 | 456.72 |
| YBR218C | 0.439 | -1.18932 | 5.01E-22 | 2.77E-20 | yes | down | 185.84 | 198.03 | 191.32 | 88.12 | 82.11 | 74.09 |
| YBR219C | 0.491 | -1.02687 | 0.004897 | 0.014648 | yes | down | 41.93 | 19.5 | 37.91 | 19.52 | 10.65 | 11.76 |
| YBR220C | 0.475 | -1.07327 | 1.35E-05 | 7.92E-05 | yes | down | 56.12 | 59.89 | 56.87 | 31.95 | 29.51 | 16.16 |
| YBR222C | 0.488 | -1.0347 | 4.62E-20 | 2.06E-18 | yes | down | 408.12 | 415.59 | 446.51 | 208.34 | 198.44 | 193.45 |
| YBR232C | 0.4 | -1.32041 | 0.009577 | 0.025933 | yes | down | 6.01 | 10.6 | 7.31 | 2.4 | 1.96 | 2.2 |
| YBR234C | 0.447 | -1.16261 | 1.57E-25 | 1.24E-23 | yes | down | 338.19 | 320.02 | 345.32 | 141.97 | 147.1 | 143.59 |
| YBR241C | 0.499 | -1.00243 | 8.67E-09 | 1.01E-07 | yes | down | 1077.79 | 1000.66 | 954.75 | 514.52 | 559.86 | 381.09 |
| YBR246W | 0.489 | -1.03074 | 6.85E-14 | 1.67E-12 | yes | down | 64.01 | 60.26 | 68.12 | 29.06 | 31.31 | 30.24 |
| YBR285W | 0.132 | -2.92229 | 4.95E-49 | 2.09E-46 | yes | down | 373.36 | 437.44 | 411.87 | 56.59 | 51.43 | 37.14 |
| YBR294W | 0.421 | -1.24841 | 1.65E-11 | 2.96E-10 | yes | down | 23.77 | 25.19 | 27.82 | 12.54 | 9.58 | 8.75 |
| YBR296C | 0.297 | -1.75129 | 2.02E-11 | 3.57E-10 | yes | down | 16.16 | 25.51 | 14.58 | 4.84 | 5.15 | 5.24 |
| YCL020W | 0.406 | -1.29937 | 0.02449 | 0.057021 | yes | down | 0.17 | 2.2 | 2.29 | 0.66 | 0 | 0.13 |
| YCL036W | 0.249 | -2.00371 | 8.58E-14 | 2.06E-12 | yes | down | 14.1 | 14.53 | 24 | 4.63 | 3.51 | 3.61 |
| YCL057W | 0.385 | -1.37796 | 1.31E-16 | 4.32E-15 | yes | down | 74.36 | 77.53 | 79.98 | 26.84 | 25.56 | 32.64 |
| YCR004C | 0.409 | -1.29138 | 1.14E-19 | 4.81E-18 | yes | down | 727.89 | 724.16 | 670.62 | 273.01 | 313.57 | 243.4 |
| YCR010C | 0.26 | -1.94293 | 4.04E-10 | 5.96E-09 | yes | down | 3513.53 | 3624.91 | 2656.78 | 635.34 | 1159.46 | 440.16 |
| YCR017C | 0.435 | -1.19996 | 2.22E-09 | 2.89E-08 | yes | down | 55.63 | 60.33 | 64.71 | 27 | 29.68 | 18.37 |
| YCR034W | 0.344 | -1.53901 | 3.22E-09 | 4.1E-08 | yes | down | 20.9 | 20.94 | 30.69 | 6.83 | 6.49 | 9.4 |
| YCR044C | 0.462 | -1.11417 | 3.67E-10 | 5.48E-09 | yes | down | 80.76 | 71.9 | 75.65 | 31.74 | 40.82 | 28.27 |
| YCR061W | 0.498 | -1.00702 | 7.89E-05 | 0.000385 | yes | down | 164.66 | 160.73 | 158.71 | 85.87 | 96.04 | 45.68 |
| YCR065W | 0.361 | -1.47169 | 0.000445 | 0.001795 | yes | down | 37.4 | 32.52 | 30.65 | 10.45 | 16.52 | 2.65 |
| YCR067C | 0.258 | -1.95605 | 1.26E-23 | 8.14E-22 | yes | down | 9.57 | 10.01 | 11.21 | 2.93 | 2.51 | 2.02 |
| YCR075C | 0.217 | -2.20673 | 1.29E-34 | 2.05E-32 | yes | down | 129.76 | 118.01 | 135.16 | 26.02 | 21.89 | 29.18 |
| YCR079W | 0.404 | -1.30849 | 0.00043 | 0.001744 | yes | down | 431.11 | 420.37 | 402.89 | 188.26 | 202.51 | 50.94 |
| YCR088W | 0.427 | -1.22819 | 1.47E-27 | 1.34E-25 | yes | down | 228.86 | 217.76 | 213.42 | 94.64 | 92.21 | 85.77 |
| YCR105W | 0.473 | -1.08018 | 1.14E-05 | 6.88E-05 | yes | down | 47.17 | 52.91 | 45.5 | 26.35 | 24.66 | 13.89 |
| YDL021W | 0.485 | -1.04335 | 1.42E-10 | 2.28E-09 | yes | down | 39.22 | 37.91 | 34.06 | 17.41 | 17.6 | 16.64 |
| YDL022W | 0.214 | -2.22562 | 1.32E-09 | 1.79E-08 | yes | down | 1394.69 | 1383.1 | 1277.69 | 306.49 | 309.68 | 82.77 |
| YDL023C | 0.192 | -2.38083 | 2.92E-10 | 4.43E-09 | yes | down | 1652.84 | 1698.9 | 1560.65 | 293.4 | 338.25 | 77.57 |
| YDL042C | 0.284 | -1.81648 | 4.69E-20 | 2.08E-18 | yes | down | 22.89 | 22.71 | 24.37 | 7.01 | 7.02 | 4.65 |
| YDL049C | 0.411 | -1.28424 | 4.12E-08 | 4.1E-07 | yes | down | 23.47 | 22.18 | 29.32 | 10.97 | 10.47 | 7.32 |
| YDL085W | 0.148 | -2.75174 | 3.05E-37 | 5.53E-35 | yes | down | 653.8 | 649.46 | 556.82 | 81.62 | 108.12 | 61.98 |
| YDL103C | 0.4 | -1.32312 | 1.07E-08 | 1.22E-07 | yes | down | 29.4 | 29.75 | 29.76 | 13.27 | 12.77 | 7.39 |
| YDL142C | 0.397 | -1.33306 | 4.19E-14 | 1.05E-12 | yes | down | 159.21 | 147.87 | 145.5 | 48.35 | 56.33 | 65.24 |
| YDL144C | 0.378 | -1.40358 | 1.6E-26 | 1.4E-24 | yes | down | 389.77 | 385.84 | 399.28 | 150.65 | 152.9 | 123.5 |
| YDL145C | 0.378 | -1.4036 | 3.43E-17 | 1.18E-15 | yes | down | 10.62 | 10.41 | 11.28 | 3.88 | 4.47 | 3.35 |
| YDL164C | 0.379 | -1.40084 | 6.58E-11 | 1.1E-09 | yes | down | 8.45 | 6.91 | 9.28 | 3.37 | 2.89 | 2.53 |
| YDL171C | 0.492 | -1.02241 | 6.56E-16 | 1.97E-14 | yes | down | 561.45 | 863.18 | 754.89 | 1012.47 | 986.98 | 799.63 |
| YDL204W | 0.053 | -4.23909 | 4.3E-173 | 1.4E-169 | yes | down | 404.34 | 383.08 | 406.08 | 16.86 | 21.17 | 20.27 |
| YDL218W | 0.094 | -3.40491 | 2.75E-49 | 1.25E-46 | yes | down | 89.98 | 78.28 | 122.15 | 9.26 | 7.9 | 6.89 |
| YDL222C | 0.091 | -3.4649 | 2.22E-47 | 8.81E-45 | yes | down | 4591.86 | 4576.66 | 4501.22 | 263.56 | 473 | 274.29 |
| YDL223C | 0.143 | -2.80761 | 2.78E-84 | 5.89E-81 | yes | down | 184.46 | 176.28 | 180.41 | 22.5 | 28.35 | 22.4 |
| YDL228C | 0.431 | -1.21435 | 2.92E-07 | 2.49E-06 | yes | down | 142.02 | 161.57 | 228.73 | 86.82 | 64.04 | 62.8 |
| YDL229W | 0.389 | -1.36082 | 1.28E-05 | 7.58E-05 | yes | down | 161.84 | 180.9 | 266.11 | 107.85 | 61.38 | 44.85 |
| YDL240W | 0.46 | -1.11928 | 0.001173 | 0.004253 | yes | down | 0.99 | 0.84 | 0.94 | 0.42 | 0.44 | 0.3 |
| YDL244W | 0.276 | -1.85578 | 3.01E-14 | 7.74E-13 | yes | down | 27.7 | 21.55 | 29.85 | 4.98 | 7.98 | 6.96 |
| YDR009W | 0.316 | -1.65978 | 1.21E-08 | 1.36E-07 | yes | down | 60.99 | 56.67 | 57.79 | 17.14 | 23.81 | 9.14 |
| YDR010C | 0.384 | -1.3817 | 3.96E-06 | 2.65E-05 | yes | down | 53.55 | 54.34 | 67.07 | 22.65 | 14.88 | 20.71 |
| YDR012W | 0.339 | -1.56097 | 4.38E-17 | 1.49E-15 | yes | down | 910.48 | 973.76 | 1148.01 | 393.03 | 288.53 | 289.93 |
| YDR032C | 0.455 | -1.13508 | 1.42E-13 | 3.34E-12 | yes | down | 1475.09 | 1481.26 | 1443.38 | 586.22 | 600.22 | 713.94 |
| YDR033W | 0.459 | -1.12262 | 8.79E-07 | 6.85E-06 | yes | down | 122.96 | 114.97 | 139.6 | 44.67 | 47.27 | 70.14 |
| YDR034C-A | 0.348 | -1.52419 | 9.19E-08 | 8.63E-07 | yes | down | 32.66 | 31.47 | 58.77 | 11.73 | 78.26 | 17.68 |
| YDR034W-B | 0.411 | -1.28104 | 0.004762 | 0.01429 | yes | down | 755.3 | 1416.23 | 1281.73 | 189.14 | 245.67 | 441.96 |
| YDR064W | 0.427 | -1.22811 | 1.2E-11 | 2.17E-10 | yes | down | 1052.16 | 1125.87 | 1396.53 | 493.65 | 428.81 | 503.42 |
| YDR070C | 0.304 | -1.72013 | 2.45E-21 | 1.25E-19 | yes | down | 1973.82 | 1658 | 1901.42 | 432.56 | 482.49 | 572.33 |
| YDR074W | 0.4 | -1.32041 | 1.72E-15 | 4.99E-14 | yes | down | 529.07 | 527.56 | 531.93 | 206.35 | 240.23 | 164.04 |
| YDR091C | 0.392 | -1.35243 | 7.39E-08 | 7.01E-07 | yes | down | 10.33 | 8.73 | 12.99 | 4.26 | 2.97 | 4.37 |
| YDR098C-A | 0.407 | -1.29824 | 1.87E-07 | 1.65E-06 | yes | down | 67.76 | 79.24 | 79.54 | 34.24 | 33.96 | 18.06 |
| YDR107C | 0.28 | -1.8362 | 1.73E-25 | 1.36E-23 | yes | down | 22.3 | 25.26 | 28.93 | 6.52 | 7.21 | 6.54 |
| YDR119W-A | 0.449 | -1.15499 | 0.000902 | 0.003367 | yes | down | 4722.78 | 6196.16 | 5226.83 | 2562.78 | 2430.95 | 816.38 |
| YDR133C | 0.429 | -1.21948 | 0.000348 | 0.001452 | yes | down | 7698.94 | 7626.71 | 6892.91 | 3614.3 | 3526.02 | 1122.05 |
| YDR134C | 0.437 | -1.19312 | 0.00049 | 0.001959 | yes | down | 25129.72 | 25788.6 | 23638.1 | 12166.52 | 12628.72 | 3880.9 |
| YDR135C | 0.336 | -1.57547 | 6.64E-26 | 5.55E-24 | yes | down | 25.48 | 25.13 | 22.86 | 7.85 | 8.85 | 6.98 |
| YDR146C | 0.479 | -1.06251 | 0.005603 | 0.016344 | yes | down | 1.31 | 1.78 | 1.65 | 1.06 | 0.54 | 0.41 |
| YDR210C-C | 0.34 | -1.55652 | 2.17E-12 | 4.37E-11 | yes | down | 66.58 | 77.26 | 77.58 | 25.32 | 28.83 | 16.45 |
| YDR210W-A | 0.481 | -1.05572 | 4.4E-07 | 3.64E-06 | yes | down | 16.85 | 21.57 | 20.13 | 10.58 | 8.85 | 7.43 |
| YDR226W | 0.443 | -1.17391 | 6.31E-13 | 1.38E-11 | yes | down | 481.71 | 427.24 | 577.97 | 216.87 | 219.98 | 189.97 |
| YDR233C | 0.394 | -1.34372 | 3.94E-19 | 1.54E-17 | yes | down | 601.15 | 607.36 | 670.31 | 263.31 | 246.09 | 199.11 |
| YDR256C | 0.314 | -1.66958 | 2.29E-05 | 0.000128 | yes | down | 1156.81 | 1212.04 | 1013.41 | 288.11 | 491.17 | 103.19 |
| YDR261C-C | 0.373 | -1.42402 | 3.73E-05 | 0.000199 | yes | down | 37.29 | 50.8 | 51.37 | 19.11 | 20.19 | 6.55 |
| YDR284C | 0.431 | -1.21484 | 2.38E-11 | 4.17E-10 | yes | down | 107.98 | 93.11 | 108.82 | 34.99 | 44.85 | 46.59 |
| YDR316W-A | 0.429 | -1.22028 | 8.45E-18 | 3.1E-16 | yes | down | 74.78 | 74.69 | 83.15 | 32.36 | 35.02 | 28.68 |
| YDR365W-A | 0.383 | -1.38358 | 1.28E-20 | 5.95E-19 | yes | down | 59.14 | 61.41 | 54.42 | 21.9 | 20.28 | 22.1 |
| YDR380W | 0.437 | -1.19338 | 2.17E-06 | 1.54E-05 | yes | down | 27.24 | 33.09 | 42.69 | 11.09 | 13.25 | 17.53 |
| YDR384C | 0.149 | -2.74562 | 7.4E-10 | 1.04E-08 | yes | down | 926.9 | 927.26 | 742.69 | 64.26 | 162.86 | 20.63 |
| YDR385W | 0.37 | -1.43496 | 3.27E-19 | 1.29E-17 | yes | down | 1057.54 | 1050.61 | 1243.62 | 392.28 | 363.16 | 399.3 |
| YDR387C | 0.498 | -1.00468 | 3.28E-05 | 0.000178 | yes | down | 106.84 | 100.82 | 103.2 | 57.92 | 58.16 | 30.76 |
| YDR451C | 0.499 | -1.0015 | 7.24E-08 | 6.9E-07 | yes | down | 53.65 | 60.51 | 69.44 | 26.54 | 28.15 | 32.61 |
| YDR470C | 0.448 | -1.16002 | 1.95E-08 | 2.11E-07 | yes | down | 27.22 | 26.15 | 25.03 | 11.72 | 13.47 | 8.22 |
| YDR488C | 0.332 | -1.58941 | 2.29E-07 | 1.98E-06 | yes | down | 10.09 | 12.29 | 10.05 | 2.54 | 2.26 | 4.72 |
| YDR498C | 0.423 | -1.24083 | 4.11E-21 | 2.04E-19 | yes | down | 190.71 | 174.4 | 168.13 | 70.31 | 72.03 | 74.51 |
| YDR508C | 0.476 | -1.07139 | 0.001184 | 0.004284 | yes | down | 495.81 | 516.3 | 403.09 | 260.01 | 264.2 | 91.79 |
| YDR509W | 0.49 | -1.03028 | 0.003881 | 0.011992 | yes | down | 304.55 | 324.27 | 220.95 | 148.97 | 165.04 | 49.66 |
| YDR513W | 0.499 | -1.00234 | 9.72E-09 | 1.12E-07 | yes | down | 1509.6 | 1408.8 | 1444.17 | 761.74 | 763.82 | 523.01 |
| YDR534C | 0.308 | -1.70071 | 8.2E-09 | 9.6E-08 | yes | down | 25.04 | 23.56 | 23.42 | 7.13 | 9.23 | 3.53 |
| YDR536W | 0.287 | -1.8024 | 2.01E-20 | 9.26E-19 | yes | down | 112.73 | 107.9 | 121.81 | 35.15 | 34.65 | 22.7 |
| YEL013W | 0.47 | -1.08898 | 1.04E-09 | 1.43E-08 | yes | down | 31.18 | 32.69 | 27.97 | 12.87 | 16.37 | 12.16 |
| YEL020C | 0.354 | -1.49949 | 6.25E-11 | 1.05E-09 | yes | down | 131.92 | 118.97 | 140.53 | 49.61 | 51.39 | 28.66 |
| YEL033W | 0.405 | -1.30334 | 0.012576 | 0.032846 | yes | down | 5.18 | 10.93 | 12.75 | 0.8 | 1.58 | 5.17 |
| YEL034C-A | 0.235 | -2.09173 | 2.71E-20 | 1.23E-18 | yes | down | 1808.53 | 1853.51 | 1950.88 | 466.29 | 472.25 | 264.24 |
| YEL035C | 0.487 | -1.03693 | 5.76E-07 | 4.66E-06 | yes | down | 47.6 | 52.8 | 61.75 | 25.18 | 24.57 | 24.27 |
| YEL053W-A | 0.476 | -1.07056 | 7.38E-08 | 7.01E-07 | yes | down | 527.94 | 540.09 | 685.11 | 282.27 | 219.67 | 264.46 |
| YEL063C | 0.459 | -1.12446 | 8.19E-11 | 1.36E-09 | yes | down | 278.67 | 263.23 | 280.32 | 138.99 | 128.95 | 94.16 |
| YER015W | 0.315 | -1.66719 | 1.95E-13 | 4.46E-12 | yes | down | 108.47 | 101.51 | 92.83 | 32.66 | 36.38 | 20.05 |
| YER019W | 0.441 | -1.18214 | 5.38E-10 | 7.74E-09 | yes | down | 133.08 | 132.45 | 125.17 | 62.42 | 61.29 | 40.77 |
| YER023W | 0.28 | -1.83576 | 1.38E-46 | 4.86E-44 | yes | down | 325.03 | 306.71 | 346.77 | 92.92 | 88.91 | 80.15 |
| YER026C | 0.301 | -1.73066 | 2.83E-38 | 5.62E-36 | yes | down | 1051.39 | 1017.45 | 1076.08 | 272.15 | 318.44 | 312.74 |
| YER031C | 0.356 | -1.48867 | 3.16E-16 | 9.99E-15 | yes | down | 74.53 | 56.25 | 65.1 | 22.79 | 22.18 | 20.67 |
| YER043C | 0.414 | -1.27151 | 6.04E-08 | 5.86E-07 | yes | down | 138.49 | 117.6 | 176.75 | 70.06 | 56.66 | 41.5 |
| YER044C | 0.494 | -1.01866 | 1.94E-08 | 2.09E-07 | yes | down | 182.27 | 154.22 | 178.93 | 84.52 | 67.58 | 85.6 |
| YER046W | 0.481 | -1.05529 | 3.45E-05 | 0.000186 | yes | down | 65.88 | 51.81 | 74.54 | 35.7 | 27.8 | 21.46 |
| YER065C | 0.274 | -1.86891 | 2.08E-05 | 0.000117 | yes | down | 9004.83 | 8705.26 | 8800.12 | 2114.65 | 2937.38 | 383.95 |
| YER066W | 0.286 | -1.80452 | 4.15E-06 | 2.76E-05 | yes | down | 613.06 | 598.06 | 594.27 | 159.35 | 219.75 | 54.46 |
| YER070W | 0.448 | -1.15994 | 1.65E-06 | 1.21E-05 | yes | down | 6.57 | 5.56 | 6.18 | 3.24 | 2.69 | 1.8 |
| YER082C | 0.42 | -1.25089 | 5.71E-07 | 4.64E-06 | yes | down | 54.55 | 50.74 | 49.75 | 22.36 | 26.02 | 12.68 |
| YER087W | 0.463 | -1.10991 | 0.000775 | 0.002947 | yes | down | 3 | 2.64 | 1.97 | 1.01 | 0.89 | 1.3 |
| YER089C | 0.493 | -1.02041 | 1.34E-05 | 7.89E-05 | yes | down | 114.14 | 111.53 | 131.59 | 65.21 | 65.27 | 36.47 |
| YER091C | 0.183 | -2.45233 | 2.38E-28 | 2.29E-26 | yes | down | 175.22 | 169.16 | 278.24 | 34.7 | 33.54 | 35.28 |
| YER096W | 0.355 | -1.49504 | 1.1E-12 | 2.35E-11 | yes | down | 94.22 | 91.97 | 78.33 | 24.33 | 27.41 | 36.12 |
| YER113C | 0.475 | -1.074 | 0.001555 | 0.005412 | yes | down | 9.39 | 8.94 | 9.07 | 5.24 | 4.89 | 1.71 |
| YER119C-A | 0.46 | -1.11873 | 0.001246 | 0.004477 | yes | down | 34.23 | 18.89 | 28.33 | 8.75 | 11.32 | 12.04 |
| YER121W | 0.223 | -2.16797 | 5.83E-16 | 1.78E-14 | yes | down | 167.61 | 202.44 | 144.64 | 36.61 | 40.09 | 22.34 |
| YER135C | 0.433 | -1.20698 | 0.00035 | 0.00146 | yes | down | 24.16 | 26.63 | 25.85 | 11.35 | 12.12 | 5.65 |
| YER137C-A | 0.385 | -1.37536 | 1.51E-09 | 2.03E-08 | yes | down | 54.16 | 60.06 | 64.54 | 27.8 | 21.63 | 15.31 |
| YER152W-A | 0.375 | -1.41399 | 2.21E-14 | 5.82E-13 | yes | down | 153.08 | 127.38 | 152.36 | 56.58 | 55.78 | 40.28 |
| YER159C-A | 0.381 | -1.39273 | 1.24E-18 | 4.8E-17 | yes | down | 69.03 | 81.06 | 73.55 | 27.16 | 30.32 | 24.1 |
| YER181C | 0.482 | -1.05275 | 0.010666 | 0.028588 | yes | down | 31.47 | 23.27 | 23.85 | 4.94 | 11.36 | 14.29 |
| YER189W | 0.309 | -1.69611 | 2.04E-05 | 0.000115 | yes | down | 19.25 | 36.34 | 27.27 | 9.36 | 5.69 | 5.03 |
| YFL037W | 0.462 | -1.11489 | 4.81E-16 | 1.49E-14 | yes | down | 59.3 | 58.87 | 64.7 | 26.25 | 26.91 | 28.06 |
| YFL055W | 0.255 | -1.97121 | 1.08E-09 | 1.48E-08 | yes | down | 227.75 | 216.24 | 215.18 | 44.41 | 75.18 | 28.35 |
| YFL064C | 0.427 | -1.22753 | 0.028999 | 0.065595 | yes | down | 2.46 | 2.42 | 4.46 | 1.69 | 0.58 | 0 |
| YFR014C | 0.421 | -1.24793 | 0.000262 | 0.001132 | yes | down | 58.24 | 57.3 | 52.09 | 25.92 | 28.53 | 8.78 |
| YFR030W | 0.323 | -1.63182 | 3.42E-30 | 4.09E-28 | yes | down | 79 | 85.23 | 98.08 | 27.94 | 27.98 | 25.31 |
| YFR044C | 0.316 | -1.66398 | 5.17E-47 | 1.93E-44 | yes | down | 431.49 | 428.35 | 434.96 | 136.9 | 124.86 | 131.5 |
| YGL017W | 0.465 | -1.10315 | 3.09E-10 | 4.64E-09 | yes | down | 39.47 | 35.72 | 40.52 | 15.71 | 15.96 | 19.68 |
| YGL021W | 0.49 | -1.02887 | 4.15E-06 | 2.76E-05 | yes | down | 9.12 | 9.39 | 11.9 | 4.49 | 5.87 | 3.8 |
| YGL034C | 0.412 | -1.27852 | 0.017861 | 0.044196 | yes | down | 7.52 | 8.32 | 3.98 | 1.72 | 0.56 | 2.62 |
| YGL055W | 0.471 | -1.08643 | 4.07E-08 | 4.06E-07 | yes | down | 637.34 | 629.13 | 794.93 | 469.92 | 443.62 | 451.61 |
| YGL080W | 0.48 | -1.05845 | 1.11E-08 | 1.26E-07 | yes | down | 394.21 | 388.35 | 363.76 | 188.25 | 194.92 | 130.39 |
| YGL084C | 0.302 | -1.72561 | 5.06E-29 | 5.27E-27 | yes | down | 44.65 | 42.66 | 41.95 | 11.11 | 12.92 | 13.19 |
| YGL104C | 0.26 | -1.94169 | 2.35E-17 | 8.27E-16 | yes | down | 80.68 | 82.54 | 79.48 | 16.83 | 15.57 | 25.42 |
| YGL105W | 0.344 | -1.54114 | 1.53E-16 | 4.97E-15 | yes | down | 132.95 | 116.88 | 158.86 | 39.19 | 43.95 | 49.11 |
| YGL119W | 0.282 | -1.82716 | 1.51E-20 | 7.01E-19 | yes | down | 21.59 | 19.45 | 25.85 | 6.12 | 6.43 | 5.14 |
| YGL123W | 0.362 | -1.46558 | 8.64E-10 | 1.2E-08 | yes | down | 703.37 | 736.63 | 1007.6 | 258 | 216.9 | 340.18 |
| YGL137W | 0.497 | -1.00911 | 1.71E-10 | 2.71E-09 | yes | down | 19.83 | 19.9 | 21.06 | 10.01 | 10.93 | 8.21 |
| YGL147C | 0.387 | -1.3709 | 4.87E-09 | 6.02E-08 | yes | down | 150.65 | 140.59 | 154.79 | 62.38 | 63.03 | 34.72 |
| YGL158W | 0.176 | -2.50464 | 2.64E-09 | 3.4E-08 | yes | down | 84.89 | 112.12 | 114.9 | 21.13 | 14.57 | 3.37 |
| YGL161C | 0.395 | -1.33903 | 6.53E-29 | 6.68E-27 | yes | down | 258.52 | 252.51 | 236.96 | 94.08 | 97.41 | 92.83 |
| YGL162W | 0.339 | -1.56124 | 0.000232 | 0.001013 | yes | down | 169.85 | 223.44 | 333.32 | 92.73 | 85.87 | 17.96 |
| YGL199C | 0.418 | -1.25926 | 9.26E-08 | 8.67E-07 | yes | down | 522.79 | 577.39 | 445.57 | 214.87 | 247.48 | 135.65 |
| YGL202W | 0.428 | -1.22424 | 1.27E-22 | 7.47E-21 | yes | down | 713.7 | 724.41 | 663.98 | 288.67 | 278.95 | 299.72 |
| YGL205W | 0.171 | -2.54735 | 1.78E-43 | 4.92E-41 | yes | down | 35.48 | 37.99 | 29.89 | 5.05 | 6.25 | 5.18 |
| YGL213C | 0.469 | -1.09152 | 6.91E-08 | 6.62E-07 | yes | down | 37.26 | 26.19 | 26.99 | 14.17 | 13.59 | 12.59 |
| YGL234W | 0.499 | -1.00158 | 0.012523 | 0.032736 | yes | down | 107.13 | 129.69 | 163.14 | 76.71 | 81.57 | 17.61 |
| YGR019W | 0.295 | -1.75899 | 1.59E-36 | 2.66E-34 | yes | down | 588.06 | 529.26 | 655.12 | 172.03 | 176.75 | 152.35 |
| YGR027W-A | 0.431 | -1.21263 | 2.46E-14 | 6.41E-13 | yes | down | 93.44 | 114.62 | 111.7 | 48.46 | 43.69 | 40.25 |
| YGR031W | 0.442 | -1.17759 | 3.05E-09 | 3.9E-08 | yes | down | 466.67 | 489.51 | 449.36 | 212.09 | 235.3 | 143.99 |
| YGR036C | 0.294 | -1.76418 | 5.53E-14 | 1.36E-12 | yes | down | 23.84 | 20.62 | 25.1 | 6.43 | 7.22 | 5.15 |
| YGR038C-A | 0.327 | -1.61387 | 7.05E-21 | 3.36E-19 | yes | down | 52.69 | 61 | 49.62 | 16.91 | 15.77 | 18 |
| YGR052W | 0.435 | -1.20007 | 0.000515 | 0.002044 | yes | down | 196.79 | 203.92 | 261.12 | 104.23 | 117.74 | 35.92 |
| YGR055W | 0.27 | -1.88742 | 2.41E-12 | 4.82E-11 | yes | down | 279.61 | 284.67 | 351.75 | 94.06 | 86.25 | 43.78 |
| YGR060W | 0.43 | -1.216 | 8.98E-10 | 1.24E-08 | yes | down | 32.06 | 32.22 | 39.24 | 16.66 | 12.97 | 12.57 |
| YGR065C | 0.163 | -2.62118 | 8.2E-14 | 1.98E-12 | yes | down | 252.86 | 318.7 | 275.8 | 43.4 | 51.97 | 14.72 |
| YGR067C | 0.39 | -1.36021 | 0.012668 | 0.033033 | yes | down | 264.14 | 245.93 | 238.52 | 56.69 | 110.32 | 10.86 |
| YGR069W | 0.455 | -1.13669 | 0.04749 | 0.098504 | yes | down | 4.52 | 8.7 | 5.86 | 0.73 | 3.62 | 0 |
| YGR077C | 0.357 | -1.48612 | 8.71E-21 | 4.1E-19 | yes | down | 72.16 | 61.87 | 68.55 | 22.07 | 26.72 | 20.38 |
| YGR087C | 0.269 | -1.89264 | 1.23E-21 | 6.48E-20 | yes | down | 34.1 | 30.61 | 43.52 | 8.23 | 10.06 | 8.95 |
| YGR088W | 0.075 | -3.72999 | 1.69E-56 | 1.19E-53 | yes | down | 998.24 | 849.67 | 1032.35 | 69.33 | 78.37 | 41.32 |
| YGR113W | 0.485 | -1.04413 | 3.09E-05 | 0.000169 | yes | down | 20.72 | 17.77 | 15.41 | 8.69 | 6.07 | 9.63 |
| YGR121C | 0.351 | -1.50982 | 2.93E-16 | 9.31E-15 | yes | down | 433.15 | 529.24 | 452.54 | 168.12 | 179.94 | 124.44 |
| YGR122W | 0.287 | -1.79994 | 1.33E-28 | 1.32E-26 | yes | down | 52.07 | 53.88 | 54.89 | 13.45 | 16.97 | 13.45 |
| YGR131W | 0.483 | -1.04952 | 3.06E-12 | 6.03E-11 | yes | down | 211.46 | 235.43 | 197.33 | 98.3 | 103.83 | 95.63 |
| YGR144W | 0.386 | -1.37324 | 5.01E-10 | 7.27E-09 | yes | down | 40.89 | 37.27 | 47.7 | 16.15 | 11.69 | 17.45 |
| YGR154C | 0.454 | -1.14007 | 1.51E-06 | 1.12E-05 | yes | down | 33.89 | 29.4 | 36.52 | 12.54 | 11.48 | 18.19 |
| YGR161C-C | 0.447 | -1.16201 | 6E-13 | 1.32E-11 | yes | down | 64.35 | 76.71 | 81.26 | 34.34 | 31.3 | 29.65 |
| YGR190C | 0.376 | -1.4122 | 0.000228 | 0.001 | yes | down | 58.98 | 38.88 | 74.03 | 27.45 | 17.29 | 8.41 |
| YGR194C | 0.291 | -1.78175 | 6.99E-15 | 1.92E-13 | yes | down | 100.46 | 96.6 | 82.55 | 29.25 | 29.34 | 17.03 |
| YGR241C | 0.475 | -1.07519 | 3.8E-08 | 3.82E-07 | yes | down | 103.46 | 103.67 | 82.07 | 49.44 | 47.59 | 34.24 |
| YGR242W | 0.349 | -1.51737 | 1.72E-10 | 2.73E-09 | yes | down | 163.07 | 129.67 | 138.45 | 41.62 | 54.57 | 36.87 |
| YGR244C | 0.314 | -1.6704 | 6.06E-12 | 1.14E-10 | yes | down | 314.93 | 293.06 | 323.49 | 95.24 | 117.66 | 57.85 |
| YGR248W | 0.361 | -1.46945 | 3.97E-25 | 2.86E-23 | yes | down | 1650.9 | 1531.25 | 1343.38 | 505.84 | 516.78 | 534.94 |
| YGR256W | 0.282 | -1.82563 | 2.11E-19 | 8.71E-18 | yes | down | 96.26 | 94.09 | 101.98 | 22.56 | 22.24 | 31.95 |
| YGR259C | 0.401 | -1.31986 | 0.014695 | 0.037489 | yes | down | 139.05 | 185.07 | 113.73 | 3.58 | 0 | 0 |
| YGR260W | 0.356 | -1.48987 | 2.34E-05 | 0.000131 | yes | down | 462.43 | 480.56 | 444.54 | 181.9 | 193.74 | 55.81 |
| YGR266W | 0.308 | -1.70075 | 6.39E-27 | 5.72E-25 | yes | down | 37.18 | 33.4 | 31.83 | 10.54 | 10.82 | 8.74 |
| YGR279C | 0.436 | -1.19868 | 3.59E-09 | 4.52E-08 | yes | down | 58.93 | 66.73 | 71.76 | 34.28 | 24.57 | 22.77 |
| YGR286C | 0.12 | -3.06471 | 2.06E-41 | 4.66E-39 | yes | down | 443.32 | 493.37 | 443.6 | 44.09 | 66.66 | 36.71 |
| YHL016C | 0.289 | -1.78841 | 1.88E-22 | 1.09E-20 | yes | down | 25.47 | 22.32 | 31.24 | 7.22 | 7.31 | 7.05 |
| YHL032C | 0.281 | -1.83092 | 4.05E-22 | 2.27E-20 | yes | down | 1702.81 | 1703.62 | 1729.98 | 516.95 | 511.5 | 335.45 |
| YHL033C | 0.431 | -1.21521 | 2.26E-08 | 2.41E-07 | yes | down | 265.77 | 301.55 | 438.32 | 157.73 | 130.64 | 141.34 |
| YHL040C | 0.394 | -1.34317 | 3.27E-09 | 4.15E-08 | yes | down | 174.3 | 191.05 | 203.04 | 84.65 | 79.31 | 47.19 |
| YHL046C | 0.481 | -1.05533 | 0.001809 | 0.006165 | yes | down | 31 | 40.7 | 30.93 | 20.48 | 12.22 | 10.95 |
| YHR007C | 0.449 | -1.15644 | 2.52E-07 | 2.16E-06 | yes | down | 43.31 | 42.84 | 62.84 | 22.33 | 17.37 | 23.16 |
| YHR020W | 0.45 | -1.15185 | 6.8E-12 | 1.27E-10 | yes | down | 292.59 | 320.12 | 361.04 | 157.31 | 147.69 | 115.99 |
| YHR033W | 0.341 | -1.55259 | 3.17E-06 | 2.15E-05 | yes | down | 1950.98 | 1961.86 | 1743.05 | 765.45 | 687.07 | 244.55 |
| YHR034C | 0.459 | -1.12348 | 3.19E-05 | 0.000174 | yes | down | 103.72 | 89.15 | 100.12 | 53.51 | 47.69 | 24.14 |
| YHR039C | 0.404 | -1.30906 | 1.02E-22 | 6.14E-21 | yes | down | 48.72 | 44.77 | 50.23 | 18.85 | 19.8 | 17.25 |
| YHR071C-A | 0.483 | -1.05034 | 0.015364 | 0.038943 | yes | down | 21.07 | 43.32 | 38.15 | 22.06 | 8.36 | 10.07 |
| YHR074W | 0.456 | -1.13271 | 7.39E-10 | 1.04E-08 | yes | down | 120.32 | 106.04 | 117.4 | 55.14 | 56.97 | 38.13 |
| YHR086W | 0.485 | -1.04352 | 0.000231 | 0.00101 | yes | down | 100.25 | 95.11 | 90.34 | 51.28 | 54.79 | 23.38 |
| YHR095W | 0.41 | -1.28631 | 0.000682 | 0.00262 | yes | down | 517.29 | 545.91 | 498.57 | 154.25 | 63.17 | 225.72 |
| YHR096C | 0.275 | -1.86304 | 1.24E-27 | 1.14E-25 | yes | down | 2548.89 | 2503.29 | 2340.62 | 550.76 | 774.86 | 600.57 |
| YHR105W | 0.476 | -1.06977 | 4.94E-07 | 4.04E-06 | yes | down | 56.97 | 56.17 | 60.81 | 29.39 | 30.05 | 18.86 |
| YHR106W | 0.429 | -1.22105 | 8.89E-14 | 2.13E-12 | yes | down | 336.9 | 335.15 | 313.45 | 142.48 | 153.66 | 108.85 |
| YHR108W | 0.386 | -1.3725 | 1.07E-08 | 1.22E-07 | yes | down | 48.96 | 42.96 | 46 | 20.57 | 18.62 | 10.72 |
| YHR117W | 0.432 | -1.20926 | 8.28E-11 | 1.37E-09 | yes | down | 26.39 | 24.87 | 26.4 | 9.45 | 9.83 | 12.62 |
| YHR136C | 0.307 | -1.70447 | 8.94E-11 | 1.47E-09 | yes | down | 48.74 | 67.8 | 47.92 | 14.96 | 16.81 | 13.47 |
| YHR137W | 0.226 | -2.14622 | 2.02E-19 | 8.38E-18 | yes | down | 151.11 | 175.93 | 232 | 40.47 | 46.77 | 28.03 |
| YHR139C | 0.149 | -2.74924 | 4.03E-18 | 1.5E-16 | yes | down | 107.01 | 100.93 | 92.53 | 6.67 | 11.32 | 18.33 |
| YHR153C | 0.486 | -1.04178 | 0.018627 | 0.045556 | yes | down | 3.83 | 4.48 | 4.7 | 2.77 | 1.56 | 0.92 |
| YHR203C | 0.436 | -1.19688 | 4.04E-07 | 3.35E-06 | yes | down | 445.46 | 456.96 | 657.19 | 272.17 | 192.1 | 172.07 |
| YHR214C-C | 0.457 | -1.12889 | 3.17E-12 | 6.21E-11 | yes | down | 43.4 | 54.25 | 51.68 | 22.97 | 21.28 | 21.23 |
| YIL018W | 0.379 | -1.39947 | 7.1E-09 | 8.44E-08 | yes | down | 1055.74 | 1082.87 | 1730.92 | 448.01 | 387.09 | 517.09 |
| YIL039W | 0.414 | -1.27392 | 6.65E-20 | 2.89E-18 | yes | down | 37.2 | 35.92 | 36.3 | 14.46 | 15.28 | 13.78 |
| YIL042C | 0.377 | -1.40897 | 2.67E-06 | 1.85E-05 | yes | down | 253.92 | 256.4 | 241.29 | 101.08 | 112.93 | 43.14 |
| YIL057C | 0.327 | -1.61108 | 1.36E-08 | 1.52E-07 | yes | down | 877.11 | 964.78 | 749.48 | 247.25 | 362.06 | 150.17 |
| YIL082W-A | 0.46 | -1.12071 | 3.61E-08 | 3.65E-07 | yes | down | 217.19 | 219.76 | 198.34 | 175.96 | 188.65 | 174.62 |
| YIL099W | 0.257 | -1.95785 | 3.88E-24 | 2.65E-22 | yes | down | 144.19 | 174.7 | 130.66 | 36.07 | 42.48 | 30.2 |
| YIL101C | 0.278 | -1.8494 | 1.16E-26 | 1.02E-24 | yes | down | 1415.59 | 1435.95 | 1340.37 | 385.35 | 429.87 | 289.51 |
| YIL109C | 0.465 | -1.10537 | 0.00031 | 0.001315 | yes | down | 110.21 | 105.9 | 109.85 | 57.36 | 59.86 | 22.7 |
| YIL113W | 0.421 | -1.24957 | 9.73E-07 | 7.49E-06 | yes | down | 245.67 | 209.2 | 198.95 | 96.71 | 105.84 | 52.26 |
| YIL124W | 0.309 | -1.69365 | 5.2E-43 | 1.32E-40 | yes | down | 293.25 | 295.01 | 296.05 | 83.15 | 88.78 | 89.72 |
| YIL155C | 0.321 | -1.63778 | 2.9E-17 | 1.01E-15 | yes | down | 555.88 | 534.18 | 516.92 | 166.97 | 199.66 | 122.17 |
| YIL160C | 0.244 | -2.03481 | 2.06E-29 | 2.25E-27 | yes | down | 480.32 | 476.09 | 375.49 | 90.16 | 117.73 | 97.6 |
| YIL171W | 0.471 | -1.08759 | 0.021749 | 0.05168 | yes | down | 18.39 | 18.4 | 9.3 | 3.11 | 5.36 | 7.81 |
| YIR007W | 0.438 | -1.19204 | 8.38E-09 | 9.78E-08 | yes | down | 33.47 | 29.91 | 31.4 | 14.36 | 15.69 | 9.47 |
| YIR008C | 0.424 | -1.23922 | 4.79E-12 | 9.17E-11 | yes | down | 27.33 | 22.91 | 28.31 | 10.22 | 11.76 | 9.71 |
| YIR014W | 0.471 | -1.08753 | 0.011008 | 0.029307 | yes | down | 76.62 | 73.52 | 65.82 | 36.27 | 42.9 | 6.78 |
| YIR016W | 0.465 | -1.1062 | 0.002549 | 0.008296 | yes | down | 924.51 | 846.49 | 752.7 | 446.8 | 468.93 | 125.04 |
| YIR017C | 0.315 | -1.66513 | 1.03E-18 | 4.01E-17 | yes | down | 178.43 | 205.71 | 230.64 | 67.4 | 52.39 | 60.95 |
| YIR017W-A | 0.392 | -1.35043 | 0.000545 | 0.002152 | yes | down | 31.32 | 40.58 | 64.95 | 9.8 | 26.25 | 9.27 |
| YIR020C-B | 0.338 | -1.5662 | 8.91E-05 | 0.00043 | yes | down | 4.57 | 11.73 | 7.51 | 1.78 | 2.49 | 2.21 |
| YIR032C | 0.446 | -1.1643 | 1.22E-05 | 7.26E-05 | yes | down | 75.13 | 73.03 | 95.63 | 25.2 | 29.3 | 44.79 |
| YIR036W-A | 0.463 | -1.10977 | 5.57E-10 | 8E-09 | yes | down | 256.33 | 248.83 | 285.15 | 102.61 | 109.65 | 127.66 |
| YJL045W | 0.232 | -2.10554 | 8.69E-26 | 7.07E-24 | yes | down | 191.96 | 166.26 | 202.7 | 35.61 | 51.88 | 34.04 |
| YJL051W | 0.474 | -1.0761 | 4.92E-12 | 9.36E-11 | yes | down | 27.36 | 23.24 | 27.02 | 11.84 | 13.11 | 10.51 |
| YJL060W | 0.479 | -1.06293 | 0.000606 | 0.002372 | yes | down | 38.41 | 36.18 | 37.47 | 11.51 | 11.88 | 25.24 |
| YJL079C | 0.442 | -1.17733 | 2.89E-08 | 3.01E-07 | yes | down | 510.04 | 537.87 | 529.63 | 264.46 | 243 | 153.26 |
| YJL088W | 0.499 | -1.00286 | 2.96E-17 | 1.02E-15 | yes | down | 437.77 | 475.82 | 482.62 | 221.94 | 230.6 | 219.85 |
| YJL091C | 0.472 | -1.0835 | 1.11E-07 | 1.02E-06 | yes | down | 24.41 | 28.19 | 26.21 | 10.49 | 10.54 | 14.2 |
| YJL097W | 0.345 | -1.53705 | 1.48E-16 | 4.84E-15 | yes | down | 100.04 | 107.98 | 110.16 | 40.63 | 35.25 | 27.42 |
| YJL119C | 0.497 | -1.00737 | 0.029561 | 0.066674 | yes | down | 17.44 | 17.24 | 9.49 | 4.12 | 8.28 | 5.62 |
| YJL153C | 0.186 | -2.42674 | 8.86E-29 | 8.93E-27 | yes | down | 907.12 | 795.31 | 891.45 | 121.45 | 128.56 | 188.57 |
| YJL160C | 0.302 | -1.72683 | 7.24E-20 | 3.08E-18 | yes | down | 36.74 | 42.06 | 41.92 | 11.4 | 13.03 | 9.87 |
| YJL172W | 0.434 | -1.2051 | 2.8E-16 | 8.94E-15 | yes | down | 88.19 | 91.8 | 78.53 | 33.45 | 38.74 | 35.67 |
| YJL180C | 0.497 | -1.00826 | 3.32E-05 | 0.00018 | yes | down | 20.22 | 15.51 | 15.81 | 9.71 | 8.48 | 5.96 |
| YJL212C | 0.316 | -1.66094 | 3.77E-10 | 5.6E-09 | yes | down | 62.4 | 57.54 | 74.76 | 21.95 | 23.25 | 11.23 |
| YJL216C | 0.296 | -1.7545 | 6.84E-12 | 1.28E-10 | yes | down | 6.9 | 8.12 | 7.37 | 1.79 | 1.68 | 2.56 |
| YJL219W | 0.424 | -1.23795 | 2.17E-08 | 2.32E-07 | yes | down | 14.36 | 16.16 | 19.77 | 8.02 | 5.58 | 6.45 |
| YJR001W | 0.421 | -1.24824 | 9.03E-13 | 1.96E-11 | yes | down | 102.74 | 100.04 | 101.4 | 44.01 | 46.94 | 31.75 |
| YJR016C | 0.496 | -1.01283 | 2.44E-05 | 0.000136 | yes | down | 2133.04 | 2083.84 | 2452.35 | 1272.55 | 1165.8 | 657.78 |
| YJR019C | 0.23 | -2.11957 | 3.7E-38 | 7.12E-36 | yes | down | 528.95 | 536.77 | 468.85 | 113.08 | 128.27 | 92.55 |
| YJR020W | 0.247 | -2.02017 | 4.54E-20 | 2.05E-18 | yes | down | 739.18 | 702.8 | 584.7 | 176.69 | 164.37 | 103.31 |
| YJR024C | 0.289 | -1.78965 | 3.51E-29 | 3.71E-27 | yes | down | 205.76 | 199.01 | 207.57 | 58.33 | 63.63 | 45.85 |
| YJR026W | 0.422 | -1.24627 | 1.04E-08 | 1.19E-07 | yes | down | 30.05 | 41.14 | 40.66 | 16.67 | 16.28 | 11.6 |
| YJR028W | 0.369 | -1.43735 | 5.97E-16 | 1.81E-14 | yes | down | 73.32 | 98.03 | 91.87 | 33.13 | 31.84 | 27.56 |
| YJR038C | 0.348 | -1.52094 | 1.43E-05 | 8.35E-05 | yes | down | 32.02 | 40.45 | 30.3 | 8.78 | 14.42 | 6.98 |
| YJR039W | 0.41 | -1.28688 | 3.96E-13 | 8.85E-12 | yes | down | 19.7 | 19.37 | 16.39 | 6.65 | 8.48 | 6.65 |
| YJR040W | 0.36 | -1.47581 | 1.37E-07 | 1.24E-06 | yes | down | 7.32 | 4.89 | 4.46 | 1.77 | 2.24 | 1.47 |
| YJR044C | 0.473 | -1.07977 | 2.01E-10 | 3.15E-09 | yes | down | 391.46 | 416.86 | 398.27 | 160.26 | 172.44 | 200.59 |
| YJR094C | 0.208 | -2.26358 | 1.25E-14 | 3.37E-13 | yes | down | 74.31 | 65.02 | 65.57 | 12.36 | 17.99 | 6.82 |
| YJR095W | 0.379 | -1.39943 | 5.34E-08 | 5.21E-07 | yes | down | 365.14 | 299.83 | 282.98 | 105.4 | 153.37 | 73.82 |
| YJR116W | 0.345 | -1.53334 | 2.96E-08 | 3.07E-07 | yes | down | 768.13 | 947.42 | 849.55 | 344.87 | 342.4 | 130.73 |
| YJR123W | 0.382 | -1.3901 | 1.68E-11 | 3.01E-10 | yes | down | 924.93 | 958.84 | 1218.06 | 365.39 | 305.83 | 432.7 |
| YJR137C | 0.237 | -2.07406 | 1.21E-29 | 1.4E-27 | yes | down | 7.45 | 6.93 | 8.41 | 1.53 | 1.83 | 1.72 |
| YJR138W | 0.426 | -1.23133 | 4.49E-06 | 2.97E-05 | yes | down | 2.4 | 1.87 | 2.25 | 0.62 | 0.86 | 1.08 |
| YJR143C | 0.398 | -1.32785 | 5.74E-12 | 1.09E-10 | yes | down | 77.03 | 72.01 | 82.02 | 34.52 | 31.3 | 21.87 |
| YJR154W | 0.469 | -1.09105 | 1.68E-08 | 1.84E-07 | yes | down | 30.48 | 30.12 | 33.2 | 13.54 | 16.98 | 11.45 |
| YJR156C | 0.499 | -1.0023 | 8.67E-08 | 8.16E-07 | yes | down | 31.13 | 35.29 | 34.8 | 13.58 | 17.28 | 17.27 |
| YKL039W | 0.008 | -6.91766 | 3.26E-53 | 2.07E-50 | yes | down | 21.68 | 15.84 | 19.13 | 0 | 0 | 0 |
| YKL050C | 0.496 | -1.01192 | 0.000648 | 0.002511 | yes | down | 4.83 | 3.91 | 4.91 | 1.31 | 2.1 | 2.82 |
| YKL081W | 0.449 | -1.15498 | 6.1E-09 | 7.34E-08 | yes | down | 52.58 | 56.44 | 77.38 | 27.41 | 25.1 | 26.85 |
| YKL096W | 0.259 | -1.94836 | 0.000275 | 0.001185 | yes | down | 1014.81 | 889.43 | 1468.85 | 168.61 | 255.13 | 31.72 |
| YKL100C | 0.438 | -1.19263 | 1.2E-07 | 1.1E-06 | yes | down | 369.55 | 346.24 | 323.61 | 149.47 | 182.71 | 98.47 |
| YKL103C | 0.473 | -1.07922 | 9.05E-16 | 2.7E-14 | yes | down | 193.93 | 174.99 | 192.5 | 86.58 | 79.55 | 89.75 |
| YKL112W | 0.442 | -1.17728 | 4.94E-09 | 6.08E-08 | yes | down | 15.96 | 16.95 | 14.79 | 7.96 | 6.99 | 5.17 |
| YKL151C | 0.367 | -1.44612 | 8.04E-30 | 9.45E-28 | yes | down | 1003.42 | 977 | 984.11 | 332.87 | 338.47 | 371.61 |
| YKL163W | 0.449 | -1.15588 | 0.03198 | 0.071195 | yes | down | 1010.7 | 1038.18 | 1529.82 | 519.54 | 492.99 | 68 |
| YKL182W | 0.344 | -1.53964 | 2.03E-08 | 2.18E-07 | yes | down | 28.28 | 29.68 | 33.95 | 6.89 | 8.03 | 13.75 |
| YKL185W | 0.226 | -2.1462 | 5.22E-13 | 1.16E-11 | yes | down | 47.22 | 52.47 | 54.07 | 12.08 | 13.19 | 5.18 |
| YKL187C | 0.335 | -1.57943 | 5E-21 | 2.42E-19 | yes | down | 307.28 | 298.15 | 270.99 | 65.3 | 82.49 | 54.03 |
| YKL210W | 0.475 | -1.07355 | 1.13E-09 | 1.54E-08 | yes | down | 48.7 | 46.15 | 47.84 | 18.48 | 21.08 | 25.27 |
| YKR003W | 0.443 | -1.17483 | 2.6E-08 | 2.74E-07 | yes | down | 17.74 | 14.58 | 17.45 | 5.63 | 7.54 | 7.62 |
| YKR009C | 0.453 | -1.14083 | 1.67E-15 | 4.87E-14 | yes | down | 195.32 | 174.37 | 155.36 | 77.19 | 78.87 | 73.62 |
| YKR039W | 0.128 | -2.96827 | 4.1E-24 | 2.74E-22 | yes | down | 188.4 | 189.51 | 192.3 | 23.41 | 27.45 | 10.57 |
| YKR052C | 0.21 | -2.25349 | 3.96E-13 | 8.85E-12 | yes | down | 495.9 | 480.3 | 545.45 | 108.67 | 121.04 | 43.7 |
| YKR053C | 0.489 | -1.03213 | 4.96E-11 | 8.42E-10 | yes | down | 107.29 | 108.04 | 115.04 | 70.75 | 76.58 | 46.87 |
| YKR059W | 0.395 | -1.34099 | 2.19E-20 | 9.98E-19 | yes | down | 194.23 | 189.58 | 234.74 | 77.54 | 80.97 | 75.48 |
| YKR067W | 0.399 | -1.32674 | 4.96E-05 | 0.000256 | yes | down | 241.25 | 236.63 | 223.35 | 101.7 | 112.84 | 37.3 |
| YKR070W | 0.466 | -1.10092 | 6.79E-16 | 2.03E-14 | yes | down | 86.63 | 89.57 | 95.38 | 42.93 | 42.17 | 36.83 |
| YKR076W | 0.298 | -1.74573 | 1.84E-31 | 2.44E-29 | yes | down | 263.71 | 227.44 | 289.7 | 73.33 | 79.92 | 68.55 |
| YKR077W | 0.267 | -1.90601 | 1.54E-15 | 4.51E-14 | yes | down | 30.78 | 23.13 | 38.26 | 8.31 | 7.33 | 6.82 |
| YKR097W | 0.399 | -1.32583 | 4.22E-05 | 0.000223 | yes | down | 1133.55 | 964.15 | 1026.23 | 371.71 | 569.61 | 181.7 |
| YKR102W | 0.469 | -1.09331 | 1.02E-06 | 7.77E-06 | yes | down | 6.03 | 5.4 | 6.51 | 3.39 | 2.54 | 2.06 |
| YLL002W | 0.36 | -1.47526 | 9.22E-16 | 2.72E-14 | yes | down | 18.54 | 16.78 | 19.7 | 5.89 | 6.71 | 6.14 |
| YLL007C | 0.407 | -1.29853 | 6.95E-05 | 0.000345 | yes | down | 2.82 | 3.4 | 3.4 | 0.75 | 1.09 | 1.64 |
| YLL048C | 0.361 | -1.47176 | 3.88E-31 | 5.02E-29 | yes | down | 34.6 | 35.62 | 36.92 | 12.85 | 11.73 | 12.67 |
| YLL052C | 0.278 | -1.84878 | 2.7E-08 | 2.83E-07 | yes | down | 57.8 | 41.32 | 66.91 | 15.08 | 17.24 | 6.63 |
| YLL053C | 0.333 | -1.58819 | 1.75E-07 | 1.56E-06 | yes | down | 83.99 | 68.12 | 97.22 | 33.8 | 25.57 | 13.62 |
| YLL055W | 0.384 | -1.38173 | 5.86E-06 | 3.77E-05 | yes | down | 70.22 | 84.59 | 96.91 | 34.63 | 38.03 | 15.1 |
| YLL060C | 0.495 | -1.01461 | 1.02E-14 | 2.77E-13 | yes | down | 189.45 | 183.48 | 188.74 | 89.04 | 97.23 | 80.61 |
| YLL061W | 0.152 | -2.72097 | 1.89E-13 | 4.34E-12 | yes | down | 180.6 | 172.52 | 266.6 | 31.66 | 31.42 | 9.02 |
| YLL062C | 0.464 | -1.10718 | 4.98E-06 | 3.24E-05 | yes | down | 21.29 | 26.62 | 25.59 | 11.55 | 13.1 | 7.48 |
| YLR011W | 0.472 | -1.08369 | 0.005801 | 0.016826 | yes | down | 10.21 | 7.25 | 14.37 | 2.32 | 5.2 | 5.27 |
| YLR027C | 0.249 | -2.00776 | 4.16E-21 | 2.05E-19 | yes | down | 1391.07 | 1418.9 | 1446.76 | 382.94 | 375.39 | 225.68 |
| YLR044C | 0.462 | -1.11326 | 7.85E-07 | 6.2E-06 | yes | down | 2810.19 | 3059.24 | 2688.87 | 1138.45 | 977.13 | 1604.38 |
| YLR050C | 0.481 | -1.05544 | 8.98E-05 | 0.000432 | yes | down | 72.96 | 58.26 | 76.05 | 28.81 | 21.82 | 40.03 |
| YLR056W | 0.406 | -1.2989 | 4.02E-10 | 5.94E-09 | yes | down | 98.92 | 103.81 | 148.29 | 45.65 | 41.03 | 47.56 |
| YLR060W | 0.491 | -1.02719 | 3.56E-05 | 0.000191 | yes | down | 45.34 | 46.96 | 70.91 | 31.23 | 25.31 | 18.94 |
| YLR062C | 0.431 | -1.21579 | 7.7E-07 | 6.11E-06 | yes | down | 148.56 | 189.02 | 228.04 | 60.4 | 83.82 | 77.54 |
| YLR070C | 0.188 | -2.41124 | 3.31E-23 | 2.08E-21 | yes | down | 208.88 | 218.99 | 199.27 | 38.37 | 45.43 | 22.54 |
| YLR096W | 0.498 | -1.00661 | 3.46E-05 | 0.000186 | yes | down | 4.15 | 3.55 | 5.54 | 2.45 | 1.95 | 1.82 |
| YLR099C | 0.492 | -1.02372 | 0.000395 | 0.001621 | yes | down | 85.99 | 96.66 | 78.89 | 47.34 | 50.92 | 21.64 |
| YLR120C | 0.168 | -2.57724 | 2.92E-34 | 4.41E-32 | yes | down | 566.7 | 548.4 | 580.37 | 91.79 | 107.99 | 61.16 |
| YLR124W | 0.432 | -1.21011 | 0.001865 | 0.006329 | yes | down | 22.86 | 20.98 | 22.3 | 9.49 | 10.02 | 4.34 |
| YLR153C | 0.189 | -2.40716 | 7.37E-17 | 2.49E-15 | yes | down | 155.58 | 144.74 | 164.39 | 33.66 | 29.17 | 13.51 |
| YLR157C-A | 0.39 | -1.36014 | 2.7E-12 | 5.36E-11 | yes | down | 50.97 | 64.91 | 57.5 | 23.11 | 24.14 | 16.95 |
| YLR164W | 0.228 | -2.13282 | 6.39E-31 | 8.11E-29 | yes | down | 398.95 | 386.62 | 396.34 | 91.93 | 108.28 | 72.26 |
| YLR178C | 0.37 | -1.43438 | 8.88E-22 | 4.77E-20 | yes | down | 2851.92 | 2753.58 | 2830.3 | 915.52 | 935.95 | 1107.13 |
| YLR183C | 0.297 | -1.75211 | 5.33E-05 | 0.000273 | yes | down | 1.17 | 2.29 | 2.38 | 0.39 | 0.33 | 0.58 |
| YLR190W | 0.422 | -1.24539 | 8.26E-06 | 5.15E-05 | yes | down | 14.38 | 11.73 | 15.56 | 6.77 | 6.25 | 3.23 |
| YLR213C | 0.425 | -1.23436 | 2.61E-06 | 1.82E-05 | yes | down | 8.57 | 9.43 | 7.1 | 3.91 | 3.59 | 2.44 |
| YLR227W-A | 0.366 | -1.44859 | 9.35E-10 | 1.29E-08 | yes | down | 36.69 | 41.63 | 54.46 | 17.89 | 16.37 | 11.14 |
| YLR232W | 0.303 | -1.7212 | 1.01E-11 | 1.85E-10 | yes | down | 96.86 | 96.39 | 88.9 | 31.29 | 26.58 | 18.39 |
| YLR251W | 0.477 | -1.06814 | 1.05E-10 | 1.71E-09 | yes | down | 331.72 | 302.06 | 301.22 | 135.51 | 126.38 | 159.92 |
| YLR256W-A | 0.387 | -1.36791 | 6.2E-10 | 8.84E-09 | yes | down | 47.55 | 59.1 | 56.44 | 25.85 | 17.34 | 16.29 |
| YLR264W | 0.315 | -1.66443 | 7.88E-05 | 0.000385 | yes | down | 191.71 | 293.08 | 271.33 | 42.26 | 41.27 | 80.65 |
| YLR274W | 0.497 | -1.00765 | 0.000188 | 0.000842 | yes | down | 2.28 | 2.8 | 2.8 | 1.39 | 1.31 | 0.98 |
| YLR284C | 0.318 | -1.65185 | 2.73E-19 | 1.1E-17 | yes | down | 80 | 84.38 | 68.43 | 23.39 | 26.76 | 19.78 |
| YLR303W | 0.48 | -1.05943 | 0.000662 | 0.002559 | yes | down | 147.98 | 129.74 | 233.81 | 62.74 | 53.44 | 106.39 |
| YLR304C | 0.206 | -2.27773 | 5.03E-21 | 2.42E-19 | yes | down | 1306.22 | 1198.6 | 1257.46 | 283.24 | 274.77 | 147.35 |
| YLR315W | 0.443 | -1.17578 | 0.002724 | 0.008799 | yes | down | 38.52 | 27.28 | 40.71 | 19.42 | 15.96 | 4.78 |
| YLR317W | 0.449 | -1.15484 | 0.006591 | 0.018709 | yes | down | 6.54 | 8.57 | 11.04 | 2.46 | 3.8 | 3.53 |
| YLR339C | 0.349 | -1.51821 | 8.05E-17 | 2.7E-15 | yes | down | 542.97 | 591.82 | 715.36 | 233.4 | 198.3 | 174.65 |
| YLR340W | 0.375 | -1.41394 | 1.03E-16 | 3.44E-15 | yes | down | 675.11 | 740.53 | 892.61 | 308.07 | 254.63 | 260.53 |
| YLR348C | 0.34 | -1.55512 | 9.95E-22 | 5.31E-20 | yes | down | 162.79 | 161.33 | 150.78 | 47.62 | 47.65 | 57.81 |
| YLR349W | 0.301 | -1.7327 | 4.18E-18 | 1.54E-16 | yes | down | 95.14 | 93.26 | 84.8 | 27.86 | 28.86 | 19.64 |
| YLR352W | 0.477 | -1.06857 | 8.09E-08 | 7.64E-07 | yes | down | 25.53 | 25.65 | 24.15 | 10.5 | 14.76 | 9.04 |
| YLR376C | 0.41 | -1.28509 | 1.37E-05 | 8.01E-05 | yes | down | 12.1 | 9.73 | 9.34 | 4.87 | 3.26 | 3.46 |
| YLR377C | 0.267 | -1.90761 | 6.44E-12 | 1.21E-10 | yes | down | 4212.35 | 3695.46 | 3839.14 | 1088.06 | 1203.97 | 517.66 |
| YLR378C | 0.477 | -1.06743 | 3.83E-05 | 0.000204 | yes | down | 266.97 | 252.53 | 260.85 | 131.05 | 149.8 | 69.32 |
| YLR379W | 0.48 | -1.05783 | 4.67E-07 | 3.84E-06 | yes | down | 273.59 | 269.63 | 273.71 | 128.84 | 147.03 | 86.84 |
| YLR392C | 0.466 | -1.1004 | 1.27E-06 | 9.57E-06 | yes | down | 14.77 | 14.42 | 14.95 | 7.51 | 7.52 | 4.5 |
| YLR415C | 0.312 | -1.68191 | 0.002427 | 0.00795 | yes | down | 8.79 | 8.46 | 12.12 | 0.71 | 3.52 | 0 |
| YLR416C | 0.202 | -2.30709 | 5.58E-07 | 4.55E-06 | yes | down | 26.85 | 16.12 | 9.95 | 1.82 | 2.24 | 2.51 |
| YLR432W | 0.39 | -1.35959 | 6.18E-08 | 5.98E-07 | yes | down | 103.99 | 110.35 | 134.75 | 56.78 | 41.55 | 28.41 |
| YML008C | 0.481 | -1.05594 | 1.5E-09 | 2.01E-08 | yes | down | 30.09 | 32.32 | 36.99 | 14.08 | 16.77 | 14.86 |
| YML027W | 0.271 | -1.88568 | 1.78E-29 | 2.02E-27 | yes | down | 98.35 | 102.4 | 93.46 | 22.98 | 23.68 | 28.47 |
| YML040W | 0.373 | -1.42446 | 8.75E-12 | 1.61E-10 | yes | down | 40 | 54.81 | 49.47 | 20.39 | 15.22 | 15.08 |
| YML042W | 0.221 | -2.17755 | 1.59E-12 | 3.27E-11 | yes | down | 1228.02 | 1159.75 | 1050.53 | 214.12 | 329 | 115.52 |
| YML045W-A | 0.373 | -1.42274 | 1.9E-25 | 1.47E-23 | yes | down | 87.8 | 86.48 | 87.08 | 30.44 | 34.8 | 28.42 |
| YML054C | 0.427 | -1.22601 | 8.48E-11 | 1.4E-09 | yes | down | 98.81 | 106.31 | 81.71 | 39.57 | 45.46 | 32.1 |
| YML057W | 0.474 | -1.0781 | 5.51E-07 | 4.49E-06 | yes | down | 167.54 | 148.53 | 154.4 | 78.12 | 85.12 | 49.02 |
| YML075C | 0.411 | -1.2814 | 2.58E-15 | 7.41E-14 | yes | down | 12.26 | 11.67 | 12.1 | 4.28 | 5.37 | 4.56 |
| YML086C | 0.307 | -1.70562 | 4.77E-20 | 2.1E-18 | yes | down | 155.63 | 152.93 | 151.01 | 50.63 | 49.52 | 33.3 |
| YML126C | 0.144 | -2.79512 | 3.31E-25 | 2.45E-23 | yes | down | 100.64 | 83.96 | 111.38 | 10.47 | 18.03 | 8.54 |
| YML131W | 0.498 | -1.00493 | 6.63E-11 | 1.1E-09 | yes | down | 415.7 | 445.01 | 452.94 | 198.78 | 194.26 | 234.27 |
| YMR026C | 0.462 | -1.11295 | 3.02E-07 | 2.57E-06 | yes | down | 35.69 | 44.14 | 35.83 | 21.17 | 16.2 | 13.37 |
| YMR031C | 0.461 | -1.11815 | 1.14E-05 | 6.87E-05 | yes | down | 18.88 | 17.16 | 17.74 | 9.59 | 8.97 | 4.76 |
| YMR051C | 0.326 | -1.61625 | 2.86E-10 | 4.36E-09 | yes | down | 69.4 | 75.51 | 86.77 | 28.4 | 26.92 | 14.27 |
| YMR084W | 0.441 | -1.18204 | 3.78E-08 | 3.8E-07 | yes | down | 51.46 | 49.65 | 44.26 | 17.71 | 25.92 | 16.79 |
| YMR092C | 0.399 | -1.32714 | 1.84E-29 | 2.05E-27 | yes | down | 145.47 | 141.36 | 145.04 | 53.76 | 55.3 | 57.04 |
| YMR116C | 0.282 | -1.82862 | 4.01E-24 | 2.71E-22 | yes | down | 410.56 | 447.06 | 535.97 | 143.48 | 110.23 | 115.45 |
| YMR117C | 0.45 | -1.15081 | 0.004337 | 0.013184 | yes | down | 6.92 | 7.45 | 8.31 | 1.58 | 2.25 | 4.71 |
| YMR118C | 0.194 | -2.36323 | 5.16E-37 | 9.1E-35 | yes | down | 168.19 | 153.71 | 187.37 | 35.66 | 31.03 | 24.81 |
| YMR120C | 0.222 | -2.17203 | 2.12E-25 | 1.6E-23 | yes | down | 95.43 | 111.09 | 111.88 | 24.1 | 25.81 | 15.75 |
| YMR122C | 0.452 | -1.14529 | 5.37E-06 | 3.47E-05 | yes | down | 123.01 | 95.33 | 93.68 | 41.2 | 55.91 | 30.94 |
| YMR133W | 0.483 | -1.05083 | 9.34E-11 | 1.53E-09 | yes | down | 128.96 | 128.76 | 121.22 | 61.82 | 57.68 | 40.58 |
| YMR148W | 0.471 | -1.08771 | 5.04E-14 | 1.25E-12 | yes | down | 379.31 | 389.8 | 333.37 | 161.04 | 171.19 | 157.42 |
| YMR152W | 0.319 | -1.64719 | 1.62E-23 | 1.03E-21 | yes | down | 90.45 | 83.69 | 80.77 | 22.91 | 25.59 | 28.6 |
| YMR169C | 0.444 | -1.17251 | 5.8E-14 | 1.42E-12 | yes | down | 214.03 | 199.44 | 180.66 | 77.51 | 82.82 | 92.18 |
| YMR196W | 0.358 | -1.48001 | 8.4E-34 | 1.18E-31 | yes | down | 149.8 | 149.6 | 155.87 | 51.03 | 57.83 | 48.81 |
| YMR199W | 0.484 | -1.04578 | 0.000372 | 0.001537 | yes | down | 5.37 | 4.41 | 4.13 | 1.38 | 2.39 | 2.43 |
| YMR209C | 0.38 | -1.3951 | 6.16E-11 | 1.03E-09 | yes | down | 16.16 | 22.45 | 18.24 | 6.29 | 6.88 | 7.16 |
| YMR217W | 0.364 | -1.45986 | 7.04E-08 | 6.73E-07 | yes | down | 82.18 | 99.21 | 115.09 | 45.63 | 32.33 | 21.21 |
| YMR246W | 0.322 | -1.63512 | 1.64E-06 | 1.2E-05 | yes | down | 104.59 | 105.25 | 130.34 | 39.15 | 42.91 | 13.4 |
| YMR250W | 0.324 | -1.6249 | 7.45E-12 | 1.38E-10 | yes | down | 840.25 | 777.92 | 813.01 | 198.02 | 200.79 | 326.7 |
| YMR262W | 0.405 | -1.30481 | 2.5E-13 | 5.69E-12 | yes | down | 486.01 | 482.93 | 424.4 | 178.46 | 214.24 | 144.62 |
| YMR267W | 0.41 | -1.28513 | 4.38E-10 | 6.44E-09 | yes | down | 48.89 | 49.79 | 48.29 | 22.21 | 21.05 | 13.82 |
| YMR301C | 0.315 | -1.66795 | 6.42E-25 | 4.48E-23 | yes | down | 32.7 | 30.46 | 36.23 | 11.03 | 10.19 | 8.62 |
| YMR303C | 0.095 | -3.39865 | 5.96E-16 | 1.81E-14 | yes | down | 4790.56 | 4858.24 | 4918.13 | 360.19 | 440.16 | 71.42 |
| YNL045W | 0.316 | -1.66221 | 3.38E-22 | 1.91E-20 | yes | down | 170.09 | 162.9 | 162.2 | 49.38 | 42.05 | 56.79 |
| YNL069C | 0.48 | -1.05978 | 6.62E-07 | 5.32E-06 | yes | down | 326.95 | 358 | 473.3 | 194.22 | 144.24 | 182.79 |
| YNL072W | 0.434 | -1.20415 | 1.63E-14 | 4.32E-13 | yes | down | 240.65 | 242.91 | 212.41 | 108.88 | 97.8 | 82.71 |
| YNL089C | 0.33 | -1.59954 | 0.000104 | 0.000493 | yes | down | 13.68 | 17.34 | 13.92 | 3.36 | 1.69 | 6.37 |
| YNL090W | 0.497 | -1.00789 | 0.001542 | 0.00538 | yes | down | 22.43 | 26.6 | 33.34 | 8.12 | 11.61 | 17.24 |
| YNL117W | 0.143 | -2.80425 | 2.27E-14 | 5.96E-13 | yes | down | 2797.28 | 2464.93 | 2484.5 | 309.04 | 434.55 | 104.58 |
| YNL138W | 0.405 | -1.30487 | 2.83E-08 | 2.96E-07 | yes | down | 115.69 | 111.52 | 109.95 | 42.43 | 56.91 | 28.98 |
| YNL141W | 0.278 | -1.84603 | 1.69E-09 | 2.23E-08 | yes | down | 28.88 | 38.13 | 43.48 | 13.05 | 8.71 | 5.3 |
| YNL142W | 0.119 | -3.07207 | 7.56E-32 | 1.02E-29 | yes | down | 193.84 | 200.75 | 303.11 | 25.8 | 29.73 | 16.3 |
| YNL148C | 0.44 | -1.18347 | 6.98E-05 | 0.000347 | yes | down | 34.11 | 32.68 | 33.17 | 10.53 | 9.48 | 19.68 |
| YNL169C | 0.461 | -1.11774 | 4.26E-14 | 1.07E-12 | yes | down | 130.45 | 122.49 | 124.4 | 60.73 | 60.06 | 46.81 |
| YNL173C | 0.471 | -1.08729 | 6.44E-17 | 2.18E-15 | yes | down | 428.25 | 395.47 | 373.16 | 180.82 | 195.64 | 166.63 |
| YNL178W | 0.497 | -1.00784 | 2.17E-12 | 4.37E-11 | yes | down | 1445.46 | 1511.35 | 1751.49 | 797.5 | 708.47 | 735.83 |
| YNL209W | 0.393 | -1.34812 | 7.06E-09 | 8.43E-08 | yes | down | 523.98 | 539.15 | 678.88 | 282.04 | 204.13 | 156.07 |
| YNL219C | 0.458 | -1.12547 | 3.36E-06 | 2.27E-05 | yes | down | 69.32 | 65.89 | 64.24 | 30.34 | 37.35 | 18.6 |
| YNL239W | 0.301 | -1.73194 | 6.99E-20 | 3E-18 | yes | down | 499.96 | 505.72 | 512.05 | 155.04 | 169.88 | 106.79 |
| YNL274C | 0.471 | -1.08512 | 3.79E-07 | 3.16E-06 | yes | down | 141.22 | 129.96 | 134.79 | 68.55 | 71.28 | 41.96 |
| YNL280C | 0.301 | -1.73436 | 7.26E-24 | 4.75E-22 | yes | down | 87.09 | 82.24 | 98.85 | 21.67 | 28.41 | 26.15 |
| YNL289W | 0.374 | -1.41754 | 0.000382 | 0.001574 | yes | down | 6.56 | 6 | 3.44 | 1.05 | 1.61 | 2.27 |
| YNL307C | 0.38 | -1.39459 | 2.09E-12 | 4.26E-11 | yes | down | 187.81 | 189.21 | 198.95 | 74.65 | 82.57 | 50.37 |
| YNL321W | 0.44 | -1.18453 | 0.000484 | 0.001937 | yes | down | 226.05 | 213.08 | 204.96 | 101.45 | 118.74 | 35.78 |
| YNR002C | 0.345 | -1.53584 | 1.82E-10 | 2.87E-09 | yes | down | 2327.23 | 2427.95 | 2446.86 | 855.84 | 963.29 | 485.81 |
| YNR013C | 0.296 | -1.75472 | 1.56E-12 | 3.22E-11 | yes | down | 29.26 | 28.38 | 35.72 | 11.19 | 8.66 | 5.64 |
| YNR016C | 0.358 | -1.48394 | 2.5E-18 | 9.44E-17 | yes | down | 17.35 | 13.88 | 17.16 | 5.77 | 6.1 | 4.68 |
| YNR021W | 0.35 | -1.51345 | 1.51E-08 | 1.68E-07 | yes | down | 34.04 | 25.9 | 39.13 | 14.37 | 7.8 | 9.53 |
| YNR030W | 0.456 | -1.13266 | 7.86E-06 | 4.91E-05 | yes | down | 7.16 | 8.29 | 7.98 | 2.68 | 2.99 | 4.27 |
| YNR033W | 0.361 | -1.47153 | 4.35E-09 | 5.43E-08 | yes | down | 76.31 | 72.74 | 75.33 | 27.91 | 31.98 | 15.41 |
| YNR050C | 0.431 | -1.21436 | 3.24E-08 | 3.33E-07 | yes | down | 28.35 | 28.36 | 29.45 | 10.69 | 9.45 | 14.69 |
| YNR056C | 0.074 | -3.75569 | 1.38E-52 | 7.94E-50 | yes | down | 167.07 | 220.42 | 192.72 | 9.84 | 9.51 | 15.58 |
| YNR057C | 0.399 | -1.32563 | 4.25E-08 | 4.21E-07 | yes | down | 364.34 | 371.5 | 319.88 | 153.92 | 155.23 | 83.34 |
| YNR058W | 0.133 | -2.91425 | 2.96E-65 | 3.13E-62 | yes | down | 102.52 | 113.52 | 122.97 | 14.84 | 12.11 | 14.83 |
| YOL020W | 0.489 | -1.03154 | 0.009096 | 0.02483 | yes | down | 197.48 | 219.3 | 194.62 | 125.97 | 111.37 | 26.28 |
| YOL030W | 0.411 | -1.2824 | 1.14E-05 | 6.88E-05 | yes | down | 336.47 | 334.43 | 377.03 | 158.2 | 168.95 | 68.73 |
| YOL049W | 0.492 | -1.0238 | 4.13E-08 | 4.11E-07 | yes | down | 25.41 | 19.98 | 24.83 | 10.41 | 10.4 | 12.08 |
| YOL057W | 0.408 | -1.29437 | 1.44E-08 | 1.61E-07 | yes | down | 10.9 | 10.22 | 11.47 | 3.89 | 3.31 | 5.22 |
| YOL059W | 0.386 | -1.37519 | 2.85E-09 | 3.66E-08 | yes | down | 219.74 | 269.1 | 259.13 | 112.37 | 96.95 | 61.4 |
| YOL065C | 0.468 | -1.09392 | 1.45E-13 | 3.39E-12 | yes | down | 51.9 | 51.89 | 47.56 | 22.45 | 24.8 | 20.95 |
| YOL084W | 0.345 | -1.53684 | 2.69E-15 | 7.7E-14 | yes | down | 1304.2 | 1294.22 | 1275 | 438.32 | 515.65 | 313.39 |
| YOL085W-A | 0.477 | -1.0676 | 3.03E-19 | 1.2E-17 | yes | down | 900.18 | 931.13 | 956.78 | 581.42 | 648.85 | 393.62 |
| YOL092W | 0.459 | -1.12312 | 1.21E-17 | 4.35E-16 | yes | down | 238.41 | 247.88 | 277.38 | 116.51 | 115.2 | 105.52 |
| YOL103W-A | 0.295 | -1.76286 | 1.95E-30 | 2.39E-28 | yes | down | 47.39 | 53.64 | 49.84 | 14.72 | 14.94 | 12.78 |
| YOL120C | 0.362 | -1.4658 | 1.25E-18 | 4.8E-17 | yes | down | 1126.47 | 1190.52 | 1470.83 | 474.02 | 428.48 | 391.48 |
| YOL122C | 0.421 | -1.24888 | 2.66E-09 | 3.42E-08 | yes | down | 31.81 | 28.31 | 32.67 | 14.07 | 14.13 | 8.86 |
| YOL126C | 0.42 | -1.25028 | 0.001134 | 0.004129 | yes | down | 5488.95 | 4825.62 | 4433.59 | 1508.24 | 3024.05 | 722.77 |
| YOL153C | 0.46 | -1.11917 | 8.09E-14 | 1.96E-12 | yes | down | 142.69 | 151.13 | 143.91 | 56.46 | 70.81 | 66.52 |
| YOL163W | 0.482 | -1.05284 | 0.000261 | 0.00113 | yes | down | 26.04 | 27.52 | 36.86 | 12.42 | 17.73 | 9.77 |
| YOR006C | 0.448 | -1.15826 | 2.24E-19 | 9.17E-18 | yes | down | 146.8 | 142.32 | 132.89 | 60.81 | 63.9 | 57.24 |
| YOR038C | 0.459 | -1.1242 | 6.14E-06 | 3.92E-05 | yes | down | 6.18 | 7.35 | 7.76 | 3.43 | 3.71 | 2.07 |
| YOR063W | 0.334 | -1.5813 | 9.52E-23 | 5.75E-21 | yes | down | 2511.75 | 2659.65 | 2858.85 | 1000.58 | 833.85 | 724.56 |
| YOR074C | 0.345 | -1.53587 | 1.03E-05 | 6.29E-05 | yes | down | 8.86 | 5.32 | 10.19 | 1.65 | 2.86 | 2.69 |
| YOR084W | 0.483 | -1.04904 | 2.59E-05 | 0.000144 | yes | down | 690.34 | 673.43 | 628.14 | 322.36 | 395.78 | 189.52 |
| YOR086C | 0.368 | -1.44277 | 3.09E-12 | 6.07E-11 | yes | down | 11.82 | 11.27 | 11.67 | 3.34 | 5.2 | 3.55 |
| YOR087W | 0.388 | -1.36662 | 1.98E-07 | 1.74E-06 | yes | down | 10.8 | 11.37 | 8.14 | 4.17 | 4.31 | 2.44 |
| YOR090C | 0.404 | -1.30584 | 2.33E-19 | 9.49E-18 | yes | down | 52.84 | 47.39 | 49.3 | 18.31 | 21.65 | 18.17 |
| YOR094W | 0.458 | -1.12779 | 0.000382 | 0.001574 | yes | down | 29.16 | 23.89 | 31.72 | 7.28 | 10.78 | 16.42 |
| YOR095C | 0.26 | -1.94193 | 1.39E-09 | 1.87E-08 | yes | down | 104.07 | 97.68 | 119.21 | 24.23 | 36.03 | 11.85 |
| YOR100C | 0.299 | -1.74261 | 6.14E-05 | 0.000309 | yes | down | 4343.33 | 4226.43 | 3285.92 | 839.99 | 1649.09 | 227.8 |
| YOR101W | 0.33 | -1.59838 | 1.08E-13 | 2.56E-12 | yes | down | 27.54 | 23.55 | 26.19 | 8.78 | 9.08 | 6 |
| YOR133W | 0.307 | -1.70424 | 7.9E-28 | 7.38E-26 | yes | down | 517.8 | 514.44 | 627.16 | 182.91 | 157.8 | 145.72 |
| YOR142W | 0.369 | -1.4396 | 5.25E-06 | 3.41E-05 | yes | down | 514.58 | 513.59 | 502.56 | 205.37 | 222.34 | 78.54 |
| YOR142W-A | 0.422 | -1.2431 | 2.43E-14 | 6.36E-13 | yes | down | 70.47 | 60.52 | 71.73 | 27.08 | 31.31 | 23.69 |
| YOR157C | 0.327 | -1.61272 | 9.84E-09 | 1.13E-07 | yes | down | 292.51 | 285.44 | 308 | 109.66 | 105.27 | 47.33 |
| YOR161C | 0.174 | -2.52111 | 4.76E-11 | 8.11E-10 | yes | down | 2117.83 | 2192.04 | 2079.49 | 333.67 | 425.61 | 92.68 |
| YOR176W | 0.267 | -1.90665 | 6.64E-26 | 5.55E-24 | yes | down | 245.31 | 246.45 | 254.48 | 52.65 | 60.25 | 73.69 |
| YOR180C | 0.422 | -1.24399 | 4.88E-07 | 4E-06 | yes | down | 24.25 | 24.79 | 20.59 | 6.91 | 11.74 | 8.67 |
| YOR192C-A | 0.471 | -1.08645 | 4.85E-06 | 3.17E-05 | yes | down | 15.43 | 12.55 | 16.4 | 6.3 | 8.39 | 5.03 |
| YOR217W | 0.376 | -1.41305 | 8.46E-05 | 0.000411 | yes | down | 3.25 | 2 | 3.73 | 0.89 | 0.62 | 1.4 |
| YOR228C | 0.487 | -1.03797 | 0.000629 | 0.002449 | yes | down | 514.4 | 477.58 | 454.14 | 258.24 | 284.55 | 107.62 |
| YOR273C | 0.42 | -1.25319 | 6.47E-13 | 1.41E-11 | yes | down | 170.24 | 168.54 | 165.89 | 73.77 | 76.83 | 52.36 |
| YOR321W | 0.47 | -1.09043 | 3.07E-07 | 2.61E-06 | yes | down | 44.47 | 40.48 | 43.2 | 21.52 | 22.52 | 13.34 |
| YOR343W-A | 0.401 | -1.31896 | 0.000204 | 0.000907 | yes | down | 29.15 | 28.02 | 37.67 | 14.38 | 14.63 | 4.51 |
| YOR356W | 0.379 | -1.39986 | 9.12E-13 | 1.97E-11 | yes | down | 99.68 | 99.42 | 100.72 | 41.48 | 40.34 | 26.2 |
| YOR373W | 0.38 | -1.39631 | 7.84E-09 | 9.2E-08 | yes | down | 238.53 | 223.14 | 209.74 | 86.64 | 101.31 | 51.01 |
| YOR374W | 0.122 | -3.0302 | 7.16E-23 | 4.41E-21 | yes | down | 5832.61 | 6115.57 | 5634.81 | 640.36 | 839.73 | 295.08 |
| YOR382W | 0.38 | -1.39571 | 0.000173 | 0.000778 | yes | down | 3244.81 | 3269.72 | 4086.95 | 1837.31 | 1155.79 | 433.98 |
| YPL003W | 0.488 | -1.03415 | 1.47E-13 | 3.43E-12 | yes | down | 186.27 | 193.27 | 166.23 | 83.02 | 93.36 | 80.72 |
| YPL004C | 0.484 | -1.04694 | 1.53E-15 | 4.5E-14 | yes | down | 5665.85 | 5575.79 | 5415.32 | 2335.75 | 2505.95 | 2799.26 |
| YPL028W | 0.326 | -1.61794 | 3.2E-25 | 2.39E-23 | yes | down | 510.23 | 465.49 | 524.4 | 167.92 | 171.16 | 127.59 |
| YPL036W | 0.332 | -1.59076 | 3.15E-14 | 8.07E-13 | yes | down | 7.24 | 8.55 | 8.05 | 2.38 | 3.03 | 2.04 |
| YPL054W | 0.414 | -1.27306 | 2.36E-10 | 3.64E-09 | yes | down | 283.11 | 282.89 | 273.69 | 111.24 | 136.88 | 81.18 |
| YPL061W | 0.134 | -2.90254 | 5.05E-15 | 1.41E-13 | yes | down | 2214.33 | 2397.68 | 2354.76 | 244.14 | 372.1 | 86.23 |
| YPL062W | 0.447 | -1.16304 | 0.002043 | 0.006849 | yes | down | 13.13 | 15.52 | 12.63 | 4.38 | 5.19 | 6.05 |
| YPL104W | 0.354 | -1.49783 | 2.01E-08 | 2.16E-07 | yes | down | 9.88 | 7.9 | 10.22 | 2.3 | 4.24 | 2.55 |
| YPL116W | 0.324 | -1.6252 | 7.99E-08 | 7.56E-07 | yes | down | 20.3 | 19.93 | 15.58 | 5.88 | 7.47 | 2.91 |
| YPL131W | 0.31 | -1.68783 | 1.35E-21 | 7.11E-20 | yes | down | 1039.5 | 1104.2 | 1329.56 | 399.32 | 319.51 | 299.56 |
| YPL223C | 0.307 | -1.70578 | 1.06E-11 | 1.94E-10 | yes | down | 359.86 | 341.12 | 443.05 | 87.94 | 85.72 | 140.77 |
| YPL231W | 0.214 | -2.2223 | 1.88E-21 | 9.72E-20 | yes | down | 96.62 | 99.07 | 126.71 | 16.21 | 19.84 | 26.69 |
| YPL246C | 0.206 | -2.27616 | 4.34E-46 | 1.45E-43 | yes | down | 224.14 | 236.5 | 256.36 | 40.64 | 49.7 | 48.6 |
| YPL257W-A | 0.395 | -1.34026 | 5.88E-12 | 1.11E-10 | yes | down | 63.04 | 57.78 | 56.86 | 25.79 | 24.34 | 16.6 |
| YPL262W | 0.424 | -1.23723 | 3.55E-34 | 5.25E-32 | yes | down | 1505.73 | 1436.49 | 1489.1 | 642.37 | 632.34 | 622.96 |
| YPL270W | 0.475 | -1.07285 | 2.99E-10 | 4.52E-09 | yes | down | 110.18 | 107.18 | 102.21 | 49.83 | 57.28 | 39.1 |
| YPL274W | 0.351 | -1.51113 | 1.27E-07 | 1.16E-06 | yes | down | 315.94 | 316.52 | 320.8 | 109.63 | 140.16 | 55.35 |
| YPR001W | 0.253 | -1.98221 | 2.93E-13 | 6.65E-12 | yes | down | 431.58 | 466.36 | 413.36 | 116.16 | 126.17 | 56.58 |
| YPR002W | 0.149 | -2.74664 | 1.31E-41 | 3.09E-39 | yes | down | 217.41 | 217.94 | 210.73 | 30.38 | 36.02 | 21.21 |
| YPR006C | 0.273 | -1.87143 | 7.41E-12 | 1.38E-10 | yes | down | 266.12 | 292.56 | 289.37 | 89.46 | 80.71 | 39.41 |
| YPR083W | 0.479 | -1.06191 | 4.01E-05 | 0.000213 | yes | down | 24.89 | 21.01 | 21.96 | 13.54 | 10.56 | 6.47 |
| YPR128C | 0.49 | -1.02985 | 1.3E-06 | 9.76E-06 | yes | down | 41.88 | 35.19 | 54.02 | 22.73 | 20.15 | 17.92 |
| YPR137C-A | 0.435 | -1.19952 | 1.02E-09 | 1.4E-08 | yes | down | 89.66 | 95.67 | 105.3 | 46.83 | 43.65 | 30.07 |
| YPR155C | 0.393 | -1.34882 | 1.33E-17 | 4.76E-16 | yes | down | 25.91 | 28.23 | 26.77 | 10.07 | 11.39 | 9.02 |
| YPR158C-C | 0.411 | -1.28428 | 1.46E-12 | 3.05E-11 | yes | down | 52.67 | 66.18 | 63 | 22.96 | 27.61 | 20.66 |
| YPR158W-A | 0.414 | -1.27165 | 1.6E-06 | 1.18E-05 | yes | down | 64.42 | 57.88 | 68.86 | 23.65 | 34.38 | 15.54 |
| YPR160W | 0.418 | -1.25674 | 1.28E-07 | 1.17E-06 | yes | down | 78.57 | 75.15 | 87.24 | 25.44 | 26.51 | 42.05 |
| YPR164W | 0.341 | -1.55158 | 1.01E-07 | 9.34E-07 | yes | down | 1.89 | 2.2 | 1.85 | 0.52 | 0.87 | 0.45 |
| YPR171W | 0.438 | -1.19146 | 1.46E-05 | 8.53E-05 | yes | down | 16.45 | 14.08 | 14.2 | 6.49 | 8.12 | 3.6 |
| YPR184W | 0.418 | -1.25708 | 0.000508 | 0.002019 | yes | down | 121.75 | 124.82 | 104.28 | 50.67 | 63.01 | 16.4 |
| YPR192W | 0.047 | -4.41801 | 1.14E-39 | 2.5E-37 | yes | down | 1210.12 | 1364.08 | 1487.46 | 58.37 | 58.28 | 19.19 |
| YPR193C | 0.335 | -1.57566 | 2.23E-06 | 1.58E-05 | yes | down | 39.95 | 39.4 | 49.83 | 18.94 | 11.95 | 6.59 |
