# Supplementary material for: Hydrogen sulfide treatment at the late growth stage of Saccharomyces cerevisiae extends chronological lifespan
Source: Aging (Albany NY). 2021 Mar 19;13(7):9859–73. doi: 10.18632/aging.202738 (PMC8064171; doi:10.18632/aging.202738)
Supplement: Supplementary Table 2 [file aging-13-202738-s003.doc]

Supplementary Table 2. DEGs induced by the late NaHS treatment.

| Gene_id | FC(NaHS/Control) | Log2FC(NaHS/Control) | Pvalue | Padjust | Significant | Regulate | Control1_Fpkm | Control2_Fpkm | Control3_Fpkm | NaHS1_Fpkm | NaHS2_Fpkm | NaHS3_Fpkm |
| --- | --- | --- | --- | --- | --- | --- | --- | --- | --- | --- | --- | --- |
| YER130C | 3.119 | 1.641 | 1.2E-09 | 2.87E-08 | yes | up | 34.727 | 23.888 | 47.264 | 110.918 | 135.449 | 111.303 |
| YDL213C | 2.024 | 1.017 | 0.000356 | 0.002802 | yes | up | 49.072 | 23.949 | 42.536 | 77.419 | 88.479 | 81.284 |
| YBR208C | 2.018 | 1.013 | 0.001588 | 0.00991 | yes | up | 147.965 | 60.569 | 119.4 | 279.366 | 236.78 | 198.134 |
| YDR353W | 2.201 | 1.138 | 3.66E-12 | 1.11E-10 | yes | up | 340.78 | 382.14 | 311.59 | 694.4 | 875.356 | 781.472 |
| YPL014W | 5.909 | 2.563 | 9.46E-31 | 9.37E-29 | yes | up | 14.901 | 24.158 | 22.895 | 127.733 | 131.531 | 133.698 |
| YDR258C | 249 | 7.96 | 9.01E-58 | 4.24E-55 | yes | up | 0 | 0 | 0 | 13.329 | 25.515 | 22.059 |
| YGL162W | 2.774 | 1.472 | 1.01E-15 | 4.22E-14 | yes | up | 28.982 | 26.51 | 31.489 | 78.831 | 81.949 | 90.598 |
| YKL096W-A | 4.101 | 2.036 | 8.68E-12 | 2.57E-10 | yes | up | 992.291 | 1547.94 | 1069.539 | 3379.441 | 5019.082 | 7151.962 |
| YDL048C | 7.749 | 2.954 | 1.9E-19 | 1.04E-17 | yes | up | 65.843 | 127.023 | 158.782 | 1403.466 | 874.721 | 913.979 |
| YFL017C | 3.176 | 1.667 | 0.001614 | 0.01004 | yes | up | 8.198 | 8.816 | 0.742 | 16.105 | 33.616 | 26.579 |
| YHR092C | 4.727 | 2.241 | 0.000161 | 0.001373 | yes | up | 24.203 | 192.418 | 103.448 | 1502.794 | 1041.44 | 343.223 |
| YGL062W | 2.723 | 1.445 | 1.73E-09 | 4.05E-08 | yes | up | 84.666 | 72.217 | 107.414 | 295.199 | 259.447 | 208.894 |
| YGL079W | 2.147 | 1.102 | 8.53E-05 | 0.000813 | yes | up | 11.764 | 15.859 | 11.769 | 33.639 | 24.114 | 30.366 |
| YMR244W | 2.216 | 1.148 | 0.000347 | 0.002745 | yes | up | 671.556 | 415.484 | 242.4 | 1091.396 | 1102.028 | 978.161 |
| YMR233W | 2.042 | 1.03 | 1.06E-05 | 0.000126 | yes | up | 20.911 | 15.884 | 16.951 | 37.677 | 36.162 | 40.24 |
| YDR246W-A | 2.483 | 1.312 | 0.03421 | 0.1157 | yes | up | 0 | 0 | 4.421 | 34.863 | 20.811 | 94.242 |
| YHL046W-A | 2.641 | 1.401 | 0.002252 | 0.01311 | yes | up | 29.848 | 10.343 | 13.658 | 32.76 | 63.182 | 65.872 |
| YJR003C | 2.098 | 1.069 | 0.000383 | 0.002986 | yes | up | 5.116 | 2.931 | 4.352 | 7.244 | 10.032 | 10.851 |
| YOR388C | 2.296 | 1.199 | 5.07E-05 | 0.000509 | yes | up | 4.742 | 6.329 | 6.389 | 10.44 | 19.061 | 13.661 |
| YBL043W | 2.227 | 1.155 | 0.000433 | 0.003322 | yes | up | 94.843 | 65.445 | 131.466 | 323.277 | 196.766 | 173.866 |
| YOR267C | 2.755 | 1.462 | 0.02513 | 0.09051 | yes | up | 13.442 | 0.48 | 22.114 | 88.318 | 66.948 | 94.15 |
| YLR216C | 2.145 | 1.101 | 3.18E-06 | 4.21E-05 | yes | up | 68.233 | 49.265 | 62.158 | 98.926 | 152.863 | 155.37 |
| YDR096W | 2.192 | 1.132 | 7.12E-05 | 0.000697 | yes | up | 1.833 | 2.216 | 3.214 | 5.823 | 5.129 | 6.026 |
| YKL217W | 2.218 | 1.149 | 0.000173 | 0.001472 | yes | up | 100.634 | 170.328 | 149.07 | 407.716 | 383.577 | 200.567 |
| YGR103W | 2.299 | 1.201 | 0.001654 | 0.01024 | yes | up | 8.408 | 3.46 | 7.16 | 10.515 | 25.458 | 13.641 |
| YJL034W | 2.44 | 1.287 | 1.26E-08 | 2.66E-07 | yes | up | 148.357 | 112.555 | 159.573 | 304.453 | 341.717 | 440 |
| YLR089C | 2.354 | 1.235 | 1.72E-05 | 0.000196 | yes | up | 93.995 | 159.37 | 131.802 | 428.111 | 238.067 | 292.763 |
| YDR033W | 9.164 | 3.196 | 5.11E-43 | 1.11E-40 | yes | up | 194.639 | 123.612 | 145.104 | 1269.031 | 1804.874 | 1617.547 |
| YMR104C | 409.012 | 8.676 | 6.4E-73 | 9.03E-70 | yes | up | 0 | 0 | 0 | 29.685 | 48.92 | 50.979 |
| YGR138C | 2.537 | 1.343 | 1.84E-12 | 5.91E-11 | yes | up | 1627.487 | 1227.243 | 1353.675 | 3404.331 | 4202.943 | 3588.273 |
| YCL026C-B | 20.563 | 4.362 | 1.27E-13 | 4.56E-12 | yes | up | 0 | 0.135 | 0 | 11.842 | 17.083 | 5.833 |
| YFL016C | 3.673 | 1.877 | 0.001273 | 0.008255 | yes | up | 32.675 | 4.211 | 22.42 | 84.532 | 128.502 | 157.243 |
| YLR455W | 2.205 | 1.141 | 0.000135 | 0.00119 | yes | up | 10.086 | 5.8 | 9.039 | 15.254 | 22.553 | 21.469 |
| YER098W | 2.469 | 1.304 | 1.07E-06 | 1.56E-05 | yes | up | 1.842 | 2.586 | 2.769 | 6.206 | 7.098 | 5.629 |
| YOR382W | 5.517 | 2.464 | 1.63E-16 | 7.29E-15 | yes | up | 801.099 | 1863.354 | 1137.591 | 8493.177 | 7842.776 | 6683.012 |
| YLR297W | 3.945 | 1.98 | 1.12E-13 | 4.06E-12 | yes | up | 147.4 | 140.555 | 213.057 | 839.434 | 662.983 | 557.472 |
| YHR126C | 3.866 | 1.951 | 0.001172 | 0.007698 | yes | up | 161.298 | 44.524 | 25.318 | 255.363 | 543.49 | 825.549 |
| YOR092W | 3.292 | 1.719 | 2.57E-12 | 8.11E-11 | yes | up | 16.525 | 23.42 | 24.369 | 81.887 | 58.279 | 85.396 |
| YGR158C | 8.926 | 3.158 | 1.27E-06 | 1.82E-05 | yes | up | 0 | 0 | 0 | 3.15 | 0.558 | 13.61 |
| YDR249C | 2.331 | 1.221 | 0.000183 | 0.001556 | yes | up | 10.579 | 4.962 | 9.385 | 15.45 | 23.698 | 24.787 |
| YDR042C | 2.383 | 1.253 | 0.000119 | 0.001068 | yes | up | 4.724 | 6.908 | 6.122 | 13.833 | 16.042 | 15.33 |
| YDL049C | 3.699 | 1.887 | 1.73E-09 | 4.05E-08 | yes | up | 11.618 | 9.481 | 7.586 | 27.695 | 34.837 | 55.56 |
| YGR211W | 2.336 | 1.224 | 1.07E-07 | 1.88E-06 | yes | up | 172.798 | 123.44 | 141.524 | 327.885 | 303.104 | 448.184 |
| YGR292W | 2.478 | 1.309 | 9.31E-05 | 0.000879 | yes | up | 2.718 | 1.798 | 1.741 | 7.029 | 4.685 | 5.212 |
| YGR181W | 2.11 | 1.077 | 0.0179 | 0.0701 | yes | up | 12.476 | 19.541 | 31.766 | 44.967 | 66.977 | 35.231 |
| YIL162W | 4.19 | 2.067 | 5.25E-11 | 1.42E-09 | yes | up | 4.496 | 3.435 | 5.034 | 27.199 | 19.505 | 14.2 |
| YBR150C | 2.024 | 1.017 | 0.002848 | 0.01585 | yes | up | 0.903 | 0.837 | 1.019 | 1.542 | 2.839 | 1.7 |
| YGL176C | 6.765 | 2.758 | 2.42E-05 | 0.000268 | yes | up | 0 | 0 | 0 | 0.682 | 1.174 | 0.285 |
| YGL174W | 22.331 | 4.481 | 1.98E-14 | 7.59E-13 | yes | up | 0 | 0.062 | 0.158 | 10.01 | 3.748 | 8.032 |
| YKR052C | 152.008 | 7.248 | 2.03E-43 | 4.77E-41 | yes | up | 0 | 0 | 0 | 59.034 | 54.976 | 24.095 |
| YPR130C | 2.27 | 1.183 | 0.04532 | 0.1428 | yes | up | 2.544 | 4.901 | 1.019 | 6.552 | 13.316 | 5.823 |
| YKR061W | 2.028 | 1.02 | 4.34E-06 | 5.61E-05 | yes | up | 62.241 | 47.861 | 55.897 | 89.589 | 123.212 | 141.17 |
| YDR360W | 2.721 | 1.444 | 0.001668 | 0.01031 | yes | up | 6.876 | 3.854 | 3.303 | 16.983 | 12.966 | 14.027 |
| YOR105W | 2.025 | 1.018 | 0.03799 | 0.125 | yes | up | 18.403 | 10.442 | 8.357 | 17.31 | 42.815 | 26.416 |
| YJL213W | 69.118 | 6.111 | 5.56E-29 | 5.32E-27 | yes | up | 0 | 0 | 0 | 17.88 | 16.685 | 9.162 |
| YJL012C | 2.107 | 1.075 | 1.78E-07 | 3.02E-06 | yes | up | 38.512 | 31.633 | 34.169 | 60.558 | 91.678 | 78.871 |
| YNL053W | 2.039 | 1.028 | 3.16E-05 | 0.00034 | yes | up | 24.878 | 19.27 | 24.497 | 56.941 | 37.856 | 51.783 |
| YGL255W | 2.594 | 1.375 | 4.6E-06 | 5.91E-05 | yes | up | 1045.276 | 716.086 | 630.498 | 2800.576 | 2417.546 | 1444.658 |
| YNL077W | 2.683 | 1.424 | 1.07E-12 | 3.54E-11 | yes | up | 56.487 | 76.071 | 70.445 | 153.801 | 222.016 | 196.282 |
| YNL125C | 4.438 | 2.15 | 7.3E-13 | 2.47E-11 | yes | up | 59.87 | 105.598 | 115.158 | 584.809 | 456.26 | 336.942 |
| YOL154W | 3.95 | 1.982 | 2.93E-17 | 1.39E-15 | yes | up | 58.037 | 63.093 | 38.62 | 203.676 | 262.295 | 204.69 |
| YMR058W | 4.17 | 2.06 | 4.59E-28 | 4.25E-26 | yes | up | 469.74 | 551.261 | 447.378 | 2660.571 | 2184.192 | 2236.065 |
| YAL018C | 2.512 | 1.329 | 0.005218 | 0.02587 | yes | up | 0.711 | 0.751 | 1.256 | 2 | 2.811 | 3.542 |
| YMR166C | 26.983 | 4.754 | 4.74E-16 | 2.06E-14 | yes | up | 0 | 0 | 0 | 4.028 | 4.429 | 3.471 |
| YLR142W | 2.018 | 1.013 | 4.24E-05 | 0.000438 | yes | up | 16.397 | 14 | 18.722 | 40.93 | 35.112 | 27.81 |
| YDL055C | 2.277 | 1.187 | 4.08E-07 | 6.62E-06 | yes | up | 931.682 | 837.21 | 731.029 | 1360.732 | 2379.813 | 2276.071 |
| YNR004W | 2.266 | 1.18 | 0.000836 | 0.005793 | yes | up | 9.092 | 19.676 | 18.642 | 36.032 | 37.071 | 41.064 |
| YGL008C | 2.738 | 1.453 | 3.98E-06 | 5.2E-05 | yes | up | 1860.437 | 749.011 | 1131.618 | 2949.339 | 4073.494 | 4363.943 |
| YPL005W | 2.826 | 1.499 | 2.24E-14 | 8.55E-13 | yes | up | 17.263 | 16.438 | 19.246 | 51.071 | 45.91 | 60.192 |
| YKL062W | 2.092 | 1.065 | 0.000751 | 0.005314 | yes | up | 3.657 | 3.263 | 3.857 | 11.076 | 6.303 | 6.637 |
| YDR171W | 2.512 | 1.329 | 3.97E-09 | 8.84E-08 | yes | up | 156.592 | 160.712 | 172.548 | 391.471 | 340.146 | 562.51 |
| YAL034W-A | 2.091 | 1.064 | 0.01475 | 0.06097 | yes | up | 1.696 | 1.231 | 1.948 | 3.187 | 3.322 | 5.141 |
| YNL144C | 3.16 | 1.66 | 5.71E-09 | 1.25E-07 | yes | up | 2.116 | 2.943 | 3.422 | 11.459 | 9.511 | 7.981 |
| YPR142C | 2.088 | 1.062 | 0.02904 | 0.1021 | yes | up | 3.465 | 2.056 | 5.776 | 5.365 | 14.082 | 8.612 |
| YGR042W | 6.797 | 2.765 | 2.24E-05 | 0.000251 | yes | up | 0 | 0 | 0 | 1.72 | 1.211 | 1.802 |
| YLR126C | 2.014 | 1.01 | 0.00021 | 0.001765 | yes | up | 14.901 | 10.072 | 10.701 | 25.292 | 28.392 | 21.998 |
| YGR169C | 134.271 | 7.069 | 9.74E-43 | 2.04E-40 | yes | up | 0 | 0 | 0 | 19.909 | 17.3 | 29.256 |
| YPL144W | 2.521 | 1.334 | 0.005073 | 0.02531 | yes | up | 5.982 | 33.516 | 12.362 | 40.836 | 68.87 | 44.861 |
| YDR380W | 195.361 | 7.61 | 2.53E-47 | 8.41E-45 | yes | up | 0 | 0 | 0 | 49.173 | 18.445 | 18.415 |
| YLR069C | 2.333 | 1.222 | 5.14E-05 | 0.000515 | yes | up | 4.979 | 2.82 | 3.234 | 7.365 | 11.849 | 8.846 |
| YPL240C | 2.556 | 1.354 | 1.83E-13 | 6.52E-12 | yes | up | 68.689 | 59.633 | 75.282 | 155.587 | 198.65 | 191.67 |
| YHR137W | 33.498 | 5.066 | 6.51E-35 | 8.55E-33 | yes | up | 32.019 | 7.314 | 27.593 | 1474.763 | 1210.912 | 893.08 |
| YDR309C | 2.745 | 1.457 | 4.18E-05 | 0.000433 | yes | up | 18.622 | 15.539 | 14.152 | 53.716 | 23.405 | 70.117 |
| YER095W | 2.041 | 1.029 | 2.77E-05 | 0.000303 | yes | up | 223.055 | 435.16 | 303.708 | 659.032 | 713.587 | 677.386 |
| YBR101C | 2.086 | 1.061 | 1E-09 | 2.43E-08 | yes | up | 148.166 | 140.567 | 159.494 | 286.385 | 311.158 | 368.794 |
| YBR054W | 3.165 | 1.662 | 2.81E-08 | 5.58E-07 | yes | up | 685.755 | 303.729 | 492.347 | 1345.432 | 2070.358 | 1745.015 |
| YIL002W-A | 2.101 | 1.071 | 0.003888 | 0.02041 | yes | up | 255.822 | 227.277 | 182.359 | 529.28 | 474.592 | 384.399 |
| YPL111W | 2.285 | 1.192 | 0.000115 | 0.00104 | yes | up | 262.47 | 495.827 | 528.88 | 1310.26 | 1075.273 | 744.765 |
| YDR264C | 2.173 | 1.12 | 0.007668 | 0.03576 | yes | up | 29.347 | 5.935 | 26.287 | 52.669 | 53.387 | 47.233 |
| YDR055W | 5.256 | 2.394 | 9.36E-25 | 7.44E-23 | yes | up | 96.54 | 122.726 | 147.596 | 521.831 | 790.605 | 782.755 |
| YAL056C-A | 2.904 | 1.538 | 0.0191 | 0.07382 | yes | up | 0.538 | 0 | 1.305 | 2.028 | 3.767 | 13.885 |
| YNL024C | 110.814 | 6.792 | 2.5E-37 | 3.82E-35 | yes | up | 0 | 0 | 0 | 23.199 | 42.077 | 60.446 |
| YKR093W | 2.196 | 1.135 | 0.000723 | 0.00518 | yes | up | 19.16 | 21.006 | 19.651 | 71.447 | 42.948 | 26.375 |
| YDR342C | 2.88 | 1.526 | 0.000198 | 0.00167 | yes | up | 213.872 | 1089.409 | 695.564 | 2410.32 | 2550.345 | 1670.338 |
| YGR023W | 2.774 | 1.472 | 3.01E-09 | 6.8E-08 | yes | up | 25.279 | 37.26 | 45.385 | 99.74 | 96.145 | 122.49 |
| YMR290W-A | 2.439 | 1.286 | 0.001465 | 0.009288 | yes | up | 33.423 | 27.84 | 22.025 | 81.139 | 39.039 | 98.222 |
| YIL131C | 19.041 | 4.251 | 6.37E-12 | 1.9E-10 | yes | up | 0 | 0 | 0 | 0.963 | 2.811 | 5.232 |
| YDR068W | 2.004 | 1.003 | 0.005198 | 0.0258 | yes | up | 1.915 | 2.413 | 2.848 | 5.094 | 5.338 | 5.12 |
| YML053C | 18.252 | 4.19 | 9.37E-12 | 2.76E-10 | yes | up | 0 | 0 | 0 | 3.44 | 11.764 | 7.899 |
| YDR270W | 2.511 | 1.328 | 1.01E-06 | 1.49E-05 | yes | up | 6.995 | 5.258 | 7.704 | 22.629 | 15.568 | 15.218 |
| YHR030C | 2.333 | 1.222 | 1.72E-06 | 2.41E-05 | yes | up | 119.849 | 98.333 | 125.285 | 210.162 | 251.014 | 393.205 |
| YOR051C | 86.044 | 6.427 | 2.02E-33 | 2.39E-31 | yes | up | 0 | 0 | 0 | 9.468 | 12.369 | 17.56 |
| YPR157W | 5.22 | 2.384 | 2.88E-16 | 1.26E-14 | yes | up | 17.765 | 32.137 | 41.31 | 190.861 | 171.621 | 168.349 |
| YGR152C | 18.038 | 4.173 | 2.12E-11 | 5.96E-10 | yes | up | 0 | 0 | 0 | 2.196 | 4.514 | 13.264 |
| YLL024C | 2.89 | 1.531 | 1.06E-09 | 2.55E-08 | yes | up | 240.337 | 151.957 | 171.53 | 491.725 | 521.079 | 737.782 |
| YHL040C | 8.011 | 3.002 | 1.57E-44 | 4.22E-42 | yes | up | 150.436 | 229.235 | 190.805 | 1646.034 | 1887.012 | 1409.559 |
| YGR273C | 2.799 | 1.485 | 0.0227 | 0.08364 | yes | up | 0 | 0.271 | 0 | 0.785 | 1.779 | 1.863 |
| YER137W-A | 2.643 | 1.402 | 0.003269 | 0.01779 | yes | up | 11.7 | 7.807 | 12.432 | 28.872 | 37.705 | 31.282 |
| YKL109W | 10.44 | 3.384 | 5.24E-31 | 5.38E-29 | yes | up | 4.76 | 6.083 | 9.959 | 95.412 | 85.375 | 71.46 |
| YDR475C | 2.102 | 1.072 | 9.35E-08 | 1.66E-06 | yes | up | 12.786 | 13.495 | 17.752 | 30.181 | 32.887 | 33.735 |
| YGL179C | 4.187 | 2.066 | 1.16E-20 | 6.89E-19 | yes | up | 7.597 | 9.666 | 11.502 | 44.771 | 43.686 | 39.873 |
| YMR307W | 3.123 | 1.643 | 4.91E-25 | 3.96E-23 | yes | up | 1012.281 | 857.552 | 895.982 | 2784.864 | 3087.958 | 3140.068 |
| YPR158W | 2.535 | 1.342 | 4.38E-05 | 0.00045 | yes | up | 11.554 | 7.265 | 12.689 | 26.04 | 21.843 | 39.324 |
| YDL182W | 2.815 | 1.493 | 2.3E-18 | 1.18E-16 | yes | up | 107.456 | 113.7 | 105.08 | 271.832 | 313.42 | 372.143 |
| YDR219C | 2.042 | 1.03 | 0.007545 | 0.03536 | yes | up | 2.043 | 0.997 | 2.591 | 4.141 | 4.278 | 4.387 |
| YPR079W | 2.104 | 1.073 | 6.79E-09 | 1.45E-07 | yes | up | 18.868 | 22.028 | 23.33 | 42.556 | 48.011 | 49.584 |
| YJR148W | 5.449 | 2.446 | 6.92E-30 | 6.74E-28 | yes | up | 256.798 | 294.162 | 262.625 | 1856.336 | 1624.423 | 1239.317 |
| YHR159W | 2.034 | 1.024 | 0.000423 | 0.003247 | yes | up | 2.572 | 2.401 | 3.62 | 5.711 | 6.521 | 6.444 |
| YIL120W | 77.332 | 6.273 | 2.67E-31 | 2.79E-29 | yes | up | 0 | 0 | 0 | 10.104 | 7.742 | 5.914 |
| YPR156C | 3.948 | 1.981 | 4.87E-16 | 2.1E-14 | yes | up | 65.25 | 63.007 | 99.621 | 348.14 | 278.659 | 341.309 |
| YOR232W | 10.375 | 3.375 | 2.19E-11 | 6.12E-10 | yes | up | 1.031 | 1.034 | 0.762 | 21.357 | 21.162 | 5.619 |
| YAR066W | 2.516 | 1.331 | 0.004008 | 0.02094 | yes | up | 4.998 | 1.416 | 4.816 | 7.664 | 13.259 | 13.101 |
| YJR078W | 2.011 | 1.008 | 2.39E-06 | 3.26E-05 | yes | up | 45.197 | 73.214 | 58.311 | 124.35 | 126.051 | 118.144 |
| YBR169C | 2.908 | 1.54 | 2.76E-11 | 7.59E-10 | yes | up | 21.896 | 15.564 | 22.025 | 49.585 | 62.699 | 72.641 |
| YHR153C | 2.504 | 1.324 | 0.001531 | 0.009605 | yes | up | 2.727 | 2.487 | 3.461 | 7.73 | 6.88 | 10.149 |
| YPR027C | 2.118 | 1.083 | 0.000636 | 0.004638 | yes | up | 5.162 | 8.422 | 7.012 | 16.609 | 18.095 | 11.594 |
| YCR026C | 618.23 | 9.272 | 1.47E-98 | 4.16E-95 | yes | up | 0 | 0 | 0.02 | 19.357 | 26.679 | 16.867 |
| YOR383C | 3.883 | 1.957 | 2.55E-18 | 1.3E-16 | yes | up | 452.011 | 588.004 | 384.201 | 2668.366 | 2498.634 | 1969.555 |
| YGR260W | 2.398 | 1.262 | 3.04E-08 | 6.01E-07 | yes | up | 423.038 | 553.354 | 349.399 | 927.612 | 1052.645 | 1364.993 |
| YFL051C | 2.415 | 1.272 | 0.01216 | 0.05233 | yes | up | 2.535 | 4.814 | 3.511 | 14.301 | 12.303 | 4.968 |
| YBL005W-B | 2.104 | 1.073 | 0.007464 | 0.03513 | yes | up | 0.264 | 0.197 | 0.336 | 0.598 | 0.502 | 0.784 |
| YGR279C | 2.388 | 1.256 | 4.2E-09 | 9.3E-08 | yes | up | 171.402 | 185.387 | 119.816 | 365.179 | 376.98 | 447.512 |
| YDR222W | 4.24 | 2.084 | 6.8E-06 | 8.46E-05 | yes | up | 33.131 | 4.581 | 13.599 | 69.923 | 101.634 | 117.879 |
| YPR065W | 3.249 | 1.7 | 4.85E-07 | 7.7E-06 | yes | up | 103.908 | 49.314 | 117.768 | 427.344 | 256.465 | 296.642 |
| YNL283C | 4.047 | 2.017 | 6.23E-10 | 1.54E-08 | yes | up | 3.301 | 7.289 | 6.705 | 24.302 | 20.007 | 34.162 |
| YOL032W | 2.317 | 1.212 | 9.43E-05 | 0.000888 | yes | up | 6.776 | 9.974 | 7.487 | 15.871 | 17.736 | 26.375 |
| YHR094C | 2.634 | 1.397 | 2.31E-06 | 3.15E-05 | yes | up | 532.555 | 629.684 | 269.845 | 1448.714 | 1456.721 | 1148.169 |
| YBL070C | 2.364 | 1.241 | 0.000272 | 0.0022 | yes | up | 61.84 | 28.037 | 39.184 | 102.927 | 103.83 | 104.401 |
| YML123C | 2.583 | 1.369 | 0.03021 | 0.1052 | yes | up | 7.569 | 0 | 1.79 | 70.606 | 114.524 | 85.131 |
| YBL069W | 2.597 | 1.377 | 1.54E-09 | 3.64E-08 | yes | up | 70.841 | 47.984 | 63.908 | 166.821 | 191.429 | 143.256 |
| YLL026W | 4.039 | 2.014 | 1.64E-07 | 2.83E-06 | yes | up | 28.043 | 9.629 | 36.058 | 87.336 | 122.673 | 148.448 |
| YLR110C | 2.497 | 1.32 | 1.26E-05 | 0.000147 | yes | up | 10188.72 | 23790.78 | 12900.01 | 40995.53 | 38376.83 | 42694.59 |
| YFL020C | 8.369 | 3.065 | 2.15E-06 | 2.96E-05 | yes | up | 0 | 0 | 0 | 6.767 | 10.212 | 17.723 |
| YOR104W | 2.272 | 1.184 | 0.000598 | 0.004383 | yes | up | 18.312 | 9.063 | 9.455 | 20.311 | 36.522 | 35.089 |
| YKL096W | 2.208 | 1.143 | 7.22E-05 | 0.000704 | yes | up | 22.452 | 45.83 | 26.406 | 63.558 | 72.589 | 84.144 |
| YOR178C | 200.159 | 7.645 | 1.32E-53 | 5.34E-51 | yes | up | 0 | 0 | 0 | 15.693 | 12.871 | 14.343 |
| YDR054C | 2.154 | 1.107 | 0.000454 | 0.00345 | yes | up | 20.008 | 9.407 | 15.478 | 27.508 | 33.588 | 43.477 |
| YER188W | 2.152 | 1.106 | 0.000811 | 0.00566 | yes | up | 13.178 | 24.035 | 18.435 | 56.782 | 40.26 | 29.714 |
| YKR013W | 2.526 | 1.337 | 8.22E-07 | 1.23E-05 | yes | up | 100.926 | 156.033 | 113.565 | 296.134 | 245.941 | 452.266 |
| YLR213C | 2.059 | 1.042 | 8.34E-07 | 1.25E-05 | yes | up | 7.496 | 8.053 | 7.289 | 14.983 | 16.06 | 17.947 |
| YLL028W | 2.338 | 1.225 | 0.04334 | 0.1385 | yes | up | 0.684 | 0 | 0 | 27.891 | 14.802 | 23.311 |
| YHL008C | 99.457 | 6.636 | 1.05E-35 | 1.45E-33 | yes | up | 0 | 0 | 0 | 12.422 | 6.398 | 10.515 |
| YDR247W | 94.878 | 6.568 | 2.03E-33 | 2.39E-31 | yes | up | 0 | 0 | 0 | 22.329 | 7.619 | 19.066 |
| YMR215W | 3.055 | 1.611 | 4.99E-16 | 2.14E-14 | yes | up | 32.985 | 34.908 | 33.744 | 93.346 | 98.076 | 135.561 |
| YLR113W | 2.275 | 1.186 | 1.63E-08 | 3.39E-07 | yes | up | 153.71 | 112.185 | 118.293 | 252.924 | 350.945 | 314.099 |
| YLR176C | 2.445 | 1.29 | 4.55E-05 | 0.000466 | yes | up | 3.693 | 2.413 | 2.73 | 4.888 | 9.36 | 9.569 |
| YGR052W | 2.053 | 1.038 | 0.005495 | 0.02694 | yes | up | 3.137 | 1.909 | 2.799 | 7.636 | 5.442 | 4.622 |
| YKL167C | 19.253 | 4.267 | 7.33E-16 | 3.11E-14 | yes | up | 0 | 0.382 | 2.69 | 75.466 | 40.307 | 39.283 |
| YOR389W | 2.533 | 1.341 | 9.23E-11 | 2.41E-09 | yes | up | 64.712 | 47.59 | 54.157 | 161.522 | 139.821 | 140.569 |
| YER145C | 3.618 | 1.855 | 1.38E-12 | 4.51E-11 | yes | up | 804.045 | 1142.269 | 622.774 | 3887.999 | 3333.209 | 2724.703 |
| YBR244W | 3.618 | 1.855 | 6.29E-08 | 1.16E-06 | yes | up | 23.611 | 18.396 | 13.144 | 47.65 | 107.142 | 68.335 |
| YGR022C | 2.398 | 1.262 | 0.002515 | 0.01438 | yes | up | 11.938 | 25.685 | 22.292 | 51.781 | 41.31 | 63.622 |
| YLR217W | 2.321 | 1.215 | 0.000128 | 0.001133 | yes | up | 54.571 | 46.827 | 78.931 | 111.47 | 142.822 | 177.103 |
| YLR057W | 2.065 | 1.046 | 0.001158 | 0.007648 | yes | up | 1.696 | 1.478 | 2.71 | 3.337 | 4.741 | 5.151 |
| YGR225W | 2.259 | 1.176 | 0.03242 | 0.1113 | yes | up | 0.228 | 0.172 | 0.504 | 0.879 | 0.587 | 1.283 |
| YPR137C-B | 2.624 | 1.392 | 0.03387 | 0.1151 | yes | up | 0 | 0 | 0.049 | 0.178 | 0.066 | 0.112 |
| YHL047C | 2.478 | 1.309 | 2.42E-07 | 4.05E-06 | yes | up | 47.859 | 32.408 | 35.96 | 72.148 | 116.635 | 120.006 |
| YER045C | 2.495 | 1.319 | 7.15E-06 | 8.8E-05 | yes | up | 13.853 | 10.392 | 17.327 | 38.453 | 26.282 | 47.121 |
| YDR343C | 2.142 | 1.099 | 0.003586 | 0.0192 | yes | up | 236.434 | 856.505 | 498.825 | 1453.658 | 1419.754 | 841.094 |
| YOR071C | 2.045 | 1.032 | 3.5E-08 | 6.8E-07 | yes | up | 28.581 | 31.928 | 26.742 | 62.708 | 53.67 | 68.498 |
| YMR180C | 2.011 | 1.008 | 0.001465 | 0.009288 | yes | up | 5.946 | 10.971 | 5.4 | 16.273 | 14.868 | 16.033 |
| YLR214W | 3.002 | 1.586 | 1.47E-12 | 4.77E-11 | yes | up | 15.458 | 11.045 | 12.55 | 36.387 | 49.856 | 38.723 |
| YLR257W | 2.717 | 1.442 | 2.29E-09 | 5.3E-08 | yes | up | 122.603 | 78.792 | 102.558 | 237.969 | 362.377 | 278.481 |
| YPR175W | 2.167 | 1.116 | 3.69E-05 | 0.000388 | yes | up | 10.15 | 7.868 | 8.772 | 13.628 | 22.117 | 26.538 |
| YER103W | 3.967 | 1.988 | 1.69E-13 | 6.04E-12 | yes | up | 60.554 | 42.431 | 56.768 | 147.969 | 285.681 | 265.93 |
| YBR296C | 7.89 | 2.98 | 1.15E-22 | 7.65E-21 | yes | up | 8.819 | 5.147 | 5.133 | 38.724 | 73.772 | 64.538 |
| YKL105C | 2.256 | 1.174 | 6.65E-05 | 0.000656 | yes | up | 8.737 | 14.419 | 11.176 | 37.331 | 24.105 | 20.542 |
| YGR250C | 3.961 | 1.986 | 6.94E-08 | 1.26E-06 | yes | up | 9.065 | 4.26 | 11.759 | 26.451 | 57.153 | 34.04 |
| YDR423C | 3.444 | 1.784 | 2.76E-23 | 1.92E-21 | yes | up | 15.029 | 13.963 | 15.685 | 53.323 | 55.904 | 51.987 |
| YDR422C | 2.317 | 1.212 | 0.04491 | 0.142 | yes | up | 0 | 0 | 0 | 0.093 | 0 | 0.794 |
| YLL052C | 3.218 | 1.686 | 0.000143 | 0.001252 | yes | up | 8.198 | 27.939 | 14.924 | 32.125 | 75.873 | 88.725 |
| YPL089C | 2.154 | 1.107 | 1.95E-06 | 2.72E-05 | yes | up | 28.873 | 26.276 | 32.102 | 46.201 | 77.387 | 75.491 |
| YNL006W | 2.099 | 1.07 | 1.65E-08 | 3.4E-07 | yes | up | 185.51 | 227.191 | 180.322 | 348.467 | 451.566 | 490.867 |
| YDR420W | 2.215 | 1.147 | 0.008825 | 0.04036 | yes | up | 1.751 | 0.616 | 2.383 | 4.038 | 2.243 | 5.935 |
| YKR010C | 2.227 | 1.155 | 0.000192 | 0.001621 | yes | up | 1.942 | 1.391 | 2.571 | 3.916 | 5.423 | 4.978 |
| YCL059C | 2.139 | 1.097 | 0.002424 | 0.01394 | yes | up | 46.273 | 13.865 | 31.381 | 63.268 | 71.33 | 80.724 |
| YML046W | 2.053 | 1.038 | 0.01607 | 0.0651 | yes | up | 4.697 | 1.551 | 2.097 | 3.486 | 8.877 | 7.482 |
| YDL174C | 5.315 | 2.41 | 3.21E-22 | 2.01E-20 | yes | up | 125.458 | 226.181 | 171.075 | 1054.214 | 833.155 | 1112.683 |
| YBL042C | 2.723 | 1.445 | 1.08E-05 | 0.000128 | yes | up | 130.775 | 52.971 | 61.208 | 238.203 | 218.874 | 280.904 |
| YLR300W | 2.85 | 1.511 | 1.89E-11 | 5.35E-10 | yes | up | 293.887 | 197.614 | 236.902 | 601.297 | 873.794 | 731.633 |
| YEL059C-A | 3.487 | 1.802 | 0.000438 | 0.003338 | yes | up | 45.516 | 56.653 | 15.171 | 174.607 | 144.374 | 190.062 |
| YMR011W | 3.027 | 1.598 | 3.15E-06 | 4.18E-05 | yes | up | 258.138 | 354.977 | 241.313 | 1450.144 | 874.882 | 517.721 |
| YMR103C | 2.809 | 1.49 | 0.000168 | 0.001439 | yes | up | 16.744 | 41.68 | 37.275 | 60.147 | 124.651 | 113.441 |
| YKL131W | 2.003 | 1.002 | 0.000806 | 0.005649 | yes | up | 10.597 | 14.234 | 14.963 | 30.181 | 27.029 | 25.866 |
| YER053C | 2.152 | 1.106 | 0.000486 | 0.003651 | yes | up | 122.357 | 142.771 | 199.528 | 509.147 | 308.991 | 242.263 |
| YJR160C | 3.56 | 1.832 | 0.000257 | 0.002088 | yes | up | 1.058 | 0.394 | 1.78 | 6.403 | 2.11 | 7.136 |
| YLL029W | 2.118 | 1.083 | 3.02E-09 | 6.81E-08 | yes | up | 59.067 | 54.141 | 69.427 | 124.957 | 148.831 | 129.728 |
| YPR119W | 2.592 | 1.374 | 0.005681 | 0.02773 | yes | up | 0.328 | 0.443 | 0.316 | 1.131 | 1.23 | 1.171 |
| YDL062W | 2.882 | 1.527 | 0.00299 | 0.01649 | yes | up | 5.609 | 1.194 | 6.438 | 16.123 | 11.215 | 21.357 |
| YDR306C | 16.359 | 4.032 | 1.09E-10 | 2.83E-09 | yes | up | 0 | 0 | 0 | 0.738 | 4.117 | 2.769 |
| YDL179W | 2.051 | 1.036 | 0.00137 | 0.008764 | yes | up | 2.836 | 3.62 | 3.966 | 6.982 | 7.117 | 8.754 |
| YLL053C | 2.352 | 1.234 | 0.000583 | 0.004296 | yes | up | 31.672 | 66.171 | 36.424 | 64.839 | 145.064 | 132.232 |
| YPR098C | 0.197 | -2.346 | 6.77E-24 | 5.03E-22 | yes | down | 273.979 | 280.113 | 214.056 | 44.612 | 59.869 | 38.682 |
| YIL014C-A | 0.499 | -1.003 | 0.004659 | 0.02362 | yes | down | 88.87 | 132.514 | 73.314 | 35.378 | 54.986 | 44.994 |
| YLL027W | 0.45 | -1.151 | 1.3E-08 | 2.74E-07 | yes | down | 305.961 | 404.402 | 377.803 | 180.645 | 161.163 | 143.572 |
| YBL095W | 0.481 | -1.056 | 0.0007 | 0.005038 | yes | down | 110.657 | 239.344 | 157.219 | 96.552 | 80.387 | 57.26 |
| YCR061W | 0.21 | -2.253 | 2.28E-12 | 7.28E-11 | yes | down | 29.365 | 45.78 | 31.549 | 7.393 | 9.227 | 3.878 |
| YPL171C | 0.453 | -1.143 | 0.000228 | 0.001892 | yes | down | 64.767 | 161.561 | 94.231 | 49.444 | 50.992 | 39.283 |
| YHR051W | 0.016 | -5.941 | 1.46E-25 | 1.23E-23 | yes | down | 86.125 | 23.432 | 89.652 | 0 | 0 | 0 |
| YDL222C | 0.46 | -1.121 | 0.001527 | 0.009592 | yes | down | 12.567 | 29.133 | 17.594 | 9.328 | 6.076 | 10.19 |
| YKL007W | 0.462 | -1.113 | 6.96E-07 | 1.07E-05 | yes | down | 33.724 | 34.046 | 36.662 | 17.095 | 17.158 | 13.549 |
| YDL003W | 0.388 | -1.366 | 2.24E-05 | 0.000251 | yes | down | 3.593 | 4.544 | 3.452 | 1.626 | 1.221 | 1.415 |
| YNR016C | 0.305 | -1.713 | 4.72E-05 | 0.000477 | yes | down | 20.884 | 5.836 | 10.335 | 2.234 | 2.47 | 5.385 |
| YGR055W | 0.366 | -1.449 | 3.27E-09 | 7.32E-08 | yes | down | 174.001 | 111.73 | 117.363 | 38.612 | 52.156 | 55.804 |
| YMR315W | 0.004 | -8.024 | 6.29E-59 | 3.56E-56 | yes | down | 37.81 | 69.914 | 49.865 | 0 | 0 | 0 |
| YLR070C | 0.416 | -1.265 | 0.000128 | 0.001132 | yes | down | 15.385 | 37.001 | 20.373 | 10.487 | 9.634 | 8.53 |
| YER061C | 0.481 | -1.056 | 1.25E-06 | 1.79E-05 | yes | down | 56.395 | 61.96 | 55.363 | 22.498 | 25.411 | 35.862 |
| YDR072C | 0.469 | -1.092 | 1.31E-06 | 1.88E-05 | yes | down | 255.257 | 197.713 | 155.657 | 90.402 | 104.275 | 90.934 |
| YER011W | 0.424 | -1.239 | 1.73E-08 | 3.56E-07 | yes | down | 57.982 | 111.705 | 58.044 | 21.348 | 20.575 | 19.748 |
| YBR234C | 0.32 | -1.644 | 1.04E-24 | 8.03E-23 | yes | down | 97.224 | 112.309 | 101.292 | 30.928 | 33.399 | 35.415 |
| YMR008C | 0.379 | -1.398 | 4.05E-12 | 1.22E-10 | yes | down | 428.592 | 523.36 | 486.275 | 211.91 | 171.668 | 158.628 |
| YER119C-A | 0.388 | -1.367 | 0.02105 | 0.07905 | yes | down | 4.742 | 18.002 | 8.268 | 2.804 | 2.158 | 2.972 |
| YLR178C | 0.211 | -2.246 | 1.85E-15 | 7.48E-14 | yes | down | 159.757 | 346.789 | 247.415 | 49.996 | 53.235 | 44.719 |
| YOR184W | 0.179 | -2.481 | 1.88E-48 | 6.63E-46 | yes | down | 187.909 | 202.256 | 193.545 | 31.059 | 32.679 | 40.006 |
| YIL034C | 0.474 | -1.076 | 3.04E-07 | 5.06E-06 | yes | down | 115.919 | 170.759 | 128.806 | 69.147 | 62.387 | 63.917 |
| YGR088W | 0.073 | -3.782 | 3.72E-43 | 8.41E-41 | yes | down | 34.518 | 41.286 | 62.741 | 2.813 | 3.284 | 3.003 |
| YDL243C | 0.193 | -2.377 | 1.62E-17 | 7.81E-16 | yes | down | 20.245 | 24.836 | 16.358 | 3.776 | 4.079 | 3.207 |
| YDR379C-A | 0.453 | -1.144 | 0.006057 | 0.02919 | yes | down | 168.457 | 200.151 | 134.176 | 60.118 | 83.198 | 48.332 |
| YBR256C | 0.4 | -1.321 | 1.48E-16 | 6.72E-15 | yes | down | 1004.411 | 1143.057 | 964.716 | 403.36 | 454.245 | 386.944 |
| YJL060W | 0.212 | -2.241 | 2.91E-21 | 1.75E-19 | yes | down | 31.855 | 22.693 | 29.076 | 5.814 | 5.726 | 5.629 |
| YDR223W | 0.015 | -6.083 | 2.06E-27 | 1.84E-25 | yes | down | 4.304 | 15.687 | 10.711 | 0 | 0 | 0 |
| YNL104C | 0.214 | -2.225 | 1.33E-43 | 3.28E-41 | yes | down | 1312.251 | 1394.998 | 1382.741 | 307.369 | 265.929 | 299.197 |
| YOR173W | 0.238 | -2.068 | 2.61E-19 | 1.39E-17 | yes | down | 110.757 | 162.694 | 157.684 | 30.611 | 40.591 | 28.879 |
| SRG1 | 0.399 | -1.327 | 5.34E-07 | 8.43E-06 | yes | down | 88.314 | 140.432 | 133.464 | 49.51 | 52.26 | 37.267 |
| YGL104C | 0.241 | -2.05 | 9.93E-15 | 3.87E-13 | yes | down | 11.016 | 13.815 | 10.256 | 2.29 | 3.275 | 2.575 |
| YBR092C | 0.286 | -1.807 | 7.26E-06 | 8.92E-05 | yes | down | 326.216 | 123.981 | 165.546 | 27.779 | 42.74 | 87.157 |
| YPR169W-A | 0.256 | -1.968 | 3.88E-05 | 0.000406 | yes | down | 238.039 | 282.07 | 259.075 | 21.049 | 78.419 | 49.055 |
| YKL107W | 0.436 | -1.197 | 0.000218 | 0.001823 | yes | down | 14.163 | 20.932 | 14.637 | 9.197 | 5.196 | 6.179 |
| YOL083W | 0.428 | -1.223 | 6.36E-08 | 1.17E-06 | yes | down | 16.807 | 15.034 | 16.279 | 6.842 | 6.18 | 7.472 |
| YLR307C-A | 0.35 | -1.515 | 0.001 | 0.006736 | yes | down | 155.069 | 91.672 | 166.229 | 20.778 | 67.696 | 28.411 |
| YGL125W | 0.089 | -3.496 | 5.27E-19 | 2.78E-17 | yes | down | 31.763 | 27.101 | 74.916 | 1.879 | 3.095 | 4.408 |
| YGR019W | 0.326 | -1.617 | 5.22E-09 | 1.14E-07 | yes | down | 158.972 | 265.817 | 204.859 | 82.551 | 47.632 | 66.208 |
| YBL097W | 0.262 | -1.933 | 7.42E-10 | 1.83E-08 | yes | down | 3.32 | 5.504 | 4.609 | 1.215 | 1.013 | 1.038 |
| YDR098C-B | 0.461 | -1.116 | 4.93E-05 | 0.000498 | yes | down | 9.338 | 17.263 | 10.414 | 5.318 | 6.719 | 4.296 |
| YNL286W | 0.043 | -4.535 | 3.96E-14 | 1.47E-12 | yes | down | 4.14 | 3.657 | 6.646 | 0 | 0 | 0 |
| LSR1 | 0.426 | -1.232 | 0.00024 | 0.001967 | yes | down | 213.088 | 604.639 | 430.387 | 207.639 | 152.248 | 142.412 |
| NME1 | 0.125 | -2.996 | 4.3E-06 | 5.57E-05 | yes | down | 5.034 | 14.813 | 36.751 | 0 | 0 | 0 |
| Q0045 | 0.296 | -1.755 | 0.007056 | 0.03343 | yes | down | 0.073 | 0.197 | 1.157 | 0 | 0 | 0.041 |
| RDN18-1 | 0.322 | -1.633 | 0.000552 | 0.004094 | yes | down | 613.355 | 2516.778 | 5636.776 | 759.94 | 925.269 | 671.218 |
| RDN18-2 | 0.322 | -1.633 | 0.000552 | 0.004094 | yes | down | 613.355 | 2516.778 | 5636.776 | 759.94 | 925.269 | 671.218 |
| RDN25-1 | 0.297 | -1.751 | 9.82E-05 | 0.000915 | yes | down | 905.81 | 3819.39 | 6831.243 | 936.435 | 1092.328 | 874.126 |
| RDN25-2 | 0.297 | -1.751 | 9.82E-05 | 0.000915 | yes | down | 905.81 | 3819.39 | 6831.243 | 936.435 | 1092.328 | 874.126 |
| RDN58-1 | 0.222 | -2.171 | 0.000333 | 0.00265 | yes | down | 486.301 | 3934.087 | 16931.48 | 816.376 | 568.939 | 714.46 |
| RDN58-2 | 0.222 | -2.171 | 0.000333 | 0.00265 | yes | down | 486.301 | 3934.087 | 16931.48 | 816.376 | 568.939 | 714.46 |
| SCR1 | 0.247 | -2.016 | 0.001676 | 0.01034 | yes | down | 26.465 | 182.408 | 583.65 | 27.087 | 24.994 | 21.387 |
| snR17a | 0.281 | -1.829 | 0.005243 | 0.02595 | yes | down | 26.994 | 224.285 | 738.446 | 36.294 | 30.758 | 7.37 |
| snR19 | 0.426 | -1.23 | 4.25E-05 | 0.000438 | yes | down | 136.876 | 304.764 | 261.231 | 97.973 | 108.325 | 80.143 |
| snR190 | 0.262 | -1.933 | 0.001526 | 0.009592 | yes | down | 96.731 | 484.942 | 1233.137 | 97.73 | 39.948 | 89.234 |
| tR(UCU)M2 | 0.476 | -1.071 | 4.68E-08 | 8.7E-07 | yes | down | 14.445 | 20.403 | 15.824 | 7.636 | 8.3 | 8.286 |
| YAL012W | 0.159 | -2.654 | 6.55E-45 | 1.85E-42 | yes | down | 687.488 | 626.482 | 492.04 | 91.337 | 92.104 | 98.039 |
| YAL028W | 0.326 | -1.617 | 9.33E-08 | 1.66E-06 | yes | down | 5.235 | 8.755 | 7.388 | 2.028 | 2.035 | 2.565 |
| YAL044C | 0.23 | -2.118 | 6.42E-13 | 2.2E-11 | yes | down | 672.486 | 1550.698 | 929.143 | 238.212 | 217.152 | 212.04 |
| YAL061W | 0.25 | -2.002 | 2.66E-12 | 8.36E-11 | yes | down | 61.256 | 138.585 | 115.939 | 22.703 | 25.856 | 26.029 |
| YAL064W | 0.446 | -1.165 | 6.27E-06 | 7.85E-05 | yes | down | 1.714 | 1.576 | 1.83 | 0.776 | 0.634 | 0.967 |
| YAR010C | 0.378 | -1.402 | 4.11E-08 | 7.7E-07 | yes | down | 41.494 | 30.143 | 24.082 | 10.973 | 12.587 | 12.114 |
| YAR015W | 0.337 | -1.57 | 6.77E-07 | 1.05E-05 | yes | down | 346.598 | 693.873 | 528.039 | 113.704 | 143.73 | 244.421 |
| YAR027W | 0.447 | -1.161 | 3.23E-08 | 6.3E-07 | yes | down | 84.137 | 92.09 | 87.97 | 30.583 | 47.632 | 39.527 |
| YAR073W | 0.458 | -1.127 | 9.52E-05 | 0.000893 | yes | down | 5.937 | 7.425 | 6.508 | 2.542 | 3.104 | 3.268 |
| YAR075W | 0.346 | -1.532 | 0.006842 | 0.03261 | yes | down | 3.976 | 8.718 | 5.944 | 0.981 | 2.517 | 1.038 |
| YBL001C | 0.186 | -2.425 | 3.66E-26 | 3.13E-24 | yes | down | 1248.086 | 1599.987 | 1090.09 | 235.95 | 222.196 | 211.072 |
| YBL015W | 0.359 | -1.478 | 4.57E-05 | 0.000467 | yes | down | 143.77 | 362.648 | 300.711 | 123.677 | 88.716 | 53.636 |
| YBL029W | 0.5 | -1.001 | 9.27E-07 | 1.37E-05 | yes | down | 30.332 | 39.341 | 35.425 | 16.151 | 16.562 | 19.87 |
| YBL080C | 0.438 | -1.19 | 0.0117 | 0.05087 | yes | down | 2.325 | 1.219 | 1.582 | 0.355 | 0.927 | 0.733 |
| YBL085W | 0.453 | -1.143 | 3.16E-05 | 0.00034 | yes | down | 3.101 | 4.925 | 4.342 | 1.72 | 1.694 | 2.077 |
| YBR005W | 0.395 | -1.34 | 4.45E-09 | 9.83E-08 | yes | down | 1433.641 | 2061.917 | 1262.075 | 540.356 | 723.212 | 580.182 |
| YBR007C | 0.461 | -1.117 | 1.86E-08 | 3.77E-07 | yes | down | 26.647 | 32.482 | 26.169 | 14.459 | 13.013 | 11.859 |
| YBR011C | 0.275 | -1.864 | 3.49E-15 | 1.39E-13 | yes | down | 788.505 | 1085.198 | 785.838 | 227.912 | 184.936 | 295.288 |
| YBR016W | 0.455 | -1.136 | 0.000143 | 0.001252 | yes | down | 520.244 | 908.824 | 602.006 | 345.728 | 206.969 | 310.18 |
| YBR026C | 0.12 | -3.055 | 5.12E-18 | 2.58E-16 | yes | down | 36.715 | 15.391 | 21.955 | 2.066 | 2.839 | 2.748 |
| YBR038W | 0.463 | -1.11 | 0.000224 | 0.001862 | yes | down | 4.35 | 4.26 | 2.73 | 1.851 | 1.874 | 1.405 |
| YBR043C | 0.325 | -1.623 | 3.67E-11 | 9.96E-10 | yes | down | 119.475 | 68.277 | 87.011 | 28.489 | 30.162 | 29.317 |
| YBR044C | 0.446 | -1.166 | 3.07E-08 | 6.05E-07 | yes | down | 14.956 | 20.575 | 16.338 | 7.487 | 7.865 | 7.716 |
| YBR053C | 0.317 | -1.656 | 1.82E-17 | 8.73E-16 | yes | down | 32.602 | 37.05 | 33.655 | 11.076 | 10.931 | 10.475 |
| YBR056W | 0.266 | -1.912 | 9.82E-10 | 2.39E-08 | yes | down | 25.79 | 12.018 | 18.731 | 4.879 | 4.618 | 4.672 |
| YBR056W-A | 0.244 | -2.036 | 5.84E-09 | 1.27E-07 | yes | down | 3252.475 | 5622.175 | 3440.109 | 473.947 | 1148.061 | 966.322 |
| YBR068C | 0.297 | -1.75 | 4.35E-20 | 2.51E-18 | yes | down | 2448.284 | 2215.573 | 1910.543 | 535.692 | 669.078 | 751.157 |
| YBR072W | 0.222 | -2.173 | 2.13E-22 | 1.38E-20 | yes | down | 1465.241 | 2311.419 | 2272.354 | 426.727 | 465.97 | 401.409 |
| YBR104W | 0.356 | -1.489 | 1.05E-12 | 3.52E-11 | yes | down | 78.364 | 59.091 | 65.125 | 23.834 | 25.837 | 21.978 |
| YBR111C | 0.322 | -1.635 | 1.88E-13 | 6.64E-12 | yes | down | 179.199 | 231.943 | 277.786 | 69.989 | 80.321 | 66.89 |
| YBR116C | 0.214 | -2.224 | 7.04E-07 | 1.08E-05 | yes | down | 17.473 | 14 | 11.492 | 1.355 | 3.861 | 2.117 |
| YBR117C | 0.078 | -3.685 | 2.41E-52 | 9.07E-50 | yes | down | 35.667 | 35.696 | 26.92 | 2.215 | 2.972 | 1.954 |
| YBR138C | 0.315 | -1.666 | 1.95E-05 | 0.00022 | yes | down | 2.873 | 3.608 | 2.482 | 1.084 | 0.795 | 0.641 |
| YBR145W | 0.449 | -1.155 | 9.51E-06 | 0.000114 | yes | down | 86.973 | 166.622 | 106.454 | 45.799 | 52.043 | 61.016 |
| YBR149W | 0.339 | -1.559 | 2.18E-16 | 9.62E-15 | yes | down | 102.914 | 98.075 | 118.471 | 36.34 | 32.66 | 38.886 |
| YBR174C | 0.452 | -1.147 | 0.01241 | 0.05323 | yes | down | 36.177 | 115.559 | 58.548 | 31.405 | 23.575 | 25.378 |
| YBR214W | 0.414 | -1.274 | 7.09E-05 | 0.000695 | yes | down | 47.486 | 130.187 | 82.116 | 37.462 | 35.367 | 29.582 |
| YBR218C | 0.402 | -1.314 | 1.57E-06 | 2.22E-05 | yes | down | 156.51 | 92.878 | 154.628 | 57.081 | 41.244 | 61.545 |
| YBR230C | 0.422 | -1.245 | 1.74E-08 | 3.56E-07 | yes | down | 585.695 | 800.726 | 725.471 | 321.314 | 283.779 | 249.582 |
| YBR230W-A | 0.464 | -1.108 | 0.02195 | 0.0816 | yes | down | 457.684 | 611.35 | 509.11 | 259.906 | 252.017 | 70.921 |
| YBR240C | 0.313 | -1.676 | 2.57E-13 | 8.94E-12 | yes | down | 121.308 | 169.836 | 201.516 | 47.762 | 47.661 | 56.272 |
| YBR241C | 0.402 | -1.313 | 0.000307 | 0.002464 | yes | down | 75.373 | 253.097 | 120.983 | 63.072 | 61.223 | 43.752 |
| YBR248C | 0.441 | -1.18 | 2.58E-08 | 5.14E-07 | yes | down | 84.256 | 89.135 | 111.103 | 44.397 | 35.414 | 45.594 |
| YBR284W | 0.427 | -1.229 | 8.2E-05 | 0.000788 | yes | down | 5.545 | 12.301 | 6.982 | 3.178 | 3.7 | 3.329 |
| YBR285W | 0.304 | -1.718 | 0.000509 | 0.003811 | yes | down | 10.25 | 22.324 | 10.167 | 3.019 | 4.145 | 2.871 |
| YBR286W | 0.352 | -1.505 | 8.86E-23 | 5.96E-21 | yes | down | 447.734 | 488.193 | 398.215 | 152.25 | 162.8 | 158.281 |
| YBR301W | 0.228 | -2.136 | 7.77E-06 | 9.5E-05 | yes | down | 27.888 | 28.37 | 20.887 | 6.057 | 2.915 | 3.746 |
| YCL007C | 0.238 | -2.072 | 1.68E-07 | 2.88E-06 | yes | down | 54.927 | 99.823 | 49.38 | 17.88 | 9.597 | 12.623 |
| YCL009C | 0.358 | -1.482 | 1.03E-07 | 1.83E-06 | yes | down | 1954.177 | 4056.972 | 2259.843 | 1048.354 | 914.395 | 878.717 |
| YCL014W | 0.427 | -1.228 | 5.8E-07 | 9.07E-06 | yes | down | 2.718 | 3.694 | 2.611 | 1.131 | 1.353 | 1.344 |
| YCL030C | 0.332 | -1.591 | 2.86E-09 | 6.5E-08 | yes | down | 235.358 | 277.847 | 413.802 | 71.101 | 56.983 | 111.476 |
| YCL033C | 0.292 | -1.778 | 1.76E-08 | 3.59E-07 | yes | down | 237.373 | 585.492 | 333.921 | 101.861 | 120.155 | 89.437 |
| YCL064C | 0.406 | -1.302 | 2.09E-07 | 3.52E-06 | yes | down | 214.2 | 151.908 | 198.737 | 93.318 | 65.529 | 65.627 |
| YCR004C | 0.412 | -1.278 | 3.33E-12 | 1.03E-10 | yes | down | 88.095 | 89.344 | 82.758 | 31.134 | 40.875 | 35.16 |
| YCR020C | 0.335 | -1.579 | 3.43E-12 | 1.06E-10 | yes | down | 68.524 | 62.625 | 73.353 | 17.507 | 23.556 | 26.141 |
| YCR069W | 0.393 | -1.346 | 1.66E-06 | 2.33E-05 | yes | down | 66.463 | 118.231 | 104.091 | 45.453 | 33.947 | 29.918 |
| YCR088W | 0.416 | -1.264 | 1.07E-11 | 3.11E-10 | yes | down | 38.384 | 52.109 | 46.453 | 18.684 | 18.72 | 19.748 |
| YCR104W | 0.361 | -1.469 | 0.02507 | 0.09035 | yes | down | 4.624 | 3.768 | 2.146 | 0.458 | 0 | 0.489 |
| YDL029W | 0.442 | -1.177 | 6.98E-08 | 1.26E-06 | yes | down | 39.679 | 29.601 | 39.451 | 13.712 | 18.322 | 16.277 |
| YDL066W | 0.38 | -1.396 | 4.66E-07 | 7.49E-06 | yes | down | 65.342 | 46.162 | 52.021 | 26.377 | 15.881 | 17.947 |
| YDL072C | 0.495 | -1.015 | 0.000851 | 0.005883 | yes | down | 183.331 | 423.844 | 259.688 | 105.778 | 167.135 | 143.012 |
| YDL085W | 0.205 | -2.287 | 5.9E-20 | 3.3E-18 | yes | down | 22.498 | 21.856 | 20.245 | 5.3 | 3.606 | 3.726 |
| YDL104C | 0.027 | -5.187 | 5.37E-19 | 2.81E-17 | yes | down | 8.481 | 3.411 | 4.144 | 0 | 0 | 0 |
| YDL126C | 0.286 | -1.804 | 6.26E-31 | 6.31E-29 | yes | down | 222.618 | 202.133 | 221.602 | 61.063 | 60.059 | 65.342 |
| YDL130W-A | 0.107 | -3.229 | 1.06E-09 | 2.55E-08 | yes | down | 342.905 | 348.352 | 361.445 | 35.733 | 19.77 | 0 |
| YDL170W | 0.489 | -1.032 | 0.000418 | 0.003218 | yes | down | 168.84 | 137.391 | 202.831 | 112.769 | 61.138 | 68.793 |
| YDL181W | 0.488 | -1.034 | 0.000255 | 0.002077 | yes | down | 855.598 | 1417.715 | 1159.507 | 521.522 | 596.65 | 426.42 |
| YDL204W | 0.212 | -2.238 | 3.22E-10 | 8.2E-09 | yes | down | 8.609 | 7.216 | 8.713 | 1.495 | 0.965 | 2.168 |
| YDL207W | 0.461 | -1.116 | 2.28E-05 | 0.000254 | yes | down | 9.831 | 14.222 | 11.63 | 4.982 | 6.795 | 4.479 |
| YDL210W | 0.308 | -1.698 | 7E-18 | 3.5E-16 | yes | down | 695.248 | 921.346 | 736.35 | 263.598 | 246.216 | 208.212 |
| YDL218W | 0.186 | -2.423 | 0.000222 | 0.001843 | yes | down | 0.894 | 1.33 | 0.623 | 0 | 0 | 0 |
| YDL221W | 0.481 | -1.055 | 0.01816 | 0.07103 | yes | down | 12.539 | 36.755 | 12.214 | 10.59 | 7.571 | 8.021 |
| YDL238C | 0.348 | -1.524 | 3.33E-07 | 5.52E-06 | yes | down | 12.849 | 25.993 | 19.345 | 7.216 | 6.719 | 5.334 |
| YDR019C | 0.255 | -1.97 | 4.37E-17 | 2.06E-15 | yes | down | 61.967 | 93.149 | 84.361 | 16.207 | 19.098 | 24.309 |
| YDR024W | 0.404 | -1.307 | 0.04602 | 0.1443 | yes | down | 2.745 | 4.113 | 0.801 | 0 | 0.795 | 0.489 |
| YDR030C | 0.5 | -1.001 | 0.000146 | 0.001266 | yes | down | 6.876 | 9.752 | 8.129 | 3.542 | 4.817 | 3.909 |
| YDR031W | 0.432 | -1.211 | 0.001633 | 0.01013 | yes | down | 107.848 | 303.606 | 165.576 | 92.944 | 76.583 | 53.707 |
| YDR032C | 0.123 | -3.025 | 3.37E-47 | 1.06E-44 | yes | down | 362.53 | 336.285 | 254.525 | 37.583 | 39.228 | 34.621 |
| YDR070C | 0.085 | -3.558 | 6.8E-29 | 6.4E-27 | yes | down | 395.443 | 374.973 | 291.049 | 19.282 | 24.209 | 29.236 |
| YDR107C | 0.486 | -1.041 | 0.000101 | 0.000933 | yes | down | 5.435 | 6.354 | 4.549 | 2.393 | 2.404 | 3.064 |
| YDR119W-A | 0.193 | -2.371 | 7.08E-07 | 1.08E-05 | yes | down | 428.474 | 1397.362 | 1109.86 | 145.24 | 158.674 | 88.653 |
| YDR127W | 0.493 | -1.02 | 7.46E-07 | 1.12E-05 | yes | down | 132.124 | 99.798 | 131.644 | 57.67 | 54.39 | 69.139 |
| YDR154C | 0.344 | -1.54 | 1.47E-06 | 2.08E-05 | yes | down | 1860.318 | 4587.855 | 2776.371 | 740.433 | 1088.447 | 1082.165 |
| YDR155C | 0.315 | -1.665 | 3.89E-08 | 7.42E-07 | yes | down | 2430.136 | 5871.702 | 3527.723 | 937.678 | 1321.224 | 1235.733 |
| YDR158W | 0.311 | -1.685 | 1.95E-19 | 1.05E-17 | yes | down | 1420.974 | 1226.713 | 1330.612 | 340.933 | 407.53 | 488.515 |
| YDR204W | 0.411 | -1.282 | 1.68E-07 | 2.88E-06 | yes | down | 41.904 | 65.309 | 44.445 | 20.535 | 23.177 | 17.468 |
| YDR210C-C | 0.329 | -1.606 | 4.2E-07 | 6.79E-06 | yes | down | 27.158 | 35.893 | 18.781 | 6.337 | 12.142 | 7.095 |
| YDR226W | 0.476 | -1.072 | 2.07E-06 | 2.86E-05 | yes | down | 1028.386 | 1241.612 | 823.143 | 369.703 | 523.436 | 565.798 |
| YDR231C | 0.495 | -1.015 | 0.00363 | 0.01939 | yes | down | 57.8 | 136.96 | 65.866 | 41.434 | 51.825 | 29.246 |
| YDR236C | 0.473 | -1.079 | 1.61E-05 | 0.000183 | yes | down | 102.039 | 176.189 | 114.673 | 63.754 | 60.305 | 57.718 |
| YDR242W | 0.494 | -1.018 | 0.001659 | 0.01027 | yes | down | 41.503 | 21.265 | 43.189 | 11.562 | 22.165 | 17.936 |
| YDR320C-A | 0.391 | -1.354 | 1.54E-05 | 0.000177 | yes | down | 922.335 | 1177.744 | 771.894 | 243.259 | 391.157 | 375.543 |
| YDR340W | 0.339 | -1.561 | 0.000885 | 0.006074 | yes | down | 33.897 | 35.721 | 41.745 | 8.59 | 11.158 | 10.251 |
| YDR354W | 0.331 | -1.595 | 8.6E-16 | 3.63E-14 | yes | down | 116.594 | 133.229 | 109.896 | 44.65 | 38.178 | 34.946 |
| YDR365W-A | 0.483 | -1.051 | 4.7E-07 | 7.49E-06 | yes | down | 68.743 | 64.755 | 49.914 | 28.816 | 26.698 | 33.083 |
| YDR368W | 0.416 | -1.267 | 1.79E-08 | 3.64E-07 | yes | down | 61.521 | 43.158 | 48.994 | 18.469 | 22.591 | 22.548 |
| YDR377W | 0.319 | -1.65 | 2.07E-06 | 2.86E-05 | yes | down | 559.422 | 1260.451 | 865.927 | 274.291 | 318.001 | 164.939 |
| YDR408C | 0.326 | -1.616 | 1.87E-06 | 2.61E-05 | yes | down | 421.953 | 1315.344 | 697.819 | 217.509 | 250.087 | 266.714 |
| YDR411C | 0.426 | -1.232 | 2.44E-05 | 0.000269 | yes | down | 68.716 | 151.883 | 79.92 | 39.369 | 43.639 | 40.667 |
| YDR425W | 0.019 | -5.696 | 2.03E-23 | 1.48E-21 | yes | down | 4.113 | 9.026 | 2.641 | 0 | 0 | 0 |
| YDR436W | 0.476 | -1.071 | 0.009615 | 0.04344 | yes | down | 4.742 | 17.005 | 12.985 | 7.225 | 4.117 | 3.807 |
| YDR451C | 0.322 | -1.637 | 7.1E-08 | 1.28E-06 | yes | down | 16.962 | 31.029 | 17.673 | 5.244 | 7.656 | 7.177 |
| YDR487C | 0.498 | -1.005 | 7.43E-07 | 1.12E-05 | yes | down | 726.994 | 1001.924 | 694.901 | 384.013 | 445.405 | 368 |
| YDR502C | 0.331 | -1.595 | 1.95E-05 | 0.00022 | yes | down | 127.227 | 40.892 | 77.269 | 26.956 | 17.414 | 30.284 |
| YDR512C | 0.449 | -1.156 | 0.00015 | 0.001296 | yes | down | 63.408 | 111.705 | 59.992 | 34.293 | 40.137 | 26.365 |
| YEL020W-A | 0.192 | -2.382 | 0.000143 | 0.001252 | yes | down | 58.183 | 36.028 | 29.116 | 1.626 | 0 | 5.813 |
| YEL023C | 0.389 | -1.363 | 7.88E-06 | 9.61E-05 | yes | down | 3.019 | 3.423 | 2.769 | 1.15 | 1.202 | 1.11 |
| YEL035C | 0.481 | -1.055 | 0.004222 | 0.02174 | yes | down | 16.26 | 28.049 | 19.147 | 9.721 | 7.145 | 11.442 |
| YEL061C | 0.425 | -1.234 | 0.003034 | 0.01667 | yes | down | 16.032 | 54.375 | 33.171 | 16.983 | 16.884 | 5.975 |
| YEL066W | 0.455 | -1.137 | 6.42E-05 | 0.000637 | yes | down | 94.424 | 131.135 | 85.636 | 56.212 | 45.711 | 33.501 |
| YER032W | 0.392 | -1.351 | 0.000108 | 0.000991 | yes | down | 1.87 | 1.675 | 1.899 | 0.551 | 0.606 | 0.875 |
| YER034W | 0.38 | -1.396 | 1.03E-06 | 1.51E-05 | yes | down | 39.296 | 43.133 | 47.452 | 15.871 | 11.574 | 19.596 |
| YER039C | 0.443 | -1.175 | 3.46E-07 | 5.71E-06 | yes | down | 36.943 | 49.745 | 37.413 | 16.6 | 20.584 | 17.081 |
| YER042W | 0.308 | -1.697 | 1.09E-09 | 2.6E-08 | yes | down | 76.686 | 64.804 | 60.566 | 17.965 | 26.774 | 14.618 |
| YER044C | 0.467 | -1.098 | 0.001471 | 0.009316 | yes | down | 20.61 | 33.96 | 26.802 | 11.272 | 12.899 | 11.33 |
| YER055C | 0.329 | -1.606 | 3.17E-22 | 2.01E-20 | yes | down | 1139.763 | 1415.856 | 1378.637 | 423.25 | 413 | 453.569 |
| YER057C | 0.008 | -6.969 | 4.08E-40 | 7.69E-38 | yes | down | 112.928 | 234.689 | 144.53 | 0 | 0 | 0 |
| YER065C | 0.047 | -4.411 | 2.73E-72 | 2.57E-69 | yes | down | 513.021 | 305.478 | 490.31 | 18.637 | 18.067 | 19.606 |
| YER069W | 0.386 | -1.373 | 0.000238 | 0.001957 | yes | down | 123.488 | 63.93 | 94.082 | 27.218 | 18.436 | 56.761 |
| YER091C | 0.012 | -6.374 | 6.17E-61 | 4.36E-58 | yes | down | 293.513 | 108.627 | 285.827 | 2.084 | 0.975 | 2.178 |
| YER137C-A | 0.483 | -1.051 | 4.7E-07 | 7.49E-06 | yes | down | 68.743 | 64.755 | 49.914 | 28.816 | 26.698 | 33.083 |
| YER141W | 0.497 | -1.008 | 0.000711 | 0.005105 | yes | down | 33.013 | 52.614 | 45.602 | 27.741 | 21.313 | 14.272 |
| YER147C | 0.008 | -7.025 | 6.41E-42 | 1.29E-39 | yes | down | 12.649 | 8.041 | 12.432 | 0 | 0 | 0 |
| YER152C | 0.004 | -7.992 | 3.55E-54 | 1.54E-51 | yes | down | 28.745 | 87.596 | 37.809 | 0 | 0 | 0 |
| YER152W-A | 0.03 | -5.07 | 1.45E-17 | 7.12E-16 | yes | down | 12.986 | 37.715 | 9.712 | 0 | 0 | 0 |
| YER159C-A | 0.495 | -1.014 | 1.6E-05 | 0.000183 | yes | down | 109.307 | 138.979 | 82.244 | 48.762 | 50.254 | 63.917 |
| YER181C | 0.37 | -1.436 | 0.02737 | 0.09735 | yes | down | 6.894 | 13.298 | 10.681 | 0 | 0.861 | 3.95 |
| YER185W | 0.333 | -1.588 | 8.6E-10 | 2.11E-08 | yes | down | 18.449 | 28.505 | 22.41 | 6.711 | 7.836 | 7.818 |
| YFL014W | 0.139 | -2.849 | 1.29E-16 | 5.92E-15 | yes | down | 657.457 | 858.093 | 1320.87 | 61.034 | 152.267 | 118.48 |
| YFL021C-A | 0.168 | -2.575 | 7.5E-12 | 2.23E-10 | yes | down | 38.539 | 120.645 | 56.59 | 12.291 | 8.205 | 9.864 |
| YFL030W | 0.419 | -1.255 | 8.58E-05 | 0.000816 | yes | down | 236.297 | 578.461 | 370.039 | 189.141 | 167.012 | 116.271 |
| YFL031W | 0.485 | -1.045 | 0.001241 | 0.008057 | yes | down | 1865.425 | 5048.652 | 2895.158 | 1750.727 | 1579.441 | 1221.564 |
| YFL033C | 0.481 | -1.056 | 0.000237 | 0.001949 | yes | down | 47.905 | 130.729 | 69.298 | 40.743 | 41.358 | 28.818 |
| YFL044C | 0.455 | -1.137 | 1.3E-07 | 2.25E-06 | yes | down | 75.948 | 55.446 | 66.915 | 31.648 | 29.66 | 28.431 |
| YFL055W | 0.492 | -1.022 | 4.2E-05 | 0.000434 | yes | down | 10.077 | 12.67 | 9.524 | 5.804 | 4.401 | 5.507 |
| YFL059W | 0.088 | -3.512 | 1.1E-36 | 1.55E-34 | yes | down | 203.795 | 170.476 | 173.122 | 10.422 | 11.811 | 21.357 |
| YFL060C | 0.478 | -1.065 | 0.002646 | 0.01493 | yes | down | 35.521 | 18.925 | 18.543 | 7.58 | 13.032 | 12.867 |
| YFR030W | 0.138 | -2.861 | 1.58E-21 | 9.59E-20 | yes | down | 131.012 | 68.572 | 121.635 | 13.805 | 9.975 | 16.745 |
| YFR031C | 0.472 | -1.083 | 0.008434 | 0.03882 | yes | down | 0.73 | 0.936 | 0.979 | 0.243 | 0.539 | 0.397 |
| YFR044C | 0.157 | -2.668 | 3.61E-24 | 2.72E-22 | yes | down | 84.329 | 72.796 | 50.962 | 10.431 | 12.313 | 8.103 |
| YFR047C | 0.39 | -1.357 | 5.94E-09 | 1.29E-07 | yes | down | 105.741 | 152.893 | 95.803 | 38.284 | 52.573 | 45.513 |
| YGL009C | 0 | -12.49 | 8E-174 | 4.5E-170 | yes | down | 1088.119 | 531.794 | 1210.39 | 0 | 0 | 0 |
| YGL026C | 0.418 | -1.26 | 2.77E-10 | 7.09E-09 | yes | down | 688.427 | 827.581 | 668.673 | 351.43 | 290.375 | 267.426 |
| YGL113W | 0.433 | -1.207 | 1.97E-05 | 0.000222 | yes | down | 5.791 | 9.075 | 7.892 | 3.271 | 3.814 | 2.545 |
| YGL116W | 0.429 | -1.221 | 0.004557 | 0.02319 | yes | down | 1.404 | 1.096 | 1.721 | 0.467 | 0.596 | 0.601 |
| YGL121C | 0.296 | -1.754 | 1.75E-10 | 4.53E-09 | yes | down | 264.431 | 160.761 | 170.392 | 43.509 | 65.112 | 55.305 |
| YGL126W | 0.445 | -1.168 | 2.33E-05 | 0.000259 | yes | down | 422.528 | 826.904 | 476.8 | 284.376 | 251.345 | 211.072 |
| YGL161C | 0.19 | -2.397 | 1.31E-21 | 8.04E-20 | yes | down | 44.348 | 68.671 | 44.959 | 8.936 | 10.761 | 8.826 |
| YGL184C | 0.347 | -1.529 | 1.68E-11 | 4.8E-10 | yes | down | 46.747 | 39.254 | 58.133 | 15.628 | 18.587 | 15.279 |
| YGL187C | 0.495 | -1.015 | 1.12E-06 | 1.62E-05 | yes | down | 285.661 | 371.834 | 388.701 | 166.167 | 188.552 | 152.55 |
| YGL223C | 0.487 | -1.038 | 1.24E-06 | 1.79E-05 | yes | down | 31.207 | 26.276 | 24.665 | 13.721 | 14.243 | 12.073 |
| YGL234W | 0.369 | -1.437 | 2.95E-07 | 4.94E-06 | yes | down | 672.969 | 1314.95 | 1019.674 | 296.115 | 303.435 | 481.227 |
| YGL242C | 0.289 | -1.79 | 1.91E-10 | 4.92E-09 | yes | down | 30.359 | 31.768 | 28.73 | 7.206 | 9.634 | 8.032 |
| YGL259W | 0.359 | -1.477 | 0.006835 | 0.03261 | yes | down | 4.952 | 5.085 | 6.557 | 2.187 | 1.249 | 0.926 |
| YGR008C | 0.256 | -1.967 | 5.77E-07 | 9.06E-06 | yes | down | 301.611 | 716.787 | 548.076 | 81.111 | 160.103 | 93.224 |
| YGR032W | 0.474 | -1.077 | 0.000154 | 0.001325 | yes | down | 92.883 | 157.178 | 131.802 | 48.669 | 84.93 | 45.095 |
| YGR037C | 0.343 | -1.542 | 1.8E-07 | 3.05E-06 | yes | down | 337.743 | 470.438 | 392.904 | 121.181 | 145.661 | 104.157 |
| YGR043C | 0.267 | -1.906 | 2.7E-11 | 7.46E-10 | yes | down | 127.2 | 64.324 | 78.525 | 19.553 | 26.348 | 23.138 |
| YGR044C | 0.425 | -1.234 | 0.000399 | 0.003076 | yes | down | 14.172 | 9.136 | 12.145 | 5.711 | 3.17 | 5.365 |
| YGR066C | 0.317 | -1.658 | 2.49E-06 | 3.38E-05 | yes | down | 12.713 | 9.764 | 7.853 | 3.384 | 2.271 | 3.176 |
| YGR086C | 0.45 | -1.152 | 5.02E-06 | 6.37E-05 | yes | down | 89.426 | 128.931 | 138.616 | 52.856 | 41.433 | 63.866 |
| YGR087C | 0.351 | -1.509 | 6.42E-14 | 2.36E-12 | yes | down | 16.16 | 18.642 | 16.328 | 5.477 | 5.063 | 7.024 |
| YGR122W | 0.49 | -1.03 | 0.000882 | 0.00606 | yes | down | 5.891 | 5.307 | 6.873 | 3.215 | 2.574 | 2.83 |
| YGR133W | 0.018 | -5.76 | 3.38E-24 | 2.58E-22 | yes | down | 14.272 | 45.645 | 33.853 | 0 | 0 | 0 |
| YGR135W | 0.406 | -1.299 | 0.000393 | 0.003045 | yes | down | 50.121 | 20.711 | 47.343 | 11.777 | 12.625 | 20.939 |
| YGR144W | 0.054 | -4.222 | 2.08E-58 | 1.07E-55 | yes | down | 346.844 | 215.936 | 198.984 | 12.17 | 13.127 | 11.381 |
| YGR175C | 0.469 | -1.093 | 8.58E-06 | 0.000104 | yes | down | 1226.518 | 937.698 | 746.793 | 526.252 | 411.391 | 412.943 |
| YGR192C | 0.489 | -1.033 | 0.001047 | 0.00703 | yes | down | 10732.62 | 23851.8 | 11797.84 | 5922.822 | 6295.087 | 9752.882 |
| YGR213C | 0.013 | -6.278 | 5.03E-32 | 5.46E-30 | yes | down | 14.299 | 13.963 | 12.006 | 0 | 0 | 0 |
| YGR236C | 0.267 | -1.907 | 0.000389 | 0.003021 | yes | down | 25.371 | 125.533 | 76.32 | 11.749 | 17.934 | 11.421 |
| YGR244C | 0.418 | -1.26 | 6.44E-07 | 1E-05 | yes | down | 86.782 | 49.08 | 68.665 | 26.143 | 27.588 | 30.936 |
| YGR247W | 0.473 | -1.08 | 0.002177 | 0.01274 | yes | down | 10.159 | 13.409 | 11.828 | 3.851 | 4.59 | 7.533 |
| YGR248W | 0.471 | -1.087 | 7.99E-05 | 0.000771 | yes | down | 51.042 | 99.983 | 65.204 | 33.125 | 36.56 | 29.47 |
| YGR254W | 0.253 | -1.981 | 3.63E-12 | 1.11E-10 | yes | down | 6738.392 | 13981.84 | 7591.428 | 1799.639 | 2755.203 | 2255.254 |
| YGR256W | 0.21 | -2.251 | 2.93E-23 | 2.02E-21 | yes | down | 23.273 | 25.796 | 19.661 | 3.823 | 5.129 | 5.12 |
| YGR268C | 0.488 | -1.036 | 0.000236 | 0.001949 | yes | down | 118.755 | 236.819 | 168.721 | 94.982 | 79.943 | 71.847 |
| YGR286C | 0.326 | -1.619 | 1.26E-12 | 4.15E-11 | yes | down | 603.214 | 435.985 | 365.44 | 140.734 | 147.8 | 162.363 |
| YHL036W | 0.372 | -1.425 | 3.14E-08 | 6.13E-07 | yes | down | 102.477 | 198.525 | 136.816 | 54.417 | 58.544 | 46.154 |
| YHR001W-A | 0.401 | -1.318 | 0.000128 | 0.001131 | yes | down | 1079.93 | 1906.758 | 1356.483 | 502.212 | 710.445 | 334.652 |
| YHR008C | 0.315 | -1.666 | 7.12E-18 | 3.53E-16 | yes | down | 567.419 | 770.473 | 601.126 | 205.05 | 216.489 | 179.75 |
| YHR018C | 0.166 | -2.595 | 7.04E-33 | 7.96E-31 | yes | down | 681.633 | 594.468 | 687.81 | 116.704 | 81.996 | 115.904 |
| YHR029C | 0.403 | -1.312 | 8.92E-11 | 2.35E-09 | yes | down | 215.286 | 232.313 | 157.041 | 74.241 | 86.236 | 81.609 |
| YHR063C | 0.463 | -1.112 | 1.63E-05 | 0.000186 | yes | down | 125.768 | 68.301 | 96.08 | 40.612 | 43.979 | 48.729 |
| YHR086W | 0.013 | -6.308 | 4.13E-32 | 4.57E-30 | yes | down | 6.867 | 6.391 | 9.01 | 0 | 0 | 0 |
| YHR087W | 0.112 | -3.164 | 9.54E-07 | 1.41E-05 | yes | down | 15.795 | 23.321 | 9.573 | 0 | 0 | 0.01 |
| YHR106W | 0.378 | -1.405 | 3.88E-08 | 7.42E-07 | yes | down | 51.042 | 81.132 | 51.724 | 21.694 | 27.105 | 18.985 |
| YHR138C | 0.34 | -1.556 | 7.48E-08 | 1.34E-06 | yes | down | 884.096 | 1580.163 | 1246.469 | 374.591 | 506.249 | 288.03 |
| YHR145C | 0.238 | -2.07 | 0.000799 | 0.005617 | yes | down | 7.861 | 25.144 | 14.499 | 2.159 | 0 | 2.82 |
| YHR174W | 0.397 | -1.331 | 1.03E-05 | 0.000122 | yes | down | 11922.18 | 19933.21 | 10259.59 | 3702.101 | 5072.355 | 7447.342 |
| YHR207C | 0.147 | -2.762 | 1.22E-39 | 2.15E-37 | yes | down | 130.811 | 214.483 | 168.029 | 24.76 | 23.565 | 24.237 |
| YHR208W | 0.077 | -3.702 | 1.12E-67 | 9.03E-65 | yes | down | 5464.891 | 8946.476 | 6972.876 | 531.692 | 528.745 | 493.768 |
| YIL024C | 0.22 | -2.184 | 0.000825 | 0.005733 | yes | down | 0.255 | 2.82 | 9.593 | 0 | 0 | 0 |
| YIL029C | 0.373 | -1.421 | 0.000866 | 0.005972 | yes | down | 25.763 | 27.865 | 23.291 | 8.132 | 12.899 | 3.97 |
| YIL042C | 0.465 | -1.104 | 0.000325 | 0.002595 | yes | down | 15.211 | 31.337 | 20.996 | 10.076 | 12.351 | 8.042 |
| YIL046W | 0.374 | -1.419 | 0.00197 | 0.01181 | yes | down | 129.024 | 68.843 | 70.732 | 16.291 | 60.996 | 11.421 |
| YIL051C | 0.231 | -2.116 | 0.001099 | 0.007336 | yes | down | 536.021 | 893.605 | 682.331 | 66.418 | 76.119 | 2.474 |
| YIL059C | 0.467 | -1.097 | 0.03018 | 0.1052 | yes | down | 20.364 | 31.362 | 12.303 | 8.365 | 4.581 | 11.533 |
| YIL060W | 0.213 | -2.232 | 0.000667 | 0.004859 | yes | down | 11.782 | 15.564 | 0.178 | 0 | 0 | 0.662 |
| YIL062C | 0.342 | -1.55 | 7.29E-07 | 1.1E-05 | yes | down | 82.076 | 129.953 | 113.753 | 25.909 | 50.538 | 27.922 |
| YIL074C | 0.39 | -1.358 | 0.004726 | 0.02392 | yes | down | 22.571 | 12.744 | 25.684 | 7.524 | 1.543 | 11.289 |
| YIL082W | 0.043 | -4.525 | 6.29E-14 | 2.32E-12 | yes | down | 4.049 | 7.991 | 3.234 | 0 | 0 | 0 |
| YIL087C | 0.28 | -1.836 | 2.03E-08 | 4.07E-07 | yes | down | 119.567 | 248.776 | 161.62 | 50.08 | 54.106 | 30.569 |
| YIL116W | 0.261 | -1.937 | 1.44E-33 | 1.77E-31 | yes | down | 195.396 | 179.514 | 201.872 | 49.22 | 49.554 | 51.895 |
| YIL124W | 0.008 | -6.936 | 1.63E-41 | 3.17E-39 | yes | down | 22.051 | 24.552 | 27.306 | 0 | 0 | 0 |
| YIL136W | 0.207 | -2.271 | 1.28E-31 | 1.37E-29 | yes | down | 77.899 | 81.329 | 99.66 | 15.899 | 19.146 | 17.947 |
| YIL159W | 0.432 | -1.211 | 0.000389 | 0.003021 | yes | down | 1.705 | 1.699 | 1.711 | 0.449 | 1.032 | 0.662 |
| YIL160C | 0.429 | -1.222 | 6.68E-05 | 0.000657 | yes | down | 19.625 | 26.793 | 21.085 | 12.562 | 6.246 | 8.815 |
| YIL163C | 0.248 | -2.01 | 0.002058 | 0.01219 | yes | down | 6.493 | 2.229 | 11.235 | 0 | 0 | 0.234 |
| YIL164C | 0.489 | -1.032 | 5.48E-07 | 8.62E-06 | yes | down | 93.594 | 117.554 | 91.738 | 46.818 | 55.175 | 44.454 |
| YIL165C | 0.457 | -1.131 | 0.000592 | 0.004349 | yes | down | 98.701 | 164.984 | 94.122 | 55.37 | 56.689 | 38.631 |
| YIR006C | 0.414 | -1.274 | 0.04194 | 0.135 | yes | down | 7.004 | 8.151 | 6.28 | 2.028 | 0.189 | 3.339 |
| YIR007W | 0.313 | -1.675 | 1.32E-05 | 0.000153 | yes | down | 2.262 | 3.817 | 3.788 | 1.103 | 0.539 | 1.13 |
| YIR017C | 0.01 | -6.575 | 1.48E-34 | 1.85E-32 | yes | down | 28.936 | 63.708 | 54.236 | 0 | 0 | 0 |
| YIR019C | 0.456 | -1.132 | 0.0001 | 0.000931 | yes | down | 3.484 | 2.537 | 2.838 | 1.159 | 1.098 | 1.731 |
| YIR030C | 0.425 | -1.233 | 1.48E-05 | 0.000171 | yes | down | 57.006 | 42.591 | 36.85 | 22.432 | 14.489 | 19.056 |
| YIR032C | 0.378 | -1.405 | 8.65E-06 | 0.000104 | yes | down | 910.479 | 1992.125 | 1153.633 | 618.991 | 435.818 | 377.66 |
| YIR034C | 0.455 | -1.137 | 6.02E-08 | 1.11E-06 | yes | down | 250.697 | 330.19 | 271.991 | 150.1 | 117.827 | 116.739 |
| YIR036C | 0.195 | -2.358 | 4.16E-15 | 1.64E-13 | yes | down | 55.866 | 91.228 | 49.38 | 12.955 | 13.297 | 8.815 |
| YIR036W-A | 0.262 | -1.93 | 0.000121 | 0.001089 | yes | down | 16.789 | 20.588 | 16.645 | 1.243 | 4.741 | 4.795 |
| YIR037W | 0.257 | -1.96 | 1.58E-17 | 7.67E-16 | yes | down | 433.836 | 606.991 | 467.207 | 130.201 | 141.563 | 98.365 |
| YIR038C | 0.126 | -2.992 | 3.75E-47 | 1.11E-44 | yes | down | 240.474 | 257.949 | 203.642 | 31.938 | 27.19 | 25.082 |
| YIR039C | 0.26 | -1.942 | 1.28E-20 | 7.52E-19 | yes | down | 17.108 | 16.524 | 15.824 | 4.309 | 4.24 | 4.143 |
| YJL001W | 0.43 | -1.219 | 2.99E-09 | 6.77E-08 | yes | down | 242.726 | 335.423 | 260.974 | 113.208 | 136.604 | 105.022 |
| YJL045W | 0.103 | -3.285 | 1.92E-37 | 3.02E-35 | yes | down | 22.161 | 35.4 | 24.893 | 2.916 | 2.574 | 2.321 |
| YJL048C | 0.481 | -1.055 | 4.01E-06 | 5.23E-05 | yes | down | 141.426 | 140.801 | 113.466 | 45.332 | 78.012 | 67.694 |
| YJL066C | 0.404 | -1.309 | 2.64E-09 | 6.09E-08 | yes | down | 145.421 | 181.336 | 145.48 | 63.839 | 74.435 | 49.717 |
| YJL067W | 0.373 | -1.421 | 0.000158 | 0.001358 | yes | down | 37.399 | 40.289 | 39.144 | 13.497 | 14.101 | 10.76 |
| YJL077C | 0.241 | -2.052 | 0.00168 | 0.01036 | yes | down | 1.778 | 4.901 | 7.239 | 0 | 0 | 0 |
| YJL121C | 0.321 | -1.64 | 1.6E-05 | 0.000183 | yes | down | 109.134 | 250.179 | 129.023 | 24.89 | 43.146 | 74.036 |
| YJL153C | 0.212 | -2.241 | 1.02E-15 | 4.25E-14 | yes | down | 549.737 | 314.577 | 268.569 | 73.952 | 77.595 | 75.99 |
| YJL163C | 0.387 | -1.369 | 4.98E-07 | 7.88E-06 | yes | down | 22.361 | 45.473 | 30.807 | 11.721 | 13.874 | 11.574 |
| YJL167W | 0.469 | -1.092 | 0.00216 | 0.01268 | yes | down | 31.253 | 13.84 | 18.168 | 8.664 | 7.06 | 12.765 |
| YJL171C | 0.48 | -1.058 | 5.27E-11 | 1.42E-09 | yes | down | 887.06 | 821.622 | 764.031 | 348.682 | 437.843 | 415.589 |
| YJL172W | 0.427 | -1.228 | 3.59E-10 | 9.08E-09 | yes | down | 72.874 | 83.496 | 71.375 | 36.798 | 28.525 | 31.658 |
| YJL199C | 0.334 | -1.582 | 0.00046 | 0.00349 | yes | down | 48.808 | 84.702 | 43.763 | 23.713 | 12.474 | 11.371 |
| YJL201W | 0.281 | -1.832 | 0.001555 | 0.009722 | yes | down | 4.231 | 2.561 | 3.748 | 0.28 | 0.085 | 1.547 |
| YJL210W | 0.328 | -1.607 | 1.52E-06 | 2.15E-05 | yes | down | 235.13 | 602.681 | 294.827 | 125.481 | 133.339 | 85.997 |
| YJL211C | 0.357 | -1.485 | 1.29E-05 | 0.00015 | yes | down | 194.374 | 494.436 | 244.398 | 118.882 | 93.542 | 91.728 |
| YJL212C | 0.209 | -2.258 | 1.1E-12 | 3.62E-11 | yes | down | 45.798 | 28.887 | 36.078 | 9.048 | 4.268 | 7.981 |
| YJL219W | 0.386 | -1.375 | 2.95E-06 | 3.95E-05 | yes | down | 6.511 | 8.151 | 5.439 | 2.393 | 3.047 | 2.016 |
| YJR005C-A | 0.336 | -1.572 | 0.01633 | 0.06572 | yes | down | 13.953 | 16.069 | 9.494 | 1.29 | 0 | 2.789 |
| YJR010W | 0.401 | -1.32 | 0.000508 | 0.003808 | yes | down | 36.907 | 10.639 | 21.372 | 7.917 | 8.272 | 9.783 |
| YJR016C | 0.194 | -2.363 | 5.92E-23 | 4.03E-21 | yes | down | 3046.582 | 5663.326 | 4027.616 | 817.451 | 736.547 | 821.335 |
| YJR019C | 0.329 | -1.606 | 1.44E-08 | 3.01E-07 | yes | down | 61.439 | 128.377 | 76.162 | 30.227 | 27.275 | 25.815 |
| YJR020W | 0.389 | -1.364 | 6.14E-05 | 0.00061 | yes | down | 98.446 | 160.256 | 107.463 | 50.688 | 32.906 | 44.505 |
| YJR026W | 0.459 | -1.125 | 0.001903 | 0.01147 | yes | down | 19.461 | 17.916 | 9.425 | 5.113 | 5.044 | 10.292 |
| YJR039W | 0.452 | -1.145 | 0.000813 | 0.005667 | yes | down | 2.243 | 1.625 | 2.294 | 0.701 | 1.249 | 0.753 |
| YJR073C | 0.15 | -2.733 | 4.98E-22 | 3.09E-20 | yes | down | 953.697 | 1085.284 | 516.122 | 118.255 | 134.824 | 97.713 |
| YJR074W | 0.244 | -2.036 | 1.32E-15 | 5.4E-14 | yes | down | 257.026 | 222.758 | 145.796 | 41.846 | 47.008 | 56.883 |
| YJR085C | 0.357 | -1.484 | 3.22E-05 | 0.000345 | yes | down | 662.071 | 1905.551 | 1063.002 | 441.196 | 390.741 | 320.625 |
| YJR099W | 0.468 | -1.094 | 0.01953 | 0.07495 | yes | down | 9.712 | 7.314 | 3.946 | 1.449 | 3.795 | 3.573 |
| YJR137C | 0.264 | -1.921 | 3.28E-09 | 7.32E-08 | yes | down | 31.444 | 12.523 | 22.717 | 4.365 | 5.783 | 6.495 |
| YJR142W | 0.436 | -1.197 | 0.002442 | 0.01403 | yes | down | 4.998 | 3.337 | 6.498 | 1.673 | 2.47 | 1.924 |
| YJR150C | 0.227 | -2.142 | 2.23E-11 | 6.22E-10 | yes | down | 26.529 | 49.019 | 23.132 | 7.262 | 6.71 | 6.423 |
| YJR152W | 0.23 | -2.12 | 1.88E-16 | 8.36E-15 | yes | down | 135.261 | 77.832 | 116.028 | 26.283 | 25.345 | 21.316 |
| YJR156C | 0.338 | -1.565 | 2.27E-05 | 0.000254 | yes | down | 5.435 | 5.492 | 5.261 | 2.159 | 1.543 | 1.293 |
| YKL001C | 0.358 | -1.48 | 4.86E-08 | 9E-07 | yes | down | 183.267 | 124.646 | 149.04 | 38.555 | 72.447 | 47.976 |
| YKL016C | 0.442 | -1.178 | 9.05E-09 | 1.93E-07 | yes | down | 414.001 | 441.612 | 500.664 | 221.145 | 171.053 | 194.51 |
| YKL017C | 0.459 | -1.122 | 0.003149 | 0.01721 | yes | down | 2.289 | 3.608 | 1.721 | 1.094 | 1.287 | 0.906 |
| YKL029C | 0.317 | -1.658 | 1.23E-15 | 5.05E-14 | yes | down | 3582.476 | 4658.767 | 3841.993 | 1373.341 | 1015.385 | 1316.997 |
| YKL030W | 0.29 | -1.788 | 1.32E-20 | 7.69E-19 | yes | down | 942.79 | 1361.407 | 1049.314 | 308.92 | 322.212 | 320.451 |
| YKL050C | 0.474 | -1.078 | 0.000743 | 0.005269 | yes | down | 2.006 | 2.327 | 2.67 | 0.794 | 1.353 | 1.099 |
| YKL065W-A | 0.419 | -1.254 | 0.03762 | 0.1242 | yes | down | 53.86 | 89.997 | 66.223 | 12.749 | 15.417 | 28.91 |
| YKL068W-A | 0.447 | -1.161 | 0.000344 | 0.002731 | yes | down | 630.208 | 387.594 | 500.842 | 210.835 | 245.866 | 142.636 |
| YKL084W | 0.465 | -1.104 | 0.000809 | 0.005656 | yes | down | 166.77 | 323.554 | 215.658 | 124.808 | 76.706 | 99.729 |
| YKL120W | 0.122 | -3.037 | 4.82E-37 | 7.16E-35 | yes | down | 1415.366 | 856.419 | 1228.568 | 112.937 | 154.396 | 138.594 |
| YKL121W | 0.283 | -1.821 | 6.41E-09 | 1.38E-07 | yes | down | 6.402 | 12.018 | 14.202 | 2.879 | 2.555 | 3.247 |
| YKL141W | 0.37 | -1.436 | 1.7E-08 | 3.5E-07 | yes | down | 235.002 | 324.489 | 265.266 | 121.835 | 97.044 | 74.066 |
| YKL148C | 0.346 | -1.532 | 8.26E-07 | 1.24E-05 | yes | down | 156.373 | 309.677 | 301.542 | 107.6 | 83.605 | 59.988 |
| YKL150W | 0.487 | -1.037 | 6.58E-08 | 1.2E-06 | yes | down | 291.899 | 411.396 | 363.413 | 160.391 | 195.829 | 164.236 |
| YKL151C | 0.194 | -2.368 | 3.2E-26 | 2.78E-24 | yes | down | 109.161 | 172.643 | 130.368 | 27.265 | 25.591 | 24.064 |
| YKL163W | 0.198 | -2.334 | 5.34E-27 | 4.71E-25 | yes | down | 2211.841 | 2253.953 | 2201.107 | 327.829 | 401.88 | 561.94 |
| YKL165C | 0.278 | -1.847 | 2.72E-25 | 2.26E-23 | yes | down | 94.205 | 104.354 | 92.559 | 22.03 | 27.502 | 31.842 |
| YKL168C | 0.483 | -1.049 | 0.001156 | 0.007648 | yes | down | 3.42 | 4.753 | 4.49 | 2.57 | 1.495 | 1.832 |
| YKL192C | 0.427 | -1.228 | 0.000101 | 0.000933 | yes | down | 410.143 | 757.704 | 408.066 | 245.026 | 153.601 | 221.7 |
| YKL221W | 0.293 | -1.77 | 2.32E-10 | 5.96E-09 | yes | down | 7.442 | 10.885 | 8.891 | 2.664 | 2.338 | 2.626 |
| YKR046C | 0.374 | -1.42 | 7.06E-06 | 8.73E-05 | yes | down | 575.536 | 1325.797 | 917.522 | 414.193 | 338.14 | 240.97 |
| YKR049C | 0.091 | -3.463 | 3.04E-39 | 5.2E-37 | yes | down | 176.135 | 249.908 | 202.772 | 15.833 | 19.269 | 15.687 |
| YKR076W | 0.405 | -1.304 | 4.41E-06 | 5.69E-05 | yes | down | 35.84 | 35.536 | 40.855 | 18.937 | 9.862 | 15.096 |
| YKR102W | 0.497 | -1.01 | 0.002048 | 0.01215 | yes | down | 2.836 | 3.891 | 5.212 | 2.066 | 1.325 | 2.362 |
| YLL040C | 0.325 | -1.623 | 2.99E-11 | 8.2E-10 | yes | down | 2.417 | 2.45 | 2.196 | 0.692 | 0.965 | 0.611 |
| YLL041C | 0.289 | -1.792 | 3.82E-14 | 1.43E-12 | yes | down | 794.697 | 951.107 | 1087.984 | 320.454 | 261.103 | 210.289 |
| YLL049W | 0.377 | -1.409 | 1.69E-07 | 2.89E-06 | yes | down | 120.387 | 158.655 | 83.105 | 41.098 | 41.935 | 47.508 |
| YLL057C | 0.452 | -1.146 | 3.56E-07 | 5.84E-06 | yes | down | 17.674 | 23.358 | 23.033 | 10.104 | 9.048 | 9.559 |
| YLL058W | 0.236 | -2.084 | 2.19E-15 | 8.76E-14 | yes | down | 10.132 | 10.048 | 9.118 | 2.533 | 2.29 | 1.771 |
| YLL060C | 0.499 | -1.004 | 0.000103 | 0.00095 | yes | down | 66.254 | 106.238 | 63.078 | 31.947 | 42.796 | 40.922 |
| YLL061W | 0.371 | -1.43 | 6.74E-06 | 8.4E-05 | yes | down | 39.542 | 69.816 | 54.602 | 25.863 | 19.572 | 12.073 |
| YLL062C | 0.186 | -2.43 | 0.000147 | 0.001271 | yes | down | 0.866 | 2.967 | 3.63 | 0 | 0.369 | 0 |
| YLR004C | 0.236 | -2.086 | 1.26E-09 | 2.98E-08 | yes | down | 211.674 | 106.694 | 142.285 | 21.413 | 27.029 | 51.651 |
| YLR011W | 0.441 | -1.182 | 0.01499 | 0.06153 | yes | down | 3.985 | 2.512 | 2.561 | 0.963 | 1.003 | 1.669 |
| YLR038C | 0.411 | -1.284 | 8.11E-05 | 0.000782 | yes | down | 1185.662 | 2716.596 | 1908.446 | 700.037 | 828.755 | 626.387 |
| YLR039C | 0.43 | -1.217 | 0.03022 | 0.1052 | yes | down | 0.447 | 0.529 | 0.208 | 0.065 | 0.17 | 0.163 |
| YLR043C | 0.257 | -1.96 | 6.2E-11 | 1.66E-09 | yes | down | 305.305 | 365.394 | 199.835 | 69.045 | 65.595 | 63.887 |
| YLR058C | 0.358 | -1.481 | 7.25E-07 | 1.1E-05 | yes | down | 603.743 | 1386.218 | 1052.825 | 279.123 | 317.783 | 451.503 |
| YLR092W | 0.11 | -3.182 | 3.03E-25 | 2.48E-23 | yes | down | 65.706 | 36.779 | 84.914 | 6.795 | 5.678 | 6.026 |
| YLR154C | 0.316 | -1.661 | 0.001837 | 0.01116 | yes | down | 17.856 | 17.608 | 19.295 | 4.028 | 5.461 | 2.86 |
| YLR157C-A | 0.483 | -1.051 | 4.7E-07 | 7.49E-06 | yes | down | 68.743 | 64.755 | 49.914 | 28.816 | 26.698 | 33.083 |
| YLR164W | 0.235 | -2.09 | 1.91E-09 | 4.44E-08 | yes | down | 27.578 | 41.434 | 24.942 | 5.047 | 7.912 | 6.688 |
| YLR179C | 0.188 | -2.413 | 6.76E-35 | 8.68E-33 | yes | down | 634.02 | 684.502 | 476.029 | 107.89 | 110.426 | 107.934 |
| YLR180W | 0.334 | -1.584 | 2.5E-14 | 9.46E-13 | yes | down | 2491.83 | 2088.981 | 1675.698 | 720.384 | 714.609 | 630.388 |
| YLR194C | 0.206 | -2.276 | 1.92E-22 | 1.26E-20 | yes | down | 3283.883 | 5141.173 | 3701.36 | 667.239 | 747.781 | 994.519 |
| YLR227W-A | 0.459 | -1.125 | 0.001903 | 0.01147 | yes | down | 19.461 | 17.916 | 9.425 | 5.113 | 5.044 | 10.292 |
| YLR231C | 0.404 | -1.306 | 7.33E-13 | 2.47E-11 | yes | down | 52.857 | 58.451 | 50.023 | 19.61 | 25.241 | 20.715 |
| YLR251W | 0.412 | -1.28 | 4.04E-08 | 7.63E-07 | yes | down | 49.729 | 61.837 | 47.234 | 18.049 | 23.452 | 22.588 |
| YLR279W | 0.436 | -1.199 | 0.008428 | 0.03882 | yes | down | 20.893 | 50.927 | 22.539 | 14.693 | 12.228 | 7.971 |
| YLR281C | 0.383 | -1.384 | 2.93E-05 | 0.000318 | yes | down | 97.142 | 189.549 | 104.971 | 57.782 | 46.837 | 33.715 |
| YLR290C | 0.378 | -1.402 | 0.02389 | 0.08708 | yes | down | 1.103 | 0 | 2.136 | 0 | 0 | 0 |
| YLR303W | 0.118 | -3.089 | 2.45E-23 | 1.73E-21 | yes | down | 212.44 | 94.848 | 146.508 | 17.105 | 18.105 | 12.521 |
| YLR315W | 0.4 | -1.321 | 0.003151 | 0.01721 | yes | down | 8.481 | 12.104 | 13.836 | 3.898 | 3.568 | 4.408 |
| YLR348C | 0.412 | -1.278 | 1.57E-06 | 2.21E-05 | yes | down | 35.958 | 48.046 | 28.878 | 14.011 | 18.531 | 12.959 |
| YLR349W | 0.384 | -1.382 | 0.001044 | 0.007019 | yes | down | 16.689 | 33.406 | 15.794 | 9.814 | 6.975 | 5.039 |
| YLR353W | 0.356 | -1.492 | 1.22E-07 | 2.15E-06 | yes | down | 98.09 | 188.478 | 152.769 | 52.557 | 36.673 | 61.759 |
| YLR355C | 0.194 | -2.365 | 4.63E-13 | 1.6E-11 | yes | down | 2689.652 | 4537.396 | 4713.439 | 700.784 | 406.271 | 991.13 |
| YLR356W | 0.214 | -2.227 | 1.91E-14 | 7.38E-13 | yes | down | 196.171 | 387.311 | 257.324 | 67.157 | 51.418 | 46.48 |
| YLR359W | 0.34 | -1.557 | 1.23E-07 | 2.16E-06 | yes | down | 180.795 | 250.573 | 267.491 | 49.463 | 67.8 | 112.698 |
| YLR364W | 0.287 | -1.801 | 3.65E-08 | 7.06E-07 | yes | down | 86.745 | 87.116 | 72.305 | 22.199 | 20.139 | 19.972 |
| YLR395C | 0.445 | -1.169 | 0.000112 | 0.001021 | yes | down | 380.359 | 333.416 | 451.512 | 139.566 | 167.475 | 159.218 |
| YLR408C | 0.131 | -2.937 | 6.58E-06 | 8.22E-05 | yes | down | 4.04 | 16.45 | 14.874 | 0 | 0 | 0 |
| YLR410W-A | 0.188 | -2.408 | 5.24E-05 | 0.000525 | yes | down | 4.268 | 7.142 | 1.513 | 0.533 | 0.625 | 0 |
| YLR414C | 0.466 | -1.101 | 4.18E-08 | 7.83E-07 | yes | down | 1291.768 | 1505.348 | 1209.866 | 473.199 | 731.976 | 664.621 |
| YML027W | 0.378 | -1.403 | 3.34E-06 | 4.41E-05 | yes | down | 12.594 | 20.452 | 12.56 | 5.879 | 6.322 | 4.286 |
| YML040W | 0.459 | -1.125 | 0.001903 | 0.01147 | yes | down | 19.461 | 17.916 | 9.425 | 5.113 | 5.044 | 10.292 |
| YML042W | 0.449 | -1.154 | 1.29E-05 | 0.00015 | yes | down | 6.11 | 5.615 | 7.615 | 2.869 | 3.199 | 2.535 |
| YML078W | 0.425 | -1.236 | 7.08E-11 | 1.89E-09 | yes | down | 780.334 | 1055.609 | 829.918 | 344.691 | 420.837 | 351.774 |
| YML079W | 0.459 | -1.122 | 3.17E-07 | 5.26E-06 | yes | down | 122.968 | 137.267 | 102.756 | 48.641 | 67.261 | 48.872 |
| YML083C | 0.31 | -1.688 | 0.000136 | 0.001195 | yes | down | 2.599 | 6.194 | 2.848 | 0.794 | 1.278 | 1.008 |
| YML131W | 0.329 | -1.606 | 0.000188 | 0.001591 | yes | down | 102.723 | 60.815 | 68.26 | 6.225 | 34.212 | 27.413 |
| YMR009W | 0.329 | -1.602 | 1.02E-13 | 3.7E-12 | yes | down | 444.579 | 418.784 | 346.155 | 142.277 | 140.285 | 104.014 |
| YMR015C | 0.384 | -1.382 | 0.000116 | 0.001051 | yes | down | 8.089 | 4.457 | 5.163 | 1.785 | 1.722 | 2.922 |
| YMR032W | 0.396 | -1.338 | 0.01167 | 0.05082 | yes | down | 0.912 | 0.985 | 0.445 | 0.308 | 0.218 | 0.214 |
| YMR034C | 0.195 | -2.362 | 1.19E-11 | 3.44E-10 | yes | down | 31.043 | 35.093 | 28.967 | 3.225 | 9.417 | 4.021 |
| YMR035W | 0.15 | -2.738 | 6.69E-13 | 2.28E-11 | yes | down | 123.169 | 120.017 | 80.316 | 7.973 | 24.124 | 8.683 |
| YMR041C | 0.369 | -1.437 | 0.02015 | 0.07662 | yes | down | 22.936 | 0.702 | 12.847 | 0 | 0 | 0 |
| YMR046C | 0.343 | -1.545 | 6.05E-06 | 7.59E-05 | yes | down | 15.759 | 21.302 | 14.736 | 5.244 | 3.199 | 8.215 |
| YMR051C | 0.495 | -1.014 | 1.6E-05 | 0.000183 | yes | down | 109.307 | 138.979 | 82.244 | 48.762 | 50.254 | 63.917 |
| YMR056C | 0.002 | -9.052 | 6.93E-78 | 1.3E-74 | yes | down | 110.265 | 244.872 | 135.274 | 0 | 0 | 0 |
| YMR062C | 0.362 | -1.467 | 2.14E-23 | 1.53E-21 | yes | down | 740.39 | 856.948 | 765.416 | 285.535 | 296.167 | 279.021 |
| YMR072W | 0.369 | -1.44 | 0.02112 | 0.07917 | yes | down | 22.607 | 8.595 | 5.884 | 2.449 | 0 | 5.466 |
| YMR086W | 0.33 | -1.599 | 2.71E-09 | 6.23E-08 | yes | down | 9.284 | 14.764 | 9 | 3.767 | 3.861 | 2.922 |
| YMR087W | 0.434 | -1.205 | 4.14E-05 | 0.000429 | yes | down | 14.208 | 20.231 | 18.84 | 6.618 | 9.757 | 6.077 |
| YMR090W | 0.185 | -2.431 | 2.05E-23 | 1.48E-21 | yes | down | 237.419 | 394.539 | 330.747 | 60.623 | 61.261 | 47.294 |
| YMR105C | 0.008 | -6.937 | 4.82E-40 | 8.79E-38 | yes | down | 11.427 | 8.619 | 16.477 | 0 | 0 | 0 |
| YMR110C | 0.409 | -1.29 | 3.2E-12 | 9.98E-11 | yes | down | 36.706 | 41.656 | 45.118 | 15.59 | 18.89 | 16.287 |
| YMR168C | 0.353 | -1.501 | 1.25E-05 | 0.000146 | yes | down | 2.435 | 8.004 | 5.449 | 1.673 | 1.921 | 1.669 |
| YMR169C | 0.396 | -1.337 | 0.000559 | 0.004135 | yes | down | 17.464 | 34.551 | 31.361 | 11.169 | 14.679 | 4.54 |
| YMR174C | 0.253 | -1.983 | 3.29E-05 | 0.00035 | yes | down | 408.93 | 480.19 | 503.166 | 91.944 | 30.985 | 133.779 |
| YMR175W | 0.017 | -5.861 | 2.4E-38 | 3.87E-36 | yes | down | 835.115 | 2238.254 | 1708.998 | 12.048 | 8.414 | 16.42 |
| YMR189W | 0.258 | -1.956 | 1.07E-11 | 3.11E-10 | yes | down | 27.049 | 46.495 | 45.454 | 9.253 | 7.06 | 12.836 |
| YMR250W | 0.003 | -8.601 | 2.17E-72 | 2.45E-69 | yes | down | 31.298 | 44.845 | 43.436 | 0 | 0 | 0 |
| YMR262W | 0.106 | -3.244 | 7.2E-07 | 1.1E-05 | yes | down | 5.134 | 40.006 | 23.577 | 0 | 0 | 0 |
| YMR265C | 0.478 | -1.065 | 2.31E-05 | 0.000257 | yes | down | 231.974 | 142.353 | 134.957 | 76.513 | 78.845 | 87.025 |
| YMR271C | 0.433 | -1.206 | 1.29E-05 | 0.00015 | yes | down | 64.53 | 102.298 | 76.686 | 36.153 | 41.462 | 24.543 |
| YMR281W | 0.054 | -4.222 | 1.32E-11 | 3.79E-10 | yes | down | 1.313 | 9.961 | 5.598 | 0 | 0 | 0 |
| YMR297W | 0.422 | -1.243 | 8.75E-06 | 0.000105 | yes | down | 550.43 | 1098.595 | 686.356 | 362.851 | 343.828 | 250.732 |
| YMR316W | 0.098 | -3.344 | 6.05E-19 | 3.13E-17 | yes | down | 290.987 | 145.862 | 186.671 | 7.403 | 25.354 | 17.804 |
| YMR317W | 0.251 | -1.994 | 1.15E-06 | 1.66E-05 | yes | down | 0.52 | 1.047 | 0.316 | 0.131 | 0.095 | 0.173 |
| YMR320W | 0.488 | -1.035 | 0.04122 | 0.1332 | yes | down | 92.117 | 44.598 | 45.8 | 40.032 | 20.329 | 9.701 |
| YNL015W | 0.173 | -2.533 | 9.66E-08 | 1.71E-06 | yes | down | 254.409 | 149.531 | 264.494 | 21.974 | 26.48 | 28.879 |
| YNL018C | 0.455 | -1.135 | 2.66E-05 | 0.000291 | yes | down | 14.828 | 16.795 | 13.134 | 7.356 | 4.647 | 7.991 |
| YNL036W | 0.376 | -1.413 | 0.000239 | 0.001962 | yes | down | 96.23 | 45.325 | 98.394 | 19.796 | 18.455 | 44.525 |
| YNL046W | 0.482 | -1.054 | 0.009911 | 0.0445 | yes | down | 22.854 | 46.568 | 19.849 | 16.983 | 13.59 | 8.225 |
| YNL050C | 0.46 | -1.119 | 6.4E-07 | 9.98E-06 | yes | down | 48.47 | 88.618 | 63.928 | 31.527 | 32.168 | 26.141 |
| YNL052W | 0.377 | -1.406 | 8.37E-06 | 0.000102 | yes | down | 1585.619 | 3840.371 | 2763.732 | 1131.39 | 999.06 | 752.807 |
| YNL055C | 0.365 | -1.456 | 9.61E-06 | 0.000115 | yes | down | 1268.833 | 3037.675 | 2101.091 | 977.692 | 687.883 | 540.757 |
| YNL059C | 0.382 | -1.39 | 5.39E-06 | 6.8E-05 | yes | down | 3.037 | 2.647 | 2.937 | 0.944 | 1.23 | 1.018 |
| YNL071W | 0.185 | -2.433 | 1.51E-19 | 8.34E-18 | yes | down | 69.108 | 91.044 | 99.561 | 12.104 | 21.379 | 12.226 |
| YNL103W-A | 0.206 | -2.279 | 2.07E-38 | 3.44E-36 | yes | down | 462.645 | 491.752 | 453.569 | 106.796 | 86.813 | 93.865 |
| YNL115C | 0.366 | -1.452 | 3.99E-08 | 7.59E-07 | yes | down | 10.624 | 8.114 | 10.315 | 2.739 | 4.335 | 3.4 |
| YNL117W | 0.156 | -2.683 | 6.14E-28 | 5.59E-26 | yes | down | 34.691 | 42.998 | 54.266 | 7.319 | 5.934 | 6.271 |
| YNL135C | 0.3 | -1.738 | 9.72E-10 | 2.38E-08 | yes | down | 1440.463 | 2955.768 | 1741.219 | 534.655 | 588.728 | 568.628 |
| YNL138W | 0.422 | -1.243 | 5.48E-10 | 1.37E-08 | yes | down | 24.103 | 21.905 | 27.375 | 9.795 | 10.287 | 11.035 |
| YNL172W | 0.351 | -1.509 | 1.91E-09 | 4.44E-08 | yes | down | 2.563 | 3.411 | 3.511 | 1.075 | 1.249 | 0.967 |
| YNL195C | 0.46 | -1.121 | 0.000104 | 0.000954 | yes | down | 46.738 | 97.114 | 91.501 | 35.471 | 33.626 | 35.964 |
| YNL200C | 0.278 | -1.845 | 2.3E-08 | 4.61E-07 | yes | down | 122.102 | 313.395 | 214.017 | 62.035 | 63.485 | 40.392 |
| YNL206C | 0.48 | -1.06 | 2.29E-05 | 0.000256 | yes | down | 273.633 | 516.649 | 339.766 | 185.954 | 162.933 | 200.842 |
| YNL208W | 0.197 | -2.344 | 1.63E-15 | 6.61E-14 | yes | down | 5628.87 | 13713.11 | 8511.946 | 1677.803 | 1794.35 | 1564.98 |
| YNL220W | 0.376 | -1.41 | 4.3E-08 | 8.01E-07 | yes | down | 498.302 | 293.017 | 387.91 | 121.77 | 133.622 | 182.6 |
| YNL241C | 0.314 | -1.67 | 2.92E-06 | 3.92E-05 | yes | down | 354.979 | 1224.251 | 769.144 | 262.719 | 227.468 | 183.191 |
| YNL245C | 0.389 | -1.361 | 0.02774 | 0.09828 | yes | down | 125.759 | 36.004 | 59.586 | 2.131 | 0 | 0 |
| YNL274C | 0.022 | -5.504 | 2.2E-22 | 1.41E-20 | yes | down | 9.101 | 8.287 | 4.747 | 0 | 0 | 0 |
| YNL276C | 0.362 | -1.466 | 0.02517 | 0.09057 | yes | down | 11.737 | 28.099 | 9.336 | 6.122 | 0 | 0 |
| YNL277W | 0.413 | -1.276 | 8.72E-08 | 1.56E-06 | yes | down | 55.018 | 67.907 | 79.208 | 31.499 | 22.155 | 28.625 |
| YNL280C | 0.011 | -6.536 | 4.3E-35 | 5.78E-33 | yes | down | 8.563 | 13.828 | 11.423 | 0 | 0 | 0 |
| YNL332W | 0.313 | -1.677 | 0.000125 | 0.00112 | yes | down | 3.356 | 4.901 | 3.452 | 0.645 | 1.543 | 1.008 |
| YNL333W | 0.121 | -3.051 | 8.52E-37 | 1.23E-34 | yes | down | 186.459 | 139.164 | 148.279 | 12.936 | 19.874 | 21.489 |
| YNL334C | 0.465 | -1.104 | 0.000772 | 0.005446 | yes | down | 32.976 | 16.019 | 25.16 | 9.244 | 12.199 | 11.88 |
| YNR034W-A | 0.189 | -2.407 | 5.95E-17 | 2.78E-15 | yes | down | 772.901 | 1318.816 | 1100.573 | 178.963 | 215.619 | 138.024 |
| YNR050C | 0.477 | -1.068 | 0.000733 | 0.005216 | yes | down | 23.474 | 12.584 | 11.987 | 6.599 | 7.335 | 8.51 |
| YNR056C | 0.436 | -1.199 | 1.9E-06 | 2.64E-05 | yes | down | 64.676 | 43.576 | 37.127 | 21.152 | 20.698 | 20.939 |
| YNR058W | 0.281 | -1.833 | 1.48E-08 | 3.09E-07 | yes | down | 237.929 | 100.82 | 109.125 | 34.611 | 40.837 | 43.059 |
| YNR059W | 0.482 | -1.053 | 0.000361 | 0.00283 | yes | down | 9.348 | 6.391 | 6.824 | 3.43 | 2.858 | 4.418 |
| YNR065C | 0.473 | -1.079 | 0.000126 | 0.001121 | yes | down | 5.563 | 3.903 | 3.353 | 1.757 | 2.101 | 2.158 |
| YOL016C | 0.245 | -2.028 | 2.37E-12 | 7.52E-11 | yes | down | 337.78 | 160.884 | 232.155 | 44.537 | 61.563 | 65.23 |
| YOL049W | 0.381 | -1.391 | 5.09E-06 | 6.45E-05 | yes | down | 11.454 | 6.551 | 8.575 | 3.346 | 3.047 | 3.41 |
| YOL052C-A | 0.393 | -1.346 | 0.00073 | 0.005199 | yes | down | 996.449 | 1421.532 | 845.86 | 261.271 | 443.068 | 396.024 |
| YOL055C | 0.473 | -1.08 | 0.001774 | 0.01084 | yes | down | 28.8 | 13.249 | 23.152 | 8.702 | 7.117 | 14.119 |
| YOL058W | 0.371 | -1.431 | 8.12E-11 | 2.15E-09 | yes | down | 1129.869 | 1097.055 | 1041.155 | 339.288 | 336.096 | 530.078 |
| YOL064C | 0.46 | -1.121 | 3.64E-12 | 1.11E-10 | yes | down | 168.201 | 149.014 | 150.474 | 69.063 | 76.98 | 70.931 |
| YOL065C | 0.447 | -1.162 | 0.000198 | 0.001669 | yes | down | 7.287 | 6.686 | 5.37 | 2.43 | 2.678 | 3.298 |
| YOL077W-A | 0.443 | -1.173 | 5E-05 | 0.000504 | yes | down | 1685.369 | 2401.822 | 1724.475 | 601.138 | 950.14 | 802.971 |
| YOL091W | 0.346 | -1.531 | 5.62E-10 | 1.4E-08 | yes | down | 8.627 | 8.656 | 6.893 | 2.804 | 2.735 | 2.667 |
| YOL103W-A | 0.451 | -1.148 | 9.12E-05 | 0.000865 | yes | down | 77.115 | 74.569 | 35.969 | 23.657 | 28.723 | 30.406 |
| YOL110W | 0.432 | -1.21 | 0.000346 | 0.002739 | yes | down | 378.38 | 1116.732 | 602.579 | 325.062 | 287.347 | 241.224 |
| YOL129W | 0.38 | -1.396 | 3.71E-07 | 6.05E-06 | yes | down | 352.444 | 700.472 | 453.539 | 195.497 | 198.11 | 153.996 |
| YOL147C | 0.423 | -1.241 | 2.95E-05 | 0.00032 | yes | down | 111.149 | 207.428 | 146.746 | 78.616 | 63.863 | 45.676 |
| YOL153C | 0.067 | -3.9 | 3.9E-10 | 9.83E-09 | yes | down | 0.803 | 1.822 | 1.108 | 0 | 0 | 0 |
| YOL162W | 0.304 | -1.72 | 4.05E-07 | 6.6E-06 | yes | down | 19.397 | 30.155 | 21.323 | 8.235 | 6.814 | 4.591 |
| YOL163W | 0.311 | -1.687 | 2.31E-06 | 3.15E-05 | yes | down | 31.225 | 46.298 | 41.824 | 13.917 | 13.022 | 6.383 |
| YOL164W | 0.499 | -1.002 | 3.09E-08 | 6.07E-07 | yes | down | 47.504 | 58.291 | 43.782 | 25.208 | 25.013 | 24.96 |
| YOR003W | 0.358 | -1.482 | 0.01717 | 0.0679 | yes | down | 0.447 | 12.313 | 3.659 | 0 | 0 | 0 |
| YOR064C | 0.042 | -4.559 | 3.55E-14 | 1.34E-12 | yes | down | 6.849 | 12.375 | 5.004 | 0 | 0 | 0 |
| YOR073W | 0.313 | -1.677 | 2.65E-05 | 0.000291 | yes | down | 2.918 | 4.494 | 2.631 | 1.131 | 1.088 | 0.55 |
| YOR086C | 0.419 | -1.254 | 0.000757 | 0.005348 | yes | down | 1.113 | 0.825 | 1.058 | 0.336 | 0.435 | 0.428 |
| YOR090C | 0.495 | -1.015 | 1.21E-06 | 1.75E-05 | yes | down | 17.437 | 21.08 | 21.303 | 10.263 | 8.688 | 10.668 |
| YOR122C | 0.399 | -1.327 | 4.53E-09 | 9.96E-08 | yes | down | 1328 | 2000.892 | 1337.06 | 553.909 | 662.179 | 563.64 |
| YOR126C | 0.457 | -1.13 | 3.22E-05 | 0.000345 | yes | down | 24.112 | 41.791 | 35.485 | 14.095 | 15.218 | 15.839 |
| YOR128C | 0.41 | -1.285 | 3.56E-06 | 4.69E-05 | yes | down | 43.227 | 67.095 | 73.6 | 18.086 | 23.329 | 32.778 |
| YOR142W-A | 0.459 | -1.124 | 1.06E-06 | 1.55E-05 | yes | down | 24.878 | 36.927 | 24.972 | 11.945 | 14.546 | 13.05 |
| YOR154W | 0.49 | -1.029 | 0.000588 | 0.004332 | yes | down | 11.6 | 25.365 | 16.298 | 9.693 | 9.237 | 6.484 |
| YOR161C | 0.397 | -1.332 | 0.000212 | 0.001778 | yes | down | 49.921 | 179.539 | 90.68 | 39.64 | 41.935 | 37.043 |
| YOR176W | 0.283 | -1.819 | 2.79E-06 | 3.76E-05 | yes | down | 20.391 | 54.067 | 23.538 | 5.272 | 12.294 | 7.177 |
| YOR185C | 0.284 | -1.817 | 5.64E-15 | 2.21E-13 | yes | down | 71.041 | 60.815 | 56.303 | 16.572 | 20.471 | 14.893 |
| YOR202W | 0.29 | -1.786 | 1.96E-19 | 1.05E-17 | yes | down | 378.125 | 522.399 | 383.549 | 119.816 | 114.638 | 130.125 |
| YOR215C | 0.407 | -1.298 | 1.25E-05 | 0.000147 | yes | down | 31.581 | 25.451 | 29.066 | 11.216 | 13.732 | 8.612 |
| YOR220W | 0.389 | -1.363 | 3.42E-11 | 9.34E-10 | yes | down | 901.679 | 1269.058 | 957.348 | 378.862 | 471.971 | 353.789 |
| YOR222W | 0.2 | -2.319 | 2.44E-13 | 8.55E-12 | yes | down | 269.921 | 176.67 | 170.57 | 29.181 | 57.958 | 26.976 |
| YOR230W | 0.432 | -1.211 | 3.62E-07 | 5.92E-06 | yes | down | 361.29 | 463.394 | 433.858 | 223.276 | 171.251 | 140.345 |
| YOR271C | 0.337 | -1.568 | 1.04E-24 | 8.03E-23 | yes | down | 634.01 | 724.151 | 622.685 | 201.47 | 227.913 | 241.54 |
| YOR289W | 0.316 | -1.66 | 1.78E-11 | 5.05E-10 | yes | down | 104.328 | 152.671 | 117.017 | 43.5 | 41.093 | 29.653 |
| YOR343W-A | 0.316 | -1.663 | 1.24E-07 | 2.17E-06 | yes | down | 25.808 | 53.698 | 27.187 | 8.197 | 10.486 | 13.152 |
| YOR350C | 0.261 | -1.939 | 3.47E-05 | 0.000365 | yes | down | 19.251 | 11.205 | 13.282 | 0.636 | 4.041 | 4.754 |
| YOR362C | 0.45 | -1.152 | 5.64E-06 | 0.000071 | yes | down | 438.487 | 848.563 | 586.024 | 286.086 | 292.012 | 247.393 |
| YOR373W | 0.468 | -1.095 | 8.49E-06 | 0.000103 | yes | down | 17.181 | 29.071 | 19.038 | 10.225 | 8.981 | 11.024 |
| YOR385W | 0.171 | -2.547 | 4.97E-05 | 0.000501 | yes | down | 131.805 | 68.19 | 82.62 | 0.589 | 7.164 | 9.742 |
| YOR391C | 0.31 | -1.688 | 0.000181 | 0.001537 | yes | down | 11.244 | 6.194 | 8.199 | 3.206 | 2.271 | 1.211 |
| YPL004C | 0.281 | -1.83 | 1.57E-16 | 7.08E-15 | yes | down | 203.458 | 314.614 | 311.897 | 69.68 | 73.63 | 85.549 |
| YPL048W | 0.471 | -1.086 | 3.93E-05 | 0.00041 | yes | down | 980.636 | 1958.486 | 1115.824 | 641.619 | 642.881 | 589.415 |
| YPL109C | 0.476 | -1.071 | 0.001213 | 0.007894 | yes | down | 2.864 | 2.253 | 2.868 | 1.365 | 1.155 | 1.16 |
| YPL123C | 0.3 | -1.736 | 1.28E-05 | 0.000149 | yes | down | 3.347 | 2.635 | 3.57 | 0.851 | 1.013 | 0.702 |
| YPL149W | 0.345 | -1.534 | 1.23E-16 | 5.68E-15 | yes | down | 194.01 | 176.362 | 146.914 | 53.295 | 63.456 | 61.291 |
| YPL196W | 0.024 | -5.37 | 5.8E-20 | 3.28E-18 | yes | down | 4.459 | 22.004 | 15.478 | 0 | 0 | 0 |
| YPL223C | 0.004 | -8.136 | 3.62E-59 | 2.27E-56 | yes | down | 147.655 | 329.353 | 189.688 | 0 | 0 | 0 |
| YPL245W | 0.421 | -1.247 | 3.43E-05 | 0.000362 | yes | down | 46.181 | 62.145 | 47.58 | 30.227 | 16.42 | 16.196 |
| YPL246C | 0.21 | -2.25 | 1.64E-12 | 5.28E-11 | yes | down | 130.811 | 197.737 | 91.58 | 25.115 | 36.285 | 19.239 |
| YPL256C | 0.486 | -1.041 | 0.001211 | 0.007889 | yes | down | 6.065 | 7.314 | 5.222 | 3.524 | 1.959 | 3.237 |
| YPL257W-A | 0.483 | -1.051 | 4.7E-07 | 7.49E-06 | yes | down | 68.743 | 64.755 | 49.914 | 28.816 | 26.698 | 33.083 |
| YPL262W | 0.383 | -1.383 | 4.57E-10 | 1.15E-08 | yes | down | 251.819 | 186.754 | 226.468 | 98.207 | 78.873 | 75.42 |
| YPL274W | 0.007 | -7.188 | 6.94E-44 | 1.78E-41 | yes | down | 19.817 | 12.055 | 11.027 | 0 | 0 | 0 |
| YPR001W | 0.187 | -2.418 | 3.79E-33 | 4.37E-31 | yes | down | 26.492 | 30.918 | 27.86 | 4.907 | 5.849 | 4.896 |
| YPR010C-A | 0.457 | -1.129 | 0.001545 | 0.009674 | yes | down | 775.145 | 1408.961 | 891.7 | 431.784 | 511.682 | 302.912 |
| YPR028W | 0.429 | -1.221 | 7.03E-07 | 1.08E-05 | yes | down | 373.656 | 603.555 | 348.469 | 185.141 | 193.009 | 173.805 |
| YPR047W | 0.477 | -1.069 | 0.002262 | 0.01314 | yes | down | 3.383 | 5.061 | 4.055 | 1.449 | 2.555 | 1.741 |
| YPR058W | 0.377 | -1.408 | 5.44E-20 | 3.11E-18 | yes | down | 246.42 | 235.194 | 220.257 | 86.589 | 92.426 | 86.577 |
| YPR059C | 0.388 | -1.365 | 3.25E-05 | 0.000347 | yes | down | 77.352 | 61.775 | 63.354 | 24.189 | 32.471 | 16.023 |
| YPR128C | 0.277 | -1.854 | 3.71E-12 | 1.12E-10 | yes | down | 29.675 | 22.878 | 19.948 | 5.253 | 6.928 | 7.207 |
| YPR137C-A | 0.378 | -1.402 | 4.11E-08 | 7.7E-07 | yes | down | 41.494 | 30.143 | 24.082 | 10.973 | 12.587 | 12.114 |
| YPR138C | 0.378 | -1.404 | 2.42E-06 | 3.29E-05 | yes | down | 851.749 | 1554.466 | 1159.655 | 574.35 | 404.502 | 306.78 |
| YPR158W-A | 0.459 | -1.124 | 1.06E-06 | 1.55E-05 | yes | down | 24.878 | 36.927 | 24.972 | 11.945 | 14.546 | 13.05 |
| YPR160W | 0.32 | -1.642 | 3.44E-13 | 1.19E-11 | yes | down | 12.083 | 11.033 | 14.034 | 4.113 | 4.278 | 3.39 |
| YPR167C | 0.338 | -1.565 | 1.16E-08 | 2.45E-07 | yes | down | 113.101 | 59.608 | 88.188 | 24.461 | 32.197 | 28.92 |
| YPR172W | 0.371 | -1.429 | 8.06E-06 | 9.82E-05 | yes | down | 30.523 | 57.687 | 37.235 | 17.553 | 12.322 | 13.6 |
| YPR191W | 0.436 | -1.196 | 0.000555 | 0.004115 | yes | down | 96.412 | 177.692 | 211.584 | 96.935 | 49.478 | 52.903 |
| YPR197C | 0.442 | -1.179 | 0.002518 | 0.01438 | yes | down | 35.074 | 18.445 | 20.393 | 13.515 | 6.464 | 9.732 |
| YPR198W | 0.331 | -1.596 | 1.3E-11 | 3.75E-10 | yes | down | 159.62 | 101.35 | 104.427 | 39.285 | 41.68 | 38.367 |
