# Supplementary material for: Hydrogen sulfide treatment at the late growth stage of Saccharomyces cerevisiae extends chronological lifespan
Source: Aging (Albany NY). 2021 Mar 19;13(7):9859–73. doi: 10.18632/aging.202738 (PMC8064171; doi:10.18632/aging.202738)
Supplement: Supplementary Table 3 [file aging-13-202738-s004.doc]

**Supplementary Table 3. Common DEGs induced by the early and late NaHS treatments.**

Similar DEGs early treatment

| Gene_id | FC(NaHS/Control) | Log2FC(NaHS/Control) | Pvalue | Padjust | Significant | Regulate | Control1_Fpkm | Control2_Fpkm | Control3_Fpkm | NaHS1_Fpkm | NaHS2_Fpkm | NaHS3_Fpkm |
| --- | --- | --- | --- | --- | --- | --- | --- | --- | --- | --- | --- | --- |
| YER130C | 2.802 | 1.486332 | 7.11E-06 | 4.47E-05 | yes | up | 11.49 | 9.23 | 10.07 | 24.29 | 19.43 | 48.38 |
| YDR258C | 4.014 | 2.004966 | 1.88E-11 | 3.34E-10 | yes | up | 89.29 | 80.4 | 79.77 | 273.58 | 255.19 | 550.52 |
| YDL048C | 2.866 | 1.518988 | 1.72E-12 | 3.52E-11 | yes | up | 74.45 | 69.71 | 95.34 | 274.88 | 249.53 | 173.03 |
| YHR092C | 2.789 | 1.479905 | 4.44E-09 | 5.52E-08 | yes | up | 97.22 | 93.77 | 95.06 | 240.69 | 200.88 | 374.52 |
| YLR216C | 6.262 | 2.646596 | 2.06E-59 | 1.86E-56 | yes | up | 122.42 | 92.72 | 98.45 | 633.59 | 649.3 | 696.89 |
| YJL034W | 5.289 | 2.402959 | 1.84E-49 | 8.99E-47 | yes | up | 139.87 | 122.93 | 127.69 | 605.9 | 696.74 | 777.67 |
| YGR138C | 2.147 | 1.102007 | 2.73E-12 | 5.4E-11 | yes | up | 53.93 | 48.84 | 58.45 | 105.11 | 110.74 | 125.84 |
| YFL016C | 3.779 | 1.917906 | 7.25E-21 | 3.44E-19 | yes | up | 162.28 | 153.03 | 158.14 | 510.59 | 542.95 | 761.31 |
| YER098W | 2.016 | 1.011368 | 4.27E-06 | 2.83E-05 | yes | up | 12.61 | 8.52 | 8.19 | 20.91 | 17.74 | 20.55 |
| YLR297W | 2.05 | 1.035633 | 0.000452 | 0.001824 | yes | up | 207.04 | 186.67 | 158.21 | 276.91 | 291.54 | 550.46 |
| YDR042C | 2.103 | 1.072443 | 0.012159 | 0.031902 | yes | up | 1.88 | 2.11 | 1.54 | 5.25 | 2.88 | 4.87 |
| YGR211W | 6.118 | 2.613172 | 2.82E-29 | 3.03E-27 | yes | up | 130.84 | 112.49 | 111.03 | 577.48 | 714.57 | 984.99 |
| YBR150C | 3.733 | 1.900358 | 5.45E-16 | 1.67E-14 | yes | up | 9.64 | 8.72 | 8.51 | 29.23 | 28.76 | 45.4 |
| YNL077W | 3.495 | 1.805163 | 1.41E-10 | 2.26E-09 | yes | up | 344.27 | 320.43 | 323.62 | 881.31 | 961.91 | 1791.87 |
| YOL154W | 2.195 | 1.134205 | 9.47E-06 | 5.86E-05 | yes | up | 35.28 | 31.67 | 34.44 | 65.1 | 57.24 | 101.19 |
| YAL018C | 2.58 | 1.367334 | 0.008924 | 0.024424 | yes | up | 0.45 | 0.17 | 0.37 | 1.79 | 1.21 | 0.7 |
| YKL062W | 2.365 | 1.241845 | 6.73E-07 | 5.4E-06 | yes | up | 14.93 | 13.54 | 20 | 50.36 | 38.84 | 27.99 |
| YDR171W | 3.503 | 1.808683 | 1.26E-12 | 2.67E-11 | yes | up | 1121.64 | 1020.93 | 951.02 | 2925 | 3091.28 | 5192.61 |
| YPR142C | 2.171 | 1.118078 | 0.010862 | 0.028978 | yes | up | 4.82 | 1.27 | 2.88 | 9.47 | 6.58 | 5.8 |
| YPL240C | 4.756 | 2.249824 | 1.13E-22 | 6.7E-21 | yes | up | 261.45 | 232.97 | 235.54 | 1000.03 | 1013.29 | 1589.12 |
| YBR101C | 5.765 | 2.527205 | 6.09E-28 | 5.77E-26 | yes | up | 336.11 | 313.9 | 313.61 | 1619.2 | 1638.04 | 2603.94 |
| YOR051C | 2.091 | 1.064413 | 0.004432 | 0.013422 | yes | up | 4.61 | 5.58 | 3.49 | 7.01 | 6.86 | 16.65 |
| YPR157W | 2.509 | 1.327112 | 1.72E-10 | 2.73E-09 | yes | up | 20.75 | 19.99 | 18.41 | 62.8 | 48.19 | 38.73 |
| YLL024C | 3.2 | 1.678053 | 9.09E-23 | 5.55E-21 | yes | up | 175.85 | 174.17 | 172.89 | 470.74 | 561.09 | 640.19 |
| YKL109W | 3.009 | 1.589093 | 2.79E-14 | 7.24E-13 | yes | up | 114.85 | 90.95 | 117.1 | 382.1 | 238.22 | 390.66 |
| YPR158W | 2.621 | 1.389876 | 3.13E-06 | 2.14E-05 | yes | up | 27.54 | 19.52 | 18.78 | 40.34 | 55.7 | 82.63 |
| YDR219C | 2.182 | 1.125887 | 1.12E-12 | 2.4E-11 | yes | up | 15.87 | 15.77 | 14.08 | 30.61 | 34.03 | 34.04 |
| YOR232W | 2.479 | 1.309702 | 3.48E-10 | 5.22E-09 | yes | up | 79.62 | 69.81 | 79.93 | 165.02 | 165.75 | 234.06 |
| YJR078W | 2.393 | 1.258635 | 2.41E-05 | 0.000135 | yes | up | 44.66 | 43.5 | 41.24 | 137.37 | 132.04 | 55.52 |
| YBR169C | 3.067 | 1.616731 | 1.62E-42 | 3.95E-40 | yes | up | 108.2 | 94.73 | 101.18 | 301.11 | 317.09 | 302.35 |
| YPR065W | 2.818 | 1.494559 | 2.73E-06 | 1.89E-05 | yes | up | 125.14 | 112.13 | 113.88 | 499.51 | 397.02 | 168.22 |
| YLL026W | 3.518 | 1.814899 | 3.6E-11 | 6.2E-10 | yes | up | 405.33 | 352.61 | 361.03 | 1080.92 | 1049.53 | 2002.92 |
| YDR054C | 2.562 | 1.357124 | 4.28E-10 | 6.3E-09 | yes | up | 61.84 | 50.78 | 44.38 | 121.27 | 118.95 | 162.83 |
| YBR244W | 2.143 | 1.099687 | 1.65E-05 | 9.47E-05 | yes | up | 12.38 | 10.93 | 11.95 | 22.41 | 24.95 | 27.86 |
| YLR217W | 7.421 | 2.891707 | 2.53E-76 | 3.22E-73 | yes | up | 93.26 | 92.02 | 93.97 | 693.07 | 704.91 | 617.64 |
| YDR343C | 2.747 | 1.457948 | 1.6E-06 | 1.17E-05 | yes | up | 2714.46 | 2408.89 | 3065.43 | 11393.07 | 8367.07 | 4176.88 |
| YER103W | 2.282 | 1.190199 | 1.09E-16 | 3.6E-15 | yes | up | 108.92 | 109.97 | 120 | 385.23 | 411.79 | 443.8 |
| YKL105C | 2.197 | 1.135557 | 0.008798 | 0.02414 | yes | up | 7.07 | 6.99 | 6.44 | 24.09 | 24.18 | 3.96 |
| YGR250C | 2.756 | 1.462475 | 4.77E-15 | 1.34E-13 | yes | up | 57.8 | 57.6 | 62.66 | 146.32 | 146.83 | 197.51 |
| YPL089C | 2.252 | 1.171281 | 9.92E-15 | 2.71E-13 | yes | up | 31.69 | 27.91 | 27.79 | 70.69 | 68.46 | 55.96 |
| YNL006W | 3.233 | 1.692703 | 5.05E-22 | 2.77E-20 | yes | up | 190.96 | 176.82 | 167.57 | 489.56 | 576.06 | 658.69 |
| YLR300W | 2.101 | 1.070755 | 0.000613 | 0.002397 | yes | up | 17.45 | 18.26 | 21.46 | 50.05 | 54.65 | 20.89 |
| YCR061W | 0.498 | -1.00702 | 7.89E-05 | 0.000385 | yes | down | 164.66 | 160.73 | 158.71 | 85.87 | 96.04 | 45.68 |
| YDL222C | 0.091 | -3.4649 | 2.22E-47 | 8.81E-45 | yes | down | 4591.86 | 4576.66 | 4501.22 | 263.56 | 473 | 274.29 |
| YNR016C | 0.358 | -1.48394 | 2.5E-18 | 9.44E-17 | yes | down | 17.35 | 13.88 | 17.16 | 5.77 | 6.1 | 4.68 |
| YGR055W | 0.27 | -1.88742 | 2.41E-12 | 4.82E-11 | yes | down | 279.61 | 284.67 | 351.75 | 94.06 | 86.25 | 43.78 |
| YLR070C | 0.188 | -2.41124 | 3.31E-23 | 2.08E-21 | yes | down | 208.88 | 218.99 | 199.27 | 38.37 | 45.43 | 22.54 |
| YBR234C | 0.447 | -1.16261 | 1.57E-25 | 1.24E-23 | yes | down | 338.19 | 320.02 | 345.32 | 141.97 | 147.1 | 143.59 |
| YER119C-A | 0.46 | -1.11873 | 0.001246 | 0.004477 | yes | down | 34.23 | 18.89 | 28.33 | 8.75 | 11.32 | 12.04 |
| YLR178C | 0.37 | -1.43438 | 8.88E-22 | 4.77E-20 | yes | down | 2851.92 | 2753.58 | 2830.3 | 915.52 | 935.95 | 1107.13 |
| YGR088W | 0.075 | -3.72999 | 1.69E-56 | 1.19E-53 | yes | down | 998.24 | 849.67 | 1032.35 | 69.33 | 78.37 | 41.32 |
| YJL060W | 0.479 | -1.06293 | 0.000606 | 0.002372 | yes | down | 38.41 | 36.18 | 37.47 | 11.51 | 11.88 | 25.24 |
| YGL104C | 0.26 | -1.94169 | 2.35E-17 | 8.27E-16 | yes | down | 80.68 | 82.54 | 79.48 | 16.83 | 15.57 | 25.42 |
| YGR019W | 0.295 | -1.75899 | 1.59E-36 | 2.66E-34 | yes | down | 588.06 | 529.26 | 655.12 | 172.03 | 176.75 | 152.35 |
| YHR086W | 0.485 | -1.04352 | 0.000231 | 0.00101 | yes | down | 100.25 | 95.11 | 90.34 | 51.28 | 54.79 | 23.38 |
| YNL280C | 0.301 | -1.73436 | 7.26E-24 | 4.75E-22 | yes | down | 87.09 | 82.24 | 98.85 | 21.67 | 28.41 | 26.15 |
| YER065C | 0.274 | -1.86891 | 2.08E-05 | 0.000117 | yes | down | 9004.83 | 8705.26 | 8800.12 | 2114.65 | 2937.38 | 383.95 |
| YJL153C | 0.186 | -2.42674 | 8.86E-29 | 8.93E-27 | yes | down | 907.12 | 795.31 | 891.45 | 121.45 | 128.56 | 188.57 |
| YMR051C | 0.326 | -1.61625 | 2.86E-10 | 4.36E-09 | yes | down | 69.4 | 75.51 | 86.77 | 28.4 | 26.92 | 14.27 |
| YGL161C | 0.395 | -1.33903 | 6.53E-29 | 6.68E-27 | yes | down | 258.52 | 252.51 | 236.96 | 94.08 | 97.41 | 92.83 |
| YFL055W | 0.255 | -1.97121 | 1.08E-09 | 1.48E-08 | yes | down | 227.75 | 216.24 | 215.18 | 44.41 | 75.18 | 28.35 |
| YML040W | 0.373 | -1.42446 | 8.75E-12 | 1.61E-10 | yes | down | 40 | 54.81 | 49.47 | 20.39 | 15.22 | 15.08 |
| YFR044C | 0.316 | -1.66398 | 5.17E-47 | 1.93E-44 | yes | down | 431.49 | 428.35 | 434.96 | 136.9 | 124.86 | 131.5 |
| YAR073W | 0.361 | -1.47027 | 1.18E-05 | 7.07E-05 | yes | down | 3.61 | 6.3 | 5.84 | 1.43 | 1.56 | 1.99 |
| YGR256W | 0.282 | -1.82563 | 2.11E-19 | 8.71E-18 | yes | down | 96.26 | 94.09 | 101.98 | 22.56 | 22.24 | 31.95 |
| YDR451C | 0.499 | -1.0015 | 7.24E-08 | 6.9E-07 | yes | down | 53.65 | 60.51 | 69.44 | 26.54 | 28.15 | 32.61 |
| YLR348C | 0.34 | -1.55512 | 9.95E-22 | 5.31E-20 | yes | down | 162.79 | 161.33 | 150.78 | 47.62 | 47.65 | 57.81 |
| YGR087C | 0.269 | -1.89264 | 1.23E-21 | 6.48E-20 | yes | down | 34.1 | 30.61 | 43.52 | 8.23 | 10.06 | 8.95 |
| YGR244C | 0.314 | -1.6704 | 6.06E-12 | 1.14E-10 | yes | down | 314.93 | 293.06 | 323.49 | 95.24 | 117.66 | 57.85 |
| YMR262W | 0.405 | -1.30481 | 2.5E-13 | 5.69E-12 | yes | down | 486.01 | 482.93 | 424.4 | 178.46 | 214.24 | 144.62 |
| YNR056C | 0.074 | -3.75569 | 1.38E-52 | 7.94E-50 | yes | down | 167.07 | 220.42 | 192.72 | 9.84 | 9.51 | 15.58 |
| YBR116C | 0.062 | -4.01843 | 4.8E-84 | 7.61E-81 | yes | down | 267.04 | 240.42 | 262.62 | 14.41 | 10.43 | 16.34 |
| YKR076W | 0.298 | -1.74573 | 1.84E-31 | 2.44E-29 | yes | down | 263.71 | 227.44 | 289.7 | 73.33 | 79.92 | 68.55 |
| YLR251W | 0.477 | -1.06814 | 1.05E-10 | 1.71E-09 | yes | down | 331.72 | 302.06 | 301.22 | 135.51 | 126.38 | 159.92 |
| YER044C | 0.494 | -1.01866 | 1.94E-08 | 2.09E-07 | yes | down | 182.27 | 154.22 | 178.93 | 84.52 | 67.58 | 85.6 |
| YLR227W-A | 0.366 | -1.44859 | 9.35E-10 | 1.29E-08 | yes | down | 36.69 | 41.63 | 54.46 | 17.89 | 16.37 | 11.14 |
| YJL045W | 0.232 | -2.10554 | 8.69E-26 | 7.07E-24 | yes | down | 191.96 | 166.26 | 202.7 | 35.61 | 51.88 | 34.04 |
| YPR160W | 0.418 | -1.25674 | 1.28E-07 | 1.17E-06 | yes | down | 78.57 | 75.15 | 87.24 | 25.44 | 26.51 | 42.05 |
| YBR026C | 0.261 | -1.93924 | 1.16E-44 | 3.34E-42 | yes | down | 282.51 | 269.52 | 284.04 | 63.79 | 69.11 | 74.69 |
| YIL042C | 0.377 | -1.40897 | 2.67E-06 | 1.85E-05 | yes | down | 253.92 | 256.4 | 241.29 | 101.08 | 112.93 | 43.14 |
| YIL124W | 0.309 | -1.69365 | 5.2E-43 | 1.32E-40 | yes | down | 293.25 | 295.01 | 296.05 | 83.15 | 88.78 | 89.72 |
| YCR004C | 0.409 | -1.29138 | 1.14E-19 | 4.81E-18 | yes | down | 727.89 | 724.16 | 670.62 | 273.01 | 313.57 | 243.4 |
| YLR315W | 0.443 | -1.17578 | 0.002724 | 0.008799 | yes | down | 38.52 | 27.28 | 40.71 | 19.42 | 15.96 | 4.78 |
| YGR144W | 0.386 | -1.37324 | 5.01E-10 | 7.27E-09 | yes | down | 40.89 | 37.27 | 47.7 | 16.15 | 11.69 | 17.45 |
| YLL060C | 0.495 | -1.01461 | 1.02E-14 | 2.77E-13 | yes | down | 189.45 | 183.48 | 188.74 | 89.04 | 97.23 | 80.61 |
| YFR030W | 0.323 | -1.63182 | 3.42E-30 | 4.09E-28 | yes | down | 79 | 85.23 | 98.08 | 27.94 | 27.98 | 25.31 |
| YPL246C | 0.206 | -2.27616 | 4.34E-46 | 1.45E-43 | yes | down | 224.14 | 236.5 | 256.36 | 40.64 | 49.7 | 48.6 |
| YNL274C | 0.471 | -1.08512 | 3.79E-07 | 3.16E-06 | yes | down | 141.22 | 129.96 | 134.79 | 68.55 | 71.28 | 41.96 |
| YJR039W | 0.41 | -1.28688 | 3.96E-13 | 8.85E-12 | yes | down | 19.7 | 19.37 | 16.39 | 6.65 | 8.48 | 6.65 |
| YLR349W | 0.301 | -1.7327 | 4.18E-18 | 1.54E-16 | yes | down | 95.14 | 93.26 | 84.8 | 27.86 | 28.86 | 19.64 |
| YMR169C | 0.444 | -1.17251 | 5.8E-14 | 1.42E-12 | yes | down | 214.03 | 199.44 | 180.66 | 77.51 | 82.82 | 92.18 |
| YKL163W | 0.449 | -1.15588 | 0.03198 | 0.071195 | yes | down | 1010.7 | 1038.18 | 1529.82 | 519.54 | 492.99 | 68 |
| YML042W | 0.221 | -2.17755 | 1.59E-12 | 3.27E-11 | yes | down | 1228.02 | 1159.75 | 1050.53 | 214.12 | 329 | 115.52 |
| YER091C | 0.183 | -2.45233 | 2.38E-28 | 2.29E-26 | yes | down | 175.22 | 169.16 | 278.24 | 34.7 | 33.54 | 35.28 |
| YOL049W | 0.492 | -1.0238 | 4.13E-08 | 4.11E-07 | yes | down | 25.41 | 19.98 | 24.83 | 10.41 | 10.4 | 12.08 |
| YNL138W | 0.405 | -1.30487 | 2.83E-08 | 2.96E-07 | yes | down | 115.69 | 111.52 | 109.95 | 42.43 | 56.91 | 28.98 |
| YKR102W | 0.469 | -1.09331 | 1.02E-06 | 7.77E-06 | yes | down | 6.03 | 5.4 | 6.51 | 3.39 | 2.54 | 2.06 |
| YAL061W | 0.367 | -1.44566 | 3.17E-06 | 2.15E-05 | yes | down | 4669.94 | 5106.9 | 4566.36 | 1988.04 | 1999.6 | 760.57 |
| YNR050C | 0.431 | -1.21436 | 3.24E-08 | 3.33E-07 | yes | down | 28.35 | 28.36 | 29.45 | 10.69 | 9.45 | 14.69 |
| YPL257W-A | 0.395 | -1.34026 | 5.88E-12 | 1.11E-10 | yes | down | 63.04 | 57.78 | 56.86 | 25.79 | 24.34 | 16.6 |
| YJR019C | 0.23 | -2.11957 | 3.7E-38 | 7.12E-36 | yes | down | 528.95 | 536.77 | 468.85 | 113.08 | 128.27 | 92.55 |
| YJR137C | 0.237 | -2.07406 | 1.21E-29 | 1.4E-27 | yes | down | 7.45 | 6.93 | 8.41 | 1.53 | 1.83 | 1.72 |
| YJR020W | 0.247 | -2.02017 | 4.54E-20 | 2.05E-18 | yes | down | 739.18 | 702.8 | 584.7 | 176.69 | 164.37 | 103.31 |
| YOR176W | 0.267 | -1.90665 | 6.64E-26 | 5.55E-24 | yes | down | 245.31 | 246.45 | 254.48 | 52.65 | 60.25 | 73.69 |
| YKL050C | 0.496 | -1.01192 | 0.000648 | 0.002511 | yes | down | 4.83 | 3.91 | 4.91 | 1.31 | 2.1 | 2.82 |
| YDL204W | 0.053 | -4.23909 | 4.3E-173 | 1.4E-169 | yes | down | 404.34 | 383.08 | 406.08 | 16.86 | 21.17 | 20.27 |
| YDR070C | 0.304 | -1.72013 | 2.45E-21 | 1.25E-19 | yes | down | 1973.82 | 1658 | 1901.42 | 432.56 | 482.49 | 572.33 |
| YLR303W | 0.48 | -1.05943 | 0.000662 | 0.002559 | yes | down | 147.98 | 129.74 | 233.81 | 62.74 | 53.44 | 106.39 |
| YDR210C-C | 0.34 | -1.55652 | 2.17E-12 | 4.37E-11 | yes | down | 66.58 | 77.26 | 77.58 | 25.32 | 28.83 | 16.45 |
| YER137C-A | 0.385 | -1.37536 | 1.51E-09 | 2.03E-08 | yes | down | 54.16 | 60.06 | 64.54 | 27.8 | 21.63 | 15.31 |
| YJR016C | 0.496 | -1.01283 | 2.44E-05 | 0.000136 | yes | down | 2133.04 | 2083.84 | 2452.35 | 1272.55 | 1165.8 | 657.78 |
| YKL151C | 0.367 | -1.44612 | 8.04E-30 | 9.45E-28 | yes | down | 1003.42 | 977 | 984.11 | 332.87 | 338.47 | 371.61 |
| YLL062C | 0.464 | -1.10718 | 4.98E-06 | 3.24E-05 | yes | down | 21.29 | 26.62 | 25.59 | 11.55 | 13.1 | 7.48 |
| YML131W | 0.498 | -1.00493 | 6.63E-11 | 1.1E-09 | yes | down | 415.7 | 445.01 | 452.94 | 198.78 | 194.26 | 234.27 |
| YGR122W | 0.287 | -1.79994 | 1.33E-28 | 1.32E-26 | yes | down | 52.07 | 53.88 | 54.89 | 13.45 | 16.97 | 13.45 |
| YOR343W-A | 0.401 | -1.31896 | 0.000204 | 0.000907 | yes | down | 29.15 | 28.02 | 37.67 | 14.38 | 14.63 | 4.51 |
| YOR373W | 0.38 | -1.39631 | 7.84E-09 | 9.2E-08 | yes | down | 238.53 | 223.14 | 209.74 | 86.64 | 101.31 | 51.01 |
| YJR026W | 0.422 | -1.24627 | 1.04E-08 | 1.19E-07 | yes | down | 30.05 | 41.14 | 40.66 | 16.67 | 16.28 | 11.6 |
| YDR119W-A | 0.449 | -1.15499 | 0.000902 | 0.003367 | yes | down | 4722.78 | 6196.16 | 5226.83 | 2562.78 | 2430.95 | 816.38 |
| YBR218C | 0.439 | -1.18932 | 5.01E-22 | 2.77E-20 | yes | down | 185.84 | 198.03 | 191.32 | 88.12 | 82.11 | 74.09 |
| YNR058W | 0.133 | -2.91425 | 2.96E-65 | 3.13E-62 | yes | down | 102.52 | 113.52 | 122.97 | 14.84 | 12.11 | 14.83 |
| YDR107C | 0.28 | -1.8362 | 1.73E-25 | 1.36E-23 | yes | down | 22.3 | 25.26 | 28.93 | 6.52 | 7.21 | 6.54 |
| YJL219W | 0.424 | -1.23795 | 2.17E-08 | 2.32E-07 | yes | down | 14.36 | 16.16 | 19.77 | 8.02 | 5.58 | 6.45 |
| YDR226W | 0.443 | -1.17391 | 6.31E-13 | 1.38E-11 | yes | down | 481.71 | 427.24 | 577.97 | 216.87 | 219.98 | 189.97 |
| YER181C | 0.482 | -1.05275 | 0.010666 | 0.028588 | yes | down | 31.47 | 23.27 | 23.85 | 4.94 | 11.36 | 14.29 |
| YDL085W | 0.148 | -2.75174 | 3.05E-37 | 5.53E-35 | yes | down | 653.8 | 649.46 | 556.82 | 81.62 | 108.12 | 61.98 |
| YBR038W | 0.277 | -1.85441 | 1.55E-12 | 3.2E-11 | yes | down | 5.88 | 3.99 | 6.5 | 1.4 | 1.57 | 1.14 |
| YDL218W | 0.094 | -3.40491 | 2.75E-49 | 1.25E-46 | yes | down | 89.98 | 78.28 | 122.15 | 9.26 | 7.9 | 6.89 |
| YGR248W | 0.361 | -1.46945 | 3.97E-25 | 2.86E-23 | yes | down | 1650.9 | 1531.25 | 1343.38 | 505.84 | 516.78 | 534.94 |
| YGL234W | 0.499 | -1.00158 | 0.012523 | 0.032736 | yes | down | 107.13 | 129.69 | 163.14 | 76.71 | 81.57 | 17.61 |
| YAR010C | 0.353 | -1.50279 | 2E-28 | 1.95E-26 | yes | down | 107 | 102.88 | 109.24 | 40.11 | 34.77 | 33.23 |
| YER159C-A | 0.381 | -1.39273 | 1.24E-18 | 4.8E-17 | yes | down | 69.03 | 81.06 | 73.55 | 27.16 | 30.32 | 24.1 |
| YAL012W | 0.325 | -1.61961 | 7.43E-20 | 3.14E-18 | yes | down | 85.08 | 79.87 | 95.97 | 26.65 | 31.7 | 22.19 |
| YIR017C | 0.315 | -1.66513 | 1.03E-18 | 4.01E-17 | yes | down | 178.43 | 205.71 | 230.64 | 67.4 | 52.39 | 60.95 |
| YDR365W-A | 0.383 | -1.38358 | 1.28E-20 | 5.95E-19 | yes | down | 59.14 | 61.41 | 54.42 | 21.9 | 20.28 | 22.1 |
| YJL172W | 0.434 | -1.2051 | 2.8E-16 | 8.94E-15 | yes | down | 88.19 | 91.8 | 78.53 | 33.45 | 38.74 | 35.67 |
| YPR001W | 0.253 | -1.98221 | 2.93E-13 | 6.65E-12 | yes | down | 431.58 | 466.36 | 413.36 | 116.16 | 126.17 | 56.58 |
| YMR250W | 0.324 | -1.6249 | 7.45E-12 | 1.38E-10 | yes | down | 840.25 | 777.92 | 813.01 | 198.02 | 200.79 | 326.7 |
| YBR138C | 0.438 | -1.19026 | 7.12E-05 | 0.000352 | yes | down | 4.07 | 5.8 | 5.05 | 1.76 | 1.59 | 2.6 |
| YIR036W-A | 0.463 | -1.10977 | 5.57E-10 | 8E-09 | yes | down | 256.33 | 248.83 | 285.15 | 102.61 | 109.65 | 127.66 |
| YHR106W | 0.429 | -1.22105 | 8.89E-14 | 2.13E-12 | yes | down | 336.9 | 335.15 | 313.45 | 142.48 | 153.66 | 108.85 |
| YIL160C | 0.244 | -2.03481 | 2.06E-29 | 2.25E-27 | yes | down | 480.32 | 476.09 | 375.49 | 90.16 | 117.73 | 97.6 |
| YER152W-A | 0.375 | -1.41399 | 2.21E-14 | 5.82E-13 | yes | down | 153.08 | 127.38 | 152.36 | 56.58 | 55.78 | 40.28 |
| YOL065C | 0.468 | -1.09392 | 1.45E-13 | 3.39E-12 | yes | down | 51.9 | 51.89 | 47.56 | 22.45 | 24.8 | 20.95 |
| YCR088W | 0.427 | -1.22819 | 1.47E-27 | 1.34E-25 | yes | down | 228.86 | 217.76 | 213.42 | 94.64 | 92.21 | 85.77 |
| YNL117W | 0.143 | -2.80425 | 2.27E-14 | 5.96E-13 | yes | down | 2797.28 | 2464.93 | 2484.5 | 309.04 | 434.55 | 104.58 |
| YPL262W | 0.424 | -1.23723 | 3.55E-34 | 5.25E-32 | yes | down | 1505.73 | 1436.49 | 1489.1 | 642.37 | 632.34 | 622.96 |
| YDR032C | 0.455 | -1.13508 | 1.42E-13 | 3.34E-12 | yes | down | 1475.09 | 1481.26 | 1443.38 | 586.22 | 600.22 | 713.94 |
| YPR128C | 0.49 | -1.02985 | 1.3E-06 | 9.76E-06 | yes | down | 41.88 | 35.19 | 54.02 | 22.73 | 20.15 | 17.92 |
| YLR011W | 0.472 | -1.08369 | 0.005801 | 0.016826 | yes | down | 10.21 | 7.25 | 14.37 | 2.32 | 5.2 | 5.27 |
| YOR161C | 0.174 | -2.52111 | 4.76E-11 | 8.11E-10 | yes | down | 2117.83 | 2192.04 | 2079.49 | 333.67 | 425.61 | 92.68 |
| YOR086C | 0.368 | -1.44277 | 3.09E-12 | 6.07E-11 | yes | down | 11.82 | 11.27 | 11.67 | 3.34 | 5.2 | 3.55 |
| YIR007W | 0.438 | -1.19204 | 8.38E-09 | 9.78E-08 | yes | down | 33.47 | 29.91 | 31.4 | 14.36 | 15.69 | 9.47 |
| YBR117C | 0.036 | -4.7993 | 0 | 0 | yes | down | 764.61 | 754.78 | 745.69 | 26.39 | 26.82 | 23.57 |
| YBR241C | 0.499 | -1.00243 | 8.67E-09 | 1.01E-07 | yes | down | 1077.79 | 1000.66 | 954.75 | 514.52 | 559.86 | 381.09 |
| YJL212C | 0.316 | -1.66094 | 3.77E-10 | 5.6E-09 | yes | down | 62.4 | 57.54 | 74.76 | 21.95 | 23.25 | 11.23 |
| YOL163W | 0.482 | -1.05284 | 0.000261 | 0.00113 | yes | down | 26.04 | 27.52 | 36.86 | 12.42 | 17.73 | 9.77 |
| YLL061W | 0.152 | -2.72097 | 1.89E-13 | 4.34E-12 | yes | down | 180.6 | 172.52 | 266.6 | 31.66 | 31.42 | 9.02 |
| YPR137C-A | 0.435 | -1.19952 | 1.02E-09 | 1.4E-08 | yes | down | 89.66 | 95.67 | 105.3 | 46.83 | 43.65 | 30.07 |
| YOR090C | 0.404 | -1.30584 | 2.33E-19 | 9.49E-18 | yes | down | 52.84 | 47.39 | 49.3 | 18.31 | 21.65 | 18.17 |
| YPL223C | 0.307 | -1.70578 | 1.06E-11 | 1.94E-10 | yes | down | 359.86 | 341.12 | 443.05 | 87.94 | 85.72 | 140.77 |
| YBR285W | 0.132 | -2.92229 | 4.95E-49 | 2.09E-46 | yes | down | 373.36 | 437.44 | 411.87 | 56.59 | 51.43 | 37.14 |
| YLR164W | 0.228 | -2.13282 | 6.39E-31 | 8.11E-29 | yes | down | 398.95 | 386.62 | 396.34 | 91.93 | 108.28 | 72.26 |
| YPR158W-A | 0.414 | -1.27165 | 1.6E-06 | 1.18E-05 | yes | down | 64.42 | 57.88 | 68.86 | 23.65 | 34.38 | 15.54 |
| YOR142W-A | 0.422 | -1.2431 | 2.43E-14 | 6.36E-13 | yes | down | 70.47 | 60.52 | 71.73 | 27.08 | 31.31 | 23.69 |
| YIR032C | 0.446 | -1.1643 | 1.22E-05 | 7.26E-05 | yes | down | 75.13 | 73.03 | 95.63 | 25.2 | 29.3 | 44.79 |
| YLR157C-A | 0.39 | -1.36014 | 2.7E-12 | 5.36E-11 | yes | down | 50.97 | 64.91 | 57.5 | 23.11 | 24.14 | 16.95 |
| YOL103W-A | 0.295 | -1.76286 | 1.95E-30 | 2.39E-28 | yes | down | 47.39 | 53.64 | 49.84 | 14.72 | 14.94 | 12.78 |
| YPL274W | 0.351 | -1.51113 | 1.27E-07 | 1.16E-06 | yes | down | 315.94 | 316.52 | 320.8 | 109.63 | 140.16 | 55.35 |
| YBL080C | 0.474 | -1.07725 | 0.000713 | 0.002725 | yes | down | 5.67 | 5.86 | 5.65 | 2.99 | 1.33 | 3.06 |
| YGR286C | 0.12 | -3.06471 | 2.06E-41 | 4.66E-39 | yes | down | 443.32 | 493.37 | 443.6 | 44.09 | 66.66 | 36.71 |
| YML027W | 0.271 | -1.88568 | 1.78E-29 | 2.02E-27 | yes | down | 98.35 | 102.4 | 93.46 | 22.98 | 23.68 | 28.47 |
| YOL153C | 0.46 | -1.11917 | 8.09E-14 | 1.96E-12 | yes | down | 142.69 | 151.13 | 143.91 | 56.46 | 70.81 | 66.52 |
| YPL004C | 0.484 | -1.04694 | 1.53E-15 | 4.5E-14 | yes | down | 5665.85 | 5575.79 | 5415.32 | 2335.75 | 2505.95 | 2799.26 |
| YJR156C | 0.499 | -1.0023 | 8.67E-08 | 8.16E-07 | yes | down | 31.13 | 35.29 | 34.8 | 13.58 | 17.28 | 17.27 |
| YBR214W | 0.431 | -1.21526 | 8.83E-26 | 7.09E-24 | yes | down | 1169.44 | 1157.93 | 1084.52 | 460.89 | 503.08 | 456.72 |
| YEL035C | 0.487 | -1.03693 | 5.76E-07 | 4.66E-06 | yes | down | 47.6 | 52.8 | 61.75 | 25.18 | 24.57 | 24.27 |

Similar DEGs late treatment

| Gene_id | FC(NaHS/Control) | Log2FC(NaHS/Control) | Pvalue | Padjust | Significant | Regulate | Control1_Fpkm | Control2_Fpkm | Control3_Fpkm | NaHS1_Fpkm | NaHS2_Fpkm | NaHS3_Fpkm |
| --- | --- | --- | --- | --- | --- | --- | --- | --- | --- | --- | --- | --- |
| YER130C | 3.119 | 1.641 | 1.2E-09 | 2.87E-08 | yes | up | 34.727 | 23.888 | 47.264 | 110.918 | 135.449 | 111.303 |
| YDR258C | 249 | 7.96 | 9.01E-58 | 4.24E-55 | yes | up | 0 | 0 | 0 | 13.329 | 25.515 | 22.059 |
| YDL048C | 7.749 | 2.954 | 1.9E-19 | 1.04E-17 | yes | up | 65.843 | 127.023 | 158.782 | 1403.466 | 874.721 | 913.979 |
| YHR092C | 4.727 | 2.241 | 0.000161 | 0.001373 | yes | up | 24.203 | 192.418 | 103.448 | 1502.794 | 1041.44 | 343.223 |
| YLR216C | 2.145 | 1.101 | 3.18E-06 | 4.21E-05 | yes | up | 68.233 | 49.265 | 62.158 | 98.926 | 152.863 | 155.37 |
| YJL034W | 2.44 | 1.287 | 1.26E-08 | 2.66E-07 | yes | up | 148.357 | 112.555 | 159.573 | 304.453 | 341.717 | 440 |
| YGR138C | 2.537 | 1.343 | 1.84E-12 | 5.91E-11 | yes | up | 1627.487 | 1227.243 | 1353.675 | 3404.331 | 4202.943 | 3588.273 |
| YFL016C | 3.673 | 1.877 | 0.001273 | 0.008255 | yes | up | 32.675 | 4.211 | 22.42 | 84.532 | 128.502 | 157.243 |
| YER098W | 2.469 | 1.304 | 1.07E-06 | 1.56E-05 | yes | up | 1.842 | 2.586 | 2.769 | 6.206 | 7.098 | 5.629 |
| YLR297W | 3.945 | 1.98 | 1.12E-13 | 4.06E-12 | yes | up | 147.4 | 140.555 | 213.057 | 839.434 | 662.983 | 557.472 |
| YDR042C | 2.383 | 1.253 | 0.000119 | 0.001068 | yes | up | 4.724 | 6.908 | 6.122 | 13.833 | 16.042 | 15.33 |
| YGR211W | 2.336 | 1.224 | 1.07E-07 | 1.88E-06 | yes | up | 172.798 | 123.44 | 141.524 | 327.885 | 303.104 | 448.184 |
| YBR150C | 2.024 | 1.017 | 0.002848 | 0.01585 | yes | up | 0.903 | 0.837 | 1.019 | 1.542 | 2.839 | 1.7 |
| YNL077W | 2.683 | 1.424 | 1.07E-12 | 3.54E-11 | yes | up | 56.487 | 76.071 | 70.445 | 153.801 | 222.016 | 196.282 |
| YOL154W | 3.95 | 1.982 | 2.93E-17 | 1.39E-15 | yes | up | 58.037 | 63.093 | 38.62 | 203.676 | 262.295 | 204.69 |
| YAL018C | 2.512 | 1.329 | 0.005218 | 0.02587 | yes | up | 0.711 | 0.751 | 1.256 | 2 | 2.811 | 3.542 |
| YKL062W | 2.092 | 1.065 | 0.000751 | 0.005314 | yes | up | 3.657 | 3.263 | 3.857 | 11.076 | 6.303 | 6.637 |
| YDR171W | 2.512 | 1.329 | 3.97E-09 | 8.84E-08 | yes | up | 156.592 | 160.712 | 172.548 | 391.471 | 340.146 | 562.51 |
| YPR142C | 2.088 | 1.062 | 0.02904 | 0.1021 | yes | up | 3.465 | 2.056 | 5.776 | 5.365 | 14.082 | 8.612 |
| YPL240C | 2.556 | 1.354 | 1.83E-13 | 6.52E-12 | yes | up | 68.689 | 59.633 | 75.282 | 155.587 | 198.65 | 191.67 |
| YBR101C | 2.086 | 1.061 | 1E-09 | 2.43E-08 | yes | up | 148.166 | 140.567 | 159.494 | 286.385 | 311.158 | 368.794 |
| YOR051C | 86.044 | 6.427 | 2.02E-33 | 2.39E-31 | yes | up | 0 | 0 | 0 | 9.468 | 12.369 | 17.56 |
| YPR157W | 5.22 | 2.384 | 2.88E-16 | 1.26E-14 | yes | up | 17.765 | 32.137 | 41.31 | 190.861 | 171.621 | 168.349 |
| YLL024C | 2.89 | 1.531 | 1.06E-09 | 2.55E-08 | yes | up | 240.337 | 151.957 | 171.53 | 491.725 | 521.079 | 737.782 |
| YKL109W | 10.44 | 3.384 | 5.24E-31 | 5.38E-29 | yes | up | 4.76 | 6.083 | 9.959 | 95.412 | 85.375 | 71.46 |
| YPR158W | 2.535 | 1.342 | 4.38E-05 | 0.00045 | yes | up | 11.554 | 7.265 | 12.689 | 26.04 | 21.843 | 39.324 |
| YDR219C | 2.042 | 1.03 | 0.007545 | 0.03536 | yes | up | 2.043 | 0.997 | 2.591 | 4.141 | 4.278 | 4.387 |
| YOR232W | 10.375 | 3.375 | 2.19E-11 | 6.12E-10 | yes | up | 1.031 | 1.034 | 0.762 | 21.357 | 21.162 | 5.619 |
| YJR078W | 2.011 | 1.008 | 2.39E-06 | 3.26E-05 | yes | up | 45.197 | 73.214 | 58.311 | 124.35 | 126.051 | 118.144 |
| YBR169C | 2.908 | 1.54 | 2.76E-11 | 7.59E-10 | yes | up | 21.896 | 15.564 | 22.025 | 49.585 | 62.699 | 72.641 |
| YPR065W | 3.249 | 1.7 | 4.85E-07 | 7.7E-06 | yes | up | 103.908 | 49.314 | 117.768 | 427.344 | 256.465 | 296.642 |
| YLL026W | 4.039 | 2.014 | 1.64E-07 | 2.83E-06 | yes | up | 28.043 | 9.629 | 36.058 | 87.336 | 122.673 | 148.448 |
| YDR054C | 2.154 | 1.107 | 0.000454 | 0.00345 | yes | up | 20.008 | 9.407 | 15.478 | 27.508 | 33.588 | 43.477 |
| YBR244W | 3.618 | 1.855 | 6.29E-08 | 1.16E-06 | yes | up | 23.611 | 18.396 | 13.144 | 47.65 | 107.142 | 68.335 |
| YLR217W | 2.321 | 1.215 | 0.000128 | 0.001133 | yes | up | 54.571 | 46.827 | 78.931 | 111.47 | 142.822 | 177.103 |
| YDR343C | 2.142 | 1.099 | 0.003586 | 0.0192 | yes | up | 236.434 | 856.505 | 498.825 | 1453.658 | 1419.754 | 841.094 |
| YER103W | 3.967 | 1.988 | 1.69E-13 | 6.04E-12 | yes | up | 60.554 | 42.431 | 56.768 | 147.969 | 285.681 | 265.93 |
| YKL105C | 2.256 | 1.174 | 6.65E-05 | 0.000656 | yes | up | 8.737 | 14.419 | 11.176 | 37.331 | 24.105 | 20.542 |
| YGR250C | 3.961 | 1.986 | 6.94E-08 | 1.26E-06 | yes | up | 9.065 | 4.26 | 11.759 | 26.451 | 57.153 | 34.04 |
| YPL089C | 2.154 | 1.107 | 1.95E-06 | 2.72E-05 | yes | up | 28.873 | 26.276 | 32.102 | 46.201 | 77.387 | 75.491 |
| YNL006W | 2.099 | 1.07 | 1.65E-08 | 3.4E-07 | yes | up | 185.51 | 227.191 | 180.322 | 348.467 | 451.566 | 490.867 |
| YLR300W | 2.85 | 1.511 | 1.89E-11 | 5.35E-10 | yes | up | 293.887 | 197.614 | 236.902 | 601.297 | 873.794 | 731.633 |
| YCR061W | 0.21 | -2.253 | 2.28E-12 | 7.28E-11 | yes | down | 29.365 | 45.78 | 31.549 | 7.393 | 9.227 | 3.878 |
| YDL222C | 0.46 | -1.121 | 0.001527 | 0.009592 | yes | down | 12.567 | 29.133 | 17.594 | 9.328 | 6.076 | 10.19 |
| YNR016C | 0.305 | -1.713 | 4.72E-05 | 0.000477 | yes | down | 20.884 | 5.836 | 10.335 | 2.234 | 2.47 | 5.385 |
| YGR055W | 0.366 | -1.449 | 3.27E-09 | 7.32E-08 | yes | down | 174.001 | 111.73 | 117.363 | 38.612 | 52.156 | 55.804 |
| YLR070C | 0.416 | -1.265 | 0.000128 | 0.001132 | yes | down | 15.385 | 37.001 | 20.373 | 10.487 | 9.634 | 8.53 |
| YBR234C | 0.32 | -1.644 | 1.04E-24 | 8.03E-23 | yes | down | 97.224 | 112.309 | 101.292 | 30.928 | 33.399 | 35.415 |
| YER119C-A | 0.388 | -1.367 | 0.02105 | 0.07905 | yes | down | 4.742 | 18.002 | 8.268 | 2.804 | 2.158 | 2.972 |
| YLR178C | 0.211 | -2.246 | 1.85E-15 | 7.48E-14 | yes | down | 159.757 | 346.789 | 247.415 | 49.996 | 53.235 | 44.719 |
| YGR088W | 0.073 | -3.782 | 3.72E-43 | 8.41E-41 | yes | down | 34.518 | 41.286 | 62.741 | 2.813 | 3.284 | 3.003 |
| YJL060W | 0.212 | -2.241 | 2.91E-21 | 1.75E-19 | yes | down | 31.855 | 22.693 | 29.076 | 5.814 | 5.726 | 5.629 |
| YGL104C | 0.241 | -2.05 | 9.93E-15 | 3.87E-13 | yes | down | 11.016 | 13.815 | 10.256 | 2.29 | 3.275 | 2.575 |
| YGR019W | 0.326 | -1.617 | 5.22E-09 | 1.14E-07 | yes | down | 158.972 | 265.817 | 204.859 | 82.551 | 47.632 | 66.208 |
| YHR086W | 0.013 | -6.308 | 4.13E-32 | 4.57E-30 | yes | down | 6.867 | 6.391 | 9.01 | 0 | 0 | 0 |
| YNL280C | 0.011 | -6.536 | 4.3E-35 | 5.78E-33 | yes | down | 8.563 | 13.828 | 11.423 | 0 | 0 | 0 |
| YER065C | 0.047 | -4.411 | 2.73E-72 | 2.57E-69 | yes | down | 513.021 | 305.478 | 490.31 | 18.637 | 18.067 | 19.606 |
| YJL153C | 0.212 | -2.241 | 1.02E-15 | 4.25E-14 | yes | down | 549.737 | 314.577 | 268.569 | 73.952 | 77.595 | 75.99 |
| YMR051C | 0.495 | -1.014 | 1.6E-05 | 0.000183 | yes | down | 109.307 | 138.979 | 82.244 | 48.762 | 50.254 | 63.917 |
| YGL161C | 0.19 | -2.397 | 1.31E-21 | 8.04E-20 | yes | down | 44.348 | 68.671 | 44.959 | 8.936 | 10.761 | 8.826 |
| YFL055W | 0.492 | -1.022 | 4.2E-05 | 0.000434 | yes | down | 10.077 | 12.67 | 9.524 | 5.804 | 4.401 | 5.507 |
| YML040W | 0.459 | -1.125 | 0.001903 | 0.01147 | yes | down | 19.461 | 17.916 | 9.425 | 5.113 | 5.044 | 10.292 |
| YFR044C | 0.157 | -2.668 | 3.61E-24 | 2.72E-22 | yes | down | 84.329 | 72.796 | 50.962 | 10.431 | 12.313 | 8.103 |
| YAR073W | 0.458 | -1.127 | 9.52E-05 | 0.000893 | yes | down | 5.937 | 7.425 | 6.508 | 2.542 | 3.104 | 3.268 |
| YGR256W | 0.21 | -2.251 | 2.93E-23 | 2.02E-21 | yes | down | 23.273 | 25.796 | 19.661 | 3.823 | 5.129 | 5.12 |
| YDR451C | 0.322 | -1.637 | 7.1E-08 | 1.28E-06 | yes | down | 16.962 | 31.029 | 17.673 | 5.244 | 7.656 | 7.177 |
| YLR348C | 0.412 | -1.278 | 1.57E-06 | 2.21E-05 | yes | down | 35.958 | 48.046 | 28.878 | 14.011 | 18.531 | 12.959 |
| YGR087C | 0.351 | -1.509 | 6.42E-14 | 2.36E-12 | yes | down | 16.16 | 18.642 | 16.328 | 5.477 | 5.063 | 7.024 |
| YGR244C | 0.418 | -1.26 | 6.44E-07 | 1E-05 | yes | down | 86.782 | 49.08 | 68.665 | 26.143 | 27.588 | 30.936 |
| YMR262W | 0.106 | -3.244 | 7.2E-07 | 1.1E-05 | yes | down | 5.134 | 40.006 | 23.577 | 0 | 0 | 0 |
| YNR056C | 0.436 | -1.199 | 1.9E-06 | 2.64E-05 | yes | down | 64.676 | 43.576 | 37.127 | 21.152 | 20.698 | 20.939 |
| YBR116C | 0.214 | -2.224 | 7.04E-07 | 1.08E-05 | yes | down | 17.473 | 14 | 11.492 | 1.355 | 3.861 | 2.117 |
| YKR076W | 0.405 | -1.304 | 4.41E-06 | 5.69E-05 | yes | down | 35.84 | 35.536 | 40.855 | 18.937 | 9.862 | 15.096 |
| YLR251W | 0.412 | -1.28 | 4.04E-08 | 7.63E-07 | yes | down | 49.729 | 61.837 | 47.234 | 18.049 | 23.452 | 22.588 |
| YER044C | 0.467 | -1.098 | 0.001471 | 0.009316 | yes | down | 20.61 | 33.96 | 26.802 | 11.272 | 12.899 | 11.33 |
| YLR227W-A | 0.459 | -1.125 | 0.001903 | 0.01147 | yes | down | 19.461 | 17.916 | 9.425 | 5.113 | 5.044 | 10.292 |
| YJL045W | 0.103 | -3.285 | 1.92E-37 | 3.02E-35 | yes | down | 22.161 | 35.4 | 24.893 | 2.916 | 2.574 | 2.321 |
| YPR160W | 0.32 | -1.642 | 3.44E-13 | 1.19E-11 | yes | down | 12.083 | 11.033 | 14.034 | 4.113 | 4.278 | 3.39 |
| YBR026C | 0.12 | -3.055 | 5.12E-18 | 2.58E-16 | yes | down | 36.715 | 15.391 | 21.955 | 2.066 | 2.839 | 2.748 |
| YIL042C | 0.465 | -1.104 | 0.000325 | 0.002595 | yes | down | 15.211 | 31.337 | 20.996 | 10.076 | 12.351 | 8.042 |
| YIL124W | 0.008 | -6.936 | 1.63E-41 | 3.17E-39 | yes | down | 22.051 | 24.552 | 27.306 | 0 | 0 | 0 |
| YCR004C | 0.412 | -1.278 | 3.33E-12 | 1.03E-10 | yes | down | 88.095 | 89.344 | 82.758 | 31.134 | 40.875 | 35.16 |
| YLR315W | 0.4 | -1.321 | 0.003151 | 0.01721 | yes | down | 8.481 | 12.104 | 13.836 | 3.898 | 3.568 | 4.408 |
| YGR144W | 0.054 | -4.222 | 2.08E-58 | 1.07E-55 | yes | down | 346.844 | 215.936 | 198.984 | 12.17 | 13.127 | 11.381 |
| YLL060C | 0.499 | -1.004 | 0.000103 | 0.00095 | yes | down | 66.254 | 106.238 | 63.078 | 31.947 | 42.796 | 40.922 |
| YFR030W | 0.138 | -2.861 | 1.58E-21 | 9.59E-20 | yes | down | 131.012 | 68.572 | 121.635 | 13.805 | 9.975 | 16.745 |
| YPL246C | 0.21 | -2.25 | 1.64E-12 | 5.28E-11 | yes | down | 130.811 | 197.737 | 91.58 | 25.115 | 36.285 | 19.239 |
| YNL274C | 0.022 | -5.504 | 2.2E-22 | 1.41E-20 | yes | down | 9.101 | 8.287 | 4.747 | 0 | 0 | 0 |
| YJR039W | 0.452 | -1.145 | 0.000813 | 0.005667 | yes | down | 2.243 | 1.625 | 2.294 | 0.701 | 1.249 | 0.753 |
| YLR349W | 0.384 | -1.382 | 0.001044 | 0.007019 | yes | down | 16.689 | 33.406 | 15.794 | 9.814 | 6.975 | 5.039 |
| YMR169C | 0.396 | -1.337 | 0.000559 | 0.004135 | yes | down | 17.464 | 34.551 | 31.361 | 11.169 | 14.679 | 4.54 |
| YKL163W | 0.198 | -2.334 | 5.34E-27 | 4.71E-25 | yes | down | 2211.841 | 2253.953 | 2201.107 | 327.829 | 401.88 | 561.94 |
| YML042W | 0.449 | -1.154 | 1.29E-05 | 0.00015 | yes | down | 6.11 | 5.615 | 7.615 | 2.869 | 3.199 | 2.535 |
| YER091C | 0.012 | -6.374 | 6.17E-61 | 4.36E-58 | yes | down | 293.513 | 108.627 | 285.827 | 2.084 | 0.975 | 2.178 |
| YOL049W | 0.381 | -1.391 | 5.09E-06 | 6.45E-05 | yes | down | 11.454 | 6.551 | 8.575 | 3.346 | 3.047 | 3.41 |
| YNL138W | 0.422 | -1.243 | 5.48E-10 | 1.37E-08 | yes | down | 24.103 | 21.905 | 27.375 | 9.795 | 10.287 | 11.035 |
| YKR102W | 0.497 | -1.01 | 0.002048 | 0.01215 | yes | down | 2.836 | 3.891 | 5.212 | 2.066 | 1.325 | 2.362 |
| YAL061W | 0.25 | -2.002 | 2.66E-12 | 8.36E-11 | yes | down | 61.256 | 138.585 | 115.939 | 22.703 | 25.856 | 26.029 |
| YNR050C | 0.477 | -1.068 | 0.000733 | 0.005216 | yes | down | 23.474 | 12.584 | 11.987 | 6.599 | 7.335 | 8.51 |
| YPL257W-A | 0.483 | -1.051 | 4.7E-07 | 7.49E-06 | yes | down | 68.743 | 64.755 | 49.914 | 28.816 | 26.698 | 33.083 |
| YJR019C | 0.329 | -1.606 | 1.44E-08 | 3.01E-07 | yes | down | 61.439 | 128.377 | 76.162 | 30.227 | 27.275 | 25.815 |
| YJR137C | 0.264 | -1.921 | 3.28E-09 | 7.32E-08 | yes | down | 31.444 | 12.523 | 22.717 | 4.365 | 5.783 | 6.495 |
| YJR020W | 0.389 | -1.364 | 6.14E-05 | 0.00061 | yes | down | 98.446 | 160.256 | 107.463 | 50.688 | 32.906 | 44.505 |
| YOR176W | 0.283 | -1.819 | 2.79E-06 | 3.76E-05 | yes | down | 20.391 | 54.067 | 23.538 | 5.272 | 12.294 | 7.177 |
| YKL050C | 0.474 | -1.078 | 0.000743 | 0.005269 | yes | down | 2.006 | 2.327 | 2.67 | 0.794 | 1.353 | 1.099 |
| YDL204W | 0.212 | -2.238 | 3.22E-10 | 8.2E-09 | yes | down | 8.609 | 7.216 | 8.713 | 1.495 | 0.965 | 2.168 |
| YDR070C | 0.085 | -3.558 | 6.8E-29 | 6.4E-27 | yes | down | 395.443 | 374.973 | 291.049 | 19.282 | 24.209 | 29.236 |
| YLR303W | 0.118 | -3.089 | 2.45E-23 | 1.73E-21 | yes | down | 212.44 | 94.848 | 146.508 | 17.105 | 18.105 | 12.521 |
| YDR210C-C | 0.329 | -1.606 | 4.2E-07 | 6.79E-06 | yes | down | 27.158 | 35.893 | 18.781 | 6.337 | 12.142 | 7.095 |
| YER137C-A | 0.483 | -1.051 | 4.7E-07 | 7.49E-06 | yes | down | 68.743 | 64.755 | 49.914 | 28.816 | 26.698 | 33.083 |
| YJR016C | 0.194 | -2.363 | 5.92E-23 | 4.03E-21 | yes | down | 3046.582 | 5663.326 | 4027.616 | 817.451 | 736.547 | 821.335 |
| YKL151C | 0.194 | -2.368 | 3.2E-26 | 2.78E-24 | yes | down | 109.161 | 172.643 | 130.368 | 27.265 | 25.591 | 24.064 |
| YLL062C | 0.186 | -2.43 | 0.000147 | 0.001271 | yes | down | 0.866 | 2.967 | 3.63 | 0 | 0.369 | 0 |
| YML131W | 0.329 | -1.606 | 0.000188 | 0.001591 | yes | down | 102.723 | 60.815 | 68.26 | 6.225 | 34.212 | 27.413 |
| YGR122W | 0.49 | -1.03 | 0.000882 | 0.00606 | yes | down | 5.891 | 5.307 | 6.873 | 3.215 | 2.574 | 2.83 |
| YOR343W-A | 0.316 | -1.663 | 1.24E-07 | 2.17E-06 | yes | down | 25.808 | 53.698 | 27.187 | 8.197 | 10.486 | 13.152 |
| YOR373W | 0.468 | -1.095 | 8.49E-06 | 0.000103 | yes | down | 17.181 | 29.071 | 19.038 | 10.225 | 8.981 | 11.024 |
| YJR026W | 0.459 | -1.125 | 0.001903 | 0.01147 | yes | down | 19.461 | 17.916 | 9.425 | 5.113 | 5.044 | 10.292 |
| YDR119W-A | 0.193 | -2.371 | 7.08E-07 | 1.08E-05 | yes | down | 428.474 | 1397.362 | 1109.86 | 145.24 | 158.674 | 88.653 |
| YBR218C | 0.402 | -1.314 | 1.57E-06 | 2.22E-05 | yes | down | 156.51 | 92.878 | 154.628 | 57.081 | 41.244 | 61.545 |
| YNR058W | 0.281 | -1.833 | 1.48E-08 | 3.09E-07 | yes | down | 237.929 | 100.82 | 109.125 | 34.611 | 40.837 | 43.059 |
| YDR107C | 0.486 | -1.041 | 0.000101 | 0.000933 | yes | down | 5.435 | 6.354 | 4.549 | 2.393 | 2.404 | 3.064 |
| YJL219W | 0.386 | -1.375 | 2.95E-06 | 3.95E-05 | yes | down | 6.511 | 8.151 | 5.439 | 2.393 | 3.047 | 2.016 |
| YDR226W | 0.476 | -1.072 | 2.07E-06 | 2.86E-05 | yes | down | 1028.386 | 1241.612 | 823.143 | 369.703 | 523.436 | 565.798 |
| YER181C | 0.37 | -1.436 | 0.02737 | 0.09735 | yes | down | 6.894 | 13.298 | 10.681 | 0 | 0.861 | 3.95 |
| YDL085W | 0.205 | -2.287 | 5.9E-20 | 3.3E-18 | yes | down | 22.498 | 21.856 | 20.245 | 5.3 | 3.606 | 3.726 |
| YBR038W | 0.463 | -1.11 | 0.000224 | 0.001862 | yes | down | 4.35 | 4.26 | 2.73 | 1.851 | 1.874 | 1.405 |
| YDL218W | 0.186 | -2.423 | 0.000222 | 0.001843 | yes | down | 0.894 | 1.33 | 0.623 | 0 | 0 | 0 |
| YGR248W | 0.471 | -1.087 | 7.99E-05 | 0.000771 | yes | down | 51.042 | 99.983 | 65.204 | 33.125 | 36.56 | 29.47 |
| YGL234W | 0.369 | -1.437 | 2.95E-07 | 4.94E-06 | yes | down | 672.969 | 1314.95 | 1019.674 | 296.115 | 303.435 | 481.227 |
| YAR010C | 0.378 | -1.402 | 4.11E-08 | 7.7E-07 | yes | down | 41.494 | 30.143 | 24.082 | 10.973 | 12.587 | 12.114 |
| YER159C-A | 0.495 | -1.014 | 1.6E-05 | 0.000183 | yes | down | 109.307 | 138.979 | 82.244 | 48.762 | 50.254 | 63.917 |
| YAL012W | 0.159 | -2.654 | 6.55E-45 | 1.85E-42 | yes | down | 687.488 | 626.482 | 492.04 | 91.337 | 92.104 | 98.039 |
| YIR017C | 0.01 | -6.575 | 1.48E-34 | 1.85E-32 | yes | down | 28.936 | 63.708 | 54.236 | 0 | 0 | 0 |
| YDR365W-A | 0.483 | -1.051 | 4.7E-07 | 7.49E-06 | yes | down | 68.743 | 64.755 | 49.914 | 28.816 | 26.698 | 33.083 |
| YJL172W | 0.427 | -1.228 | 3.59E-10 | 9.08E-09 | yes | down | 72.874 | 83.496 | 71.375 | 36.798 | 28.525 | 31.658 |
| YPR001W | 0.187 | -2.418 | 3.79E-33 | 4.37E-31 | yes | down | 26.492 | 30.918 | 27.86 | 4.907 | 5.849 | 4.896 |
| YMR250W | 0.003 | -8.601 | 2.17E-72 | 2.45E-69 | yes | down | 31.298 | 44.845 | 43.436 | 0 | 0 | 0 |
| YBR138C | 0.315 | -1.666 | 1.95E-05 | 0.00022 | yes | down | 2.873 | 3.608 | 2.482 | 1.084 | 0.795 | 0.641 |
| YIR036W-A | 0.262 | -1.93 | 0.000121 | 0.001089 | yes | down | 16.789 | 20.588 | 16.645 | 1.243 | 4.741 | 4.795 |
| YHR106W | 0.378 | -1.405 | 3.88E-08 | 7.42E-07 | yes | down | 51.042 | 81.132 | 51.724 | 21.694 | 27.105 | 18.985 |
| YIL160C | 0.429 | -1.222 | 6.68E-05 | 0.000657 | yes | down | 19.625 | 26.793 | 21.085 | 12.562 | 6.246 | 8.815 |
| YER152W-A | 0.03 | -5.07 | 1.45E-17 | 7.12E-16 | yes | down | 12.986 | 37.715 | 9.712 | 0 | 0 | 0 |
| YOL065C | 0.447 | -1.162 | 0.000198 | 0.001669 | yes | down | 7.287 | 6.686 | 5.37 | 2.43 | 2.678 | 3.298 |
| YCR088W | 0.416 | -1.264 | 1.07E-11 | 3.11E-10 | yes | down | 38.384 | 52.109 | 46.453 | 18.684 | 18.72 | 19.748 |
| YNL117W | 0.156 | -2.683 | 6.14E-28 | 5.59E-26 | yes | down | 34.691 | 42.998 | 54.266 | 7.319 | 5.934 | 6.271 |
| YPL262W | 0.383 | -1.383 | 4.57E-10 | 1.15E-08 | yes | down | 251.819 | 186.754 | 226.468 | 98.207 | 78.873 | 75.42 |
| YDR032C | 0.123 | -3.025 | 3.37E-47 | 1.06E-44 | yes | down | 362.53 | 336.285 | 254.525 | 37.583 | 39.228 | 34.621 |
| YPR128C | 0.277 | -1.854 | 3.71E-12 | 1.12E-10 | yes | down | 29.675 | 22.878 | 19.948 | 5.253 | 6.928 | 7.207 |
| YLR011W | 0.441 | -1.182 | 0.01499 | 0.06153 | yes | down | 3.985 | 2.512 | 2.561 | 0.963 | 1.003 | 1.669 |
| YOR161C | 0.397 | -1.332 | 0.000212 | 0.001778 | yes | down | 49.921 | 179.539 | 90.68 | 39.64 | 41.935 | 37.043 |
| YOR086C | 0.419 | -1.254 | 0.000757 | 0.005348 | yes | down | 1.113 | 0.825 | 1.058 | 0.336 | 0.435 | 0.428 |
| YIR007W | 0.313 | -1.675 | 1.32E-05 | 0.000153 | yes | down | 2.262 | 3.817 | 3.788 | 1.103 | 0.539 | 1.13 |
| YBR117C | 0.078 | -3.685 | 2.41E-52 | 9.07E-50 | yes | down | 35.667 | 35.696 | 26.92 | 2.215 | 2.972 | 1.954 |
| YBR241C | 0.402 | -1.313 | 0.000307 | 0.002464 | yes | down | 75.373 | 253.097 | 120.983 | 63.072 | 61.223 | 43.752 |
| YJL212C | 0.209 | -2.258 | 1.1E-12 | 3.62E-11 | yes | down | 45.798 | 28.887 | 36.078 | 9.048 | 4.268 | 7.981 |
| YOL163W | 0.311 | -1.687 | 2.31E-06 | 3.15E-05 | yes | down | 31.225 | 46.298 | 41.824 | 13.917 | 13.022 | 6.383 |
| YLL061W | 0.371 | -1.43 | 6.74E-06 | 8.4E-05 | yes | down | 39.542 | 69.816 | 54.602 | 25.863 | 19.572 | 12.073 |
| YPR137C-A | 0.378 | -1.402 | 4.11E-08 | 7.7E-07 | yes | down | 41.494 | 30.143 | 24.082 | 10.973 | 12.587 | 12.114 |
| YOR090C | 0.495 | -1.015 | 1.21E-06 | 1.75E-05 | yes | down | 17.437 | 21.08 | 21.303 | 10.263 | 8.688 | 10.668 |
| YPL223C | 0.004 | -8.136 | 3.62E-59 | 2.27E-56 | yes | down | 147.655 | 329.353 | 189.688 | 0 | 0 | 0 |
| YBR285W | 0.304 | -1.718 | 0.000509 | 0.003811 | yes | down | 10.25 | 22.324 | 10.167 | 3.019 | 4.145 | 2.871 |
| YLR164W | 0.235 | -2.09 | 1.91E-09 | 4.44E-08 | yes | down | 27.578 | 41.434 | 24.942 | 5.047 | 7.912 | 6.688 |
| YPR158W-A | 0.459 | -1.124 | 1.06E-06 | 1.55E-05 | yes | down | 24.878 | 36.927 | 24.972 | 11.945 | 14.546 | 13.05 |
| YOR142W-A | 0.459 | -1.124 | 1.06E-06 | 1.55E-05 | yes | down | 24.878 | 36.927 | 24.972 | 11.945 | 14.546 | 13.05 |
| YIR032C | 0.378 | -1.405 | 8.65E-06 | 0.000104 | yes | down | 910.479 | 1992.125 | 1153.633 | 618.991 | 435.818 | 377.66 |
| YLR157C-A | 0.483 | -1.051 | 4.7E-07 | 7.49E-06 | yes | down | 68.743 | 64.755 | 49.914 | 28.816 | 26.698 | 33.083 |
| YOL103W-A | 0.451 | -1.148 | 9.12E-05 | 0.000865 | yes | down | 77.115 | 74.569 | 35.969 | 23.657 | 28.723 | 30.406 |
| YPL274W | 0.007 | -7.188 | 6.94E-44 | 1.78E-41 | yes | down | 19.817 | 12.055 | 11.027 | 0 | 0 | 0 |
| YBL080C | 0.438 | -1.19 | 0.0117 | 0.05087 | yes | down | 2.325 | 1.219 | 1.582 | 0.355 | 0.927 | 0.733 |
| YGR286C | 0.326 | -1.619 | 1.26E-12 | 4.15E-11 | yes | down | 603.214 | 435.985 | 365.44 | 140.734 | 147.8 | 162.363 |
| YML027W | 0.378 | -1.403 | 3.34E-06 | 4.41E-05 | yes | down | 12.594 | 20.452 | 12.56 | 5.879 | 6.322 | 4.286 |
| YOL153C | 0.067 | -3.9 | 3.9E-10 | 9.83E-09 | yes | down | 0.803 | 1.822 | 1.108 | 0 | 0 | 0 |
| YPL004C | 0.281 | -1.83 | 1.57E-16 | 7.08E-15 | yes | down | 203.458 | 314.614 | 311.897 | 69.68 | 73.63 | 85.549 |
| YJR156C | 0.338 | -1.565 | 2.27E-05 | 0.000254 | yes | down | 5.435 | 5.492 | 5.261 | 2.159 | 1.543 | 1.293 |
| YBR214W | 0.414 | -1.274 | 7.09E-05 | 0.000695 | yes | down | 47.486 | 130.187 | 82.116 | 37.462 | 35.367 | 29.582 |
| YEL035C | 0.481 | -1.055 | 0.004222 | 0.02174 | yes | down | 16.26 | 28.049 | 19.147 | 9.721 | 7.145 | 11.442 |

Opposite DEGs early treatment

| Gene_id | FC(NaHS/Control) | Log2FC(NaHS/Control) | Pvalue | Padjust | Significant | Regulate | Control1_Fpkm | Control2_Fpkm | Control3_Fpkm | NaHS1_Fpkm | NaHS2_Fpkm | NaHS3_Fpkm |
| --- | --- | --- | --- | --- | --- | --- | --- | --- | --- | --- | --- | --- |
| YBL042C | 0.389 | -1.36304 | 1.21E-17 | 4.35E-16 | yes | down | 26.39 | 28.1 | 25.71 | 10.89 | 9.02 | 9.95 |
| YBR208C | 0.145 | -2.7822 | 4.71E-38 | 8.79E-36 | yes | down | 8.73 | 9.43 | 12.84 | 1.5 | 1.41 | 1.19 |
| YBR296C | 0.297 | -1.75129 | 2.02E-11 | 3.57E-10 | yes | down | 16.16 | 25.51 | 14.58 | 4.84 | 5.15 | 5.24 |
| YDL049C | 0.411 | -1.28424 | 4.12E-08 | 4.1E-07 | yes | down | 23.47 | 22.18 | 29.32 | 10.97 | 10.47 | 7.32 |
| YDR033W | 0.459 | -1.12262 | 8.79E-07 | 6.85E-06 | yes | down | 122.96 | 114.97 | 139.6 | 44.67 | 47.27 | 70.14 |
| YDR380W | 0.437 | -1.19338 | 2.17E-06 | 1.54E-05 | yes | down | 27.24 | 33.09 | 42.69 | 11.09 | 13.25 | 17.53 |
| YGL162W | 0.339 | -1.56124 | 0.000232 | 0.001013 | yes | down | 169.85 | 223.44 | 333.32 | 92.73 | 85.87 | 17.96 |
| YGR052W | 0.435 | -1.20007 | 0.000515 | 0.002044 | yes | down | 196.79 | 203.92 | 261.12 | 104.23 | 117.74 | 35.92 |
| YGR260W | 0.356 | -1.48987 | 2.34E-05 | 0.000131 | yes | down | 462.43 | 480.56 | 444.54 | 181.9 | 193.74 | 55.81 |
| YGR279C | 0.436 | -1.19868 | 3.59E-09 | 4.52E-08 | yes | down | 58.93 | 66.73 | 71.76 | 34.28 | 24.57 | 22.77 |
| YHL040C | 0.394 | -1.34317 | 3.27E-09 | 4.15E-08 | yes | down | 174.3 | 191.05 | 203.04 | 84.65 | 79.31 | 47.19 |
| YHR137W | 0.226 | -2.14622 | 2.02E-19 | 8.38E-18 | yes | down | 151.11 | 175.93 | 232 | 40.47 | 46.77 | 28.03 |
| YHR153C | 0.486 | -1.04178 | 0.018627 | 0.045556 | yes | down | 3.83 | 4.48 | 4.7 | 2.77 | 1.56 | 0.92 |
| YKL096W | 0.259 | -1.94836 | 0.000275 | 0.001185 | yes | down | 1014.81 | 889.43 | 1468.85 | 168.61 | 255.13 | 31.72 |
| YKR052C | 0.21 | -2.25349 | 3.96E-13 | 8.85E-12 | yes | down | 495.9 | 480.3 | 545.45 | 108.67 | 121.04 | 43.7 |
| YLL052C | 0.278 | -1.84878 | 2.7E-08 | 2.83E-07 | yes | down | 57.8 | 41.32 | 66.91 | 15.08 | 17.24 | 6.63 |
| YLL053C | 0.333 | -1.58819 | 1.75E-07 | 1.56E-06 | yes | down | 83.99 | 68.12 | 97.22 | 33.8 | 25.57 | 13.62 |
| YLR213C | 0.425 | -1.23436 | 2.61E-06 | 1.82E-05 | yes | down | 8.57 | 9.43 | 7.1 | 3.91 | 3.59 | 2.44 |
| YOR382W | 0.38 | -1.39571 | 0.000173 | 0.000778 | yes | down | 3244.81 | 3269.72 | 4086.95 | 1837.31 | 1155.79 | 433.98 |
| LSR1 | 2.051 | 1.036039 | 5.65E-07 | 4.6E-06 | yes | up | 490.91 | 509.64 | 463.96 | 938.36 | 806.92 | 1238.37 |
| RDN25-1 | 2.318 | 1.212823 | 4.69E-05 | 0.000244 | yes | up | 2789.23 | 3856.38 | 2364.87 | 6219.43 | 5153.67 | 10281.97 |
| RDN25-2 | 2.523 | 1.335199 | 4.55E-06 | 3E-05 | yes | up | 2454.45 | 3309.54 | 2278.22 | 5856.18 | 5085.91 | 10144.52 |
| YBR104W | 3.267 | 1.707773 | 3.88E-14 | 9.81E-13 | yes | up | 44.27 | 44.19 | 49.77 | 132.55 | 128.21 | 197.68 |
| YBR301W | 2.196 | 1.134837 | 0.028231 | 0.064216 | yes | up | 1.83 | 1.6 | 4.66 | 6.44 | 5.77 | 10.2 |
| YDL221W | 20.763 | 4.375947 | 2.12E-16 | 6.79E-15 | yes | up | 0 | 0 | 0 | 13.16 | 19.67 | 6.23 |
| YGL184C | 2.633 | 1.396546 | 2.9E-06 | 2E-05 | yes | up | 63.6 | 59.47 | 64.58 | 130.49 | 121.81 | 261.85 |
| YHR207C | 2.655 | 1.408504 | 1.13E-05 | 6.81E-05 | yes | up | 5.47 | 4.15 | 6 | 10 | 12.02 | 21.74 |
| YMR168C | 2.388 | 1.255743 | 2.28E-09 | 2.97E-08 | yes | up | 3.91 | 4.99 | 4.07 | 9.31 | 11.87 | 10.05 |
| YMR316W | 2.029 | 1.020792 | 0.00036 | 0.001493 | yes | up | 114.96 | 110.02 | 89.46 | 159.78 | 171.89 | 315.34 |
| YMR320W | 2.021 | 1.015214 | 0.000902 | 0.003367 | yes | up | 79.07 | 60 | 90.7 | 136.47 | 109.89 | 205.05 |
| YOL016C | 2.295 | 1.198655 | 0.000151 | 0.00069 | yes | up | 137.77 | 143.7 | 130.91 | 237.55 | 224.96 | 520.86 |
| YOR220W | 2.135 | 1.094524 | 3.65E-05 | 0.000195 | yes | up | 2908.06 | 2706.46 | 2717.98 | 4679.37 | 4688.54 | 8539.88 |
| YOR385W | 2.266 | 1.180044 | 0.000584 | 0.002297 | yes | up | 31.05 | 28.4 | 24.57 | 43.75 | 46.59 | 109.42 |
| YPR010C-A | 2.301 | 1.202503 | 6.72E-10 | 9.49E-09 | yes | up | 4214.46 | 5207.52 | 4850.92 | 9599.04 | 9535.07 | 10573.98 |
| YPR167C | 2.125 | 1.087274 | 0.000514 | 0.00204 | yes | up | 32.5 | 30.47 | 34.39 | 50.6 | 52 | 109.4 |
| YPR169W-A | 2.376 | 1.248719 | 0.005734 | 0.016678 | yes | up | 63.82 | 37.64 | 25.04 | 65.68 | 97.94 | 148.86 |

Opposite DEGs late treatment

| Gene_id | FC(NaHS/Control) | Log2FC(NaHS/Control) | Pvalue | Padjust | Significant | Regulate | Control1_Fpkm | Control2_Fpkm | Control3_Fpkm | NaHS1_Fpkm | NaHS2_Fpkm | NaHS3_Fpkm |
| --- | --- | --- | --- | --- | --- | --- | --- | --- | --- | --- | --- | --- |
| YBL042C | 2.723 | 1.445 | 1.08E-05 | 0.000128 | yes | up | 130.775 | 52.971 | 61.208 | 238.203 | 218.874 | 280.904 |
| YBR208C | 2.018 | 1.013 | 0.001588 | 0.00991 | yes | up | 147.965 | 60.569 | 119.4 | 279.366 | 236.78 | 198.134 |
| YBR296C | 7.89 | 2.98 | 1.15E-22 | 7.65E-21 | yes | up | 8.819 | 5.147 | 5.133 | 38.724 | 73.772 | 64.538 |
| YDL049C | 3.699 | 1.887 | 1.73E-09 | 4.05E-08 | yes | up | 11.618 | 9.481 | 7.586 | 27.695 | 34.837 | 55.56 |
| YDR033W | 9.164 | 3.196 | 5.11E-43 | 1.11E-40 | yes | up | 194.639 | 123.612 | 145.104 | 1269.031 | 1804.874 | 1617.547 |
| YDR380W | 195.361 | 7.61 | 2.53E-47 | 8.41E-45 | yes | up | 0 | 0 | 0 | 49.173 | 18.445 | 18.415 |
| YGL162W | 2.774 | 1.472 | 1.01E-15 | 4.22E-14 | yes | up | 28.982 | 26.51 | 31.489 | 78.831 | 81.949 | 90.598 |
| YGR052W | 2.053 | 1.038 | 0.005495 | 0.02694 | yes | up | 3.137 | 1.909 | 2.799 | 7.636 | 5.442 | 4.622 |
| YGR260W | 2.398 | 1.262 | 3.04E-08 | 6.01E-07 | yes | up | 423.038 | 553.354 | 349.399 | 927.612 | 1052.645 | 1364.993 |
| YGR279C | 2.388 | 1.256 | 4.2E-09 | 9.3E-08 | yes | up | 171.402 | 185.387 | 119.816 | 365.179 | 376.98 | 447.512 |
| YHL040C | 8.011 | 3.002 | 1.57E-44 | 4.22E-42 | yes | up | 150.436 | 229.235 | 190.805 | 1646.034 | 1887.012 | 1409.559 |
| YHR137W | 33.498 | 5.066 | 6.51E-35 | 8.55E-33 | yes | up | 32.019 | 7.314 | 27.593 | 1474.763 | 1210.912 | 893.08 |
| YHR153C | 2.504 | 1.324 | 0.001531 | 0.009605 | yes | up | 2.727 | 2.487 | 3.461 | 7.73 | 6.88 | 10.149 |
| YKL096W | 2.208 | 1.143 | 7.22E-05 | 0.000704 | yes | up | 22.452 | 45.83 | 26.406 | 63.558 | 72.589 | 84.144 |
| YKR052C | 152.008 | 7.248 | 2.03E-43 | 4.77E-41 | yes | up | 0 | 0 | 0 | 59.034 | 54.976 | 24.095 |
| YLL052C | 3.218 | 1.686 | 0.000143 | 0.001252 | yes | up | 8.198 | 27.939 | 14.924 | 32.125 | 75.873 | 88.725 |
| YLL053C | 2.352 | 1.234 | 0.000583 | 0.004296 | yes | up | 31.672 | 66.171 | 36.424 | 64.839 | 145.064 | 132.232 |
| YLR213C | 2.059 | 1.042 | 8.34E-07 | 1.25E-05 | yes | up | 7.496 | 8.053 | 7.289 | 14.983 | 16.06 | 17.947 |
| YOR382W | 5.517 | 2.464 | 1.63E-16 | 7.29E-15 | yes | up | 801.099 | 1863.354 | 1137.591 | 8493.177 | 7842.776 | 6683.012 |
| LSR1 | 0.426 | -1.232 | 0.00024 | 0.001967 | yes | down | 213.088 | 604.639 | 430.387 | 207.639 | 152.248 | 142.412 |
| RDN25-1 | 0.297 | -1.751 | 9.82E-05 | 0.000915 | yes | down | 905.81 | 3819.39 | 6831.243 | 936.435 | 1092.328 | 874.126 |
| RDN25-2 | 0.297 | -1.751 | 9.82E-05 | 0.000915 | yes | down | 905.81 | 3819.39 | 6831.243 | 936.435 | 1092.328 | 874.126 |
| YBR104W | 0.356 | -1.489 | 1.05E-12 | 3.52E-11 | yes | down | 78.364 | 59.091 | 65.125 | 23.834 | 25.837 | 21.978 |
| YBR301W | 0.228 | -2.136 | 7.77E-06 | 9.5E-05 | yes | down | 27.888 | 28.37 | 20.887 | 6.057 | 2.915 | 3.746 |
| YDL221W | 0.481 | -1.055 | 0.01816 | 0.07103 | yes | down | 12.539 | 36.755 | 12.214 | 10.59 | 7.571 | 8.021 |
| YGL184C | 0.347 | -1.529 | 1.68E-11 | 4.8E-10 | yes | down | 46.747 | 39.254 | 58.133 | 15.628 | 18.587 | 15.279 |
| YHR207C | 0.147 | -2.762 | 1.22E-39 | 2.15E-37 | yes | down | 130.811 | 214.483 | 168.029 | 24.76 | 23.565 | 24.237 |
| YMR168C | 0.353 | -1.501 | 1.25E-05 | 0.000146 | yes | down | 2.435 | 8.004 | 5.449 | 1.673 | 1.921 | 1.669 |
| YMR316W | 0.098 | -3.344 | 6.05E-19 | 3.13E-17 | yes | down | 290.987 | 145.862 | 186.671 | 7.403 | 25.354 | 17.804 |
| YMR320W | 0.488 | -1.035 | 0.04122 | 0.1332 | yes | down | 92.117 | 44.598 | 45.8 | 40.032 | 20.329 | 9.701 |
| YOL016C | 0.245 | -2.028 | 2.37E-12 | 7.52E-11 | yes | down | 337.78 | 160.884 | 232.155 | 44.537 | 61.563 | 65.23 |
| YOR220W | 0.389 | -1.363 | 3.42E-11 | 9.34E-10 | yes | down | 901.679 | 1269.058 | 957.348 | 378.862 | 471.971 | 353.789 |
| YOR385W | 0.171 | -2.547 | 4.97E-05 | 0.000501 | yes | down | 131.805 | 68.19 | 82.62 | 0.589 | 7.164 | 9.742 |
| YPR010C-A | 0.457 | -1.129 | 0.001545 | 0.009674 | yes | down | 775.145 | 1408.961 | 891.7 | 431.784 | 511.682 | 302.912 |
| YPR167C | 0.338 | -1.565 | 1.16E-08 | 2.45E-07 | yes | down | 113.101 | 59.608 | 88.188 | 24.461 | 32.197 | 28.92 |
| YPR169W-A | 0.256 | -1.968 | 3.88E-05 | 0.000406 | yes | down | 238.039 | 282.07 | 259.075 | 21.049 | 78.419 | 49.055 |
