# Supplementary material for: Hydrogen sulfide treatment at the late growth stage of Saccharomyces cerevisiae extends chronological lifespan
Source: Aging (Albany NY). 2021 Mar 19;13(7):9859–73. doi: 10.18632/aging.202738 (PMC8064171; doi:10.18632/aging.202738)
Supplement: Supplementary Table 4 [file aging-13-202738-s005.doc]

**Supplementary Table 4. Specific DEGs induced by the early NaHS treatment and the expression of these genes in the late NaHS treatment.**

Spesific DEGs early treatment

| Gene_id | FC(NaHS/Control) | Log2FC(NaHS/Control) | Pvalue | Padjust | Significant | Regulate | Control1_Fpkm | Control2_Fpkm | Control3_Fpkm | NaHS1_Fpkm | NaHS2_Fpkm | NaHS3_Fpkm |
| --- | --- | --- | --- | --- | --- | --- | --- | --- | --- | --- | --- | --- |
| YKR071C | 4.631 | 2.211279 | 6.09E-57 | 4.83E-54 | yes | up | 309.86 | 245.65 | 319.35 | 1127.03 | 1001.56 | 1273.05 |
| YPL106C | 4.579 | 2.195139 | 1.51E-51 | 7.97E-49 | yes | up | 204.93 | 205.68 | 221.51 | 975.64 | 866.8 | 1047.23 |
| YOR204W | 4.147 | 2.052141 | 6.29E-46 | 2E-43 | yes | up | 248.37 | 234.26 | 245.53 | 962.53 | 933.26 | 1110.67 |
| RUF5-2 | 15.023 | 3.909083 | 6.29E-45 | 1.9E-42 | yes | up | 1463.24 | 1289.42 | 1307.1 | 26947.99 | 30955.38 | 13111.4 |
| YPR154W | 3.14 | 1.650921 | 2.94E-43 | 7.78E-41 | yes | up | 887.71 | 877.86 | 873.53 | 2646.71 | 2629.39 | 2825.22 |
| YHR054C | 11.256 | 3.49261 | 4.29E-39 | 9.08E-37 | yes | up | 773.43 | 743.31 | 723.93 | 11458.8 | 12778.67 | 5764.22 |
| YHR052W-A | 13.981 | 3.805353 | 6.08E-39 | 1.25E-36 | yes | up | 12.33 | 5.52 | 10.51 | 177.38 | 178.3 | 112.38 |
| YPL239W | 4.587 | 2.197683 | 1.38E-36 | 2.36E-34 | yes | up | 23.51 | 20.43 | 24.81 | 97.11 | 113.55 | 104.36 |
| YNL064C | 4.111 | 2.039646 | 9.28E-36 | 1.51E-33 | yes | up | 78.21 | 71.39 | 72.15 | 272.84 | 293.07 | 345.68 |
| YPL026C | 4.556 | 2.187816 | 1.35E-34 | 2.09E-32 | yes | up | 12.55 | 12.66 | 13 | 69.22 | 50.8 | 56.2 |
| YPL151C | 3.101 | 1.63289 | 6.88E-34 | 9.92E-32 | yes | up | 44.79 | 46.03 | 49.73 | 138.68 | 158.2 | 134.68 |
| RUF5-1 | 12.664 | 3.662623 | 1.1E-32 | 1.52E-30 | yes | up | 1439.91 | 1673.22 | 1697.05 | 29106.23 | 32335.88 | 12053.25 |
| YHR055C | 15.183 | 3.924412 | 7.93E-31 | 9.86E-29 | yes | up | 2546.33 | 4771.31 | 2030.68 | 62502.73 | 64744.93 | 34272.64 |
| YOR011W | 2.507 | 1.326094 | 1.86E-26 | 1.59E-24 | yes | up | 43.64 | 45.11 | 44.26 | 117.68 | 123.94 | 136.3 |
| YMR161W | 2.776 | 1.472954 | 7.26E-26 | 5.98E-24 | yes | up | 79.59 | 87.13 | 86.02 | 224.31 | 252.14 | 213.67 |
| YGL058W | 3.773 | 1.915681 | 1.94E-25 | 1.48E-23 | yes | up | 64.97 | 64.76 | 69.49 | 221.94 | 235.9 | 288 |
| YML081W | 2.707 | 1.436888 | 3.56E-25 | 2.6E-23 | yes | up | 20.74 | 19.21 | 21.57 | 51.24 | 55.11 | 58.48 |
| YGR142W | 10.406 | 3.379363 | 4.64E-25 | 3.31E-23 | yes | up | 38.79 | 35.08 | 38.48 | 303.14 | 347.23 | 761.46 |
| YMR295C | 2.737 | 1.452566 | 5.99E-25 | 4.22E-23 | yes | up | 964.81 | 991.85 | 992.55 | 2581.33 | 2445.78 | 2866.48 |
| YGR210C | 2.512 | 1.329019 | 1.83E-24 | 1.26E-22 | yes | up | 128.49 | 123.48 | 142.8 | 308.69 | 345.14 | 322.73 |
| YLR259C | 2.428 | 1.279906 | 4.66E-24 | 3.08E-22 | yes | up | 1077.37 | 987.27 | 990.76 | 2345.13 | 2389.45 | 2569.09 |
| YKL156W | 3.04 | 1.60402 | 1.38E-23 | 8.86E-22 | yes | up | 855.15 | 782.58 | 807.11 | 2227.15 | 2125.94 | 2477 |
| YHR053C | 11.301 | 3.498423 | 4.17E-23 | 2.59E-21 | yes | up | 5911.88 | 1996.99 | 4102.87 | 52452.45 | 68558.7 | 26181.01 |
| YNL074C | 2.565 | 1.358989 | 2.8E-22 | 1.6E-20 | yes | up | 395.45 | 337.86 | 354.09 | 884.45 | 885.35 | 980.79 |
| YOR020C | 2.701 | 1.433577 | 4.91E-22 | 2.74E-20 | yes | up | 1826.47 | 1726.75 | 1665.06 | 4235.47 | 4202.26 | 4887.03 |
| YOR208W | 2.643 | 1.402337 | 8.07E-22 | 4.38E-20 | yes | up | 17.61 | 14.79 | 17.78 | 46.89 | 42.39 | 42.17 |
| YER150W | 3.055 | 1.611323 | 1.75E-21 | 9.11E-20 | yes | up | 10664.53 | 9832.02 | 9352.38 | 26959.86 | 27627.15 | 34343.7 |
| YOR338W | 5.112 | 2.353985 | 2.25E-21 | 1.15E-19 | yes | up | 100.49 | 86.94 | 93.57 | 433.5 | 388.71 | 685.82 |
| YKL009W | 4.388 | 2.133566 | 4E-21 | 2.01E-19 | yes | up | 15.88 | 13.47 | 15.15 | 62.42 | 56.05 | 81.53 |
| YNL007C | 4.313 | 2.108784 | 4.03E-21 | 2.02E-19 | yes | up | 494.47 | 415.43 | 417.35 | 1638.47 | 1644.69 | 2531.36 |
| YJR046W | 2.956 | 1.563633 | 4.52E-21 | 2.21E-19 | yes | up | 111.47 | 104.85 | 91.59 | 311.99 | 344.32 | 254.53 |
| YPL169C | 2.258 | 1.174916 | 5.38E-20 | 2.35E-18 | yes | up | 40.34 | 37.75 | 42.53 | 84.95 | 93.89 | 89.3 |
| YPR145W | 2.454 | 1.294974 | 6.74E-20 | 2.91E-18 | yes | up | 167.17 | 173.58 | 198.3 | 448.22 | 406.2 | 451.67 |
| YNL124W | 3.123 | 1.64276 | 1.32E-19 | 5.52E-18 | yes | up | 52.02 | 48.23 | 50.52 | 145.9 | 138.5 | 186.43 |
| YMR256C | 3.148 | 1.654255 | 2.74E-19 | 1.1E-17 | yes | up | 4864.24 | 4850.82 | 4930.05 | 12521.33 | 11981.73 | 14539.13 |
| YPL213W | 2.651 | 1.406802 | 2.82E-19 | 1.13E-17 | yes | up | 27.67 | 27.27 | 25.99 | 66.47 | 72.9 | 72.16 |
| YBR118W | 2.027 | 1.019126 | 1.87E-18 | 7.14E-17 | yes | up | 12870.19 | 12896.18 | 14188.46 | 26935.24 | 25777.18 | 26692.12 |
| YIL130W | 2.844 | 1.508013 | 3.2E-18 | 1.2E-16 | yes | up | 5.97 | 5.15 | 6.87 | 17.38 | 16.05 | 17.69 |
| YBL033C | 2.183 | 1.126634 | 9.27E-18 | 3.38E-16 | yes | up | 1158.78 | 1111.35 | 1097.53 | 2458.65 | 2215.46 | 2539.66 |
| YML130C | 4.531 | 2.17973 | 9.4E-18 | 3.41E-16 | yes | up | 12.97 | 11.35 | 14.35 | 48.73 | 52.25 | 83.05 |
| YOL067C | 2.336 | 1.224034 | 2.06E-17 | 7.29E-16 | yes | up | 79.08 | 67.91 | 79.44 | 169.34 | 181.45 | 164.43 |
| YMR294W-A | 2.883 | 1.527623 | 2.43E-17 | 8.5E-16 | yes | up | 532.79 | 540.83 | 492.88 | 1366.81 | 1296.99 | 1690.73 |
| YCR008W | 2.575 | 1.364605 | 1.06E-16 | 3.52E-15 | yes | up | 68.22 | 55.83 | 60.23 | 145.02 | 146.5 | 175.17 |
| YOR007C | 2.625 | 1.392113 | 1.58E-16 | 5.11E-15 | yes | up | 279.19 | 270.14 | 270.86 | 619.49 | 689.5 | 823.43 |
| YKR062W | 2.163 | 1.113255 | 1.75E-16 | 5.63E-15 | yes | up | 72.76 | 83.52 | 73.86 | 164.02 | 165.54 | 159.96 |
| YNL157W | 2.99 | 1.580236 | 4.31E-16 | 1.34E-14 | yes | up | 1035.74 | 1087.53 | 1014.39 | 2840.22 | 2664.12 | 3768.5 |
| YKL031W | 2.113 | 1.079524 | 5.24E-16 | 1.62E-14 | yes | up | 50.75 | 46.53 | 49.92 | 98.51 | 99.06 | 108.46 |
| YBR155W | 2.962 | 1.566774 | 9.16E-16 | 2.72E-14 | yes | up | 7.04 | 6.08 | 6.56 | 21.16 | 17.55 | 19.82 |
| YER147C-A | 2.703 | 1.434826 | 2.39E-15 | 6.9E-14 | yes | up | 96.93 | 85.05 | 83.64 | 246.12 | 200.44 | 251.23 |
| YFR040W | 2.39 | 1.257294 | 3.19E-15 | 9.08E-14 | yes | up | 16.38 | 14.42 | 14.84 | 34.09 | 33.94 | 40.12 |
| YDR186C | 2.075 | 1.052937 | 3.77E-15 | 1.06E-13 | yes | up | 44.48 | 36.71 | 41.56 | 83.66 | 83.09 | 84.14 |
| YJL217W | 2.061 | 1.043014 | 6.97E-15 | 1.92E-13 | yes | up | 713.24 | 680.7 | 724.14 | 1574.33 | 1448.08 | 1236.47 |
| YER148W | 2.615 | 1.387016 | 1.25E-14 | 3.37E-13 | yes | up | 100.86 | 85.28 | 92.14 | 217.21 | 222.73 | 279.53 |
| YPR190C | 2.917 | 1.544343 | 1.68E-14 | 4.44E-13 | yes | up | 17.4 | 13.48 | 14.9 | 54.81 | 44.05 | 36.24 |
| YBR065C | 3.889 | 1.959513 | 3.56E-14 | 9.07E-13 | yes | up | 27.81 | 21.44 | 23.39 | 79.2 | 81.38 | 133.82 |
| YDR217C | 4.063 | 2.022684 | 4.56E-14 | 1.14E-12 | yes | up | 0.67 | 0.59 | 1.01 | 2.8 | 3.39 | 3.56 |
| YMR272C | 2.21 | 1.143767 | 9.55E-14 | 2.28E-12 | yes | up | 846.85 | 767.25 | 872.42 | 1772.55 | 1631.45 | 2005.83 |
| YDR151C | 2.281 | 1.189802 | 1.54E-13 | 3.54E-12 | yes | up | 50.14 | 50.37 | 50.22 | 101.55 | 110.95 | 126.54 |
| YER104W | 2.108 | 1.075644 | 2.14E-13 | 4.89E-12 | yes | up | 90 | 89.36 | 81.91 | 168.36 | 180.08 | 189.42 |
| YNR075W | 2.785 | 1.477442 | 3.12E-13 | 7.05E-12 | yes | up | 16.18 | 13.26 | 16.4 | 39.85 | 37.9 | 50.11 |
| YMR043W | 2.798 | 1.484529 | 4.79E-13 | 1.07E-11 | yes | up | 181.08 | 139.49 | 144.46 | 387.2 | 388.83 | 525.35 |
| YPL055C | 2.342 | 1.227663 | 5.9E-13 | 1.3E-11 | yes | up | 619.62 | 560.02 | 570.63 | 1253.52 | 1220.56 | 1574.33 |
| YOR344C | 3.591 | 1.844321 | 7.66E-13 | 1.66E-11 | yes | up | 3.77 | 4.36 | 4.87 | 14.59 | 14.84 | 19.05 |
| YBR105C | 2.101 | 1.070762 | 1.02E-12 | 2.19E-11 | yes | up | 844.39 | 869.48 | 760.98 | 1917.56 | 1744.36 | 1471.45 |
| YKR086W | 2.151 | 1.104951 | 1.13E-12 | 2.4E-11 | yes | up | 7.18 | 6.25 | 7.38 | 14.06 | 14.7 | 15.49 |
| YOR213C | 2.163 | 1.112827 | 1.23E-12 | 2.61E-11 | yes | up | 81.2 | 92.72 | 72.83 | 175.33 | 182.86 | 167.32 |
| YML076C | 2.644 | 1.402909 | 1.23E-12 | 2.61E-11 | yes | up | 25.5 | 22.82 | 27.66 | 57.56 | 62.49 | 81.31 |
| YNL281W | 3.091 | 1.6283 | 1.4E-12 | 2.92E-11 | yes | up | 166.17 | 151.93 | 155.48 | 416.13 | 407.61 | 636.35 |
| YPR153W | 2.412 | 1.269999 | 1.55E-12 | 3.2E-11 | yes | up | 60.74 | 65.9 | 61.48 | 162.23 | 156.48 | 124.64 |
| YOL047C | 2.024 | 1.017275 | 2.07E-12 | 4.22E-11 | yes | up | 16.41 | 21.35 | 17.44 | 52.21 | 47.49 | 47.89 |
| YMR186W | 2.751 | 1.460195 | 2.56E-12 | 5.1E-11 | yes | up | 179.78 | 162.02 | 178.82 | 405.01 | 427.96 | 608.07 |
| YML118W | 2.905 | 1.538668 | 2.6E-12 | 5.16E-11 | yes | up | 50.87 | 43.67 | 40.64 | 113.59 | 116.17 | 167.46 |
| YOR027W | 3.471 | 1.795216 | 3.33E-12 | 6.48E-11 | yes | up | 309.94 | 266.61 | 265.61 | 799.5 | 819.32 | 1414.61 |
| YMR115W | 2.191 | 1.131313 | 3.53E-12 | 6.85E-11 | yes | up | 32.36 | 35.94 | 34.52 | 69 | 70.41 | 83.23 |
| YBR025C | 2.285 | 1.192459 | 3.56E-12 | 6.89E-11 | yes | up | 71.2 | 66.34 | 83.01 | 155.76 | 155.94 | 186.77 |
| YBR247C | 2.283 | 1.190904 | 4.26E-12 | 8.21E-11 | yes | up | 15.37 | 11.56 | 14.8 | 30.94 | 31.48 | 31.98 |
| YDL070W | 2.822 | 1.496793 | 4.31E-12 | 8.3E-11 | yes | up | 142.41 | 124.31 | 139.44 | 344.75 | 318.62 | 493.23 |
| YER052C | 2.389 | 1.256459 | 7.49E-12 | 1.39E-10 | yes | up | 68.55 | 66.37 | 81.47 | 147.42 | 169.49 | 197.15 |
| YDR433W | 2.335 | 1.223144 | 7.9E-12 | 1.46E-10 | yes | up | 466 | 377.26 | 413.86 | 971.06 | 805.21 | 1070.18 |
| YKL032C | 2.313 | 1.209609 | 1.19E-11 | 2.16E-10 | yes | up | 184.72 | 160.37 | 165.28 | 360.33 | 351.25 | 459.93 |
| YDR524C-B | 2.091 | 1.064282 | 1.19E-11 | 2.16E-10 | yes | up | 7413.76 | 7436.64 | 8108.47 | 15337.37 | 14003.54 | 12490.59 |
| YFR033C | 2.224 | 1.153304 | 2.24E-11 | 3.96E-10 | yes | up | 1640.99 | 1392.44 | 1600.96 | 3100.5 | 3025.59 | 3852.01 |
| YNR010W | 2.472 | 1.305956 | 2.73E-11 | 4.77E-10 | yes | up | 91.93 | 62.14 | 70.98 | 172.03 | 175.09 | 196.19 |
| YOR031W | 2.768 | 1.468646 | 2.86E-11 | 4.98E-10 | yes | up | 3951.4 | 3425.62 | 3377.35 | 8019.28 | 7369.05 | 11160.78 |
| YBR114W | 2.117 | 1.081859 | 3.84E-11 | 6.6E-10 | yes | up | 53.79 | 55.73 | 53.49 | 106.77 | 103.85 | 130.65 |
| YEL026W | 2.901 | 1.536499 | 4.2E-11 | 7.19E-10 | yes | up | 85.12 | 86.68 | 107.08 | 265.13 | 212.33 | 321.59 |
| YKR008W | 2.712 | 1.439522 | 4.56E-11 | 7.78E-10 | yes | up | 5.5 | 5.29 | 6.81 | 14.54 | 14.45 | 19.19 |
| YPR045C | 2.264 | 1.178661 | 5.19E-11 | 8.79E-10 | yes | up | 19.41 | 18.39 | 23.32 | 41.6 | 44.9 | 50.73 |
| YIL117C | 2.095 | 1.066854 | 5.69E-11 | 9.58E-10 | yes | up | 75.62 | 71.01 | 73.27 | 162.63 | 169.88 | 123.07 |
| YOR327C | 2.403 | 1.264934 | 8.03E-11 | 1.33E-09 | yes | up | 772.47 | 792.1 | 746.86 | 1581.94 | 1602.64 | 2149.58 |
| YOR298C-A | 2.616 | 1.387188 | 8.52E-11 | 1.4E-09 | yes | up | 2452.86 | 2369.13 | 2376.81 | 5395.25 | 5233.44 | 7910.15 |
| YDR149C | 6.643 | 2.731774 | 1.01E-10 | 1.65E-09 | yes | up | 1.57 | 2.02 | 4.02 | 15.79 | 15.51 | 38.59 |
| YAL005C | 2.766 | 1.467738 | 1.29E-10 | 2.08E-09 | yes | up | 1392.87 | 1385.94 | 1335.99 | 3123.74 | 3315.98 | 5091.48 |
| YLR189C | 2.89 | 1.531188 | 1.3E-10 | 2.1E-09 | yes | up | 9.8 | 7.85 | 9.61 | 21.83 | 23.44 | 35.12 |
| YMR251W-A | 3.081 | 1.623427 | 1.32E-10 | 2.12E-09 | yes | up | 7580.95 | 6328.66 | 6448.26 | 15483.97 | 14081.02 | 23980.83 |
| YKR092C | 2.075 | 1.05297 | 1.75E-10 | 2.77E-09 | yes | up | 14.84 | 15.27 | 13.31 | 31.66 | 31.15 | 26.43 |
| YHR179W | 2.625 | 1.392524 | 2.02E-10 | 3.16E-09 | yes | up | 219.59 | 228.77 | 230.9 | 794.33 | 572.07 | 441.92 |
| YML090W | 3.456 | 1.78916 | 2.09E-10 | 3.26E-09 | yes | up | 41.5 | 44.71 | 62.62 | 164.88 | 134.4 | 227.85 |
| YNL164C | 2.3 | 1.201656 | 2.26E-10 | 3.51E-09 | yes | up | 10.94 | 12.08 | 11.76 | 30.66 | 23.73 | 25.33 |
| YGR136W | 2.008 | 1.0059 | 2.3E-10 | 3.57E-09 | yes | up | 451.3 | 423.28 | 431.98 | 817.26 | 777.04 | 973.99 |
| YDL113C | 2.518 | 1.332405 | 2.32E-10 | 3.59E-09 | yes | up | 12.06 | 13.38 | 12.85 | 29.12 | 27.77 | 39.94 |
| YMR135C | 2.128 | 1.089765 | 2.85E-10 | 4.35E-09 | yes | up | 323.7 | 296.3 | 323.22 | 607.3 | 599.18 | 777.02 |
| YER151C | 2.479 | 1.309481 | 2.89E-10 | 4.4E-09 | yes | up | 6.84 | 4.76 | 4.81 | 13.18 | 13 | 14.71 |
| YKR075C | 3.445 | 1.784424 | 3.86E-10 | 5.73E-09 | yes | up | 161.18 | 153.68 | 169 | 462.81 | 421.1 | 865.78 |
| YJL166W | 2.861 | 1.516631 | 4.77E-10 | 6.96E-09 | yes | up | 3934.75 | 3672.83 | 3989.91 | 9181.48 | 8436.26 | 14265.4 |
| YER050C | 2.828 | 1.499762 | 4.88E-10 | 7.1E-09 | yes | up | 174.61 | 163.8 | 167.78 | 406.33 | 382.95 | 631.39 |
| YBR082C | 2.745 | 1.457045 | 5.8E-10 | 8.31E-09 | yes | up | 885.96 | 736.71 | 791.55 | 1857.36 | 1804.83 | 2909.53 |
| YLR175W | 2.318 | 1.213125 | 6.31E-10 | 8.96E-09 | yes | up | 8.88 | 7.48 | 10.52 | 22.15 | 18.84 | 21.14 |
| YDR029W | 3.44 | 1.782227 | 6.46E-10 | 9.15E-09 | yes | up | 28.13 | 42.82 | 38.81 | 117.38 | 112.09 | 155.65 |
| YMR193W | 2.018 | 1.013189 | 7.09E-10 | 1E-08 | yes | up | 33.94 | 29.93 | 32.92 | 63.71 | 59.07 | 68.93 |
| YJR014W | 2.077 | 1.054573 | 7.8E-10 | 1.09E-08 | yes | up | 219.18 | 211.61 | 216.4 | 394.97 | 409.27 | 510.25 |
| YOL081W | 3.698 | 1.886651 | 8.02E-10 | 1.12E-08 | yes | up | 7.31 | 6.59 | 7.33 | 20.12 | 20.65 | 43.98 |
| YHR081W | 2.87 | 1.520924 | 8.59E-10 | 1.2E-08 | yes | up | 15.96 | 12.44 | 16.48 | 42.12 | 36.02 | 51.9 |
| YGR274C | 2.901 | 1.536308 | 1.09E-09 | 1.49E-08 | yes | up | 2.74 | 3.23 | 3.07 | 7.97 | 7.19 | 11.8 |
| YOR244W | 2.571 | 1.362567 | 1.61E-09 | 2.14E-08 | yes | up | 22.86 | 15.16 | 19.09 | 47.56 | 41.89 | 58.56 |
| YMR070W | 2.16 | 1.11089 | 1.75E-09 | 2.3E-08 | yes | up | 8.51 | 10.09 | 10.01 | 21.6 | 18.24 | 21.56 |
| YJR052W | 2.308 | 1.206582 | 1.93E-09 | 2.53E-08 | yes | up | 4.71 | 3.68 | 4.76 | 9.43 | 10.82 | 10.1 |
| YCL050C | 2.929 | 1.550473 | 2.45E-09 | 3.18E-08 | yes | up | 46.33 | 47.06 | 47.38 | 105.46 | 121.15 | 196.36 |
| YBR290W | 2.407 | 1.267108 | 2.63E-09 | 3.4E-08 | yes | up | 44.68 | 50.44 | 56.36 | 115.53 | 100.78 | 148.07 |
| YML097C | 2.167 | 1.115808 | 3.02E-09 | 3.87E-08 | yes | up | 6.59 | 7.01 | 7.28 | 13.46 | 15.88 | 15.64 |
| YNL075W | 2.322 | 1.215195 | 3.27E-09 | 4.15E-08 | yes | up | 15.26 | 22.12 | 17.94 | 41.32 | 41.69 | 45.34 |
| YGR057C | 2.008 | 1.005584 | 3.34E-09 | 4.22E-08 | yes | up | 23.66 | 20.1 | 22.5 | 45.16 | 41.34 | 44.16 |
| YIL152W | 2.241 | 1.163877 | 3.39E-09 | 4.28E-08 | yes | up | 221.91 | 202.77 | 222.23 | 425.4 | 419.07 | 587.23 |
| YOL078W | 2.228 | 1.155671 | 4.64E-09 | 5.76E-08 | yes | up | 2.52 | 2.36 | 2 | 4.79 | 5.06 | 5.49 |
| YDR147W | 2.314 | 1.210199 | 5.1E-09 | 6.26E-08 | yes | up | 6.95 | 6.63 | 5.59 | 15.34 | 17.22 | 12.11 |
| YDR145W | 2.042 | 1.02987 | 5.41E-09 | 6.6E-08 | yes | up | 12.62 | 12.9 | 14.71 | 26.4 | 24.65 | 30.21 |
| YMR044W | 2.267 | 1.180924 | 5.46E-09 | 6.64E-08 | yes | up | 16.13 | 14.79 | 14.41 | 31.34 | 29.88 | 41.25 |
| YDR277C | 3.293 | 1.719222 | 5.69E-09 | 6.9E-08 | yes | up | 140.91 | 130.99 | 147.72 | 359.25 | 360.17 | 739.51 |
| RDN37-2 | 4.024 | 2.008702 | 5.9E-09 | 7.11E-08 | yes | up | 3.81 | 4.99 | 2.26 | 13.07 | 12.33 | 25.07 |
| YDR527W | 2.305 | 1.204569 | 6.18E-09 | 7.41E-08 | yes | up | 31.01 | 30.47 | 35.68 | 65.51 | 66 | 91.98 |
| YPR062W | 2.436 | 1.284704 | 6.61E-09 | 7.92E-08 | yes | up | 1469.42 | 1438.16 | 1410.52 | 3061.65 | 2828.6 | 4487.16 |
| YHR102W | 2.106 | 1.07456 | 7.57E-09 | 8.95E-08 | yes | up | 26.47 | 21.22 | 24.45 | 43.82 | 48.53 | 58.57 |
| YBR022W | 2.183 | 1.126422 | 7.79E-09 | 9.18E-08 | yes | up | 87.08 | 93.76 | 89.97 | 178.78 | 170.47 | 231.09 |
| YOL136C | 3.006 | 1.587701 | 8.31E-09 | 9.71E-08 | yes | up | 44.13 | 43.43 | 46.16 | 111.23 | 104.1 | 201.43 |
| YER049W | 2.312 | 1.209276 | 8.96E-09 | 1.04E-07 | yes | up | 4.81 | 5.63 | 5.5 | 14.98 | 12.03 | 10.12 |
| YDR035W | 2.067 | 1.047654 | 1.26E-08 | 1.41E-07 | yes | up | 1266.08 | 1277.35 | 1231.88 | 2347.5 | 2264.64 | 3101.71 |
| YBL086C | 2.298 | 1.200472 | 1.65E-08 | 1.81E-07 | yes | up | 67.11 | 53.82 | 56.18 | 119.66 | 118.25 | 168.86 |
| YGR137W | 2.264 | 1.17898 | 1.78E-08 | 1.94E-07 | yes | up | 155.94 | 146.51 | 164.57 | 309.38 | 296.67 | 416.49 |
| YBR085C-A | 3.13 | 1.646242 | 1.8E-08 | 1.97E-07 | yes | up | 5058.32 | 5305.79 | 5022.19 | 12343.55 | 12666.17 | 24504.84 |
| YMR255W | 2.161 | 1.111417 | 1.83E-08 | 1.99E-07 | yes | up | 174.18 | 156.56 | 159.85 | 316.65 | 300.36 | 423.84 |
| YMR185W | 2.05 | 1.03555 | 1.93E-08 | 2.09E-07 | yes | up | 5.26 | 4.07 | 5.31 | 9.29 | 10.01 | 10.5 |
| YBL071W-A | 2.758 | 1.463662 | 2.05E-08 | 2.2E-07 | yes | up | 1525.35 | 1516.74 | 1564.22 | 3119.29 | 3406.53 | 5510.26 |
| YML081C-A | 2.141 | 1.098582 | 2.1E-08 | 2.24E-07 | yes | up | 1273.23 | 995.61 | 1048.11 | 2025.98 | 1972.07 | 1900.86 |
| YCR009C | 3.429 | 1.777892 | 2.37E-08 | 2.52E-07 | yes | up | 107.92 | 94.04 | 116.86 | 276.02 | 277.47 | 618.93 |
| YKL157W | 2.001 | 1.000943 | 2.58E-08 | 2.72E-07 | yes | up | 54.65 | 58.84 | 60.65 | 108.36 | 100.6 | 136.16 |
| YPL052W | 2.386 | 1.254306 | 3.06E-08 | 3.15E-07 | yes | up | 31.47 | 33.45 | 32.95 | 67.78 | 65.88 | 100.27 |
| YGR267C | 2.097 | 1.068473 | 3.3E-08 | 3.36E-07 | yes | up | 296.32 | 307.59 | 312.25 | 575.16 | 547.78 | 771.97 |
| YLR333C | 2.629 | 1.394691 | 3.39E-08 | 3.45E-07 | yes | up | 549.02 | 538.02 | 565.28 | 1039.05 | 896.9 | 1293.33 |
| YHR146W | 2.644 | 1.402626 | 3.48E-08 | 3.53E-07 | yes | up | 214.79 | 183.33 | 201.63 | 440.67 | 426.4 | 749.18 |
| YNL139C | 2.019 | 1.01373 | 3.52E-08 | 3.57E-07 | yes | up | 2.04 | 1.81 | 1.86 | 3.97 | 3.48 | 4.01 |
| YPR165W | 2.016 | 1.01154 | 3.56E-08 | 3.6E-07 | yes | up | 378.7 | 362.14 | 362 | 624.37 | 687.86 | 867.49 |
| YBR257W | 2.258 | 1.175242 | 3.72E-08 | 3.75E-07 | yes | up | 13.57 | 16.2 | 14.35 | 31.77 | 28.97 | 38.69 |
| YOR089C | 2.008 | 1.005605 | 4E-08 | 4.01E-07 | yes | up | 154.79 | 160.98 | 157.16 | 320.5 | 256.24 | 355.16 |
| YML125C | 2.07 | 1.049373 | 4.06E-08 | 4.06E-07 | yes | up | 105.46 | 100.34 | 125.02 | 218 | 194.6 | 264.12 |
| YLR327C | 2.185 | 1.127656 | 4.64E-08 | 4.58E-07 | yes | up | 7754.76 | 9002.14 | 7653.83 | 15092.79 | 14904.93 | 19730.75 |
| YAL019W | 2.313 | 1.209897 | 4.64E-08 | 4.58E-07 | yes | up | 2.88 | 1.95 | 2.86 | 6.24 | 6.64 | 5.08 |
| YPR125W | 2.4 | 1.26274 | 4.79E-08 | 4.72E-07 | yes | up | 9.84 | 6.5 | 6.82 | 22.06 | 17.56 | 16.65 |
| YFL001W | 2.356 | 1.236405 | 4.9E-08 | 4.82E-07 | yes | up | 4.41 | 4.43 | 6.02 | 11.38 | 13.58 | 10.35 |
| YLR244C | 2.419 | 1.274582 | 5.13E-08 | 5.04E-07 | yes | up | 296 | 274.24 | 307.93 | 619.34 | 568.88 | 949.62 |
| YDR092W | 2.2 | 1.137486 | 5.25E-08 | 5.14E-07 | yes | up | 719.44 | 682.22 | 674.48 | 1289.16 | 1294.64 | 1881.88 |
| YAR019W-A | 2.002 | 1.001565 | 6.4E-08 | 6.19E-07 | yes | up | 15.51 | 14.99 | 11.71 | 21.65 | 34.46 | 27.19 |
| YOR257W | 2.203 | 1.139671 | 6.78E-08 | 6.52E-07 | yes | up | 235.41 | 226.45 | 234.49 | 412.59 | 462.46 | 629.97 |
| YLR221C | 2.602 | 1.379722 | 6.88E-08 | 6.61E-07 | yes | up | 12.49 | 11.89 | 13.3 | 31.27 | 26.11 | 41.94 |
| YPR143W | 2.4 | 1.262931 | 6.94E-08 | 6.65E-07 | yes | up | 9.2 | 12.54 | 12.36 | 32.73 | 24.48 | 25.51 |
| YOR303W | 2.625 | 1.392503 | 9.92E-08 | 9.21E-07 | yes | up | 970.36 | 955.62 | 898.95 | 2058.11 | 1940.23 | 3579.3 |
| YPR170C | 3.462 | 1.791583 | 1.13E-07 | 1.03E-06 | yes | up | 16.58 | 8.03 | 12.46 | 44.11 | 39.81 | 51.1 |
| YJL152W | 2.24 | 1.16351 | 1.24E-07 | 1.13E-06 | yes | up | 442.24 | 451.59 | 486.6 | 899.12 | 830.89 | 1261.9 |
| YOR036W | 2.028 | 1.020021 | 1.3E-07 | 1.18E-06 | yes | up | 75.99 | 69.52 | 69.28 | 120.62 | 137.73 | 171.76 |
| YMR252C | 2.464 | 1.300833 | 1.43E-07 | 1.28E-06 | yes | up | 984.77 | 1058.32 | 1014.1 | 2064.11 | 2067.83 | 3507.17 |
| RDN37-1 | 2.855 | 1.513665 | 1.5E-07 | 1.34E-06 | yes | up | 9012.26 | 10565.03 | 7619.3 | 19100.34 | 16862.76 | 35336.83 |
| YJL218W | 3.579 | 1.839504 | 1.5E-07 | 1.34E-06 | yes | up | 7.21 | 4.19 | 3.98 | 22.16 | 26.85 | 12.69 |
| YNL244C | 2.124 | 1.086653 | 1.54E-07 | 1.37E-06 | yes | up | 1281.3 | 1160 | 1272.55 | 2248.37 | 2142.87 | 3120.19 |
| YLR336C | 3.051 | 1.609241 | 1.67E-07 | 1.49E-06 | yes | up | 1 | 1.21 | 1.77 | 3.84 | 3.46 | 5.63 |
| YOL080C | 4.727 | 2.240987 | 1.76E-07 | 1.57E-06 | yes | up | 0.55 | 0.66 | 0.6 | 3.35 | 5.07 | 3.02 |
| YDR184C | 2.926 | 1.549053 | 1.78E-07 | 1.58E-06 | yes | up | 3.51 | 4.69 | 5.23 | 12.54 | 11 | 17.68 |
| YDL012C | 2.299 | 1.200757 | 1.86E-07 | 1.64E-06 | yes | up | 581.82 | 611.72 | 486.06 | 1068.05 | 1070.99 | 1574.58 |
| YDR026C | 2.381 | 1.251394 | 1.86E-07 | 1.64E-06 | yes | up | 2.44 | 3.81 | 2.77 | 7.41 | 7.51 | 6.99 |
| YNL002C | 2.618 | 1.388617 | 2.08E-07 | 1.82E-06 | yes | up | 14.29 | 15.28 | 14.5 | 32.65 | 31.03 | 54.39 |
| YDL074C | 2.049 | 1.034963 | 2.24E-07 | 1.94E-06 | yes | up | 3.82 | 4.65 | 4.01 | 7.96 | 8.23 | 9.31 |
| YPR025C | 2.198 | 1.135962 | 2.29E-07 | 1.98E-06 | yes | up | 15.53 | 14.91 | 15.17 | 31.08 | 27.51 | 41.57 |
| YCR035C | 2.044 | 1.031275 | 2.55E-07 | 2.18E-06 | yes | up | 10.25 | 9.02 | 8.32 | 20.19 | 16.25 | 19.66 |
| YNL192W | 2.934 | 1.552678 | 2.95E-07 | 2.51E-06 | yes | up | 19.95 | 20.78 | 21.91 | 47.32 | 46.88 | 99.77 |
| YMR240C | 2.04 | 1.028667 | 3.17E-07 | 2.69E-06 | yes | up | 7.3 | 8.3 | 8.54 | 16.82 | 14.19 | 17.99 |
| YKR014C | 2.462 | 1.299754 | 3.19E-07 | 2.7E-06 | yes | up | 125.69 | 127.52 | 122.66 | 239.99 | 263.09 | 430.92 |
| YOR047C | 2.704 | 1.434872 | 3.29E-07 | 2.77E-06 | yes | up | 4.14 | 3.66 | 2.93 | 10.44 | 7.38 | 12.3 |
| YJL151C | 2.029 | 1.020474 | 3.82E-07 | 3.18E-06 | yes | up | 7098.49 | 6811.6 | 7105.69 | 12255.91 | 11850.19 | 17072.75 |
| YER079W | 2.067 | 1.047579 | 3.83E-07 | 3.19E-06 | yes | up | 165 | 139.4 | 146.53 | 247.32 | 298.21 | 372.94 |
| YJL144W | 2.181 | 1.125284 | 6.89E-07 | 5.52E-06 | yes | up | 1154.31 | 1203.12 | 1094.85 | 1943.16 | 2179.45 | 3078.43 |
| YOR191W | 2.637 | 1.399009 | 7.62E-07 | 6.05E-06 | yes | up | 1.6 | 1.56 | 1.41 | 3.32 | 3.32 | 5.85 |
| YBR142W | 2.998 | 1.583976 | 7.8E-07 | 6.18E-06 | yes | up | 0.88 | 0.72 | 0.95 | 2.88 | 1.93 | 3.36 |
| YGL166W | 2.152 | 1.105682 | 8.07E-07 | 6.34E-06 | yes | up | 576.93 | 566.14 | 552.67 | 1020.99 | 1018.94 | 1581.54 |
| YPL268W | 2.659 | 1.410661 | 8.29E-07 | 6.49E-06 | yes | up | 2.64 | 2.15 | 3.18 | 4.97 | 7.76 | 9.29 |
| YCR021C | 2.929 | 1.550644 | 9.18E-07 | 7.13E-06 | yes | up | 6532.06 | 6071.9 | 6529.72 | 26041.26 | 24525.56 | 9274.27 |
| YKL049C | 2.421 | 1.275734 | 9.57E-07 | 7.41E-06 | yes | up | 21.75 | 17.51 | 19.27 | 41.05 | 38.54 | 63.55 |
| YPL250C | 2.091 | 1.064458 | 9.98E-07 | 7.64E-06 | yes | up | 2157.7 | 2406.46 | 2766.66 | 4325.75 | 4445.45 | 6168.61 |
| YDR464W | 2.131 | 1.091711 | 1.07E-06 | 8.17E-06 | yes | up | 3.19 | 3.06 | 3.16 | 5.61 | 6.05 | 8.44 |
| YBL004W | 2.351 | 1.233037 | 1.1E-06 | 8.36E-06 | yes | up | 0.73 | 0.58 | 0.68 | 1.71 | 1.22 | 1.86 |
| YOR219C | 2.422 | 1.275915 | 1.21E-06 | 9.12E-06 | yes | up | 12.92 | 11.9 | 12.24 | 25.38 | 23.36 | 42.87 |
| YER116C | 2.029 | 1.02065 | 1.29E-06 | 9.72E-06 | yes | up | 56.29 | 50.31 | 55.15 | 99.11 | 90.82 | 134.93 |
| YDR526C | 2.926 | 1.548881 | 1.52E-06 | 1.13E-05 | yes | up | 9.91 | 9.67 | 5.72 | 28.46 | 32.04 | 18.73 |
| YER002W | 2.62 | 1.389489 | 1.85E-06 | 1.33E-05 | yes | up | 14.65 | 15.32 | 12.47 | 29.26 | 31.63 | 53.84 |
| YOR123C | 2.087 | 1.061582 | 1.93E-06 | 1.38E-05 | yes | up | 18.24 | 17.55 | 15.77 | 31.52 | 30.68 | 45.34 |
| YBR119W | 2.171 | 1.118478 | 1.94E-06 | 1.38E-05 | yes | up | 40.51 | 40.58 | 38 | 76.71 | 68.97 | 112.96 |
| YPL033C | 2.945 | 1.558342 | 2E-06 | 1.43E-05 | yes | up | 1.87 | 2.29 | 2.27 | 5.18 | 7.97 | 7.27 |
| YOL108C | 2.137 | 1.095677 | 2.24E-06 | 1.58E-05 | yes | up | 201.42 | 196.86 | 186.78 | 361.47 | 324.78 | 532.92 |
| YPR133C | 2.165 | 1.114334 | 2.44E-06 | 1.71E-05 | yes | up | 16.11 | 14.7 | 18.58 | 32.67 | 28.91 | 45.65 |
| YLR067C | 2.057 | 1.040848 | 2.59E-06 | 1.81E-05 | yes | up | 2.86 | 2.34 | 2.12 | 5.54 | 5.48 | 4.15 |
| YLR460C | 2.55 | 1.350635 | 2.88E-06 | 1.98E-05 | yes | up | 6.81 | 4.58 | 4.53 | 19.16 | 11.85 | 11.3 |
| YJR025C | 2.539 | 1.344436 | 3.03E-06 | 2.08E-05 | yes | up | 438.47 | 420.15 | 411.81 | 832.68 | 815.58 | 1636.88 |
| YNL271C | 2.069 | 1.049187 | 3.1E-06 | 2.12E-05 | yes | up | 2.37 | 2.05 | 2.2 | 3.97 | 4.02 | 5.75 |
| YPR126C | 2.572 | 1.362769 | 3.17E-06 | 2.15E-05 | yes | up | 46.67 | 25.65 | 25.57 | 93.01 | 83.81 | 71.36 |
| 21S_rRNA | 2.298 | 1.200139 | 0.002092 | 0.006974 | yes | up | 0.34 | 0.39 | 0.33 | 1.28 | 0.56 | 0.86 |
| snR191 | 2.84 | 1.50612 | 0.002531 | 0.008245 | yes | up | 22.45 | 5.49 | 16.56 | 31.44 | 32.41 | 93.61 |
| snR4 | 2.542 | 1.34568 | 4.88E-05 | 0.000253 | yes | up | 527.18 | 463.37 | 584.72 | 1015.82 | 864.41 | 1703.71 |
| snR63 | 2.959 | 1.565069 | 0.000726 | 0.002773 | yes | up | 21.98 | 18.53 | 26.46 | 36.24 | 67.28 | 129.07 |
| snR81 | 2.429 | 1.280543 | 0.019359 | 0.046987 | yes | up | 23.26 | 16.7 | 6.88 | 62.82 | 49.61 | 48.6 |
| YAR009C | 3.848 | 1.944155 | 1.41E-05 | 8.24E-05 | yes | up | 0.21 | 0.25 | 0.31 | 0.94 | 0.7 | 2.37 |
| YAR029W | 2.731 | 1.449517 | 0.003501 | 0.010973 | yes | up | 16.36 | 10.09 | 25.87 | 54.09 | 42.39 | 77.54 |
| YBL071C | 2.428 | 1.279693 | 3.22E-05 | 0.000175 | yes | up | 355.49 | 264.56 | 327.61 | 536.59 | 587.42 | 1141.24 |
| YBL093C | 2.568 | 1.360757 | 4.66E-06 | 3.06E-05 | yes | up | 417.01 | 448.82 | 393.24 | 831.86 | 812.23 | 1688.38 |
| YBR034C | 2.07 | 1.049311 | 1.92E-05 | 0.000109 | yes | up | 10.16 | 10.2 | 9.34 | 25.49 | 22.46 | 14.33 |
| YBR210W | 2.096 | 1.067894 | 0.005584 | 0.016326 | yes | up | 18.93 | 28.06 | 21.21 | 35.14 | 31.64 | 83.71 |
| YCL035C | 2.274 | 1.185447 | 4.26E-05 | 0.000225 | yes | up | 1225.55 | 1155.68 | 1110.77 | 1956.55 | 1965.13 | 3873.17 |
| YCL058C | 2.256 | 1.17404 | 1.63E-05 | 9.41E-05 | yes | up | 22.68 | 17.26 | 30.03 | 56.54 | 42.38 | 58.7 |
| YCL067C | 2.017 | 1.012333 | 5.41E-05 | 0.000276 | yes | up | 81.77 | 77.33 | 81.51 | 138.91 | 124.83 | 219.29 |
| YCR039C | 2.017 | 1.012333 | 5.41E-05 | 0.000276 | yes | up | 81.77 | 77.33 | 81.51 | 138.91 | 124.83 | 219.29 |
| YDL009C | 2.858 | 1.515197 | 9.89E-06 | 6.07E-05 | yes | up | 36.58 | 36.78 | 38.65 | 83.99 | 79.51 | 167.68 |
| YDL010W | 2.063 | 1.044729 | 6.75E-06 | 4.28E-05 | yes | up | 73.58 | 73.93 | 75.58 | 131.88 | 125.1 | 200.2 |
| YDL011C | 2.018 | 1.013255 | 0.000199 | 0.000884 | yes | up | 106.1 | 109.02 | 76.43 | 143.75 | 184.45 | 242.28 |
| YDL031W | 2.247 | 1.168173 | 1.31E-05 | 7.69E-05 | yes | up | 1.25 | 0.93 | 1.16 | 2.96 | 2.8 | 1.96 |
| YDL065C | 2.036 | 1.025646 | 0.000592 | 0.002326 | yes | up | 16.11 | 12.58 | 14.69 | 25.2 | 21.07 | 43.66 |
| YDL153C | 2.204 | 1.140428 | 0.001037 | 0.003812 | yes | up | 2.46 | 1.29 | 2.26 | 4.07 | 3.34 | 6.58 |
| YDL234C | 2.301 | 1.202082 | 2.19E-05 | 0.000123 | yes | up | 41.45 | 35.36 | 33.58 | 64.2 | 69.28 | 127.01 |
| YDR028C | 2.039 | 1.02805 | 0.000816 | 0.003084 | yes | up | 7.69 | 8.42 | 9.57 | 13.48 | 12.93 | 27.39 |
| YDR210W | 2.168 | 1.116473 | 0.014611 | 0.037326 | yes | up | 5.38 | 14.28 | 1.86 | 6.8 | 3.49 | 10.84 |
| YDR289C | 2.133 | 1.093194 | 5.66E-05 | 0.000288 | yes | up | 4.66 | 4.75 | 3.73 | 8.75 | 7.68 | 12.05 |
| YDR299W | 2.065 | 1.046166 | 0.000992 | 0.003664 | yes | up | 1.29 | 1.46 | 0.9 | 3.04 | 2.5 | 2.36 |
| YDR345C | 3.092 | 1.628579 | 3.91E-06 | 2.61E-05 | yes | up | 161.21 | 148.15 | 144.17 | 350.29 | 308.48 | 882.37 |
| YDR397C | 2.082 | 1.0581 | 3.54E-05 | 0.00019 | yes | up | 85.61 | 80.99 | 109.46 | 157.07 | 161.72 | 248.62 |
| YDR446W | 2.217 | 1.148369 | 0.034112 | 0.075308 | yes | up | 0.4 | 0.38 | 0.1 | 1.25 | 0.82 | 0.77 |
| YDR457W | 2.506 | 1.325564 | 7.26E-05 | 0.000359 | yes | up | 3.2 | 2.72 | 3.4 | 5.77 | 5.56 | 13.47 |
| YDR485C | 2.103 | 1.072497 | 2.09E-05 | 0.000118 | yes | up | 5.3 | 5.22 | 5.09 | 9.66 | 8.75 | 14.73 |
| YDR532C | 2.373 | 1.246918 | 0.000467 | 0.001878 | yes | up | 1.94 | 3.14 | 3.2 | 4.38 | 6.95 | 9.77 |
| YEL012W | 2.066 | 1.046801 | 1.3E-05 | 7.68E-05 | yes | up | 535.92 | 517.14 | 518.24 | 865.74 | 904.52 | 1456.92 |
| YER044C-A | 2.225 | 1.153804 | 0.036765 | 0.080161 | yes | up | 0.6 | 0.19 | 0.07 | 0.42 | 1.04 | 1.44 |
| YER132C | 2.233 | 1.158679 | 1.14E-05 | 6.87E-05 | yes | up | 2.04 | 1.19 | 1.44 | 3.59 | 2.9 | 4.15 |
| YER149C | 2.082 | 1.057683 | 0.000119 | 0.000561 | yes | up | 2.5 | 2.07 | 2.21 | 5.04 | 4.14 | 5.23 |
| YER158W-A | 2.191 | 1.131659 | 0.001811 | 0.006166 | yes | up | 371.25 | 273.18 | 267.04 | 414.02 | 455.8 | 998.96 |
| YER162C | 2.025 | 1.017843 | 0.000405 | 0.001656 | yes | up | 13.29 | 13.6 | 14.39 | 22.71 | 20.5 | 41.89 |
| YER165C-A | 3.456 | 1.789096 | 0.000166 | 0.000754 | yes | up | 1.96 | 3.43 | 12.93 | 29.26 | 29.08 | 25.17 |
| YER167W | 2.056 | 1.039886 | 0.000101 | 0.00048 | yes | up | 7.89 | 5.51 | 7.42 | 17.85 | 16.62 | 9.34 |
| YFL026W | 2.396 | 1.26047 | 5.7E-05 | 0.00029 | yes | up | 7.1 | 7.06 | 10.28 | 15.02 | 16.21 | 29.72 |
| YFL034C-A | 3.218 | 1.686024 | 0.000663 | 0.00256 | yes | up | 2.61 | 4.14 | 11.24 | 25.32 | 12.08 | 61.8 |
| YFL046W | 2.233 | 1.159069 | 0.000115 | 0.000544 | yes | up | 36.33 | 38.56 | 38.47 | 67.04 | 62.38 | 128.82 |
| YFR039C | 2.085 | 1.060192 | 0.000116 | 0.000548 | yes | up | 3.6 | 4.3 | 3.69 | 7.43 | 6.4 | 10.75 |
| YGL005C | 2.106 | 1.074378 | 9.27E-05 | 0.000445 | yes | up | 30.36 | 26.41 | 31.81 | 52.02 | 47.59 | 88.72 |
| YGL013C | 2.308 | 1.206698 | 6.74E-06 | 4.28E-05 | yes | up | 5.87 | 4.51 | 5.96 | 11.21 | 9.77 | 17.49 |
| YGL089C | 2.706 | 1.436039 | 0.001325 | 0.004729 | yes | up | 4.74 | 6.92 | 2.33 | 11.12 | 19.04 | 15.02 |
| YGL094C | 2.413 | 1.270684 | 8.84E-05 | 0.000428 | yes | up | 2.65 | 1.82 | 2.33 | 4.01 | 4.6 | 8.66 |
| YGL165C | 2.086 | 1.061063 | 4.53E-05 | 0.000237 | yes | up | 282.87 | 279.19 | 266.62 | 461.18 | 454.92 | 809.03 |
| YGL183C | 2.322 | 1.215625 | 0.000989 | 0.003655 | yes | up | 6.13 | 6.06 | 6.78 | 14.74 | 8.6 | 23.76 |
| YGR114C | 3.132 | 1.647208 | 0.00172 | 0.005891 | yes | up | 3.29 | 0.03 | 2.31 | 8.78 | 7.8 | 11.01 |
| YGR115C | 3.237 | 1.694809 | 0.001371 | 0.004852 | yes | up | 0 | 1.04 | 0.8 | 1.97 | 3.63 | 4.22 |
| YGR139W | 2.119 | 1.083053 | 0.040725 | 0.08703 | yes | up | 5.86 | 4.56 | 2.14 | 8.58 | 4.93 | 20.27 |
| YGR187C | 2.09 | 1.063388 | 0.000624 | 0.002433 | yes | up | 1.39 | 2.19 | 1.74 | 3.6 | 4.21 | 3.75 |
| YGR264C | 2.142 | 1.098655 | 0.00036 | 0.001494 | yes | up | 3.17 | 3.83 | 3.67 | 5.7 | 6.29 | 11.59 |
| YHR046C | 2.442 | 1.287805 | 2.94E-05 | 0.000161 | yes | up | 2.88 | 4.86 | 5.2 | 14.1 | 9.46 | 9.6 |
| YHR052W | 2.2 | 1.137184 | 0.000289 | 0.001239 | yes | up | 2.52 | 1.76 | 2.46 | 6.7 | 4.62 | 4.13 |
| YHR068W | 2.294 | 1.198045 | 1.41E-05 | 8.23E-05 | yes | up | 7.92 | 7.8 | 6.29 | 12.09 | 17.36 | 22.21 |
| YHR082C | 2.639 | 1.400258 | 9.86E-06 | 6.06E-05 | yes | up | 9.64 | 11.15 | 12.23 | 20.8 | 23.28 | 47.91 |
| YHR088W | 2.384 | 1.253585 | 5.56E-05 | 0.000283 | yes | up | 23.63 | 20.72 | 21.87 | 38.09 | 41.88 | 83.56 |
| YHR099W | 3.023 | 1.59601 | 9.2E-05 | 0.000441 | yes | up | 1.76 | 1.52 | 1.35 | 3.08 | 2.86 | 10.37 |
| YHR162W | 2.103 | 1.072146 | 2.93E-05 | 0.000161 | yes | up | 5232.16 | 5889.94 | 5173.5 | 9229.74 | 8865.52 | 15303.54 |
| YIL019W | 2.051 | 1.036147 | 0.000313 | 0.001322 | yes | up | 4.54 | 4.39 | 6.85 | 11.45 | 8.4 | 13.07 |
| YIL053W | 3.52 | 1.815671 | 8.02E-06 | 5.01E-05 | yes | up | 88.72 | 104.95 | 112.12 | 659.1 | 523.29 | 124.03 |
| YIL158W | 2.456 | 1.296116 | 0.005277 | 0.015592 | yes | up | 1.27 | 0.85 | 3.35 | 4.71 | 4.46 | 6.97 |
| YJL050W | 2.38 | 1.251168 | 1.26E-05 | 7.5E-05 | yes | up | 1.48 | 1.83 | 2.16 | 4.43 | 3.22 | 5.81 |
| YJL202C | 2.368 | 1.243524 | 0.005372 | 0.015823 | yes | up | 6.88 | 5.83 | 6.59 | 13.96 | 11.35 | 26.36 |
| YJL203W | 2.072 | 1.050794 | 4.8E-05 | 0.00025 | yes | up | 15.69 | 10.19 | 10.05 | 24.76 | 20.88 | 29.18 |
| YJR047C | 2.415 | 1.272083 | 0.000334 | 0.001397 | yes | up | 5.71 | 6.39 | 5.77 | 10.11 | 16.93 | 18.66 |
| YJR097W | 3.238 | 1.695027 | 4.9E-05 | 0.000254 | yes | up | 2.49 | 2.54 | 1.01 | 7.41 | 9.33 | 6.85 |
| YKL061W | 2.043 | 1.030904 | 0.00014 | 0.000647 | yes | up | 99.68 | 62.1 | 104.79 | 169.81 | 141.97 | 216.51 |
| YKL076C | 2.079 | 1.056151 | 0.001685 | 0.005784 | yes | up | 20.66 | 9.2 | 15.51 | 32 | 26.48 | 36.35 |
| YKL082C | 2.36 | 1.238824 | 0.000295 | 0.001262 | yes | up | 1.83 | 2 | 2.44 | 4.46 | 3.72 | 7.53 |
| YKL106W | 2.017 | 1.011925 | 1.77E-05 | 0.000101 | yes | up | 10.77 | 11.44 | 10.52 | 23.76 | 27.53 | 15.41 |
| YKL152C | 2.713 | 1.439682 | 0.000432 | 0.001752 | yes | up | 759.08 | 790.94 | 880.91 | 3612.58 | 3374.09 | 686.96 |
| YKL153W | 2.649 | 1.405291 | 0.00024 | 0.001044 | yes | up | 373.37 | 375.91 | 383.68 | 1547.86 | 1443.52 | 360.91 |
| YKL172W | 2.334 | 1.222503 | 0.000117 | 0.000554 | yes | up | 4.22 | 3.36 | 5.5 | 7.9 | 8.99 | 14.88 |
| YKL202W | 2.321 | 1.214808 | 0.023682 | 0.055608 | yes | up | 0.45 | 0.75 | 0.82 | 1.21 | 2.04 | 3.18 |
| YKR054C | 2.116 | 1.081574 | 0.005603 | 0.016344 | yes | up | 0.83 | 0.71 | 0.59 | 0.86 | 1.14 | 2.88 |
| YLR106C | 2.208 | 1.142855 | 0.002051 | 0.006864 | yes | up | 0.66 | 0.56 | 0.65 | 0.99 | 0.89 | 2.56 |
| YLR154W-E | 2.545 | 1.347465 | 0.018263 | 0.045067 | yes | up | 1.13 | 0.62 | 0 | 2.08 | 0.73 | 5.64 |
| YLR223C | 2.234 | 1.159357 | 0.001362 | 0.004829 | yes | up | 0.69 | 0.56 | 0.52 | 1.03 | 1.13 | 2.06 |
| YLR225C | 2.214 | 1.14649 | 5.74E-05 | 0.000291 | yes | up | 11.58 | 9.36 | 7.29 | 17.47 | 17.69 | 28.77 |
| YLR331C | 2.176 | 1.121869 | 0.039843 | 0.085607 | yes | up | 1.24 | 8.27 | 20.94 | 35.18 | 48.95 | 9.94 |
| YLR345W | 2.134 | 1.09377 | 1.09E-05 | 6.62E-05 | yes | up | 94.63 | 86.71 | 87.13 | 151.18 | 161.33 | 264.37 |
| YLR435W | 2.472 | 1.305846 | 0.002971 | 0.009519 | yes | up | 1.26 | 2.03 | 4.06 | 10.08 | 6.63 | 4.67 |
| YLR447C | 2.338 | 1.225422 | 0.000165 | 0.00075 | yes | up | 54.39 | 61.57 | 61.85 | 102.09 | 99.32 | 232.63 |
| YLR453C | 2.331 | 1.221098 | 0.003066 | 0.009794 | yes | up | 1.88 | 1.39 | 2.89 | 3.51 | 3.76 | 8.71 |
| YML034C-A | 2.463 | 1.300463 | 2.17E-05 | 0.000122 | yes | up | 33.42 | 19.81 | 22.35 | 52.23 | 51.47 | 83.05 |
| YML034W | 2.398 | 1.261587 | 0.000338 | 0.001413 | yes | up | 23.23 | 17.83 | 18.7 | 33.75 | 32.94 | 86.34 |
| YML054C-A | 2.134 | 1.093277 | 0.032162 | 0.071526 | yes | up | 73.05 | 205.24 | 191.25 | 387.88 | 458.61 | 218.74 |
| YML058W-A | 2.29 | 1.195374 | 0.021354 | 0.050915 | yes | up | 26.07 | 37 | 11.61 | 94.76 | 43.57 | 69.56 |
| YMR114C | 2.065 | 1.046204 | 0.000105 | 0.000497 | yes | up | 186.83 | 166.2 | 170.43 | 280.23 | 287.65 | 523.19 |
| YMR122W-A | 2.075 | 1.053015 | 0.003965 | 0.012212 | yes | up | 472.64 | 607.94 | 726.71 | 1804.08 | 1486.17 | 523.56 |
| YMR141W-A | 2.378 | 1.249737 | 0.023496 | 0.055213 | yes | up | 12.27 | 10.09 | 14.78 | 10.82 | 56.52 | 58.16 |
| YMR191W | 2.159 | 1.110144 | 0.00045 | 0.001817 | yes | up | 1136.63 | 1086.23 | 1098.03 | 1700.93 | 1761.48 | 3932.19 |
| YMR193C-A | 3.175 | 1.666929 | 1.55E-05 | 8.98E-05 | yes | up | 5.43 | 12.97 | 18.1 | 38.88 | 37.36 | 54.25 |
| YMR201C | 2.253 | 1.172001 | 4.18E-06 | 2.77E-05 | yes | up | 15.98 | 16.83 | 14.77 | 32.18 | 27.88 | 48.34 |
| YMR239C | 2.218 | 1.14894 | 0.000151 | 0.000689 | yes | up | 1.56 | 2.5 | 2.09 | 5.36 | 3.66 | 5.15 |
| YMR269W | 3.096 | 1.630264 | 1.94E-05 | 0.00011 | yes | up | 2.92 | 2.9 | 4.04 | 9.43 | 7.91 | 16.99 |
| YMR270C | 2.302 | 1.202882 | 2.75E-05 | 0.000152 | yes | up | 4 | 2.56 | 4.23 | 6.96 | 10.4 | 8.2 |
| YMR280C | 2.081 | 1.057248 | 1.17E-05 | 7.04E-05 | yes | up | 14.41 | 13.49 | 13.39 | 23.82 | 23.71 | 38.92 |
| YMR299C | 2.543 | 1.346374 | 3.37E-05 | 0.000182 | yes | up | 14.25 | 13.87 | 16.61 | 31.58 | 26.08 | 61.84 |
| YMR311C | 2.255 | 1.173376 | 3.9E-06 | 2.61E-05 | yes | up | 416.18 | 389.28 | 365.46 | 696.15 | 727.55 | 1227.08 |
| YNL065W | 2.506 | 1.32519 | 3.21E-05 | 0.000174 | yes | up | 8.6 | 9.85 | 9.84 | 34.83 | 28.32 | 12.51 |
| YNL068C | 2.023 | 1.016516 | 3.71E-06 | 2.49E-05 | yes | up | 11.72 | 9.3 | 10.86 | 19.42 | 18.21 | 26.77 |
| YNL093W | 2.024 | 1.01739 | 9.63E-05 | 0.000461 | yes | up | 319.2 | 317.77 | 308.81 | 511.55 | 500.54 | 901.01 |
| YNL113W | 2.043 | 1.030743 | 0.000245 | 0.001065 | yes | up | 29.18 | 19.92 | 19.79 | 38.59 | 42.37 | 58.46 |
| YNL129W | 2.169 | 1.117027 | 0.015954 | 0.040214 | yes | up | 2.93 | 3.55 | 3.58 | 7.84 | 2.72 | 14.82 |
| YNL133C | 2.217 | 1.148761 | 3.68E-05 | 0.000197 | yes | up | 40.85 | 47.58 | 39.23 | 79.56 | 73.03 | 132.39 |
| YNL162W-A | 2.26 | 1.176427 | 0.041905 | 0.088923 | yes | up | 4.74 | 15.5 | 0 | 8.27 | 64.77 | 29.6 |
| YNL171C | 3.347 | 1.74267 | 0.000955 | 0.003537 | yes | up | 2.26 | 1.02 | 1.11 | 4.47 | 9.37 | 9.74 |
| YNL248C | 2.086 | 1.06089 | 0.005428 | 0.015943 | yes | up | 2.55 | 1.49 | 2.92 | 5.43 | 2.92 | 7.16 |
| YNL308C | 2.813 | 1.492058 | 1.01E-05 | 6.18E-05 | yes | up | 5.11 | 5.24 | 6.03 | 12.19 | 10.89 | 26.45 |
| YNR067C | 2.019 | 1.013607 | 0.003914 | 0.012076 | yes | up | 1.52 | 1.86 | 1.59 | 2.81 | 2.16 | 5.56 |
| YOL014W | 2.033 | 1.023922 | 0.001993 | 0.006699 | yes | up | 62.05 | 68.58 | 45.78 | 82.41 | 97.58 | 180.22 |
| YOL029C | 2.778 | 1.474012 | 6.8E-05 | 0.000339 | yes | up | 2.42 | 3.14 | 3.62 | 6.17 | 11.24 | 10.72 |
| YOR075W | 2.371 | 1.245725 | 0.000257 | 0.001112 | yes | up | 8.09 | 7.06 | 7.02 | 12.24 | 13.98 | 29.28 |
| YOR077W | 2.463 | 1.300338 | 7.81E-06 | 4.89E-05 | yes | up | 6.03 | 6.09 | 8 | 14.84 | 14.2 | 21.85 |
| YOR139C | 2.391 | 1.257497 | 4.73E-05 | 0.000246 | yes | up | 27.88 | 11.66 | 20.38 | 54.11 | 44.96 | 45.68 |
| YOR210W | 2.368 | 1.243792 | 0.000256 | 0.00111 | yes | up | 99.53 | 148.62 | 158.32 | 368.16 | 229.09 | 332.99 |
| YOR242C | 2.783 | 1.47668 | 0.009434 | 0.025633 | yes | up | 0.15 | 0 | 0.39 | 1.03 | 0.39 | 1.69 |
| YOR287C | 2.001 | 1.00103 | 0.034579 | 0.076153 | yes | up | 2.31 | 1.14 | 0.41 | 2.1 | 3.64 | 3.32 |
| YOR304W | 2.042 | 1.029891 | 6.97E-06 | 4.39E-05 | yes | up | 2.08 | 1.55 | 1.78 | 4.01 | 4.16 | 2.98 |
| YOR308C | 2.144 | 1.100568 | 1.19E-05 | 7.15E-05 | yes | up | 2.36 | 1.76 | 1.97 | 4.94 | 3.99 | 4.3 |
| YOR319W | 2.105 | 1.073851 | 6.28E-06 | 4E-05 | yes | up | 30.23 | 29.81 | 21.12 | 56.42 | 47.23 | 66.75 |
| YPL043W | 2.507 | 1.326126 | 0.000151 | 0.00069 | yes | up | 1.29 | 0.55 | 0.9 | 2.47 | 2.03 | 2.91 |
| YPL081W | 2.19 | 1.130977 | 1.64E-05 | 9.43E-05 | yes | up | 21.63 | 23.86 | 39.47 | 69.3 | 55.99 | 62.32 |
| YPL095C | 2.017 | 1.012523 | 0.003291 | 0.010393 | yes | up | 116.88 | 108.48 | 101.26 | 148.88 | 151.36 | 385.25 |
| YPL232W | 2.273 | 1.184382 | 6.88E-05 | 0.000342 | yes | up | 288.35 | 262.83 | 278.34 | 475.06 | 471.69 | 984.56 |
| YPL257W-B | 2.131 | 1.091746 | 2.74E-05 | 0.000152 | yes | up | 4.53 | 4.31 | 4.35 | 7.6 | 7.67 | 13.23 |
| YPR049C | 2.34 | 1.226679 | 7.38E-05 | 0.000364 | yes | up | 5.83 | 6.56 | 7.13 | 11.96 | 11.24 | 24.33 |
| YPR087W | 2.136 | 1.09478 | 0.00032 | 0.001349 | yes | up | 57.9 | 48.12 | 53.79 | 130.67 | 69.4 | 134.63 |
| ICR1 | 0.4 | -1.32308 | 1.35E-12 | 2.83E-11 | yes | down | 9.75 | 8.09 | 10.98 | 3.5 | 3.87 | 3.58 |
| tS(AGA)H | 0.1 | -3.32306 | 5.78E-09 | 6.99E-08 | yes | down | 0.64 | 8.26 | 3.05 | 0 | 0 | 0 |
| YAL021C | 0.449 | -1.15373 | 0.000277 | 0.001191 | yes | down | 111.99 | 109.99 | 124.85 | 60.64 | 59.94 | 22.16 |
| YAL022C | 0.45 | -1.15066 | 4.72E-06 | 3.1E-05 | yes | down | 107.03 | 111.97 | 106.22 | 57.31 | 52.32 | 28.11 |
| YAL040C | 0.435 | -1.20125 | 0.001036 | 0.003809 | yes | down | 6.83 | 8.3 | 7.11 | 4.46 | 2.91 | 1.2 |
| YAL043C | 0.436 | -1.19865 | 0.000366 | 0.001516 | yes | down | 180.95 | 173.44 | 184.08 | 95.72 | 86.31 | 29.89 |
| YAL054C | 0.167 | -2.5826 | 1.98E-09 | 2.59E-08 | yes | down | 4293.89 | 4365.56 | 3623.01 | 393.64 | 888.91 | 129.53 |
| YAL058W | 0.456 | -1.13396 | 1.08E-07 | 9.96E-07 | yes | down | 33.02 | 34.21 | 39.12 | 17.21 | 17.82 | 11.01 |
| YAL062W | 0.374 | -1.4201 | 4.35E-09 | 5.43E-08 | yes | down | 2714.32 | 2723.23 | 2837.19 | 1161.97 | 1122.42 | 603.72 |
| YAR008W | 0.386 | -1.37361 | 2.5E-05 | 0.000139 | yes | down | 11.47 | 9.77 | 14.65 | 4.16 | 5.75 | 2.43 |
| YAR035W | 0.428 | -1.22456 | 0.000303 | 0.001288 | yes | down | 6022.68 | 6038.34 | 5474.5 | 2243.05 | 3498.79 | 1014.42 |
| YBL024W | 0.464 | -1.10665 | 0.000606 | 0.002372 | yes | down | 8.7 | 11.09 | 13.82 | 6.27 | 5.52 | 2.48 |
| YBL057C | 0.458 | -1.1252 | 5.84E-13 | 1.29E-11 | yes | down | 162.95 | 157.95 | 178.22 | 75.43 | 80.77 | 61.96 |
| YBL082C | 0.385 | -1.37853 | 2.94E-05 | 0.000162 | yes | down | 71.19 | 75.51 | 94.54 | 39.16 | 30.93 | 12.78 |
| YBL083C | 0.441 | -1.17992 | 0.001285 | 0.004599 | yes | down | 50.94 | 55.86 | 46.55 | 33.71 | 11.46 | 13.36 |
| YBL100W-A | 0.459 | -1.12394 | 3.92E-05 | 0.000208 | yes | down | 6.08 | 8.08 | 7.68 | 2.39 | 3.12 | 3.76 |
| YBL111C | 0.476 | -1.07098 | 0.018338 | 0.045154 | yes | down | 2.56 | 8.41 | 3.84 | 0.74 | 2.9 | 2.15 |
| YBR003W | 0.497 | -1.0076 | 6.4E-06 | 4.07E-05 | yes | down | 52.63 | 46.08 | 43.86 | 23.6 | 28.01 | 15.85 |
| YBR008C | 0.38 | -1.39532 | 2.77E-22 | 1.6E-20 | yes | down | 66.42 | 61.02 | 65.44 | 25.25 | 24.95 | 20.3 |
| YBR012W-A | 0.437 | -1.19566 | 7.3E-07 | 5.81E-06 | yes | down | 56.81 | 69.48 | 62.29 | 19.77 | 35.29 | 22.27 |
| YBR029C | 0.4 | -1.32098 | 2.29E-08 | 2.44E-07 | yes | down | 34.11 | 31.89 | 32.56 | 9.89 | 10.56 | 16.19 |
| YBR030W | 0.476 | -1.07076 | 0.001573 | 0.005466 | yes | down | 12.96 | 10.91 | 13.97 | 3.85 | 3.56 | 8.66 |
| YBR031W | 0.355 | -1.49569 | 5.21E-11 | 8.8E-10 | yes | down | 2227.8 | 2426.82 | 2636.06 | 1002.44 | 870.85 | 545.06 |
| YBR037C | 0.453 | -1.14227 | 0.00035 | 0.001459 | yes | down | 326.25 | 309.37 | 292.79 | 170.23 | 153.92 | 58.97 |
| YBR089W | 0.348 | -1.5231 | 0.003166 | 0.010073 | yes | down | 5.33 | 4.63 | 14.12 | 1.67 | 3.36 | 0.3 |
| YBR110W | 0.413 | -1.27622 | 1.39E-14 | 3.72E-13 | yes | down | 37.46 | 42.34 | 48.15 | 17.28 | 17.14 | 16.08 |
| YBR125C | 0.495 | -1.01395 | 1.03E-10 | 1.68E-09 | yes | down | 95.02 | 100.76 | 96.06 | 46.17 | 53.48 | 39.45 |
| YBR126C | 0.443 | -1.17506 | 3.48E-16 | 1.09E-14 | yes | down | 2167.1 | 2091.55 | 2020.25 | 818.74 | 870.17 | 980.71 |
| YBR157C | 0.325 | -1.62066 | 9.22E-06 | 5.72E-05 | yes | down | 170.17 | 187.14 | 193.65 | 71.85 | 61.8 | 18.45 |
| YBR219C | 0.491 | -1.02687 | 0.004897 | 0.014648 | yes | down | 41.93 | 19.5 | 37.91 | 19.52 | 10.65 | 11.76 |
| YBR220C | 0.475 | -1.07327 | 1.35E-05 | 7.92E-05 | yes | down | 56.12 | 59.89 | 56.87 | 31.95 | 29.51 | 16.16 |
| YBR222C | 0.488 | -1.0347 | 4.62E-20 | 2.06E-18 | yes | down | 408.12 | 415.59 | 446.51 | 208.34 | 198.44 | 193.45 |
| YBR232C | 0.4 | -1.32041 | 0.009577 | 0.025933 | yes | down | 6.01 | 10.6 | 7.31 | 2.4 | 1.96 | 2.2 |
| YBR246W | 0.489 | -1.03074 | 6.85E-14 | 1.67E-12 | yes | down | 64.01 | 60.26 | 68.12 | 29.06 | 31.31 | 30.24 |
| YBR294W | 0.421 | -1.24841 | 1.65E-11 | 2.96E-10 | yes | down | 23.77 | 25.19 | 27.82 | 12.54 | 9.58 | 8.75 |
| YCL020W | 0.406 | -1.29937 | 0.02449 | 0.057021 | yes | down | 0.17 | 2.2 | 2.29 | 0.66 | 0 | 0.13 |
| YCL036W | 0.249 | -2.00371 | 8.58E-14 | 2.06E-12 | yes | down | 14.1 | 14.53 | 24 | 4.63 | 3.51 | 3.61 |
| YCL057W | 0.385 | -1.37796 | 1.31E-16 | 4.32E-15 | yes | down | 74.36 | 77.53 | 79.98 | 26.84 | 25.56 | 32.64 |
| YCR010C | 0.26 | -1.94293 | 4.04E-10 | 5.96E-09 | yes | down | 3513.53 | 3624.91 | 2656.78 | 635.34 | 1159.46 | 440.16 |
| YCR017C | 0.435 | -1.19996 | 2.22E-09 | 2.89E-08 | yes | down | 55.63 | 60.33 | 64.71 | 27 | 29.68 | 18.37 |
| YCR034W | 0.344 | -1.53901 | 3.22E-09 | 4.1E-08 | yes | down | 20.9 | 20.94 | 30.69 | 6.83 | 6.49 | 9.4 |
| YCR044C | 0.462 | -1.11417 | 3.67E-10 | 5.48E-09 | yes | down | 80.76 | 71.9 | 75.65 | 31.74 | 40.82 | 28.27 |
| YCR065W | 0.361 | -1.47169 | 0.000445 | 0.001795 | yes | down | 37.4 | 32.52 | 30.65 | 10.45 | 16.52 | 2.65 |
| YCR067C | 0.258 | -1.95605 | 1.26E-23 | 8.14E-22 | yes | down | 9.57 | 10.01 | 11.21 | 2.93 | 2.51 | 2.02 |
| YCR075C | 0.217 | -2.20673 | 1.29E-34 | 2.05E-32 | yes | down | 129.76 | 118.01 | 135.16 | 26.02 | 21.89 | 29.18 |
| YCR079W | 0.404 | -1.30849 | 0.00043 | 0.001744 | yes | down | 431.11 | 420.37 | 402.89 | 188.26 | 202.51 | 50.94 |
| YCR105W | 0.473 | -1.08018 | 1.14E-05 | 6.88E-05 | yes | down | 47.17 | 52.91 | 45.5 | 26.35 | 24.66 | 13.89 |
| YDL021W | 0.485 | -1.04335 | 1.42E-10 | 2.28E-09 | yes | down | 39.22 | 37.91 | 34.06 | 17.41 | 17.6 | 16.64 |
| YDL022W | 0.214 | -2.22562 | 1.32E-09 | 1.79E-08 | yes | down | 1394.69 | 1383.1 | 1277.69 | 306.49 | 309.68 | 82.77 |
| YDL023C | 0.192 | -2.38083 | 2.92E-10 | 4.43E-09 | yes | down | 1652.84 | 1698.9 | 1560.65 | 293.4 | 338.25 | 77.57 |
| YDL042C | 0.284 | -1.81648 | 4.69E-20 | 2.08E-18 | yes | down | 22.89 | 22.71 | 24.37 | 7.01 | 7.02 | 4.65 |
| YDL103C | 0.4 | -1.32312 | 1.07E-08 | 1.22E-07 | yes | down | 29.4 | 29.75 | 29.76 | 13.27 | 12.77 | 7.39 |
| YDL142C | 0.397 | -1.33306 | 4.19E-14 | 1.05E-12 | yes | down | 159.21 | 147.87 | 145.5 | 48.35 | 56.33 | 65.24 |
| YDL144C | 0.378 | -1.40358 | 1.6E-26 | 1.4E-24 | yes | down | 389.77 | 385.84 | 399.28 | 150.65 | 152.9 | 123.5 |
| YDL145C | 0.378 | -1.4036 | 3.43E-17 | 1.18E-15 | yes | down | 10.62 | 10.41 | 11.28 | 3.88 | 4.47 | 3.35 |
| YDL164C | 0.379 | -1.40084 | 6.58E-11 | 1.1E-09 | yes | down | 8.45 | 6.91 | 9.28 | 3.37 | 2.89 | 2.53 |
| YDL171C | 0.492 | -1.02241 | 6.56E-16 | 1.97E-14 | yes | down | 561.45 | 863.18 | 754.89 | 1012.47 | 986.98 | 799.63 |
| YDL223C | 0.143 | -2.80761 | 2.78E-84 | 5.89E-81 | yes | down | 184.46 | 176.28 | 180.41 | 22.5 | 28.35 | 22.4 |
| YDL228C | 0.431 | -1.21435 | 2.92E-07 | 2.49E-06 | yes | down | 142.02 | 161.57 | 228.73 | 86.82 | 64.04 | 62.8 |
| YDL229W | 0.389 | -1.36082 | 1.28E-05 | 7.58E-05 | yes | down | 161.84 | 180.9 | 266.11 | 107.85 | 61.38 | 44.85 |
| YDL240W | 0.46 | -1.11928 | 0.001173 | 0.004253 | yes | down | 0.99 | 0.84 | 0.94 | 0.42 | 0.44 | 0.3 |
| YDL244W | 0.276 | -1.85578 | 3.01E-14 | 7.74E-13 | yes | down | 27.7 | 21.55 | 29.85 | 4.98 | 7.98 | 6.96 |
| YDR009W | 0.316 | -1.65978 | 1.21E-08 | 1.36E-07 | yes | down | 60.99 | 56.67 | 57.79 | 17.14 | 23.81 | 9.14 |
| YDR010C | 0.384 | -1.3817 | 3.96E-06 | 2.65E-05 | yes | down | 53.55 | 54.34 | 67.07 | 22.65 | 14.88 | 20.71 |
| YDR012W | 0.339 | -1.56097 | 4.38E-17 | 1.49E-15 | yes | down | 910.48 | 973.76 | 1148.01 | 393.03 | 288.53 | 289.93 |
| YDR034C-A | 0.348 | -1.52419 | 9.19E-08 | 8.63E-07 | yes | down | 32.66 | 31.47 | 58.77 | 11.73 | 78.26 | 17.68 |
| YDR034W-B | 0.411 | -1.28104 | 0.004762 | 0.01429 | yes | down | 755.3 | 1416.23 | 1281.73 | 189.14 | 245.67 | 441.96 |
| YDR064W | 0.427 | -1.22811 | 1.2E-11 | 2.17E-10 | yes | down | 1052.16 | 1125.87 | 1396.53 | 493.65 | 428.81 | 503.42 |
| YDR074W | 0.4 | -1.32041 | 1.72E-15 | 4.99E-14 | yes | down | 529.07 | 527.56 | 531.93 | 206.35 | 240.23 | 164.04 |
| YDR091C | 0.392 | -1.35243 | 7.39E-08 | 7.01E-07 | yes | down | 10.33 | 8.73 | 12.99 | 4.26 | 2.97 | 4.37 |
| YDR098C-A | 0.407 | -1.29824 | 1.87E-07 | 1.65E-06 | yes | down | 67.76 | 79.24 | 79.54 | 34.24 | 33.96 | 18.06 |
| YDR133C | 0.429 | -1.21948 | 0.000348 | 0.001452 | yes | down | 7698.94 | 7626.71 | 6892.91 | 3614.3 | 3526.02 | 1122.05 |
| YDR134C | 0.437 | -1.19312 | 0.00049 | 0.001959 | yes | down | 25129.72 | 25788.6 | 23638.1 | 12166.52 | 12628.72 | 3880.9 |
| YDR135C | 0.336 | -1.57547 | 6.64E-26 | 5.55E-24 | yes | down | 25.48 | 25.13 | 22.86 | 7.85 | 8.85 | 6.98 |
| YDR146C | 0.479 | -1.06251 | 0.005603 | 0.016344 | yes | down | 1.31 | 1.78 | 1.65 | 1.06 | 0.54 | 0.41 |
| YDR210W-A | 0.481 | -1.05572 | 4.4E-07 | 3.64E-06 | yes | down | 16.85 | 21.57 | 20.13 | 10.58 | 8.85 | 7.43 |
| YDR233C | 0.394 | -1.34372 | 3.94E-19 | 1.54E-17 | yes | down | 601.15 | 607.36 | 670.31 | 263.31 | 246.09 | 199.11 |
| YDR256C | 0.314 | -1.66958 | 2.29E-05 | 0.000128 | yes | down | 1156.81 | 1212.04 | 1013.41 | 288.11 | 491.17 | 103.19 |
| YDR261C-C | 0.373 | -1.42402 | 3.73E-05 | 0.000199 | yes | down | 37.29 | 50.8 | 51.37 | 19.11 | 20.19 | 6.55 |
| YDR284C | 0.431 | -1.21484 | 2.38E-11 | 4.17E-10 | yes | down | 107.98 | 93.11 | 108.82 | 34.99 | 44.85 | 46.59 |
| YDR316W-A | 0.429 | -1.22028 | 8.45E-18 | 3.1E-16 | yes | down | 74.78 | 74.69 | 83.15 | 32.36 | 35.02 | 28.68 |
| YDR384C | 0.149 | -2.74562 | 7.4E-10 | 1.04E-08 | yes | down | 926.9 | 927.26 | 742.69 | 64.26 | 162.86 | 20.63 |
| YDR385W | 0.37 | -1.43496 | 3.27E-19 | 1.29E-17 | yes | down | 1057.54 | 1050.61 | 1243.62 | 392.28 | 363.16 | 399.3 |
| YDR387C | 0.498 | -1.00468 | 3.28E-05 | 0.000178 | yes | down | 106.84 | 100.82 | 103.2 | 57.92 | 58.16 | 30.76 |
| YDR470C | 0.448 | -1.16002 | 1.95E-08 | 2.11E-07 | yes | down | 27.22 | 26.15 | 25.03 | 11.72 | 13.47 | 8.22 |
| YDR488C | 0.332 | -1.58941 | 2.29E-07 | 1.98E-06 | yes | down | 10.09 | 12.29 | 10.05 | 2.54 | 2.26 | 4.72 |
| YDR498C | 0.423 | -1.24083 | 4.11E-21 | 2.04E-19 | yes | down | 190.71 | 174.4 | 168.13 | 70.31 | 72.03 | 74.51 |
| YDR508C | 0.476 | -1.07139 | 0.001184 | 0.004284 | yes | down | 495.81 | 516.3 | 403.09 | 260.01 | 264.2 | 91.79 |
| YDR509W | 0.49 | -1.03028 | 0.003881 | 0.011992 | yes | down | 304.55 | 324.27 | 220.95 | 148.97 | 165.04 | 49.66 |
| YDR513W | 0.499 | -1.00234 | 9.72E-09 | 1.12E-07 | yes | down | 1509.6 | 1408.8 | 1444.17 | 761.74 | 763.82 | 523.01 |
| YDR534C | 0.308 | -1.70071 | 8.2E-09 | 9.6E-08 | yes | down | 25.04 | 23.56 | 23.42 | 7.13 | 9.23 | 3.53 |
| YDR536W | 0.287 | -1.8024 | 2.01E-20 | 9.26E-19 | yes | down | 112.73 | 107.9 | 121.81 | 35.15 | 34.65 | 22.7 |
| YEL013W | 0.47 | -1.08898 | 1.04E-09 | 1.43E-08 | yes | down | 31.18 | 32.69 | 27.97 | 12.87 | 16.37 | 12.16 |
| YEL020C | 0.354 | -1.49949 | 6.25E-11 | 1.05E-09 | yes | down | 131.92 | 118.97 | 140.53 | 49.61 | 51.39 | 28.66 |
| YEL033W | 0.405 | -1.30334 | 0.012576 | 0.032846 | yes | down | 5.18 | 10.93 | 12.75 | 0.8 | 1.58 | 5.17 |
| YEL034C-A | 0.235 | -2.09173 | 2.71E-20 | 1.23E-18 | yes | down | 1808.53 | 1853.51 | 1950.88 | 466.29 | 472.25 | 264.24 |
| YEL053W-A | 0.476 | -1.07056 | 7.38E-08 | 7.01E-07 | yes | down | 527.94 | 540.09 | 685.11 | 282.27 | 219.67 | 264.46 |
| YEL063C | 0.459 | -1.12446 | 8.19E-11 | 1.36E-09 | yes | down | 278.67 | 263.23 | 280.32 | 138.99 | 128.95 | 94.16 |
| YER015W | 0.315 | -1.66719 | 1.95E-13 | 4.46E-12 | yes | down | 108.47 | 101.51 | 92.83 | 32.66 | 36.38 | 20.05 |
| YER019W | 0.441 | -1.18214 | 5.38E-10 | 7.74E-09 | yes | down | 133.08 | 132.45 | 125.17 | 62.42 | 61.29 | 40.77 |
| YER023W | 0.28 | -1.83576 | 1.38E-46 | 4.86E-44 | yes | down | 325.03 | 306.71 | 346.77 | 92.92 | 88.91 | 80.15 |
| YER026C | 0.301 | -1.73066 | 2.83E-38 | 5.62E-36 | yes | down | 1051.39 | 1017.45 | 1076.08 | 272.15 | 318.44 | 312.74 |
| YER031C | 0.356 | -1.48867 | 3.16E-16 | 9.99E-15 | yes | down | 74.53 | 56.25 | 65.1 | 22.79 | 22.18 | 20.67 |
| YER043C | 0.414 | -1.27151 | 6.04E-08 | 5.86E-07 | yes | down | 138.49 | 117.6 | 176.75 | 70.06 | 56.66 | 41.5 |
| YER046W | 0.481 | -1.05529 | 3.45E-05 | 0.000186 | yes | down | 65.88 | 51.81 | 74.54 | 35.7 | 27.8 | 21.46 |
| YER066W | 0.286 | -1.80452 | 4.15E-06 | 2.76E-05 | yes | down | 613.06 | 598.06 | 594.27 | 159.35 | 219.75 | 54.46 |
| YER070W | 0.448 | -1.15994 | 1.65E-06 | 1.21E-05 | yes | down | 6.57 | 5.56 | 6.18 | 3.24 | 2.69 | 1.8 |
| YER082C | 0.42 | -1.25089 | 5.71E-07 | 4.64E-06 | yes | down | 54.55 | 50.74 | 49.75 | 22.36 | 26.02 | 12.68 |
| YER087W | 0.463 | -1.10991 | 0.000775 | 0.002947 | yes | down | 3 | 2.64 | 1.97 | 1.01 | 0.89 | 1.3 |
| YER089C | 0.493 | -1.02041 | 1.34E-05 | 7.89E-05 | yes | down | 114.14 | 111.53 | 131.59 | 65.21 | 65.27 | 36.47 |
| YER096W | 0.355 | -1.49504 | 1.1E-12 | 2.35E-11 | yes | down | 94.22 | 91.97 | 78.33 | 24.33 | 27.41 | 36.12 |
| YER113C | 0.475 | -1.074 | 0.001555 | 0.005412 | yes | down | 9.39 | 8.94 | 9.07 | 5.24 | 4.89 | 1.71 |
| YER121W | 0.223 | -2.16797 | 5.83E-16 | 1.78E-14 | yes | down | 167.61 | 202.44 | 144.64 | 36.61 | 40.09 | 22.34 |
| YER135C | 0.433 | -1.20698 | 0.00035 | 0.00146 | yes | down | 24.16 | 26.63 | 25.85 | 11.35 | 12.12 | 5.65 |
| YER189W | 0.309 | -1.69611 | 2.04E-05 | 0.000115 | yes | down | 19.25 | 36.34 | 27.27 | 9.36 | 5.69 | 5.03 |
| YFL037W | 0.462 | -1.11489 | 4.81E-16 | 1.49E-14 | yes | down | 59.3 | 58.87 | 64.7 | 26.25 | 26.91 | 28.06 |
| YFL064C | 0.427 | -1.22753 | 0.028999 | 0.065595 | yes | down | 2.46 | 2.42 | 4.46 | 1.69 | 0.58 | 0 |
| YFR014C | 0.421 | -1.24793 | 0.000262 | 0.001132 | yes | down | 58.24 | 57.3 | 52.09 | 25.92 | 28.53 | 8.78 |
| YGL017W | 0.465 | -1.10315 | 3.09E-10 | 4.64E-09 | yes | down | 39.47 | 35.72 | 40.52 | 15.71 | 15.96 | 19.68 |
| YGL021W | 0.49 | -1.02887 | 4.15E-06 | 2.76E-05 | yes | down | 9.12 | 9.39 | 11.9 | 4.49 | 5.87 | 3.8 |
| YGL034C | 0.412 | -1.27852 | 0.017861 | 0.044196 | yes | down | 7.52 | 8.32 | 3.98 | 1.72 | 0.56 | 2.62 |
| YGL055W | 0.471 | -1.08643 | 4.07E-08 | 4.06E-07 | yes | down | 637.34 | 629.13 | 794.93 | 469.92 | 443.62 | 451.61 |
| YGL080W | 0.48 | -1.05845 | 1.11E-08 | 1.26E-07 | yes | down | 394.21 | 388.35 | 363.76 | 188.25 | 194.92 | 130.39 |
| YGL084C | 0.302 | -1.72561 | 5.06E-29 | 5.27E-27 | yes | down | 44.65 | 42.66 | 41.95 | 11.11 | 12.92 | 13.19 |
| YGL105W | 0.344 | -1.54114 | 1.53E-16 | 4.97E-15 | yes | down | 132.95 | 116.88 | 158.86 | 39.19 | 43.95 | 49.11 |
| YGL119W | 0.282 | -1.82716 | 1.51E-20 | 7.01E-19 | yes | down | 21.59 | 19.45 | 25.85 | 6.12 | 6.43 | 5.14 |
| YGL123W | 0.362 | -1.46558 | 8.64E-10 | 1.2E-08 | yes | down | 703.37 | 736.63 | 1007.6 | 258 | 216.9 | 340.18 |
| YGL137W | 0.497 | -1.00911 | 1.71E-10 | 2.71E-09 | yes | down | 19.83 | 19.9 | 21.06 | 10.01 | 10.93 | 8.21 |
| YGL147C | 0.387 | -1.3709 | 4.87E-09 | 6.02E-08 | yes | down | 150.65 | 140.59 | 154.79 | 62.38 | 63.03 | 34.72 |
| YGL158W | 0.176 | -2.50464 | 2.64E-09 | 3.4E-08 | yes | down | 84.89 | 112.12 | 114.9 | 21.13 | 14.57 | 3.37 |
| YGL199C | 0.418 | -1.25926 | 9.26E-08 | 8.67E-07 | yes | down | 522.79 | 577.39 | 445.57 | 214.87 | 247.48 | 135.65 |
| YGL202W | 0.428 | -1.22424 | 1.27E-22 | 7.47E-21 | yes | down | 713.7 | 724.41 | 663.98 | 288.67 | 278.95 | 299.72 |
| YGL205W | 0.171 | -2.54735 | 1.78E-43 | 4.92E-41 | yes | down | 35.48 | 37.99 | 29.89 | 5.05 | 6.25 | 5.18 |
| YGL213C | 0.469 | -1.09152 | 6.91E-08 | 6.62E-07 | yes | down | 37.26 | 26.19 | 26.99 | 14.17 | 13.59 | 12.59 |
| YGR027W-A | 0.431 | -1.21263 | 2.46E-14 | 6.41E-13 | yes | down | 93.44 | 114.62 | 111.7 | 48.46 | 43.69 | 40.25 |
| YGR031W | 0.442 | -1.17759 | 3.05E-09 | 3.9E-08 | yes | down | 466.67 | 489.51 | 449.36 | 212.09 | 235.3 | 143.99 |
| YGR036C | 0.294 | -1.76418 | 5.53E-14 | 1.36E-12 | yes | down | 23.84 | 20.62 | 25.1 | 6.43 | 7.22 | 5.15 |
| YGR038C-A | 0.327 | -1.61387 | 7.05E-21 | 3.36E-19 | yes | down | 52.69 | 61 | 49.62 | 16.91 | 15.77 | 18 |
| YGR060W | 0.43 | -1.216 | 8.98E-10 | 1.24E-08 | yes | down | 32.06 | 32.22 | 39.24 | 16.66 | 12.97 | 12.57 |
| YGR065C | 0.163 | -2.62118 | 8.2E-14 | 1.98E-12 | yes | down | 252.86 | 318.7 | 275.8 | 43.4 | 51.97 | 14.72 |
| YGR067C | 0.39 | -1.36021 | 0.012668 | 0.033033 | yes | down | 264.14 | 245.93 | 238.52 | 56.69 | 110.32 | 10.86 |
| YGR069W | 0.455 | -1.13669 | 0.04749 | 0.098504 | yes | down | 4.52 | 8.7 | 5.86 | 0.73 | 3.62 | 0 |
| YGR077C | 0.357 | -1.48612 | 8.71E-21 | 4.1E-19 | yes | down | 72.16 | 61.87 | 68.55 | 22.07 | 26.72 | 20.38 |
| YGR113W | 0.485 | -1.04413 | 3.09E-05 | 0.000169 | yes | down | 20.72 | 17.77 | 15.41 | 8.69 | 6.07 | 9.63 |
| YGR121C | 0.351 | -1.50982 | 2.93E-16 | 9.31E-15 | yes | down | 433.15 | 529.24 | 452.54 | 168.12 | 179.94 | 124.44 |
| YGR131W | 0.483 | -1.04952 | 3.06E-12 | 6.03E-11 | yes | down | 211.46 | 235.43 | 197.33 | 98.3 | 103.83 | 95.63 |
| YGR154C | 0.454 | -1.14007 | 1.51E-06 | 1.12E-05 | yes | down | 33.89 | 29.4 | 36.52 | 12.54 | 11.48 | 18.19 |
| YGR161C-C | 0.447 | -1.16201 | 6E-13 | 1.32E-11 | yes | down | 64.35 | 76.71 | 81.26 | 34.34 | 31.3 | 29.65 |
| YGR190C | 0.376 | -1.4122 | 0.000228 | 0.001 | yes | down | 58.98 | 38.88 | 74.03 | 27.45 | 17.29 | 8.41 |
| YGR194C | 0.291 | -1.78175 | 6.99E-15 | 1.92E-13 | yes | down | 100.46 | 96.6 | 82.55 | 29.25 | 29.34 | 17.03 |
| YGR241C | 0.475 | -1.07519 | 3.8E-08 | 3.82E-07 | yes | down | 103.46 | 103.67 | 82.07 | 49.44 | 47.59 | 34.24 |
| YGR242W | 0.349 | -1.51737 | 1.72E-10 | 2.73E-09 | yes | down | 163.07 | 129.67 | 138.45 | 41.62 | 54.57 | 36.87 |
| YGR259C | 0.401 | -1.31986 | 0.014695 | 0.037489 | yes | down | 139.05 | 185.07 | 113.73 | 3.58 | 0 | 0 |
| YGR266W | 0.308 | -1.70075 | 6.39E-27 | 5.72E-25 | yes | down | 37.18 | 33.4 | 31.83 | 10.54 | 10.82 | 8.74 |
| YHL016C | 0.289 | -1.78841 | 1.88E-22 | 1.09E-20 | yes | down | 25.47 | 22.32 | 31.24 | 7.22 | 7.31 | 7.05 |
| YHL032C | 0.281 | -1.83092 | 4.05E-22 | 2.27E-20 | yes | down | 1702.81 | 1703.62 | 1729.98 | 516.95 | 511.5 | 335.45 |
| YHL033C | 0.431 | -1.21521 | 2.26E-08 | 2.41E-07 | yes | down | 265.77 | 301.55 | 438.32 | 157.73 | 130.64 | 141.34 |
| YHL046C | 0.481 | -1.05533 | 0.001809 | 0.006165 | yes | down | 31 | 40.7 | 30.93 | 20.48 | 12.22 | 10.95 |
| YHR007C | 0.449 | -1.15644 | 2.52E-07 | 2.16E-06 | yes | down | 43.31 | 42.84 | 62.84 | 22.33 | 17.37 | 23.16 |
| YHR020W | 0.45 | -1.15185 | 6.8E-12 | 1.27E-10 | yes | down | 292.59 | 320.12 | 361.04 | 157.31 | 147.69 | 115.99 |
| YHR033W | 0.341 | -1.55259 | 3.17E-06 | 2.15E-05 | yes | down | 1950.98 | 1961.86 | 1743.05 | 765.45 | 687.07 | 244.55 |
| YHR034C | 0.459 | -1.12348 | 3.19E-05 | 0.000174 | yes | down | 103.72 | 89.15 | 100.12 | 53.51 | 47.69 | 24.14 |
| YHR039C | 0.404 | -1.30906 | 1.02E-22 | 6.14E-21 | yes | down | 48.72 | 44.77 | 50.23 | 18.85 | 19.8 | 17.25 |
| YHR071C-A | 0.483 | -1.05034 | 0.015364 | 0.038943 | yes | down | 21.07 | 43.32 | 38.15 | 22.06 | 8.36 | 10.07 |
| YHR074W | 0.456 | -1.13271 | 7.39E-10 | 1.04E-08 | yes | down | 120.32 | 106.04 | 117.4 | 55.14 | 56.97 | 38.13 |
| YHR095W | 0.41 | -1.28631 | 0.000682 | 0.00262 | yes | down | 517.29 | 545.91 | 498.57 | 154.25 | 63.17 | 225.72 |
| YHR096C | 0.275 | -1.86304 | 1.24E-27 | 1.14E-25 | yes | down | 2548.89 | 2503.29 | 2340.62 | 550.76 | 774.86 | 600.57 |
| YHR105W | 0.476 | -1.06977 | 4.94E-07 | 4.04E-06 | yes | down | 56.97 | 56.17 | 60.81 | 29.39 | 30.05 | 18.86 |
| YHR108W | 0.386 | -1.3725 | 1.07E-08 | 1.22E-07 | yes | down | 48.96 | 42.96 | 46 | 20.57 | 18.62 | 10.72 |
| YHR117W | 0.432 | -1.20926 | 8.28E-11 | 1.37E-09 | yes | down | 26.39 | 24.87 | 26.4 | 9.45 | 9.83 | 12.62 |
| YHR136C | 0.307 | -1.70447 | 8.94E-11 | 1.47E-09 | yes | down | 48.74 | 67.8 | 47.92 | 14.96 | 16.81 | 13.47 |
| YHR139C | 0.149 | -2.74924 | 4.03E-18 | 1.5E-16 | yes | down | 107.01 | 100.93 | 92.53 | 6.67 | 11.32 | 18.33 |
| YHR203C | 0.436 | -1.19688 | 4.04E-07 | 3.35E-06 | yes | down | 445.46 | 456.96 | 657.19 | 272.17 | 192.1 | 172.07 |
| YHR214C-C | 0.457 | -1.12889 | 3.17E-12 | 6.21E-11 | yes | down | 43.4 | 54.25 | 51.68 | 22.97 | 21.28 | 21.23 |
| YIL018W | 0.379 | -1.39947 | 7.1E-09 | 8.44E-08 | yes | down | 1055.74 | 1082.87 | 1730.92 | 448.01 | 387.09 | 517.09 |
| YIL039W | 0.414 | -1.27392 | 6.65E-20 | 2.89E-18 | yes | down | 37.2 | 35.92 | 36.3 | 14.46 | 15.28 | 13.78 |
| YIL057C | 0.327 | -1.61108 | 1.36E-08 | 1.52E-07 | yes | down | 877.11 | 964.78 | 749.48 | 247.25 | 362.06 | 150.17 |
| YIL082W-A | 0.46 | -1.12071 | 3.61E-08 | 3.65E-07 | yes | down | 217.19 | 219.76 | 198.34 | 175.96 | 188.65 | 174.62 |
| YIL099W | 0.257 | -1.95785 | 3.88E-24 | 2.65E-22 | yes | down | 144.19 | 174.7 | 130.66 | 36.07 | 42.48 | 30.2 |
| YIL101C | 0.278 | -1.8494 | 1.16E-26 | 1.02E-24 | yes | down | 1415.59 | 1435.95 | 1340.37 | 385.35 | 429.87 | 289.51 |
| YIL109C | 0.465 | -1.10537 | 0.00031 | 0.001315 | yes | down | 110.21 | 105.9 | 109.85 | 57.36 | 59.86 | 22.7 |
| YIL113W | 0.421 | -1.24957 | 9.73E-07 | 7.49E-06 | yes | down | 245.67 | 209.2 | 198.95 | 96.71 | 105.84 | 52.26 |
| YIL155C | 0.321 | -1.63778 | 2.9E-17 | 1.01E-15 | yes | down | 555.88 | 534.18 | 516.92 | 166.97 | 199.66 | 122.17 |
| YIL171W | 0.471 | -1.08759 | 0.021749 | 0.05168 | yes | down | 18.39 | 18.4 | 9.3 | 3.11 | 5.36 | 7.81 |
| YIR008C | 0.424 | -1.23922 | 4.79E-12 | 9.17E-11 | yes | down | 27.33 | 22.91 | 28.31 | 10.22 | 11.76 | 9.71 |
| YIR014W | 0.471 | -1.08753 | 0.011008 | 0.029307 | yes | down | 76.62 | 73.52 | 65.82 | 36.27 | 42.9 | 6.78 |
| YIR016W | 0.465 | -1.1062 | 0.002549 | 0.008296 | yes | down | 924.51 | 846.49 | 752.7 | 446.8 | 468.93 | 125.04 |
| YIR017W-A | 0.392 | -1.35043 | 0.000545 | 0.002152 | yes | down | 31.32 | 40.58 | 64.95 | 9.8 | 26.25 | 9.27 |
| YIR020C-B | 0.338 | -1.5662 | 8.91E-05 | 0.00043 | yes | down | 4.57 | 11.73 | 7.51 | 1.78 | 2.49 | 2.21 |
| YJL051W | 0.474 | -1.0761 | 4.92E-12 | 9.36E-11 | yes | down | 27.36 | 23.24 | 27.02 | 11.84 | 13.11 | 10.51 |
| YJL079C | 0.442 | -1.17733 | 2.89E-08 | 3.01E-07 | yes | down | 510.04 | 537.87 | 529.63 | 264.46 | 243 | 153.26 |
| YJL088W | 0.499 | -1.00286 | 2.96E-17 | 1.02E-15 | yes | down | 437.77 | 475.82 | 482.62 | 221.94 | 230.6 | 219.85 |
| YJL091C | 0.472 | -1.0835 | 1.11E-07 | 1.02E-06 | yes | down | 24.41 | 28.19 | 26.21 | 10.49 | 10.54 | 14.2 |
| YJL097W | 0.345 | -1.53705 | 1.48E-16 | 4.84E-15 | yes | down | 100.04 | 107.98 | 110.16 | 40.63 | 35.25 | 27.42 |
| YJL119C | 0.497 | -1.00737 | 0.029561 | 0.066674 | yes | down | 17.44 | 17.24 | 9.49 | 4.12 | 8.28 | 5.62 |
| YJL160C | 0.302 | -1.72683 | 7.24E-20 | 3.08E-18 | yes | down | 36.74 | 42.06 | 41.92 | 11.4 | 13.03 | 9.87 |
| YJL180C | 0.497 | -1.00826 | 3.32E-05 | 0.00018 | yes | down | 20.22 | 15.51 | 15.81 | 9.71 | 8.48 | 5.96 |
| YJL216C | 0.296 | -1.7545 | 6.84E-12 | 1.28E-10 | yes | down | 6.9 | 8.12 | 7.37 | 1.79 | 1.68 | 2.56 |
| YJR001W | 0.421 | -1.24824 | 9.03E-13 | 1.96E-11 | yes | down | 102.74 | 100.04 | 101.4 | 44.01 | 46.94 | 31.75 |
| YJR024C | 0.289 | -1.78965 | 3.51E-29 | 3.71E-27 | yes | down | 205.76 | 199.01 | 207.57 | 58.33 | 63.63 | 45.85 |
| YJR028W | 0.369 | -1.43735 | 5.97E-16 | 1.81E-14 | yes | down | 73.32 | 98.03 | 91.87 | 33.13 | 31.84 | 27.56 |
| YJR038C | 0.348 | -1.52094 | 1.43E-05 | 8.35E-05 | yes | down | 32.02 | 40.45 | 30.3 | 8.78 | 14.42 | 6.98 |
| YJR040W | 0.36 | -1.47581 | 1.37E-07 | 1.24E-06 | yes | down | 7.32 | 4.89 | 4.46 | 1.77 | 2.24 | 1.47 |
| YJR044C | 0.473 | -1.07977 | 2.01E-10 | 3.15E-09 | yes | down | 391.46 | 416.86 | 398.27 | 160.26 | 172.44 | 200.59 |
| YJR094C | 0.208 | -2.26358 | 1.25E-14 | 3.37E-13 | yes | down | 74.31 | 65.02 | 65.57 | 12.36 | 17.99 | 6.82 |
| YJR095W | 0.379 | -1.39943 | 5.34E-08 | 5.21E-07 | yes | down | 365.14 | 299.83 | 282.98 | 105.4 | 153.37 | 73.82 |
| YJR116W | 0.345 | -1.53334 | 2.96E-08 | 3.07E-07 | yes | down | 768.13 | 947.42 | 849.55 | 344.87 | 342.4 | 130.73 |
| YJR123W | 0.382 | -1.3901 | 1.68E-11 | 3.01E-10 | yes | down | 924.93 | 958.84 | 1218.06 | 365.39 | 305.83 | 432.7 |
| YJR138W | 0.426 | -1.23133 | 4.49E-06 | 2.97E-05 | yes | down | 2.4 | 1.87 | 2.25 | 0.62 | 0.86 | 1.08 |
| YJR143C | 0.398 | -1.32785 | 5.74E-12 | 1.09E-10 | yes | down | 77.03 | 72.01 | 82.02 | 34.52 | 31.3 | 21.87 |
| YJR154W | 0.469 | -1.09105 | 1.68E-08 | 1.84E-07 | yes | down | 30.48 | 30.12 | 33.2 | 13.54 | 16.98 | 11.45 |
| YKL039W | 0.008 | -6.91766 | 3.26E-53 | 2.07E-50 | yes | down | 21.68 | 15.84 | 19.13 | 0 | 0 | 0 |
| YKL081W | 0.449 | -1.15498 | 6.1E-09 | 7.34E-08 | yes | down | 52.58 | 56.44 | 77.38 | 27.41 | 25.1 | 26.85 |
| YKL100C | 0.438 | -1.19263 | 1.2E-07 | 1.1E-06 | yes | down | 369.55 | 346.24 | 323.61 | 149.47 | 182.71 | 98.47 |
| YKL103C | 0.473 | -1.07922 | 9.05E-16 | 2.7E-14 | yes | down | 193.93 | 174.99 | 192.5 | 86.58 | 79.55 | 89.75 |
| YKL112W | 0.442 | -1.17728 | 4.94E-09 | 6.08E-08 | yes | down | 15.96 | 16.95 | 14.79 | 7.96 | 6.99 | 5.17 |
| YKL182W | 0.344 | -1.53964 | 2.03E-08 | 2.18E-07 | yes | down | 28.28 | 29.68 | 33.95 | 6.89 | 8.03 | 13.75 |
| YKL185W | 0.226 | -2.1462 | 5.22E-13 | 1.16E-11 | yes | down | 47.22 | 52.47 | 54.07 | 12.08 | 13.19 | 5.18 |
| YKL187C | 0.335 | -1.57943 | 5E-21 | 2.42E-19 | yes | down | 307.28 | 298.15 | 270.99 | 65.3 | 82.49 | 54.03 |
| YKL210W | 0.475 | -1.07355 | 1.13E-09 | 1.54E-08 | yes | down | 48.7 | 46.15 | 47.84 | 18.48 | 21.08 | 25.27 |
| YKR003W | 0.443 | -1.17483 | 2.6E-08 | 2.74E-07 | yes | down | 17.74 | 14.58 | 17.45 | 5.63 | 7.54 | 7.62 |
| YKR009C | 0.453 | -1.14083 | 1.67E-15 | 4.87E-14 | yes | down | 195.32 | 174.37 | 155.36 | 77.19 | 78.87 | 73.62 |
| YKR039W | 0.128 | -2.96827 | 4.1E-24 | 2.74E-22 | yes | down | 188.4 | 189.51 | 192.3 | 23.41 | 27.45 | 10.57 |
| YKR053C | 0.489 | -1.03213 | 4.96E-11 | 8.42E-10 | yes | down | 107.29 | 108.04 | 115.04 | 70.75 | 76.58 | 46.87 |
| YKR059W | 0.395 | -1.34099 | 2.19E-20 | 9.98E-19 | yes | down | 194.23 | 189.58 | 234.74 | 77.54 | 80.97 | 75.48 |
| YKR067W | 0.399 | -1.32674 | 4.96E-05 | 0.000256 | yes | down | 241.25 | 236.63 | 223.35 | 101.7 | 112.84 | 37.3 |
| YKR070W | 0.466 | -1.10092 | 6.79E-16 | 2.03E-14 | yes | down | 86.63 | 89.57 | 95.38 | 42.93 | 42.17 | 36.83 |
| YKR077W | 0.267 | -1.90601 | 1.54E-15 | 4.51E-14 | yes | down | 30.78 | 23.13 | 38.26 | 8.31 | 7.33 | 6.82 |
| YKR097W | 0.399 | -1.32583 | 4.22E-05 | 0.000223 | yes | down | 1133.55 | 964.15 | 1026.23 | 371.71 | 569.61 | 181.7 |
| YLL002W | 0.36 | -1.47526 | 9.22E-16 | 2.72E-14 | yes | down | 18.54 | 16.78 | 19.7 | 5.89 | 6.71 | 6.14 |
| YLL007C | 0.407 | -1.29853 | 6.95E-05 | 0.000345 | yes | down | 2.82 | 3.4 | 3.4 | 0.75 | 1.09 | 1.64 |
| YLL048C | 0.361 | -1.47176 | 3.88E-31 | 5.02E-29 | yes | down | 34.6 | 35.62 | 36.92 | 12.85 | 11.73 | 12.67 |
| YLL055W | 0.384 | -1.38173 | 5.86E-06 | 3.77E-05 | yes | down | 70.22 | 84.59 | 96.91 | 34.63 | 38.03 | 15.1 |
| YLR027C | 0.249 | -2.00776 | 4.16E-21 | 2.05E-19 | yes | down | 1391.07 | 1418.9 | 1446.76 | 382.94 | 375.39 | 225.68 |
| YLR044C | 0.462 | -1.11326 | 7.85E-07 | 6.2E-06 | yes | down | 2810.19 | 3059.24 | 2688.87 | 1138.45 | 977.13 | 1604.38 |
| YLR050C | 0.481 | -1.05544 | 8.98E-05 | 0.000432 | yes | down | 72.96 | 58.26 | 76.05 | 28.81 | 21.82 | 40.03 |
| YLR056W | 0.406 | -1.2989 | 4.02E-10 | 5.94E-09 | yes | down | 98.92 | 103.81 | 148.29 | 45.65 | 41.03 | 47.56 |
| YLR060W | 0.491 | -1.02719 | 3.56E-05 | 0.000191 | yes | down | 45.34 | 46.96 | 70.91 | 31.23 | 25.31 | 18.94 |
| YLR062C | 0.431 | -1.21579 | 7.7E-07 | 6.11E-06 | yes | down | 148.56 | 189.02 | 228.04 | 60.4 | 83.82 | 77.54 |
| YLR096W | 0.498 | -1.00661 | 3.46E-05 | 0.000186 | yes | down | 4.15 | 3.55 | 5.54 | 2.45 | 1.95 | 1.82 |
| YLR099C | 0.492 | -1.02372 | 0.000395 | 0.001621 | yes | down | 85.99 | 96.66 | 78.89 | 47.34 | 50.92 | 21.64 |
| YLR120C | 0.168 | -2.57724 | 2.92E-34 | 4.41E-32 | yes | down | 566.7 | 548.4 | 580.37 | 91.79 | 107.99 | 61.16 |
| YLR124W | 0.432 | -1.21011 | 0.001865 | 0.006329 | yes | down | 22.86 | 20.98 | 22.3 | 9.49 | 10.02 | 4.34 |
| YLR153C | 0.189 | -2.40716 | 7.37E-17 | 2.49E-15 | yes | down | 155.58 | 144.74 | 164.39 | 33.66 | 29.17 | 13.51 |
| YLR183C | 0.297 | -1.75211 | 5.33E-05 | 0.000273 | yes | down | 1.17 | 2.29 | 2.38 | 0.39 | 0.33 | 0.58 |
| YLR190W | 0.422 | -1.24539 | 8.26E-06 | 5.15E-05 | yes | down | 14.38 | 11.73 | 15.56 | 6.77 | 6.25 | 3.23 |
| YLR232W | 0.303 | -1.7212 | 1.01E-11 | 1.85E-10 | yes | down | 96.86 | 96.39 | 88.9 | 31.29 | 26.58 | 18.39 |
| YLR256W-A | 0.387 | -1.36791 | 6.2E-10 | 8.84E-09 | yes | down | 47.55 | 59.1 | 56.44 | 25.85 | 17.34 | 16.29 |
| YLR264W | 0.315 | -1.66443 | 7.88E-05 | 0.000385 | yes | down | 191.71 | 293.08 | 271.33 | 42.26 | 41.27 | 80.65 |
| YLR274W | 0.497 | -1.00765 | 0.000188 | 0.000842 | yes | down | 2.28 | 2.8 | 2.8 | 1.39 | 1.31 | 0.98 |
| YLR284C | 0.318 | -1.65185 | 2.73E-19 | 1.1E-17 | yes | down | 80 | 84.38 | 68.43 | 23.39 | 26.76 | 19.78 |
| YLR304C | 0.206 | -2.27773 | 5.03E-21 | 2.42E-19 | yes | down | 1306.22 | 1198.6 | 1257.46 | 283.24 | 274.77 | 147.35 |
| YLR317W | 0.449 | -1.15484 | 0.006591 | 0.018709 | yes | down | 6.54 | 8.57 | 11.04 | 2.46 | 3.8 | 3.53 |
| YLR339C | 0.349 | -1.51821 | 8.05E-17 | 2.7E-15 | yes | down | 542.97 | 591.82 | 715.36 | 233.4 | 198.3 | 174.65 |
| YLR340W | 0.375 | -1.41394 | 1.03E-16 | 3.44E-15 | yes | down | 675.11 | 740.53 | 892.61 | 308.07 | 254.63 | 260.53 |
| YLR352W | 0.477 | -1.06857 | 8.09E-08 | 7.64E-07 | yes | down | 25.53 | 25.65 | 24.15 | 10.5 | 14.76 | 9.04 |
| YLR376C | 0.41 | -1.28509 | 1.37E-05 | 8.01E-05 | yes | down | 12.1 | 9.73 | 9.34 | 4.87 | 3.26 | 3.46 |
| YLR377C | 0.267 | -1.90761 | 6.44E-12 | 1.21E-10 | yes | down | 4212.35 | 3695.46 | 3839.14 | 1088.06 | 1203.97 | 517.66 |
| YLR378C | 0.477 | -1.06743 | 3.83E-05 | 0.000204 | yes | down | 266.97 | 252.53 | 260.85 | 131.05 | 149.8 | 69.32 |
| YLR379W | 0.48 | -1.05783 | 4.67E-07 | 3.84E-06 | yes | down | 273.59 | 269.63 | 273.71 | 128.84 | 147.03 | 86.84 |
| YLR392C | 0.466 | -1.1004 | 1.27E-06 | 9.57E-06 | yes | down | 14.77 | 14.42 | 14.95 | 7.51 | 7.52 | 4.5 |
| YLR415C | 0.312 | -1.68191 | 0.002427 | 0.00795 | yes | down | 8.79 | 8.46 | 12.12 | 0.71 | 3.52 | 0 |
| YLR416C | 0.202 | -2.30709 | 5.58E-07 | 4.55E-06 | yes | down | 26.85 | 16.12 | 9.95 | 1.82 | 2.24 | 2.51 |
| YLR432W | 0.39 | -1.35959 | 6.18E-08 | 5.98E-07 | yes | down | 103.99 | 110.35 | 134.75 | 56.78 | 41.55 | 28.41 |
| YML008C | 0.481 | -1.05594 | 1.5E-09 | 2.01E-08 | yes | down | 30.09 | 32.32 | 36.99 | 14.08 | 16.77 | 14.86 |
| YML045W-A | 0.373 | -1.42274 | 1.9E-25 | 1.47E-23 | yes | down | 87.8 | 86.48 | 87.08 | 30.44 | 34.8 | 28.42 |
| YML054C | 0.427 | -1.22601 | 8.48E-11 | 1.4E-09 | yes | down | 98.81 | 106.31 | 81.71 | 39.57 | 45.46 | 32.1 |
| YML057W | 0.474 | -1.0781 | 5.51E-07 | 4.49E-06 | yes | down | 167.54 | 148.53 | 154.4 | 78.12 | 85.12 | 49.02 |
| YML075C | 0.411 | -1.2814 | 2.58E-15 | 7.41E-14 | yes | down | 12.26 | 11.67 | 12.1 | 4.28 | 5.37 | 4.56 |
| YML086C | 0.307 | -1.70562 | 4.77E-20 | 2.1E-18 | yes | down | 155.63 | 152.93 | 151.01 | 50.63 | 49.52 | 33.3 |
| YML126C | 0.144 | -2.79512 | 3.31E-25 | 2.45E-23 | yes | down | 100.64 | 83.96 | 111.38 | 10.47 | 18.03 | 8.54 |
| YMR026C | 0.462 | -1.11295 | 3.02E-07 | 2.57E-06 | yes | down | 35.69 | 44.14 | 35.83 | 21.17 | 16.2 | 13.37 |
| YMR031C | 0.461 | -1.11815 | 1.14E-05 | 6.87E-05 | yes | down | 18.88 | 17.16 | 17.74 | 9.59 | 8.97 | 4.76 |
| YMR084W | 0.441 | -1.18204 | 3.78E-08 | 3.8E-07 | yes | down | 51.46 | 49.65 | 44.26 | 17.71 | 25.92 | 16.79 |
| YMR092C | 0.399 | -1.32714 | 1.84E-29 | 2.05E-27 | yes | down | 145.47 | 141.36 | 145.04 | 53.76 | 55.3 | 57.04 |
| YMR116C | 0.282 | -1.82862 | 4.01E-24 | 2.71E-22 | yes | down | 410.56 | 447.06 | 535.97 | 143.48 | 110.23 | 115.45 |
| YMR117C | 0.45 | -1.15081 | 0.004337 | 0.013184 | yes | down | 6.92 | 7.45 | 8.31 | 1.58 | 2.25 | 4.71 |
| YMR118C | 0.194 | -2.36323 | 5.16E-37 | 9.1E-35 | yes | down | 168.19 | 153.71 | 187.37 | 35.66 | 31.03 | 24.81 |
| YMR120C | 0.222 | -2.17203 | 2.12E-25 | 1.6E-23 | yes | down | 95.43 | 111.09 | 111.88 | 24.1 | 25.81 | 15.75 |
| YMR122C | 0.452 | -1.14529 | 5.37E-06 | 3.47E-05 | yes | down | 123.01 | 95.33 | 93.68 | 41.2 | 55.91 | 30.94 |
| YMR133W | 0.483 | -1.05083 | 9.34E-11 | 1.53E-09 | yes | down | 128.96 | 128.76 | 121.22 | 61.82 | 57.68 | 40.58 |
| YMR148W | 0.471 | -1.08771 | 5.04E-14 | 1.25E-12 | yes | down | 379.31 | 389.8 | 333.37 | 161.04 | 171.19 | 157.42 |
| YMR152W | 0.319 | -1.64719 | 1.62E-23 | 1.03E-21 | yes | down | 90.45 | 83.69 | 80.77 | 22.91 | 25.59 | 28.6 |
| YMR196W | 0.358 | -1.48001 | 8.4E-34 | 1.18E-31 | yes | down | 149.8 | 149.6 | 155.87 | 51.03 | 57.83 | 48.81 |
| YMR199W | 0.484 | -1.04578 | 0.000372 | 0.001537 | yes | down | 5.37 | 4.41 | 4.13 | 1.38 | 2.39 | 2.43 |
| YMR209C | 0.38 | -1.3951 | 6.16E-11 | 1.03E-09 | yes | down | 16.16 | 22.45 | 18.24 | 6.29 | 6.88 | 7.16 |
| YMR217W | 0.364 | -1.45986 | 7.04E-08 | 6.73E-07 | yes | down | 82.18 | 99.21 | 115.09 | 45.63 | 32.33 | 21.21 |
| YMR246W | 0.322 | -1.63512 | 1.64E-06 | 1.2E-05 | yes | down | 104.59 | 105.25 | 130.34 | 39.15 | 42.91 | 13.4 |
| YMR267W | 0.41 | -1.28513 | 4.38E-10 | 6.44E-09 | yes | down | 48.89 | 49.79 | 48.29 | 22.21 | 21.05 | 13.82 |
| YMR301C | 0.315 | -1.66795 | 6.42E-25 | 4.48E-23 | yes | down | 32.7 | 30.46 | 36.23 | 11.03 | 10.19 | 8.62 |
| YMR303C | 0.095 | -3.39865 | 5.96E-16 | 1.81E-14 | yes | down | 4790.56 | 4858.24 | 4918.13 | 360.19 | 440.16 | 71.42 |
| YNL045W | 0.316 | -1.66221 | 3.38E-22 | 1.91E-20 | yes | down | 170.09 | 162.9 | 162.2 | 49.38 | 42.05 | 56.79 |
| YNL069C | 0.48 | -1.05978 | 6.62E-07 | 5.32E-06 | yes | down | 326.95 | 358 | 473.3 | 194.22 | 144.24 | 182.79 |
| YNL072W | 0.434 | -1.20415 | 1.63E-14 | 4.32E-13 | yes | down | 240.65 | 242.91 | 212.41 | 108.88 | 97.8 | 82.71 |
| YNL089C | 0.33 | -1.59954 | 0.000104 | 0.000493 | yes | down | 13.68 | 17.34 | 13.92 | 3.36 | 1.69 | 6.37 |
| YNL090W | 0.497 | -1.00789 | 0.001542 | 0.00538 | yes | down | 22.43 | 26.6 | 33.34 | 8.12 | 11.61 | 17.24 |
| YNL141W | 0.278 | -1.84603 | 1.69E-09 | 2.23E-08 | yes | down | 28.88 | 38.13 | 43.48 | 13.05 | 8.71 | 5.3 |
| YNL142W | 0.119 | -3.07207 | 7.56E-32 | 1.02E-29 | yes | down | 193.84 | 200.75 | 303.11 | 25.8 | 29.73 | 16.3 |
| YNL148C | 0.44 | -1.18347 | 6.98E-05 | 0.000347 | yes | down | 34.11 | 32.68 | 33.17 | 10.53 | 9.48 | 19.68 |
| YNL169C | 0.461 | -1.11774 | 4.26E-14 | 1.07E-12 | yes | down | 130.45 | 122.49 | 124.4 | 60.73 | 60.06 | 46.81 |
| YNL173C | 0.471 | -1.08729 | 6.44E-17 | 2.18E-15 | yes | down | 428.25 | 395.47 | 373.16 | 180.82 | 195.64 | 166.63 |
| YNL178W | 0.497 | -1.00784 | 2.17E-12 | 4.37E-11 | yes | down | 1445.46 | 1511.35 | 1751.49 | 797.5 | 708.47 | 735.83 |
| YNL209W | 0.393 | -1.34812 | 7.06E-09 | 8.43E-08 | yes | down | 523.98 | 539.15 | 678.88 | 282.04 | 204.13 | 156.07 |
| YNL219C | 0.458 | -1.12547 | 3.36E-06 | 2.27E-05 | yes | down | 69.32 | 65.89 | 64.24 | 30.34 | 37.35 | 18.6 |
| YNL239W | 0.301 | -1.73194 | 6.99E-20 | 3E-18 | yes | down | 499.96 | 505.72 | 512.05 | 155.04 | 169.88 | 106.79 |
| YNL289W | 0.374 | -1.41754 | 0.000382 | 0.001574 | yes | down | 6.56 | 6 | 3.44 | 1.05 | 1.61 | 2.27 |
| YNL307C | 0.38 | -1.39459 | 2.09E-12 | 4.26E-11 | yes | down | 187.81 | 189.21 | 198.95 | 74.65 | 82.57 | 50.37 |
| YNL321W | 0.44 | -1.18453 | 0.000484 | 0.001937 | yes | down | 226.05 | 213.08 | 204.96 | 101.45 | 118.74 | 35.78 |
| YNR002C | 0.345 | -1.53584 | 1.82E-10 | 2.87E-09 | yes | down | 2327.23 | 2427.95 | 2446.86 | 855.84 | 963.29 | 485.81 |
| YNR013C | 0.296 | -1.75472 | 1.56E-12 | 3.22E-11 | yes | down | 29.26 | 28.38 | 35.72 | 11.19 | 8.66 | 5.64 |
| YNR021W | 0.35 | -1.51345 | 1.51E-08 | 1.68E-07 | yes | down | 34.04 | 25.9 | 39.13 | 14.37 | 7.8 | 9.53 |
| YNR030W | 0.456 | -1.13266 | 7.86E-06 | 4.91E-05 | yes | down | 7.16 | 8.29 | 7.98 | 2.68 | 2.99 | 4.27 |
| YNR033W | 0.361 | -1.47153 | 4.35E-09 | 5.43E-08 | yes | down | 76.31 | 72.74 | 75.33 | 27.91 | 31.98 | 15.41 |
| YNR057C | 0.399 | -1.32563 | 4.25E-08 | 4.21E-07 | yes | down | 364.34 | 371.5 | 319.88 | 153.92 | 155.23 | 83.34 |
| YOL020W | 0.489 | -1.03154 | 0.009096 | 0.02483 | yes | down | 197.48 | 219.3 | 194.62 | 125.97 | 111.37 | 26.28 |
| YOL030W | 0.411 | -1.2824 | 1.14E-05 | 6.88E-05 | yes | down | 336.47 | 334.43 | 377.03 | 158.2 | 168.95 | 68.73 |
| YOL057W | 0.408 | -1.29437 | 1.44E-08 | 1.61E-07 | yes | down | 10.9 | 10.22 | 11.47 | 3.89 | 3.31 | 5.22 |
| YOL059W | 0.386 | -1.37519 | 2.85E-09 | 3.66E-08 | yes | down | 219.74 | 269.1 | 259.13 | 112.37 | 96.95 | 61.4 |
| YOL084W | 0.345 | -1.53684 | 2.69E-15 | 7.7E-14 | yes | down | 1304.2 | 1294.22 | 1275 | 438.32 | 515.65 | 313.39 |
| YOL085W-A | 0.477 | -1.0676 | 3.03E-19 | 1.2E-17 | yes | down | 900.18 | 931.13 | 956.78 | 581.42 | 648.85 | 393.62 |
| YOL092W | 0.459 | -1.12312 | 1.21E-17 | 4.35E-16 | yes | down | 238.41 | 247.88 | 277.38 | 116.51 | 115.2 | 105.52 |
| YOL120C | 0.362 | -1.4658 | 1.25E-18 | 4.8E-17 | yes | down | 1126.47 | 1190.52 | 1470.83 | 474.02 | 428.48 | 391.48 |
| YOL122C | 0.421 | -1.24888 | 2.66E-09 | 3.42E-08 | yes | down | 31.81 | 28.31 | 32.67 | 14.07 | 14.13 | 8.86 |
| YOL126C | 0.42 | -1.25028 | 0.001134 | 0.004129 | yes | down | 5488.95 | 4825.62 | 4433.59 | 1508.24 | 3024.05 | 722.77 |
| YOR006C | 0.448 | -1.15826 | 2.24E-19 | 9.17E-18 | yes | down | 146.8 | 142.32 | 132.89 | 60.81 | 63.9 | 57.24 |
| YOR038C | 0.459 | -1.1242 | 6.14E-06 | 3.92E-05 | yes | down | 6.18 | 7.35 | 7.76 | 3.43 | 3.71 | 2.07 |
| YOR063W | 0.334 | -1.5813 | 9.52E-23 | 5.75E-21 | yes | down | 2511.75 | 2659.65 | 2858.85 | 1000.58 | 833.85 | 724.56 |
| YOR074C | 0.345 | -1.53587 | 1.03E-05 | 6.29E-05 | yes | down | 8.86 | 5.32 | 10.19 | 1.65 | 2.86 | 2.69 |
| YOR084W | 0.483 | -1.04904 | 2.59E-05 | 0.000144 | yes | down | 690.34 | 673.43 | 628.14 | 322.36 | 395.78 | 189.52 |
| YOR087W | 0.388 | -1.36662 | 1.98E-07 | 1.74E-06 | yes | down | 10.8 | 11.37 | 8.14 | 4.17 | 4.31 | 2.44 |
| YOR094W | 0.458 | -1.12779 | 0.000382 | 0.001574 | yes | down | 29.16 | 23.89 | 31.72 | 7.28 | 10.78 | 16.42 |
| YOR095C | 0.26 | -1.94193 | 1.39E-09 | 1.87E-08 | yes | down | 104.07 | 97.68 | 119.21 | 24.23 | 36.03 | 11.85 |
| YOR100C | 0.299 | -1.74261 | 6.14E-05 | 0.000309 | yes | down | 4343.33 | 4226.43 | 3285.92 | 839.99 | 1649.09 | 227.8 |
| YOR101W | 0.33 | -1.59838 | 1.08E-13 | 2.56E-12 | yes | down | 27.54 | 23.55 | 26.19 | 8.78 | 9.08 | 6 |
| YOR133W | 0.307 | -1.70424 | 7.9E-28 | 7.38E-26 | yes | down | 517.8 | 514.44 | 627.16 | 182.91 | 157.8 | 145.72 |
| YOR142W | 0.369 | -1.4396 | 5.25E-06 | 3.41E-05 | yes | down | 514.58 | 513.59 | 502.56 | 205.37 | 222.34 | 78.54 |
| YOR157C | 0.327 | -1.61272 | 9.84E-09 | 1.13E-07 | yes | down | 292.51 | 285.44 | 308 | 109.66 | 105.27 | 47.33 |
| YOR180C | 0.422 | -1.24399 | 4.88E-07 | 4E-06 | yes | down | 24.25 | 24.79 | 20.59 | 6.91 | 11.74 | 8.67 |
| YOR192C-A | 0.471 | -1.08645 | 4.85E-06 | 3.17E-05 | yes | down | 15.43 | 12.55 | 16.4 | 6.3 | 8.39 | 5.03 |
| YOR217W | 0.376 | -1.41305 | 8.46E-05 | 0.000411 | yes | down | 3.25 | 2 | 3.73 | 0.89 | 0.62 | 1.4 |
| YOR228C | 0.487 | -1.03797 | 0.000629 | 0.002449 | yes | down | 514.4 | 477.58 | 454.14 | 258.24 | 284.55 | 107.62 |
| YOR273C | 0.42 | -1.25319 | 6.47E-13 | 1.41E-11 | yes | down | 170.24 | 168.54 | 165.89 | 73.77 | 76.83 | 52.36 |
| YOR321W | 0.47 | -1.09043 | 3.07E-07 | 2.61E-06 | yes | down | 44.47 | 40.48 | 43.2 | 21.52 | 22.52 | 13.34 |
| YOR356W | 0.379 | -1.39986 | 9.12E-13 | 1.97E-11 | yes | down | 99.68 | 99.42 | 100.72 | 41.48 | 40.34 | 26.2 |
| YOR374W | 0.122 | -3.0302 | 7.16E-23 | 4.41E-21 | yes | down | 5832.61 | 6115.57 | 5634.81 | 640.36 | 839.73 | 295.08 |
| YPL003W | 0.488 | -1.03415 | 1.47E-13 | 3.43E-12 | yes | down | 186.27 | 193.27 | 166.23 | 83.02 | 93.36 | 80.72 |
| YPL028W | 0.326 | -1.61794 | 3.2E-25 | 2.39E-23 | yes | down | 510.23 | 465.49 | 524.4 | 167.92 | 171.16 | 127.59 |
| YPL036W | 0.332 | -1.59076 | 3.15E-14 | 8.07E-13 | yes | down | 7.24 | 8.55 | 8.05 | 2.38 | 3.03 | 2.04 |
| YPL054W | 0.414 | -1.27306 | 2.36E-10 | 3.64E-09 | yes | down | 283.11 | 282.89 | 273.69 | 111.24 | 136.88 | 81.18 |
| YPL061W | 0.134 | -2.90254 | 5.05E-15 | 1.41E-13 | yes | down | 2214.33 | 2397.68 | 2354.76 | 244.14 | 372.1 | 86.23 |
| YPL062W | 0.447 | -1.16304 | 0.002043 | 0.006849 | yes | down | 13.13 | 15.52 | 12.63 | 4.38 | 5.19 | 6.05 |
| YPL104W | 0.354 | -1.49783 | 2.01E-08 | 2.16E-07 | yes | down | 9.88 | 7.9 | 10.22 | 2.3 | 4.24 | 2.55 |
| YPL116W | 0.324 | -1.6252 | 7.99E-08 | 7.56E-07 | yes | down | 20.3 | 19.93 | 15.58 | 5.88 | 7.47 | 2.91 |
| YPL131W | 0.31 | -1.68783 | 1.35E-21 | 7.11E-20 | yes | down | 1039.5 | 1104.2 | 1329.56 | 399.32 | 319.51 | 299.56 |
| YPL231W | 0.214 | -2.2223 | 1.88E-21 | 9.72E-20 | yes | down | 96.62 | 99.07 | 126.71 | 16.21 | 19.84 | 26.69 |
| YPL270W | 0.475 | -1.07285 | 2.99E-10 | 4.52E-09 | yes | down | 110.18 | 107.18 | 102.21 | 49.83 | 57.28 | 39.1 |
| YPR002W | 0.149 | -2.74664 | 1.31E-41 | 3.09E-39 | yes | down | 217.41 | 217.94 | 210.73 | 30.38 | 36.02 | 21.21 |
| YPR006C | 0.273 | -1.87143 | 7.41E-12 | 1.38E-10 | yes | down | 266.12 | 292.56 | 289.37 | 89.46 | 80.71 | 39.41 |
| YPR083W | 0.479 | -1.06191 | 4.01E-05 | 0.000213 | yes | down | 24.89 | 21.01 | 21.96 | 13.54 | 10.56 | 6.47 |
| YPR155C | 0.393 | -1.34882 | 1.33E-17 | 4.76E-16 | yes | down | 25.91 | 28.23 | 26.77 | 10.07 | 11.39 | 9.02 |
| YPR158C-C | 0.411 | -1.28428 | 1.46E-12 | 3.05E-11 | yes | down | 52.67 | 66.18 | 63 | 22.96 | 27.61 | 20.66 |
| YPR164W | 0.341 | -1.55158 | 1.01E-07 | 9.34E-07 | yes | down | 1.89 | 2.2 | 1.85 | 0.52 | 0.87 | 0.45 |
| YPR171W | 0.438 | -1.19146 | 1.46E-05 | 8.53E-05 | yes | down | 16.45 | 14.08 | 14.2 | 6.49 | 8.12 | 3.6 |
| YPR184W | 0.418 | -1.25708 | 0.000508 | 0.002019 | yes | down | 121.75 | 124.82 | 104.28 | 50.67 | 63.01 | 16.4 |
| YPR192W | 0.047 | -4.41801 | 1.14E-39 | 2.5E-37 | yes | down | 1210.12 | 1364.08 | 1487.46 | 58.37 | 58.28 | 19.19 |
| YPR193C | 0.335 | -1.57566 | 2.23E-06 | 1.58E-05 | yes | down | 39.95 | 39.4 | 49.83 | 18.94 | 11.95 | 6.59 |
| ENSRNA049651861 | 2.542 | 1.34568 | 4.88E-05 | 0.000253 | yes | up | 527.18 | 463.37 | 584.72 | 1015.82 | 864.41 | 1703.71 |
| ENSRNA049651872 | 2.14 | 1.097381 | 0.001877 | 0.006363 | yes | up | 232.6 | 214.54 | 205.16 | 298.61 | 323.38 | 823.76 |
| ENSRNA049652191 | 2.672 | 1.417768 | 0.000109 | 0.000515 | yes | up | 4226.1 | 5914.37 | 3125.26 | 7169.27 | 7388.14 | 15475.39 |
| ENSRNA049652654 | 2.417 | 1.273143 | 0.004982 | 0.014838 | yes | up | 564.84 | 128.94 | 672.98 | 1138.95 | 614.53 | 1522.81 |
| ENSRNA049652728 | 2.107 | 1.075288 | 0.014173 | 0.036391 | yes | up | 140.15 | 49.42 | 104.59 | 190.86 | 136.05 | 270.7 |

Resp genes late treatment

| Gene_id | FC(NaHS/Control) | Log2FC(NaHS/Control) | Pvalue | Padjust | Significant | Regulate | Control1_Fpkm | Control2_Fpkm | Control3_Fpkm | NaHS1_Fpkm | NaHS2_Fpkm | NaHS3_Fpkm |
| --- | --- | --- | --- | --- | --- | --- | --- | --- | --- | --- | --- | --- |
| YKR071C | 0.548 | -0.8674 | 0.005739 | 0.02796 | no | down | 213.097 | 88.655 | 154.153 | 73.251 | 72.457 | 101.011 |
| YPL106C | 1.345 | 0.4272 | 0.1919 | 0.3862 | no | up | 602.803 | 337.812 | 545.129 | 824.405 | 403.801 | 859.386 |
| YOR204W | 0.83 | -0.2692 | 0.1561 | 0.3397 | no | down | 1649.967 | 1390.873 | 1258.406 | 1354.498 | 1242.597 | 1151.355 |
| RUF5-2 | 0.787 | -0.3448 | 0.3536 | 0.5644 | no | down | 3262.534 | 12277.4 | 8349.861 | 5037.317 | 8006.314 | 5595.472 |
| YPR154W | 0.983 | -0.02403 | 0.9266 | 0.9663 | no | down | 512.118 | 1008.696 | 622.913 | 725.273 | 667.535 | 727.124 |
| YHR054C | 1.069 | 0.09564 | 0.744 | 0.8616 | no | up | 136.812 | 281.775 | 188.916 | 180.44 | 263.658 | 170.833 |
| YHR052W-A | 1 | 0 | 1 | 1 | no | no change | 0 | 0 | 0 | 0 | 0 | 0 |
| YPL239W | 1.654 | 0.7263 | 0.004374 | 0.02242 | no | up | 78.519 | 50.373 | 64.264 | 105.46 | 91.356 | 132.629 |
| YNL064C | 1.652 | 0.7238 | 0.03414 | 0.1157 | no | up | 363.652 | 118.908 | 265.889 | 397.135 | 433.339 | 493.85 |
| YPL026C | 1.009 | 0.01309 | 0.9682 | 0.985 | no | up | 35.867 | 15.096 | 34.051 | 25.18 | 36.474 | 27.016 |
| YPL151C | 1.656 | 0.7274 | 0.000101 | 0.000933 | no | up | 62.305 | 56.678 | 55.136 | 88.897 | 91.621 | 117.94 |
| RUF5-1 | 0.787 | -0.3448 | 0.3536 | 0.5644 | no | down | 3262.534 | 12277.4 | 8349.861 | 5037.317 | 8006.314 | 5595.472 |
| YHR055C | 0.907 | -0.1403 | 0.7048 | 0.8372 | no | down | 10400.98 | 33210.18 | 23950.49 | 15156.3 | 25085.09 | 18068.53 |
| YOR011W | 0.697 | -0.5198 | 0.064 | 0.184 | no | down | 8.189 | 5.381 | 5.103 | 3.271 | 4.59 | 5.385 |
| YMR161W | 1.744 | 0.8021 | 7.18E-05 | 0.000702 | no | up | 118.864 | 115.042 | 117.175 | 159.437 | 249.405 | 223.125 |
| YGL058W | 1.55 | 0.6323 | 0.002047 | 0.01215 | no | up | 81.283 | 77.142 | 94.082 | 134.098 | 119.218 | 143.226 |
| YML081W | 1.015 | 0.02137 | 0.9039 | 0.9545 | no | up | 12.074 | 11.402 | 13.757 | 12.132 | 13.562 | 13.162 |
| YGR142W | 1.281 | 0.3568 | 0.3851 | 0.5925 | no | up | 0 | 0 | 0 | 0 | 10.704 | 1.893 |
| YMR295C | 0.834 | -0.2613 | 0.1918 | 0.386 | no | down | 1459.359 | 1977.891 | 2077.8 | 1404.709 | 1742.411 | 1481.732 |
| YGR210C | 1.45 | 0.5362 | 0.009445 | 0.04281 | no | up | 223.028 | 339.339 | 227.328 | 370.675 | 405.912 | 400.717 |
| YLR259C | 1.527 | 0.6108 | 0.004536 | 0.0231 | no | up | 291.151 | 480.362 | 385.803 | 618.701 | 636.493 | 566.776 |
| YKL156W | 1.489 | 0.5747 | 0.0028 | 0.01561 | no | up | 2895.781 | 2883.846 | 2325.383 | 3254.269 | 4522.296 | 3797.686 |
| YHR053C | 0.907 | -0.1403 | 0.7048 | 0.8372 | no | down | 10400.98 | 33210.18 | 23950.49 | 15156.3 | 25085.09 | 18068.53 |
| YNL074C | 1.081 | 0.112 | 0.8502 | 0.9224 | no | up | 22.361 | 92.743 | 99.63 | 124.574 | 28.26 | 87.035 |
| YOR020C | 1.245 | 0.3164 | 0.2756 | 0.484 | no | up | 963.245 | 1915.156 | 1054.783 | 1526.862 | 1816.278 | 1486.771 |
| YOR208W | 1.441 | 0.5268 | 0.03418 | 0.1157 | no | up | 5.49 | 6.735 | 7.378 | 7.197 | 11.101 | 11.187 |
| YER150W | 1.731 | 0.7913 | 0.01017 | 0.04551 | no | up | 824.062 | 2118.557 | 1522.149 | 2638.942 | 2531.966 | 2781.932 |
| YOR338W | 0.685 | -0.5462 | 0.08199 | 0.22 | no | down | 1229.007 | 480.436 | 702.447 | 555.937 | 560.98 | 534.12 |
| YKL009W | 1.291 | 0.3683 | 0.169 | 0.3562 | no | up | 218.322 | 110.338 | 142.8 | 190.403 | 215.874 | 220.285 |
| YNL007C | 1.921 | 0.9418 | 2.01E-08 | 4.06E-07 | no | up | 238.012 | 246.769 | 230.81 | 390.836 | 496.719 | 534.252 |
| YJR046W | 1.611 | 0.6883 | 4.79E-06 | 6.12E-05 | no | up | 75.601 | 84.161 | 75.598 | 124.723 | 125.379 | 140.315 |
| YPL169C | 1.31 | 0.3893 | 0.18 | 0.3716 | no | up | 47.504 | 47.627 | 54.958 | 86.691 | 41.254 | 74.728 |
| YPR145W | 0.927 | -0.1101 | 0.6753 | 0.8207 | no | down | 1399.662 | 727.808 | 1128.275 | 978.281 | 961.601 | 1152.689 |
| YNL124W | 1.059 | 0.0832 | 0.7569 | 0.8667 | no | up | 233.826 | 131.542 | 164.478 | 208.443 | 154.216 | 213.241 |
| YMR256C | 0.557 | -0.8436 | 0.05067 | 0.1548 | no | down | 856.254 | 2042.893 | 1083.286 | 749.21 | 520.37 | 641.951 |
| YPL213W | 0.825 | -0.2768 | 0.2405 | 0.4437 | no | down | 34.718 | 26.153 | 37.117 | 26.545 | 27.938 | 27.22 |
| YBR118W | 0.849 | -0.2356 | 0.2558 | 0.4608 | no | down | 19679.76 | 31739.99 | 24130.92 | 21102.37 | 20991.98 | 22971.55 |
| YIL130W | 1.005 | 0.007293 | 0.963 | 0.9831 | no | up | 9.238 | 11.636 | 9.88 | 10.16 | 10.458 | 10.739 |
| YBL033C | 1 | 0.000614 | 0.998 | 0.9994 | no | up | 350.821 | 547.974 | 429.339 | 534.103 | 456.005 | 352.497 |
| YML130C | 1.326 | 0.407 | 0.4999 | 0.6904 | no | up | 46.026 | 0 | 15.596 | 37.883 | 58.885 | 70.636 |
| YOL067C | 1.272 | 0.3471 | 0.2114 | 0.4107 | no | up | 66.29 | 40.966 | 64.284 | 61.1 | 68.68 | 93 |
| YMR294W-A | 0.86 | -0.2177 | 0.3431 | 0.5552 | no | down | 833.464 | 1178.913 | 1208.768 | 786.877 | 1091.041 | 834.701 |
| YCR008W | 1.701 | 0.7662 | 0.000623 | 0.004552 | no | up | 19.862 | 14.603 | 21.036 | 27.863 | 34.752 | 36.259 |
| YOR007C | 1.334 | 0.4162 | 0.06596 | 0.1881 | no | up | 261.732 | 191.901 | 292.275 | 284.357 | 352.781 | 393.398 |
| YKR062W | 1.222 | 0.2887 | 0.2586 | 0.4642 | no | up | 68.178 | 37.765 | 55.739 | 67.587 | 65.377 | 70.646 |
| YNL157W | 0.879 | -0.1866 | 0.4181 | 0.6186 | no | down | 607.91 | 904.847 | 642.663 | 573.079 | 775.86 | 543.505 |
| YKL031W | 1 | 0 | 1 | 1 | no | no change | 0 | 0 | 0 | 0 | 0 | 0 |
| YBR155W | 0.965 | -0.05068 | 0.9043 | 0.9545 | no | down | 22.297 | 13.705 | 28.552 | 33.349 | 8.868 | 20.379 |
| YER147C-A | 1.281 | 0.3568 | 0.4247 | 0.6256 | no | up | 68.789 | 17.546 | 40.361 | 56.557 | 32.717 | 75.705 |
| YFR040W | 1.173 | 0.2308 | 0.4087 | 0.6097 | no | up | 6.283 | 7.893 | 6.033 | 5.72 | 11.489 | 7.39 |
| YDR186C | 1.113 | 0.1544 | 0.614 | 0.7815 | no | up | 13.543 | 8.447 | 19.325 | 16.441 | 13.95 | 17.234 |
| YJL217W | 0.888 | -0.1721 | 0.6116 | 0.7797 | no | down | 131.86 | 201.357 | 141.207 | 205.33 | 128.701 | 80.998 |
| YER148W | 1.291 | 0.3683 | 0.3425 | 0.5546 | no | up | 78.273 | 20.735 | 60.14 | 56.389 | 75.561 | 84.409 |
| YPR190C | 1.175 | 0.2328 | 0.5019 | 0.6921 | no | up | 26.073 | 14.764 | 29.472 | 36.826 | 17.007 | 31.628 |
| YBR065C | 0.933 | -0.09992 | 0.7241 | 0.8506 | no | down | 14.619 | 10.245 | 11.868 | 10.235 | 15.161 | 9.671 |
| YDR217C | 0.899 | -0.1535 | 0.7149 | 0.8437 | no | down | 0.875 | 1.157 | 0.801 | 0.486 | 1.448 | 0.662 |
| YMR272C | 1.467 | 0.5527 | 0.0823 | 0.2206 | no | up | 101.218 | 203.525 | 221.671 | 344.588 | 248.601 | 207.143 |
| YDR151C | 1.384 | 0.4687 | 0.03663 | 0.1218 | no | up | 24.623 | 23.075 | 24.428 | 34.471 | 27.919 | 40.27 |
| YER104W | 1.233 | 0.3022 | 0.2848 | 0.4955 | no | up | 13.597 | 19.479 | 21.194 | 19.675 | 22.628 | 26.08 |
| YNR075W | 1.166 | 0.2221 | 0.3696 | 0.5796 | no | up | 7.314 | 7.548 | 9.306 | 9.188 | 9.663 | 10.129 |
| YMR043W | 1.549 | 0.6316 | 0.1208 | 0.2866 | no | up | 34.071 | 27.212 | 67.805 | 84.476 | 33.285 | 96.166 |
| YPL055C | 1.406 | 0.4921 | 0.01463 | 0.06058 | no | up | 77.498 | 116.126 | 101.183 | 142.351 | 136.793 | 145.537 |
| YOR344C | 1 | 0 | 1 | 1 | no | no change | 0 | 0 | 0 | 0 | 0 | 0 |
| YBR105C | 0.662 | -0.5947 | 0.06492 | 0.1858 | no | down | 2331.453 | 2181.761 | 1994.241 | 2051.394 | 780.148 | 1388.253 |
| YKR086W | 0.895 | -0.1598 | 0.484 | 0.6758 | no | down | 6.311 | 4.802 | 5.904 | 4.365 | 5.612 | 5.69 |
| YOR213C | 1.068 | 0.09478 | 0.6892 | 0.8289 | no | up | 59.605 | 37.568 | 51.605 | 51.239 | 53.68 | 57.239 |
| YML076C | 0.888 | -0.172 | 0.6344 | 0.7943 | no | down | 3.903 | 3.398 | 5.924 | 4.636 | 2.215 | 5.039 |
| YNL281W | 1.561 | 0.6425 | 1.02E-05 | 0.000121 | no | up | 442.919 | 431.368 | 418.974 | 639.329 | 723.591 | 660.57 |
| YPR153W | 1.158 | 0.2121 | 0.4228 | 0.6239 | no | up | 76.449 | 90.305 | 68.032 | 109.077 | 76.64 | 83.106 |
| YOL047C | 0.604 | -0.7275 | 0.002931 | 0.01621 | no | down | 138.709 | 123.304 | 83.846 | 77.98 | 62.415 | 67.745 |
| YMR186W | 1.412 | 0.4978 | 0.01669 | 0.06661 | no | up | 94.652 | 69.754 | 96.09 | 118.377 | 119.881 | 143.43 |
| YML118W | 1.312 | 0.3914 | 0.08652 | 0.2281 | no | up | 6.174 | 8.077 | 7.358 | 9.319 | 10.723 | 9.131 |
| YOR027W | 1.972 | 0.9793 | 4.02E-08 | 7.62E-07 | no | up | 69.619 | 71.724 | 81.542 | 144.296 | 138.373 | 173.327 |
| YMR115W | 1.743 | 0.8018 | 0.002633 | 0.01489 | no | up | 7.77 | 5.369 | 7.744 | 10.001 | 13.884 | 14.659 |
| YBR025C | 1.41 | 0.4962 | 0.06333 | 0.1827 | no | up | 195.578 | 117.098 | 164.844 | 200.442 | 196.681 | 305.345 |
| YBR247C | 1.428 | 0.5141 | 0.0725 | 0.201 | no | up | 100.671 | 46.987 | 81.097 | 112.18 | 104.757 | 125.106 |
| YDL070W | 1 | 0 | 1 | 1 | no | no change | 0 | 0 | 0 | 0 | 0 | 3.522 |
| YER052C | 0.755 | -0.4054 | 0.1965 | 0.3921 | no | down | 171.32 | 95.119 | 168.118 | 117.283 | 71.955 | 140.925 |
| YDR433W | 0.873 | -0.1955 | 0.4271 | 0.6273 | no | down | 118.828 | 171.436 | 149.96 | 96.019 | 150.194 | 136.09 |
| YKL032C | 1 | 0 | 1 | 1 | no | no change | 0 | 0 | 0 | 0 | 0 | 0 |
| YDR524C-B | 1.33 | 0.4114 | 0.02882 | 0.1016 | no | up | 7999.793 | 9418.81 | 8603.18 | 10430.81 | 11117.36 | 11189.14 |
| YFR033C | 0.883 | -0.1795 | 0.5256 | 0.7125 | no | down | 211.054 | 321.054 | 406.295 | 311.893 | 233.175 | 270.836 |
| YNR010W | 0.852 | -0.2319 | 0.3273 | 0.5396 | no | down | 59.551 | 70.973 | 63.938 | 56.567 | 47.878 | 58.675 |
| YOR031W | 0.848 | -0.2374 | 0.5369 | 0.7228 | no | down | 380.787 | 833.295 | 865.482 | 586.791 | 556.598 | 491.793 |
| YBR114W | 0.871 | -0.199 | 0.4368 | 0.6361 | no | down | 61.092 | 34.181 | 49.321 | 44.771 | 45.304 | 38.652 |
| YEL026W | 1.332 | 0.4138 | 0.02013 | 0.0766 | no | up | 1502.658 | 1605.257 | 1483.41 | 2185.175 | 1829.262 | 2001.549 |
| YKR008W | 1.463 | 0.5488 | 0.1175 | 0.2807 | no | up | 1.55 | 1.281 | 2.393 | 2.383 | 2.858 | 2.881 |
| YPR045C | 1.693 | 0.7594 | 0.002006 | 0.01196 | no | up | 5.7 | 6.391 | 6.982 | 8.907 | 13.06 | 11.849 |
| YIL117C | 1.781 | 0.8328 | 9.91E-05 | 0.000922 | no | up | 66.235 | 72.229 | 70.06 | 93.178 | 142.945 | 150.718 |
| YOR327C | 1.61 | 0.6873 | 0.001301 | 0.008391 | no | up | 259.826 | 266.026 | 257.166 | 392.705 | 513.839 | 341.706 |
| YOR298C-A | 1.107 | 0.1467 | 0.5245 | 0.7114 | no | up | 959.415 | 1584.054 | 1075.156 | 1194.556 | 1475.346 | 1339.89 |
| YDR149C | 1 | 0 | 1 | 1 | no | no change | 0 | 0 | 0.138 | 0.122 | 0.142 | 0 |
| YAL005C | 1.701 | 0.7664 | 4.59E-06 | 5.91E-05 | no | up | 495.32 | 507.5 | 574.186 | 887.524 | 1095.801 | 1248.488 |
| YLR189C | 1.3 | 0.3787 | 0.2343 | 0.4372 | no | up | 1.678 | 1.588 | 2.037 | 2.01 | 3.407 | 1.822 |
| YMR251W-A | 0.876 | -0.1911 | 0.6299 | 0.7918 | no | down | 1291.695 | 2734.007 | 2551.693 | 1703.731 | 2452.014 | 1156.353 |
| YKR092C | 1.812 | 0.8574 | 0.005927 | 0.02876 | no | up | 140.314 | 61.381 | 84.242 | 138.594 | 195.158 | 221.425 |
| YHR179W | 1.152 | 0.2036 | 0.399 | 0.6024 | no | up | 1626.283 | 1193.8 | 1162.187 | 1640.566 | 1860.229 | 1204.666 |
| YML090W | 1.266 | 0.3404 | 0.5905 | 0.7656 | no | up | 0.465 | 3.423 | 6.339 | 4.159 | 6.691 | 3.96 |
| YNL164C | 0.955 | -0.06572 | 0.7849 | 0.8834 | no | down | 17.747 | 13.384 | 14.785 | 13.179 | 14.31 | 17.397 |
| YGR136W | 1.01 | 0.01415 | 0.9434 | 0.9734 | no | up | 266.337 | 368.472 | 326.257 | 332.979 | 285.141 | 343.447 |
| YDL113C | 1.282 | 0.3583 | 0.423 | 0.624 | no | up | 1.45 | 0.763 | 1.661 | 1.495 | 2.716 | 1.13 |
| YMR135C | 0.957 | -0.06299 | 0.7535 | 0.8647 | no | down | 34.581 | 36.89 | 47.234 | 35.836 | 41.367 | 39.038 |
| YER151C | 1 | 0 | 1 | 1 | no | no change | 0 | 0 | 0 | 0 | 0 | 0 |
| YKR075C | 0.755 | -0.4062 | 0.1221 | 0.2885 | no | down | 74.799 | 125.164 | 125.661 | 94.907 | 81.589 | 69.027 |
| YJL166W | 0.978 | -0.03163 | 0.9148 | 0.9609 | no | down | 534.872 | 997.577 | 905.001 | 725.955 | 1007.928 | 682.751 |
| YER050C | 1.664 | 0.7349 | 0.003979 | 0.02083 | no | up | 63.235 | 66.553 | 67.607 | 110.937 | 137.645 | 83.828 |
| YBR082C | 1.542 | 0.6245 | 0.002273 | 0.01319 | no | up | 840.459 | 893.789 | 913.072 | 1475.174 | 1105.634 | 1504.117 |
| YLR175W | 1.487 | 0.5726 | 0.1021 | 0.2553 | no | up | 133.675 | 45.189 | 111.36 | 152.128 | 125.919 | 181.094 |
| YDR029W | 1.078 | 0.1085 | 0.8681 | 0.9333 | no | up | 2.891 | 4.544 | 2.977 | 5.758 | 3.833 | 1.761 |
| YMR193W | 1.425 | 0.5106 | 0.09198 | 0.2369 | no | up | 6.375 | 5.947 | 6.725 | 7.562 | 10.524 | 10.23 |
| YJR014W | 1.712 | 0.7755 | 6.97E-05 | 0.000684 | no | up | 191.374 | 187.702 | 193.011 | 355.953 | 278.223 | 362.625 |
| YOL081W | 1.309 | 0.389 | 0.09245 | 0.2379 | no | up | 4.149 | 4.802 | 4.263 | 4.468 | 7.827 | 5.69 |
| YHR081W | 1 | 0 | 1 | 1 | no | no change | 1.286 | 0 | 0 | 0 | 0 | 0 |
| YGR274C | 1.13 | 0.1768 | 0.6784 | 0.8228 | no | up | 0.52 | 1.108 | 0.781 | 0.645 | 1.41 | 0.784 |
| YOR244W | 1 | 0 | 1 | 1 | no | no change | 0 | 0 | 0 | 0 | 0 | 0 |
| YMR070W | 1.311 | 0.3902 | 0.09185 | 0.2367 | no | up | 23.848 | 16.044 | 19.483 | 22.619 | 29.499 | 28.737 |
| YJR052W | 1.141 | 0.1899 | 0.5352 | 0.7216 | no | up | 6.995 | 4.457 | 7.229 | 5.346 | 9.322 | 7.574 |
| YCL050C | 1.968 | 0.9767 | 5.17E-06 | 6.53E-05 | no | up | 84.94 | 71.7 | 79.524 | 140.772 | 140.711 | 202.613 |
| YBR290W | 1.168 | 0.2246 | 0.4714 | 0.667 | no | up | 33.296 | 15.428 | 26.06 | 25.367 | 37.619 | 27.77 |
| YML097C | 1 | 0 | 1 | 1 | no | no change | 0 | 0 | 0 | 0 | 0 | 0 |
| YNL075W | 1.482 | 0.5671 | 0.3619 | 0.5726 | no | up | 35.922 | 0 | 23.459 | 41.631 | 81.409 | 80.031 |
| YGR057C | 0.878 | -0.1876 | 0.5978 | 0.7711 | no | down | 52.62 | 20.489 | 30.51 | 22.404 | 39.938 | 29.928 |
| YIL152W | 1.186 | 0.2466 | 0.244 | 0.4467 | no | up | 37.828 | 48.957 | 42.902 | 56.455 | 48.645 | 50.786 |
| YOL078W | 0.867 | -0.2051 | 0.5031 | 0.6925 | no | down | 3.475 | 2.352 | 3.649 | 2.393 | 3.767 | 2.28 |
| YDR147W | 1.053 | 0.07395 | 0.8219 | 0.9068 | no | up | 11.418 | 5.011 | 7.249 | 7.178 | 9.767 | 8.927 |
| YDR145W | 1.555 | 0.6367 | 0.02304 | 0.08462 | no | up | 3.848 | 2.746 | 3.986 | 5.907 | 5.641 | 5.741 |
| YMR044W | 1 | 0 | 1 | 1 | no | no change | 0 | 0 | 0 | 0 | 0 | 0 |
| YDR277C | 1.695 | 0.7611 | 0.01105 | 0.04883 | no | up | 21.65 | 35.216 | 45.286 | 73.466 | 49.885 | 57.708 |
| RDN37-2 | 0.677 | -0.5637 | 0.07797 | 0.2119 | no | down | 11.409 | 30.069 | 18.454 | 14.132 | 15.36 | 10.627 |
| YDR527W | 1 | 0 | 1 | 1 | no | no change | 0 | 0 | 0 | 0 | 0 | 0 |
| YPR062W | 1.069 | 0.09688 | 0.6245 | 0.7882 | no | up | 1705.851 | 1826.796 | 1428.304 | 1855.486 | 1974.516 | 1464.528 |
| YHR102W | 1.358 | 0.441 | 0.02547 | 0.09128 | no | up | 13.734 | 12.596 | 16.585 | 20.189 | 18.824 | 21.224 |
| YBR022W | 0.716 | -0.4829 | 0.01628 | 0.06564 | no | down | 93.895 | 100.5 | 83.332 | 60.904 | 76.403 | 60.802 |
| YOL136C | 1.744 | 0.8027 | 0.002414 | 0.0139 | no | up | 120.479 | 74.482 | 104.664 | 222.22 | 173.248 | 150.016 |
| YER049W | 1.474 | 0.5595 | 0.1116 | 0.2716 | no | up | 83.107 | 26.806 | 55.512 | 77.382 | 77.151 | 105.877 |
| YDR035W | 0.681 | -0.5548 | 0.006508 | 0.03121 | no | down | 964.285 | 696.717 | 748.099 | 465.853 | 604.741 | 594.8 |
| YBL086C | 1.408 | 0.4937 | 0.1291 | 0.2987 | no | up | 2.882 | 2.401 | 4.045 | 3.963 | 5.338 | 4.56 |
| YGR137W | 0.85 | -0.2348 | 0.4018 | 0.6048 | no | down | 76.185 | 121.543 | 120.577 | 88.252 | 82.649 | 92.176 |
| YBR085C-A | 0.903 | -0.1468 | 0.379 | 0.5891 | no | down | 2695.488 | 2444.61 | 2331.119 | 1865.889 | 2443.979 | 2089.643 |
| YMR255W | 1 | 0 | 1 | 1 | no | no change | 0 | 0 | 0 | 0 | 0 | 0 |
| YMR185W | 1 | 0 | 1 | 1 | no | no change | 0 | 0 | 0 | 0 | 0 | 0 |
| YBL071W-A | 1.233 | 0.3025 | 0.1804 | 0.3716 | no | up | 1176.214 | 1402.977 | 1113.173 | 1557.725 | 1460.241 | 1310.227 |
| YML081C-A | 1.431 | 0.5167 | 0.2994 | 0.5107 | no | up | 253.743 | 246.424 | 590.979 | 492.286 | 681.324 | 383.29 |
| YCR009C | 0.941 | -0.08841 | 0.7039 | 0.8372 | no | down | 56.715 | 38.442 | 54.849 | 42.032 | 49.326 | 52.455 |
| YKL157W | 0.628 | -0.6711 | 0.1972 | 0.3931 | no | down | 0.866 | 0.308 | 0.91 | 0.458 | 0.208 | 0.519 |
| YPL052W | 1.787 | 0.8379 | 0.002111 | 0.01246 | no | up | 34.536 | 30.734 | 23.924 | 43.126 | 76.829 | 47.732 |
| YGR267C | 0.79 | -0.3409 | 0.1188 | 0.2827 | no | down | 156.382 | 107.999 | 145.084 | 111.554 | 115.887 | 98.64 |
| YLR333C | 1.319 | 0.3989 | 0.03554 | 0.1192 | no | up | 2356.067 | 2759.976 | 2191.376 | 2586.694 | 3446.957 | 3398.934 |
| YHR146W | 1.543 | 0.6255 | 0.02391 | 0.08708 | no | up | 15.512 | 13.766 | 22.213 | 28.405 | 33.427 | 21.234 |
| YNL139C | 0.844 | -0.2444 | 0.4631 | 0.6607 | no | down | 0.757 | 0.874 | 1.266 | 0.869 | 0.719 | 0.886 |
| YPR165W | 0.954 | -0.06801 | 0.7209 | 0.8486 | no | down | 775.929 | 684.318 | 694.387 | 541.721 | 806.656 | 736.937 |
| YBR257W | 1.473 | 0.5591 | 0.1035 | 0.2578 | no | up | 9.94 | 7.154 | 9.573 | 9.291 | 20.423 | 12.022 |
| YOR089C | 1.135 | 0.1831 | 0.3088 | 0.5192 | no | up | 76.832 | 69.914 | 73.076 | 75.447 | 94.233 | 83.869 |
| YML125C | 1.306 | 0.3854 | 0.01879 | 0.07292 | no | up | 283.5 | 295.75 | 257.532 | 393.743 | 353.756 | 366.992 |
| YLR327C | 1.975 | 0.9821 | 0.001786 | 0.01091 | no | up | 432.769 | 860.458 | 580.98 | 1125.904 | 1590.646 | 1012.181 |
| YAL019W | 1.702 | 0.7669 | 0.02543 | 0.09121 | no | up | 2.189 | 1.416 | 3.55 | 4.571 | 3.786 | 4.713 |
| YPR125W | 1.664 | 0.7346 | 0.000156 | 0.001337 | no | up | 30.842 | 23.838 | 28.245 | 43.575 | 47.983 | 51.641 |
| YFL001W | 1.935 | 0.9523 | 0.03435 | 0.116 | no | up | 15.786 | 2.906 | 16.348 | 20.834 | 26.149 | 30.936 |
| YLR244C | 1.244 | 0.3148 | 0.08497 | 0.2256 | no | up | 658.98 | 689.834 | 646.164 | 955.129 | 748.755 | 828.715 |
| YDR092W | 1.511 | 0.5957 | 0.001986 | 0.01188 | no | up | 230.296 | 308.962 | 265.068 | 400.323 | 444.629 | 377.426 |
| YAR019W-A | 1 | 0 | 1 | 1 | no | no change | 1.55 | 0 | 0.801 | 2.019 | 0 | 0.743 |
| YOR257W | 0.965 | -0.05089 | 0.7843 | 0.8834 | no | down | 119.019 | 136.898 | 116.226 | 110.965 | 133.187 | 114.815 |
| YLR221C | 1.511 | 0.5959 | 0.07436 | 0.2049 | no | up | 25.489 | 11.488 | 25.209 | 26.498 | 37.298 | 35.282 |
| YPR143W | 1.648 | 0.7205 | 0.05414 | 0.1622 | no | up | 15.184 | 8.201 | 11.136 | 16.413 | 31.411 | 13.457 |
| YOR303W | 1.41 | 0.4961 | 0.02207 | 0.08189 | no | up | 1245.897 | 871.626 | 924.425 | 1509.44 | 1299.949 | 1617.944 |
| YPR170C | 0.741 | -0.433 | 0.358 | 0.5687 | no | down | 19.525 | 31.436 | 19.473 | 17.609 | 9.814 | 20.115 |
| YJL152W | 0.805 | -0.3131 | 0.3379 | 0.5503 | no | down | 143.825 | 339.647 | 222.413 | 195.703 | 201.233 | 152.204 |
| YOR036W | 0.585 | -0.7727 | 0.003302 | 0.01793 | no | down | 44.558 | 31.017 | 45.296 | 17.731 | 24.957 | 28.207 |
| YMR252C | 1 | 0 | 1 | 1 | no | no change | 0 | 0 | 0 | 0 | 0 | 0 |
| RDN37-1 | 0.677 | -0.5637 | 0.07797 | 0.2119 | no | down | 11.409 | 30.069 | 18.454 | 14.132 | 15.36 | 10.627 |
| YJL218W | 0.696 | -0.5221 | 0.4026 | 0.6051 | no | down | 2.125 | 2.955 | 1.414 | 1.851 | 0.18 | 1.639 |
| YNL244C | 1.234 | 0.3032 | 0.2083 | 0.4071 | no | up | 2720.987 | 3879.379 | 2585.398 | 3054.061 | 4658.143 | 3445.637 |
| YLR336C | 1.018 | 0.02577 | 0.9341 | 0.9686 | no | up | 8.007 | 6.661 | 5.647 | 4.748 | 10.221 | 5.639 |
| YOL080C | 1.444 | 0.5301 | 0.1123 | 0.2724 | no | up | 23.811 | 9.506 | 15.151 | 24.152 | 26.196 | 23.108 |
| YDR184C | 1.33 | 0.4117 | 0.1609 | 0.3474 | no | up | 19.534 | 11.193 | 16.546 | 23.339 | 17.736 | 24.054 |
| YDL012C | 1.185 | 0.2451 | 0.398 | 0.6019 | no | up | 348.139 | 605.624 | 457.456 | 589.782 | 417.732 | 623.974 |
| YDR026C | 1.306 | 0.3849 | 0.1985 | 0.3948 | no | up | 6.849 | 3.669 | 5.973 | 6.973 | 8.574 | 7.034 |
| YNL002C | 1.489 | 0.5748 | 0.0243 | 0.08825 | no | up | 49.829 | 31.965 | 37.206 | 54.23 | 77.122 | 53.269 |
| YDL074C | 1.358 | 0.4417 | 0.1976 | 0.3934 | no | up | 1.769 | 2.487 | 2.215 | 2.355 | 4.41 | 2.463 |
| YPR025C | 1.181 | 0.2401 | 0.3412 | 0.5532 | no | up | 6.347 | 5.836 | 5.815 | 6.683 | 7.846 | 7.37 |
| YCR035C | 1.38 | 0.4651 | 0.1732 | 0.3625 | no | up | 16.397 | 6.329 | 12.669 | 15.516 | 16.117 | 20.003 |
| YNL192W | 0.582 | -0.781 | 0.01119 | 0.04928 | no | down | 52.109 | 51.087 | 53.059 | 15.946 | 44.68 | 30.61 |
| YMR240C | 1 | 0 | 1 | 1 | no | no change | 0 | 0 | 0 | 0 | 0 | 0 |
| YKR014C | 0.979 | -0.03123 | 0.9414 | 0.972 | no | down | 42.579 | 10.552 | 38.867 | 34.265 | 34.828 | 22.1 |
| YOR047C | 1.71 | 0.7737 | 0.002256 | 0.01312 | no | up | 5.226 | 4.802 | 6.468 | 8.87 | 10.931 | 9.783 |
| YJL151C | 0.732 | -0.4502 | 0.09104 | 0.2355 | no | down | 2942.036 | 5460.626 | 3678.445 | 3183.439 | 2985.557 | 2424.753 |
| YER079W | 1 | 0 | 1 | 1 | no | no change | 0 | 0 | 0 | 1.458 | 0 | 0 |
| YJL144W | 1.814 | 0.8591 | 0.000965 | 0.006553 | no | up | 191.639 | 303.409 | 225.162 | 348.579 | 491.334 | 464.359 |
| YOR191W | 0.798 | -0.3253 | 0.0399 | 0.13 | no | down | 6.32 | 6.44 | 6.112 | 4.832 | 5.745 | 4.866 |
| YBR142W | 1.361 | 0.4451 | 0.1626 | 0.349 | no | up | 1.888 | 1.551 | 1.553 | 2.084 | 3.038 | 2.026 |
| YGL166W | 0.676 | -0.5655 | 0.003736 | 0.01983 | no | down | 297.243 | 403.392 | 301.443 | 214.35 | 254.592 | 209.749 |
| YPL268W | 1.315 | 0.3952 | 0.3043 | 0.5152 | no | up | 1.532 | 1.576 | 0.752 | 1.58 | 2.338 | 1.445 |
| YCR021C | 1.848 | 0.8859 | 0.004047 | 0.02107 | no | up | 673.644 | 1682.301 | 1309.813 | 2166.322 | 2967.396 | 2021.176 |
| YKL049C | 1.486 | 0.571 | 0.05857 | 0.1728 | no | up | 9.11 | 7.203 | 9.761 | 14.787 | 13.297 | 12.124 |
| YPL250C | 1.544 | 0.6269 | 0.01669 | 0.06661 | no | up | 402.1 | 634.634 | 406.839 | 731.488 | 608.319 | 899.687 |
| YDR464W | 1.318 | 0.3982 | 0.1987 | 0.3948 | no | up | 2.107 | 1.342 | 2.769 | 2.879 | 3.095 | 2.626 |
| YBL004W | 1.199 | 0.2623 | 0.4294 | 0.629 | no | up | 2.088 | 1.084 | 1.582 | 1.327 | 2.697 | 1.995 |
| YOR219C | 1 | 0 | 1 | 1 | no | no change | 0 | 0 | 0 | 0 | 0 | 0 |
| YER116C | 1.246 | 0.3173 | 0.3157 | 0.5269 | no | up | 16.205 | 8.927 | 18.474 | 17.684 | 17.594 | 21.123 |
| YDR526C | 1 | 0 | 1 | 1 | no | no change | 0 | 0 | 0 | 0 | 0 | 0 |
| YER002W | 1 | 0 | 1 | 1 | no | no change | 0 | 0 | 0 | 0 | 0 | 0 |
| YOR123C | 1.308 | 0.3869 | 0.115 | 0.2766 | no | up | 7.98 | 6.427 | 8.218 | 8.44 | 11.177 | 11.157 |
| YBR119W | 1.886 | 0.9153 | 0.000256 | 0.002083 | no | up | 9.01 | 10.651 | 12.145 | 17.497 | 24.446 | 20.837 |
| YPL033C | 1.428 | 0.5142 | 0.3036 | 0.515 | no | up | 1.067 | 1.515 | 1.741 | 1.617 | 3.369 | 1.812 |
| YOL108C | 0.995 | -0.00666 | 0.9767 | 0.9891 | no | down | 61.347 | 70.173 | 62.771 | 70.596 | 65.661 | 57.382 |
| YPR133C | 1.319 | 0.3996 | 0.1363 | 0.3102 | no | up | 9.037 | 6.144 | 9.395 | 9.767 | 12.218 | 11.798 |
| YLR067C | 0.819 | -0.2877 | 0.3932 | 0.5976 | no | down | 2.189 | 1.736 | 1.246 | 1.14 | 1.685 | 1.486 |
| YLR460C | 0.93 | -0.1042 | 0.6821 | 0.8253 | no | down | 7.204 | 8.989 | 8.218 | 8.216 | 7.287 | 7.451 |
| YJR025C | 0.768 | -0.3806 | 0.1234 | 0.2906 | no | down | 115.855 | 107.199 | 80.741 | 69.204 | 98.511 | 64.803 |
| YNL271C | 1.191 | 0.2527 | 0.2449 | 0.4476 | no | up | 3.62 | 3.177 | 3.333 | 4.141 | 4.997 | 3.339 |
| YPR126C | 1.96 | 0.9706 | 0.000144 | 0.001252 | no | up | 77.744 | 78.041 | 82.907 | 135.556 | 164.144 | 165.204 |
| 21S_rRNA | 0.716 | -0.481 | 0.45 | 0.648 | no | down | 0.036 | 0.283 | 0.574 | 0.159 | 0.303 | 0.071 |
| snR191 | 0.679 | -0.5583 | 0.2377 | 0.4411 | no | down | 37.253 | 103.529 | 75.855 | 35.032 | 60.295 | 38.092 |
| snR4 | 0.746 | -0.4232 | 0.2242 | 0.4256 | no | down | 1327.116 | 2798.368 | 1960.497 | 1310.017 | 1494.662 | 1398.799 |
| snR63 | 0.876 | -0.1902 | 0.6848 | 0.827 | no | down | 50.586 | 182.715 | 146.419 | 79.737 | 116.748 | 119.274 |
| snR81 | 0.674 | -0.5691 | 0.3587 | 0.5695 | no | down | 68.169 | 174.675 | 50.913 | 38.219 | 46.667 | 70.921 |
| YAR009C | 0.762 | -0.392 | 0.507 | 0.6963 | no | down | 0.219 | 0.222 | 0.129 | 0.215 | 0.104 | 0.081 |
| YAR029W | 1.254 | 0.3268 | 0.6181 | 0.7844 | no | up | 0 | 22.225 | 20.403 | 15.768 | 33.304 | 17.814 |
| YBL071C | 1.085 | 0.1175 | 0.5871 | 0.7632 | no | up | 228.053 | 215.358 | 201.269 | 227.323 | 244.617 | 201.901 |
| YBL093C | 0.531 | -0.9133 | 1.24E-05 | 0.000146 | no | down | 717.746 | 529.935 | 498.093 | 289.115 | 348.352 | 286.157 |
| YBR034C | 1.031 | 0.04399 | 0.9041 | 0.9545 | no | up | 193.654 | 56.505 | 106 | 128.331 | 110.71 | 138.36 |
| YBR210W | 0.721 | -0.4716 | 0.219 | 0.4207 | no | down | 20.61 | 20.575 | 18.553 | 9.702 | 20.433 | 11.584 |
| YCL035C | 0.575 | -0.7986 | 0.001988 | 0.01188 | no | down | 451.802 | 714.657 | 594.094 | 306.453 | 392.17 | 267.04 |
| YCL058C | 1.073 | 0.102 | 0.7375 | 0.8583 | no | up | 93.293 | 92.804 | 90.304 | 126.527 | 60.91 | 106.02 |
| YCL067C | 1.492 | 0.5774 | 0.0253 | 0.09084 | no | up | 19.598 | 19.443 | 27.247 | 31.461 | 39.314 | 31.363 |
| YCR039C | 1.492 | 0.5774 | 0.0253 | 0.09084 | no | up | 19.598 | 19.443 | 27.247 | 31.461 | 39.314 | 31.363 |
| YDL009C | 0.909 | -0.1373 | 0.7772 | 0.8791 | no | down | 18.96 | 23.53 | 16.022 | 11.113 | 25.837 | 14.231 |
| YDL010W | 1.039 | 0.05582 | 0.8357 | 0.9146 | no | up | 31.472 | 28.493 | 41.874 | 41.556 | 29.585 | 35.801 |
| YDL011C | 1.088 | 0.1219 | 0.7395 | 0.8592 | no | up | 41.85 | 33.27 | 38.481 | 28.003 | 44.973 | 49.33 |
| YDL031W | 1.348 | 0.4305 | 0.2759 | 0.4842 | no | up | 3.101 | 1.293 | 3.214 | 2.711 | 5.442 | 2.881 |
| YDL065C | 1 | 0 | 1 | 1 | no | no change | 0 | 0 | 0 | 0 | 0 | 0 |
| YDL153C | 1.511 | 0.5953 | 0.06101 | 0.1776 | no | up | 3.447 | 2.413 | 4.253 | 5.038 | 6.719 | 4.428 |
| YDL234C | 0.876 | -0.1911 | 0.4843 | 0.6761 | no | down | 14.026 | 14.874 | 16.427 | 9.038 | 18.994 | 12.714 |
| YDR028C | 1.1 | 0.1372 | 0.5907 | 0.7657 | no | up | 4.003 | 4.26 | 4.806 | 4.141 | 6.445 | 4.265 |
| YDR210W | 1 | 0 | 1 | 1 | no | no change | 0 | 0 | 0 | 0 | 0 | 0 |
| YDR289C | 1 | 0 | 1 | 1 | no | no change | 0 | 0 | 0 | 0 | 0 | 0 |
| YDR299W | 1.092 | 0.1265 | 0.6546 | 0.8082 | no | up | 10.478 | 7.203 | 10.543 | 9.58 | 13.713 | 8.571 |
| YDR345C | 1.929 | 0.9479 | 0.001323 | 0.008509 | no | up | 1730.273 | 3699.016 | 2146.95 | 5692.05 | 5788.658 | 3892.274 |
| YDR397C | 0.99 | -0.01472 | 0.9415 | 0.972 | no | down | 120.214 | 101.732 | 94.686 | 110.834 | 115.773 | 74.555 |
| YDR446W | 1.348 | 0.4312 | 0.4341 | 0.6342 | no | up | 0.958 | 1.047 | 0.613 | 1.767 | 0.861 | 1.232 |
| YDR457W | 0.8 | -0.3219 | 0.1626 | 0.349 | no | down | 3.712 | 3.817 | 3.185 | 2.299 | 3.767 | 2.708 |
| YDR485C | 0.826 | -0.275 | 0.3024 | 0.514 | no | down | 3.803 | 3.62 | 4.263 | 3.047 | 4.051 | 2.748 |
| YDR532C | 1 | 0 | 1 | 1 | no | no change | 0 | 0 | 0 | 0 | 0 | 0 |
| YEL012W | 1.079 | 0.1095 | 0.5684 | 0.7495 | no | up | 55.146 | 60.532 | 71.632 | 57.389 | 74.548 | 57.016 |
| YER044C-A | 1.02 | 0.02823 | 0.9602 | 0.9819 | no | up | 1.213 | 0.763 | 0.405 | 0.411 | 1.088 | 1.038 |
| YER132C | 0.966 | -0.05008 | 0.7905 | 0.8872 | no | down | 14.81 | 21.388 | 15.666 | 16.067 | 17.631 | 17.56 |
| YER149C | 1 | 0 | 1 | 1 | no | no change | 0 | 0 | 0 | 0 | 0 | 0 |
| YER158W-A | 0.942 | -0.08606 | 0.8384 | 0.9165 | no | down | 259.078 | 361.096 | 236.556 | 210.125 | 379.034 | 169.978 |
| YER162C | 1.042 | 0.05992 | 0.7843 | 0.8834 | no | up | 4.961 | 5.541 | 6.043 | 5.309 | 6.521 | 5.884 |
| YER165C-A | 1 | 0 | 1 | 1 | no | no change | 0.62 | 0.763 | 0.682 | 0 | 0.615 | 2.26 |
| YER167W | 1.513 | 0.5972 | 0.1024 | 0.2558 | no | up | 1.696 | 1.909 | 4.025 | 3.944 | 4.94 | 3.431 |
| YFL026W | 1 | 0 | 1 | 1 | no | no change | 0 | 0 | 0 | 0 | 0 | 0 |
| YFL034C-A | 1.116 | 0.1578 | 0.4206 | 0.6216 | no | up | 155.288 | 154.888 | 138.072 | 148.633 | 180.772 | 162.139 |
| YFL046W | 0.721 | -0.4727 | 0.1294 | 0.2991 | no | down | 19.169 | 18.322 | 22.499 | 13.973 | 10.306 | 18.425 |
| YFR039C | 1.024 | 0.0338 | 0.9302 | 0.9677 | no | up | 3.602 | 1.625 | 2.957 | 2.084 | 2.905 | 3.298 |
| YGL005C | 1 | 0 | 1 | 1 | no | no change | 0 | 0 | 0 | 0 | 0 | 0 |
| YGL013C | 1 | 0 | 1 | 1 | no | no change | 0 | 0 | 0 | 0 | 0 | 0 |
| YGL089C | 1 | 0 | 1 | 1 | no | no change | 0 | 0 | 0 | 0 | 0 | 0 |
| YGL094C | 0.918 | -0.1231 | 0.7052 | 0.8372 | no | down | 1.742 | 1.564 | 1.513 | 1.122 | 2.11 | 1.313 |
| YGL165C | 0.691 | -0.5323 | 0.03004 | 0.1047 | no | down | 129.252 | 202.49 | 139.675 | 97.749 | 132.506 | 93.794 |
| YGL183C | 0.762 | -0.3914 | 0.393 | 0.5974 | no | down | 5.855 | 4.15 | 7.358 | 2.15 | 6.313 | 4.581 |
| YGR114C | 1 | 0 | 1 | 1 | no | no change | 0 | 0.554 | 0.475 | 0 | 0.464 | 0 |
| YGR115C | 1 | 0 | 1 | 1 | no | no change | 0 | 0 | 0 | 0 | 0 | 0 |
| YGR139W | 1 | 0 | 1 | 1 | no | no change | 0 | 0 | 0 | 0 | 0 | 0 |
| YGR187C | 1.416 | 0.5018 | 0.1847 | 0.3766 | no | up | 25.909 | 8.287 | 13.697 | 17.058 | 27.36 | 28.319 |
| YGR264C | 0.78 | -0.3578 | 0.3841 | 0.5925 | no | down | 15.339 | 0 | 7.437 | 0 | 0 | 0 |
| YHR046C | 1.639 | 0.713 | 0.002546 | 0.01448 | no | up | 68.771 | 48.329 | 69.18 | 110.582 | 89.123 | 116.566 |
| YHR052W | 1 | 0 | 1 | 1 | no | no change | 0 | 0 | 0 | 0 | 0 | 0 |
| YHR068W | 1.871 | 0.9036 | 0.005048 | 0.02523 | no | up | 82.651 | 31.694 | 58.439 | 99.431 | 111.363 | 136.711 |
| YHR082C | 1 | 0 | 1 | 1 | no | no change | 0 | 0 | 0 | 0 | 0 | 0 |
| YHR088W | 1.008 | 0.01193 | 0.9647 | 0.9839 | no | up | 56.778 | 35.684 | 36.998 | 33.658 | 47.339 | 52.903 |
| YHR099W | 1.018 | 0.02625 | 0.9039 | 0.9545 | no | up | 1.131 | 1.17 | 1.226 | 1.094 | 1.391 | 1.079 |
| YHR162W | 0.774 | -0.3697 | 0.06719 | 0.1907 | no | down | 2752.002 | 3483.621 | 2603.407 | 2407.385 | 2292.167 | 1975.235 |
| YIL019W | 1.51 | 0.595 | 0.08712 | 0.2287 | no | up | 8.654 | 3.632 | 7.328 | 10.328 | 11.3 | 9.884 |
| YIL053W | 0.918 | -0.1241 | 0.6277 | 0.7901 | no | down | 6884.907 | 8151.463 | 7210.055 | 8364.921 | 4654.272 | 7455.984 |
| YIL158W | 1.01 | 0.01451 | 0.9805 | 0.9912 | no | up | 2.426 | 2.943 | 1.147 | 0.57 | 3.095 | 2.901 |
| YJL050W | 1.19 | 0.2512 | 0.4022 | 0.6049 | no | up | 15.175 | 8.545 | 10.948 | 9.982 | 19.344 | 13.936 |
| YJL202C | 1.762 | 0.8171 | 0.1379 | 0.3121 | no | up | 0 | 0 | 0 | 3.58 | 6.227 | 0 |
| YJL203W | 1 | 0 | 1 | 1 | no | no change | 0 | 0 | 0 | 0.308 | 0 | 0 |
| YJR047C | 1.51 | 0.5946 | 0.1054 | 0.2604 | no | up | 9.539 | 16.746 | 16.684 | 23.012 | 27.398 | 16.847 |
| YJR097W | 1.145 | 0.1949 | 0.5366 | 0.7226 | no | up | 31.955 | 16.684 | 23.696 | 29.022 | 25.307 | 29.49 |
| YKL061W | 0.872 | -0.1975 | 0.5946 | 0.7685 | no | down | 45.032 | 27.027 | 34.338 | 29.424 | 35.5 | 24.125 |
| YKL076C | 0.887 | -0.1731 | 0.6511 | 0.8056 | no | down | 29.11 | 33.307 | 27.187 | 18.451 | 39.446 | 19.626 |
| YKL082C | 1.41 | 0.4953 | 0.3185 | 0.53 | no | up | 3.082 | 0.714 | 2.789 | 4.365 | 3.833 | 1.965 |
| YKL106W | 0.786 | -0.3479 | 0.2129 | 0.413 | no | down | 49.191 | 25.07 | 32.815 | 29.022 | 26.395 | 29.836 |
| YKL152C | 0.728 | -0.4582 | 0.1114 | 0.2714 | no | down | 13191.46 | 27625.91 | 14958.02 | 10745.66 | 13553.15 | 16171.73 |
| YKL153W | 0.71 | -0.494 | 0.08191 | 0.2199 | no | down | 5734.693 | 11552.17 | 6472.251 | 4327.99 | 5872.244 | 6449.473 |
| YKL172W | 1.341 | 0.4233 | 0.1867 | 0.3789 | no | up | 9.366 | 7.277 | 10.276 | 8.936 | 18.644 | 10.434 |
| YKL202W | 1.812 | 0.8576 | 0.1909 | 0.3849 | no | up | 0.565 | 0.69 | 0 | 0.664 | 1.637 | 1.486 |
| YKR054C | 1.359 | 0.4428 | 0.01897 | 0.0735 | no | up | 123.306 | 144.187 | 102.944 | 180.01 | 180.318 | 158.414 |
| YLR106C | 0.817 | -0.2917 | 0.3237 | 0.5358 | no | down | 3.155 | 1.625 | 2.215 | 1.58 | 2.48 | 1.812 |
| YLR154W-E | 0.73 | -0.4533 | 0.3983 | 0.6021 | no | down | 89.007 | 298.25 | 171.737 | 102.656 | 136.537 | 124.791 |
| YLR223C | 0.593 | -0.7539 | 0.02227 | 0.08254 | no | down | 4.076 | 2.253 | 2.631 | 1.411 | 2.262 | 1.629 |
| YLR225C | 1.011 | 0.01554 | 0.9598 | 0.9818 | no | up | 25.571 | 18.95 | 23.825 | 14.207 | 33.947 | 23.331 |
| YLR331C | 1.985 | 0.9892 | 0.1245 | 0.2924 | no | up | 0 | 0 | 2.294 | 2.206 | 1.069 | 9.945 |
| YLR345W | 1.339 | 0.421 | 0.1285 | 0.2977 | no | up | 6.913 | 8.681 | 9.692 | 8.842 | 15.625 | 10.821 |
| YLR435W | 1.312 | 0.3915 | 0.2067 | 0.4053 | no | up | 61.265 | 27.569 | 41.419 | 48.491 | 59.538 | 68.63 |
| YLR447C | 1.025 | 0.03552 | 0.8768 | 0.9382 | no | up | 56.487 | 38.171 | 49.449 | 39.696 | 53.14 | 51.376 |
| YLR453C | 1.005 | 0.007633 | 0.9862 | 0.9937 | no | up | 1.696 | 1.662 | 1.444 | 1.066 | 2.281 | 1.649 |
| YML034C-A | 1 | 0 | 1 | 1 | no | no change | 0 | 0 | 0 | 0 | 0 | 0 |
| YML034W | 0.851 | -0.2321 | 0.1209 | 0.2866 | no | down | 11.272 | 12.818 | 11.017 | 9.861 | 11.234 | 10.444 |
| YML054C-A | 1 | 0 | 1 | 1 | no | no change | 0 | 100.857 | 0 | 17.834 | 0 | 0 |
| YML058W-A | 1 | 0 | 1 | 1 | no | no change | 0 | 0 | 0 | 0 | 0 | 0 |
| YMR114C | 1.038 | 0.05346 | 0.8149 | 0.9023 | no | up | 17.054 | 26.166 | 23.914 | 22.105 | 25.468 | 23.433 |
| YMR122W-A | 1.39 | 0.475 | 0.195 | 0.3896 | no | up | 1326.815 | 4027.519 | 2496.735 | 3512.362 | 2961.131 | 4373.552 |
| YMR141W-A | 0.889 | -0.1691 | 0.7935 | 0.8894 | no | down | 59.843 | 22.225 | 15.3 | 19.712 | 9.521 | 44.535 |
| YMR191W | 1.33 | 0.411 | 0.0711 | 0.198 | no | up | 451.09 | 649.053 | 538.592 | 877.653 | 737.749 | 611.739 |
| YMR193C-A | 0.785 | -0.3484 | 0.2651 | 0.4709 | no | down | 47.841 | 30.241 | 41.468 | 27.844 | 31.07 | 32.116 |
| YMR201C | 1.224 | 0.2913 | 0.0694 | 0.195 | no | up | 28.298 | 32.716 | 28.423 | 32.611 | 40.799 | 39.313 |
| YMR239C | 1.162 | 0.2169 | 0.5068 | 0.6962 | no | up | 10.278 | 5.504 | 8.218 | 7.991 | 13.221 | 7.909 |
| YMR269W | 1 | 0 | 1 | 1 | no | no change | 0 | 0 | 0 | 0 | 0 | 0 |
| YMR270C | 1 | 0 | 1 | 1 | no | no change | 0 | 0 | 0 | 0 | 0 | 0 |
| YMR280C | 1 | 0 | 1 | 1 | no | no change | 0 | 0 | 0 | 0 | 0 | 0 |
| YMR299C | 1.025 | 0.03494 | 0.8883 | 0.9463 | no | up | 31.262 | 21.351 | 26.465 | 21.871 | 29.244 | 32.096 |
| YMR311C | 0.771 | -0.3746 | 0.5106 | 0.6988 | no | down | 0.155 | 14.172 | 22.846 | 1.14 | 11.991 | 0 |
| YNL065W | 0.611 | -0.7115 | 0.000791 | 0.005569 | no | down | 1732.006 | 1118.714 | 1230.684 | 558.302 | 332.017 | 338.357 |
| YNL068C | 1 | 0 | 1 | 1 | no | no change | 0 | 0 | 0 | 0 | 0 | 0 |
| YNL093W | 0.686 | -0.543 | 0.1117 | 0.2716 | no | down | 24.176 | 53.058 | 56.975 | 34.181 | 32.386 | 23.555 |
| YNL113W | 1.2 | 0.2629 | 0.2567 | 0.462 | no | up | 178.36 | 135.063 | 148.486 | 214.471 | 161.513 | 173.103 |
| YNL129W | 1 | 0 | 1 | 1 | no | no change | 0 | 0 | 0 | 0 | 0 | 0 |
| YNL133C | 1 | 0 | 1 | 1 | no | no change | 0 | 0 | 0 | 0 | 0 | 0 |
| YNL162W-A | 1.13 | 0.1767 | 0.7559 | 0.8663 | no | up | 122.394 | 45.448 | 53.86 | 78.092 | 122.36 | 41.777 |
| YNL171C | 0.576 | -0.7958 | 0.1675 | 0.3541 | no | down | 12.549 | 18.113 | 7.932 | 2.196 | 12.086 | 3.39 |
| YNL248C | 1.471 | 0.557 | 0.07067 | 0.1971 | no | up | 119.184 | 49.13 | 79.238 | 111.255 | 125.124 | 147.715 |
| YNL308C | 1.344 | 0.4269 | 0.1566 | 0.3403 | no | up | 4.013 | 3.559 | 4.342 | 6.524 | 6.142 | 3.97 |
| YNR067C | 0.799 | -0.3242 | 0.09984 | 0.2516 | no | down | 5.672 | 7.191 | 6.102 | 4.832 | 6.104 | 5.365 |
| YOL014W | 1.242 | 0.3122 | 0.3484 | 0.5598 | no | up | 21.048 | 26.966 | 26.881 | 28.293 | 31.544 | 32.88 |
| YOL029C | 1.342 | 0.4241 | 0.2099 | 0.4083 | no | up | 10.442 | 6.637 | 7.744 | 9.87 | 12.881 | 11.91 |
| YOR075W | 1.277 | 0.3522 | 0.4805 | 0.6736 | no | up | 2.553 | 1.724 | 1.009 | 1.645 | 4.003 | 1.69 |
| YOR077W | 1.249 | 0.3213 | 0.4732 | 0.6683 | no | up | 4.332 | 2.66 | 6.28 | 5.057 | 4.164 | 8.317 |
| YOR139C | 1.395 | 0.4805 | 0.3778 | 0.5879 | no | up | 7.378 | 2.266 | 8.515 | 7.636 | 7.723 | 12.297 |
| YOR210W | 1.217 | 0.283 | 0.3224 | 0.5346 | no | up | 789.016 | 612.027 | 679.71 | 733.872 | 1022.237 | 642.196 |
| YOR242C | 1 | 0 | 1 | 1 | no | no change | 0 | 0 | 0 | 0 | 0 | 0 |
| YOR287C | 1.327 | 0.4084 | 0.3605 | 0.571 | no | up | 2.253 | 1.268 | 2.285 | 2.243 | 3.492 | 2.555 |
| YOR304W | 1 | 0 | 1 | 1 | no | no change | 0 | 0 | 0 | 0 | 0 | 0 |
| YOR308C | 1 | 0 | 1 | 1 | no | no change | 0 | 0 | 0 | 0 | 0 | 0 |
| YOR319W | 1.42 | 0.5057 | 0.06787 | 0.1917 | no | up | 14.974 | 11.784 | 11.828 | 19.525 | 16.874 | 19.921 |
| YPL043W | 1.335 | 0.417 | 0.2181 | 0.4191 | no | up | 6.165 | 3.682 | 4.53 | 3.963 | 8.981 | 7.482 |
| YPL081W | 1.33 | 0.411 | 0.0163 | 0.06568 | no | up | 466.521 | 485.964 | 432.207 | 575.865 | 579.548 | 739.38 |
| YPL095C | 1.046 | 0.06496 | 0.7565 | 0.8666 | no | up | 76.696 | 104.502 | 83.935 | 94.664 | 109.451 | 78.81 |
| YPL232W | 1.01 | 0.01499 | 0.9325 | 0.9682 | no | up | 157.212 | 135.913 | 136.619 | 139.865 | 133.348 | 159.554 |
| YPL257W-B | 0.967 | -0.04809 | 0.7938 | 0.8895 | no | down | 35.594 | 46.421 | 35.149 | 35.798 | 36.181 | 44.21 |
| YPR049C | 1.056 | 0.07898 | 0.8006 | 0.8935 | no | up | 2.289 | 2.364 | 2.522 | 2.43 | 3.634 | 1.751 |
| YPR087W | 0.958 | -0.06118 | 0.8826 | 0.9423 | no | down | 38.393 | 41.939 | 44.781 | 20.731 | 40.61 | 55.58 |
| ICR1 | 0.514 | -0.9593 | 0.002481 | 0.01423 | no | down | 1.35 | 1.551 | 1.523 | 0.776 | 0.786 | 0.672 |
| tS(AGA)H | 1 | 0 | 1 | 1 | no | no change | 0 | 0 | 0 | 0 | 0 | 0 |
| YAL021C | 1.153 | 0.2056 | 0.3546 | 0.5653 | no | up | 84.11 | 107.691 | 97.277 | 128.584 | 87.873 | 124.638 |
| YAL022C | 1.046 | 0.06444 | 0.7625 | 0.8702 | no | up | 90.794 | 133.044 | 87.476 | 115.61 | 110.748 | 105.155 |
| YAL040C | 1.681 | 0.7491 | 0.03043 | 0.1057 | no | up | 22.936 | 8.952 | 13.312 | 23.171 | 22.562 | 36.168 |
| YAL043C | 0.791 | -0.3376 | 0.08782 | 0.2302 | no | down | 56.368 | 74.125 | 60.556 | 56.436 | 45.058 | 51.671 |
| YAL054C | 0.504 | -0.9892 | 0.003757 | 0.01989 | no | down | 14.144 | 37.998 | 30.214 | 16.759 | 11.764 | 11.187 |
| YAL058W | 1.875 | 0.907 | 2.48E-05 | 0.000273 | no | up | 16.862 | 15.601 | 12.56 | 27.33 | 26.66 | 33.898 |
| YAL062W | 1.242 | 0.3131 | 0.1462 | 0.3256 | no | up | 1043.525 | 1526.219 | 1104.411 | 1661.194 | 1689.583 | 1318.381 |
| YAR008W | 0.807 | -0.31 | 0.2102 | 0.4088 | no | down | 17.665 | 18.47 | 15.082 | 11.169 | 16.004 | 14.628 |
| YAR035W | 0.667 | -0.5846 | 0.1283 | 0.2973 | no | down | 50.541 | 135.544 | 85.597 | 88.075 | 52.838 | 33.277 |
| YBL024W | 1.349 | 0.4319 | 0.197 | 0.3928 | no | up | 30.149 | 19.504 | 29.61 | 45.164 | 20.215 | 46.337 |
| YBL057C | 0.723 | -0.4681 | 0.02324 | 0.08516 | no | down | 146.597 | 185.56 | 126.551 | 96.973 | 120.07 | 115.202 |
| YBL082C | 0.9 | -0.1514 | 0.5122 | 0.7003 | no | down | 112.581 | 163.014 | 95.813 | 119.704 | 108.098 | 110.621 |
| YBL083C | 0.765 | -0.3868 | 0.3251 | 0.5373 | no | down | 36.615 | 75.517 | 33.012 | 47.818 | 27.853 | 29.958 |
| YBL100W-A | 0.696 | -0.5228 | 0.06533 | 0.1866 | no | down | 10.032 | 14.665 | 8.011 | 7.851 | 7.609 | 7.177 |
| YBL111C | 1.503 | 0.5882 | 0.3459 | 0.5575 | no | up | 2.891 | 0 | 2.641 | 7.104 | 4.278 | 8.123 |
| YBR003W | 0.826 | -0.2751 | 0.3484 | 0.5598 | no | down | 4.651 | 4.15 | 4.302 | 4.141 | 3.625 | 3.166 |
| YBR008C | 1.159 | 0.2123 | 0.349 | 0.5606 | no | up | 33.195 | 55.791 | 37.947 | 48.239 | 53.074 | 49.167 |
| YBR012W-A | 0.607 | -0.7194 | 0.03415 | 0.1157 | no | down | 38.065 | 42.825 | 22.44 | 10.954 | 25.818 | 25.449 |
| YBR029C | 1.971 | 0.9792 | 0.000114 | 0.001035 | no | up | 57.59 | 33.8 | 38.709 | 74.466 | 95.331 | 101.622 |
| YBR030W | 1.622 | 0.6981 | 0.02996 | 0.1045 | no | up | 13.232 | 6.107 | 10.641 | 16.002 | 13.959 | 21.876 |
| YBR031W | 0.751 | -0.4127 | 0.02952 | 0.1033 | no | down | 4561.826 | 5986.879 | 4410.749 | 4027.528 | 3530.373 | 3779.587 |
| YBR037C | 0.698 | -0.5178 | 0.1171 | 0.2804 | no | down | 46.118 | 119.376 | 68.913 | 59.773 | 59.746 | 41.278 |
| YBR089W | 1.434 | 0.5198 | 0.3601 | 0.5707 | no | up | 1.176 | 1.921 | 2.977 | 1.299 | 3.464 | 4.907 |
| YBR110W | 1.796 | 0.845 | 4.74E-06 | 6.07E-05 | no | up | 34.791 | 31.608 | 28.463 | 53.043 | 66.655 | 56.965 |
| YBR125C | 0.637 | -0.65 | 0.00288 | 0.01601 | no | down | 23.346 | 21.585 | 18.039 | 11.497 | 14.603 | 14.465 |
| YBR126C | 0.562 | -0.8324 | 0.003013 | 0.0166 | no | down | 66.618 | 141.786 | 117.739 | 66.886 | 60.882 | 52.699 |
| YBR157C | 0.87 | -0.2011 | 0.523 | 0.7099 | no | down | 21.741 | 12.116 | 20.195 | 15.497 | 12.956 | 19.036 |
| YBR219C | 1.085 | 0.1183 | 0.7339 | 0.8571 | no | up | 44.941 | 39.131 | 27.949 | 25.75 | 51.38 | 43.68 |
| YBR220C | 1.233 | 0.302 | 0.06734 | 0.1909 | no | up | 69.5 | 68.905 | 57.688 | 75.615 | 85.28 | 87.595 |
| YBR222C | 0.709 | -0.4961 | 0.01198 | 0.05181 | no | down | 173.6 | 255.425 | 201.674 | 149.044 | 164.977 | 138.228 |
| YBR232C | 1 | 0 | 1 | 1 | no | no change | 3.529 | 1.022 | 1.474 | 0 | 0.7 | 0 |
| YBR246W | 1.295 | 0.3732 | 0.0453 | 0.1428 | no | up | 97.178 | 105.586 | 83.461 | 130.976 | 112.47 | 135.836 |
| YBR294W | 0.968 | -0.0472 | 0.8637 | 0.9303 | no | down | 21.987 | 12.165 | 14.261 | 13.02 | 16.666 | 18.69 |
| YCL020W | 0.667 | -0.5842 | 0.3173 | 0.5287 | no | down | 0.137 | 0 | 0.593 | 0.131 | 0 | 0 |
| YCL036W | 0.8 | -0.3224 | 0.11 | 0.2694 | no | down | 78.82 | 74.421 | 63.523 | 65.166 | 50.633 | 59.957 |
| YCL057W | 0.836 | -0.2582 | 0.53 | 0.717 | no | down | 7.971 | 0 | 9.109 | 0 | 0 | 2.453 |
| YCR010C | 1.503 | 0.5882 | 0.06694 | 0.1904 | no | up | 5.609 | 10.799 | 8.99 | 14.992 | 13.041 | 11.503 |
| YCR017C | 1.295 | 0.373 | 0.02518 | 0.09057 | no | up | 44.923 | 49.179 | 52.713 | 68.362 | 63.333 | 63.622 |
| YCR034W | 1.514 | 0.5984 | 0.004235 | 0.02176 | no | up | 168.621 | 178.098 | 125.581 | 202.18 | 251.497 | 285.149 |
| YCR044C | 0.689 | -0.5383 | 0.3927 | 0.5974 | no | down | 21.312 | 29.983 | 19.542 | 0 | 13.013 | 12.46 |
| YCR065W | 1.676 | 0.7447 | 0.1757 | 0.366 | no | up | 2.553 | 0 | 0 | 18.843 | 24.881 | 25.205 |
| YCR067C | 1 | 0 | 1 | 1 | no | no change | 0 | 0 | 0 | 0 | 0 | 0 |
| YCR075C | 0.558 | -0.8406 | 0.01184 | 0.05134 | no | down | 14.683 | 35.019 | 19.918 | 11.87 | 13.647 | 12.042 |
| YCR079W | 0.535 | -0.9025 | 0.002692 | 0.01514 | no | down | 72.546 | 176.67 | 104.368 | 64.035 | 66.797 | 53.911 |
| YCR105W | 0.642 | -0.6392 | 0.02417 | 0.0879 | no | down | 13.296 | 19.344 | 12.877 | 10.655 | 7.496 | 10.739 |
| YDL021W | 0.575 | -0.7994 | 0.003419 | 0.01845 | no | down | 8.463 | 10.318 | 9.672 | 5.216 | 5.394 | 5.568 |
| YDL022W | 0.887 | -0.1726 | 0.4644 | 0.6612 | no | down | 101.291 | 179.108 | 144.254 | 122.611 | 117.155 | 141.933 |
| YDL023C | 0.898 | -0.1552 | 0.6528 | 0.8073 | no | down | 121.929 | 298.742 | 195.434 | 160.858 | 164.068 | 208.456 |
| YDL042C | 1.054 | 0.07555 | 0.7553 | 0.866 | no | up | 12.43 | 12.806 | 11.452 | 12.805 | 10.268 | 16.491 |
| YDL103C | 1.237 | 0.3071 | 0.3086 | 0.5192 | no | up | 7.934 | 5.947 | 6.656 | 9.599 | 6.085 | 10.556 |
| YDL142C | 0.684 | -0.5478 | 0.04455 | 0.1411 | no | down | 21.65 | 32.987 | 19.701 | 17.02 | 18.852 | 14.648 |
| YDL144C | 0.601 | -0.7355 | 0.0137 | 0.05764 | no | down | 208.756 | 490.631 | 276.886 | 210.789 | 197.968 | 166.629 |
| YDL145C | 1.526 | 0.6098 | 0.0649 | 0.1858 | no | up | 12.202 | 5.085 | 10.513 | 11.898 | 14.101 | 19.423 |
| YDL164C | 1.004 | 0.005313 | 0.9853 | 0.9937 | no | up | 4.222 | 3.115 | 4.193 | 3.374 | 3.473 | 5.1 |
| YDL171C | 0.67 | -0.5772 | 0.000297 | 0.002387 | no | down | 235.148 | 245.168 | 215.461 | 120.134 | 150.024 | 143.124 |
| YDL223C | 1 | 0 | 1 | 1 | no | no change | 0 | 0 | 0 | 0 | 0 | 0 |
| YDL228C | 1.152 | 0.204 | 0.2822 | 0.4926 | no | up | 1224.238 | 971.018 | 931.219 | 1089.162 | 1189.854 | 1378.318 |
| YDL229W | 1.204 | 0.2677 | 0.2926 | 0.5034 | no | up | 2597.626 | 1996.767 | 2217.88 | 3104.131 | 1961.276 | 3379.45 |
| YDL240W | 0.891 | -0.1668 | 0.7602 | 0.8688 | no | down | 0.146 | 0.283 | 0.356 | 0.206 | 0.237 | 0.244 |
| YDL244W | 1 | 0 | 1 | 1 | no | no change | 0 | 0 | 0 | 0 | 0 | 0 |
| YDR009W | 1.115 | 0.1569 | 0.5791 | 0.7567 | no | up | 4.295 | 5.381 | 5.608 | 6.963 | 5.044 | 5.395 |
| YDR010C | 0.608 | -0.717 | 0.1497 | 0.3311 | no | down | 16.306 | 30.586 | 19.295 | 15.46 | 7.798 | 11.452 |
| YDR012W | 0.764 | -0.3887 | 0.03858 | 0.1266 | no | down | 2195.399 | 2682.206 | 1870.42 | 1752.559 | 1645.178 | 1812.658 |
| YDR034C-A | 1 | 0 | 1 | 1 | no | no change | 21.778 | 0 | 47.372 | 0 | 0 | 0 |
| YDR034W-B | 1.354 | 0.4367 | 0.4849 | 0.6767 | no | up | 0 | 369.26 | 0 | 81.027 | 299.924 | 393.795 |
| YDR064W | 1.244 | 0.3153 | 0.08323 | 0.2224 | no | up | 7062.912 | 7638.176 | 6374.46 | 7509.728 | 8440.448 | 10294.02 |
| YDR074W | 1.009 | 0.01358 | 0.9662 | 0.9841 | no | up | 9.576 | 12.449 | 22.559 | 16.31 | 12.493 | 16.348 |
| YDR091C | 1.245 | 0.3156 | 0.3276 | 0.5396 | no | up | 80.334 | 33.689 | 58.232 | 70.288 | 58.914 | 95.067 |
| YDR098C-A | 0.804 | -0.3154 | 0.6163 | 0.7831 | no | down | 26.255 | 336.002 | 243.884 | 86.663 | 105.723 | 247.648 |
| YDR133C | 1.126 | 0.1713 | 0.5712 | 0.7517 | no | up | 1545.493 | 2252.254 | 1351.093 | 2557.756 | 1564.128 | 1495.423 |
| YDR134C | 1.19 | 0.2507 | 0.3679 | 0.5778 | no | up | 4756.337 | 6956.641 | 4744.849 | 8437.816 | 4872.531 | 6023.511 |
| YDR135C | 1.004 | 0.006464 | 0.9787 | 0.9901 | no | up | 9.448 | 7.4 | 8.238 | 6.197 | 10.656 | 8.999 |
| YDR146C | 0.652 | -0.6164 | 0.1592 | 0.3449 | no | down | 1.122 | 0.813 | 0.791 | 0.523 | 0.71 | 0.499 |
| YDR210W-A | 0.639 | -0.6469 | 0.01135 | 0.04981 | no | down | 25.134 | 45.066 | 27.504 | 20.161 | 20.783 | 21.285 |
| YDR233C | 0.878 | -0.1879 | 0.5714 | 0.7517 | no | down | 344.099 | 642.551 | 491.536 | 551.937 | 241.872 | 498.552 |
| YDR256C | 0.824 | -0.28 | 0.2374 | 0.4407 | no | down | 29.839 | 49.598 | 31.45 | 30.209 | 32.727 | 29.388 |
| YDR261C-C | 0.793 | -0.3348 | 0.4408 | 0.6392 | no | down | 20.61 | 124.572 | 63.167 | 47.155 | 69.911 | 44.922 |
| YDR284C | 0.587 | -0.7676 | 0.01168 | 0.05083 | no | down | 14.582 | 12.793 | 14.637 | 10.543 | 6.89 | 6.718 |
| YDR316W-A | 0.489 | -1.031 | 0.1028 | 0.2567 | no | down | 277.098 | 136.553 | 79.722 | 68.568 | 85.791 | 7.574 |
| YDR384C | 1 | 0 | 1 | 1 | no | no change | 0 | 0 | 0 | 34.733 | 0 | 0 |
| YDR385W | 1.025 | 0.03616 | 0.8494 | 0.9217 | no | up | 3422.655 | 4662.264 | 3340.231 | 4133.175 | 3757.982 | 4065.632 |
| YDR387C | 0.609 | -0.7166 | 0.00035 | 0.002756 | no | down | 32.174 | 38.232 | 33.062 | 22.61 | 22.893 | 17.885 |
| YDR470C | 0.529 | -0.9187 | 0.006178 | 0.02972 | no | down | 4.523 | 4.556 | 6.171 | 2.393 | 1.95 | 3.532 |
| YDR488C | 0.609 | -0.716 | 0.02095 | 0.07877 | no | down | 12.868 | 7.363 | 7.714 | 4.346 | 6.133 | 6.566 |
| YDR498C | 0.825 | -0.2772 | 0.2289 | 0.4317 | no | down | 19.133 | 24.737 | 21.797 | 19.03 | 15.038 | 20.634 |
| YDR508C | 1.197 | 0.2594 | 0.3355 | 0.5482 | no | up | 351.897 | 194.376 | 278.083 | 397.173 | 288.88 | 332.158 |
| YDR509W | 1.472 | 0.5578 | 0.08048 | 0.2171 | no | up | 151.284 | 87.399 | 119.984 | 220.107 | 121.736 | 178.406 |
| YDR513W | 0.571 | -0.8078 | 0.004596 | 0.02336 | no | down | 181.333 | 322.852 | 271.803 | 157.334 | 162.582 | 106.956 |
| YDR534C | 0.902 | -0.1481 | 0.7174 | 0.8457 | no | down | 1.268 | 1.945 | 1.276 | 1.094 | 1.192 | 1.761 |
| YDR536W | 0.933 | -0.1002 | 0.7506 | 0.8638 | no | down | 7.907 | 14.037 | 13.46 | 14.011 | 7.912 | 11.34 |
| YEL013W | 1.723 | 0.7853 | 0.08245 | 0.2208 | no | up | 0.109 | 0.16 | 0.099 | 0.318 | 0.265 | 0.112 |
| YEL020C | 1.519 | 0.6031 | 0.01064 | 0.04719 | no | up | 104.145 | 167.127 | 112.863 | 207.33 | 231.727 | 165.326 |
| YEL033W | 0.91 | -0.1368 | 0.7767 | 0.879 | no | down | 7.651 | 12.239 | 5.4 | 6.281 | 7.581 | 8.398 |
| YEL034C-A | 0.964 | -0.053 | 0.7895 | 0.8865 | no | down | 1544.663 | 1720.46 | 1517.283 | 1620.994 | 1258.014 | 1750.919 |
| YEL053W-A | 1.491 | 0.576 | 0.001519 | 0.009587 | no | up | 2991.774 | 2564.171 | 2514.151 | 3636.076 | 3568.579 | 4575.514 |
| YEL063C | 0.541 | -0.8875 | 0.000254 | 0.002076 | no | down | 688.372 | 764.895 | 833.171 | 530.019 | 335.339 | 361.149 |
| YER015W | 0.587 | -0.7677 | 0.1636 | 0.3503 | no | down | 0.347 | 0.406 | 0.405 | 0.187 | 0.161 | 0.244 |
| YER019W | 0.798 | -0.3261 | 0.1049 | 0.2599 | no | down | 55.438 | 101.695 | 62.741 | 50.482 | 57.541 | 54.043 |
| YER023W | 0.779 | -0.3598 | 0.09776 | 0.2476 | no | down | 173.591 | 164.615 | 111.657 | 117.629 | 118.366 | 117.431 |
| YER026C | 0.86 | -0.2176 | 0.2582 | 0.4637 | no | down | 180.166 | 208.044 | 145.401 | 137.547 | 162.223 | 165.173 |
| YER031C | 1.177 | 0.235 | 0.2104 | 0.4092 | no | up | 71.434 | 75.886 | 67.854 | 76.7 | 81.504 | 99.149 |
| YER043C | 1.135 | 0.1823 | 0.4724 | 0.6675 | no | up | 1499.64 | 909.378 | 891.423 | 1274.284 | 1150.162 | 1429.888 |
| YER046W | 0.998 | -0.00356 | 0.9891 | 0.9951 | no | down | 43.145 | 44.623 | 44.999 | 42.098 | 52.781 | 36.738 |
| YER066W | 0.782 | -0.3556 | 0.2484 | 0.4518 | no | down | 13.223 | 18.15 | 14.874 | 10.179 | 13.146 | 12.45 |
| YER070W | 1 | 0 | 1 | 1 | no | no change | 0 | 0 | 0 | 0.28 | 0 | 4.55 |
| YER082C | 1 | 0 | 1 | 1 | no | no change | 0 | 0 | 0 | 0 | 0 | 0 |
| YER087W | 1 | 0 | 1 | 1 | no | no change | 0 | 0 | 0 | 0 | 0 | 0 |
| YER089C | 1.055 | 0.07767 | 0.751 | 0.8639 | no | up | 71.89 | 137.858 | 96.317 | 102.385 | 117.496 | 109.644 |
| YER096W | 0.786 | -0.3472 | 0.07885 | 0.2138 | no | down | 17.309 | 17.768 | 14.301 | 12.581 | 13.004 | 13.865 |
| YER113C | 1.574 | 0.6543 | 0.01127 | 0.04959 | no | up | 17.291 | 11.008 | 17.08 | 22.245 | 22.269 | 30.528 |
| YER121W | 0.764 | -0.3876 | 0.5264 | 0.7133 | no | down | 3.411 | 5.738 | 9.207 | 4.767 | 3.445 | 3.797 |
| YER135C | 0.981 | -0.02716 | 0.9667 | 0.9841 | no | down | 0 | 2.179 | 3.264 | 0.794 | 3.653 | 0.845 |
| YER189W | 0.951 | -0.07189 | 0.8559 | 0.9258 | no | down | 16.725 | 27.2 | 19.978 | 20.535 | 16.988 | 20.654 |
| YFL037W | 1.164 | 0.2185 | 0.403 | 0.6053 | no | up | 91.934 | 55.508 | 72.73 | 73.494 | 78.968 | 112.85 |
| YFL064C | 1.328 | 0.4097 | 0.471 | 0.6669 | no | up | 1.669 | 1.244 | 4.727 | 4.982 | 3.066 | 3.288 |
| YFR014C | 1.362 | 0.4458 | 0.1531 | 0.3353 | no | up | 6.046 | 3.349 | 5.252 | 5.823 | 7.846 | 7.339 |
| YGL017W | 1 | 0 | 1 | 1 | no | no change | 0 | 0 | 0 | 0 | 0 | 0 |
| YGL021W | 1 | 0 | 1 | 1 | no | no change | 0 | 0 | 0 | 0 | 0 | 0 |
| YGL034C | 1 | 0 | 1 | 1 | no | no change | 0.556 | 0.677 | 0.584 | 0.981 | 0.568 | 1.038 |
| YGL055W | 0.511 | -0.9677 | 5.33E-05 | 0.000533 | no | down | 556.895 | 642.564 | 790.655 | 243.437 | 354.882 | 424.12 |
| YGL080W | 0.767 | -0.383 | 0.5555 | 0.7389 | no | down | 2.718 | 13.545 | 9.821 | 9.655 | 6.256 | 0 |
| YGL084C | 0.509 | -0.9749 | 0.000115 | 0.001044 | no | down | 10.214 | 7.93 | 9.91 | 3.823 | 5.309 | 5.192 |
| YGL105W | 1.183 | 0.2425 | 0.1441 | 0.3222 | no | up | 239.288 | 224.753 | 206.075 | 233.987 | 279.662 | 299.777 |
| YGL119W | 0.789 | -0.3417 | 0.244 | 0.4467 | no | down | 21.458 | 10.515 | 14.726 | 12.319 | 12.871 | 12.215 |
| YGL123W | 1.221 | 0.2885 | 0.1393 | 0.3141 | no | up | 5262.573 | 5233.3 | 4437.907 | 4964.898 | 6187.652 | 7508.979 |
| YGL137W | 0.93 | -0.1043 | 0.7329 | 0.8565 | no | down | 24.924 | 12.227 | 17.347 | 12.534 | 17.972 | 21.866 |
| YGL147C | 1.248 | 0.3194 | 0.2205 | 0.4224 | no | up | 1240.462 | 789.915 | 865.333 | 919.639 | 1172.866 | 1598.725 |
| YGL158W | 1.909 | 0.9331 | 0.000348 | 0.002747 | no | up | 2.718 | 3.349 | 3.422 | 6.169 | 6.398 | 6.444 |
| YGL199C | 0.625 | -0.679 | 0.0201 | 0.0766 | no | down | 141.399 | 304.32 | 182.784 | 112.423 | 133.045 | 136.528 |
| YGL202W | 0.582 | -0.782 | 1.38E-05 | 0.000159 | no | down | 738.092 | 785.113 | 670.651 | 449.786 | 370.715 | 466.701 |
| YGL205W | 0.881 | -0.1828 | 0.6179 | 0.7844 | no | down | 1.733 | 3.041 | 3.274 | 2.673 | 2.877 | 1.578 |
| YGL213C | 1.178 | 0.2366 | 0.3691 | 0.5791 | no | up | 24.404 | 15.194 | 18.336 | 21.142 | 20.906 | 28.503 |
| YGR027W-A | 1 | 0 | 1 | 1 | no | no change | 0 | 0 | 0 | 0 | 0 | 0 |
| YGR031W | 0.818 | -0.2896 | 0.5759 | 0.7545 | no | down | 5.691 | 15.81 | 4.035 | 8.206 | 0 | 0 |
| YGR036C | 0.662 | -0.5948 | 0.05753 | 0.1703 | no | down | 31.618 | 21.351 | 15.942 | 15.534 | 13.382 | 16.084 |
| YGR038C-A | 0.694 | -0.5266 | 0.05323 | 0.1605 | no | down | 44.695 | 61.48 | 31.361 | 36.172 | 30.219 | 28.716 |
| YGR060W | 0.659 | -0.6016 | 0.05764 | 0.1704 | no | down | 77.252 | 52.097 | 74.105 | 43.313 | 27.143 | 62.543 |
| YGR065C | 0.713 | -0.4873 | 0.1191 | 0.2833 | no | down | 322.149 | 153.348 | 146.38 | 144.398 | 132.515 | 169.367 |
| YGR067C | 0.721 | -0.471 | 0.2226 | 0.424 | no | down | 1.322 | 1.231 | 1.968 | 1.131 | 0.767 | 1.334 |
| YGR069W | 1 | 0 | 1 | 1 | no | no change | 0 | 0 | 0 | 0 | 0 | 0 |
| YGR077C | 0.578 | -0.7902 | 0.000315 | 0.002523 | no | down | 12.977 | 11.488 | 13.727 | 7.029 | 6.748 | 8.5 |
| YGR113W | 0.705 | -0.5039 | 0.1842 | 0.3765 | no | down | 6.967 | 3.669 | 5.519 | 3.337 | 2.972 | 4.998 |
| YGR121C | 0.511 | -0.9692 | 4.41E-05 | 0.000453 | no | down | 702.27 | 1024.777 | 696.76 | 482.369 | 406.224 | 337.431 |
| YGR131W | 1 | 0 | 1 | 1 | no | no change | 0 | 0 | 0 | 0 | 0 | 0 |
| YGR154C | 1 | 0 | 1 | 1 | no | no change | 0 | 0 | 0 | 0 | 0 | 0 |
| YGR161C-C | 0.489 | -1.031 | 0.1028 | 0.2567 | no | down | 277.098 | 136.553 | 79.722 | 68.568 | 85.791 | 7.574 |
| YGR190C | 1.386 | 0.4705 | 0.4565 | 0.654 | no | up | 141.107 | 0 | 68.111 | 207.227 | 139.963 | 149.334 |
| YGR194C | 1 | 0 | 1 | 1 | no | no change | 0 | 0 | 0 | 0 | 0 | 0 |
| YGR241C | 1.094 | 0.1297 | 0.6778 | 0.8226 | no | up | 4.049 | 8.471 | 7.408 | 7.702 | 6.417 | 8.154 |
| YGR242W | 1.153 | 0.2056 | 0.7063 | 0.8372 | no | up | 6.247 | 19.578 | 16.061 | 14.151 | 19.609 | 15.167 |
| YGR259C | 1.341 | 0.4237 | 0.09952 | 0.2512 | no | up | 242.471 | 308.285 | 220.653 | 326.978 | 264.822 | 445.588 |
| YGR266W | 1 | 0 | 1 | 1 | no | no change | 0 | 0 | 0 | 0 | 0 | 0 |
| YHL016C | 1.026 | 0.03747 | 0.9316 | 0.9679 | no | up | 55.675 | 0 | 38.224 | 109.077 | 5.442 | 0 |
| YHL032C | 0.77 | -0.378 | 0.2186 | 0.42 | no | down | 48.106 | 118.12 | 79.317 | 70.615 | 67.545 | 50.307 |
| YHL033C | 1.193 | 0.2546 | 0.2181 | 0.4191 | no | up | 2117.828 | 2145.622 | 1778.968 | 2699.453 | 1976.646 | 2642.3 |
| YHL046C | 0.498 | -1.005 | 0.125 | 0.2931 | no | down | 3.429 | 5.861 | 1.919 | 0.542 | 1.363 | 1.069 |
| YHR007C | 0.669 | -0.5796 | 0.03642 | 0.1214 | no | down | 149.698 | 113.06 | 149.604 | 101.843 | 60.314 | 114.235 |
| YHR020W | 0.834 | -0.2624 | 0.09625 | 0.2449 | no | down | 565.34 | 676.622 | 601.274 | 542.384 | 502.492 | 519.736 |
| YHR033W | 0.523 | -0.936 | 0.00073 | 0.005199 | no | down | 212.541 | 369.506 | 285.105 | 192.432 | 128.342 | 122.419 |
| YHR034C | 0.925 | -0.1132 | 0.5948 | 0.7685 | no | down | 37.673 | 53.747 | 37.631 | 39.35 | 43.184 | 38.407 |
| YHR039C | 1.043 | 0.06029 | 0.7579 | 0.8675 | no | up | 43.573 | 40.954 | 48.164 | 49.547 | 40.515 | 51.59 |
| YHR071C-A | 1.488 | 0.5732 | 0.1526 | 0.3349 | no | up | 49.145 | 38.109 | 24.2 | 48.454 | 42.796 | 75.532 |
| YHR074W | 0.652 | -0.6165 | 0.002798 | 0.01561 | no | down | 48.151 | 64.152 | 53.959 | 41.687 | 34.26 | 33.155 |
| YHR095W | 1 | 0 | 1 | 1 | no | no change | 0 | 0 | 0 | 0 | 0 | 0 |
| YHR096C | 0.589 | -0.7627 | 0.000104 | 0.000952 | no | down | 171.758 | 256.705 | 215.688 | 114.292 | 141.941 | 127.214 |
| YHR105W | 0.574 | -0.801 | 0.0316 | 0.1091 | no | down | 8.882 | 8.274 | 8.416 | 3.29 | 6.275 | 4.622 |
| YHR108W | 0.94 | -0.08897 | 0.7391 | 0.8591 | no | down | 25.726 | 21.597 | 24.982 | 26.433 | 15.672 | 27.057 |
| YHR117W | 0.737 | -0.4399 | 0.09144 | 0.2362 | no | down | 12.986 | 8.632 | 9.603 | 6.571 | 7.496 | 9.365 |
| YHR136C | 1 | 0 | 1 | 1 | no | no change | 0 | 0 | 0 | 0 | 0 | 0 |
| YHR139C | 1 | 0 | 1 | 1 | no | no change | 0 | 0 | 0 | 0 | 0 | 0 |
| YHR203C | 1.072 | 0.1006 | 0.6638 | 0.8145 | no | up | 1862.48 | 2555.749 | 2147.336 | 2468.205 | 1815.994 | 2852.599 |
| YHR214C-C | 0.722 | -0.4697 | 0.06311 | 0.1823 | no | down | 79.431 | 144.175 | 87.209 | 64.661 | 86.416 | 74.82 |
| YIL018W | 0.807 | -0.3093 | 0.1907 | 0.3847 | no | down | 7403.656 | 12288.92 | 7304.285 | 6771.977 | 7108.359 | 8010.758 |
| YIL039W | 1.449 | 0.5348 | 0.1205 | 0.2861 | no | up | 46.318 | 18.162 | 26.772 | 36.256 | 41.433 | 63.001 |
| YIL057C | 1 | 0 | 1 | 1 | no | no change | 0 | 0 | 0 | 0 | 0 | 0 |
| YIL082W-A | 1 | 0 | 1 | 1 | no | no change | 0 | 0 | 0 | 0 | 0 | 0 |
| YIL099W | 0.781 | -0.3571 | 0.3847 | 0.5925 | no | down | 0 | 6.427 | 3.758 | 0 | 0 | 0 |
| YIL101C | 0.55 | -0.8638 | 0.004743 | 0.02398 | no | down | 57.006 | 152.4 | 112.121 | 53.791 | 59.141 | 60.477 |
| YIL109C | 1.222 | 0.2893 | 0.1616 | 0.3482 | no | up | 41.184 | 29.761 | 36.286 | 41.752 | 42.456 | 51.396 |
| YIL113W | 0.969 | -0.04561 | 0.8698 | 0.9341 | no | down | 36.36 | 62.071 | 33.873 | 42.715 | 45.03 | 40.901 |
| YIL155C | 0.592 | -0.7569 | 2.97E-05 | 0.000321 | no | down | 37.782 | 37.358 | 40.281 | 25.04 | 21.625 | 22.375 |
| YIL171W | 1 | 0 | 1 | 1 | no | no change | 0 | 0 | 0 | 0 | 0 | 0 |
| YIR008C | 1.535 | 0.6185 | 0.01158 | 0.05055 | no | up | 22.316 | 14.123 | 16.892 | 24.33 | 30.417 | 30.834 |
| YIR014W | 1.005 | 0.007765 | 0.9855 | 0.9937 | no | up | 3.365 | 3.509 | 2.314 | 3.112 | 3.275 | 2.972 |
| YIR016W | 0.927 | -0.1097 | 0.7275 | 0.8531 | no | down | 77.826 | 197.577 | 105.535 | 112.675 | 136.869 | 105.592 |
| YIR017W-A | 1 | 0 | 1 | 1 | no | no change | 0 | 0 | 0 | 0 | 0 | 0 |
| YIR020C-B | 1.098 | 0.1344 | 0.738 | 0.8584 | no | up | 7.715 | 5.615 | 3.946 | 4.645 | 6 | 9.039 |
| YJL051W | 0.937 | -0.09341 | 0.6111 | 0.7796 | no | down | 21.641 | 25.858 | 21.51 | 22.638 | 19.799 | 23.535 |
| YJL079C | 0.625 | -0.6792 | 0.02134 | 0.07972 | no | down | 270.277 | 548.885 | 288.359 | 171.691 | 231.518 | 282.024 |
| YJL088W | 0.933 | -0.1005 | 0.6947 | 0.8329 | no | down | 205.847 | 205.31 | 208.943 | 166.13 | 146.323 | 276.802 |
| YJL091C | 1.07 | 0.09783 | 0.6326 | 0.7932 | no | up | 12.357 | 12.128 | 11.116 | 11.085 | 14.092 | 13.976 |
| YJL097W | 1.081 | 0.1128 | 0.6972 | 0.8341 | no | up | 39.652 | 71.269 | 42.685 | 63.782 | 45.342 | 58.013 |
| YJL119C | 1 | 0 | 1 | 1 | no | no change | 0 | 0 | 0 | 0 | 0 | 0 |
| YJL160C | 1 | 0 | 1 | 1 | no | no change | 0 | 0 | 0 | 0 | 0 | 0 |
| YJL180C | 1.24 | 0.3101 | 0.284 | 0.4947 | no | up | 7.515 | 5.75 | 5.795 | 8.15 | 9.048 | 7.227 |
| YJL216C | 0.551 | -0.8594 | 0.007247 | 0.03426 | no | down | 2.709 | 2.832 | 2.492 | 1.318 | 1.448 | 1.588 |
| YJR001W | 0.643 | -0.6374 | 0.00175 | 0.01073 | no | down | 57.499 | 84.358 | 70.01 | 49.201 | 43.856 | 44.037 |
| YJR024C | 1.052 | 0.07282 | 0.8012 | 0.8936 | no | up | 265.206 | 558.366 | 282.84 | 421.867 | 381.126 | 371.583 |
| YJR028W | 1 | 0 | 1 | 1 | no | no change | 0 | 0 | 0 | 0 | 0 | 0 |
| YJR038C | 1 | 0 | 1 | 1 | no | no change | 2.854 | 0.69 | 0.593 | 0 | 0 | 1.069 |
| YJR040W | 0.7 | -0.5141 | 0.01392 | 0.05838 | no | down | 10.807 | 9.038 | 9.237 | 6.748 | 6.436 | 7.502 |
| YJR044C | 0.556 | -0.8481 | 0.005447 | 0.02678 | no | down | 37.153 | 54.264 | 39.312 | 24.021 | 24.928 | 20.257 |
| YJR094C | 0.604 | -0.7284 | 0.008753 | 0.04006 | no | down | 12.603 | 9.087 | 11.907 | 7.505 | 6.644 | 6.036 |
| YJR095W | 0.612 | -0.7092 | 0.1336 | 0.3059 | no | down | 3.046 | 1.724 | 2.383 | 0.841 | 1.58 | 1.72 |
| YJR116W | 1 | 0 | 1 | 1 | no | no change | 0 | 0 | 0 | 0 | 0 | 3.929 |
| YJR123W | 1.06 | 0.0834 | 0.7215 | 0.8492 | no | up | 3946.775 | 3760.951 | 3334.782 | 3494.163 | 3111.221 | 5267.488 |
| YJR138W | 0.943 | -0.08498 | 0.7526 | 0.8646 | no | down | 1.432 | 1.859 | 1.79 | 1.832 | 1.675 | 1.354 |
| YJR143C | 1.282 | 0.3582 | 0.04404 | 0.1402 | no | up | 122.74 | 160.97 | 127.836 | 175.14 | 167.844 | 198.674 |
| YJR154W | 0.719 | -0.4753 | 0.161 | 0.3474 | no | down | 57.107 | 24.626 | 35.9 | 23.573 | 22.6 | 38.58 |
| YKL039W | 1.004 | 0.005117 | 0.978 | 0.9899 | no | up | 41.786 | 37.174 | 47.798 | 40.425 | 43.279 | 45.808 |
| YKL081W | 1.476 | 0.5614 | 0.00169 | 0.01041 | no | up | 506.227 | 431.811 | 385.121 | 637.815 | 649.942 | 723.194 |
| YKL100C | 0.617 | -0.6962 | 0.01476 | 0.06097 | no | down | 21.312 | 31.091 | 37.077 | 21.703 | 13.448 | 19.565 |
| YKL103C | 1.118 | 0.161 | 0.4365 | 0.636 | no | up | 40.482 | 50.139 | 39.975 | 38.387 | 55.942 | 55.886 |
| YKL112W | 1.455 | 0.541 | 0.04735 | 0.1471 | no | up | 8.089 | 6.637 | 11.245 | 11.609 | 11.707 | 16.277 |
| YKL182W | 1 | 0 | 1 | 1 | no | no change | 36.378 | 0 | 0.326 | 0 | 0 | 0 |
| YKL185W | 1 | 0 | 1 | 1 | no | no change | 0 | 0 | 0 | 0 | 0 | 0 |
| YKL187C | 0.575 | -0.7977 | 0.000393 | 0.003045 | no | down | 7.952 | 11.611 | 9.534 | 5.505 | 5.924 | 5.344 |
| YKL210W | 0.698 | -0.5184 | 0.07225 | 0.2005 | no | down | 29.091 | 14.296 | 22.806 | 12.328 | 17.054 | 17.59 |
| YKR003W | 0.897 | -0.1561 | 0.6546 | 0.8082 | no | down | 3.684 | 2.586 | 2.611 | 2.355 | 3.312 | 2.463 |
| YKR009C | 0.697 | -0.5216 | 0.04257 | 0.1367 | no | down | 5.244 | 7.252 | 8.228 | 5.328 | 4.912 | 4.275 |
| YKR039W | 0.649 | -0.6228 | 0.01116 | 0.04919 | no | down | 690.287 | 687.617 | 847.413 | 571.799 | 529.266 | 348.17 |
| YKR053C | 1 | 0 | 1 | 1 | no | no change | 0 | 0 | 0 | 0 | 0 | 0 |
| YKR059W | 1.367 | 0.4509 | 0.04978 | 0.1527 | no | up | 1023.179 | 740.872 | 769.52 | 957.643 | 1126.824 | 1464.885 |
| YKR067W | 1.17 | 0.227 | 0.4933 | 0.6843 | no | up | 3.164 | 6.403 | 7.259 | 7.449 | 5.432 | 7.39 |
| YKR070W | 0.574 | -0.8015 | 8.72E-06 | 0.000105 | no | down | 35.794 | 35.031 | 35.979 | 19.563 | 22.288 | 20.043 |
| YKR077W | 0.819 | -0.2887 | 0.3541 | 0.565 | no | down | 10.068 | 19.036 | 17.159 | 12.179 | 9.852 | 15.931 |
| YKR097W | 1.222 | 0.2894 | 0.2699 | 0.4772 | no | up | 48.981 | 54.375 | 88.663 | 82.635 | 72.845 | 86.689 |
| YLL002W | 0.745 | -0.4254 | 0.3638 | 0.5738 | no | down | 0 | 3.189 | 1.958 | 0.318 | 0 | 0 |
| YLL007C | 0.888 | -0.172 | 0.6934 | 0.8315 | no | down | 0.775 | 1.342 | 1.236 | 0.785 | 0.757 | 1.445 |
| YLL048C | 0.967 | -0.04876 | 0.8251 | 0.9082 | no | down | 30.988 | 24.195 | 23.271 | 19.993 | 30.048 | 28.248 |
| YLL055W | 0.65 | -0.6211 | 0.02294 | 0.08447 | no | down | 18.586 | 14.419 | 16.556 | 7.104 | 11.896 | 13.59 |
| YLR027C | 0.648 | -0.6255 | 0.002244 | 0.01308 | no | down | 498.53 | 740.059 | 585.025 | 426.952 | 400.867 | 359.775 |
| YLR044C | 0.758 | -0.3995 | 0.1462 | 0.3256 | no | down | 14564.31 | 22819.01 | 14839.67 | 10209.02 | 11105.64 | 18685.62 |
| YLR050C | 0.717 | -0.4805 | 0.1135 | 0.274 | no | down | 36.679 | 53.747 | 39.451 | 30.742 | 22.685 | 37.216 |
| YLR056W | 1.411 | 0.4964 | 0.02051 | 0.0777 | no | up | 351.149 | 548.269 | 425.037 | 692.783 | 636.275 | 584.468 |
| YLR060W | 1.152 | 0.2045 | 0.5469 | 0.7306 | no | up | 242.143 | 245.611 | 319.957 | 443.841 | 158.929 | 350.848 |
| YLR062C | 1.485 | 0.5704 | 0.019 | 0.07353 | no | up | 1593.982 | 1250.958 | 1187.96 | 1570.456 | 1735.54 | 2652.774 |
| YLR096W | 1.443 | 0.5293 | 0.03985 | 0.1299 | no | up | 11.655 | 6.637 | 9.623 | 12.356 | 13.997 | 15.931 |
| YLR099C | 0.639 | -0.6463 | 0.001416 | 0.009017 | no | down | 105.632 | 163.027 | 128.271 | 83.112 | 87.788 | 84.266 |
| YLR120C | 0.556 | -0.8463 | 2.14E-06 | 2.95E-05 | no | down | 671.145 | 840.806 | 604.785 | 359.58 | 423.221 | 407.568 |
| YLR124W | 1.156 | 0.2096 | 0.7455 | 0.862 | no | up | 0.684 | 0.825 | 2.126 | 1.785 | 0.691 | 2.535 |
| YLR153C | 1.048 | 0.06775 | 0.791 | 0.8873 | no | up | 43.674 | 26.67 | 37.839 | 38.069 | 32.433 | 46.276 |
| YLR183C | 1.61 | 0.6874 | 0.2753 | 0.4839 | no | up | 0.164 | 0.16 | 0.188 | 0.243 | 0.274 | 0.611 |
| YLR190W | 0.915 | -0.1285 | 0.6033 | 0.7745 | no | down | 6.065 | 6.661 | 6.656 | 6.496 | 5.366 | 6.128 |
| YLR232W | 0.538 | -0.8944 | 0.003804 | 0.02006 | no | down | 56.924 | 61.898 | 71.612 | 26.844 | 36.105 | 33.592 |
| YLR256W-A | 0.573 | -0.8036 | 4.61E-05 | 0.00047 | no | down | 65.068 | 66.097 | 52.99 | 38.658 | 35.149 | 32.167 |
| YLR264W | 1.268 | 0.3429 | 0.1774 | 0.3683 | no | up | 3066.618 | 1726.099 | 2082.201 | 2165.574 | 2870.607 | 3203.354 |
| YLR274W | 1.748 | 0.806 | 0.02458 | 0.08895 | no | up | 6.429 | 2.758 | 4.045 | 5.216 | 10.145 | 9.976 |
| YLR284C | 0.955 | -0.06685 | 0.882 | 0.9419 | no | down | 2.225 | 1.872 | 2.037 | 1.795 | 2.319 | 1.822 |
| YLR304C | 0.8 | -0.3219 | 0.3405 | 0.5524 | no | down | 162.52 | 197.75 | 334.03 | 264.392 | 133.32 | 155.93 |
| YLR317W | 0.658 | -0.6046 | 0.3179 | 0.5295 | no | down | 6.302 | 2.98 | 3.728 | 3.832 | 1.931 | 0.825 |
| YLR339C | 1.016 | 0.02289 | 0.9142 | 0.9605 | no | up | 3180.713 | 4747.163 | 3122.338 | 3500.902 | 3764.38 | 4016.974 |
| YLR340W | 1.006 | 0.00909 | 0.9663 | 0.9841 | no | up | 3792.709 | 5523.374 | 3556.572 | 3752.77 | 4556.727 | 4879.648 |
| YLR352W | 0.881 | -0.1831 | 0.4548 | 0.6522 | no | down | 3.967 | 3.657 | 4.371 | 3.533 | 3.246 | 4.001 |
| YLR376C | 1 | 0 | 1 | 1 | no | no change | 0 | 0 | 0 | 0 | 0 | 0 |
| YLR377C | 1.164 | 0.2189 | 0.4681 | 0.6638 | no | up | 25.672 | 59.744 | 55.274 | 56.856 | 53.633 | 57.168 |
| YLR378C | 0.583 | -0.7777 | 0.007921 | 0.03681 | no | down | 71.78 | 170.119 | 108.996 | 70.306 | 59.33 | 71.796 |
| YLR379W | 0.649 | -0.6248 | 0.06698 | 0.1904 | no | down | 75.373 | 164.566 | 92.975 | 77.896 | 61.289 | 64.151 |
| YLR392C | 0.798 | -0.3248 | 0.3599 | 0.5706 | no | down | 2.061 | 2.832 | 2.433 | 1.402 | 2.385 | 2.107 |
| YLR415C | 0.513 | -0.9619 | 0.1176 | 0.281 | no | down | 11.627 | 6.095 | 6.784 | 1.897 | 2.196 | 4.703 |
| YLR416C | 0.873 | -0.1959 | 0.7513 | 0.864 | no | down | 4.651 | 4.704 | 2.225 | 2.664 | 1.315 | 5.232 |
| YLR432W | 1.061 | 0.08582 | 0.6923 | 0.8309 | no | up | 757.234 | 529.787 | 568.202 | 648.068 | 600.303 | 773.746 |
| YML008C | 0.847 | -0.2402 | 0.3624 | 0.5726 | no | down | 22.507 | 15.084 | 17.851 | 13.441 | 14.537 | 19.86 |
| YML045W-A | 0.571 | -0.8073 | 0.000805 | 0.005649 | no | down | 144.892 | 132.601 | 89.76 | 55.314 | 73.933 | 81.681 |
| YML054C | 1.126 | 0.1713 | 0.6951 | 0.8332 | no | up | 6.603 | 5.997 | 9.445 | 12.029 | 2.763 | 10.77 |
| YML057W | 0.892 | -0.1655 | 0.4398 | 0.6385 | no | down | 113.092 | 160.219 | 114.475 | 132.257 | 115.849 | 103.088 |
| YML075C | 0.732 | -0.4504 | 0.03725 | 0.1233 | no | down | 5.408 | 4.765 | 5.894 | 3.57 | 4.259 | 4.184 |
| YML086C | 0.898 | -0.1547 | 0.3144 | 0.5256 | no | down | 51.06 | 55.077 | 48.431 | 43.855 | 49.043 | 48.943 |
| YML126C | 1 | 0 | 1 | 1 | no | no change | 0 | 0 | 0 | 0 | 0 | 0 |
| YMR026C | 0.794 | -0.3334 | 0.1239 | 0.2914 | no | down | 12.503 | 12.658 | 13.233 | 9.02 | 10.524 | 11.432 |
| YMR031C | 1.276 | 0.3518 | 0.3304 | 0.5428 | no | up | 0.812 | 1.194 | 0.771 | 1.262 | 1.183 | 1.242 |
| YMR084W | 0.89 | -0.1674 | 0.6226 | 0.7867 | no | down | 4.542 | 6.588 | 6.023 | 5.019 | 5.243 | 5.049 |
| YMR092C | 0.717 | -0.4793 | 0.007248 | 0.03426 | no | down | 54.033 | 72.906 | 65.659 | 43.855 | 46.27 | 50.195 |
| YMR116C | 1.099 | 0.1361 | 0.5477 | 0.7311 | no | up | 2786.164 | 4853.857 | 2716.666 | 3442.803 | 3509.845 | 4008.056 |
| YMR117C | 1 | 0 | 1 | 1 | no | no change | 0 | 0 | 0 | 0 | 0 | 0 |
| YMR118C | 1 | 0 | 1 | 1 | no | no change | 0 | 0 | 0 | 0 | 0 | 0 |
| YMR120C | 0.744 | -0.4263 | 0.3543 | 0.565 | no | down | 205.582 | 489.018 | 480.291 | 0 | 0 | 57.504 |
| YMR122C | 1 | 0 | 1 | 1 | no | no change | 0 | 0 | 0 | 0 | 0 | 0 |
| YMR133W | 0.95 | -0.074 | 0.6298 | 0.7918 | no | down | 20.957 | 31.152 | 24.309 | 19.039 | 19.666 | 12.124 |
| YMR148W | 0.888 | -0.1719 | 0.5595 | 0.7422 | no | down | 54.991 | 46.445 | 53.168 | 37.752 | 35.963 | 61.637 |
| YMR152W | 1.019 | 0.02682 | 0.8676 | 0.9332 | no | up | 33.688 | 30.98 | 29.808 | 31.172 | 31.733 | 36.005 |
| YMR196W | 0.541 | -0.8854 | 3.72E-06 | 4.88E-05 | no | down | 20.884 | 17.694 | 20.828 | 10.319 | 12.265 | 10.037 |
| YMR199W | 1 | 0 | 1 | 1 | no | no change | 0 | 0 | 0 | 0 | 0 | 0 |
| YMR209C | 0.84 | -0.2518 | 0.421 | 0.6218 | no | down | 11.764 | 7.031 | 7.279 | 5.645 | 7.41 | 9.294 |
| YMR217W | 1.188 | 0.2491 | 0.3121 | 0.5232 | no | up | 1455.072 | 832.716 | 996.295 | 1319.485 | 1250.509 | 1461.709 |
| YMR246W | 0.776 | -0.3654 | 0.1658 | 0.3525 | no | down | 177.175 | 170.217 | 197.866 | 127.322 | 101.227 | 201.178 |
| YMR267W | 1 | 0 | 1 | 1 | no | no change | 0 | 0 | 0 | 0 | 0 | 0 |
| YMR301C | 0.633 | -0.66 | 0.005998 | 0.029 | no | down | 22.498 | 15.859 | 15.962 | 10.609 | 10.524 | 13.641 |
| YMR303C | 0.772 | -0.3737 | 0.1155 | 0.2773 | no | down | 99.093 | 175.771 | 123.485 | 89.851 | 106.385 | 113.919 |
| YNL045W | 1 | 0 | 1 | 1 | no | no change | 0 | 0 | 0.524 | 0 | 0 | 0 |
| YNL069C | 1.248 | 0.3198 | 0.1541 | 0.3366 | no | up | 2106.72 | 1811.602 | 1738.796 | 2028.625 | 2029.805 | 3122.773 |
| YNL072W | 1 | 0 | 1 | 1 | no | no change | 0 | 0 | 0 | 0 | 0 | 0 |
| YNL089C | 0.917 | -0.1251 | 0.7943 | 0.8896 | no | down | 7.369 | 10.922 | 5.667 | 4.206 | 6.625 | 10.79 |
| YNL090W | 1.001 | 0.001161 | 0.9971 | 0.999 | no | up | 15.686 | 15.047 | 10.582 | 14.628 | 15.294 | 11.686 |
| YNL141W | 1.304 | 0.3834 | 0.2164 | 0.4178 | no | up | 959.05 | 420.754 | 516.636 | 943.875 | 725.938 | 897.122 |
| YNL142W | 0.695 | -0.5247 | 0.04433 | 0.1406 | no | down | 3927.095 | 5861.814 | 4311.97 | 3977.317 | 3459.771 | 2337.881 |
| YNL148C | 0.67 | -0.5782 | 0.04633 | 0.1447 | no | down | 19.078 | 18.691 | 11.967 | 10.132 | 10.448 | 12.49 |
| YNL169C | 1.536 | 0.6188 | 0.004525 | 0.02307 | no | up | 43.984 | 40.904 | 30.777 | 58.53 | 54.172 | 71.196 |
| YNL173C | 0.775 | -0.3679 | 0.244 | 0.4467 | no | down | 4.979 | 6.895 | 7.922 | 5.814 | 4.505 | 4.957 |
| YNL178W | 1.021 | 0.03045 | 0.8758 | 0.9378 | no | up | 5996.279 | 7746.569 | 5701.811 | 5783.087 | 6561.547 | 7809.906 |
| YNL209W | 1.139 | 0.1881 | 0.3707 | 0.581 | no | up | 2729.495 | 2813.008 | 2530.044 | 3266.167 | 2433.938 | 3725.106 |
| YNL219C | 0.904 | -0.1457 | 0.4538 | 0.6518 | no | down | 19.616 | 18.297 | 20.788 | 19.376 | 17.631 | 17.081 |
| YNL239W | 0.531 | -0.9138 | 0.004109 | 0.02129 | no | down | 58.949 | 70.579 | 68.359 | 49.687 | 20.357 | 31.445 |
| YNL289W | 0.592 | -0.756 | 0.08906 | 0.2321 | no | down | 3.219 | 5.639 | 4.005 | 2.841 | 1.363 | 2.85 |
| YNL307C | 0.831 | -0.2674 | 0.107 | 0.2631 | no | down | 79.203 | 75.701 | 72.067 | 65.184 | 66.362 | 60.12 |
| YNL321W | 0.751 | -0.4124 | 0.09967 | 0.2514 | no | down | 29.94 | 57.404 | 45.226 | 35.378 | 33.436 | 31.434 |
| YNR002C | 0.623 | -0.6826 | 0.1006 | 0.2528 | no | down | 13.406 | 54.141 | 25.18 | 23.021 | 17.177 | 14.272 |
| YNR013C | 0.886 | -0.1749 | 0.3093 | 0.5195 | no | down | 88.296 | 77.647 | 75.835 | 75.802 | 68.359 | 74.728 |
| YNR021W | 1.216 | 0.2816 | 0.3824 | 0.5921 | no | up | 21.823 | 11.254 | 18.039 | 21.311 | 15.36 | 27.912 |
| YNR030W | 0.631 | -0.6634 | 0.01434 | 0.0598 | no | down | 6.557 | 4.876 | 6.854 | 3.505 | 4.155 | 3.98 |
| YNR033W | 1.002 | 0.002874 | 0.9867 | 0.9937 | no | up | 22.908 | 22.927 | 20.947 | 23.778 | 22.903 | 21.469 |
| YNR057C | 0.503 | -0.9913 | 6.87E-06 | 8.52E-05 | no | down | 283.226 | 250.499 | 178.552 | 121.443 | 117.799 | 115.772 |
| YOL020W | 1.338 | 0.4196 | 0.03422 | 0.1157 | no | up | 141.654 | 192.147 | 137.331 | 227.37 | 223.029 | 195.834 |
| YOL030W | 1.225 | 0.293 | 0.236 | 0.4387 | no | up | 378.699 | 505.936 | 386.347 | 583.847 | 378.845 | 632.179 |
| YOL057W | 0.708 | -0.4985 | 0.1053 | 0.2603 | no | down | 8.828 | 4.433 | 6.171 | 4.655 | 4.221 | 4.988 |
| YOL059W | 0.669 | -0.5803 | 0.01152 | 0.05042 | no | down | 2134.015 | 2312.576 | 1341.579 | 1270.751 | 1272.153 | 1351.556 |
| YOL084W | 0.722 | -0.4704 | 0.1064 | 0.2619 | no | down | 14.217 | 34.477 | 25.18 | 16.918 | 18.124 | 18.333 |
| YOL085W-A | 0.577 | -0.7944 | 0.2257 | 0.4277 | no | down | 13.725 | 76.502 | 49.558 | 21.582 | 0 | 18.527 |
| YOL092W | 0.949 | -0.07499 | 0.7235 | 0.8505 | no | down | 419.719 | 592.535 | 383.252 | 469.302 | 446.21 | 424.771 |
| YOL120C | 0.927 | -0.1094 | 0.5841 | 0.7608 | no | down | 3981.265 | 4806.772 | 3831.49 | 3747.826 | 3344.206 | 4634.779 |
| YOL122C | 0.895 | -0.1595 | 0.4979 | 0.6886 | no | down | 28.143 | 22.866 | 25.269 | 16.675 | 27.417 | 26.121 |
| YOL126C | 0.537 | -0.8963 | 0.006116 | 0.02945 | no | down | 90.138 | 172.064 | 161.927 | 98.225 | 46.97 | 74.484 |
| YOR006C | 0.941 | -0.08721 | 0.7505 | 0.8638 | no | down | 108.742 | 57.207 | 70.841 | 66.035 | 72.901 | 88.786 |
| YOR038C | 1.278 | 0.3535 | 0.1391 | 0.3138 | no | up | 3.155 | 3.62 | 3.966 | 3.907 | 5.347 | 4.978 |
| YOR063W | 1.045 | 0.06313 | 0.7711 | 0.8753 | no | up | 4672.209 | 5244.887 | 4491.045 | 6256.502 | 4334.483 | 4677.523 |
| YOR074C | 1.548 | 0.6306 | 0.05515 | 0.1646 | no | up | 4.806 | 4.457 | 6.567 | 6.3 | 9.265 | 10.373 |
| YOR084W | 1.479 | 0.5643 | 0.05999 | 0.1755 | no | up | 52.337 | 121.433 | 82.284 | 149.474 | 135.922 | 106.203 |
| YOR087W | 0.698 | -0.5197 | 0.1227 | 0.2893 | no | down | 5.435 | 2.857 | 4.055 | 2.954 | 2.271 | 3.4 |
| YOR094W | 1.013 | 0.01836 | 0.9431 | 0.9732 | no | up | 28.918 | 21.917 | 27.484 | 23.096 | 29.272 | 27.872 |
| YOR095C | 1.514 | 0.5987 | 0.01801 | 0.07051 | no | up | 74.945 | 43.232 | 53.435 | 78.494 | 90.116 | 101.134 |
| YOR100C | 0.544 | -0.8783 | 0.002598 | 0.01474 | no | down | 56.195 | 98.259 | 80.513 | 53.828 | 39.597 | 31.16 |
| YOR101W | 1.182 | 0.2418 | 0.4399 | 0.6385 | no | up | 32.63 | 14.874 | 18.929 | 26.844 | 25.382 | 28.971 |
| YOR133W | 1.077 | 0.1076 | 0.5314 | 0.7179 | no | up | 1746.908 | 2143.627 | 1644.951 | 1989.537 | 1907.35 | 2209.334 |
| YOR142W | 0.679 | -0.5576 | 0.002525 | 0.01441 | no | down | 106.152 | 112.986 | 104.387 | 81.896 | 73.611 | 65.851 |
| YOR157C | 0.503 | -0.9901 | 0.000258 | 0.002089 | no | down | 170.089 | 340.053 | 220.455 | 123.966 | 133.925 | 101.958 |
| YOR180C | 1 | 0 | 1 | 1 | no | no change | 0 | 0 | 0 | 0 | 0 | 0 |
| YOR192C-A | 0.661 | -0.5981 | 0.04583 | 0.1439 | no | down | 11.655 | 23.087 | 13.5 | 11.011 | 11.565 | 8.897 |
| YOR217W | 1 | 0 | 1 | 1 | no | no change | 0 | 0 | 0 | 0 | 0 | 0 |
| YOR228C | 1 | 0 | 1 | 1 | no | no change | 0 | 0 | 0 | 0 | 0 | 0 |
| YOR273C | 1.487 | 0.5728 | 0.3621 | 0.5726 | no | up | 0 | 7.055 | 3.926 | 15.656 | 9.398 | 9.477 |
| YOR321W | 0.586 | -0.7713 | 7.91E-05 | 0.000765 | no | down | 14.929 | 19.763 | 18.998 | 10.599 | 10.694 | 10.434 |
| YOR356W | 0.961 | -0.0579 | 0.7164 | 0.8449 | no | down | 68.88 | 85.872 | 67.963 | 67.802 | 76.649 | 74.962 |
| YOR374W | 0.509 | -0.9731 | 4.68E-05 | 0.000475 | no | down | 81.638 | 90.785 | 93.034 | 37.069 | 36.929 | 61.413 |
| YPL003W | 0.502 | -0.9953 | 4.07E-05 | 0.000423 | no | down | 9.721 | 11.87 | 10.483 | 5.44 | 5.934 | 4.611 |
| YPL028W | 0.531 | -0.9125 | 0.000126 | 0.001121 | no | down | 176.974 | 305.724 | 199.657 | 127.537 | 121.291 | 110.733 |
| YPL036W | 0.695 | -0.5249 | 0.09433 | 0.2415 | no | down | 5.162 | 2.82 | 3.55 | 2.542 | 2.29 | 3.247 |
| YPL054W | 1 | 0 | 1 | 1 | no | no change | 0 | 0 | 0 | 0.869 | 0 | 0 |
| YPL061W | 1.039 | 0.0547 | 0.7351 | 0.8576 | no | up | 41.576 | 30.968 | 32.577 | 79.149 | 96.012 | 137.709 |
| YPL062W | 1 | 0 | 1 | 1 | no | no change | 0 | 0 | 0 | 0 | 0 | 0 |
| YPL104W | 0.749 | -0.4163 | 0.2379 | 0.4411 | no | down | 2.772 | 1.588 | 2.413 | 1.767 | 1.505 | 1.832 |
| YPL116W | 1.388 | 0.4734 | 0.2265 | 0.4283 | no | up | 2.234 | 1.675 | 3.125 | 4.262 | 1.95 | 4.133 |
| YPL131W | 1.074 | 0.1034 | 0.6958 | 0.8336 | no | up | 3713.277 | 4176.275 | 3775.019 | 4666.381 | 2725.174 | 5338.724 |
| YPL231W | 0.657 | -0.6069 | 0.2797 | 0.4888 | no | down | 85.742 | 30.118 | 41.419 | 0 | 0.521 | 21.764 |
| YPL270W | 1 | 0 | 1 | 1 | no | no change | 0.456 | 0 | 0 | 0 | 0 | 0 |
| YPR002W | 0.682 | -0.5516 | 0.1323 | 0.3037 | no | down | 6.913 | 2.795 | 4.806 | 3 | 3.341 | 3.532 |
| YPR006C | 0.601 | -0.7341 | 0.009858 | 0.0443 | no | down | 19.343 | 29.81 | 26.099 | 17.273 | 17.423 | 10.057 |
| YPR083W | 0.582 | -0.7818 | 0.01922 | 0.07417 | no | down | 3.256 | 2.34 | 3.59 | 1.486 | 1.864 | 1.944 |
| YPR155C | 0.76 | -0.3962 | 0.2306 | 0.4339 | no | down | 2.362 | 2.512 | 3.501 | 1.841 | 1.94 | 2.636 |
| YPR158C-C | 0.577 | -0.7941 | 9.64E-05 | 0.000902 | no | down | 96.667 | 115.362 | 77.685 | 48.846 | 57.409 | 62.044 |
| YPR164W | 0.782 | -0.3543 | 0.311 | 0.522 | no | down | 1.35 | 1.084 | 0.742 | 0.645 | 0.965 | 0.906 |
| YPR171W | 0.704 | -0.5069 | 0.02145 | 0.08009 | no | down | 24.869 | 32.925 | 21.352 | 19.367 | 17.65 | 19.087 |
| YPR184W | 0.779 | -0.3598 | 0.102 | 0.2553 | no | down | 5.499 | 5.689 | 7.457 | 5.206 | 4.647 | 4.927 |
| YPR192W | 0.636 | -0.6524 | 0.01638 | 0.06574 | no | down | 29.009 | 51.814 | 31.381 | 23.517 | 21.057 | 26.1 |
| YPR193C | 1 | 0 | 1 | 1 | no | no change | 0 | 0 | 0 | 0 | 0 | 0 |
| ENSRNA049651861 | |  |  |  |  |  |  |  |  |  |  |  |
| ENSRNA049651872 | |  |  |  |  |  |  |  |  |  |  |  |
| ENSRNA049652191 | |  |  |  |  |  |  |  |  |  |  |  |
| ENSRNA049652654 | |  |  |  |  |  |  |  |  |  |  |  |
| ENSRNA049652728 | |  |  |  |  |  |  |  |  |  |  |  |
