# Supplementary material for: Hydrogen sulfide treatment at the late growth stage of Saccharomyces cerevisiae extends chronological lifespan
Source: Aging (Albany NY). 2021 Mar 19;13(7):9859–73. doi: 10.18632/aging.202738 (PMC8064171; doi:10.18632/aging.202738)
Supplement: Supplementary Table 5 [file aging-13-202738-s006.doc]

**Supplementary Table 5. Specific DEGs induced by the late NaHS treatment and the expression of these genes in the early NaHS treatment.**

Specific DEGs late treatment

| Gene_id | FC(NaHS/Control) | Log2FC(NaHS/Control) | Pvalue | Padjust | Significant | Regulate | Control1_Fpkm | Control2_Fpkm | Control3_Fpkm | NaHS1_Fpkm | NaHS2_Fpkm | NaHS3_Fpkm |
| --- | --- | --- | --- | --- | --- | --- | --- | --- | --- | --- | --- | --- |
| YDL213C | 2.024 | 1.017 | 0.000356 | 0.002802 | yes | up | 49.072 | 23.949 | 42.536 | 77.419 | 88.479 | 81.284 |
| YDR353W | 2.201 | 1.138 | 3.66E-12 | 1.11E-10 | yes | up | 340.78 | 382.14 | 311.59 | 694.4 | 875.356 | 781.472 |
| YPL014W | 5.909 | 2.563 | 9.46E-31 | 9.37E-29 | yes | up | 14.901 | 24.158 | 22.895 | 127.733 | 131.531 | 133.698 |
| YKL096W-A | 4.101 | 2.036 | 8.68E-12 | 2.57E-10 | yes | up | 992.291 | 1547.94 | 1069.539 | 3379.441 | 5019.082 | 7151.962 |
| YFL017C | 3.176 | 1.667 | 0.001614 | 0.01004 | yes | up | 8.198 | 8.816 | 0.742 | 16.105 | 33.616 | 26.579 |
| YGL062W | 2.723 | 1.445 | 1.73E-09 | 4.05E-08 | yes | up | 84.666 | 72.217 | 107.414 | 295.199 | 259.447 | 208.894 |
| YGL079W | 2.147 | 1.102 | 8.53E-05 | 0.000813 | yes | up | 11.764 | 15.859 | 11.769 | 33.639 | 24.114 | 30.366 |
| YMR244W | 2.216 | 1.148 | 0.000347 | 0.002745 | yes | up | 671.556 | 415.484 | 242.4 | 1091.396 | 1102.028 | 978.161 |
| YMR233W | 2.042 | 1.03 | 1.06E-05 | 0.000126 | yes | up | 20.911 | 15.884 | 16.951 | 37.677 | 36.162 | 40.24 |
| YDR246W-A | 2.483 | 1.312 | 0.03421 | 0.1157 | yes | up | 0 | 0 | 4.421 | 34.863 | 20.811 | 94.242 |
| YHL046W-A | 2.641 | 1.401 | 0.002252 | 0.01311 | yes | up | 29.848 | 10.343 | 13.658 | 32.76 | 63.182 | 65.872 |
| YJR003C | 2.098 | 1.069 | 0.000383 | 0.002986 | yes | up | 5.116 | 2.931 | 4.352 | 7.244 | 10.032 | 10.851 |
| YOR388C | 2.296 | 1.199 | 5.07E-05 | 0.000509 | yes | up | 4.742 | 6.329 | 6.389 | 10.44 | 19.061 | 13.661 |
| YBL043W | 2.227 | 1.155 | 0.000433 | 0.003322 | yes | up | 94.843 | 65.445 | 131.466 | 323.277 | 196.766 | 173.866 |
| YOR267C | 2.755 | 1.462 | 0.02513 | 0.09051 | yes | up | 13.442 | 0.48 | 22.114 | 88.318 | 66.948 | 94.15 |
| YDR096W | 2.192 | 1.132 | 7.12E-05 | 0.000697 | yes | up | 1.833 | 2.216 | 3.214 | 5.823 | 5.129 | 6.026 |
| YKL217W | 2.218 | 1.149 | 0.000173 | 0.001472 | yes | up | 100.634 | 170.328 | 149.07 | 407.716 | 383.577 | 200.567 |
| YGR103W | 2.299 | 1.201 | 0.001654 | 0.01024 | yes | up | 8.408 | 3.46 | 7.16 | 10.515 | 25.458 | 13.641 |
| YLR089C | 2.354 | 1.235 | 1.72E-05 | 0.000196 | yes | up | 93.995 | 159.37 | 131.802 | 428.111 | 238.067 | 292.763 |
| YMR104C | 409.012 | 8.676 | 6.4E-73 | 9.03E-70 | yes | up | 0 | 0 | 0 | 29.685 | 48.92 | 50.979 |
| YCL026C-B | 20.563 | 4.362 | 1.27E-13 | 4.56E-12 | yes | up | 0 | 0.135 | 0 | 11.842 | 17.083 | 5.833 |
| YLR455W | 2.205 | 1.141 | 0.000135 | 0.00119 | yes | up | 10.086 | 5.8 | 9.039 | 15.254 | 22.553 | 21.469 |
| YHR126C | 3.866 | 1.951 | 0.001172 | 0.007698 | yes | up | 161.298 | 44.524 | 25.318 | 255.363 | 543.49 | 825.549 |
| YOR092W | 3.292 | 1.719 | 2.57E-12 | 8.11E-11 | yes | up | 16.525 | 23.42 | 24.369 | 81.887 | 58.279 | 85.396 |
| YGR158C | 8.926 | 3.158 | 1.27E-06 | 1.82E-05 | yes | up | 0 | 0 | 0 | 3.15 | 0.558 | 13.61 |
| YDR249C | 2.331 | 1.221 | 0.000183 | 0.001556 | yes | up | 10.579 | 4.962 | 9.385 | 15.45 | 23.698 | 24.787 |
| YGR292W | 2.478 | 1.309 | 9.31E-05 | 0.000879 | yes | up | 2.718 | 1.798 | 1.741 | 7.029 | 4.685 | 5.212 |
| YGR181W | 2.11 | 1.077 | 0.0179 | 0.0701 | yes | up | 12.476 | 19.541 | 31.766 | 44.967 | 66.977 | 35.231 |
| YIL162W | 4.19 | 2.067 | 5.25E-11 | 1.42E-09 | yes | up | 4.496 | 3.435 | 5.034 | 27.199 | 19.505 | 14.2 |
| YGL176C | 6.765 | 2.758 | 2.42E-05 | 0.000268 | yes | up | 0 | 0 | 0 | 0.682 | 1.174 | 0.285 |
| YGL174W | 22.331 | 4.481 | 1.98E-14 | 7.59E-13 | yes | up | 0 | 0.062 | 0.158 | 10.01 | 3.748 | 8.032 |
| YPR130C | 2.27 | 1.183 | 0.04532 | 0.1428 | yes | up | 2.544 | 4.901 | 1.019 | 6.552 | 13.316 | 5.823 |
| YKR061W | 2.028 | 1.02 | 4.34E-06 | 5.61E-05 | yes | up | 62.241 | 47.861 | 55.897 | 89.589 | 123.212 | 141.17 |
| YDR360W | 2.721 | 1.444 | 0.001668 | 0.01031 | yes | up | 6.876 | 3.854 | 3.303 | 16.983 | 12.966 | 14.027 |
| YOR105W | 2.025 | 1.018 | 0.03799 | 0.125 | yes | up | 18.403 | 10.442 | 8.357 | 17.31 | 42.815 | 26.416 |
| YJL213W | 69.118 | 6.111 | 5.56E-29 | 5.32E-27 | yes | up | 0 | 0 | 0 | 17.88 | 16.685 | 9.162 |
| YJL012C | 2.107 | 1.075 | 1.78E-07 | 3.02E-06 | yes | up | 38.512 | 31.633 | 34.169 | 60.558 | 91.678 | 78.871 |
| YNL053W | 2.039 | 1.028 | 3.16E-05 | 0.00034 | yes | up | 24.878 | 19.27 | 24.497 | 56.941 | 37.856 | 51.783 |
| YGL255W | 2.594 | 1.375 | 4.6E-06 | 5.91E-05 | yes | up | 1045.276 | 716.086 | 630.498 | 2800.576 | 2417.546 | 1444.658 |
| YNL125C | 4.438 | 2.15 | 7.3E-13 | 2.47E-11 | yes | up | 59.87 | 105.598 | 115.158 | 584.809 | 456.26 | 336.942 |
| YMR058W | 4.17 | 2.06 | 4.59E-28 | 4.25E-26 | yes | up | 469.74 | 551.261 | 447.378 | 2660.571 | 2184.192 | 2236.065 |
| YMR166C | 26.983 | 4.754 | 4.74E-16 | 2.06E-14 | yes | up | 0 | 0 | 0 | 4.028 | 4.429 | 3.471 |
| YLR142W | 2.018 | 1.013 | 4.24E-05 | 0.000438 | yes | up | 16.397 | 14 | 18.722 | 40.93 | 35.112 | 27.81 |
| YDL055C | 2.277 | 1.187 | 4.08E-07 | 6.62E-06 | yes | up | 931.682 | 837.21 | 731.029 | 1360.732 | 2379.813 | 2276.071 |
| YNR004W | 2.266 | 1.18 | 0.000836 | 0.005793 | yes | up | 9.092 | 19.676 | 18.642 | 36.032 | 37.071 | 41.064 |
| YGL008C | 2.738 | 1.453 | 3.98E-06 | 5.2E-05 | yes | up | 1860.437 | 749.011 | 1131.618 | 2949.339 | 4073.494 | 4363.943 |
| YPL005W | 2.826 | 1.499 | 2.24E-14 | 8.55E-13 | yes | up | 17.263 | 16.438 | 19.246 | 51.071 | 45.91 | 60.192 |
| YAL034W-A | 2.091 | 1.064 | 0.01475 | 0.06097 | yes | up | 1.696 | 1.231 | 1.948 | 3.187 | 3.322 | 5.141 |
| YNL144C | 3.16 | 1.66 | 5.71E-09 | 1.25E-07 | yes | up | 2.116 | 2.943 | 3.422 | 11.459 | 9.511 | 7.981 |
| YGR042W | 6.797 | 2.765 | 2.24E-05 | 0.000251 | yes | up | 0 | 0 | 0 | 1.72 | 1.211 | 1.802 |
| YLR126C | 2.014 | 1.01 | 0.00021 | 0.001765 | yes | up | 14.901 | 10.072 | 10.701 | 25.292 | 28.392 | 21.998 |
| YGR169C | 134.271 | 7.069 | 9.74E-43 | 2.04E-40 | yes | up | 0 | 0 | 0 | 19.909 | 17.3 | 29.256 |
| YPL144W | 2.521 | 1.334 | 0.005073 | 0.02531 | yes | up | 5.982 | 33.516 | 12.362 | 40.836 | 68.87 | 44.861 |
| YLR069C | 2.333 | 1.222 | 5.14E-05 | 0.000515 | yes | up | 4.979 | 2.82 | 3.234 | 7.365 | 11.849 | 8.846 |
| YDR309C | 2.745 | 1.457 | 4.18E-05 | 0.000433 | yes | up | 18.622 | 15.539 | 14.152 | 53.716 | 23.405 | 70.117 |
| YER095W | 2.041 | 1.029 | 2.77E-05 | 0.000303 | yes | up | 223.055 | 435.16 | 303.708 | 659.032 | 713.587 | 677.386 |
| YBR054W | 3.165 | 1.662 | 2.81E-08 | 5.58E-07 | yes | up | 685.755 | 303.729 | 492.347 | 1345.432 | 2070.358 | 1745.015 |
| YIL002W-A | 2.101 | 1.071 | 0.003888 | 0.02041 | yes | up | 255.822 | 227.277 | 182.359 | 529.28 | 474.592 | 384.399 |
| YPL111W | 2.285 | 1.192 | 0.000115 | 0.00104 | yes | up | 262.47 | 495.827 | 528.88 | 1310.26 | 1075.273 | 744.765 |
| YDR264C | 2.173 | 1.12 | 0.007668 | 0.03576 | yes | up | 29.347 | 5.935 | 26.287 | 52.669 | 53.387 | 47.233 |
| YDR055W | 5.256 | 2.394 | 9.36E-25 | 7.44E-23 | yes | up | 96.54 | 122.726 | 147.596 | 521.831 | 790.605 | 782.755 |
| YAL056C-A | 2.904 | 1.538 | 0.0191 | 0.07382 | yes | up | 0.538 | 0 | 1.305 | 2.028 | 3.767 | 13.885 |
| YNL024C | 110.814 | 6.792 | 2.5E-37 | 3.82E-35 | yes | up | 0 | 0 | 0 | 23.199 | 42.077 | 60.446 |
| YKR093W | 2.196 | 1.135 | 0.000723 | 0.00518 | yes | up | 19.16 | 21.006 | 19.651 | 71.447 | 42.948 | 26.375 |
| YDR342C | 2.88 | 1.526 | 0.000198 | 0.00167 | yes | up | 213.872 | 1089.409 | 695.564 | 2410.32 | 2550.345 | 1670.338 |
| YGR023W | 2.774 | 1.472 | 3.01E-09 | 6.8E-08 | yes | up | 25.279 | 37.26 | 45.385 | 99.74 | 96.145 | 122.49 |
| YMR290W-A | 2.439 | 1.286 | 0.001465 | 0.009288 | yes | up | 33.423 | 27.84 | 22.025 | 81.139 | 39.039 | 98.222 |
| YIL131C | 19.041 | 4.251 | 6.37E-12 | 1.9E-10 | yes | up | 0 | 0 | 0 | 0.963 | 2.811 | 5.232 |
| YDR068W | 2.004 | 1.003 | 0.005198 | 0.0258 | yes | up | 1.915 | 2.413 | 2.848 | 5.094 | 5.338 | 5.12 |
| YML053C | 18.252 | 4.19 | 9.37E-12 | 2.76E-10 | yes | up | 0 | 0 | 0 | 3.44 | 11.764 | 7.899 |
| YDR270W | 2.511 | 1.328 | 1.01E-06 | 1.49E-05 | yes | up | 6.995 | 5.258 | 7.704 | 22.629 | 15.568 | 15.218 |
| YHR030C | 2.333 | 1.222 | 1.72E-06 | 2.41E-05 | yes | up | 119.849 | 98.333 | 125.285 | 210.162 | 251.014 | 393.205 |
| YGR152C | 18.038 | 4.173 | 2.12E-11 | 5.96E-10 | yes | up | 0 | 0 | 0 | 2.196 | 4.514 | 13.264 |
| YGR273C | 2.799 | 1.485 | 0.0227 | 0.08364 | yes | up | 0 | 0.271 | 0 | 0.785 | 1.779 | 1.863 |
| YER137W-A | 2.643 | 1.402 | 0.003269 | 0.01779 | yes | up | 11.7 | 7.807 | 12.432 | 28.872 | 37.705 | 31.282 |
| YDR475C | 2.102 | 1.072 | 9.35E-08 | 1.66E-06 | yes | up | 12.786 | 13.495 | 17.752 | 30.181 | 32.887 | 33.735 |
| YGL179C | 4.187 | 2.066 | 1.16E-20 | 6.89E-19 | yes | up | 7.597 | 9.666 | 11.502 | 44.771 | 43.686 | 39.873 |
| YMR307W | 3.123 | 1.643 | 4.91E-25 | 3.96E-23 | yes | up | 1012.281 | 857.552 | 895.982 | 2784.864 | 3087.958 | 3140.068 |
| YDL182W | 2.815 | 1.493 | 2.3E-18 | 1.18E-16 | yes | up | 107.456 | 113.7 | 105.08 | 271.832 | 313.42 | 372.143 |
| YPR079W | 2.104 | 1.073 | 6.79E-09 | 1.45E-07 | yes | up | 18.868 | 22.028 | 23.33 | 42.556 | 48.011 | 49.584 |
| YJR148W | 5.449 | 2.446 | 6.92E-30 | 6.74E-28 | yes | up | 256.798 | 294.162 | 262.625 | 1856.336 | 1624.423 | 1239.317 |
| YHR159W | 2.034 | 1.024 | 0.000423 | 0.003247 | yes | up | 2.572 | 2.401 | 3.62 | 5.711 | 6.521 | 6.444 |
| YIL120W | 77.332 | 6.273 | 2.67E-31 | 2.79E-29 | yes | up | 0 | 0 | 0 | 10.104 | 7.742 | 5.914 |
| YPR156C | 3.948 | 1.981 | 4.87E-16 | 2.1E-14 | yes | up | 65.25 | 63.007 | 99.621 | 348.14 | 278.659 | 341.309 |
| YAR066W | 2.516 | 1.331 | 0.004008 | 0.02094 | yes | up | 4.998 | 1.416 | 4.816 | 7.664 | 13.259 | 13.101 |
| YPR027C | 2.118 | 1.083 | 0.000636 | 0.004638 | yes | up | 5.162 | 8.422 | 7.012 | 16.609 | 18.095 | 11.594 |
| YCR026C | 618.23 | 9.272 | 1.47E-98 | 4.16E-95 | yes | up | 0 | 0 | 0.02 | 19.357 | 26.679 | 16.867 |
| YOR383C | 3.883 | 1.957 | 2.55E-18 | 1.3E-16 | yes | up | 452.011 | 588.004 | 384.201 | 2668.366 | 2498.634 | 1969.555 |
| YFL051C | 2.415 | 1.272 | 0.01216 | 0.05233 | yes | up | 2.535 | 4.814 | 3.511 | 14.301 | 12.303 | 4.968 |
| YBL005W-B | 2.104 | 1.073 | 0.007464 | 0.03513 | yes | up | 0.264 | 0.197 | 0.336 | 0.598 | 0.502 | 0.784 |
| YDR222W | 4.24 | 2.084 | 6.8E-06 | 8.46E-05 | yes | up | 33.131 | 4.581 | 13.599 | 69.923 | 101.634 | 117.879 |
| YNL283C | 4.047 | 2.017 | 6.23E-10 | 1.54E-08 | yes | up | 3.301 | 7.289 | 6.705 | 24.302 | 20.007 | 34.162 |
| YOL032W | 2.317 | 1.212 | 9.43E-05 | 0.000888 | yes | up | 6.776 | 9.974 | 7.487 | 15.871 | 17.736 | 26.375 |
| YHR094C | 2.634 | 1.397 | 2.31E-06 | 3.15E-05 | yes | up | 532.555 | 629.684 | 269.845 | 1448.714 | 1456.721 | 1148.169 |
| YBL070C | 2.364 | 1.241 | 0.000272 | 0.0022 | yes | up | 61.84 | 28.037 | 39.184 | 102.927 | 103.83 | 104.401 |
| YML123C | 2.583 | 1.369 | 0.03021 | 0.1052 | yes | up | 7.569 | 0 | 1.79 | 70.606 | 114.524 | 85.131 |
| YBL069W | 2.597 | 1.377 | 1.54E-09 | 3.64E-08 | yes | up | 70.841 | 47.984 | 63.908 | 166.821 | 191.429 | 143.256 |
| YLR110C | 2.497 | 1.32 | 1.26E-05 | 0.000147 | yes | up | 10188.72 | 23790.78 | 12900.01 | 40995.53 | 38376.83 | 42694.59 |
| YFL020C | 8.369 | 3.065 | 2.15E-06 | 2.96E-05 | yes | up | 0 | 0 | 0 | 6.767 | 10.212 | 17.723 |
| YOR104W | 2.272 | 1.184 | 0.000598 | 0.004383 | yes | up | 18.312 | 9.063 | 9.455 | 20.311 | 36.522 | 35.089 |
| YOR178C | 200.159 | 7.645 | 1.32E-53 | 5.34E-51 | yes | up | 0 | 0 | 0 | 15.693 | 12.871 | 14.343 |
| YER188W | 2.152 | 1.106 | 0.000811 | 0.00566 | yes | up | 13.178 | 24.035 | 18.435 | 56.782 | 40.26 | 29.714 |
| YKR013W | 2.526 | 1.337 | 8.22E-07 | 1.23E-05 | yes | up | 100.926 | 156.033 | 113.565 | 296.134 | 245.941 | 452.266 |
| YLL028W | 2.338 | 1.225 | 0.04334 | 0.1385 | yes | up | 0.684 | 0 | 0 | 27.891 | 14.802 | 23.311 |
| YHL008C | 99.457 | 6.636 | 1.05E-35 | 1.45E-33 | yes | up | 0 | 0 | 0 | 12.422 | 6.398 | 10.515 |
| YDR247W | 94.878 | 6.568 | 2.03E-33 | 2.39E-31 | yes | up | 0 | 0 | 0 | 22.329 | 7.619 | 19.066 |
| YMR215W | 3.055 | 1.611 | 4.99E-16 | 2.14E-14 | yes | up | 32.985 | 34.908 | 33.744 | 93.346 | 98.076 | 135.561 |
| YLR113W | 2.275 | 1.186 | 1.63E-08 | 3.39E-07 | yes | up | 153.71 | 112.185 | 118.293 | 252.924 | 350.945 | 314.099 |
| YLR176C | 2.445 | 1.29 | 4.55E-05 | 0.000466 | yes | up | 3.693 | 2.413 | 2.73 | 4.888 | 9.36 | 9.569 |
| YKL167C | 19.253 | 4.267 | 7.33E-16 | 3.11E-14 | yes | up | 0 | 0.382 | 2.69 | 75.466 | 40.307 | 39.283 |
| YOR389W | 2.533 | 1.341 | 9.23E-11 | 2.41E-09 | yes | up | 64.712 | 47.59 | 54.157 | 161.522 | 139.821 | 140.569 |
| YER145C | 3.618 | 1.855 | 1.38E-12 | 4.51E-11 | yes | up | 804.045 | 1142.269 | 622.774 | 3887.999 | 3333.209 | 2724.703 |
| YGR022C | 2.398 | 1.262 | 0.002515 | 0.01438 | yes | up | 11.938 | 25.685 | 22.292 | 51.781 | 41.31 | 63.622 |
| YLR057W | 2.065 | 1.046 | 0.001158 | 0.007648 | yes | up | 1.696 | 1.478 | 2.71 | 3.337 | 4.741 | 5.151 |
| YGR225W | 2.259 | 1.176 | 0.03242 | 0.1113 | yes | up | 0.228 | 0.172 | 0.504 | 0.879 | 0.587 | 1.283 |
| YPR137C-B | 2.624 | 1.392 | 0.03387 | 0.1151 | yes | up | 0 | 0 | 0.049 | 0.178 | 0.066 | 0.112 |
| YHL047C | 2.478 | 1.309 | 2.42E-07 | 4.05E-06 | yes | up | 47.859 | 32.408 | 35.96 | 72.148 | 116.635 | 120.006 |
| YER045C | 2.495 | 1.319 | 7.15E-06 | 8.8E-05 | yes | up | 13.853 | 10.392 | 17.327 | 38.453 | 26.282 | 47.121 |
| YOR071C | 2.045 | 1.032 | 3.5E-08 | 6.8E-07 | yes | up | 28.581 | 31.928 | 26.742 | 62.708 | 53.67 | 68.498 |
| YMR180C | 2.011 | 1.008 | 0.001465 | 0.009288 | yes | up | 5.946 | 10.971 | 5.4 | 16.273 | 14.868 | 16.033 |
| YLR214W | 3.002 | 1.586 | 1.47E-12 | 4.77E-11 | yes | up | 15.458 | 11.045 | 12.55 | 36.387 | 49.856 | 38.723 |
| YLR257W | 2.717 | 1.442 | 2.29E-09 | 5.3E-08 | yes | up | 122.603 | 78.792 | 102.558 | 237.969 | 362.377 | 278.481 |
| YPR175W | 2.167 | 1.116 | 3.69E-05 | 0.000388 | yes | up | 10.15 | 7.868 | 8.772 | 13.628 | 22.117 | 26.538 |
| YDR423C | 3.444 | 1.784 | 2.76E-23 | 1.92E-21 | yes | up | 15.029 | 13.963 | 15.685 | 53.323 | 55.904 | 51.987 |
| YDR422C | 2.317 | 1.212 | 0.04491 | 0.142 | yes | up | 0 | 0 | 0 | 0.093 | 0 | 0.794 |
| YDR420W | 2.215 | 1.147 | 0.008825 | 0.04036 | yes | up | 1.751 | 0.616 | 2.383 | 4.038 | 2.243 | 5.935 |
| YKR010C | 2.227 | 1.155 | 0.000192 | 0.001621 | yes | up | 1.942 | 1.391 | 2.571 | 3.916 | 5.423 | 4.978 |
| YCL059C | 2.139 | 1.097 | 0.002424 | 0.01394 | yes | up | 46.273 | 13.865 | 31.381 | 63.268 | 71.33 | 80.724 |
| YML046W | 2.053 | 1.038 | 0.01607 | 0.0651 | yes | up | 4.697 | 1.551 | 2.097 | 3.486 | 8.877 | 7.482 |
| YDL174C | 5.315 | 2.41 | 3.21E-22 | 2.01E-20 | yes | up | 125.458 | 226.181 | 171.075 | 1054.214 | 833.155 | 1112.683 |
| YEL059C-A | 3.487 | 1.802 | 0.000438 | 0.003338 | yes | up | 45.516 | 56.653 | 15.171 | 174.607 | 144.374 | 190.062 |
| YMR011W | 3.027 | 1.598 | 3.15E-06 | 4.18E-05 | yes | up | 258.138 | 354.977 | 241.313 | 1450.144 | 874.882 | 517.721 |
| YMR103C | 2.809 | 1.49 | 0.000168 | 0.001439 | yes | up | 16.744 | 41.68 | 37.275 | 60.147 | 124.651 | 113.441 |
| YKL131W | 2.003 | 1.002 | 0.000806 | 0.005649 | yes | up | 10.597 | 14.234 | 14.963 | 30.181 | 27.029 | 25.866 |
| YER053C | 2.152 | 1.106 | 0.000486 | 0.003651 | yes | up | 122.357 | 142.771 | 199.528 | 509.147 | 308.991 | 242.263 |
| YJR160C | 3.56 | 1.832 | 0.000257 | 0.002088 | yes | up | 1.058 | 0.394 | 1.78 | 6.403 | 2.11 | 7.136 |
| YLL029W | 2.118 | 1.083 | 3.02E-09 | 6.81E-08 | yes | up | 59.067 | 54.141 | 69.427 | 124.957 | 148.831 | 129.728 |
| YPR119W | 2.592 | 1.374 | 0.005681 | 0.02773 | yes | up | 0.328 | 0.443 | 0.316 | 1.131 | 1.23 | 1.171 |
| YDL062W | 2.882 | 1.527 | 0.00299 | 0.01649 | yes | up | 5.609 | 1.194 | 6.438 | 16.123 | 11.215 | 21.357 |
| YDR306C | 16.359 | 4.032 | 1.09E-10 | 2.83E-09 | yes | up | 0 | 0 | 0 | 0.738 | 4.117 | 2.769 |
| YDL179W | 2.051 | 1.036 | 0.00137 | 0.008764 | yes | up | 2.836 | 3.62 | 3.966 | 6.982 | 7.117 | 8.754 |
| YPR098C | 0.197 | -2.346 | 6.77E-24 | 5.03E-22 | yes | down | 273.979 | 280.113 | 214.056 | 44.612 | 59.869 | 38.682 |
| YIL014C-A | 0.499 | -1.003 | 0.004659 | 0.02362 | yes | down | 88.87 | 132.514 | 73.314 | 35.378 | 54.986 | 44.994 |
| YLL027W | 0.45 | -1.151 | 1.3E-08 | 2.74E-07 | yes | down | 305.961 | 404.402 | 377.803 | 180.645 | 161.163 | 143.572 |
| YBL095W | 0.481 | -1.056 | 0.0007 | 0.005038 | yes | down | 110.657 | 239.344 | 157.219 | 96.552 | 80.387 | 57.26 |
| YPL171C | 0.453 | -1.143 | 0.000228 | 0.001892 | yes | down | 64.767 | 161.561 | 94.231 | 49.444 | 50.992 | 39.283 |
| YHR051W | 0.016 | -5.941 | 1.46E-25 | 1.23E-23 | yes | down | 86.125 | 23.432 | 89.652 | 0 | 0 | 0 |
| YKL007W | 0.462 | -1.113 | 6.96E-07 | 1.07E-05 | yes | down | 33.724 | 34.046 | 36.662 | 17.095 | 17.158 | 13.549 |
| YDL003W | 0.388 | -1.366 | 2.24E-05 | 0.000251 | yes | down | 3.593 | 4.544 | 3.452 | 1.626 | 1.221 | 1.415 |
| YMR315W | 0.004 | -8.024 | 6.29E-59 | 3.56E-56 | yes | down | 37.81 | 69.914 | 49.865 | 0 | 0 | 0 |
| YER061C | 0.481 | -1.056 | 1.25E-06 | 1.79E-05 | yes | down | 56.395 | 61.96 | 55.363 | 22.498 | 25.411 | 35.862 |
| YDR072C | 0.469 | -1.092 | 1.31E-06 | 1.88E-05 | yes | down | 255.257 | 197.713 | 155.657 | 90.402 | 104.275 | 90.934 |
| YER011W | 0.424 | -1.239 | 1.73E-08 | 3.56E-07 | yes | down | 57.982 | 111.705 | 58.044 | 21.348 | 20.575 | 19.748 |
| YMR008C | 0.379 | -1.398 | 4.05E-12 | 1.22E-10 | yes | down | 428.592 | 523.36 | 486.275 | 211.91 | 171.668 | 158.628 |
| YOR184W | 0.179 | -2.481 | 1.88E-48 | 6.63E-46 | yes | down | 187.909 | 202.256 | 193.545 | 31.059 | 32.679 | 40.006 |
| YIL034C | 0.474 | -1.076 | 3.04E-07 | 5.06E-06 | yes | down | 115.919 | 170.759 | 128.806 | 69.147 | 62.387 | 63.917 |
| YDL243C | 0.193 | -2.377 | 1.62E-17 | 7.81E-16 | yes | down | 20.245 | 24.836 | 16.358 | 3.776 | 4.079 | 3.207 |
| YDR379C-A | 0.453 | -1.144 | 0.006057 | 0.02919 | yes | down | 168.457 | 200.151 | 134.176 | 60.118 | 83.198 | 48.332 |
| YBR256C | 0.4 | -1.321 | 1.48E-16 | 6.72E-15 | yes | down | 1004.411 | 1143.057 | 964.716 | 403.36 | 454.245 | 386.944 |
| YDR223W | 0.015 | -6.083 | 2.06E-27 | 1.84E-25 | yes | down | 4.304 | 15.687 | 10.711 | 0 | 0 | 0 |
| YNL104C | 0.214 | -2.225 | 1.33E-43 | 3.28E-41 | yes | down | 1312.251 | 1394.998 | 1382.741 | 307.369 | 265.929 | 299.197 |
| YOR173W | 0.238 | -2.068 | 2.61E-19 | 1.39E-17 | yes | down | 110.757 | 162.694 | 157.684 | 30.611 | 40.591 | 28.879 |
| SRG1 | 0.399 | -1.327 | 5.34E-07 | 8.43E-06 | yes | down | 88.314 | 140.432 | 133.464 | 49.51 | 52.26 | 37.267 |
| YBR092C | 0.286 | -1.807 | 7.26E-06 | 8.92E-05 | yes | down | 326.216 | 123.981 | 165.546 | 27.779 | 42.74 | 87.157 |
| YKL107W | 0.436 | -1.197 | 0.000218 | 0.001823 | yes | down | 14.163 | 20.932 | 14.637 | 9.197 | 5.196 | 6.179 |
| YOL083W | 0.428 | -1.223 | 6.36E-08 | 1.17E-06 | yes | down | 16.807 | 15.034 | 16.279 | 6.842 | 6.18 | 7.472 |
| YLR307C-A | 0.35 | -1.515 | 0.001 | 0.006736 | yes | down | 155.069 | 91.672 | 166.229 | 20.778 | 67.696 | 28.411 |
| YGL125W | 0.089 | -3.496 | 5.27E-19 | 2.78E-17 | yes | down | 31.763 | 27.101 | 74.916 | 1.879 | 3.095 | 4.408 |
| YBL097W | 0.262 | -1.933 | 7.42E-10 | 1.83E-08 | yes | down | 3.32 | 5.504 | 4.609 | 1.215 | 1.013 | 1.038 |
| YDR098C-B | 0.461 | -1.116 | 4.93E-05 | 0.000498 | yes | down | 9.338 | 17.263 | 10.414 | 5.318 | 6.719 | 4.296 |
| YNL286W | 0.043 | -4.535 | 3.96E-14 | 1.47E-12 | yes | down | 4.14 | 3.657 | 6.646 | 0 | 0 | 0 |
| NME1 | 0.125 | -2.996 | 4.3E-06 | 5.57E-05 | yes | down | 5.034 | 14.813 | 36.751 | 0 | 0 | 0 |
| Q0045 | 0.296 | -1.755 | 0.007056 | 0.03343 | yes | down | 0.073 | 0.197 | 1.157 | 0 | 0 | 0.041 |
| RDN18-1 | 0.322 | -1.633 | 0.000552 | 0.004094 | yes | down | 613.355 | 2516.778 | 5636.776 | 759.94 | 925.269 | 671.218 |
| RDN18-2 | 0.322 | -1.633 | 0.000552 | 0.004094 | yes | down | 613.355 | 2516.778 | 5636.776 | 759.94 | 925.269 | 671.218 |
| RDN58-1 | 0.222 | -2.171 | 0.000333 | 0.00265 | yes | down | 486.301 | 3934.087 | 16931.48 | 816.376 | 568.939 | 714.46 |
| RDN58-2 | 0.222 | -2.171 | 0.000333 | 0.00265 | yes | down | 486.301 | 3934.087 | 16931.48 | 816.376 | 568.939 | 714.46 |
| SCR1 | 0.247 | -2.016 | 0.001676 | 0.01034 | yes | down | 26.465 | 182.408 | 583.65 | 27.087 | 24.994 | 21.387 |
| snR17a | 0.281 | -1.829 | 0.005243 | 0.02595 | yes | down | 26.994 | 224.285 | 738.446 | 36.294 | 30.758 | 7.37 |
| snR19 | 0.426 | -1.23 | 4.25E-05 | 0.000438 | yes | down | 136.876 | 304.764 | 261.231 | 97.973 | 108.325 | 80.143 |
| snR190 | 0.262 | -1.933 | 0.001526 | 0.009592 | yes | down | 96.731 | 484.942 | 1233.137 | 97.73 | 39.948 | 89.234 |
| tR(UCU)M2 | 0.476 | -1.071 | 4.68E-08 | 8.7E-07 | yes | down | 14.445 | 20.403 | 15.824 | 7.636 | 8.3 | 8.286 |
| YAL028W | 0.326 | -1.617 | 9.33E-08 | 1.66E-06 | yes | down | 5.235 | 8.755 | 7.388 | 2.028 | 2.035 | 2.565 |
| YAL044C | 0.23 | -2.118 | 6.42E-13 | 2.2E-11 | yes | down | 672.486 | 1550.698 | 929.143 | 238.212 | 217.152 | 212.04 |
| YAL064W | 0.446 | -1.165 | 6.27E-06 | 7.85E-05 | yes | down | 1.714 | 1.576 | 1.83 | 0.776 | 0.634 | 0.967 |
| YAR015W | 0.337 | -1.57 | 6.77E-07 | 1.05E-05 | yes | down | 346.598 | 693.873 | 528.039 | 113.704 | 143.73 | 244.421 |
| YAR027W | 0.447 | -1.161 | 3.23E-08 | 6.3E-07 | yes | down | 84.137 | 92.09 | 87.97 | 30.583 | 47.632 | 39.527 |
| YAR075W | 0.346 | -1.532 | 0.006842 | 0.03261 | yes | down | 3.976 | 8.718 | 5.944 | 0.981 | 2.517 | 1.038 |
| YBL001C | 0.186 | -2.425 | 3.66E-26 | 3.13E-24 | yes | down | 1248.086 | 1599.987 | 1090.09 | 235.95 | 222.196 | 211.072 |
| YBL015W | 0.359 | -1.478 | 4.57E-05 | 0.000467 | yes | down | 143.77 | 362.648 | 300.711 | 123.677 | 88.716 | 53.636 |
| YBL029W | 0.5 | -1.001 | 9.27E-07 | 1.37E-05 | yes | down | 30.332 | 39.341 | 35.425 | 16.151 | 16.562 | 19.87 |
| YBL085W | 0.453 | -1.143 | 3.16E-05 | 0.00034 | yes | down | 3.101 | 4.925 | 4.342 | 1.72 | 1.694 | 2.077 |
| YBR005W | 0.395 | -1.34 | 4.45E-09 | 9.83E-08 | yes | down | 1433.641 | 2061.917 | 1262.075 | 540.356 | 723.212 | 580.182 |
| YBR007C | 0.461 | -1.117 | 1.86E-08 | 3.77E-07 | yes | down | 26.647 | 32.482 | 26.169 | 14.459 | 13.013 | 11.859 |
| YBR011C | 0.275 | -1.864 | 3.49E-15 | 1.39E-13 | yes | down | 788.505 | 1085.198 | 785.838 | 227.912 | 184.936 | 295.288 |
| YBR016W | 0.455 | -1.136 | 0.000143 | 0.001252 | yes | down | 520.244 | 908.824 | 602.006 | 345.728 | 206.969 | 310.18 |
| YBR043C | 0.325 | -1.623 | 3.67E-11 | 9.96E-10 | yes | down | 119.475 | 68.277 | 87.011 | 28.489 | 30.162 | 29.317 |
| YBR044C | 0.446 | -1.166 | 3.07E-08 | 6.05E-07 | yes | down | 14.956 | 20.575 | 16.338 | 7.487 | 7.865 | 7.716 |
| YBR053C | 0.317 | -1.656 | 1.82E-17 | 8.73E-16 | yes | down | 32.602 | 37.05 | 33.655 | 11.076 | 10.931 | 10.475 |
| YBR056W | 0.266 | -1.912 | 9.82E-10 | 2.39E-08 | yes | down | 25.79 | 12.018 | 18.731 | 4.879 | 4.618 | 4.672 |
| YBR056W-A | 0.244 | -2.036 | 5.84E-09 | 1.27E-07 | yes | down | 3252.475 | 5622.175 | 3440.109 | 473.947 | 1148.061 | 966.322 |
| YBR068C | 0.297 | -1.75 | 4.35E-20 | 2.51E-18 | yes | down | 2448.284 | 2215.573 | 1910.543 | 535.692 | 669.078 | 751.157 |
| YBR072W | 0.222 | -2.173 | 2.13E-22 | 1.38E-20 | yes | down | 1465.241 | 2311.419 | 2272.354 | 426.727 | 465.97 | 401.409 |
| YBR111C | 0.322 | -1.635 | 1.88E-13 | 6.64E-12 | yes | down | 179.199 | 231.943 | 277.786 | 69.989 | 80.321 | 66.89 |
| YBR145W | 0.449 | -1.155 | 9.51E-06 | 0.000114 | yes | down | 86.973 | 166.622 | 106.454 | 45.799 | 52.043 | 61.016 |
| YBR149W | 0.339 | -1.559 | 2.18E-16 | 9.62E-15 | yes | down | 102.914 | 98.075 | 118.471 | 36.34 | 32.66 | 38.886 |
| YBR174C | 0.452 | -1.147 | 0.01241 | 0.05323 | yes | down | 36.177 | 115.559 | 58.548 | 31.405 | 23.575 | 25.378 |
| YBR230C | 0.422 | -1.245 | 1.74E-08 | 3.56E-07 | yes | down | 585.695 | 800.726 | 725.471 | 321.314 | 283.779 | 249.582 |
| YBR230W-A | 0.464 | -1.108 | 0.02195 | 0.0816 | yes | down | 457.684 | 611.35 | 509.11 | 259.906 | 252.017 | 70.921 |
| YBR240C | 0.313 | -1.676 | 2.57E-13 | 8.94E-12 | yes | down | 121.308 | 169.836 | 201.516 | 47.762 | 47.661 | 56.272 |
| YBR248C | 0.441 | -1.18 | 2.58E-08 | 5.14E-07 | yes | down | 84.256 | 89.135 | 111.103 | 44.397 | 35.414 | 45.594 |
| YBR284W | 0.427 | -1.229 | 8.2E-05 | 0.000788 | yes | down | 5.545 | 12.301 | 6.982 | 3.178 | 3.7 | 3.329 |
| YBR286W | 0.352 | -1.505 | 8.86E-23 | 5.96E-21 | yes | down | 447.734 | 488.193 | 398.215 | 152.25 | 162.8 | 158.281 |
| YCL007C | 0.238 | -2.072 | 1.68E-07 | 2.88E-06 | yes | down | 54.927 | 99.823 | 49.38 | 17.88 | 9.597 | 12.623 |
| YCL009C | 0.358 | -1.482 | 1.03E-07 | 1.83E-06 | yes | down | 1954.177 | 4056.972 | 2259.843 | 1048.354 | 914.395 | 878.717 |
| YCL014W | 0.427 | -1.228 | 5.8E-07 | 9.07E-06 | yes | down | 2.718 | 3.694 | 2.611 | 1.131 | 1.353 | 1.344 |
| YCL030C | 0.332 | -1.591 | 2.86E-09 | 6.5E-08 | yes | down | 235.358 | 277.847 | 413.802 | 71.101 | 56.983 | 111.476 |
| YCL033C | 0.292 | -1.778 | 1.76E-08 | 3.59E-07 | yes | down | 237.373 | 585.492 | 333.921 | 101.861 | 120.155 | 89.437 |
| YCL064C | 0.406 | -1.302 | 2.09E-07 | 3.52E-06 | yes | down | 214.2 | 151.908 | 198.737 | 93.318 | 65.529 | 65.627 |
| YCR020C | 0.335 | -1.579 | 3.43E-12 | 1.06E-10 | yes | down | 68.524 | 62.625 | 73.353 | 17.507 | 23.556 | 26.141 |
| YCR069W | 0.393 | -1.346 | 1.66E-06 | 2.33E-05 | yes | down | 66.463 | 118.231 | 104.091 | 45.453 | 33.947 | 29.918 |
| YCR104W | 0.361 | -1.469 | 0.02507 | 0.09035 | yes | down | 4.624 | 3.768 | 2.146 | 0.458 | 0 | 0.489 |
| YDL029W | 0.442 | -1.177 | 6.98E-08 | 1.26E-06 | yes | down | 39.679 | 29.601 | 39.451 | 13.712 | 18.322 | 16.277 |
| YDL066W | 0.38 | -1.396 | 4.66E-07 | 7.49E-06 | yes | down | 65.342 | 46.162 | 52.021 | 26.377 | 15.881 | 17.947 |
| YDL072C | 0.495 | -1.015 | 0.000851 | 0.005883 | yes | down | 183.331 | 423.844 | 259.688 | 105.778 | 167.135 | 143.012 |
| YDL104C | 0.027 | -5.187 | 5.37E-19 | 2.81E-17 | yes | down | 8.481 | 3.411 | 4.144 | 0 | 0 | 0 |
| YDL126C | 0.286 | -1.804 | 6.26E-31 | 6.31E-29 | yes | down | 222.618 | 202.133 | 221.602 | 61.063 | 60.059 | 65.342 |
| YDL130W-A | 0.107 | -3.229 | 1.06E-09 | 2.55E-08 | yes | down | 342.905 | 348.352 | 361.445 | 35.733 | 19.77 | 0 |
| YDL170W | 0.489 | -1.032 | 0.000418 | 0.003218 | yes | down | 168.84 | 137.391 | 202.831 | 112.769 | 61.138 | 68.793 |
| YDL181W | 0.488 | -1.034 | 0.000255 | 0.002077 | yes | down | 855.598 | 1417.715 | 1159.507 | 521.522 | 596.65 | 426.42 |
| YDL207W | 0.461 | -1.116 | 2.28E-05 | 0.000254 | yes | down | 9.831 | 14.222 | 11.63 | 4.982 | 6.795 | 4.479 |
| YDL210W | 0.308 | -1.698 | 7E-18 | 3.5E-16 | yes | down | 695.248 | 921.346 | 736.35 | 263.598 | 246.216 | 208.212 |
| YDL238C | 0.348 | -1.524 | 3.33E-07 | 5.52E-06 | yes | down | 12.849 | 25.993 | 19.345 | 7.216 | 6.719 | 5.334 |
| YDR019C | 0.255 | -1.97 | 4.37E-17 | 2.06E-15 | yes | down | 61.967 | 93.149 | 84.361 | 16.207 | 19.098 | 24.309 |
| YDR024W | 0.404 | -1.307 | 0.04602 | 0.1443 | yes | down | 2.745 | 4.113 | 0.801 | 0 | 0.795 | 0.489 |
| YDR030C | 0.5 | -1.001 | 0.000146 | 0.001266 | yes | down | 6.876 | 9.752 | 8.129 | 3.542 | 4.817 | 3.909 |
| YDR031W | 0.432 | -1.211 | 0.001633 | 0.01013 | yes | down | 107.848 | 303.606 | 165.576 | 92.944 | 76.583 | 53.707 |
| YDR127W | 0.493 | -1.02 | 7.46E-07 | 1.12E-05 | yes | down | 132.124 | 99.798 | 131.644 | 57.67 | 54.39 | 69.139 |
| YDR154C | 0.344 | -1.54 | 1.47E-06 | 2.08E-05 | yes | down | 1860.318 | 4587.855 | 2776.371 | 740.433 | 1088.447 | 1082.165 |
| YDR155C | 0.315 | -1.665 | 3.89E-08 | 7.42E-07 | yes | down | 2430.136 | 5871.702 | 3527.723 | 937.678 | 1321.224 | 1235.733 |
| YDR158W | 0.311 | -1.685 | 1.95E-19 | 1.05E-17 | yes | down | 1420.974 | 1226.713 | 1330.612 | 340.933 | 407.53 | 488.515 |
| YDR204W | 0.411 | -1.282 | 1.68E-07 | 2.88E-06 | yes | down | 41.904 | 65.309 | 44.445 | 20.535 | 23.177 | 17.468 |
| YDR231C | 0.495 | -1.015 | 0.00363 | 0.01939 | yes | down | 57.8 | 136.96 | 65.866 | 41.434 | 51.825 | 29.246 |
| YDR236C | 0.473 | -1.079 | 1.61E-05 | 0.000183 | yes | down | 102.039 | 176.189 | 114.673 | 63.754 | 60.305 | 57.718 |
| YDR242W | 0.494 | -1.018 | 0.001659 | 0.01027 | yes | down | 41.503 | 21.265 | 43.189 | 11.562 | 22.165 | 17.936 |
| YDR320C-A | 0.391 | -1.354 | 1.54E-05 | 0.000177 | yes | down | 922.335 | 1177.744 | 771.894 | 243.259 | 391.157 | 375.543 |
| YDR340W | 0.339 | -1.561 | 0.000885 | 0.006074 | yes | down | 33.897 | 35.721 | 41.745 | 8.59 | 11.158 | 10.251 |
| YDR354W | 0.331 | -1.595 | 8.6E-16 | 3.63E-14 | yes | down | 116.594 | 133.229 | 109.896 | 44.65 | 38.178 | 34.946 |
| YDR368W | 0.416 | -1.267 | 1.79E-08 | 3.64E-07 | yes | down | 61.521 | 43.158 | 48.994 | 18.469 | 22.591 | 22.548 |
| YDR377W | 0.319 | -1.65 | 2.07E-06 | 2.86E-05 | yes | down | 559.422 | 1260.451 | 865.927 | 274.291 | 318.001 | 164.939 |
| YDR408C | 0.326 | -1.616 | 1.87E-06 | 2.61E-05 | yes | down | 421.953 | 1315.344 | 697.819 | 217.509 | 250.087 | 266.714 |
| YDR411C | 0.426 | -1.232 | 2.44E-05 | 0.000269 | yes | down | 68.716 | 151.883 | 79.92 | 39.369 | 43.639 | 40.667 |
| YDR425W | 0.019 | -5.696 | 2.03E-23 | 1.48E-21 | yes | down | 4.113 | 9.026 | 2.641 | 0 | 0 | 0 |
| YDR436W | 0.476 | -1.071 | 0.009615 | 0.04344 | yes | down | 4.742 | 17.005 | 12.985 | 7.225 | 4.117 | 3.807 |
| YDR487C | 0.498 | -1.005 | 7.43E-07 | 1.12E-05 | yes | down | 726.994 | 1001.924 | 694.901 | 384.013 | 445.405 | 368 |
| YDR502C | 0.331 | -1.595 | 1.95E-05 | 0.00022 | yes | down | 127.227 | 40.892 | 77.269 | 26.956 | 17.414 | 30.284 |
| YDR512C | 0.449 | -1.156 | 0.00015 | 0.001296 | yes | down | 63.408 | 111.705 | 59.992 | 34.293 | 40.137 | 26.365 |
| YEL020W-A | 0.192 | -2.382 | 0.000143 | 0.001252 | yes | down | 58.183 | 36.028 | 29.116 | 1.626 | 0 | 5.813 |
| YEL023C | 0.389 | -1.363 | 7.88E-06 | 9.61E-05 | yes | down | 3.019 | 3.423 | 2.769 | 1.15 | 1.202 | 1.11 |
| YEL061C | 0.425 | -1.234 | 0.003034 | 0.01667 | yes | down | 16.032 | 54.375 | 33.171 | 16.983 | 16.884 | 5.975 |
| YEL066W | 0.455 | -1.137 | 6.42E-05 | 0.000637 | yes | down | 94.424 | 131.135 | 85.636 | 56.212 | 45.711 | 33.501 |
| YER032W | 0.392 | -1.351 | 0.000108 | 0.000991 | yes | down | 1.87 | 1.675 | 1.899 | 0.551 | 0.606 | 0.875 |
| YER034W | 0.38 | -1.396 | 1.03E-06 | 1.51E-05 | yes | down | 39.296 | 43.133 | 47.452 | 15.871 | 11.574 | 19.596 |
| YER039C | 0.443 | -1.175 | 3.46E-07 | 5.71E-06 | yes | down | 36.943 | 49.745 | 37.413 | 16.6 | 20.584 | 17.081 |
| YER042W | 0.308 | -1.697 | 1.09E-09 | 2.6E-08 | yes | down | 76.686 | 64.804 | 60.566 | 17.965 | 26.774 | 14.618 |
| YER055C | 0.329 | -1.606 | 3.17E-22 | 2.01E-20 | yes | down | 1139.763 | 1415.856 | 1378.637 | 423.25 | 413 | 453.569 |
| YER057C | 0.008 | -6.969 | 4.08E-40 | 7.69E-38 | yes | down | 112.928 | 234.689 | 144.53 | 0 | 0 | 0 |
| YER069W | 0.386 | -1.373 | 0.000238 | 0.001957 | yes | down | 123.488 | 63.93 | 94.082 | 27.218 | 18.436 | 56.761 |
| YER141W | 0.497 | -1.008 | 0.000711 | 0.005105 | yes | down | 33.013 | 52.614 | 45.602 | 27.741 | 21.313 | 14.272 |
| YER147C | 0.008 | -7.025 | 6.41E-42 | 1.29E-39 | yes | down | 12.649 | 8.041 | 12.432 | 0 | 0 | 0 |
| YER152C | 0.004 | -7.992 | 3.55E-54 | 1.54E-51 | yes | down | 28.745 | 87.596 | 37.809 | 0 | 0 | 0 |
| YER185W | 0.333 | -1.588 | 8.6E-10 | 2.11E-08 | yes | down | 18.449 | 28.505 | 22.41 | 6.711 | 7.836 | 7.818 |
| YFL014W | 0.139 | -2.849 | 1.29E-16 | 5.92E-15 | yes | down | 657.457 | 858.093 | 1320.87 | 61.034 | 152.267 | 118.48 |
| YFL021C-A | 0.168 | -2.575 | 7.5E-12 | 2.23E-10 | yes | down | 38.539 | 120.645 | 56.59 | 12.291 | 8.205 | 9.864 |
| YFL030W | 0.419 | -1.255 | 8.58E-05 | 0.000816 | yes | down | 236.297 | 578.461 | 370.039 | 189.141 | 167.012 | 116.271 |
| YFL031W | 0.485 | -1.045 | 0.001241 | 0.008057 | yes | down | 1865.425 | 5048.652 | 2895.158 | 1750.727 | 1579.441 | 1221.564 |
| YFL033C | 0.481 | -1.056 | 0.000237 | 0.001949 | yes | down | 47.905 | 130.729 | 69.298 | 40.743 | 41.358 | 28.818 |
| YFL044C | 0.455 | -1.137 | 1.3E-07 | 2.25E-06 | yes | down | 75.948 | 55.446 | 66.915 | 31.648 | 29.66 | 28.431 |
| YFL059W | 0.088 | -3.512 | 1.1E-36 | 1.55E-34 | yes | down | 203.795 | 170.476 | 173.122 | 10.422 | 11.811 | 21.357 |
| YFL060C | 0.478 | -1.065 | 0.002646 | 0.01493 | yes | down | 35.521 | 18.925 | 18.543 | 7.58 | 13.032 | 12.867 |
| YFR031C | 0.472 | -1.083 | 0.008434 | 0.03882 | yes | down | 0.73 | 0.936 | 0.979 | 0.243 | 0.539 | 0.397 |
| YFR047C | 0.39 | -1.357 | 5.94E-09 | 1.29E-07 | yes | down | 105.741 | 152.893 | 95.803 | 38.284 | 52.573 | 45.513 |
| YGL009C | 0 | -12.49 | 8E-174 | 4.5E-170 | yes | down | 1088.119 | 531.794 | 1210.39 | 0 | 0 | 0 |
| YGL026C | 0.418 | -1.26 | 2.77E-10 | 7.09E-09 | yes | down | 688.427 | 827.581 | 668.673 | 351.43 | 290.375 | 267.426 |
| YGL113W | 0.433 | -1.207 | 1.97E-05 | 0.000222 | yes | down | 5.791 | 9.075 | 7.892 | 3.271 | 3.814 | 2.545 |
| YGL116W | 0.429 | -1.221 | 0.004557 | 0.02319 | yes | down | 1.404 | 1.096 | 1.721 | 0.467 | 0.596 | 0.601 |
| YGL121C | 0.296 | -1.754 | 1.75E-10 | 4.53E-09 | yes | down | 264.431 | 160.761 | 170.392 | 43.509 | 65.112 | 55.305 |
| YGL126W | 0.445 | -1.168 | 2.33E-05 | 0.000259 | yes | down | 422.528 | 826.904 | 476.8 | 284.376 | 251.345 | 211.072 |
| YGL187C | 0.495 | -1.015 | 1.12E-06 | 1.62E-05 | yes | down | 285.661 | 371.834 | 388.701 | 166.167 | 188.552 | 152.55 |
| YGL223C | 0.487 | -1.038 | 1.24E-06 | 1.79E-05 | yes | down | 31.207 | 26.276 | 24.665 | 13.721 | 14.243 | 12.073 |
| YGL242C | 0.289 | -1.79 | 1.91E-10 | 4.92E-09 | yes | down | 30.359 | 31.768 | 28.73 | 7.206 | 9.634 | 8.032 |
| YGL259W | 0.359 | -1.477 | 0.006835 | 0.03261 | yes | down | 4.952 | 5.085 | 6.557 | 2.187 | 1.249 | 0.926 |
| YGR008C | 0.256 | -1.967 | 5.77E-07 | 9.06E-06 | yes | down | 301.611 | 716.787 | 548.076 | 81.111 | 160.103 | 93.224 |
| YGR032W | 0.474 | -1.077 | 0.000154 | 0.001325 | yes | down | 92.883 | 157.178 | 131.802 | 48.669 | 84.93 | 45.095 |
| YGR037C | 0.343 | -1.542 | 1.8E-07 | 3.05E-06 | yes | down | 337.743 | 470.438 | 392.904 | 121.181 | 145.661 | 104.157 |
| YGR043C | 0.267 | -1.906 | 2.7E-11 | 7.46E-10 | yes | down | 127.2 | 64.324 | 78.525 | 19.553 | 26.348 | 23.138 |
| YGR044C | 0.425 | -1.234 | 0.000399 | 0.003076 | yes | down | 14.172 | 9.136 | 12.145 | 5.711 | 3.17 | 5.365 |
| YGR066C | 0.317 | -1.658 | 2.49E-06 | 3.38E-05 | yes | down | 12.713 | 9.764 | 7.853 | 3.384 | 2.271 | 3.176 |
| YGR086C | 0.45 | -1.152 | 5.02E-06 | 6.37E-05 | yes | down | 89.426 | 128.931 | 138.616 | 52.856 | 41.433 | 63.866 |
| YGR133W | 0.018 | -5.76 | 3.38E-24 | 2.58E-22 | yes | down | 14.272 | 45.645 | 33.853 | 0 | 0 | 0 |
| YGR135W | 0.406 | -1.299 | 0.000393 | 0.003045 | yes | down | 50.121 | 20.711 | 47.343 | 11.777 | 12.625 | 20.939 |
| YGR175C | 0.469 | -1.093 | 8.58E-06 | 0.000104 | yes | down | 1226.518 | 937.698 | 746.793 | 526.252 | 411.391 | 412.943 |
| YGR192C | 0.489 | -1.033 | 0.001047 | 0.00703 | yes | down | 10732.62 | 23851.8 | 11797.84 | 5922.822 | 6295.087 | 9752.882 |
| YGR213C | 0.013 | -6.278 | 5.03E-32 | 5.46E-30 | yes | down | 14.299 | 13.963 | 12.006 | 0 | 0 | 0 |
| YGR236C | 0.267 | -1.907 | 0.000389 | 0.003021 | yes | down | 25.371 | 125.533 | 76.32 | 11.749 | 17.934 | 11.421 |
| YGR247W | 0.473 | -1.08 | 0.002177 | 0.01274 | yes | down | 10.159 | 13.409 | 11.828 | 3.851 | 4.59 | 7.533 |
| YGR254W | 0.253 | -1.981 | 3.63E-12 | 1.11E-10 | yes | down | 6738.392 | 13981.84 | 7591.428 | 1799.639 | 2755.203 | 2255.254 |
| YGR268C | 0.488 | -1.036 | 0.000236 | 0.001949 | yes | down | 118.755 | 236.819 | 168.721 | 94.982 | 79.943 | 71.847 |
| YHL036W | 0.372 | -1.425 | 3.14E-08 | 6.13E-07 | yes | down | 102.477 | 198.525 | 136.816 | 54.417 | 58.544 | 46.154 |
| YHR001W-A | 0.401 | -1.318 | 0.000128 | 0.001131 | yes | down | 1079.93 | 1906.758 | 1356.483 | 502.212 | 710.445 | 334.652 |
| YHR008C | 0.315 | -1.666 | 7.12E-18 | 3.53E-16 | yes | down | 567.419 | 770.473 | 601.126 | 205.05 | 216.489 | 179.75 |
| YHR018C | 0.166 | -2.595 | 7.04E-33 | 7.96E-31 | yes | down | 681.633 | 594.468 | 687.81 | 116.704 | 81.996 | 115.904 |
| YHR029C | 0.403 | -1.312 | 8.92E-11 | 2.35E-09 | yes | down | 215.286 | 232.313 | 157.041 | 74.241 | 86.236 | 81.609 |
| YHR063C | 0.463 | -1.112 | 1.63E-05 | 0.000186 | yes | down | 125.768 | 68.301 | 96.08 | 40.612 | 43.979 | 48.729 |
| YHR087W | 0.112 | -3.164 | 9.54E-07 | 1.41E-05 | yes | down | 15.795 | 23.321 | 9.573 | 0 | 0 | 0.01 |
| YHR138C | 0.34 | -1.556 | 7.48E-08 | 1.34E-06 | yes | down | 884.096 | 1580.163 | 1246.469 | 374.591 | 506.249 | 288.03 |
| YHR145C | 0.238 | -2.07 | 0.000799 | 0.005617 | yes | down | 7.861 | 25.144 | 14.499 | 2.159 | 0 | 2.82 |
| YHR174W | 0.397 | -1.331 | 1.03E-05 | 0.000122 | yes | down | 11922.18 | 19933.21 | 10259.59 | 3702.101 | 5072.355 | 7447.342 |
| YHR208W | 0.077 | -3.702 | 1.12E-67 | 9.03E-65 | yes | down | 5464.891 | 8946.476 | 6972.876 | 531.692 | 528.745 | 493.768 |
| YIL024C | 0.22 | -2.184 | 0.000825 | 0.005733 | yes | down | 0.255 | 2.82 | 9.593 | 0 | 0 | 0 |
| YIL029C | 0.373 | -1.421 | 0.000866 | 0.005972 | yes | down | 25.763 | 27.865 | 23.291 | 8.132 | 12.899 | 3.97 |
| YIL046W | 0.374 | -1.419 | 0.00197 | 0.01181 | yes | down | 129.024 | 68.843 | 70.732 | 16.291 | 60.996 | 11.421 |
| YIL051C | 0.231 | -2.116 | 0.001099 | 0.007336 | yes | down | 536.021 | 893.605 | 682.331 | 66.418 | 76.119 | 2.474 |
| YIL059C | 0.467 | -1.097 | 0.03018 | 0.1052 | yes | down | 20.364 | 31.362 | 12.303 | 8.365 | 4.581 | 11.533 |
| YIL060W | 0.213 | -2.232 | 0.000667 | 0.004859 | yes | down | 11.782 | 15.564 | 0.178 | 0 | 0 | 0.662 |
| YIL062C | 0.342 | -1.55 | 7.29E-07 | 1.1E-05 | yes | down | 82.076 | 129.953 | 113.753 | 25.909 | 50.538 | 27.922 |
| YIL074C | 0.39 | -1.358 | 0.004726 | 0.02392 | yes | down | 22.571 | 12.744 | 25.684 | 7.524 | 1.543 | 11.289 |
| YIL082W | 0.043 | -4.525 | 6.29E-14 | 2.32E-12 | yes | down | 4.049 | 7.991 | 3.234 | 0 | 0 | 0 |
| YIL087C | 0.28 | -1.836 | 2.03E-08 | 4.07E-07 | yes | down | 119.567 | 248.776 | 161.62 | 50.08 | 54.106 | 30.569 |
| YIL116W | 0.261 | -1.937 | 1.44E-33 | 1.77E-31 | yes | down | 195.396 | 179.514 | 201.872 | 49.22 | 49.554 | 51.895 |
| YIL136W | 0.207 | -2.271 | 1.28E-31 | 1.37E-29 | yes | down | 77.899 | 81.329 | 99.66 | 15.899 | 19.146 | 17.947 |
| YIL159W | 0.432 | -1.211 | 0.000389 | 0.003021 | yes | down | 1.705 | 1.699 | 1.711 | 0.449 | 1.032 | 0.662 |
| YIL163C | 0.248 | -2.01 | 0.002058 | 0.01219 | yes | down | 6.493 | 2.229 | 11.235 | 0 | 0 | 0.234 |
| YIL164C | 0.489 | -1.032 | 5.48E-07 | 8.62E-06 | yes | down | 93.594 | 117.554 | 91.738 | 46.818 | 55.175 | 44.454 |
| YIL165C | 0.457 | -1.131 | 0.000592 | 0.004349 | yes | down | 98.701 | 164.984 | 94.122 | 55.37 | 56.689 | 38.631 |
| YIR006C | 0.414 | -1.274 | 0.04194 | 0.135 | yes | down | 7.004 | 8.151 | 6.28 | 2.028 | 0.189 | 3.339 |
| YIR019C | 0.456 | -1.132 | 0.0001 | 0.000931 | yes | down | 3.484 | 2.537 | 2.838 | 1.159 | 1.098 | 1.731 |
| YIR030C | 0.425 | -1.233 | 1.48E-05 | 0.000171 | yes | down | 57.006 | 42.591 | 36.85 | 22.432 | 14.489 | 19.056 |
| YIR034C | 0.455 | -1.137 | 6.02E-08 | 1.11E-06 | yes | down | 250.697 | 330.19 | 271.991 | 150.1 | 117.827 | 116.739 |
| YIR036C | 0.195 | -2.358 | 4.16E-15 | 1.64E-13 | yes | down | 55.866 | 91.228 | 49.38 | 12.955 | 13.297 | 8.815 |
| YIR037W | 0.257 | -1.96 | 1.58E-17 | 7.67E-16 | yes | down | 433.836 | 606.991 | 467.207 | 130.201 | 141.563 | 98.365 |
| YIR038C | 0.126 | -2.992 | 3.75E-47 | 1.11E-44 | yes | down | 240.474 | 257.949 | 203.642 | 31.938 | 27.19 | 25.082 |
| YIR039C | 0.26 | -1.942 | 1.28E-20 | 7.52E-19 | yes | down | 17.108 | 16.524 | 15.824 | 4.309 | 4.24 | 4.143 |
| YJL001W | 0.43 | -1.219 | 2.99E-09 | 6.77E-08 | yes | down | 242.726 | 335.423 | 260.974 | 113.208 | 136.604 | 105.022 |
| YJL048C | 0.481 | -1.055 | 4.01E-06 | 5.23E-05 | yes | down | 141.426 | 140.801 | 113.466 | 45.332 | 78.012 | 67.694 |
| YJL066C | 0.404 | -1.309 | 2.64E-09 | 6.09E-08 | yes | down | 145.421 | 181.336 | 145.48 | 63.839 | 74.435 | 49.717 |
| YJL067W | 0.373 | -1.421 | 0.000158 | 0.001358 | yes | down | 37.399 | 40.289 | 39.144 | 13.497 | 14.101 | 10.76 |
| YJL077C | 0.241 | -2.052 | 0.00168 | 0.01036 | yes | down | 1.778 | 4.901 | 7.239 | 0 | 0 | 0 |
| YJL121C | 0.321 | -1.64 | 1.6E-05 | 0.000183 | yes | down | 109.134 | 250.179 | 129.023 | 24.89 | 43.146 | 74.036 |
| YJL163C | 0.387 | -1.369 | 4.98E-07 | 7.88E-06 | yes | down | 22.361 | 45.473 | 30.807 | 11.721 | 13.874 | 11.574 |
| YJL167W | 0.469 | -1.092 | 0.00216 | 0.01268 | yes | down | 31.253 | 13.84 | 18.168 | 8.664 | 7.06 | 12.765 |
| YJL171C | 0.48 | -1.058 | 5.27E-11 | 1.42E-09 | yes | down | 887.06 | 821.622 | 764.031 | 348.682 | 437.843 | 415.589 |
| YJL199C | 0.334 | -1.582 | 0.00046 | 0.00349 | yes | down | 48.808 | 84.702 | 43.763 | 23.713 | 12.474 | 11.371 |
| YJL201W | 0.281 | -1.832 | 0.001555 | 0.009722 | yes | down | 4.231 | 2.561 | 3.748 | 0.28 | 0.085 | 1.547 |
| YJL210W | 0.328 | -1.607 | 1.52E-06 | 2.15E-05 | yes | down | 235.13 | 602.681 | 294.827 | 125.481 | 133.339 | 85.997 |
| YJL211C | 0.357 | -1.485 | 1.29E-05 | 0.00015 | yes | down | 194.374 | 494.436 | 244.398 | 118.882 | 93.542 | 91.728 |
| YJR005C-A | 0.336 | -1.572 | 0.01633 | 0.06572 | yes | down | 13.953 | 16.069 | 9.494 | 1.29 | 0 | 2.789 |
| YJR010W | 0.401 | -1.32 | 0.000508 | 0.003808 | yes | down | 36.907 | 10.639 | 21.372 | 7.917 | 8.272 | 9.783 |
| YJR073C | 0.15 | -2.733 | 4.98E-22 | 3.09E-20 | yes | down | 953.697 | 1085.284 | 516.122 | 118.255 | 134.824 | 97.713 |
| YJR074W | 0.244 | -2.036 | 1.32E-15 | 5.4E-14 | yes | down | 257.026 | 222.758 | 145.796 | 41.846 | 47.008 | 56.883 |
| YJR085C | 0.357 | -1.484 | 3.22E-05 | 0.000345 | yes | down | 662.071 | 1905.551 | 1063.002 | 441.196 | 390.741 | 320.625 |
| YJR099W | 0.468 | -1.094 | 0.01953 | 0.07495 | yes | down | 9.712 | 7.314 | 3.946 | 1.449 | 3.795 | 3.573 |
| YJR142W | 0.436 | -1.197 | 0.002442 | 0.01403 | yes | down | 4.998 | 3.337 | 6.498 | 1.673 | 2.47 | 1.924 |
| YJR150C | 0.227 | -2.142 | 2.23E-11 | 6.22E-10 | yes | down | 26.529 | 49.019 | 23.132 | 7.262 | 6.71 | 6.423 |
| YJR152W | 0.23 | -2.12 | 1.88E-16 | 8.36E-15 | yes | down | 135.261 | 77.832 | 116.028 | 26.283 | 25.345 | 21.316 |
| YKL001C | 0.358 | -1.48 | 4.86E-08 | 9E-07 | yes | down | 183.267 | 124.646 | 149.04 | 38.555 | 72.447 | 47.976 |
| YKL016C | 0.442 | -1.178 | 9.05E-09 | 1.93E-07 | yes | down | 414.001 | 441.612 | 500.664 | 221.145 | 171.053 | 194.51 |
| YKL017C | 0.459 | -1.122 | 0.003149 | 0.01721 | yes | down | 2.289 | 3.608 | 1.721 | 1.094 | 1.287 | 0.906 |
| YKL029C | 0.317 | -1.658 | 1.23E-15 | 5.05E-14 | yes | down | 3582.476 | 4658.767 | 3841.993 | 1373.341 | 1015.385 | 1316.997 |
| YKL030W | 0.29 | -1.788 | 1.32E-20 | 7.69E-19 | yes | down | 942.79 | 1361.407 | 1049.314 | 308.92 | 322.212 | 320.451 |
| YKL065W-A | 0.419 | -1.254 | 0.03762 | 0.1242 | yes | down | 53.86 | 89.997 | 66.223 | 12.749 | 15.417 | 28.91 |
| YKL068W-A | 0.447 | -1.161 | 0.000344 | 0.002731 | yes | down | 630.208 | 387.594 | 500.842 | 210.835 | 245.866 | 142.636 |
| YKL084W | 0.465 | -1.104 | 0.000809 | 0.005656 | yes | down | 166.77 | 323.554 | 215.658 | 124.808 | 76.706 | 99.729 |
| YKL120W | 0.122 | -3.037 | 4.82E-37 | 7.16E-35 | yes | down | 1415.366 | 856.419 | 1228.568 | 112.937 | 154.396 | 138.594 |
| YKL121W | 0.283 | -1.821 | 6.41E-09 | 1.38E-07 | yes | down | 6.402 | 12.018 | 14.202 | 2.879 | 2.555 | 3.247 |
| YKL141W | 0.37 | -1.436 | 1.7E-08 | 3.5E-07 | yes | down | 235.002 | 324.489 | 265.266 | 121.835 | 97.044 | 74.066 |
| YKL148C | 0.346 | -1.532 | 8.26E-07 | 1.24E-05 | yes | down | 156.373 | 309.677 | 301.542 | 107.6 | 83.605 | 59.988 |
| YKL150W | 0.487 | -1.037 | 6.58E-08 | 1.2E-06 | yes | down | 291.899 | 411.396 | 363.413 | 160.391 | 195.829 | 164.236 |
| YKL165C | 0.278 | -1.847 | 2.72E-25 | 2.26E-23 | yes | down | 94.205 | 104.354 | 92.559 | 22.03 | 27.502 | 31.842 |
| YKL168C | 0.483 | -1.049 | 0.001156 | 0.007648 | yes | down | 3.42 | 4.753 | 4.49 | 2.57 | 1.495 | 1.832 |
| YKL192C | 0.427 | -1.228 | 0.000101 | 0.000933 | yes | down | 410.143 | 757.704 | 408.066 | 245.026 | 153.601 | 221.7 |
| YKL221W | 0.293 | -1.77 | 2.32E-10 | 5.96E-09 | yes | down | 7.442 | 10.885 | 8.891 | 2.664 | 2.338 | 2.626 |
| YKR046C | 0.374 | -1.42 | 7.06E-06 | 8.73E-05 | yes | down | 575.536 | 1325.797 | 917.522 | 414.193 | 338.14 | 240.97 |
| YKR049C | 0.091 | -3.463 | 3.04E-39 | 5.2E-37 | yes | down | 176.135 | 249.908 | 202.772 | 15.833 | 19.269 | 15.687 |
| YLL040C | 0.325 | -1.623 | 2.99E-11 | 8.2E-10 | yes | down | 2.417 | 2.45 | 2.196 | 0.692 | 0.965 | 0.611 |
| YLL041C | 0.289 | -1.792 | 3.82E-14 | 1.43E-12 | yes | down | 794.697 | 951.107 | 1087.984 | 320.454 | 261.103 | 210.289 |
| YLL049W | 0.377 | -1.409 | 1.69E-07 | 2.89E-06 | yes | down | 120.387 | 158.655 | 83.105 | 41.098 | 41.935 | 47.508 |
| YLL057C | 0.452 | -1.146 | 3.56E-07 | 5.84E-06 | yes | down | 17.674 | 23.358 | 23.033 | 10.104 | 9.048 | 9.559 |
| YLL058W | 0.236 | -2.084 | 2.19E-15 | 8.76E-14 | yes | down | 10.132 | 10.048 | 9.118 | 2.533 | 2.29 | 1.771 |
| YLR004C | 0.236 | -2.086 | 1.26E-09 | 2.98E-08 | yes | down | 211.674 | 106.694 | 142.285 | 21.413 | 27.029 | 51.651 |
| YLR038C | 0.411 | -1.284 | 8.11E-05 | 0.000782 | yes | down | 1185.662 | 2716.596 | 1908.446 | 700.037 | 828.755 | 626.387 |
| YLR039C | 0.43 | -1.217 | 0.03022 | 0.1052 | yes | down | 0.447 | 0.529 | 0.208 | 0.065 | 0.17 | 0.163 |
| YLR043C | 0.257 | -1.96 | 6.2E-11 | 1.66E-09 | yes | down | 305.305 | 365.394 | 199.835 | 69.045 | 65.595 | 63.887 |
| YLR058C | 0.358 | -1.481 | 7.25E-07 | 1.1E-05 | yes | down | 603.743 | 1386.218 | 1052.825 | 279.123 | 317.783 | 451.503 |
| YLR092W | 0.11 | -3.182 | 3.03E-25 | 2.48E-23 | yes | down | 65.706 | 36.779 | 84.914 | 6.795 | 5.678 | 6.026 |
| YLR154C | 0.316 | -1.661 | 0.001837 | 0.01116 | yes | down | 17.856 | 17.608 | 19.295 | 4.028 | 5.461 | 2.86 |
| YLR179C | 0.188 | -2.413 | 6.76E-35 | 8.68E-33 | yes | down | 634.02 | 684.502 | 476.029 | 107.89 | 110.426 | 107.934 |
| YLR180W | 0.334 | -1.584 | 2.5E-14 | 9.46E-13 | yes | down | 2491.83 | 2088.981 | 1675.698 | 720.384 | 714.609 | 630.388 |
| YLR194C | 0.206 | -2.276 | 1.92E-22 | 1.26E-20 | yes | down | 3283.883 | 5141.173 | 3701.36 | 667.239 | 747.781 | 994.519 |
| YLR231C | 0.404 | -1.306 | 7.33E-13 | 2.47E-11 | yes | down | 52.857 | 58.451 | 50.023 | 19.61 | 25.241 | 20.715 |
| YLR279W | 0.436 | -1.199 | 0.008428 | 0.03882 | yes | down | 20.893 | 50.927 | 22.539 | 14.693 | 12.228 | 7.971 |
| YLR281C | 0.383 | -1.384 | 2.93E-05 | 0.000318 | yes | down | 97.142 | 189.549 | 104.971 | 57.782 | 46.837 | 33.715 |
| YLR290C | 0.378 | -1.402 | 0.02389 | 0.08708 | yes | down | 1.103 | 0 | 2.136 | 0 | 0 | 0 |
| YLR353W | 0.356 | -1.492 | 1.22E-07 | 2.15E-06 | yes | down | 98.09 | 188.478 | 152.769 | 52.557 | 36.673 | 61.759 |
| YLR355C | 0.194 | -2.365 | 4.63E-13 | 1.6E-11 | yes | down | 2689.652 | 4537.396 | 4713.439 | 700.784 | 406.271 | 991.13 |
| YLR356W | 0.214 | -2.227 | 1.91E-14 | 7.38E-13 | yes | down | 196.171 | 387.311 | 257.324 | 67.157 | 51.418 | 46.48 |
| YLR359W | 0.34 | -1.557 | 1.23E-07 | 2.16E-06 | yes | down | 180.795 | 250.573 | 267.491 | 49.463 | 67.8 | 112.698 |
| YLR364W | 0.287 | -1.801 | 3.65E-08 | 7.06E-07 | yes | down | 86.745 | 87.116 | 72.305 | 22.199 | 20.139 | 19.972 |
| YLR395C | 0.445 | -1.169 | 0.000112 | 0.001021 | yes | down | 380.359 | 333.416 | 451.512 | 139.566 | 167.475 | 159.218 |
| YLR408C | 0.131 | -2.937 | 6.58E-06 | 8.22E-05 | yes | down | 4.04 | 16.45 | 14.874 | 0 | 0 | 0 |
| YLR410W-A | 0.188 | -2.408 | 5.24E-05 | 0.000525 | yes | down | 4.268 | 7.142 | 1.513 | 0.533 | 0.625 | 0 |
| YLR414C | 0.466 | -1.101 | 4.18E-08 | 7.83E-07 | yes | down | 1291.768 | 1505.348 | 1209.866 | 473.199 | 731.976 | 664.621 |
| YML078W | 0.425 | -1.236 | 7.08E-11 | 1.89E-09 | yes | down | 780.334 | 1055.609 | 829.918 | 344.691 | 420.837 | 351.774 |
| YML079W | 0.459 | -1.122 | 3.17E-07 | 5.26E-06 | yes | down | 122.968 | 137.267 | 102.756 | 48.641 | 67.261 | 48.872 |
| YML083C | 0.31 | -1.688 | 0.000136 | 0.001195 | yes | down | 2.599 | 6.194 | 2.848 | 0.794 | 1.278 | 1.008 |
| YMR009W | 0.329 | -1.602 | 1.02E-13 | 3.7E-12 | yes | down | 444.579 | 418.784 | 346.155 | 142.277 | 140.285 | 104.014 |
| YMR015C | 0.384 | -1.382 | 0.000116 | 0.001051 | yes | down | 8.089 | 4.457 | 5.163 | 1.785 | 1.722 | 2.922 |
| YMR032W | 0.396 | -1.338 | 0.01167 | 0.05082 | yes | down | 0.912 | 0.985 | 0.445 | 0.308 | 0.218 | 0.214 |
| YMR034C | 0.195 | -2.362 | 1.19E-11 | 3.44E-10 | yes | down | 31.043 | 35.093 | 28.967 | 3.225 | 9.417 | 4.021 |
| YMR035W | 0.15 | -2.738 | 6.69E-13 | 2.28E-11 | yes | down | 123.169 | 120.017 | 80.316 | 7.973 | 24.124 | 8.683 |
| YMR041C | 0.369 | -1.437 | 0.02015 | 0.07662 | yes | down | 22.936 | 0.702 | 12.847 | 0 | 0 | 0 |
| YMR046C | 0.343 | -1.545 | 6.05E-06 | 7.59E-05 | yes | down | 15.759 | 21.302 | 14.736 | 5.244 | 3.199 | 8.215 |
| YMR056C | 0.002 | -9.052 | 6.93E-78 | 1.3E-74 | yes | down | 110.265 | 244.872 | 135.274 | 0 | 0 | 0 |
| YMR062C | 0.362 | -1.467 | 2.14E-23 | 1.53E-21 | yes | down | 740.39 | 856.948 | 765.416 | 285.535 | 296.167 | 279.021 |
| YMR072W | 0.369 | -1.44 | 0.02112 | 0.07917 | yes | down | 22.607 | 8.595 | 5.884 | 2.449 | 0 | 5.466 |
| YMR086W | 0.33 | -1.599 | 2.71E-09 | 6.23E-08 | yes | down | 9.284 | 14.764 | 9 | 3.767 | 3.861 | 2.922 |
| YMR087W | 0.434 | -1.205 | 4.14E-05 | 0.000429 | yes | down | 14.208 | 20.231 | 18.84 | 6.618 | 9.757 | 6.077 |
| YMR090W | 0.185 | -2.431 | 2.05E-23 | 1.48E-21 | yes | down | 237.419 | 394.539 | 330.747 | 60.623 | 61.261 | 47.294 |
| YMR105C | 0.008 | -6.937 | 4.82E-40 | 8.79E-38 | yes | down | 11.427 | 8.619 | 16.477 | 0 | 0 | 0 |
| YMR110C | 0.409 | -1.29 | 3.2E-12 | 9.98E-11 | yes | down | 36.706 | 41.656 | 45.118 | 15.59 | 18.89 | 16.287 |
| YMR174C | 0.253 | -1.983 | 3.29E-05 | 0.00035 | yes | down | 408.93 | 480.19 | 503.166 | 91.944 | 30.985 | 133.779 |
| YMR175W | 0.017 | -5.861 | 2.4E-38 | 3.87E-36 | yes | down | 835.115 | 2238.254 | 1708.998 | 12.048 | 8.414 | 16.42 |
| YMR189W | 0.258 | -1.956 | 1.07E-11 | 3.11E-10 | yes | down | 27.049 | 46.495 | 45.454 | 9.253 | 7.06 | 12.836 |
| YMR265C | 0.478 | -1.065 | 2.31E-05 | 0.000257 | yes | down | 231.974 | 142.353 | 134.957 | 76.513 | 78.845 | 87.025 |
| YMR271C | 0.433 | -1.206 | 1.29E-05 | 0.00015 | yes | down | 64.53 | 102.298 | 76.686 | 36.153 | 41.462 | 24.543 |
| YMR281W | 0.054 | -4.222 | 1.32E-11 | 3.79E-10 | yes | down | 1.313 | 9.961 | 5.598 | 0 | 0 | 0 |
| YMR297W | 0.422 | -1.243 | 8.75E-06 | 0.000105 | yes | down | 550.43 | 1098.595 | 686.356 | 362.851 | 343.828 | 250.732 |
| YMR317W | 0.251 | -1.994 | 1.15E-06 | 1.66E-05 | yes | down | 0.52 | 1.047 | 0.316 | 0.131 | 0.095 | 0.173 |
| YNL015W | 0.173 | -2.533 | 9.66E-08 | 1.71E-06 | yes | down | 254.409 | 149.531 | 264.494 | 21.974 | 26.48 | 28.879 |
| YNL018C | 0.455 | -1.135 | 2.66E-05 | 0.000291 | yes | down | 14.828 | 16.795 | 13.134 | 7.356 | 4.647 | 7.991 |
| YNL036W | 0.376 | -1.413 | 0.000239 | 0.001962 | yes | down | 96.23 | 45.325 | 98.394 | 19.796 | 18.455 | 44.525 |
| YNL046W | 0.482 | -1.054 | 0.009911 | 0.0445 | yes | down | 22.854 | 46.568 | 19.849 | 16.983 | 13.59 | 8.225 |
| YNL050C | 0.46 | -1.119 | 6.4E-07 | 9.98E-06 | yes | down | 48.47 | 88.618 | 63.928 | 31.527 | 32.168 | 26.141 |
| YNL052W | 0.377 | -1.406 | 8.37E-06 | 0.000102 | yes | down | 1585.619 | 3840.371 | 2763.732 | 1131.39 | 999.06 | 752.807 |
| YNL055C | 0.365 | -1.456 | 9.61E-06 | 0.000115 | yes | down | 1268.833 | 3037.675 | 2101.091 | 977.692 | 687.883 | 540.757 |
| YNL059C | 0.382 | -1.39 | 5.39E-06 | 6.8E-05 | yes | down | 3.037 | 2.647 | 2.937 | 0.944 | 1.23 | 1.018 |
| YNL071W | 0.185 | -2.433 | 1.51E-19 | 8.34E-18 | yes | down | 69.108 | 91.044 | 99.561 | 12.104 | 21.379 | 12.226 |
| YNL103W-A | 0.206 | -2.279 | 2.07E-38 | 3.44E-36 | yes | down | 462.645 | 491.752 | 453.569 | 106.796 | 86.813 | 93.865 |
| YNL115C | 0.366 | -1.452 | 3.99E-08 | 7.59E-07 | yes | down | 10.624 | 8.114 | 10.315 | 2.739 | 4.335 | 3.4 |
| YNL135C | 0.3 | -1.738 | 9.72E-10 | 2.38E-08 | yes | down | 1440.463 | 2955.768 | 1741.219 | 534.655 | 588.728 | 568.628 |
| YNL172W | 0.351 | -1.509 | 1.91E-09 | 4.44E-08 | yes | down | 2.563 | 3.411 | 3.511 | 1.075 | 1.249 | 0.967 |
| YNL195C | 0.46 | -1.121 | 0.000104 | 0.000954 | yes | down | 46.738 | 97.114 | 91.501 | 35.471 | 33.626 | 35.964 |
| YNL200C | 0.278 | -1.845 | 2.3E-08 | 4.61E-07 | yes | down | 122.102 | 313.395 | 214.017 | 62.035 | 63.485 | 40.392 |
| YNL206C | 0.48 | -1.06 | 2.29E-05 | 0.000256 | yes | down | 273.633 | 516.649 | 339.766 | 185.954 | 162.933 | 200.842 |
| YNL208W | 0.197 | -2.344 | 1.63E-15 | 6.61E-14 | yes | down | 5628.87 | 13713.11 | 8511.946 | 1677.803 | 1794.35 | 1564.98 |
| YNL220W | 0.376 | -1.41 | 4.3E-08 | 8.01E-07 | yes | down | 498.302 | 293.017 | 387.91 | 121.77 | 133.622 | 182.6 |
| YNL241C | 0.314 | -1.67 | 2.92E-06 | 3.92E-05 | yes | down | 354.979 | 1224.251 | 769.144 | 262.719 | 227.468 | 183.191 |
| YNL245C | 0.389 | -1.361 | 0.02774 | 0.09828 | yes | down | 125.759 | 36.004 | 59.586 | 2.131 | 0 | 0 |
| YNL276C | 0.362 | -1.466 | 0.02517 | 0.09057 | yes | down | 11.737 | 28.099 | 9.336 | 6.122 | 0 | 0 |
| YNL277W | 0.413 | -1.276 | 8.72E-08 | 1.56E-06 | yes | down | 55.018 | 67.907 | 79.208 | 31.499 | 22.155 | 28.625 |
| YNL332W | 0.313 | -1.677 | 0.000125 | 0.00112 | yes | down | 3.356 | 4.901 | 3.452 | 0.645 | 1.543 | 1.008 |
| YNL333W | 0.121 | -3.051 | 8.52E-37 | 1.23E-34 | yes | down | 186.459 | 139.164 | 148.279 | 12.936 | 19.874 | 21.489 |
| YNL334C | 0.465 | -1.104 | 0.000772 | 0.005446 | yes | down | 32.976 | 16.019 | 25.16 | 9.244 | 12.199 | 11.88 |
| YNR034W-A | 0.189 | -2.407 | 5.95E-17 | 2.78E-15 | yes | down | 772.901 | 1318.816 | 1100.573 | 178.963 | 215.619 | 138.024 |
| YNR059W | 0.482 | -1.053 | 0.000361 | 0.00283 | yes | down | 9.348 | 6.391 | 6.824 | 3.43 | 2.858 | 4.418 |
| YNR065C | 0.473 | -1.079 | 0.000126 | 0.001121 | yes | down | 5.563 | 3.903 | 3.353 | 1.757 | 2.101 | 2.158 |
| YOL052C-A | 0.393 | -1.346 | 0.00073 | 0.005199 | yes | down | 996.449 | 1421.532 | 845.86 | 261.271 | 443.068 | 396.024 |
| YOL055C | 0.473 | -1.08 | 0.001774 | 0.01084 | yes | down | 28.8 | 13.249 | 23.152 | 8.702 | 7.117 | 14.119 |
| YOL058W | 0.371 | -1.431 | 8.12E-11 | 2.15E-09 | yes | down | 1129.869 | 1097.055 | 1041.155 | 339.288 | 336.096 | 530.078 |
| YOL064C | 0.46 | -1.121 | 3.64E-12 | 1.11E-10 | yes | down | 168.201 | 149.014 | 150.474 | 69.063 | 76.98 | 70.931 |
| YOL077W-A | 0.443 | -1.173 | 5E-05 | 0.000504 | yes | down | 1685.369 | 2401.822 | 1724.475 | 601.138 | 950.14 | 802.971 |
| YOL091W | 0.346 | -1.531 | 5.62E-10 | 1.4E-08 | yes | down | 8.627 | 8.656 | 6.893 | 2.804 | 2.735 | 2.667 |
| YOL110W | 0.432 | -1.21 | 0.000346 | 0.002739 | yes | down | 378.38 | 1116.732 | 602.579 | 325.062 | 287.347 | 241.224 |
| YOL129W | 0.38 | -1.396 | 3.71E-07 | 6.05E-06 | yes | down | 352.444 | 700.472 | 453.539 | 195.497 | 198.11 | 153.996 |
| YOL147C | 0.423 | -1.241 | 2.95E-05 | 0.00032 | yes | down | 111.149 | 207.428 | 146.746 | 78.616 | 63.863 | 45.676 |
| YOL162W | 0.304 | -1.72 | 4.05E-07 | 6.6E-06 | yes | down | 19.397 | 30.155 | 21.323 | 8.235 | 6.814 | 4.591 |
| YOL164W | 0.499 | -1.002 | 3.09E-08 | 6.07E-07 | yes | down | 47.504 | 58.291 | 43.782 | 25.208 | 25.013 | 24.96 |
| YOR003W | 0.358 | -1.482 | 0.01717 | 0.0679 | yes | down | 0.447 | 12.313 | 3.659 | 0 | 0 | 0 |
| YOR064C | 0.042 | -4.559 | 3.55E-14 | 1.34E-12 | yes | down | 6.849 | 12.375 | 5.004 | 0 | 0 | 0 |
| YOR073W | 0.313 | -1.677 | 2.65E-05 | 0.000291 | yes | down | 2.918 | 4.494 | 2.631 | 1.131 | 1.088 | 0.55 |
| YOR122C | 0.399 | -1.327 | 4.53E-09 | 9.96E-08 | yes | down | 1328 | 2000.892 | 1337.06 | 553.909 | 662.179 | 563.64 |
| YOR126C | 0.457 | -1.13 | 3.22E-05 | 0.000345 | yes | down | 24.112 | 41.791 | 35.485 | 14.095 | 15.218 | 15.839 |
| YOR128C | 0.41 | -1.285 | 3.56E-06 | 4.69E-05 | yes | down | 43.227 | 67.095 | 73.6 | 18.086 | 23.329 | 32.778 |
| YOR154W | 0.49 | -1.029 | 0.000588 | 0.004332 | yes | down | 11.6 | 25.365 | 16.298 | 9.693 | 9.237 | 6.484 |
| YOR185C | 0.284 | -1.817 | 5.64E-15 | 2.21E-13 | yes | down | 71.041 | 60.815 | 56.303 | 16.572 | 20.471 | 14.893 |
| YOR202W | 0.29 | -1.786 | 1.96E-19 | 1.05E-17 | yes | down | 378.125 | 522.399 | 383.549 | 119.816 | 114.638 | 130.125 |
| YOR215C | 0.407 | -1.298 | 1.25E-05 | 0.000147 | yes | down | 31.581 | 25.451 | 29.066 | 11.216 | 13.732 | 8.612 |
| YOR222W | 0.2 | -2.319 | 2.44E-13 | 8.55E-12 | yes | down | 269.921 | 176.67 | 170.57 | 29.181 | 57.958 | 26.976 |
| YOR230W | 0.432 | -1.211 | 3.62E-07 | 5.92E-06 | yes | down | 361.29 | 463.394 | 433.858 | 223.276 | 171.251 | 140.345 |
| YOR271C | 0.337 | -1.568 | 1.04E-24 | 8.03E-23 | yes | down | 634.01 | 724.151 | 622.685 | 201.47 | 227.913 | 241.54 |
| YOR289W | 0.316 | -1.66 | 1.78E-11 | 5.05E-10 | yes | down | 104.328 | 152.671 | 117.017 | 43.5 | 41.093 | 29.653 |
| YOR350C | 0.261 | -1.939 | 3.47E-05 | 0.000365 | yes | down | 19.251 | 11.205 | 13.282 | 0.636 | 4.041 | 4.754 |
| YOR362C | 0.45 | -1.152 | 5.64E-06 | 0.000071 | yes | down | 438.487 | 848.563 | 586.024 | 286.086 | 292.012 | 247.393 |
| YOR391C | 0.31 | -1.688 | 0.000181 | 0.001537 | yes | down | 11.244 | 6.194 | 8.199 | 3.206 | 2.271 | 1.211 |
| YPL048W | 0.471 | -1.086 | 3.93E-05 | 0.00041 | yes | down | 980.636 | 1958.486 | 1115.824 | 641.619 | 642.881 | 589.415 |
| YPL109C | 0.476 | -1.071 | 0.001213 | 0.007894 | yes | down | 2.864 | 2.253 | 2.868 | 1.365 | 1.155 | 1.16 |
| YPL123C | 0.3 | -1.736 | 1.28E-05 | 0.000149 | yes | down | 3.347 | 2.635 | 3.57 | 0.851 | 1.013 | 0.702 |
| YPL149W | 0.345 | -1.534 | 1.23E-16 | 5.68E-15 | yes | down | 194.01 | 176.362 | 146.914 | 53.295 | 63.456 | 61.291 |
| YPL196W | 0.024 | -5.37 | 5.8E-20 | 3.28E-18 | yes | down | 4.459 | 22.004 | 15.478 | 0 | 0 | 0 |
| YPL245W | 0.421 | -1.247 | 3.43E-05 | 0.000362 | yes | down | 46.181 | 62.145 | 47.58 | 30.227 | 16.42 | 16.196 |
| YPL256C | 0.486 | -1.041 | 0.001211 | 0.007889 | yes | down | 6.065 | 7.314 | 5.222 | 3.524 | 1.959 | 3.237 |
| YPR028W | 0.429 | -1.221 | 7.03E-07 | 1.08E-05 | yes | down | 373.656 | 603.555 | 348.469 | 185.141 | 193.009 | 173.805 |
| YPR047W | 0.477 | -1.069 | 0.002262 | 0.01314 | yes | down | 3.383 | 5.061 | 4.055 | 1.449 | 2.555 | 1.741 |
| YPR058W | 0.377 | -1.408 | 5.44E-20 | 3.11E-18 | yes | down | 246.42 | 235.194 | 220.257 | 86.589 | 92.426 | 86.577 |
| YPR059C | 0.388 | -1.365 | 3.25E-05 | 0.000347 | yes | down | 77.352 | 61.775 | 63.354 | 24.189 | 32.471 | 16.023 |
| YPR138C | 0.378 | -1.404 | 2.42E-06 | 3.29E-05 | yes | down | 851.749 | 1554.466 | 1159.655 | 574.35 | 404.502 | 306.78 |
| YPR172W | 0.371 | -1.429 | 8.06E-06 | 9.82E-05 | yes | down | 30.523 | 57.687 | 37.235 | 17.553 | 12.322 | 13.6 |
| YPR191W | 0.436 | -1.196 | 0.000555 | 0.004115 | yes | down | 96.412 | 177.692 | 211.584 | 96.935 | 49.478 | 52.903 |
| YPR197C | 0.442 | -1.179 | 0.002518 | 0.01438 | yes | down | 35.074 | 18.445 | 20.393 | 13.515 | 6.464 | 9.732 |
| YPR198W | 0.331 | -1.596 | 1.3E-11 | 3.75E-10 | yes | down | 159.62 | 101.35 | 104.427 | 39.285 | 41.68 | 38.367 |

Resp genes early treatment

| Gene_id | FC(NaHS/Control) | Log2FC(NaHS/Control) | Pvalue | Padjust | Significant | Regulate | Control1_Fpkm | Control2_Fpkm | Control3_Fpkm | NaHS1_Fpkm | NaHS2_Fpkm | NaHS3_Fpkm |
| --- | --- | --- | --- | --- | --- | --- | --- | --- | --- | --- | --- | --- |
| YDL213C | 1.53 | 0.613938 | 0.016212 | 0.040785 | no | up | 31.48 | 24.68 | 22.5 | 38.51 | 30.42 | 49.55 |
| YDR353W | 1.314 | 0.394191 | 0.052833 | 0.107582 | no | up | 371.86 | 385.44 | 378.9 | 436.72 | 412.36 | 608.79 |
| YPL014W | 1.319 | 0.399498 | 0.044924 | 0.094103 | no | up | 31.53 | 29.7 | 21.9 | 35.62 | 33.83 | 38.11 |
| YKL096W-A | 0.649 | -0.62471 | 0.031585 | 0.070514 | no | down | 1118.63 | 1247.93 | 1405.99 | 534.37 | 591.88 | 1036.3 |
| YFL017C | 0.836 | -0.25901 | 0.215788 | 0.335113 | no | down | 97.75 | 84.93 | 79.21 | 67.76 | 58.01 | 81.56 |
| YGL062W | 1.201 | 0.26415 | 0.231711 | 0.353642 | no | up | 605.87 | 599.61 | 580.84 | 842.46 | 806.82 | 475.61 |
| YGL079W | 1.021 | 0.029283 | 0.898556 | 0.932569 | no | up | 33.47 | 37.41 | 30.67 | 41.35 | 26.16 | 32.85 |
| YMR244W | 1.105 | 0.144028 | 0.513024 | 0.634863 | no | up | 8.3 | 8.72 | 8.69 | 9.31 | 10.94 | 7.57 |
| YMR233W | 1.419 | 0.504709 | 0.049296 | 0.101548 | no | up | 11.19 | 9.08 | 8.66 | 15.78 | 14.16 | 10.64 |
| YDR246W-A | 1.25 | 0.322431 | 0.574781 | 0.689367 | no | up | 0 | 6.27 | 82.53 | 32.77 | 19.19 | 75.81 |
| YHL046W-A | 0.745 | -0.42405 | 0.338175 | 0.470392 | no | down | 12.05 | 28.63 | 40.39 | 11.99 | 19.07 | 22.96 |
| YJR003C | 1.799 | 0.847033 | 0.001515 | 0.005302 | no | up | 1.89 | 2.46 | 2.63 | 4.03 | 3.88 | 4.73 |
| YOR388C | 0.821 | -0.28405 | 0.354149 | 0.486112 | no | down | 4.69 | 4.28 | 5.15 | 4.7 | 4.18 | 2.3 |
| YBL043W | 0.774 | -0.36882 | 0.227697 | 0.348967 | no | down | 386.82 | 390.48 | 424.63 | 380.14 | 364.86 | 145.47 |
| YOR267C | 1.688 | 0.755066 | 6.82E-07 | 5.47E-06 | no | up | 57.45 | 45 | 53.18 | 92.2 | 81.38 | 84.92 |
| YDR096W | 1.382 | 0.466657 | 0.001529 | 0.00534 | no | up | 52.56 | 44.97 | 42.77 | 62.86 | 68.79 | 58.84 |
| YKL217W | 0.543 | -0.88134 | 0.002089 | 0.006969 | no | down | 3283.75 | 3098.81 | 3235.24 | 1577.13 | 2280.65 | 869.65 |
| YGR103W | 1.594 | 0.673077 | 0.009345 | 0.025413 | no | up | 3.48 | 4.31 | 4.05 | 7.7 | 4.63 | 6.56 |
| YLR089C | 0.526 | -0.92743 | 1.17E-08 | 1.33E-07 | no | down | 500.71 | 517.68 | 555.09 | 265.93 | 284.84 | 203.81 |
| YMR104C | 1 | 0.000271 | 0.99912 | 0.999435 | no | up | 137.2 | 139.12 | 146.08 | 155.3 | 173.07 | 86.05 |
| YCL026C-B | 0.876 | -0.19047 | 0.459128 | 0.585041 | no | down | 33.05 | 28.47 | 21.07 | 24 | 18.79 | 26.25 |
| YLR455W | 1.504 | 0.588827 | 0.01505 | 0.038238 | no | up | 12.41 | 7.47 | 9.67 | 13.52 | 15.3 | 15.06 |
| YHR126C | 1.727 | 0.787861 | 0.170197 | 0.279576 | no | up | 0.59 | 0.27 | 1.18 | 3.28 | 1.18 | 0.83 |
| YOR092W | 0.745 | -0.42533 | 0.00597 | 0.01723 | no | down | 21.77 | 22.86 | 21.66 | 17.8 | 14.7 | 15.41 |
| YGR158C | 1.319 | 0.399088 | 0.195498 | 0.310906 | no | up | 5.89 | 7.41 | 5.79 | 5.73 | 9.71 | 9.5 |
| YDR249C | 1.206 | 0.269836 | 0.371622 | 0.50324 | no | up | 37.04 | 37.92 | 28.25 | 52.26 | 49.46 | 22.1 |
| YGR292W | 1.206 | 0.270042 | 0.275652 | 0.402847 | no | up | 13.54 | 8.04 | 11.08 | 12.73 | 10.91 | 14.99 |
| YGR181W | 0.939 | -0.09056 | 0.579786 | 0.694453 | no | down | 216.8 | 213.84 | 216.87 | 204.77 | 188.81 | 173.26 |
| YIL162W | 1.496 | 0.581565 | 0.036706 | 0.080059 | no | up | 87.97 | 91.37 | 84.67 | 169.08 | 155.54 | 73.52 |
| YGL176C | 1.405 | 0.490539 | 0.024604 | 0.057203 | no | up | 12.86 | 10.95 | 10.66 | 16.58 | 19.46 | 11.96 |
| YGL174W | 1.185 | 0.245345 | 0.271566 | 0.398435 | no | up | 12.16 | 13.97 | 11.07 | 15.45 | 15.76 | 12.03 |
| YPR130C | 1.116 | 0.157819 | 0.677121 | 0.774917 | no | up | 11.38 | 17.56 | 8.24 | 13.81 | 10.41 | 15.65 |
| YKR061W | 0.921 | -0.11811 | 0.531934 | 0.652668 | no | down | 19.45 | 17.98 | 22.84 | 16.69 | 17.02 | 20.06 |
| YDR360W | 0.976 | -0.03468 | 0.937999 | 0.959774 | no | down | 14.21 | 11.82 | 10.63 | 21.54 | 7.78 | 5.2 |
| YOR105W | 1.717 | 0.779584 | 0.04931 | 0.101548 | no | up | 12.97 | 9.4 | 7.78 | 15.48 | 19.79 | 18.44 |
| YJL213W | 1.573 | 0.653722 | 0.004766 | 0.014296 | no | up | 43.04 | 35.1 | 32.58 | 47.94 | 51.46 | 72.43 |
| YJL012C | 1.474 | 0.560022 | 0.000187 | 0.00084 | no | up | 22.64 | 21.61 | 23.53 | 33.66 | 36.53 | 28.14 |
| YNL053W | 1.045 | 0.063608 | 0.790783 | 0.857894 | no | up | 111.37 | 115.07 | 114.76 | 149.05 | 125.57 | 75.32 |
| YGL255W | 1.84 | 0.879939 | 0.041762 | 0.08868 | no | up | 351.66 | 419.51 | 547.94 | 1506.44 | 970.85 | 223.12 |
| YNL125C | 0.957 | -0.06414 | 0.614394 | 0.722944 | no | down | 421.25 | 416.96 | 415.16 | 400.67 | 427.1 | 343.04 |
| YMR058W | 0.917 | -0.12566 | 0.7157 | 0.802996 | no | down | 82.56 | 95.28 | 124.8 | 124.38 | 106.44 | 39.23 |
| YMR166C | 0.864 | -0.21086 | 0.613936 | 0.722941 | no | down | 21.33 | 22.58 | 20.14 | 25.39 | 22.95 | 4.69 |
| YLR142W | 1.71 | 0.773767 | 0.020414 | 0.04906 | no | up | 127.2 | 115.67 | 92.55 | 236.84 | 268.91 | 89.16 |
| YDL055C | 1.002 | 0.002784 | 0.990294 | 0.99421 | no | up | 65.45 | 82.44 | 109.62 | 79.31 | 76.82 | 94.19 |
| YNR004W | 0.854 | -0.22709 | 0.368833 | 0.500606 | no | down | 45.71 | 45.84 | 53.31 | 39.8 | 50.21 | 27.75 |
| YGL008C | 1.322 | 0.402711 | 0.138797 | 0.238093 | no | up | 720.1 | 651.82 | 726.22 | 1091.07 | 1015.82 | 912.84 |
| YPL005W | 1.764 | 0.818823 | 1.63E-09 | 2.16E-08 | no | up | 32.03 | 27.38 | 30.3 | 53.76 | 54.22 | 47.65 |
| YAL034W-A | 1.087 | 0.120157 | 0.797709 | 0.863119 | no | up | 2.64 | 1.8 | 3.82 | 1 | 3.28 | 4.53 |
| YNL144C | 1.525 | 0.608705 | 3.46E-07 | 2.91E-06 | no | up | 63.72 | 63.86 | 67.25 | 103.9 | 98.17 | 89.41 |
| YGR042W | 1.047 | 0.065689 | 0.696858 | 0.78939 | no | up | 27.61 | 26.11 | 24.54 | 26.57 | 25.4 | 27.48 |
| YLR126C | 1.193 | 0.254833 | 0.387834 | 0.519474 | no | up | 12.23 | 12.76 | 18.35 | 20.9 | 19.3 | 10.83 |
| YGR169C | 0.663 | -0.59381 | 0.064943 | 0.127259 | no | down | 26.82 | 28.22 | 22.72 | 22.05 | 18.76 | 7.87 |
| YPL144W | 0.959 | -0.05983 | 0.793563 | 0.859511 | no | down | 33.64 | 28.7 | 29.99 | 32.35 | 28.82 | 23.4 |
| YLR069C | 1.058 | 0.08175 | 0.717139 | 0.80417 | no | up | 13.15 | 12.05 | 10.46 | 13.68 | 14.59 | 8.82 |
| YDR309C | 1.625 | 0.700739 | 0.001638 | 0.005645 | no | up | 20.32 | 20.91 | 26.18 | 38.04 | 44.06 | 26.96 |
| YER095W | 1.008 | 0.01161 | 0.949858 | 0.968026 | no | up | 1123.69 | 1094 | 1059.79 | 930.22 | 986.45 | 1288.66 |
| YBR054W | 0.906 | -0.14191 | 0.65621 | 0.758542 | no | down | 479.96 | 429.41 | 603.12 | 558.21 | 558.96 | 212.95 |
| YIL002W-A | 1.822 | 0.865761 | 0.000904 | 0.003374 | no | up | 317.79 | 297.24 | 262.27 | 381.62 | 534.28 | 510.65 |
| YPL111W | 1.019 | 0.026592 | 0.950074 | 0.968026 | no | up | 440.18 | 495.56 | 468.6 | 659.12 | 642.02 | 111.31 |
| YDR264C | 1.246 | 0.316983 | 0.096713 | 0.177563 | no | up | 46.09 | 45.95 | 43.86 | 58.04 | 66.79 | 42.05 |
| YDR055W | 1.059 | 0.082056 | 0.817038 | 0.877006 | no | up | 111.08 | 103.91 | 171.84 | 181.59 | 164.76 | 57.96 |
| YAL056C-A | 0.532 | -0.90954 | 0.012446 | 0.032602 | no | down | 21.4 | 20.21 | 24.25 | 7.23 | 12.84 | 10.6 |
| YNL024C | 1.393 | 0.478606 | 0.307081 | 0.438283 | no | up | 5.63 | 2.66 | 2.21 | 2.1 | 4.97 | 8.18 |
| YKR093W | 0.664 | -0.58981 | 0.065612 | 0.128333 | no | down | 15.02 | 16.85 | 12.82 | 11.77 | 11.77 | 4.6 |
| YDR342C | 1.833 | 0.87429 | 0.002045 | 0.006854 | no | up | 5044.7 | 5098 | 4914.9 | 14679.71 | 11661.97 | 5210.94 |
| YGR023W | 0.8 | -0.32224 | 0.238142 | 0.360823 | no | down | 370.92 | 340.38 | 385.34 | 342.06 | 343.95 | 165.19 |
| YMR290W-A | 0.917 | -0.12453 | 0.507949 | 0.630417 | no | down | 32.66 | 28.81 | 33.79 | 29.76 | 27.75 | 28.66 |
| YIL131C | 0.582 | -0.78057 | 0.001694 | 0.005811 | no | down | 8.91 | 8.49 | 9.88 | 4.44 | 4.11 | 6.41 |
| YDR068W | 1.358 | 0.441254 | 0.189657 | 0.303671 | no | up | 7.19 | 8.18 | 5.85 | 7.54 | 6.85 | 14.29 |
| YML053C | 1.183 | 0.242992 | 0.322417 | 0.454368 | no | up | 16.16 | 17.73 | 12.18 | 18.5 | 20.59 | 18.16 |
| YDR270W | 1.13 | 0.176496 | 0.531128 | 0.652197 | no | up | 23.34 | 19.41 | 19.77 | 26.31 | 30.55 | 13.13 |
| YHR030C | 1.468 | 0.553699 | 0.000641 | 0.002489 | no | up | 47.02 | 48.16 | 47.66 | 73.12 | 77.33 | 56.01 |
| YGR152C | 1.846 | 0.884111 | 0.065733 | 0.12849 | no | up | 0.5 | 0.89 | 2.08 | 2.21 | 2.05 | 2.96 |
| YGR273C | 1.097 | 0.133758 | 0.79242 | 0.858568 | no | up | 2.19 | 3.85 | 1.48 | 3.25 | 3.94 | 1.15 |
| YER137W-A | 1.984 | 0.988571 | 0.004746 | 0.014255 | no | up | 29.59 | 22.56 | 26.45 | 43.45 | 39.11 | 72.07 |
| YDR475C | 1.497 | 0.581881 | 0.000167 | 0.000758 | no | up | 63.07 | 58.93 | 58.47 | 97.13 | 96.01 | 73.16 |
| YGL179C | 0.769 | -0.37812 | 0.10579 | 0.191047 | no | down | 11.33 | 12.76 | 9.48 | 8.24 | 10.36 | 6.42 |
| YMR307W | 1.01 | 0.014728 | 0.964001 | 0.976619 | no | up | 97.81 | 97.59 | 126.35 | 152.83 | 116 | 50.56 |
| YDL182W | 1.316 | 0.395922 | 0.004896 | 0.014648 | no | up | 1239.88 | 1261.91 | 1272.7 | 1704.14 | 1791.31 | 1371.08 |
| YPR079W | 0.852 | -0.23157 | 0.304009 | 0.43517 | no | down | 69.1 | 63.69 | 57.7 | 60.36 | 61.23 | 35.97 |
| YJR148W | 0.65 | -0.62162 | 4.47E-05 | 0.000235 | no | down | 1243.02 | 1095.18 | 1088.97 | 691.17 | 830.83 | 631.39 |
| YHR159W | 0.625 | -0.67754 | 0.006624 | 0.018794 | no | down | 21.95 | 21.13 | 17.59 | 13.72 | 14.64 | 7.93 |
| YIL120W | 1.061 | 0.085699 | 0.651587 | 0.7544 | no | up | 15.99 | 14.88 | 11.54 | 15.56 | 14.77 | 13.69 |
| YPR156C | 0.913 | -0.13073 | 0.512816 | 0.634863 | no | down | 201.7 | 183.15 | 201.71 | 158.4 | 146.12 | 214.2 |
| YAR066W | 0.858 | -0.22125 | 0.589079 | 0.702398 | no | down | 5.14 | 4.52 | 6.9 | 6.37 | 4.95 | 2.14 |
| YPR027C | 0.636 | -0.65381 | 0.000428 | 0.00174 | no | down | 85.53 | 84.09 | 67.4 | 56.08 | 46.45 | 42.14 |
| YCR026C | 1.219 | 0.285692 | 0.094794 | 0.174596 | no | up | 26.73 | 22.96 | 24.49 | 34.29 | 29.98 | 24.54 |
| YOR383C | 0.562 | -0.83072 | 0.006906 | 0.019498 | no | down | 1463.03 | 1644.03 | 1956.92 | 1245.88 | 941.89 | 458.62 |
| YFL051C | 1.239 | 0.309458 | 0.169963 | 0.279398 | no | up | 65.27 | 57.94 | 54.27 | 76.32 | 54.08 | 81.34 |
| YBL005W-B | 1.508 | 0.592701 | 0.075842 | 0.145237 | no | up | 1.96 | 1.54 | 1.84 | 1.75 | 2.17 | 4.25 |
| YDR222W | 1.222 | 0.289113 | 0.04297 | 0.090698 | no | up | 29.06 | 30.08 | 27.05 | 34.11 | 36.29 | 32.56 |
| YNL283C | 0.858 | -0.22033 | 0.162304 | 0.269999 | no | down | 44.78 | 38.81 | 44.59 | 37.46 | 39.28 | 30.31 |
| YOL032W | 1.419 | 0.504904 | 0.063008 | 0.124159 | no | up | 64.11 | 53 | 47.49 | 60.42 | 62.71 | 106.41 |
| YHR094C | 1.804 | 0.850858 | 0.013635 | 0.03517 | no | up | 7.37 | 11.02 | 11.09 | 13.27 | 12.38 | 29.17 |
| YBL070C | 1.129 | 0.174477 | 0.621985 | 0.729576 | no | up | 27.47 | 26.3 | 19.5 | 27.63 | 33.35 | 17.2 |
| YML123C | 1.258 | 0.331408 | 0.220805 | 0.340903 | no | up | 10.89 | 10.81 | 12.77 | 18.72 | 15.63 | 8.78 |
| YBL069W | 1.169 | 0.225197 | 0.446694 | 0.574735 | no | up | 38.36 | 36.02 | 37.84 | 55.95 | 51.6 | 22.57 |
| YLR110C | 1.029 | 0.040769 | 0.822992 | 0.880788 | no | up | 7357.97 | 8779.57 | 9377.21 | 8915.15 | 7264.82 | 8771.72 |
| YFL020C | 0.775 | -0.3672 | 0.26594 | 0.392175 | no | down | 36.17 | 31.52 | 41.73 | 27.96 | 35.83 | 14.87 |
| YOR104W | 1.468 | 0.553645 | 0.034986 | 0.076862 | no | up | 14.71 | 10.99 | 13.83 | 23.57 | 21.05 | 12.96 |
| YOR178C | 0.744 | -0.42659 | 0.082053 | 0.155183 | no | down | 103.24 | 93.05 | 122.47 | 94.09 | 85.44 | 49.51 |
| YER188W | 1 | 0 | 1 | 1 | no | no change | 0 | 0 | 0 | 0 | 0 | 0 |
| YKR013W | 0.723 | -0.46784 | 0.000934 | 0.00347 | no | down | 146.96 | 148.24 | 151.89 | 98.81 | 100.01 | 113.25 |
| YLL028W | 1.016 | 0.023006 | 0.878838 | 0.918943 | no | up | 32.4 | 31.84 | 35.4 | 29.56 | 34.38 | 34.66 |
| YHL008C | 1.345 | 0.427301 | 0.117908 | 0.208573 | no | up | 80 | 67.26 | 69.43 | 117.2 | 118.82 | 55.28 |
| YDR247W | 1.372 | 0.456376 | 0.00019 | 0.000852 | no | up | 123.63 | 113.43 | 117.78 | 160.02 | 152.47 | 163 |
| YMR215W | 0.783 | -0.35222 | 0.156515 | 0.262458 | no | down | 16.19 | 16.71 | 14.09 | 15.74 | 11.62 | 8.24 |
| YLR113W | 1.019 | 0.026484 | 0.850198 | 0.900869 | no | up | 160.23 | 147.6 | 150.59 | 165.73 | 159.48 | 130.34 |
| YLR176C | 1.914 | 0.93656 | 5.38E-05 | 0.000275 | no | up | 7.23 | 9.55 | 8.66 | 19.46 | 17.96 | 11.72 |
| YKL167C | 1.303 | 0.381998 | 0.025734 | 0.059417 | no | up | 265.9 | 249.65 | 222.71 | 281.32 | 292.96 | 342.97 |
| YOR389W | 0.946 | -0.08057 | 0.606548 | 0.716367 | no | down | 16.56 | 18.91 | 20.01 | 17.64 | 17.88 | 15.68 |
| YER145C | 0.723 | -0.4677 | 0.242451 | 0.365692 | no | down | 186.84 | 166.34 | 214.91 | 165.85 | 180.12 | 37.31 |
| YGR022C | 0.898 | -0.15538 | 0.679894 | 0.77697 | no | down | 139.02 | 80.56 | 141.26 | 139.2 | 120.13 | 41.02 |
| YLR057W | 0.853 | -0.23004 | 0.448979 | 0.576739 | no | down | 1.77 | 1.81 | 1.85 | 1.4 | 1.06 | 1.97 |
| YGR225W | 1.145 | 0.195448 | 0.526017 | 0.6479 | no | up | 1.48 | 2.02 | 1.85 | 2.11 | 2.48 | 1.51 |
| YPR137C-B | 0.888 | -0.17104 | 0.425216 | 0.554521 | no | down | 5.2 | 4.36 | 4.74 | 3.98 | 3.33 | 4.98 |
| YHL047C | 0.933 | -0.10073 | 0.549704 | 0.669154 | no | down | 36.27 | 35.61 | 45.83 | 37.9 | 32.87 | 35.94 |
| YER045C | 1.03 | 0.042738 | 0.848977 | 0.900026 | no | up | 16.58 | 12.71 | 17.63 | 15.86 | 19.18 | 12.2 |
| YOR071C | 0.774 | -0.3687 | 0.149522 | 0.252667 | no | down | 22.58 | 27.41 | 28.45 | 22.09 | 24.29 | 12.39 |
| YMR180C | 0.979 | -0.03031 | 0.847469 | 0.898878 | no | down | 35.6 | 36.12 | 41.03 | 35.33 | 34.24 | 37.49 |
| YLR214W | 1.208 | 0.2729 | 0.368889 | 0.500606 | no | up | 5.98 | 6.16 | 6.23 | 9.43 | 8.8 | 3.92 |
| YLR257W | 1.23 | 0.298838 | 0.132056 | 0.22874 | no | up | 141.17 | 133.64 | 130.41 | 141.12 | 144.64 | 199.75 |
| YPR175W | 1.546 | 0.628406 | 0.004619 | 0.013922 | no | up | 4.56 | 3.54 | 5.42 | 6.41 | 7.64 | 6.67 |
| YDR423C | 1.694 | 0.76035 | 2.04E-07 | 1.78E-06 | no | up | 119.71 | 110.44 | 112.47 | 177.11 | 182.76 | 208.83 |
| YDR422C | 0.854 | -0.22833 | 0.446629 | 0.574735 | no | down | 23.62 | 23.21 | 21.68 | 20.1 | 26.69 | 10 |
| YDR420W | 1.037 | 0.05303 | 0.806795 | 0.869688 | no | up | 5.54 | 4.29 | 6.1 | 5.61 | 6.38 | 4.21 |
| YKR010C | 0.8 | -0.32117 | 0.29487 | 0.424386 | no | down | 4.49 | 3.93 | 3.24 | 4.38 | 2.56 | 2.04 |
| YCL059C | 1.95 | 0.963565 | 1.26E-07 | 1.15E-06 | no | up | 10.27 | 12.13 | 11.69 | 22.52 | 21.86 | 21.38 |
| YML046W | 1.137 | 0.185828 | 0.582675 | 0.697386 | no | up | 1.98 | 1.02 | 1.32 | 1.76 | 1.78 | 1.32 |
| YDL174C | 0.617 | -0.69766 | 0.010659 | 0.028582 | no | down | 670.95 | 683.68 | 613.28 | 463.4 | 475.43 | 216.56 |
| YEL059C-A | 0.841 | -0.2492 | 0.38387 | 0.515755 | no | down | 642.17 | 766.82 | 661.5 | 620.24 | 621.68 | 303.71 |
| YMR011W | 1.066 | 0.091734 | 0.82466 | 0.88161 | no | up | 177.99 | 235.33 | 146.5 | 104.37 | 103.75 | 372.66 |
| YMR103C | 1.287 | 0.3641 | 0.02534 | 0.058698 | no | up | 316.64 | 303.38 | 328.02 | 350.56 | 378.4 | 423.88 |
| YKL131W | 1.039 | 0.054931 | 0.884999 | 0.923104 | no | up | 18.32 | 14.42 | 16.79 | 24.95 | 17.98 | 6.9 |
| YER053C | 0.788 | -0.34302 | 0.100069 | 0.182769 | no | down | 1080.03 | 930.7 | 981.16 | 681.56 | 630.84 | 947.75 |
| YJR160C | 0.955 | -0.06626 | 0.79054 | 0.857894 | no | down | 15.83 | 13.76 | 14.31 | 17.69 | 14.31 | 8.97 |
| YLL029W | 1.426 | 0.511783 | 8.66E-06 | 5.38E-05 | no | up | 330.52 | 310.73 | 318.21 | 436.04 | 441.51 | 460.98 |
| YPR119W | 1.136 | 0.183859 | 0.748704 | 0.827149 | no | up | 0.05 | 0.3 | 0.22 | 0.39 | 0.11 | 0.21 |
| YDL062W | 1 | 0 | 1 | 1 | no | no change | 0 | 2.71 | 0 | 1.55 | 0.38 | 0.36 |
| YDR306C | 1.174 | 0.23127 | 0.21616 | 0.335363 | no | up | 51.08 | 51.19 | 43.08 | 48.45 | 53.82 | 64.34 |
| YDL179W | 1.538 | 0.621123 | 0.055195 | 0.111496 | no | up | 3.64 | 3.83 | 3.46 | 4.54 | 4.69 | 7.67 |
| YPR098C | 0.76 | -0.39547 | 0.001485 | 0.005213 | no | down | 894.6 | 883.4 | 925.85 | 624.41 | 659.1 | 678.33 |
| YIL014C-A | 0.586 | -0.7703 | 0.005262 | 0.015554 | no | down | 127.53 | 102.11 | 139 | 44.24 | 79.17 | 70 |
| YLL027W | 0.9 | -0.15256 | 0.33811 | 0.470392 | no | down | 1358.29 | 1368.99 | 1420.78 | 1111.92 | 1114.8 | 1371.91 |
| YBL095W | 0.78 | -0.35935 | 0.105991 | 0.191128 | no | down | 900.75 | 919.86 | 883.06 | 744.23 | 833.2 | 456.75 |
| YPL171C | 1.2 | 0.263538 | 0.492064 | 0.615536 | no | up | 52.13 | 40.87 | 62.7 | 96.43 | 69.31 | 22.5 |
| YHR051W | 1.997 | 0.997658 | 1.52E-13 | 3.51E-12 | no | up | 1021.55 | 942.33 | 1055.97 | 1831.62 | 1913.92 | 2056.39 |
| YKL007W | 0.69 | -0.53597 | 7.36E-05 | 0.000363 | no | down | 129.97 | 133.76 | 118.49 | 83.82 | 85.35 | 85.15 |
| YDL003W | 0.807 | -0.30964 | 0.281095 | 0.408263 | no | down | 5.37 | 6.92 | 7.27 | 5.86 | 6.26 | 3.13 |
| YMR315W | 0.628 | -0.67064 | 6.46E-07 | 5.21E-06 | no | down | 228.15 | 207.69 | 207.13 | 123.38 | 128.52 | 137.74 |
| YER061C | 1.516 | 0.600501 | 0.000124 | 0.000581 | no | up | 71.99 | 67.3 | 72.98 | 114.16 | 115.65 | 86.8 |
| YDR072C | 0.749 | -0.4179 | 0.060278 | 0.120007 | no | down | 247.51 | 254.55 | 244.4 | 202.77 | 217.12 | 121.12 |
| YER011W | 0.782 | -0.35555 | 0.106528 | 0.19181 | no | down | 17.25 | 13.51 | 16.32 | 12.93 | 12.38 | 10.02 |
| YMR008C | 0.604 | -0.7266 | 4.58E-10 | 6.69E-09 | no | down | 274.73 | 262.04 | 269.57 | 163.83 | 166.87 | 142.68 |
| YOR184W | 0.726 | -0.46152 | 0.002831 | 0.009106 | no | down | 350.56 | 354.31 | 319.65 | 218.77 | 233.46 | 266.34 |
| YIL034C | 0.757 | -0.40127 | 0.004177 | 0.012757 | no | down | 454.14 | 395.8 | 412.61 | 319.23 | 337.88 | 268.04 |
| YDL243C | 0.686 | -0.54352 | 0.000233 | 0.001017 | no | down | 63.54 | 72.78 | 65.51 | 47.36 | 43.07 | 43.33 |
| YDR379C-A | 1.511 | 0.595532 | 0.0029 | 0.009314 | no | up | 2342.12 | 2204.41 | 2285.06 | 2786.17 | 2778.37 | 3746.72 |
| YBR256C | 1.393 | 0.478635 | 0.001136 | 0.00413 | no | up | 2314.82 | 2383.82 | 2170.89 | 3012.67 | 2882.94 | 3407.7 |
| YDR223W | 0.676 | -0.56583 | 0.000404 | 0.00165 | no | down | 42.01 | 35.41 | 37.22 | 23.13 | 24.8 | 26.78 |
| YNL104C | 0.503 | -0.99182 | 8.41E-10 | 1.17E-08 | no | down | 5391.84 | 5147.02 | 5334.26 | 2626.4 | 2990.94 | 2078.8 |
| YOR173W | 0.753 | -0.40917 | 0.063785 | 0.125377 | no | down | 1706.22 | 1630.02 | 1524.41 | 939.37 | 1055.78 | 1508.01 |
| SRG1 | 1.276 | 0.351825 | 0.224943 | 0.345944 | no | up | 664.92 | 616.65 | 647.57 | 1014.84 | 993.09 | 422.13 |
| YBR092C | 0.556 | -0.8466 | 0.000402 | 0.001645 | no | down | 7.78 | 8.54 | 10.55 | 3.87 | 5.47 | 4.81 |
| YKL107W | 0.57 | -0.8119 | 8.2E-05 | 0.0004 | no | down | 102.54 | 87.34 | 79.77 | 39.37 | 59.54 | 47.46 |
| YOL083W | 0.768 | -0.38025 | 0.031229 | 0.069841 | no | down | 77.03 | 78.01 | 78.09 | 52.6 | 52.3 | 67.77 |
| YLR307C-A | 0.664 | -0.59039 | 0.278346 | 0.405569 | no | down | 3410.38 | 3140.4 | 3055.17 | 2149.97 | 2418.96 | 258.02 |
| YGL125W | 0.556 | -0.84812 | 0.00052 | 0.00206 | no | down | 174.38 | 162.26 | 184.34 | 105.59 | 112.44 | 57.54 |
| YBL097W | 0.819 | -0.28891 | 0.288127 | 0.416571 | no | down | 2.32 | 2.95 | 2.28 | 2.42 | 1.62 | 1.93 |
| YDR098C-B | 0.973 | -0.03962 | 0.84155 | 0.894571 | no | down | 18.34 | 18.04 | 19.9 | 15.84 | 15.43 | 18.92 |
| YNL286W | 1.101 | 0.138727 | 0.357566 | 0.489581 | no | up | 40.5 | 34.35 | 40.15 | 35.77 | 44.56 | 49.84 |
| NME1 | 0.693 | -0.52872 | 0.002363 | 0.007758 | no | down | 171.21 | 172.88 | 167.44 | 102.72 | 115.88 | 110.82 |
| Q0045 | 1.308 | 0.387825 | 0.502414 | 0.625137 | no | up | 0.14 | 0.41 | 0 | 0.46 | 0.4 | 0.09 |
| RDN18-1 | 1.836 | 0.876467 | 0.127428 | 0.22201 | no | up | 240.18 | 981.3 | 289.76 | 923.44 | 503.41 | 2994.04 |
| RDN18-2 | 1.836 | 0.876527 | 0.127405 | 0.22201 | no | up | 240.18 | 981.3 | 289.76 | 923.44 | 503.41 | 2994.71 |
| RDN58-1 | 1 | 0 | 1 | 1 | no | no change | 0 | 0 | 0 | 0 | 0 | 0 |
| RDN58-2 | 1 | 0 | 1 | 1 | no | no change | 0 | 0 | 0 | 0 | 0 | 0 |
| SCR1 | 1 | 0 | 1 | 1 | no | no change | 0 | 0 | 0.01 | 0 | 0.53 | 0.53 |
| snR17a | 1.855 | 0.89106 | 0.000118 | 0.000557 | no | up | 408.55 | 396.63 | 305.72 | 635.32 | 523.56 | 809.18 |
| snR19 | 1.423 | 0.508624 | 0.002754 | 0.008882 | no | up | 252.36 | 247.01 | 230.25 | 316.95 | 303.05 | 385.12 |
| snR190 | 1.455 | 0.541087 | 0.276521 | 0.403745 | no | up | 54.81 | 88.09 | 48.28 | 94.97 | 66.1 | 92.24 |
| tR(UCU)M2 | 1 | 0 | 1 | 1 | no | no change | 0 | 0 | 0 | 0 | 0 | 0 |
| YAL028W | 0.708 | -0.49791 | 0.050093 | 0.102861 | no | down | 45.99 | 42.85 | 36.87 | 30.9 | 36.62 | 18.15 |
| YAL044C | 0.852 | -0.23131 | 0.415575 | 0.546778 | no | down | 1268.16 | 1266.16 | 1353.03 | 1361.94 | 1241.61 | 566.56 |
| YAL064W | 1 | 0 | 1 | 1 | no | no change | 0 | 0 | 3.87 | 0 | 1.27 | 0 |
| YAR015W | 0.642 | -0.64006 | 0.03603 | 0.078937 | no | down | 141.61 | 151.22 | 159.46 | 113.25 | 115.63 | 45.15 |
| YAR027W | 1.044 | 0.062034 | 0.750146 | 0.827887 | no | up | 189.36 | 200.33 | 171.3 | 168.84 | 170 | 226.99 |
| YAR075W | 0.838 | -0.25459 | 0.648564 | 0.752685 | no | down | 2.41 | 1.67 | 5.49 | 4.01 | 2.74 | 0.28 |
| YBL001C | 0.817 | -0.29196 | 0.04954 | 0.101889 | no | down | 2130.46 | 1915.44 | 2084.1 | 1415.84 | 1530.95 | 1681.69 |
| YBL015W | 0.659 | -0.60255 | 0.033741 | 0.074644 | no | down | 5891.76 | 5896.19 | 5337.51 | 4173.88 | 4645.3 | 1938.85 |
| YBL029W | 0.709 | -0.49544 | 0.023729 | 0.055697 | no | down | 103.91 | 110.33 | 117.45 | 87.02 | 87.99 | 51.94 |
| YBL085W | 1.128 | 0.173293 | 0.425256 | 0.554521 | no | up | 6.85 | 5.25 | 7.83 | 6.34 | 7.48 | 8.14 |
| YBR005W | 0.898 | -0.15575 | 0.202487 | 0.319777 | no | down | 1212.72 | 1193.21 | 1134.14 | 1041.02 | 1106.78 | 923.07 |
| YBR007C | 0.519 | -0.94577 | 5.75E-07 | 4.66E-06 | no | down | 30.92 | 34.93 | 28.78 | 15.64 | 18.78 | 12.8 |
| YBR011C | 0.892 | -0.16512 | 0.321811 | 0.453796 | no | down | 1597.83 | 1568.22 | 1693.92 | 1312.02 | 1255.11 | 1616.45 |
| YBR016W | 0.926 | -0.11135 | 0.448448 | 0.57629 | no | down | 2400.08 | 2304.47 | 2102.62 | 2061.75 | 2224.68 | 1684.46 |
| YBR043C | 0.731 | -0.4519 | 0.017393 | 0.043224 | no | down | 61.73 | 61.68 | 68.38 | 48.22 | 53.54 | 34.26 |
| YBR044C | 0.899 | -0.15312 | 0.406516 | 0.538093 | no | down | 35.5 | 37.9 | 37.79 | 37.93 | 34.44 | 25.17 |
| YBR053C | 1.034 | 0.048059 | 0.822369 | 0.880495 | no | up | 405.22 | 384.31 | 366.26 | 320.61 | 344.42 | 493.14 |
| YBR056W | 0.764 | -0.38928 | 0.121854 | 0.214478 | no | down | 107.27 | 108.85 | 104.09 | 60.21 | 65.6 | 107.69 |
| YBR056W-A | 1.133 | 0.180741 | 0.590826 | 0.703664 | no | up | 550.6 | 422.85 | 286.88 | 471.92 | 255.15 | 483.32 |
| YBR068C | 0.782 | -0.35426 | 0.25802 | 0.382898 | no | down | 2556.56 | 2686.98 | 2540.53 | 2312.94 | 2620.83 | 927.07 |
| YBR072W | 1.231 | 0.29989 | 0.125138 | 0.219044 | no | up | 6144.39 | 5388.77 | 6570.35 | 8110.58 | 8266.43 | 5364.7 |
| YBR111C | 1.146 | 0.196365 | 0.135151 | 0.232783 | no | up | 612.57 | 550.76 | 529.46 | 599.46 | 629.86 | 648.52 |
| YBR145W | 0.642 | -0.63888 | 1.01E-07 | 9.34E-07 | no | down | 469.97 | 492.94 | 468.03 | 315.26 | 304.26 | 270.91 |
| YBR149W | 0.82 | -0.28705 | 0.211372 | 0.329901 | no | down | 732.26 | 684.07 | 723.85 | 448.74 | 490.11 | 742.49 |
| YBR174C | 0.591 | -0.75881 | 0.082641 | 0.156107 | no | down | 23.38 | 13.87 | 14.67 | 11.02 | 9.26 | 5.38 |
| YBR230C | 0.51 | -0.97205 | 1.81E-06 | 1.3E-05 | no | down | 9820.71 | 9665.52 | 9140.84 | 4831.29 | 5546.94 | 3242.15 |
| YBR230W-A | 1.802 | 0.849675 | 0.000198 | 0.000883 | no | up | 1783.64 | 1440.97 | 1691.84 | 2182.63 | 2315.8 | 3172.21 |
| YBR240C | 0.608 | -0.71745 | 0.004417 | 0.013387 | no | down | 21.96 | 19.34 | 19.5 | 12.13 | 15.43 | 7.7 |
| YBR248C | 1.375 | 0.459804 | 0.161279 | 0.268672 | no | up | 19.78 | 23.1 | 29.85 | 24.15 | 23.39 | 52.08 |
| YBR284W | 1.16 | 0.213681 | 0.444801 | 0.572996 | no | up | 14.57 | 12.43 | 9.65 | 11.2 | 11.29 | 19.16 |
| YBR286W | 0.638 | -0.6488 | 0.001646 | 0.005668 | no | down | 2633.72 | 2697.55 | 2572.15 | 1355.36 | 1434.53 | 2029.47 |
| YCL007C | 0.924 | -0.11345 | 0.771076 | 0.844088 | no | down | 10.9 | 18.47 | 13.63 | 12.77 | 7.93 | 16.53 |
| YCL009C | 0.71 | -0.49422 | 0.003012 | 0.009636 | no | down | 3140.33 | 3202.41 | 3260.37 | 2429.21 | 2435.31 | 1729.17 |
| YCL014W | 0.662 | -0.59482 | 0.029536 | 0.066643 | no | down | 7.28 | 7.47 | 7.03 | 5.21 | 5.95 | 2.66 |
| YCL030C | 0.78 | -0.35788 | 0.050508 | 0.103679 | no | down | 334.51 | 366.56 | 380.3 | 259.88 | 233.85 | 321.92 |
| YCL033C | 0.687 | -0.54252 | 7.77E-05 | 0.000381 | no | down | 1723.63 | 1609.02 | 1559.01 | 1122.88 | 1171.04 | 918.23 |
| YCL064C | 1.484 | 0.569603 | 0.100485 | 0.183164 | no | up | 876.41 | 756.45 | 930.6 | 1643.9 | 1730.3 | 524.06 |
| YCR020C | 1.526 | 0.609596 | 0.010492 | 0.028169 | no | up | 15.13 | 14.99 | 21.14 | 30.2 | 25.05 | 21.91 |
| YCR069W | 0.515 | -0.95614 | 4.24E-09 | 5.32E-08 | no | down | 305.62 | 293.34 | 309.54 | 150.88 | 176.21 | 122.8 |
| YCR104W | 1 | 0 | 1 | 1 | no | no change | 1.62 | 2.43 | 0.53 | 3.75 | 1.05 | 1.47 |
| YDL029W | 0.633 | -0.66049 | 2.16E-07 | 1.89E-06 | no | down | 115.99 | 107.31 | 113.27 | 64.63 | 71.77 | 69.48 |
| YDL066W | 0.736 | -0.44269 | 0.049074 | 0.101219 | no | down | 329.46 | 306.2 | 347.67 | 255.12 | 286.96 | 156.68 |
| YDL072C | 0.629 | -0.66821 | 4.55E-07 | 3.76E-06 | no | down | 876.21 | 874.61 | 815.87 | 505.35 | 494.42 | 547.58 |
| YDL104C | 0.849 | -0.23635 | 0.073025 | 0.140367 | no | down | 46.71 | 49.8 | 49.81 | 40.94 | 42.12 | 37.68 |
| YDL126C | 0.938 | -0.09299 | 0.629049 | 0.735822 | no | down | 540.68 | 517.61 | 562.03 | 439.99 | 430.17 | 604 |
| YDL130W-A | 1.211 | 0.276268 | 0.158763 | 0.265456 | no | up | 2046.04 | 2289.93 | 2124.22 | 2161.58 | 2209.73 | 2775.1 |
| YDL170W | 1.676 | 0.744707 | 0.000343 | 0.001436 | no | up | 260.08 | 273.04 | 251.9 | 375.94 | 376.06 | 547.98 |
| YDL181W | 1.416 | 0.501697 | 0.000328 | 0.001375 | no | up | 3430.68 | 3350.44 | 3582.59 | 4698.09 | 4808.67 | 3925.96 |
| YDL207W | 0.756 | -0.40407 | 0.007979 | 0.022115 | no | down | 32.71 | 31.11 | 31.22 | 21.84 | 26.59 | 21.3 |
| YDL210W | 0.572 | -0.8058 | 0.000143 | 0.000658 | no | down | 17.1 | 14.97 | 23.06 | 9.83 | 10.92 | 9.37 |
| YDL238C | 0.545 | -0.87648 | 0.002118 | 0.007051 | no | down | 85.46 | 78.78 | 76.23 | 46.91 | 53.98 | 22.3 |
| YDR019C | 0.582 | -0.78059 | 0.031618 | 0.07054 | no | down | 222.06 | 232.61 | 254.12 | 170.96 | 161.68 | 46.28 |
| YDR024W | 1.318 | 0.398878 | 0.484756 | 0.609861 | no | up | 0.28 | 0 | 2.3 | 1.75 | 0.29 | 2.69 |
| YDR030C | 1.576 | 0.656378 | 0.007561 | 0.021141 | no | up | 19.95 | 21.9 | 19.69 | 26.94 | 26.25 | 43.01 |
| YDR031W | 0.678 | -0.56017 | 4.8E-06 | 3.14E-05 | no | down | 1445.73 | 1384.97 | 1389.01 | 937.41 | 954.41 | 798.81 |
| YDR127W | 0.958 | -0.06141 | 0.651144 | 0.754263 | no | down | 139.74 | 143.02 | 148.6 | 127.34 | 129.56 | 146.12 |
| YDR154C | 0.661 | -0.59725 | 8.02E-05 | 0.000392 | no | down | 3127.41 | 3098.09 | 3110.17 | 1728.35 | 1913.12 | 2103.07 |
| YDR155C | 0.649 | -0.62422 | 5.8E-06 | 3.74E-05 | no | down | 3932.07 | 3822.48 | 3861.45 | 2178.76 | 2415.15 | 2572.62 |
| YDR158W | 1.366 | 0.450027 | 0.062221 | 0.122951 | no | up | 1318.76 | 1328.36 | 1339.99 | 1467.01 | 1450.17 | 2428.37 |
| YDR204W | 0.889 | -0.17 | 0.438884 | 0.568489 | no | down | 346.44 | 341.67 | 333.05 | 248.58 | 246.94 | 378.34 |
| YDR231C | 0.664 | -0.59009 | 0.002888 | 0.009281 | no | down | 831.28 | 796.88 | 743.03 | 583.72 | 558.91 | 365.15 |
| YDR236C | 0.806 | -0.31135 | 0.043829 | 0.092149 | no | down | 99.45 | 92.07 | 96.85 | 79.65 | 81.32 | 63.2 |
| YDR242W | 1.304 | 0.382577 | 0.023906 | 0.05599 | no | up | 18.66 | 21.12 | 22.27 | 26.56 | 23.98 | 28.65 |
| YDR320C-A | 0.583 | -0.77795 | 0.000212 | 0.000937 | no | down | 1614.84 | 1813.49 | 1630.61 | 967.9 | 947.27 | 651.13 |
| YDR340W | 0.461 | -1.11599 | 0.053147 | 0.108151 | no | down | 7.94 | 3.76 | 13.41 | 4.12 | 0 | 0.94 |
| YDR354W | 0.584 | -0.77677 | 7.12E-08 | 6.79E-07 | no | down | 256.43 | 248.4 | 309.88 | 157.71 | 156.86 | 144.08 |
| YDR368W | 0.958 | -0.06238 | 0.599906 | 0.710639 | no | down | 186.31 | 188.97 | 194.21 | 182.53 | 169.7 | 177.1 |
| YDR377W | 0.983 | -0.02482 | 0.891772 | 0.928185 | no | down | 2239.94 | 1959.87 | 2432.83 | 2285.05 | 2212.59 | 1573.28 |
| YDR408C | 0.759 | -0.3982 | 0.020223 | 0.048694 | no | down | 695.56 | 713.05 | 661.86 | 564.63 | 553.91 | 395.35 |
| YDR411C | 0.571 | -0.80866 | 3.77E-11 | 6.48E-10 | no | down | 311.01 | 301.67 | 296 | 163.02 | 163.52 | 174.06 |
| YDR425W | 1.106 | 0.145027 | 0.55379 | 0.672967 | no | up | 38.16 | 37.42 | 40.92 | 37.06 | 32 | 56.44 |
| YDR436W | 0.692 | -0.53216 | 0.011938 | 0.03148 | no | down | 400.35 | 361.91 | 345.19 | 211.31 | 333.07 | 153.22 |
| YDR487C | 1.109 | 0.149146 | 0.347443 | 0.479624 | no | up | 1225.54 | 1234.74 | 1240.51 | 1237 | 1216.84 | 1507.12 |
| YDR502C | 0.672 | -0.57438 | 0.027266 | 0.06234 | no | down | 32.29 | 26.87 | 40.69 | 21.01 | 15.66 | 26.73 |
| YDR512C | 0.638 | -0.64909 | 9.75E-08 | 9.06E-07 | no | down | 397.84 | 372.57 | 369.27 | 235.87 | 247.72 | 213.42 |
| YEL020W-A | 1.769 | 0.82292 | 0.001016 | 0.003744 | no | up | 255.15 | 278.44 | 267.73 | 359.25 | 402.85 | 563.49 |
| YEL023C | 0.646 | -0.62962 | 0.080476 | 0.152637 | no | down | 1.88 | 2 | 2.63 | 0.92 | 0.98 | 1.95 |
| YEL061C | 1.795 | 0.843608 | 0.003761 | 0.011661 | no | up | 1.15 | 0.9 | 0.71 | 1.52 | 1.65 | 1.88 |
| YEL066W | 0.768 | -0.38047 | 0.00648 | 0.018442 | no | down | 270.63 | 275.43 | 313.75 | 212.51 | 224.94 | 196.11 |
| YER032W | 0.698 | -0.51894 | 0.131087 | 0.227325 | no | down | 2.04 | 1.12 | 2.24 | 1.12 | 0.91 | 1.48 |
| YER034W | 0.696 | -0.52243 | 0.017248 | 0.042913 | no | down | 64.5 | 51.29 | 43.01 | 39.26 | 36.11 | 29.72 |
| YER039C | 0.613 | -0.706 | 1.21E-06 | 9.13E-06 | no | down | 149.83 | 147.31 | 128.25 | 81.17 | 83.56 | 85.77 |
| YER042W | 0.643 | -0.63689 | 3.36E-06 | 2.27E-05 | no | down | 163.54 | 140.92 | 158.15 | 96.64 | 95.16 | 92.54 |
| YER055C | 1.836 | 0.876916 | 0.000412 | 0.001684 | no | up | 311.26 | 292.6 | 305.41 | 444.95 | 452.52 | 763.79 |
| YER057C | 1.021 | 0.03014 | 0.833128 | 0.887822 | no | up | 488.09 | 420.23 | 460.72 | 417.29 | 433.74 | 469.36 |
| YER069W | 0.832 | -0.26584 | 0.279016 | 0.40636 | no | down | 240.82 | 242.29 | 253.7 | 162.93 | 156.42 | 269.85 |
| YER141W | 1.336 | 0.41748 | 0.000685 | 0.002626 | no | up | 435.29 | 400 | 424.92 | 585.92 | 570.93 | 490.28 |
| YER147C | 0.657 | -0.60601 | 0.004089 | 0.012544 | no | down | 79.65 | 81.29 | 75.85 | 54.47 | 60.43 | 35.26 |
| YER152C | 0.568 | -0.81662 | 4E-07 | 3.32E-06 | no | down | 304.7 | 299.34 | 259.84 | 165.35 | 176.14 | 132.01 |
| YER185W | 0.971 | -0.04257 | 0.865728 | 0.912147 | no | down | 15.14 | 11.25 | 16.88 | 11.41 | 12.81 | 16.28 |
| YFL014W | 0.785 | -0.34913 | 0.149082 | 0.252124 | no | down | 7899.68 | 7668.05 | 7382.69 | 4148.14 | 5034.94 | 7339.82 |
| YFL021C-A | 0.982 | -0.02579 | 0.868199 | 0.913337 | no | down | 204.38 | 175.87 | 169.35 | 193.33 | 158.92 | 171.02 |
| YFL030W | 0.55 | -0.86181 | 0.000522 | 0.002066 | no | down | 4176.62 | 3772.81 | 3851.5 | 2489.67 | 2411.94 | 1260.74 |
| YFL031W | 1.581 | 0.660805 | 0.000148 | 0.000678 | no | up | 1555.35 | 1385.31 | 1440.14 | 2130.79 | 1985.28 | 2637.62 |
| YFL033C | 0.932 | -0.10147 | 0.66766 | 0.767672 | no | down | 6.73 | 5.41 | 6.2 | 6.48 | 6.5 | 3.75 |
| YFL044C | 1.248 | 0.31988 | 0.011643 | 0.030804 | no | up | 116.24 | 112.49 | 124.1 | 140.44 | 141.02 | 146.9 |
| YFL059W | 0.681 | -0.55517 | 0.166821 | 0.275309 | no | down | 272.77 | 258.61 | 255.14 | 218.45 | 228.45 | 46.63 |
| YFL060C | 0.958 | -0.0626 | 0.773818 | 0.845776 | no | down | 28.53 | 28.87 | 27.76 | 29.89 | 29.8 | 19.47 |
| YFR031C | 0.87 | -0.20124 | 0.584697 | 0.698358 | no | down | 0.56 | 0.65 | 0.77 | 0.8 | 0.39 | 0.45 |
| YFR047C | 0.72 | -0.47424 | 0.003003 | 0.009613 | no | down | 398.06 | 352.37 | 400.02 | 293.44 | 289.35 | 217.6 |
| YGL009C | 1.06 | 0.083584 | 0.809341 | 0.871398 | no | up | 288.43 | 282.1 | 312.68 | 373.77 | 428.92 | 124.55 |
| YGL026C | 0.564 | -0.82685 | 1.19E-08 | 1.34E-07 | no | down | 368.43 | 368.92 | 441.17 | 232.15 | 219.08 | 191.53 |
| YGL113W | 1.215 | 0.281534 | 0.177688 | 0.28894 | no | up | 5.39 | 5.29 | 5.69 | 6.37 | 5.6 | 7.53 |
| YGL116W | 0.767 | -0.38336 | 0.142402 | 0.243094 | no | down | 3.03 | 3.02 | 3.33 | 1.95 | 2.18 | 2.74 |
| YGL121C | 0.761 | -0.39471 | 0.013221 | 0.034252 | no | down | 386.92 | 334.59 | 428.33 | 290.77 | 286.64 | 245.78 |
| YGL126W | 0.585 | -0.77399 | 0.003531 | 0.011051 | no | down | 451.72 | 413.45 | 389.45 | 272.54 | 288.36 | 135.01 |
| YGL187C | 0.954 | -0.06791 | 0.526182 | 0.647977 | no | down | 1619.84 | 1505.63 | 1632.11 | 1461.44 | 1469.76 | 1546.55 |
| YGL223C | 1.165 | 0.220763 | 0.391304 | 0.522425 | no | up | 59.64 | 58.56 | 64.22 | 61.45 | 50.59 | 95.31 |
| YGL242C | 0.897 | -0.15654 | 0.42483 | 0.554244 | no | down | 28.86 | 25.85 | 27.77 | 23.68 | 24.51 | 22.76 |
| YGL259W | 0.724 | -0.46632 | 0.146436 | 0.248465 | no | down | 18.68 | 21.83 | 22.48 | 16.62 | 18.31 | 7.93 |
| YGR008C | 1.177 | 0.234726 | 0.306937 | 0.438175 | no | up | 3629.46 | 3742.17 | 3519.53 | 3199.43 | 3433.43 | 4982.93 |
| YGR032W | 1.845 | 0.883246 | 0.004953 | 0.014772 | no | up | 155.46 | 141.76 | 165.86 | 211.74 | 199.86 | 460.71 |
| YGR037C | 0.533 | -0.90728 | 3.15E-05 | 0.000171 | no | down | 665.9 | 581.44 | 678.73 | 274.83 | 261.74 | 369.72 |
| YGR043C | 0.702 | -0.51039 | 0.031333 | 0.070024 | no | down | 862.29 | 817.94 | 892.72 | 663.6 | 703.44 | 369.22 |
| YGR044C | 0.547 | -0.87165 | 1.12E-06 | 8.5E-06 | no | down | 221.73 | 215.66 | 231.7 | 117.54 | 140.94 | 92.29 |
| YGR066C | 1.702 | 0.767522 | 0.071248 | 0.137451 | no | up | 34.18 | 41.54 | 31.77 | 89.72 | 93.05 | 16.63 |
| YGR086C | 0.51 | -0.97187 | 2.04E-18 | 7.74E-17 | no | down | 3053.47 | 2855.53 | 3040.46 | 1380.48 | 1498.5 | 1465.42 |
| YGR133W | 0.687 | -0.54107 | 0.000167 | 0.000756 | no | down | 234.36 | 226.15 | 209.22 | 162.44 | 147.53 | 131.13 |
| YGR135W | 1.036 | 0.05105 | 0.764386 | 0.83908 | no | up | 92.46 | 95.68 | 87.71 | 89.51 | 82.49 | 104.61 |
| YGR175C | 0.627 | -0.67288 | 0.000617 | 0.002411 | no | down | 132.79 | 128.16 | 164.26 | 77.8 | 76.34 | 101.06 |
| YGR192C | 0.845 | -0.24332 | 0.279551 | 0.406777 | no | down | 5182.76 | 5230.43 | 6252.82 | 5433.22 | 5120.69 | 3095.6 |
| YGR213C | 1.142 | 0.191353 | 0.535814 | 0.656527 | no | up | 11.62 | 11.64 | 8.46 | 8.39 | 10.31 | 16.67 |
| YGR236C | 0.572 | -0.80652 | 0.062307 | 0.12308 | no | down | 2125.01 | 2114.27 | 1867.83 | 399.65 | 520.2 | 1886.91 |
| YGR247W | 0.516 | -0.95458 | 3.43E-09 | 4.32E-08 | no | down | 165.91 | 171.54 | 154.94 | 93.51 | 80.66 | 69.13 |
| YGR254W | 1.215 | 0.28097 | 0.608723 | 0.718269 | no | up | 1250.59 | 1221.38 | 1560.91 | 2541.09 | 2526.48 | 279.17 |
| YGR268C | 1.173 | 0.229655 | 0.0731 | 0.140469 | no | up | 499.42 | 444.72 | 455.69 | 563.25 | 553.32 | 471.38 |
| YHL036W | 1.514 | 0.598299 | 0.00084 | 0.003165 | no | up | 59.65 | 57.41 | 68.84 | 89.58 | 80.69 | 106.06 |
| YHR001W-A | 1.796 | 0.84445 | 6.94E-09 | 8.3E-08 | no | up | 4456.24 | 3967.97 | 4551.13 | 6945.54 | 6668.59 | 7294.3 |
| YHR008C | 0.567 | -0.81791 | 2.38E-12 | 4.78E-11 | no | down | 3495.49 | 3249.17 | 3322.08 | 1933.27 | 1916.27 | 1650.11 |
| YHR018C | 0.749 | -0.4165 | 0.026863 | 0.061618 | no | down | 2822.11 | 2796.08 | 2930.35 | 1838.36 | 1820.01 | 2506.97 |
| YHR029C | 1.072 | 0.099762 | 0.579407 | 0.694338 | no | up | 460.87 | 459.21 | 441.06 | 424.99 | 426.36 | 562.49 |
| YHR063C | 1.131 | 0.178093 | 0.498748 | 0.622038 | no | up | 20.97 | 24.88 | 23.19 | 31.93 | 29.26 | 15.85 |
| YHR087W | 0.558 | -0.84208 | 7.5E-05 | 0.000369 | no | down | 635.39 | 622.14 | 586.95 | 273.22 | 272.85 | 391.56 |
| YHR138C | 1.329 | 0.410697 | 0.048051 | 0.099568 | no | up | 1567.33 | 1588.03 | 1615.39 | 1800.7 | 1695.05 | 2485.93 |
| YHR145C | 1 | 0 | 1 | 1 | no | no change | 0 | 1.12 | 0.61 | 0 | 3.02 | 0.57 |
| YHR174W | 1.216 | 0.281914 | 0.027844 | 0.063525 | no | up | 112.31 | 125.32 | 118.68 | 143.33 | 150 | 130.22 |
| YHR208W | 0.632 | -0.66121 | 0.001352 | 0.004806 | no | down | 683.72 | 680.77 | 996.73 | 534.47 | 498.23 | 399.74 |
| YIL024C | 1 | -0.00011 | 0.999672 | 0.999672 | no | down | 96.34 | 88.04 | 91.9 | 118.08 | 97.91 | 51.87 |
| YIL029C | 0.849 | -0.23637 | 0.227993 | 0.349118 | no | down | 72.68 | 71.71 | 90.91 | 62.25 | 69.68 | 57.68 |
| YIL046W | 0.729 | -0.45573 | 0.000309 | 0.001312 | no | down | 404 | 379.41 | 398.62 | 282.2 | 307.42 | 248.84 |
| YIL051C | 1.248 | 0.319947 | 0.142355 | 0.243094 | no | up | 3641.53 | 3256.54 | 3515.53 | 4804.76 | 4937.58 | 2822.67 |
| YIL059C | 0.935 | -0.09672 | 0.560415 | 0.67755 | no | down | 239.16 | 269.28 | 260.71 | 244.16 | 208.62 | 225.59 |
| YIL060W | 0.895 | -0.16046 | 0.319182 | 0.450689 | no | down | 191.72 | 207.39 | 193.66 | 150.94 | 185.28 | 168.62 |
| YIL062C | 1.238 | 0.307569 | 0.030411 | 0.068324 | no | up | 404.13 | 383.82 | 375.55 | 423.48 | 462.75 | 493.12 |
| YIL074C | 0.921 | -0.11848 | 0.709092 | 0.79785 | no | down | 18.51 | 15.29 | 21.52 | 20 | 21.28 | 8.31 |
| YIL082W | 0.557 | -0.84344 | 0.026666 | 0.061241 | no | down | 32.28 | 24.24 | 26.15 | 17.13 | 19.55 | 4.94 |
| YIL087C | 0.813 | -0.2993 | 0.02599 | 0.059899 | no | down | 1231.37 | 1174.08 | 1131.04 | 883.44 | 876.36 | 978.47 |
| YIL116W | 1.634 | 0.708572 | 0.012137 | 0.031858 | no | up | 186.35 | 197.91 | 208.31 | 249.13 | 241.32 | 476.36 |
| YIL136W | 0.608 | -0.71842 | 2.3E-06 | 1.62E-05 | no | down | 2812.83 | 2456.4 | 2576.27 | 1405.25 | 1480.78 | 1701.84 |
| YIL159W | 1.116 | 0.158943 | 0.643793 | 0.748655 | no | up | 0.67 | 0.48 | 0.51 | 0.44 | 0.8 | 0.58 |
| YIL163C | 0.863 | -0.21239 | 0.433453 | 0.562833 | no | down | 46.68 | 44.04 | 48.85 | 32.06 | 32.84 | 46.65 |
| YIL164C | 0.815 | -0.29428 | 0.05364 | 0.108772 | no | down | 317.23 | 311.57 | 321.22 | 229.84 | 236.4 | 276.67 |
| YIL165C | 0.898 | -0.15601 | 0.498225 | 0.621997 | no | down | 281.4 | 295.9 | 324.53 | 262.4 | 188.95 | 304.76 |
| YIR006C | 0.615 | -0.70031 | 2.86E-08 | 2.99E-07 | no | down | 24.5 | 22.39 | 22.7 | 13.91 | 14.75 | 12.97 |
| YIR019C | 0.603 | -0.73066 | 0.000829 | 0.00313 | no | down | 4.46 | 3.76 | 4.4 | 2.39 | 1.99 | 2.89 |
| YIR030C | 0.85 | -0.23444 | 0.514899 | 0.636677 | no | down | 10.87 | 7.3 | 6.85 | 5.1 | 4.89 | 9.98 |
| YIR034C | 0.967 | -0.0487 | 0.721423 | 0.806664 | no | down | 537.03 | 593.02 | 596.61 | 561.28 | 500.55 | 560.25 |
| YIR036C | 0.534 | -0.90611 | 1.83E-08 | 1.99E-07 | no | down | 707.26 | 639.75 | 674.56 | 324.47 | 312.96 | 392.1 |
| YIR037W | 1.221 | 0.287494 | 0.199572 | 0.316354 | no | up | 1581.82 | 1370.91 | 1463.39 | 1414.49 | 1511.05 | 2245.41 |
| YIR038C | 0.629 | -0.6692 | 0.003609 | 0.011257 | no | down | 872.58 | 837.61 | 801.96 | 415.07 | 417.07 | 658.14 |
| YIR039C | 0.569 | -0.81412 | 3.23E-09 | 4.11E-08 | no | down | 104.53 | 100.59 | 95.39 | 57.5 | 59.66 | 48.37 |
| YJL001W | 0.761 | -0.39309 | 0.073713 | 0.141518 | no | down | 407.86 | 382.56 | 415.97 | 259.39 | 238.4 | 375.75 |
| YJL048C | 1.077 | 0.106697 | 0.616442 | 0.724414 | no | up | 247.16 | 252.85 | 254.63 | 225.59 | 226.59 | 337.12 |
| YJL066C | 1.787 | 0.837333 | 0.003233 | 0.010232 | no | up | 632.79 | 600.78 | 577.66 | 790.08 | 844.26 | 1603.11 |
| YJL067W | 1.789 | 0.839251 | 0.002094 | 0.006978 | no | up | 214.88 | 216.28 | 196.54 | 294.73 | 280.57 | 509.64 |
| YJL077C | 1.3 | 0.378963 | 0.060486 | 0.120232 | no | up | 233.05 | 204.33 | 224.44 | 250.51 | 236.01 | 331.73 |
| YJL121C | 0.749 | -0.41682 | 0.002223 | 0.007363 | no | down | 397.1 | 398.37 | 392.88 | 266.31 | 283.2 | 307.48 |
| YJL163C | 0.504 | -0.9871 | 5.94E-08 | 5.77E-07 | no | down | 458.71 | 434.59 | 414.36 | 204.79 | 259.6 | 169.27 |
| YJL167W | 0.55 | -0.86353 | 1.6E-05 | 9.27E-05 | no | down | 55.71 | 51.53 | 56.64 | 26.13 | 24.56 | 34.86 |
| YJL171C | 1.929 | 0.94762 | 4.02E-06 | 2.68E-05 | no | up | 61.67 | 63.28 | 63.28 | 133.32 | 142.99 | 86.48 |
| YJL199C | 0.884 | -0.17765 | 0.321306 | 0.453285 | no | down | 368.56 | 443.74 | 376.02 | 331.82 | 308.04 | 340.36 |
| YJL201W | 0.886 | -0.17506 | 0.25683 | 0.381846 | no | down | 23.79 | 26.12 | 24.86 | 24.17 | 21.22 | 19.16 |
| YJL210W | 0.564 | -0.82549 | 6.77E-05 | 0.000338 | no | down | 1309.19 | 1257.45 | 1348.84 | 812.49 | 805.25 | 492.54 |
| YJL211C | 0.796 | -0.32968 | 0.182035 | 0.29432 | no | down | 810.26 | 828.43 | 817.14 | 725.29 | 751.09 | 380.74 |
| YJR005C-A | 1 | 0 | 1 | 1 | no | no change | 2.85 | 0 | 1.34 | 1.34 | 0 | 12.15 |
| YJR010W | 0.776 | -0.36549 | 0.086796 | 0.162842 | no | down | 17.64 | 15.24 | 18.46 | 13.82 | 15.24 | 9.53 |
| YJR073C | 0.555 | -0.84903 | 1.32E-05 | 7.74E-05 | no | down | 2208.03 | 2028.01 | 2404.8 | 1057.06 | 1012.21 | 1412.15 |
| YJR074W | 0.586 | -0.77225 | 7.29E-05 | 0.00036 | no | down | 257.07 | 240.17 | 296.38 | 134 | 130.81 | 175.37 |
| YJR085C | 0.943 | -0.08476 | 0.458764 | 0.584929 | no | down | 4051.12 | 3653.63 | 3887.92 | 3556.33 | 3564.11 | 3063.66 |
| YJR099W | 0.755 | -0.40634 | 0.042807 | 0.090414 | no | down | 20.46 | 22.23 | 21.62 | 15.94 | 14.12 | 16.44 |
| YJR142W | 1.232 | 0.301118 | 0.326925 | 0.459374 | no | up | 10.23 | 8.92 | 8.52 | 7.23 | 10.53 | 15.73 |
| YJR150C | 1.037 | 0.052243 | 0.852031 | 0.902069 | no | up | 5.06 | 6.04 | 6.18 | 7.11 | 5.97 | 4.44 |
| YJR152W | 0.77 | -0.37783 | 0.162488 | 0.270118 | no | down | 4.14 | 3.14 | 5.38 | 3.08 | 3.29 | 2.96 |
| YKL001C | 1.706 | 0.770549 | 7.07E-06 | 4.45E-05 | no | up | 29.39 | 26.32 | 30.07 | 49.75 | 45.53 | 47.5 |
| YKL016C | 1.195 | 0.256719 | 0.026978 | 0.061838 | no | up | 1604.2 | 1500.42 | 1654.14 | 1902.19 | 1909.25 | 1667.57 |
| YKL017C | 0.814 | -0.29619 | 0.420996 | 0.551235 | no | down | 1.08 | 1.24 | 2.14 | 0.95 | 1.02 | 1.45 |
| YKL029C | 1.079 | 0.110102 | 0.652835 | 0.755018 | no | up | 147.37 | 136.07 | 153.56 | 180.28 | 187.28 | 96.68 |
| YKL030W | 1.179 | 0.237123 | 0.109538 | 0.196062 | no | up | 114.49 | 118.23 | 124.6 | 150.82 | 137.51 | 119.74 |
| YKL065W-A | 0.513 | -0.96399 | 0.023042 | 0.054407 | no | down | 342.91 | 320.94 | 495.27 | 73.31 | 109.56 | 269.41 |
| YKL068W-A | 1.761 | 0.816799 | 0.039959 | 0.085785 | no | up | 40.62 | 38.93 | 74.15 | 89.63 | 115.42 | 62.95 |
| YKL084W | 0.744 | -0.42753 | 0.007224 | 0.020324 | no | down | 712.15 | 615.71 | 662.61 | 502.6 | 507.39 | 375.32 |
| YKL120W | 1.035 | 0.049329 | 0.819879 | 0.878719 | no | up | 70.1 | 69.07 | 82.65 | 89.95 | 80.92 | 53.32 |
| YKL121W | 0.649 | -0.62297 | 0.00151 | 0.00529 | no | down | 14.59 | 12.96 | 16.98 | 9.66 | 10.78 | 7.47 |
| YKL141W | 0.826 | -0.27555 | 0.056724 | 0.114149 | no | down | 2521.4 | 2345.79 | 2292.74 | 2053.84 | 2054.37 | 1591.97 |
| YKL148C | 0.582 | -0.77974 | 0.004615 | 0.013915 | no | down | 2667.78 | 2704.82 | 2727.45 | 1764.45 | 1883.88 | 826.22 |
| YKL150W | 0.999 | -0.00114 | 0.99387 | 0.996225 | no | down | 1847.56 | 1733.56 | 1738.75 | 1581.64 | 1653.6 | 1914.77 |
| YKL165C | 1.112 | 0.153276 | 0.450103 | 0.577666 | no | up | 5.03 | 5.58 | 5.09 | 5.9 | 4.76 | 6.42 |
| YKL168C | 0.574 | -0.79998 | 0.000125 | 0.000584 | no | down | 42.28 | 41.79 | 35.26 | 21.06 | 27.73 | 17.06 |
| YKL192C | 0.628 | -0.67051 | 2.8E-05 | 0.000155 | no | down | 1933.72 | 1925.85 | 1872.04 | 1240.77 | 1240.65 | 903.35 |
| YKL221W | 0.678 | -0.5596 | 0.077597 | 0.147962 | no | down | 6.61 | 5.93 | 7.21 | 2.46 | 4.28 | 5.78 |
| YKR046C | 0.708 | -0.49747 | 0.016749 | 0.041885 | no | down | 1783.11 | 1634.4 | 1736.79 | 1374.24 | 1320.92 | 820.1 |
| YKR049C | 1.2 | 0.26285 | 0.103826 | 0.188282 | no | up | 1257.19 | 1252.72 | 1317.04 | 1317.69 | 1400.91 | 1644.44 |
| YLL040C | 0.969 | -0.04496 | 0.862422 | 0.910326 | no | down | 4.98 | 4.44 | 4.32 | 3.28 | 3.68 | 5.97 |
| YLL041C | 0.801 | -0.32005 | 0.023391 | 0.055051 | no | down | 3569.25 | 3198.77 | 3667.79 | 2911.17 | 2868.16 | 2310.39 |
| YLL049W | 0.914 | -0.13011 | 0.379504 | 0.511621 | no | down | 140.27 | 160.88 | 147.62 | 135.99 | 127.43 | 130.81 |
| YLL057C | 0.698 | -0.51948 | 0.003387 | 0.010659 | no | down | 25.83 | 28.72 | 23.74 | 16.45 | 19.95 | 16.39 |
| YLL058W | 0.942 | -0.0855 | 0.675195 | 0.773571 | no | down | 7.51 | 7.8 | 8.65 | 6.87 | 6.61 | 8.43 |
| YLR004C | 0.877 | -0.18857 | 0.661812 | 0.763038 | no | down | 57.63 | 58.76 | 51.72 | 57.25 | 73.61 | 10.92 |
| YLR038C | 1.551 | 0.633365 | 2.41E-06 | 1.69E-05 | no | up | 5099.92 | 4713.18 | 5183.81 | 6848.34 | 7115.26 | 7692.97 |
| YLR039C | 0.969 | -0.04604 | 0.902522 | 0.935998 | no | down | 0.98 | 0.66 | 0.74 | 0.54 | 0.58 | 1.09 |
| YLR043C | 0.51 | -0.9701 | 4.74E-06 | 3.11E-05 | no | down | 393.83 | 352.09 | 512.74 | 193.93 | 181.86 | 206.55 |
| YLR058C | 0.771 | -0.37462 | 0.364233 | 0.495773 | no | down | 504.13 | 526.71 | 576 | 533.9 | 531.08 | 101.12 |
| YLR092W | 0.644 | -0.63486 | 1.62E-05 | 9.35E-05 | no | down | 29.08 | 25 | 30.24 | 16.76 | 17.73 | 18.05 |
| YLR154C | 0.512 | -0.96638 | 8.77E-07 | 6.85E-06 | no | down | 207.23 | 156.84 | 174.83 | 92.88 | 89.29 | 70.41 |
| YLR179C | 0.588 | -0.76619 | 0.001846 | 0.006273 | no | down | 258.97 | 253.57 | 323.03 | 119.83 | 136.6 | 202.51 |
| YLR180W | 0.572 | -0.80555 | 0.054776 | 0.110686 | no | down | 127.71 | 132.82 | 228.23 | 137.34 | 85.09 | 25.2 |
| YLR194C | 1.041 | 0.057892 | 0.738299 | 0.818798 | no | up | 433.95 | 408.41 | 399.04 | 365.73 | 397.45 | 486.32 |
| YLR231C | 0.518 | -0.94987 | 5.85E-09 | 7.06E-08 | no | down | 67.28 | 68.02 | 69.23 | 39.6 | 34.04 | 28.28 |
| YLR279W | 0.675 | -0.56777 | 0.00504 | 0.014996 | no | down | 148.15 | 149.49 | 150.78 | 92.39 | 78.74 | 110.52 |
| YLR281C | 0.585 | -0.77423 | 9.68E-07 | 7.47E-06 | no | down | 574.72 | 496.56 | 551.56 | 259.58 | 318.65 | 318.01 |
| YLR290C | 1.289 | 0.366202 | 0.139612 | 0.239167 | no | up | 133.29 | 132.35 | 138.17 | 136.61 | 139.24 | 232.42 |
| YLR353W | 0.506 | -0.98324 | 4.89E-06 | 3.19E-05 | no | down | 8.23 | 8.14 | 10.39 | 4.26 | 3.82 | 4.74 |
| YLR355C | 1.005 | 0.006753 | 0.986984 | 0.991828 | no | up | 1489.22 | 1571.43 | 1927.52 | 1993.96 | 2503.98 | 437.82 |
| YLR356W | 0.703 | -0.50919 | 1.09E-05 | 6.63E-05 | no | down | 1441.02 | 1356.13 | 1345.5 | 895.89 | 948.7 | 950.19 |
| YLR359W | 0.785 | -0.34984 | 0.149987 | 0.253251 | no | down | 60.77 | 55.08 | 62.11 | 53.74 | 52.83 | 28.55 |
| YLR364W | 0.768 | -0.38088 | 0.18958 | 0.303624 | no | down | 70.38 | 37.51 | 56.6 | 41.95 | 35.21 | 37.62 |
| YLR395C | 1.753 | 0.809594 | 3.73E-10 | 5.56E-09 | no | up | 1700.36 | 1556.6 | 1620.97 | 2652.71 | 2687.84 | 2338.69 |
| YLR408C | 0.714 | -0.48678 | 0.023096 | 0.054514 | no | down | 144.67 | 123.54 | 113.51 | 91.7 | 98.66 | 64.11 |
| YLR410W-A | 1.409 | 0.495101 | 0.063187 | 0.124394 | no | up | 14.76 | 14.52 | 8.77 | 22.11 | 17.14 | 14.22 |
| YLR414C | 0.599 | -0.7399 | 0.000328 | 0.001374 | no | down | 534.77 | 624.58 | 468.86 | 280.22 | 277.04 | 369.51 |
| YML078W | 1.049 | 0.069124 | 0.632214 | 0.738865 | no | up | 1285.24 | 1252.81 | 1364.06 | 1415.71 | 1429.5 | 1107.08 |
| YML079W | 0.806 | -0.3108 | 0.013303 | 0.034435 | no | down | 226.9 | 209.91 | 213.19 | 165.37 | 181.37 | 157.82 |
| YML083C | 0.546 | -0.87279 | 0.011062 | 0.029435 | no | down | 11 | 12.56 | 11.02 | 8.18 | 6.6 | 2.6 |
| YMR009W | 0.952 | -0.07081 | 0.623036 | 0.73054 | no | down | 265.18 | 256.53 | 241.78 | 283.32 | 267.84 | 370.87 |
| YMR015C | 0.806 | -0.31124 | 0.3961 | 0.52717 | no | down | 3.42 | 4.17 | 6.23 | 2.46 | 2.58 | 5.42 |
| YMR032W | 0.501 | -0.99573 | 0.005573 | 0.016299 | no | down | 2.99 | 1.73 | 1.54 | 1.17 | 0.81 | 0.83 |
| YMR034C | 0.686 | -0.54352 | 0.000377 | 0.001555 | no | down | 149.82 | 156.42 | 153.45 | 114.49 | 106.51 | 84.78 |
| YMR035W | 0.708 | -0.49841 | 0.00022 | 0.000969 | no | down | 229.94 | 238.33 | 235.22 | 149.99 | 163.78 | 162.64 |
| YMR041C | 0.857 | -0.22242 | 0.384952 | 0.516552 | no | down | 110.05 | 128.6 | 124.06 | 71.18 | 92.51 | 134.42 |
| YMR046C | 0.731 | -0.45204 | 0.002251 | 0.007438 | no | down | 47.25 | 48.3 | 41.84 | 32 | 35.15 | 30.33 |
| YMR056C | 1.029 | 0.041837 | 0.700283 | 0.791434 | no | up | 635.77 | 599.8 | 638.45 | 632.46 | 658.15 | 584.55 |
| YMR062C | 1.923 | 0.943167 | 0.001043 | 0.003831 | no | up | 2593.15 | 2608.22 | 2458.36 | 3730.41 | 3716.77 | 7459.09 |
| YMR072W | 0.805 | -0.31304 | 0.226833 | 0.34824 | no | down | 167.21 | 157.15 | 133.85 | 96.69 | 92.29 | 159.33 |
| YMR086W | 0.896 | -0.15815 | 0.368594 | 0.500421 | no | down | 35.32 | 36.1 | 33.73 | 29.03 | 26.86 | 35.63 |
| YMR087W | 0.561 | -0.8329 | 6.96E-07 | 5.57E-06 | no | down | 66.03 | 63.31 | 55.65 | 30.53 | 37.44 | 31.62 |
| YMR090W | 1.109 | 0.149423 | 0.380895 | 0.512734 | no | up | 723.98 | 725.17 | 745.87 | 727.18 | 703.23 | 916.74 |
| YMR105C | 0.532 | -0.90929 | 3.52E-11 | 6.09E-10 | no | down | 580.89 | 582.88 | 534.75 | 270.7 | 290.96 | 311.13 |
| YMR110C | 1.024 | 0.03364 | 0.889634 | 0.926568 | no | up | 365.66 | 363.5 | 354.07 | 277.58 | 310.73 | 487.58 |
| YMR174C | 1.894 | 0.921082 | 0.007831 | 0.021762 | no | up | 2678.71 | 2108.88 | 2304.46 | 2514.06 | 2973.38 | 6647.56 |
| YMR175W | 1.271 | 0.345658 | 0.214571 | 0.333713 | no | up | 5902.27 | 5102.89 | 5203.81 | 4864.57 | 4696.28 | 8902.31 |
| YMR189W | 0.605 | -0.72463 | 0.072981 | 0.140325 | no | down | 40.95 | 42.32 | 50.94 | 32.9 | 33.85 | 6.96 |
| YMR265C | 1.046 | 0.065447 | 0.6309 | 0.73758 | no | up | 164.54 | 176.37 | 162.62 | 180.92 | 183.51 | 150.18 |
| YMR271C | 1.254 | 0.326719 | 0.106848 | 0.192333 | no | up | 356.95 | 347.73 | 357.09 | 370.62 | 382.71 | 538.56 |
| YMR281W | 1.261 | 0.334064 | 0.037819 | 0.081987 | no | up | 76.13 | 84.84 | 71.39 | 101.79 | 102.8 | 82 |
| YMR297W | 0.836 | -0.25825 | 0.221746 | 0.341932 | no | down | 3898.62 | 3721.78 | 3885.13 | 2592.49 | 2721.86 | 3954.32 |
| YMR317W | 1.028 | 0.040426 | 0.880725 | 0.920141 | no | up | 26.16 | 26.58 | 24.29 | 30.47 | 32.41 | 15.04 |
| YNL015W | 0.698 | -0.5194 | 0.013744 | 0.035416 | no | down | 671.14 | 653.8 | 673.51 | 354.54 | 403.68 | 457.21 |
| YNL018C | 1.149 | 0.200682 | 0.385226 | 0.51681 | no | up | 11.46 | 10.16 | 12.53 | 11.89 | 10.31 | 16.09 |
| YNL036W | 0.879 | -0.18591 | 0.644575 | 0.749152 | no | down | 2185.47 | 2338.23 | 1987.65 | 2712.67 | 2251.02 | 519.82 |
| YNL046W | 1.085 | 0.117793 | 0.572759 | 0.688243 | no | up | 220.06 | 199.61 | 178.89 | 174.69 | 193.56 | 254.48 |
| YNL050C | 1.507 | 0.591723 | 0.040716 | 0.08703 | no | up | 13.88 | 7.94 | 14.32 | 18.41 | 14.21 | 21.4 |
| YNL052W | 1.181 | 0.239522 | 0.219437 | 0.339121 | no | up | 3873.84 | 3607.02 | 4140.81 | 5195.45 | 4805.42 | 3239.94 |
| YNL055C | 0.961 | -0.0567 | 0.609542 | 0.719101 | no | down | 11454.8 | 10705.69 | 10917.45 | 10999.71 | 10138.09 | 9720.81 |
| YNL059C | 0.832 | -0.26611 | 0.360504 | 0.491751 | no | down | 2.57 | 2.09 | 2.45 | 1.87 | 1.38 | 2.41 |
| YNL071W | 0.851 | -0.23331 | 0.037206 | 0.080956 | no | down | 700.65 | 660.77 | 661.15 | 548.8 | 555.37 | 568.41 |
| YNL103W-A | 1 | 0 | 1 | 1 | no | no change | 0 | 0 | 0 | 0 | 0 | 0 |
| YNL115C | 0.779 | -0.35973 | 0.093532 | 0.172772 | no | down | 97.55 | 87.15 | 90.96 | 55.01 | 64.52 | 87.16 |
| YNL135C | 1.375 | 0.459095 | 0.000359 | 0.001493 | no | up | 2262 | 2266.94 | 2287.91 | 2857.67 | 2898.36 | 3066.77 |
| YNL172W | 0.74 | -0.4344 | 0.182188 | 0.294437 | no | down | 1.97 | 1.96 | 1.7 | 1.19 | 0.79 | 1.94 |
| YNL195C | 0.541 | -0.88692 | 3.36E-07 | 2.83E-06 | no | down | 2924.14 | 3005.2 | 2533.03 | 1305.48 | 1751.53 | 1323 |
| YNL200C | 0.873 | -0.19546 | 0.291272 | 0.419969 | no | down | 1444.62 | 1283.89 | 1279.32 | 989.07 | 1030.58 | 1344.71 |
| YNL206C | 1.738 | 0.797717 | 1.53E-05 | 8.89E-05 | no | up | 14.44 | 17.03 | 13.43 | 28.42 | 23.35 | 25.47 |
| YNL208W | 1.057 | 0.080623 | 0.687162 | 0.782037 | no | up | 6314.9 | 6290.82 | 6114.47 | 5483.29 | 5648.53 | 7917.29 |
| YNL220W | 0.621 | -0.68762 | 0.015751 | 0.03975 | no | down | 145.67 | 158.52 | 155.31 | 118.34 | 103.4 | 48.91 |
| YNL241C | 0.641 | -0.64153 | 1.44E-07 | 1.29E-06 | no | down | 2301.92 | 2277.26 | 2421.81 | 1507.53 | 1557.53 | 1291.85 |
| YNL245C | 1.135 | 0.183087 | 0.385728 | 0.51708 | no | up | 133.36 | 134.93 | 139.88 | 181.71 | 161.1 | 107.15 |
| YNL276C | 0.546 | -0.8739 | 0.01211 | 0.031813 | no | down | 32.81 | 31.88 | 53.19 | 24.63 | 11.5 | 20.99 |
| YNL277W | 0.739 | -0.43577 | 0.012032 | 0.031671 | no | down | 119.42 | 114.61 | 151.11 | 98.14 | 98.94 | 78.66 |
| YNL332W | 0.704 | -0.50534 | 0.049352 | 0.101568 | no | down | 6.27 | 5.87 | 6.1 | 4.27 | 3.52 | 4.49 |
| YNL333W | 0.662 | -0.595 | 0.11775 | 0.208411 | no | down | 187.42 | 197.83 | 223.88 | 175.52 | 156.25 | 41.49 |
| YNL334C | 0.711 | -0.49253 | 0.085163 | 0.160204 | no | down | 26.84 | 26.3 | 32.96 | 22.56 | 24.1 | 11.38 |
| YNR034W-A | 1.818 | 0.862737 | 4.29E-05 | 0.000226 | no | up | 2050.73 | 2071.04 | 2235.95 | 3277.46 | 3107.97 | 4492.43 |
| YNR059W | 1.127 | 0.173083 | 0.442695 | 0.572248 | no | up | 6.48 | 8.51 | 6.39 | 6.85 | 7.9 | 8.86 |
| YNR065C | 0.944 | -0.08315 | 0.778164 | 0.849503 | no | down | 4.27 | 3.83 | 5.35 | 2.85 | 3.62 | 5.78 |
| YOL052C-A | 0.972 | -0.04093 | 0.852041 | 0.902069 | no | down | 8946.34 | 8831.64 | 8395.6 | 5755.86 | 6624.82 | 8544.1 |
| YOL055C | 0.951 | -0.07198 | 0.803263 | 0.867798 | no | down | 8.78 | 9.88 | 8.13 | 9.03 | 11.05 | 4.86 |
| YOL058W | 0.639 | -0.64583 | 3.12E-05 | 0.00017 | no | down | 1395.22 | 1529.81 | 1500.82 | 884.37 | 824.99 | 1016.42 |
| YOL064C | 1.581 | 0.660761 | 4.97E-05 | 0.000256 | no | up | 50.72 | 50.59 | 59.06 | 74.47 | 84.03 | 89.91 |
| YOL077W-A | 1.249 | 0.321291 | 0.102179 | 0.185914 | no | up | 3799.72 | 3831.92 | 3848.51 | 4989.09 | 4438.29 | 3144.92 |
| YOL091W | 1.72 | 0.782332 | 0.040458 | 0.086664 | no | up | 2.67 | 2.9 | 2.82 | 3.43 | 3.05 | 8.57 |
| YOL110W | 0.778 | -0.36137 | 0.030824 | 0.069033 | no | down | 915.96 | 833.1 | 941.83 | 706.87 | 776.3 | 538.61 |
| YOL129W | 0.778 | -0.36299 | 0.000968 | 0.003581 | no | down | 1137.81 | 1100.84 | 1078.95 | 804.64 | 860.69 | 812.22 |
| YOL147C | 0.518 | -0.94869 | 6.76E-15 | 1.87E-13 | no | down | 716.93 | 729.66 | 675.13 | 354.69 | 377.65 | 325.15 |
| YOL162W | 0.905 | -0.14322 | 0.705544 | 0.79568 | no | down | 9.48 | 9.37 | 13.47 | 5.52 | 7.33 | 14.72 |
| YOL164W | 0.619 | -0.69154 | 3.05E-05 | 0.000167 | no | down | 90.45 | 90.98 | 97.35 | 65.37 | 55.95 | 45.71 |
| YOR003W | 0.589 | -0.7643 | 6.04E-06 | 3.87E-05 | no | down | 58.1 | 53.39 | 53.22 | 30.41 | 36.97 | 26.2 |
| YOR064C | 1.229 | 0.297576 | 0.023651 | 0.055557 | no | up | 168.54 | 158.84 | 158.19 | 188.3 | 212.73 | 177.97 |
| YOR073W | 0.78 | -0.35855 | 0.146435 | 0.248465 | no | down | 7.16 | 7.65 | 6.79 | 6.26 | 6.37 | 3.69 |
| YOR122C | 1.041 | 0.057947 | 0.758032 | 0.83363 | no | up | 1672.01 | 1764.28 | 1777.07 | 1580.54 | 1504.98 | 2036.4 |
| YOR126C | 0.668 | -0.58213 | 0.0058 | 0.016826 | no | down | 132.69 | 119.98 | 123.27 | 85.44 | 98.03 | 57.16 |
| YOR128C | 0.749 | -0.41782 | 0.044476 | 0.09335 | no | down | 31.32 | 25.75 | 28.92 | 23.73 | 23.41 | 15.21 |
| YOR154W | 0.966 | -0.05018 | 0.836791 | 0.890379 | no | down | 23.12 | 35.06 | 36.69 | 21.7 | 28.01 | 24.64 |
| YOR185C | 0.583 | -0.77721 | 3.62E-07 | 3.03E-06 | no | down | 637.6 | 560.46 | 548.46 | 300.18 | 316.61 | 356.46 |
| YOR202W | 1.123 | 0.167314 | 0.207867 | 0.326163 | no | up | 510.5 | 574 | 586.24 | 612.5 | 592.99 | 611.46 |
| YOR215C | 0.848 | -0.23718 | 0.070216 | 0.13579 | no | down | 237.44 | 240.11 | 227.18 | 191.39 | 185.17 | 197.53 |
| YOR222W | 0.77 | -0.37735 | 0.013913 | 0.035823 | no | down | 645.44 | 695.11 | 652.58 | 533.42 | 502.28 | 597.04 |
| YOR230W | 0.745 | -0.42392 | 0.011371 | 0.030198 | no | down | 1771.26 | 1698.8 | 1694.34 | 1097.12 | 1176.28 | 1440.6 |
| YOR271C | 0.647 | -0.6272 | 0.038159 | 0.082548 | no | down | 78.86 | 66.87 | 80.25 | 56.77 | 58.12 | 23.38 |
| YOR289W | 0.773 | -0.37145 | 0.014516 | 0.037149 | no | down | 834.36 | 798.86 | 776.16 | 546.49 | 576.62 | 668.99 |
| YOR350C | 1.392 | 0.476751 | 0.013522 | 0.034901 | no | up | 18.62 | 16.19 | 20.06 | 20.95 | 25.34 | 28.68 |
| YOR362C | 0.725 | -0.46404 | 0.000159 | 0.000725 | no | down | 871.49 | 859.36 | 934.94 | 619.1 | 600.05 | 647.47 |
| YOR391C | 0.68 | -0.55736 | 0.002063 | 0.006894 | no | down | 42.3 | 40.76 | 46.42 | 24.56 | 31.61 | 28.16 |
| YPL048W | 0.941 | -0.08827 | 0.583795 | 0.698068 | no | down | 1966.63 | 1943.41 | 1932.39 | 1645.29 | 1635.23 | 2046.19 |
| YPL109C | 1.451 | 0.537135 | 8.24E-05 | 0.000401 | no | up | 324.44 | 327.12 | 319.34 | 504.42 | 497.97 | 399.5 |
| YPL123C | 0.552 | -0.85851 | 1.45E-07 | 1.3E-06 | no | down | 40.93 | 43.38 | 37.26 | 19.84 | 21.6 | 23 |
| YPL149W | 1.012 | 0.017063 | 0.910684 | 0.941693 | no | up | 138.93 | 135.09 | 129.28 | 120.22 | 129.77 | 145.52 |
| YPL196W | 1.136 | 0.184562 | 0.38037 | 0.512571 | no | up | 250.47 | 238.02 | 225.89 | 215.46 | 241.76 | 330.35 |
| YPL245W | 1.297 | 0.375195 | 0.246096 | 0.370048 | no | up | 9.92 | 7.84 | 12.98 | 10.03 | 9.85 | 19.53 |
| YPL256C | 0.535 | -0.90107 | 0.000176 | 0.000791 | no | down | 5.24 | 5.99 | 4.87 | 2.72 | 2.44 | 2.99 |
| YPR028W | 0.893 | -0.16346 | 0.383812 | 0.515755 | no | down | 1567.21 | 1367.5 | 1603.62 | 1175.03 | 1147.22 | 1546.45 |
| YPR047W | 0.648 | -0.62562 | 0.005598 | 0.016344 | no | down | 5.72 | 5.35 | 6.3 | 3.67 | 3.8 | 3.3 |
| YPR058W | 0.534 | -0.90445 | 2.34E-10 | 3.61E-09 | no | down | 291.54 | 284.02 | 304.39 | 166.03 | 158.69 | 128.34 |
| YPR059C | 0.767 | -0.3826 | 0.132925 | 0.229697 | no | down | 71.74 | 62.82 | 98.56 | 54.57 | 67.42 | 45.11 |
| YPR138C | 0.835 | -0.25998 | 0.317845 | 0.449601 | no | down | 151.68 | 151.05 | 165.93 | 157.49 | 147.25 | 74.9 |
| YPR172W | 0.551 | -0.86104 | 0.00025 | 0.001085 | no | down | 523.22 | 433.79 | 410.6 | 238.59 | 308.17 | 163.3 |
| YPR191W | 1.014 | 0.019845 | 0.908525 | 0.940074 | no | up | 1818.16 | 1618.49 | 1796.34 | 2004.12 | 1809.88 | 1360.93 |
| YPR197C | 1.088 | 0.12151 | 0.587747 | 0.70134 | no | up | 28.8 | 41.75 | 39.84 | 42.86 | 39.65 | 34.1 |
| YPR198W | 1.286 | 0.363426 | 0.036654 | 0.080001 | no | up | 80.56 | 76.9 | 73.59 | 89.43 | 87.96 | 113.25 |
