# Supplementary material for: Hydrogen sulfide treatment at the late growth stage of Saccharomyces cerevisiae extends chronological lifespan
Source: Aging (Albany NY). 2021 Mar 19;13(7):9859–73. doi: 10.18632/aging.202738 (PMC8064171; doi:10.18632/aging.202738)
Supplement: Supplementary Table 6 [file aging-13-202738-s007.doc]

**Supplementary Table 6. Antioxidant DEGs induced by the early and late NaHS treatments.**

Early Treatment antioxidant DEGs

| Gene_id | Gene name | FC(NaHS/Control) | Log2FC(NaHS/Control) | Pvalue | Padjust | Significant | Regulate | Control1_Fpkm | Control2_Fpkm | Control3_Fpkm | NaHS1_Fpkm | NaHS2_Fpkm | NaHS3_Fpkm |
| --- | --- | --- | --- | --- | --- | --- | --- | --- | --- | --- | --- | --- | --- |
| YCL035C | GRX1 | 2.274 | 1.185447 | 4.26E-05 | 0.000225 | yes | up | 1225.55 | 1155.68 | 1110.77 | 1956.55 | 1965.13 | 3873.17 |
| YBR244W | GPX2 | 2.143 | 1.099687 | 1.65E-05 | 9.47E-05 | yes | up | 12.38 | 10.93 | 11.95 | 22.41 | 24.95 | 27.86 |
| YHR055C | CUP1-2 | 15.183 | 3.924412 | 7.93E-31 | 9.86E-29 | yes | up | 2546.33 | 4771.31 | 2030.68 | 62502.73 | 64744.93 | 34272.64 |
| YHR106W | TRR2 | 0.429 | -1.22105 | 8.89E-14 | 2.13E-12 | yes | down | 336.9 | 335.15 | 313.45 | 142.48 | 153.66 | 108.85 |
| YDR513W | GRX2 | 0.499 | -1.00234 | 9.72E-09 | 1.12E-07 | yes | down | 1509.6 | 1408.8 | 1444.17 | 761.74 | 763.82 | 523.01 |
| YDR256C | CTA1 | 0.314 | -1.66958 | 2.29E-05 | 0.000128 | yes | down | 1156.81 | 1212.04 | 1013.41 | 288.11 | 491.17 | 103.19 |
| YGR088W | CTT1 | 0.075 | -3.72999 | 1.69E-56 | 1.19E-53 | yes | down | 998.24 | 849.67 | 1032.35 | 69.33 | 78.37 | 41.32 |

Late Treatment antioxidant DEGs

| Gene_id | Gene name | FC(NaHS/Control) | Log2FC(NaHS/Control) | Pvalue | Padjust | Significant | Regulate | Control1_Fpkm | Control2_Fpkm | Control3_Fpkm | NaHS1_Fpkm | NaHS2_Fpkm | NaHS3_Fpkm |
| --- | --- | --- | --- | --- | --- | --- | --- | --- | --- | --- | --- | --- | --- |
| YBR244W | GPX2 | 3.618 | 1.855 | 6.29E-08 | 1.16E-06 | yes | up | 23.611 | 18.396 | 13.144 | 47.65 | 107.142 | 68.335 |
| YDR353W | TRR1 | 2.201 | 1.138 | 3.66E-12 | 1.11E-10 | yes | up | 340.78 | 382.14 | 311.59 | 694.4 | 875.356 | 781.472 |
| YNL241C | ZWF1 | 0.314 | -1.67 | 2.92E-06 | 3.92E-05 | yes | down | 354.979 | 1224.251 | 769.144 | 262.719 | 227.468 | 183.191 |
| YER042W | MXR1 | 0.308 | -1.697 | 1.09E-09 | 2.60E-08 | yes | down | 76.686 | 64.804 | 60.566 | 17.965 | 26.774 | 14.618 |
| YHR008C | SOD2 | 0.315 | -1.666 | 7.12E-18 | 3.53E-16 | yes | down | 567.419 | 770.473 | 601.126 | 205.05 | 216.489 | 179.75 |
| YGR088W | CTT1 | 0.073 | -3.782 | 3.72E-43 | 8.41E-41 | yes | down | 34.518 | 41.286 | 62.741 | 2.813 | 3.284 | 3.003 |
| YHR106W | TRR2 | 0.378 | -1.405 | 3.88E-08 | 7.42E-07 | yes | down | 51.042 | 81.132 | 51.724 | 21.694 | 27.105 | 18.985 |
