# Supplementary material for: Hydrogen sulfide treatment at the late growth stage of Saccharomyces cerevisiae extends chronological lifespan
Source: Aging (Albany NY). 2021 Mar 19;13(7):9859–73. doi: 10.18632/aging.202738 (PMC8064171; doi:10.18632/aging.202738)
Supplement: Supplementary Table 7 [file aging-13-202738-s008.doc]

**Supplementary Table 7. HSP DEGs induced by the early and late NaHS treatments.**

| Gene_id | Gene name | FC(NaHS/Control) | Log2FC(NaHS/Control) | Pvalue | Padjust | Significant | Regulate | Control1_Fpkm | Control2_Fpkm | Control3_Fpkm | NaHS1_Fpkm | NaHS2_Fpkm | NaHS3_Fpkm |
| --- | --- | --- | --- | --- | --- | --- | --- | --- | --- | --- | --- | --- | --- |
| YLL026W | HSP104 | 3.518 | 1.814899 | 3.6E-11 | 6.2E-10 | yes | up | 405.33 | 352.61 | 361.03 | 1080.92 | 1049.53 | 2002.92 |
| YPL240C | HSP82 | 4.756 | 2.249824 | 1.13E-22 | 6.7E-21 | yes | up | 261.45 | 232.97 | 235.54 | 1000.03 | 1013.29 | 1589.12 |
| YMR186W | HSC82 | 2.751 | 1.460195 | 2.56E-12 | 5.1E-11 | yes | up | 179.78 | 162.02 | 178.82 | 405.01 | 427.96 | 608.07 |
| YOR027W | STI1 | 3.471 | 1.795216 | 3.33E-12 | 6.48E-11 | yes | up | 309.94 | 266.61 | 265.61 | 799.5 | 819.32 | 1414.61 |
| YBR155W | CNS1 | 2.962 | 1.566774 | 9.16E-16 | 2.72E-14 | yes | up | 7.04 | 6.08 | 6.56 | 21.16 | 17.55 | 19.82 |
| YLR216C | CPR6 | 6.262 | 2.646596 | 2.06E-59 | 1.86E-56 | yes | up | 122.42 | 92.72 | 98.45 | 633.59 | 649.3 | 696.89 |
| YPL106C | SSE1 | 4.579 | 2.195139 | 1.51E-51 | 7.97E-49 | yes | up | 204.93 | 205.68 | 221.51 | 975.64 | 866.8 | 1047.23 |
| YBR169C | SSE2 | 3.067 | 1.616731 | 1.62E-42 | 3.95E-40 | yes | up | 108.2 | 94.73 | 101.18 | 301.11 | 317.09 | 302.35 |
| YER103W | SSA4 | 2.282 | 1.190199 | 1.09E-16 | 3.6E-15 | yes | up | 108.92 | 109.97 | 120 | 385.23 | 411.79 | 443.8 |
| YBR101C | FES1 | 5.765 | 2.527205 | 6.09E-28 | 5.77E-26 | yes | up | 336.11 | 313.9 | 313.61 | 1619.2 | 1638.04 | 2603.94 |
| YNL064C | YDJ1 | 4.111 | 2.039646 | 9.28E-36 | 1.51E-33 | yes | up | 78.21 | 71.39 | 72.15 | 272.84 | 293.07 | 345.68 |
| YNL007C | SIS1 | 4.313 | 2.108784 | 4.03E-21 | 2.02E-19 | yes | up | 494.47 | 415.43 | 417.35 | 1638.47 | 1644.69 | 2531.36 |
| YNL077W | APJ1 | 3.495 | 1.805163 | 1.41E-10 | 2.26E-09 | yes | up | 344.27 | 320.43 | 323.62 | 881.31 | 961.91 | 1791.87 |
| YJR097W | JJJ3 | 3.238 | 1.695027 | 4.9E-05 | 0.000254 | yes | up | 2.49 | 2.54 | 1.01 | 7.41 | 9.33 | 6.85 |
| YMR161W | HLJ1 | 2.776 | 1.472954 | 7.26E-26 | 5.98E-24 | yes | up | 79.59 | 87.13 | 86.02 | 224.31 | 252.14 | 213.67 |
| YDR171W | HSP42 | 3.503 | 1.808683 | 1.26E-12 | 2.67E-11 | yes | up | 1121.64 | 1020.93 | 951.02 | 2925 | 3091.28 | 5192.61 |
| YJL034W | KAR2 | 5.289 | 2.402959 | 1.84E-49 | 8.99E-47 | yes | up | 139.87 | 122.93 | 127.69 | 605.9 | 696.74 | 777.67 |
| YDR258C | HSP78 | 4.014 | 2.004966 | 1.88E-11 | 3.34E-10 | yes | up | 89.29 | 80.4 | 79.77 | 273.58 | 255.19 | 550.52 |
| YOR232W | MGE1 | 2.479 | 1.309702 | 3.48E-10 | 5.22E-09 | yes | up | 79.62 | 69.81 | 79.93 | 165.02 | 165.75 | 234.06 |
| YFL016C | MDJ1 | 3.779 | 1.917906 | 7.25E-21 | 3.44E-19 | yes | up | 162.28 | 153.03 | 158.14 | 510.59 | 542.95 | 761.31 |
| YLR259C | HSP60 | 2.428 | 1.279906 | 4.66E-24 | 3.08E-22 | yes | up | 1077.37 | 987.27 | 990.76 | 2345.13 | 2389.45 | 2569.09 |
| YOR020C | HSP10 | 2.701 | 1.433577 | 4.91E-22 | 2.74E-20 | yes | up | 1826.47 | 1726.75 | 1665.06 | 4235.47 | 4202.26 | 4887.03 |
| YDL229W | SSB1 | 0.389 | -1.36082 | 1.28E-05 | 7.58E-05 | yes | down | 161.84 | 180.9 | 266.11 | 107.85 | 61.38 | 44.85 |
| YNL209W | SSB2 | 0.393 | -1.34812 | 7.06E-09 | 8.43E-08 | yes | down | 523.98 | 539.15 | 678.88 | 282.04 | 204.13 | 156.07 |
| YAL058W | CNE1 | 0.456 | -1.13396 | 1.08E-07 | 9.96E-07 | yes | down | 33.02 | 34.21 | 39.12 | 17.21 | 17.82 | 11.01 |

**Supplementary Table 7.** HSPs late treatment

| Gene_id | Gene name | FC(NaHS/Control) | Log2FC(NaHS/Control) | Pvalue | Padjust | Significant | Regulate | Control1_Fpkm | Control2_Fpkm | Control3_Fpkm | NaHS1_Fpkm | NaHS2_Fpkm | NaHS3_Fpkm |
| --- | --- | --- | --- | --- | --- | --- | --- | --- | --- | --- | --- | --- | --- |
| YLL026W | HSP104 | 4.039 | 2.014 | 1.64E-07 | 2.83E-06 | yes | up | 28.043 | 9.629 | 36.058 | 87.336 | 122.673 | 148.448 |
| YER103W | SSA4 | 3.967 | 1.988 | 1.69E-13 | 6.04E-12 | yes | up | 60.554 | 42.431 | 56.768 | 147.969 | 285.681 | 265.93 |
| YDR258C | HSP78 | 249 | 7.96 | 9.01E-58 | 4.24E-55 | yes | up | 0 | 0 | 0 | 13.329 | 25.515 | 22.059 |
| YOR232W | MGE1 | 10.375 | 3.375 | 2.19E-11 | 6.12E-10 | yes | up | 1.031 | 1.034 | 0.762 | 21.357 | 21.162 | 5.619 |
| YFL016C | MDJ1 | 3.673 | 1.877 | 0.001273 | 0.008255 | yes | up | 32.675 | 4.211 | 22.42 | 84.532 | 128.502 | 157.243 |
| YPL240C | HSP82 | 2.556 | 1.354 | 1.83E-13 | 6.52E-12 | yes | up | 68.689 | 59.633 | 75.282 | 155.587 | 198.65 | 191.67 |
| YNL077W | APJ1 | 2.683 | 1.424 | 1.07E-12 | 3.54E-11 | yes | up | 56.487 | 76.071 | 70.445 | 153.801 | 222.016 | 196.282 |
| YBR169C | SSE2 | 2.908 | 1.54 | 2.76E-11 | 7.59E-10 | yes | up | 21.896 | 15.564 | 22.025 | 49.585 | 62.699 | 72.641 |
| YBR101C | FES1 | 2.086 | 1.061 | 1E-09 | 2.43E-08 | yes | up | 148.166 | 140.567 | 159.494 | 286.385 | 311.158 | 368.794 |
| YDR171W | HSP42 | 2.512 | 1.329 | 3.97E-09 | 8.84E-08 | yes | up | 156.592 | 160.712 | 172.548 | 391.471 | 340.146 | 562.51 |
| YJL034W | KAR2 | 2.44 | 1.287 | 1.26E-08 | 2.66E-07 | yes | up | 148.357 | 112.555 | 159.573 | 304.453 | 341.717 | 440 |
| YLR216C | CPR6 | 2.145 | 1.101 | 3.18E-06 | 4.21E-05 | yes | up | 68.233 | 49.265 | 62.158 | 98.926 | 152.863 | 155.37 |
| YBR072W | HSP26 | 0.222 | -2.173 | 2.13E-22 | 1.38E-20 | yes | down | 1465.241 | 2311.419 | 2272.354 | 426.727 | 465.97 | 401.409 |
| YFL014W | HSP12 | 0.139 | -2.849 | 1.29E-16 | 5.92E-15 | yes | down | 657.457 | 858.093 | 1320.87 | 61.034 | 152.267 | 118.48 |
