# Supplementary material for: Hydrogen sulfide treatment at the late growth stage of Saccharomyces cerevisiae extends chronological lifespan
Source: Aging (Albany NY). 2021 Mar 19;13(7):9859–73. doi: 10.18632/aging.202738 (PMC8064171; doi:10.18632/aging.202738)
Supplement: Supplementary Tables 8-10 [file aging-13-202738-s009.pdf]

SUPPLEMENTARY TABLES

Supplementary Table 8. Yeast strains.

Strain	Genotype	Source
BY4742	*MATa his3-Δ1 leu2-Δ0 ura3-Δ0 lys2-Δ0*	Lab stock
BY4741	*MATa his3-Δ1 leu2-Δ0 ura3-Δ0 met15-Δ0*	Lab stock
YMR104C	*MATa ypk2::KAN his3-Δ1 leu2-Δ0 ura3-Δ0 met15-Δ0*	Saccharomyces Genome Deletion Project
RCD490	*MATa ypk2::KAN his3-Δ1 leu2-Δ0 ura3-Δ0 lys2-Δ0*	Gift (Dr. Robert Dickson)

Supplementary Table 9. Composition of SDC.

Component	Concentration	Component	Concentration
YNB	1.8 g/L	Trp	80 mg/L
NaH_2PO_4	1.82 g/L	Glu	100 mg/L
$(NH_4)_2SO_4$	5.0 g/L	Arg	40 mg/L
Tyr	40 mg/L	Met	80 mg/L
Adenine	80 mg/L	Ile	60 mg/L
Leu	120 mg/L	Val	150 mg/L
Asp	100 mg/L	Phe	60 mg/L
Ser	400 mg/L	Lys	60 mg/L
Thr	200 mg/L	His	80 mg/L
Ura	40 mg/L	D-glucose	20 g/L or 5 g/L

Supplementary Table 10. RT-qPCR primers.

ACT1-F	CGTTCCAATTTACGCTGGTT
ACT1-R	AGCGGTTTGCATTTCTTGTT
Hsp104-F	GGCCATCAAGCAACAAGCTC
Hsp104-R	GCGGTCTTACCGATACCTGG
Hsp78-F	CGCGCGACGGTAAATTAGAC
Hsp78-R	GCGCCAGCAATCAAAGATCC
YPK2-F	TGAGATGATGACAGGGCTGC
YPK2-R	CTGTACCGTTAACGCCGAGT
GPX2-F	TAATGTTGCCTCCAAGTGCG
GPX2-R	GGTTCCTGCTTCCCGAACTG
